# Tunably strained metallacycles enable modular differentiation of aza-arene C–H bonds

**DOI:** 10.1038/s41467-023-39753-2

**Published:** 2023-07-06

**Authors:** Longlong Xi, Minyan Wang, Yong Liang, Yue Zhao, Zhuangzhi Shi

**Affiliations:** State Key Laboratory of Coordination Chemistry, Chemistry and Biomedicine Innovation Center (ChemBIC), School of Chemistry and Chemical Engineering, Nanjing University, Nanjing, China

**Keywords:** Homogeneous catalysis, Synthetic chemistry methodology, Density functional theory

## Abstract

The precise activation of C–H bonds will eventually provide chemists with transformative methods to access complex molecular architectures. Current approaches to selective C–H activation relying on directing groups are effective for the generation of five-membered, six-membered and even larger ring metallacycles but show narrow applicability to generate three- and four-membered rings bearing high ring strain. Furthermore, the identification of distinct small intermediates remains unsolved. Here, we developed a strategy to control the size of strained metallacycles in the rhodium-catalysed C−H activation of aza-arenes and applied this discovery to tunably incorporate the alkynes into their azine and benzene skeletons. By merging the rhodium catalyst with a bipyridine-type ligand, a three-membered metallacycle was obtained in the catalytic cycle, while utilizing an NHC ligand favours the generation of the four-membered metallacycle. The generality of this method was demonstrated with a range of aza-arenes, such as quinoline, benzo[*f*]quinolone, phenanthridine, 4,7-phenanthroline, 1,7-phenanthroline and acridine. Mechanistic studies revealed the origin of the ligand-controlled regiodivergence in the strained metallacycles.

## Abstract (web summary)

The ability to selectively functionalize different sites on simple starting materials is a constant pursuit in organic chemistry. Here, the authors report a catalytic system to regioselectively differentiate and alkynylate different positions on azaarenes via rhodium catalysis.

## Introduction

**Fig. 1:**
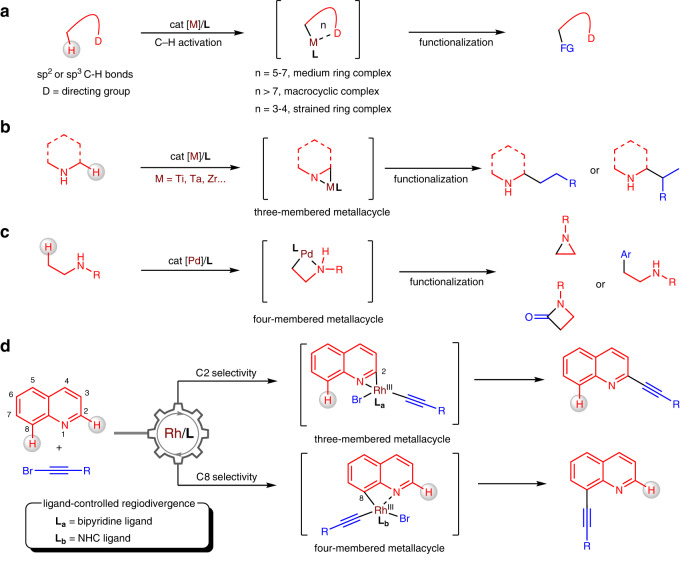
Background and discovery. **a** General procedures for selective C–H activation assisted by directing groups. **b** α-Selective C–H functionalization of aliphatic amines via metallaaziridine intermediates. **c** β-Selective C–H functionalization of aliphatic amines via four-membered metallacycles. **d** Modular differentiation of quinolines by tunable three- and four-membered ring cyclometallation.

Due to the near universal advantage of C − H bonds in organic molecules, the C − H activation strategy provides an opportunity to functionalize any carbon centre in an atom-economical and streamlined way^1–10^. Because organic molecules typically contain multiple C − H bonds with comparable strengths and steric environments, regiocontrol has been a long-standing challenge within this type of chemistry^11^. The differentiation of C − H bonds is traditionally dominated by steric and electronic effects^12^, and there have been considerable efforts to utilize directing groups in less constrained molecules (Fig. 1a)^13–17^. Directed C − H activation is thermodynamically favoured through conformationally rigid five-, six-, and seven-membered metallacyclic intermediates. Several elegant methods have been exploited to achieve the *meta*- and *para*-selective C − H activation of arenes through chelation-assisted macrocyclic complexes by directing groups^18–22^. Despite these advances, the generation of highly strained metallacycles in directed C − H activation remains much desired but more challenging.

Substantial progress has been made regarding the C–H activation of aliphatic amines through strained metallacycles, allowing for site-selective functionalization at the α or β position of the amino group in one catalytic step^23,24^. Early transition metals, including titanium, tantalum and zirconium, enable the hydroaminomethylation of alkenes with unprotected N-heterocycles and amines through metallaaziridine intermediates, affording either linear or branched products (Fig. 1b)^25–30^. A series of C–H functionalizations of aliphatic amines have been developed by palladium catalysis, showing β-selectivity through a four-membered ring cyclopalladation pathway (Fig. 1c)^31–33^. Compared to small aliphatic metallacycles, the formation of benzo-fused analogues comes with larger ring strain. We questioned whether such metallacycles could be generated through chelation with inherent nitrogen atoms in aza-arenes. Furthermore, switching the ring size in a unifying system to differentiate of two C–H bonds would likely have a broad impact on the continued advancement of this field.

As a representative example of bicyclic aza-arenes, quinoline contains seven C − H bonds in the pyridine (C2 to C4) and benzene (C5 to C8) cores that can be functionalized^34,35^. Structural modification of the pyridine ring has been achieved by taking advantage of strong electronic and steric bias^36–43^. An indirect route to access the benzene core involves the oxidation of quinoline to its *N*-oxide, followed by oxygen-directed C − H functionalization and subsequent reduction to the desired products^44–46^. To provide more flexibility to access the benzene core, metal clusters and dimers such as Os_3_(CO)_10_^47^, Ru_3_(CO)_12_^48^, and Rh_2_(OAc)_4_^49^ have been employed in C − H activation through bridged metal-metal bonds. Enabled by bimetallic palladium catalysis, Yu and coworkers also designed a class of remote-directing template for differentiation of C − H bonds (C5-C7) at the benzene core of quinolines^50–53^.

Here, we report the successful realization of a strategy for regiodivergent C–H activation of aza-arenes enabled by tunably strained metallacycles under monomeric rhodium catalysts (Fig. 1d). The precise differentiation of two C–H bonds proceeds through a switchable three- or four-membered-ring cyclometallation pathway by just tuning the structure of the ligands. Incorporation of alkyne motifs into aza-arenes are valuable transformations to build C–C bonds and provide a versatile handle for further modifications.

## Results

### Reaction design

**Fig. 2:**
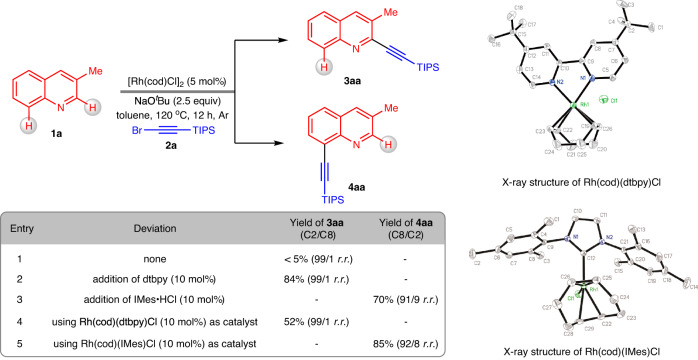
Effect of the ligands on reactivity and regioselectivity. The regioselectivity ratio (r.r.) was determined by GC‒MS analysis of the reaction mixtures. dtbpy = 4,4’-di-tert-butyl-2,2’-bipyridine. IMes =1,3-bis (2,4,6-trimethylphenyl)−1,3-dihydro-2H-imidazol-2-ylidene.

As shown in Fig. 2, we first evaluated the reaction of 3-methylquinoline (**1a**) with (bromoethynyl)triisopropylsilane (**2a**). After treatment of [Rh(cod)Cl]_2_ (5 mol%) with NaO^*t*^Bu (2.5 equiv) at 120 °C under an Ar atmosphere in toluene, the desired product **3aa** was generated in trace amounts after 12 h (entry 1). After screening a variety of ligands for optimization (for the details of the reaction optimization, see the Supplementary Information), the optimized conditions for C2-alkynylation were determined, using 10 mol% dtbpy as a ligand. Under these conditions, product **3aa** was afforded in 84% yield, showing 99/1 *r.r*. of the C2 to C8 positions (entry 2). Interestingly, employing NHC ligands^54^ resulted in a preference for C8 selectivity, and the use of IMes·HCl led to the formation of product **4aa** in 70% yield (C8/C2 = 91/9 *r.r*.) (entry 3). Employment of [Rh(cod)Cl]_2_ with the optimal ligands resulted in the production of two rhodium complexes, Rh(cod)(dtbpy)Cl and Rh(cod)(IMes)Cl, both of which were unambiguously assigned by X-ray crystallography. Utilizing the pregenerated Rh(cod)(dtbpy)Cl as the catalyst showed a much lower reactivity than the in situ generation of the catalyst (entry 4). In contrast, the use of Rh(cod)(IMes)Cl as the catalyst improved the generation of **4aa** to 85% yield with 92/8 *r.r*. (entry 5).

### Scope of the methodology

**Fig. 3:**
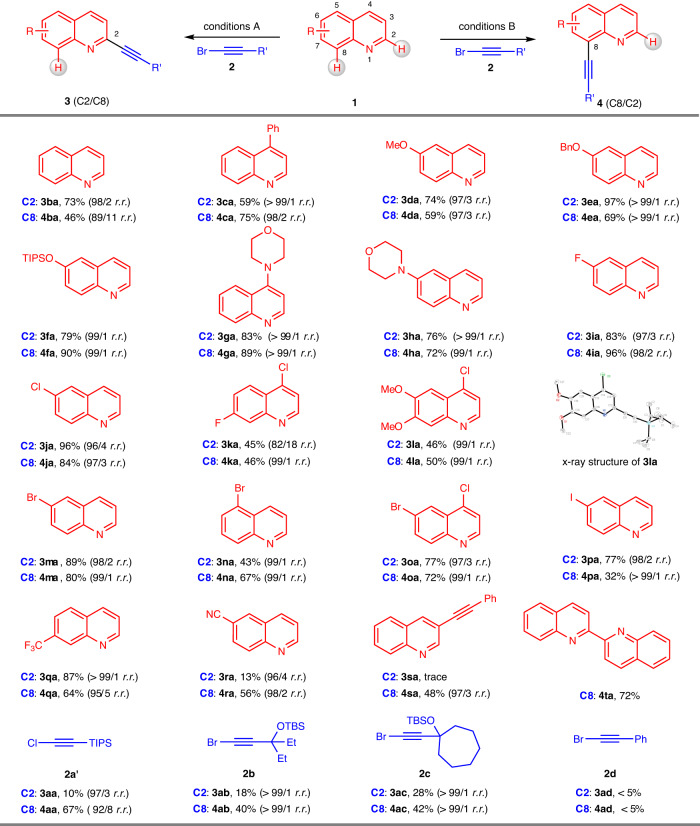
Substrate scope of the regiodivergent C–H alkynylation of quinolines at the C2 and C8 positions. Reaction Conditions A: [Rh(cod)Cl]_2_ (5 mol%), dtbpy (10 mol%), **1** (0.20 mmol, 1.0 equiv), **2** (0.50 mmol, 2.5 equiv), NaO^*t*^Bu (0.50 mmol, 2.5 equiv), 1 mL of toluene, 120 °C, 12 h, under argon. Reaction Conditions B: [Rh(cod)(IMes)Cl (10 mol%), **1** (0.20 mmol, 1.0 equiv), **2** (0.50 mmol, 2.5 equiv), NaO^*t*^Bu (0.50 mmol, 2.5 equiv), 1 mL of toluene, 120 °C, 12 h, under argon. r.r. values were determined by GC‒MS analysis of the reaction mixture.

The scope of the regiodivergent C–H alkynylation was then examined (Fig. 3). In general, a series of commercially available quinolines with varied substituents reacted smoothly with bromoalkyne **2a**, yielding two types of alkynylation products under reaction conditions A and B. The reaction of quinoline (**1b**) with bromoalkyne **2a** underwent C2- and C8-selective C–H alkynylation, providing products **3ba** (98/2 *r.r*.) and **4ba** (89/11 *r.r*.) with excellent regioselectivities. The quinolines that incorporated electron-donating groups, including phenyl (**1c**), ether (**1d-e**), silyloxy (**1** **f**), and amino (**1g-h**) groups, at different positions were readily tolerated with bromoalkyne **2a** to produce the corresponding products **3ca**-**ha** and **4ca**-**ha** with excellent regioselectivity under both reaction conditions. Halide-containing quinolines **1i-p** bearing F, Cl, Br, and even I motifs were compatible with the generation of both the C2 and C8 alkynylation products. Among them, substrates **1k**-**l** containing substituents at the C7 position did not sterically block the reaction at the C8 position. Quinoline **1q** bearing a CF_3_ group was also compatible with both sets of reaction conditions, affording **3qa** and **4qa** in 87% and 64% yields, respectively. The use of 6-cyanoquinoline (**1r**) under condition A became too sluggish to provide product **3ra**, but this substrate did not interfere with productive C–H alkynylation at the C8 position. Quinoline **1** **s**, containing a phenylethynyl motif at the C3 position inhibited the reactivity at the C2 position but enabled C8-selective C–H alkynylation to generate product **4sa**. As a bidentate nitrogen ligand, 2,2′-biquinoline (**1t**) could also engage under reaction conditions B to deliver the expected products **4ta** in a good yield. We further explored the scope of alkynyl bromides with quinoline **1a**. Compared to reagent **2a**, the reaction of TIPS-protected ethynyl chloride (**2a’**) showed much lower reactivity in both reaction systems. Alkynylation with propargyl silyl ethers **2b-c** afforded the desired products in significantly lower yields, but maintained the excellent regioselectivity. Alkynyl bromides **2d** with a less sterically hindered phenyl group were problematic under both reaction conditions, and the corresponding products **3ad** and **4ad** were observed in only trace amounts. The poor outcomes are attributed to the lower stability of these bromoalkynes without TIPs group under the reaction conditions^55–59^.

**Fig. 4:**
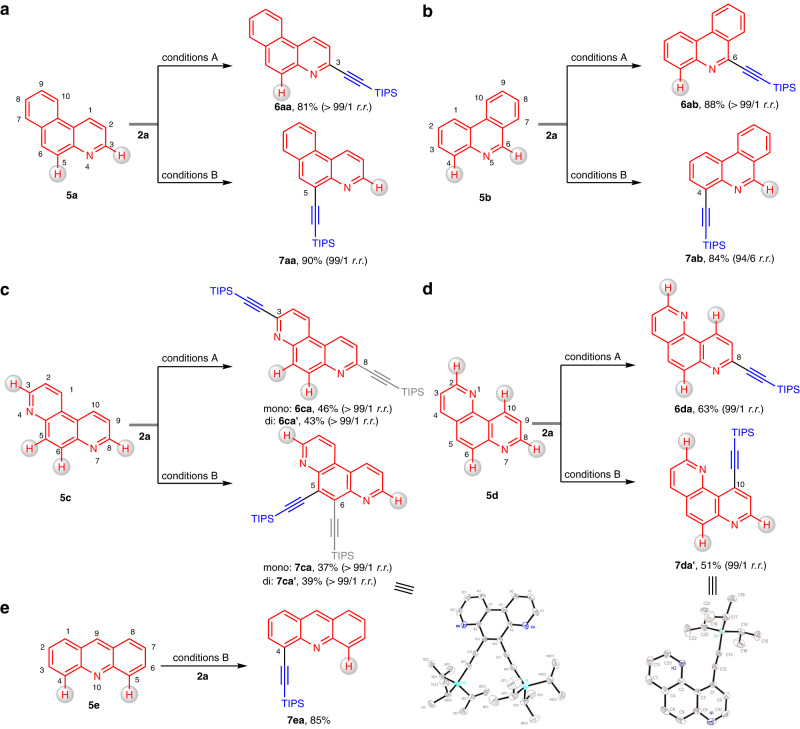
Regiodivergent C–H alkynylation of diverse aza-arenes. **a** C–H alkynylation of benzo[*f*]quinolone (**5a**). **b** C–H alkynylation of phenanthridine (**5b**). **c** C–H Alkynylation of 4,7-phenanthroline (**5c**). **d** C–H alkynylation of 1,7-phenanthroline (**5d**). **e** C–H alkynylation of acridine (**5e**).

Other aza-arenes commonly featured in bioactive molecules and functional materials were next targeted. Benzo[*f*]quinoline (**5a**) was first employed in the catalytic systems and gave desired products **6aa** and **7aa** at the C3 and C5 positions, respectively (Fig. 4a). Phenanthridine (**5b**), the key aromatic unit of many DNA stains, underwent regiodivergent C–H alkynylation at the C6 and C4 positions in high yields with excellent selectivities (Fig. 4b). Treatment of 4,7-phenanthroline (**5c**) containing two azine motifs under reaction conditions A afforded a promising mixture of mono- and di-substituted products **6ca** and

**6ca’** with a high regioselectivity (Fig. 4c). Similarly, the two products **7ca** and **7ca’** were also generated under reaction conditions B with excellent regioselectivity. Notably, the products with one or two alkynyl motifs could be separated by column chromatography on silica. When 1,7-phenanthroline (**5d**) was treated under reaction conditions A, C–H alkynylation occurred at the C8 position. However, an unusual C10-functionalized product **7da’** was obtained under reaction conditions B, indicating that C–H metalation process preferred to undergo five-membered metallacyclic intermediate (Fig. 4d). Finally, acridine (**5e**), bearing two C–H bonds (C4 and C5) for activation, gave only the monosubstituted product **7ea** in 85% yield with complete selectivity (Fig. 4e).

### Synthetic applications

**Fig. 5:**
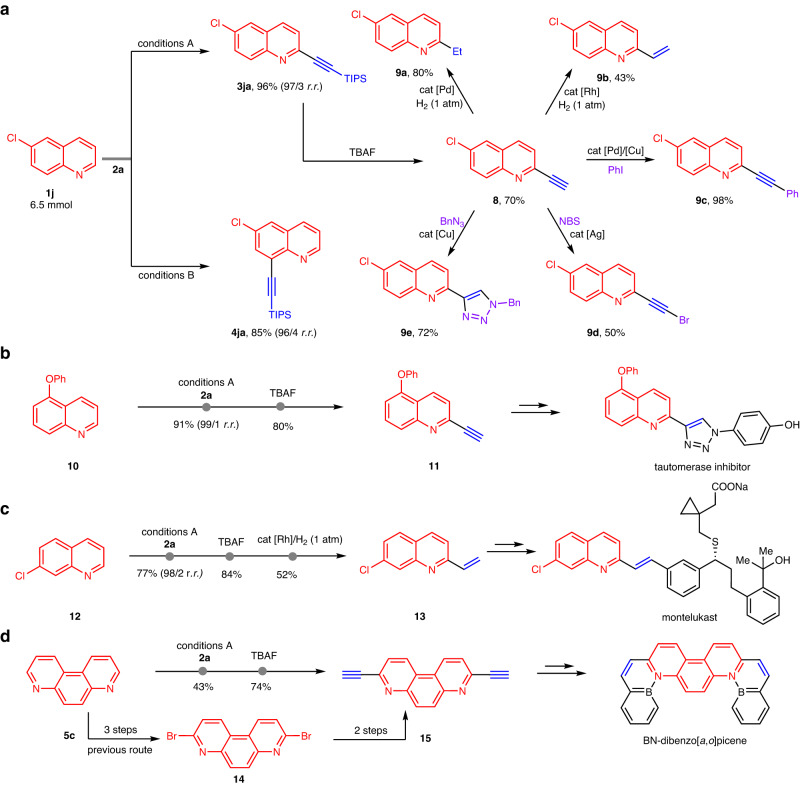
Synthetic applications of regiodivergent C–H alkynylation. **a** Gram-scale preparation of **3ja** and **4ja** and their derivatizations. **b** Application of the process to the synthesis of potent tautomerase inhibitors. **c** Application of the process to the synthesis of montelukast. **d** Application of the process to the synthesis of BN-dibenzo[a,o]picene.

To showcase the synthetic utility of this discovery, gram-scale reactions of quinolone **1j** were first performed under both reaction conditions, and products **3ja** and **4ja** were isolated in 96% and 85% yields without erosion of the regioselectivity (Fig. 5a). Further derivatizations of **3ja** were achieved with synthetically useful intermediate **8**, which was obtained in 70% yield by the TBAF-mediated removal of the TIPS group. For instance, hydrogenation of **8** under 1 atm of hydrogen with a catalytic amount of Pd/BaCO_3_ yielded alkylation product **9a** in high yield. The use of the Wilkinson catalyst for hydrogenation allowed for the chemoselective synthesis of olefin **9b**. The terminal triple bond in **8** could be effectively transformed into an internal bond to provide product **9c** in nearly quantitative conversion through the Sonogashira reaction. Ag catalysis of the reaction of **8** with NBS formed bromination product **9d** in excellent yield. In addition, alkyne **8** was easily converted to triazole **9e** with BnN_3_ via a copper-catalysed click reaction. Based on diverse alkyne conversion, our strategy provides a simple and distinct way to construct a number of pharmaceutically relevant compounds and materials. The reaction of quinoline **10** under Conditions B furnished C2-alkynylation, and further removal of the TIPS group provided Compound **11** in good yield, acting as a core framework for the tautomerase inhibitor (Fig. 5b). As a key intermediate for the synthesis of montelukast, Compound **13** was rapidly prepared from quinoline **12** by C2-selective C–H alkynylation with bromoalkyne **2a**, further removal of the TIPS group and reduction with the Wilkinson catalyst under 1 atm of hydrogen (Fig. 5c). Compound **15** is a key intermediate in the synthesis of BN-dibenzo[a,o]picene, and 3,8-dibromo-4,7-phenanthroline (**14**) must be pregenerated through multistep synthesis from **5c** to access this molecule by Sonogashira coupling (Fig. 5d)^60^. Notably, this molecule can be prepared in an atom- and step-economic way, further indicating the value of the developed method.

### Mechanistic study

**Fig. 6:**
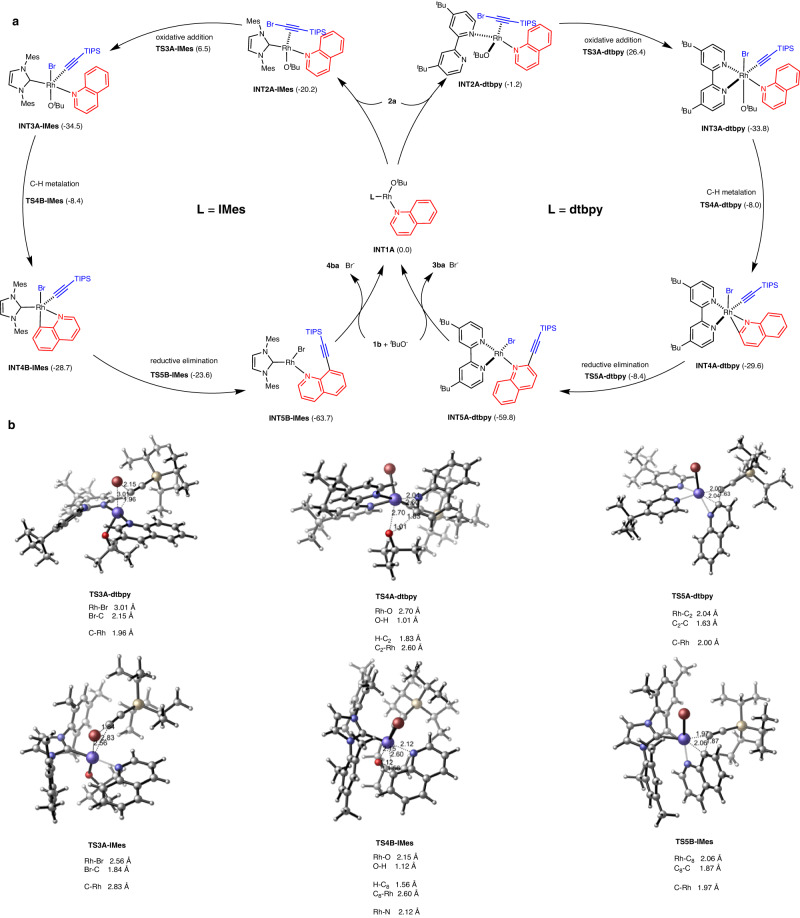
Calculated energy profiles of the regiodivergent C–H alkynylation of 1b with 2a. **a** The proposed catalytic cycles with different ligands. **b** The key transition states in the catalytic cycle.

In order to gain insights into the reaction pathways and origin of positional selectivity, density functional theory (DFT) calculations were performed using the reaction of quinoline **1b** and alkynyl bromide **2a** as the model (Fig. 6). The reaction initially commences by the formation of **1b** and the ligand bound Rh(I) intermediate **INT1A**, which is set as the relative zero point of the Gibbs free energy. The previous reported Rh(I)-involved C–H activation by oxidative addition might be a reversible process with an activation energy barrier of 26.2 kcal mol^−1^ (for the details of the computational data, see the Supplementary Information)^36–38^. Comparably, **INT1A** more favorably coordinates with **2a** to generate **INT2A**, which then undergoes C–Br bond oxidative addition to afford Rh(III) species **INT3A**. In the case of the dtbpy ligand, the C–Br oxidative addition through transition state **TS3A-dtbpy** was calculated to have an activation free energy of 27.6 kcal mol^−1^. This step is the rate-determining step in the catalytic cycle. The resulting intermediate **INT3A-dtbpy** occurs C2–H metalation through a ^*t*^butoxide-assisted deprotonation with a 25.8 kcal mol^−1^ energy barrier^61–66^. Further reductive elimination and ligand exchange yield the desired products with high selectivity and regenerates **INT1A** to complete the catalytic cycle. For the IMes ligand, the C–Br bond oxidative addition to the Rh(I) center from **INT2A-IMes** has an activation energy of 26.7 kcal mol^−1^, which shows a comparable energy barrier with the subsequent C–H activation (26.1 kcal mol^−1^). In the following C − H metalation, IMes ligand favors C8 selective transition state **TS4B-IMes**.

**Fig. 7:**
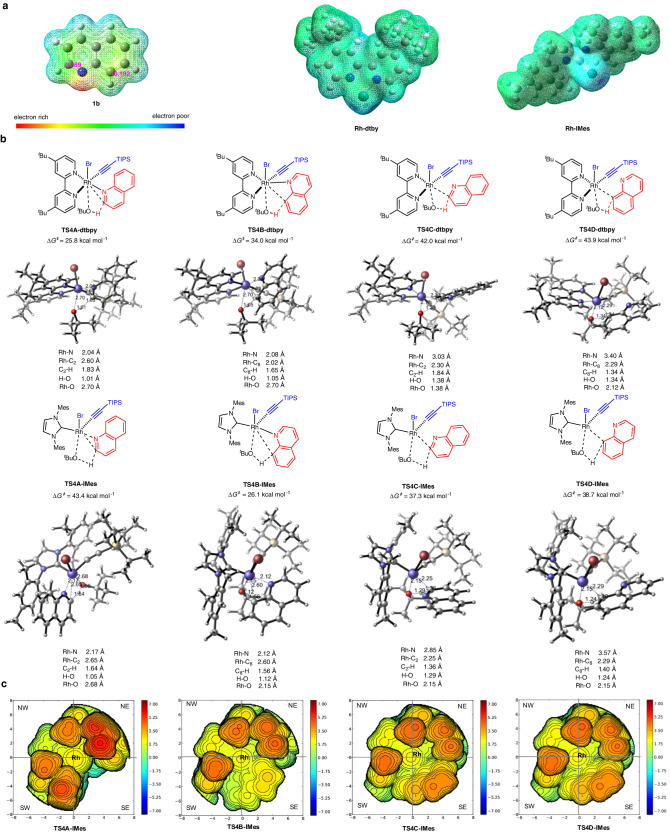
The origin of the site selectivity. **a** Molecular electrostatic potential map of **1b,** **Rh−dtbpy**, and **Rh−IMes**. **b** Key transition states for the different site selectivities. **c** Steric maps of the Rh(III) transition states **TS4A-IMes,** **TS4B-IMes,** **TS4C-IMes**, and **TS4D-IMes**.

As shown in Fig. 7, extensive computational studies on different site selectivity were also carried out. The molecular electrostatic potential map was used to analysis the possible nucleophilic sites of quinoline **1b** (Fig. 7a). Quantitatively, the natural population analysis of **1a** shows that C2 has an atomic charge of −0.069e, which is less charged than C8 (−0.192e), illustrating C2 − H is relative electron deficient and more prone to C − H metallization with electron-rich rhodium catalyst (Supplementary Data 1). Inspecting computed electrostatic potential maps of **Rh**−**dtbpy** and **Rh**−**IMes**, the dtbpy ligand exhibits more electron-donating ability than IMes, allowing **INT3A-dtbpy** to preferentially react with the C2 position of **1b**. The energy barrier of the rhodacycle transition state **TS4A-dtbpy** that delivers the three-membered intermediate **INT4A-dtbpy** is 8.2 kcal mol^−1^ lower than that of the competing transition state **TS4B-dtbpy** that leads to the **INT4B-dtbpy** (Fig. 7b). This energy difference is consistent with the experimentally excellent site selectivity of **3ba**. Without the nitrogen chelation, transition states **TS4C-dtbpy** and **TS4D-dtbpy** have activation barriers of 42.0 and 43.9 kcal mol^−1^, respectively. Such high computed energies are mainly ascribed to the intrinsic inertness of the C − H bonds at the C2 and C8 position of quinoline, indirectly illustrating the importance of the directing group. The directed C–H metalation transition state through **TS4B-IMes** has an activation Gibbs free energy of 26.1 kcal mol^−1^, which is 17.3 kcal mol^−1^ lower than the same process through **TS4A-IMes** (26.1 and 43.4 kcal mol^−1^) and 11.2 kcal mol^−1^ lower than the original nucleophilic attack at quinoline C2 position through **TS4C-IMes** (26.1 and 37.3 kcal mol^−1^). In addition, the steric maps around the Rh(III) centre with IMes ligand were analyzed based on the SambVca 2.1 tool (Fig. 7c)^65,66^. All transition states adopt trigonal bipyramidal geometries, where Br atom and O^*t*^Bu group occupy the two vertex positions and the IMes ligand in the NW quadrant of the steric map extends to the NE and SW quadrants. In disfavored transition state **TS4A-IMes**, bicyclo 3-4 fused rhodacycle enables the Rh−C bond formation to occur in the SW quadrant, which suffers from significant repulsion with Mes group of the carbene ligand. The favored transition state **TS4B-IMes** is a bicyclo 4-4 fused rhodacycle, the expansion of ring size makes the quinoline backbone more horizontally extended and reduce the steric hindrance between quinoline and TIPS group, thereby effectively lowering the energy barrier of **TS4B-IMes**. In **TS4C-IMes** and **TS4D-IMes**, Rh−N bond distances are elongated to 2.85 and 3.57 Å, respectively, indicating nitrogen atom in **1b** is not anchored around the Rh(III) centre. Taken together, the electronic and steric effects account for the tunability of the strained metallacycles, achieving different positional selectivity^67,68^.

## Discussion

In conclusion, the present results demonstrate an important initial advance in C–H activation through a benzo-fused three- or four-membered ring cyclometallation pathway in a switchable mode. This chemistry provides a unique tool for the functionalization of high-value aza-arenes with divergent site selectivities controlled by ligands. The switch of the positional selectivity through strained metallacycles fills a major methodological gap in directed C–H activation. We anticipate that other transformations based on this strategy could be exploited for molecular editing of aza-arene C–H bonds, providing inspiration for the design of new tactics to produce complex bioactive molecules, natural products and functional materials.

## Methods

Due to slight variations in experimental protocols for all the processes we present, we refer the reader to the Supplementary Methods for experimental details.

## Supplementary information

Supplementary Information

Description of Additional Supplementary Files

Supplementary Data 1

Supplementary Data 2

## Data Availability

Crystallographic data for the structures of Rh(cod)(dtbpy)Cl, Rh(cod)(IMes)Cl, **3ia**, **7ca’** and **7da’** reported in this paper have been deposited at the Cambridge Crystallographic Data Centre under deposition numbers CCDC 2192163, 2192164, 2192165, 2192166 and 2192167 (Supplementary Data 2). Copies of the data can be obtained free of charge via www.ccdc.cam.ac.uk/getstructures. All other data supporting the findings of the study, including experimental procedures and compound characterization, are available within the paper and its Supplementary Information, or from the corresponding author upon reasonable request.
