# Supplementary material for: Tunably strained metallacycles enable modular differentiation of aza-arene C–H bonds
Source: Nat Commun. 2023 Jul 6;14:3986. doi: 10.1038/s41467-023-39753-2 (PMC10326034; doi:10.1038/s41467-023-39753-2)
Supplement: Supplementary file 1 — Supplementary Information [file 41467_2023_39753_MOESM1_ESM.pdf]

Supplementary Information for

Tunably strained metallacycles enable modular differentiation of aza-arene C–H bonds

Longlong Xi[1,2], Minyan Wang[1,2], Yong Liang[1], Yue Zhao[1], and Zhuangzhi Shi[1]*

[1]State Key Laboratory of Coordination Chemistry, Chemistry and Biomedicine Innovation Center (ChemBIC), School of Chemistry and Chemical Engineering, Nanjing University, Nanjing 210093 (China)

[2]These authors contributed equally to this work.

*Corresponding author. E-mail: shiz@nju.edu.cn

Table of Contents

1. Supplementary Notes.. 2

2. Supplementary Methods. 2

2.1 General Procedure for Pre-generation of Rhodium Complexes. 2

2.2 Experimental Procedures and Characterization of Products. 4

2.3 Synthetic Applications on Regiodivergent C-H Alkynylation of Benzoazines.......... 28

2.4 Computational Details. 36

2.5 Copies of ^{1}H NMR, ^{13}C NMR and ^{19}F NMR Spectras 43

3. Supplementary References 113

1. Supplementary Notes.

All new compounds were fully characterized. All reactions and manipulations involving air-sensitive compounds were performed using standard Schlenk techniques or in a glovebox. Other reagents and solvents were directly used from the supplier without further purification unless noted. ^{1}H, ^{13}C and ^{19}F NMR spectra were recorded on a Bruker AVANCE III 400 MHz or 500 MHz spectrometer. Chemical shifts (δ values) were reported in ppm with $CDCl_3$ (^{1}H, ^{13}C NMR). ^{1}H NMR spectra data were reported as δ values in ppm relative to chloroform (δ 7.26) if collected in $CDCl_3$. ^{13}C NMR spectra data were reported as δ values in ppm relative to chloroform (δ 77.00) Mass spectra were conducted at Micromass Q-Tof instrument (ESI) and Agilent Technologies 5973N (EI). IR spectra were recorded on a FT-IR spectrometer. Unless otherwise noted, materials obtained from commercial suppliers were used without further purification. The preparation of the **1c, 1e, 1f, 1g, 1h** and **1s** were described according to the literatures[1-4].

2. Supplementary Methods.

2.1 General Procedure for Pre-generation of Rhodium Complexes.

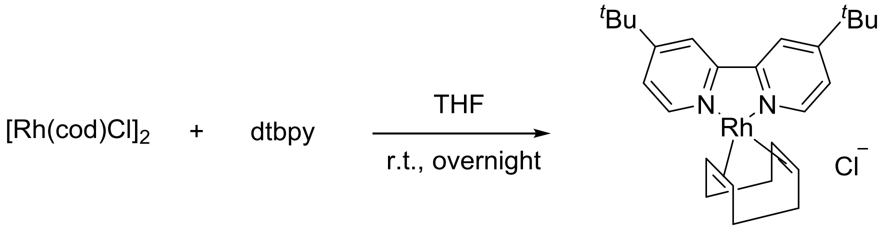

Rh(cod)(dtbpy)Cl

To a 50 mL Schlenk flask containing dtbpy (56.1 mg, 0.209 mmol) and $[Rh(cod)Cl]_2$ (51.5 mg, 0.105 mmol) was added 10 mL anhydrous THF. The reactants dissolved immediately into a red-orange solution. Within seconds, a neon-orange solid precipitated. The mixture was allowed to stir overnight, and the solvent was removed in vacuo, leaving a neon-orange solid (quantitative conversion), m.p. > 230 °C. **^{1}H**

NMR (500 MHz, CDCl$_3$) δ 8.38 (d, *J* = 2.0 Hz, 2H), 7.89 (d, *J* = 5.9 Hz, 2H), 7.63 (dd, *J* = 5.9, 2.0 Hz, 2H), 4.50 (s, 4H), 2.66 – 2.54 (m, 4H), 2.10 (q, *J* = 7.7 Hz, 4H), 1.46 (s, 18H). **^{13}C NMR (126 MHz, CDCl$_3$)** δ 165.8, 156.2, 148.3, 124.5, 120.4, 84.1, 84.0, 36.0, 30.6, 30.34 , 30.32. **ATR-FTIR (cm^{-1})**: 3053, 2982, 1376, 1264, 907, 732, 649. **HRMS m/z (ESI)**: $C_{27}H_{36}N_2Rh^+$ (M - Cl)$^+$ 479.1928, found 479.1928.

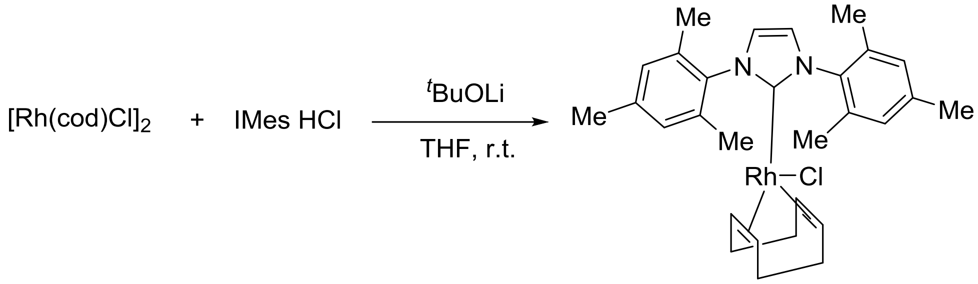

Rh(cod)(IMes)Cl

To a N_2 purged round bottom flask was added lithium tert-butoxide (23.5 mg, 0.295 mmol), $[Rh(cod)Cl]_2$ (60.2 mg, 0.122 mmol), and THF (2 mL). The mixture was vigorously stirred at room temperature for 30 min under nitrogen atmosphere. A solution of NHC ligand 1,3-Bis(2,4,6-trimethylphenyl)imidazolium chloride (83.2 mg, 0.244 mmol) in THF (0.5 mL) was then added to the Rh-containing solution via a cannula. The resulting solution was stirred for additional 5 h at room temperature under nitrogen atmosphere. Solvent was removed in vacuo, and the desired product was isolated by a silica gel column chromatography (PE/EA = 4:1) as a light yellow solid (122 mg, 91%), m.p. = 208.4-210.0 °C. **^{1}H NMR (500 MHz, CDCl$_3$)** δ 7.04 (d, *J* = 27.3 Hz, 4H), 6.95 (s, 2H), 4.52 (s, 2H), 3.30 (s, 2H), 2.40 – 2.39 (m, 12H), 2.11 (s, 6H), 1.92 – 1.78 (m, 4H), 1.55 (d, *J* = 8.8 Hz, 4H). **^{13}C NMR (126 MHz, CDCl$_3$)** δ 183.7, 183.3, 138.6, 137.6, 136.2, 134.3, 129.7, 128.1, 123.5, 96.11, 96.05, 67.9, 67.7, 32.7, 28.4, 21.1, 19.8, 18.1. **ATR-FTIR (cm^{-1})**: 3054, 2985, 1422, 1264, 896, 739, 702. **HRMS m/z (ESI)**: calcd for $C_{29}H_{36}N_2Rh^+$ (M - Cl)$^+$ 515.1928, found 515.1915.

2.2 Experimental Procedures and Characterization of Products.

Supplementary Table 1. Optimization of Reaction Conditions.

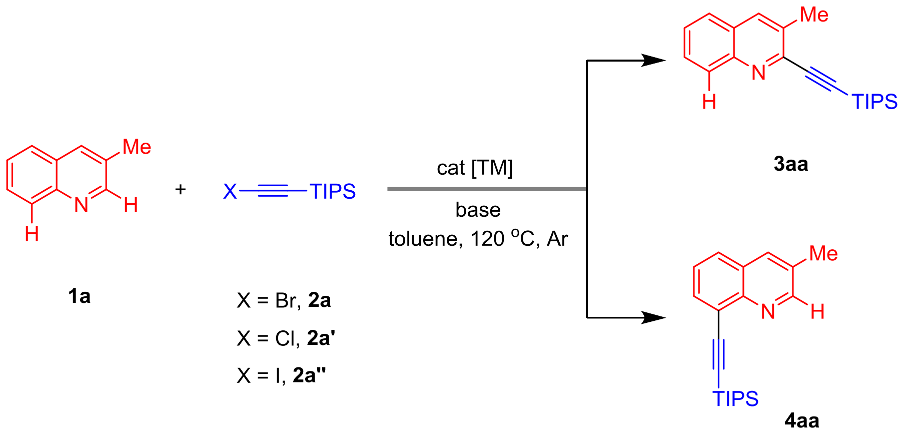

Entry	Cat. [TM] (mol%)	X	Ligand (mol%)	Base (equiv)	T (°C)	Yield (%)[a] 3aa	4aa	Ratio of 3aa/4aa[b]
1	[Rh(cod)Cl]$_2$ (5)	**2a**	-	NaO^{t}Bu (2.5)	120	nd	trace	-
2	[Rh(cod)Cl]$_2$ (5)	**2a**	BINAP (10)	NaO^{t}Bu (2.5)	120	nd	trace	-
3[c]	[Rh(cod)Cl]$_2$ (5)	**2a**	1,10-Phen (10)	NaO^{t}Bu (2.5)	120	33	trace	97/3
4	[Rh(cod)Cl]$_2$ (5)	**2a**	Bpy (10)	NaO^{t}Bu (2.5)	120	38	trace	95/5
**5**	**[Rh(cod)Cl]$_2$ (5)**	**2a**	**dtbpy (10)**	**NaO^{t}Bu (2.5)**	**120**	**84[c]**	trace	**99/1**
6	[Rh(cod)Cl]$_2$ (5)	**2a**	dtbpy (10)	LiO^{t}Bu (2.5)	120	nd	nd	-
7	[Rh(cod)Cl]$_2$ (5)	**2a**	dtbpy (10)	KO^{t}Bu (2.5)	120	nd	nd	-
8	[Rh(OAc)$_2$]$_2$ (5)	**2a**	dtbpy (10)	NaO^{t}Bu (2.5)	120	nd	nd	-
9	[(Cp*RhCl$_2$)]$_2$ (5)	**2a**	dtbpy (10)	NaO^{t}Bu (2.5)	120	nd	nd	-
10	[Rh(cod)Cl]$_2$ (5)	**2a**	dtbpy (10)	NaO^{t}Bu (2.5)	100	35	trace	98/2
11	[Rh(cod)Cl]$_2$ (5)	**2a**	ICy·HCl (10)	NaO^{t}Bu (2.5)	120	trace	20	23/77
12	[Rh(cod)Cl]$_2$ (5)	**2a**	IPr·HCl (10)	NaO^{t}Bu (2.5)	120	trace	19	30/70
13	[Rh(cod)Cl]$_2$ (5)	**2a**	IMes·HCl (10)	NaO^{t}Bu (2.5)	120	7	70	9/91
**14**	**Rh(cod)(IMes)Cl (10)**	**2a**	-	**NaO^{t}Bu (2.5)**	**120**	**7**	**85[c]**	**8/92**
15	Rh(cod)(IMes)Cl (10)	**2a'**	-	NaO^{t}Bu (2.5)	120	6	67	8/92
16	Rh(cod)(IMes)Cl (10)	**2a''**	-	NaO^{t}Bu (2.5)	120	5	28	16/84
17	Rh(cod)(IMes)Cl (5)	**2a**	-	NaO^{t}Bu (2.5)	120	4	35	11/89

Unless otherwise specified, Reaction Conditions: **1a** (0.20 mmol), 5 mol% or 10 mol% catalyst, 10 mol% ligand, 2.5 equiv of base, 2.5 equiv of **2a**, **2a'** or **2a''**, 1.0 ml toluene, 120 ℃, 12 h. [a]Determined by ^{1}H-NMR analysis using dibromomethane as an internal standard. [b]Regioselectivity were determined by crude ^{1}H NMR associated with GC-MS. [c]Yield of isolated products.

Successful exapmples:

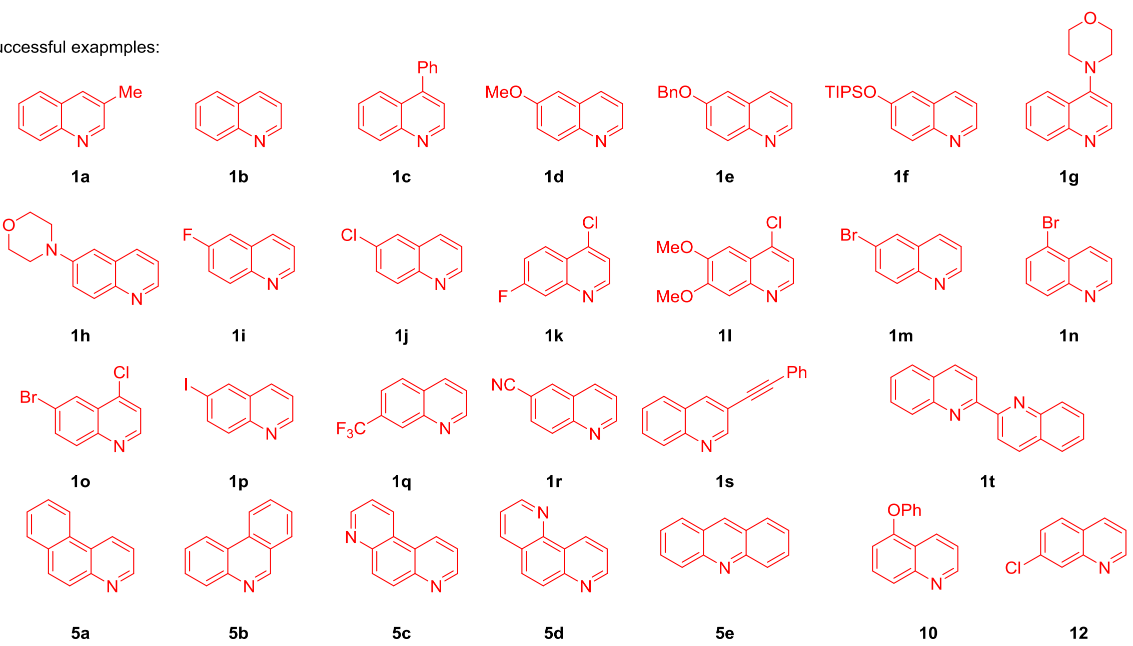

Failed exapmples:

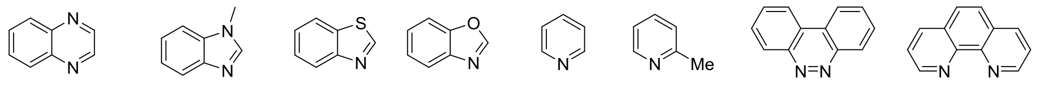

Supplementary Fig. 1. Substrate scope of aza-arenes

Successful exapmples:

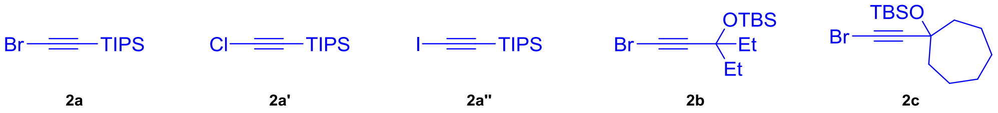

Failed exapmples:

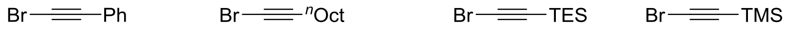

Supplementary Fig. 2. Substrate scope of alkynyl bromides

***General Procedure I*:**

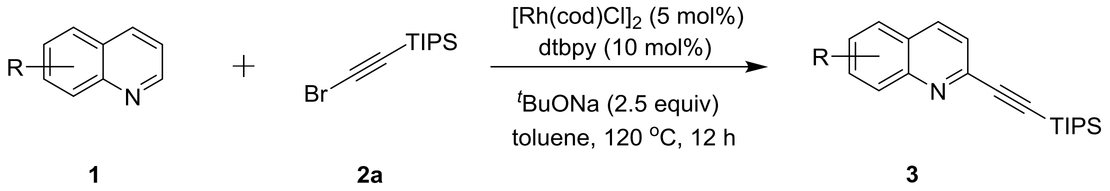

Under argon atmosphere, to a flame-dried 25 mL Schlenk tube was added $[Rh(cod)Cl]_2$ (4.9 mg, 0.01 mmol, 5 mol%), dtbpy (5.4 mg, 0.02 mmol, 10 mol%) and anhydrous toluene (1 ml). The resulting solution was stirred for 30 min at room temperature. ^{t}BuONa (48.1 mg, 0.5 mmol, 2.5 equiv) was added in the glove box. Then

1 (0.2 mmol) and **2a** (130 mg, 0.5 mmol, 2.5 equiv) were added and the tube was sealed. Then heated at 120 ℃ for 12 h. Afterwards, the mixture was cooled to room temperature. The mixture was diluted with DCM (~5 mL), silica gel was added and the solvent was evaporated under reduced pressure. The regioisomeric ratios (r.r.) were determined at this stage by GC-MS analysis. It was purified by silica gel chromatography to afford the desired product.

***General Procedure II*:**

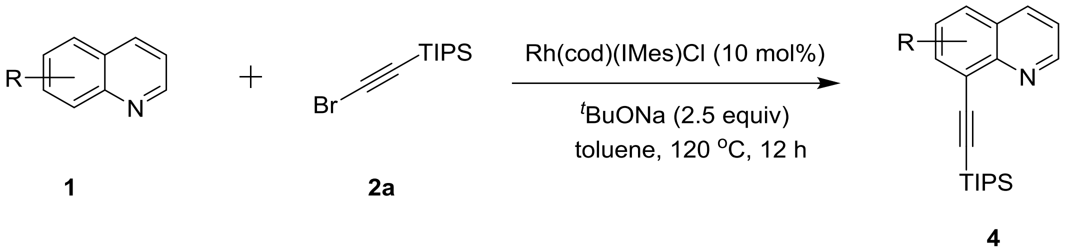

Under argon atmosphere, to a flame-dried 25 mL Schlenk tube was added Rh(cod)(IMes)Cl (11.0 mg, 0.02 mmol, 10 mol%) and ^{t}BuONa (48.1 mg, 0.5 mmol, 2.5 equiv) in the glove box. Then **1** (0.2 mmol), **2a** (130 mg, 0.5 mmol, 2.5 equiv) and anhydrous toluene (1 ml) were added and the tube was sealed. The resulting solution was stirred at 120 ℃ for 12 h. Afterwards, the mixture was cooled to room temperature. The mixture was diluted with DCM (~5 mL), silica gel was added and the solvent was evaporated under reduced pressure. The regioisomeric ratios (r.r.) were determined at this stage by GC-MS analysis. It was purified by silica gel chromatography to afford the desired product.

3-Methyl-2-((triisopropylsilyl)ethynyl)quinoline (3aa)

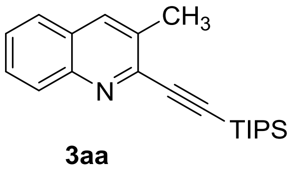

3aa

Following the ***general procedure I***, product **3aa** was obtained as a brown liquid (54.2 mg, 84% yield, 99/1 r.r.) after purification by flash column chromatography (PE/EA = 30:1). **^{1}H NMR (500 MHz, $CDCl_3$)** δ 8.07 (d, *J* = 8.5 Hz, 1H), 7.90 (s, 1H), 7.67 (d, *J* = 8.1 Hz, 1H), 7.62 (ddd, *J* = 8.5, 6.7, 1.5 Hz, 1H), 7.47 (td, *J* = 7.4, 6.7, 1.2 Hz, 1H), 2.60 (s, 3H), 1.22 – 1.16 (m, 21H). **^{13}C NMR (126 MHz, $CDCl_3$)** δ

146.6, 144.5, 135.0, 132.8, 129.0, 128.8, 127.5, 127.0, 126.6, 104.8, 96.2, 20.1, 18.7, 11.3. **ATR-FTIR (cm^{-1})**: 2943, 2865, 1264, 732, 702. **HRMS m/z (ESI)**: calcd for $C_{21}H_{30}NSi^+$ $(M + H)^+$ 324.2142, found 324.2140.

3-Methyl-8-((triisopropylsilyl)ethynyl)quinoline (4aa)

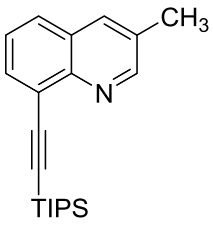

4aa

Following the ***general procedure II***, product **4aa** was obtained as a brown liquid (54.9 mg, 85% yield, 92/8 r.r.) after purification by flash column chromatography (PE/EA = 30:1). **^{1}H NMR (400 MHz, $CDCl_3$)** δ 8.84 (d, *J* = 2.3 Hz, 1H), 7.90 – 7.81 (m, 2H), 7.67 (dd, *J* = 8.2, 1.5 Hz, 1H), 7.42 (dd, *J* = 8.2, 7.2 Hz, 1H), 2.49 (s, 3H), 1.24 – 1.21 (m, 21H). **^{13}C NMR (101 MHz, $CDCl_3$)** δ 153.0, 146.8, 134.6, 133.5, 130.9, 128.0, 127.7, 125.8, 123.4, 104.9, 96.9, 28.2, 18.9, 11.5. **ATR-FTIR (cm^{-1})**: 2941, 2864, 2151, 1463, 881, 740. **HRMS m/z (ESI)**: calcd for $C_{21}H_{30}NSi^+$ $(M + H)^+$ 324.2142, found 324.2141.

2-((Triisopropylsilyl)ethynyl)quinoline (3ba)

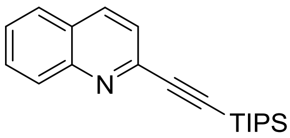

3ba

Following the ***general procedure I***, product **3ba** was obtained as a brown liquid (50.4 mg, 73% yield, 98/2 r.r.) after purification by flash column chromatography (PE/EA = 30:1). **^{1}H NMR (400 MHz, $CDCl_3$)** δ 8.15 – 8.04 (m, 2H), 7.77 (dd, *J* = 8.1, 1.4 Hz, 1H), 7.72 – 7.68 (m, 1H), 7.56 – 7.49 (m, 2H), 1.20 – 1.16 (m, 21H). **^{13}C NMR (101 MHz, $CDCl_3$)** δ 148.1, 143.5, 135.9, 129.9, 129.4, 127.4, 127.1, 127.0, 125.0, 106.6, 92.8, 18.7, 11.3. **ATR-FTIR (cm^{-1})**: 2942, 2865, 1594, 1462, 1220, 750. **HRMS m/z (ESI)**: calcd for $C_{20}H_{28}NSi^+$ $(M + H)^+$ 310.1986, found 310.1984.

8-((Triisopropylsilyl)ethynyl)quinoline (4ba)

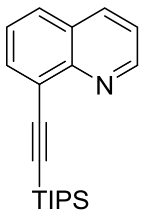

4ba

Following the ***general procedure II***, product **4ba** was obtained as a brown liquid (28.6 mg, 46% yield, 89/11 r.r.) after purification by flash column chromatography (PE/EA = 30:1). **^{1}H NMR (400 MHz, CDCl$_3$)** δ 8.99 (dd, *J* = 4.2, 1.8 Hz, 1H), 8.12 (dd, *J* = 8.3, 1.8 Hz, 1H), 7.95 (dd, *J* = 7.2, 1.4 Hz, 1H), 7.76 (dd, *J* = 8.2, 1.4 Hz, 1H), 7.48 – 7.45 (m, 1H), 7.40 (dd, *J* = 8.3, 4.2 Hz, 1H), 1.23 – 1.21 (m, 21H). **^{13}C NMR (101 MHz, CDCl$_3$)** δ 151.1, 148.6, 136.1, 134.6, 128.3, 128.2, 125.8, 123.8, 121.5, 104.7, 97.3, 18.8, 11.5. **ATR-FTIR (cm^{-1})**: 2942, 2864, 1463, 1264, 882, 732. **HRMS m/z (ESI)**: calcd for $C_{20}H_{28}NSi^{+}$ (M + H)$^{+}$ 310.1986, found 310.1986.

4-Phenyl-2-((triisopropylsilyl)ethynyl)quinoline (3ca)

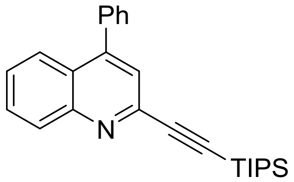

3ca

Following the ***general procedure I***, product **3ca** was obtained as a brown liquid (45.6 mg, 59% yield, >99/1 r.r.) after purification by flash column chromatography (PE/EA = 15:1). **^{1}H NMR (500 MHz, CDCl$_3$)** δ 8.19 (dd, *J* = 8.5, 1.2 Hz, 1H), 7.85 (dd, *J* = 8.5, 1.3 Hz, 1H), 7.73 – 7.70 (m, 1H), 7.61 – 7.45 (m, 7H), 1.20 – 1.16 (m, 21H). **^{13}C NMR (126 MHz, CDCl$_3$)** δ 148.6, 148.4, 143.1, 137.4, 129.8, 129.7, 129.4, 128.6, 128.5, 127.1, 125.9, 125.6, 125.0, 106.6, 92.7, 18.7, 11.3. **ATR-FTIR (cm^{-1})**: 2942, 2864, 1586, 1462, 1189, 693. **HRMS m/z (ESI)**: calcd for $C_{26}H_{32}NSi^{+}$ (M + H)$^{+}$ 386.2299, found 386.2301.

4-Phenyl-8-((triisopropylsilyl)ethynyl)quinolone (4ca)

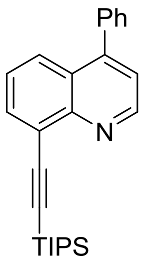

4ca

Following the ***general procedure II***, product **4ca** was obtained as a brown liquid (57.7 mg, 75% yield, 98/2 r.r) after purification by flash column chromatography (PE/EA = 30:1). **^{1}H NMR (500 MHz, CDCl$_3$)** δ 9.07 (d, *J* = 2.1 Hz, 1H), 8.27 (d, *J* = 2.1 Hz, 1H), 7.93 (dd, *J* = 7.2, 1.4 Hz, 1H), 7.74 (dd, *J* = 8.2, 1.4 Hz, 1H), 7.61 – 7.58 (m, 2H), 7.51 – 7.49 (m, 1H), 7.40 – 7.38 (m, 3H), 1.23 – 1.21 (m, 21H). **^{13}C NMR (126 MHz, CDCl$_3$)** δ 152.7, 147.1, 138.2, 134.8, 131.8, 128.8, 128.5, 128.0, 127.2, 126.6, 123.8, 122.6,

117.9, 104.2, 97.8, 92.8, 86.6, 18.8, 11.5. **ATR-FTIR (cm^{-1})**: 2941, 2864, 2156, 1464, 1264, 737. **HRMS m/z (ESI)**: calcd for $C_{26}H_{32}NSi^+$ $(M + H)^+$ 386.2299, found 386.2300.

6-Methoxy-2-((triisopropylsilyl)ethynyl)quinoline (3da)

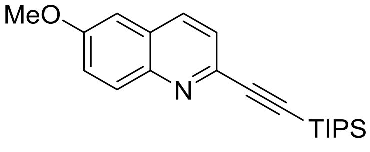

3da

Following the ***general procedure I***, product **3da** was obtained as a brown liquid (49.9 mg, 74% yield, 97/3 r.r.) after purification by flash column chromatography (PE/EA = 15:1). **^{1}H NMR (500 MHz, $CDCl_3$)** δ 7.98 (d, *J* = 9.2 Hz, 1H), 7.94 (d, *J* = 8.4 Hz, 1H), 7.48 (d, *J* = 8.4 Hz, 1H), 7.33 (dd, *J* = 9.2, 2.8 Hz, 1H), 6.99 (d, *J* = 2.8 Hz, 1H), 3.89 (s, 3H), 1.17 – 1.15 (m, 21H). **^{13}C NMR (126 MHz, $CDCl_3$)** δ 158.2, 144.1, 140.9, 134.5, 130.8, 128.3, 125.3, 122.7, 106.7, 104.9, 91.6, 55.5, 18.7, 11.3. **ATR-FTIR (cm^{-1})**: 2942, 2864, 1622, 1462, 1233, 833. **HRMS m/z (ESI)**: calcd for $C_{21}H_{30}NOSi^+$ $(M + H)^+$ 340.2091, found 340.2091.

6-Methoxy-8-((triisopropylsilyl)ethynyl)quinolone (4da)

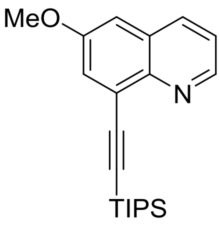

4da

Following the ***general procedure II***, product **4da** was obtained as a brown liquid (39.8 mg, 59% yield, 97/3 r.r.) after purification by flash column chromatography (PE/EA = 20:1). **^{1}H NMR (400 MHz, $CDCl_3$)** δ 8.83 (dd, *J* = 4.2, 1.7 Hz, 1H), 7.99 (dd, *J* = 8.3, 1.8 Hz, 1H), 7.59 (d, *J* = 2.8 Hz, 1H), 7.34 (dd, *J* = 8.3, 4.2 Hz, 1H), 7.03 (d, *J* = 2.9 Hz, 1H), 3.91 (s, 3H), 1.22 – 1.21 (m, 21H). **^{13}C NMR (101 MHz, $CDCl_3$)** δ 156.7, 148.6, 144.7, 134.8, 129.2, 126.6, 125.0, 121.7, 106.4, 104.2, 97.3, 55.6, 18.8, 11.5. **ATR-FTIR (cm^{-1})**: 2942, 2864, 2361, 1468, 1264, 733. **HRMS m/z (ESI)**: calcd for $C_{21}H_{30}NOSi^+$ $(M + H)^+$ 340.2091, found 340.2091.

6-(Benzyloxy)-2-((triisopropylsilyl)ethynyl)quinoline (3ea)

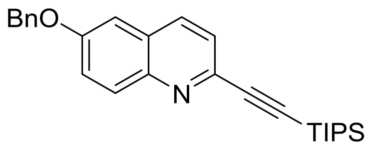

3ea

Following the ***general procedure I***, product **3ea** was obtained as a brown liquid (80.4 mg, 97% yield, >99/1 r.r.) after purification by flash column chromatography (PE/EA = 10:1). **^{1}H NMR (400 MHz, CDCl$_3$)** δ 8.04 (d, *J* = 9.2 Hz, 1H), 7.95 (d, *J* = 8.4 Hz, 1H), 7.54 – 7.34 (m, 7H), 7.10 (d, *J* = 2.7 Hz, 1H), 5.15 (s, 2H), 1.20 – 1.18 (m, 21H). **^{13}C NMR (101 MHz, CDCl$_3$)** δ 157.3, 144.2, 141.1, 136.3, 134.6, 131.0, 128.6, 128.23, 128.15, 127.5, 125.3, 122.96 , 106.7, 106.2, 91.8, 70.3, 18.7, 11.3. **ATR-FTIR (cm^{-1})**: 2942, 2864, 1619, 1264, 1228, 733. **HRMS m/z (ESI)**: calcd for $C_{27}H_{34}NOSi^+$ $(M + H)^+$ 416.2404, found 416.2404.

6-(Benzyloxy)-8-((triisopropylsilyl)ethynyl)quinolone (4ea)

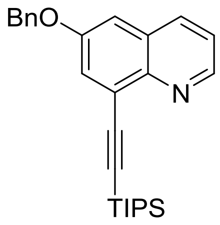

4ea

Following the ***general procedure II***, product **4ea** was obtained as a brown liquid (57.2 mg, 69% yield, >99/1 r.r.) after purification by flash column chromatography (PE/EA = 10:1). **^{1}H NMR (500 MHz, CDCl$_3$)** δ 8.85 (dd, *J* = 4.2, 1.7 Hz, 1H), 7.98 (dd, *J* = 8.3, 1.8 Hz, 1H), 7.71 (d, *J* = 2.8 Hz, 1H), 7.54 – 7.46 (m, 2H), 7.45 – 7.40 (m, 2H), 7.39 – 7.32 (m, 2H), 7.12 (d, *J* = 2.8 Hz, 1H), 5.16 (s, 2H), 1.25 – 1.22 (m, 21H). **^{13}C NMR (126 MHz, CDCl$_3$)** δ 155.8, 148.7, 144.7, 136.3, 134.9, 129.1, 128.6, 128.2, 127.5, 127.0, 125.1, 121.7, 107.6, 104.2, 97.4, 70.3, 18.8, 11.5. **ATR-FTIR (cm^{-1})**: 2940, 2863, 2153, 1463, 1414, 1096, 771. **HRMS m/z (ESI)**: calcd for $C_{27}H_{34}NOSi^+$ $(M + H)^+$ 416.2404, found 416.2405.

2-((Triisopropylsilyl)ethynyl)-6-((triisopropylsilyl)oxy)quinoline (3fa)

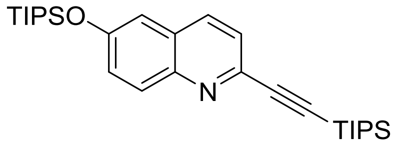

3fa

Following the ***general procedure I***, product **3fa** was obtained as a brown liquid (75.9 mg, 79% yield, 99/1 r.r.) after purification by flash column chromatography (PE/EA = 30:1). **^{1}H NMR (500 MHz, CDCl$_3$)** δ 7.99 (d, *J* = 9.1 Hz, 1H), 7.92 (d, *J* = 8.5 Hz, 1H), 7.47 (d, *J* = 8.4 Hz, 1H), 7.33 (dd, *J* = 9.2, 2.7 Hz, 1H), 7.12 (d, *J* = 2.7 Hz, 1H), 1.18 – 1.15 (m, 21H), 1.12 –

1.11 (m, 21H). **^{13}C NMR (126 MHz, CDCl$_3$)** δ 154.7, 144.1, 141.2, 134.4, 130.8, 128.3, 125.9, 125.1, 113.7, 106.8, 91.7, 18.7, 17.9, 12.6, 11.3. **ATR-FTIR (cm^{-1})**: 2944, 2866, 1618, 1484, 1241, 882. **HRMS m/z (ESI)**: calcd for $C_{29}H_{48}NOSi_2^+$ $(M + H)^+$ 482.3269, found 482.3268.

8-((Triisopropylsilyl)ethynyl)-6-((triisopropylsilyl)oxy)quinolone (4fa)

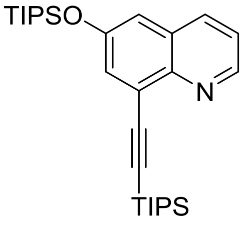

4fa

Following the ***general procedure II***, product **4fa** was obtained as a brown liquid (87.1 mg, 90% yield, 99/1 r.r.) after purification by flash column chromatography (PE/EA = 20:1). **^{1}H NMR (500 MHz, CDCl$_3$)** δ 8.83 (dd, *J* = 4.2, 1.8 Hz, 1H), 7.95 (dd, *J* = 8.3, 1.7 Hz, 1H), 7.56 (d, *J* = 2.7 Hz, 1H), 7.32 (dd, *J* = 8.3, 4.2 Hz, 1H), 7.13 (d, *J* = 2.8 Hz, 1H), 1.24 – 1.20 (m, 21H), 1.15 – 1.13 (m, 21H). **^{13}C NMR (126 MHz, CDCl$_3$)** δ 153.3, 148.8, 144.7, 134.7, 129.9, 129.3, 124.9, 121.5, 114.8, 104.4, 97.3, 18.8, 17.9, 12.7, 11.5. **ATR-FTIR (cm^{-1})**: 2943, 2865, 1591, 1460, 1263, 735. **HRMS m/z (ESI)**: calcd for $C_{29}H_{48}NOSi_2^+$ $(M + H)^+$ 482.3269, found 482.3267.

4-(2-((Triisopropylsilyl)ethynyl)quinolin-4-yl)morpholine (3ga)

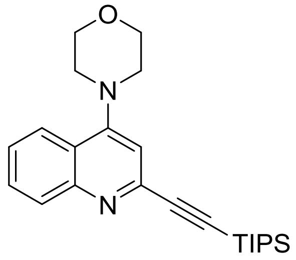

3ga

Following the ***general procedure I***, product **3ga** was obtained as a brown liquid (65.2 mg, 83% yield, >99/1 r.r.) after purification by flash column chromatography (PE/EA = 10:1). **^{1}H NMR (500 MHz, CDCl$_3$)** δ 8.05 (dd, *J* = 8.5, 1.2 Hz, 1H), 7.93 (dd, *J* = 8.4, 1.4 Hz, 1H), 7.64 – 7.60 (m, 1H), 7.46 – 7.43 (m, 1H), 6.96 (s, 1H), 3.97 – 3.95 (m, 4H), 3.22 – 3.20 (m, 4H), 1.18 – 1.15 (m, 21H). **^{13}C NMR (126 MHz, CDCl$_3$)** δ 156.4, 149.4, 143.8, 130.1, 129.4, 125.9, 123.1, 122.5, 112.4, 106.8, 91.7, 66.8, 52.4, 18.6, 11.2. **ATR-FTIR (cm^{-1})**: 2942, 2863, 1578, 1413, 1118, 766. **HRMS m/z (ESI)**: calcd for $C_{24}H_{35}N_2OSi^+$ $(M + H)^+$ 395.2513, found 395.2514.

4-(8-((Triisopropylsilyl)ethynyl)quinolin-4-yl)morpholine (4ga)

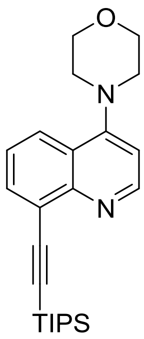

4ga

Following the ***general procedure II***, product **4ga** was obtained as a brown liquid (70.2 mg, 89% yield, >99/1 r.r.) after purification by flash column chromatography (PE/EA = 5:1). **^{1}H NMR (500 MHz, CDCl$_3$)** δ 8.81 (d, *J* = 4.9 Hz, 1H), 7.97 (dd, *J* = 8.5, 1.5 Hz, 1H), 7.89 (dd, *J* = 7.2, 1.5 Hz, 1H), 7.41 – 7.38 (m, 1H), 6.85 (d, *J* = 4.9 Hz, 1H), 3.97 – 3.95 (m, 4H), 3.17 – 3.15 (m, 4H), 1.22 – 1.19 m, 21H). **^{13}C NMR (126 MHz, CDCl$_3$)** δ 156.7, 151.4, 149.9, 134.4, 124.6, 124.2, 123.9, 123.4, 109.1, 105.1, 97.0, 66.8, 52.6, 18.8, 11.5. **ATR-FTIR (cm^{-1})**: 2942, 2862, 1581, 1504, 1118, 961, 661. **HRMS m/z (ESI)**: calcd for $C_{24}H_{35}N_2OSi^+$ $(M + H)^+$ 395.2513, found 395.2513.

4-(2-((Triisopropylsilyl)ethynyl)quinolin-6-yl)morpholine (3ha)

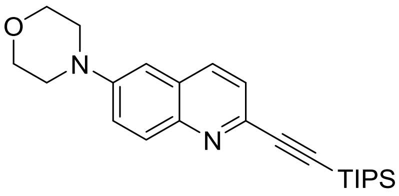

3ha

Following the ***general procedure I***, product **3ha** was obtained as a brown liquid (57.5 mg, 76% yield, >99/1 r.r.) after purification by flash column chromatography (PE/EA = 10:1). **^{1}H NMR (500 MHz, CDCl$_3$)** δ 7.96 (d, *J* = 9.3 Hz, 1H), 7.88 (d, *J* = 8.5 Hz, 1H), 7.45 – 7.41 (m, 2H), 6.92 (d, *J* = 2.7 Hz, 1H), 3.88 – 3.86 (m, 4H), 3.27 – 3.25 (m, 4H), 1.17 – 1.13 (m, 21H). **^{13}C NMR (126 MHz, CDCl$_3$)** δ 149.4, 143.6, 140.5, 134.3, 130.2, 128.3, 125.4, 122.0, 108.2, 106.8, 91.5, 66.7, 48.9, 18.6, 11.2. **ATR-FTIR (cm^{-1})**: 2941, 2862, 1606, 1462, 1122, 987, 675. **HRMS m/z (ESI)**: calcd for $C_{24}H_{35}N_2OSi^+$ $(M + H)^+$ 395.2513, found 395.2513.

4-(8-((Triisopropylsilyl)ethynyl)quinolin-6-yl)morpholine (4ha)

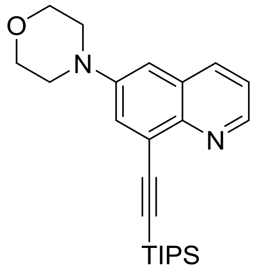

4ha

Following the ***general procedure II***, product **4ha** was obtained as a brown liquid (56.8 mg, 72% yield, 99/1 r.r.) after purification by flash column chromatography (PE/EA = 5:1). **^{1}H NMR (500 MHz, CDCl$_3$)** δ 8.80 – 8.78 (m, 1H), 7.94 (dd, *J* = 8.2, 1.7 Hz, 1H), 7.68 (d, *J* = 2.8 Hz, 1H), 7.30 (dd, *J* = 8.2, 4.1 Hz, 1H), 6.98 (d, *J* = 2.8 Hz, 1H), 3.91 – 3.89 (m, 4H), 3.27 – 3.25 (m, 4H), 1.23 – 1.19 (m, 21H). **^{13}C NMR (126 MHz, CDCl$_3$)** δ 148.43, 148.40, 144.3, 134.7, 129.2, 126.6, 124.3, 121.7, 110.1, 104.6, 96.7, 66.8, 49.3, 18.8, 11.5. **ATR-FTIR (cm^{-1})**: 2942, 2863, 1617, 1264, 1228, 1124, 733. **HRMS m/z (ESI)**: calcd for $C_{24}H_{35}N_2OSi^+$ (M + H)$^+$ 395.2513, found 395.2513.

6-Fluoro-2-((triisopropylsilyl)ethynyl)quinoline (3ia)

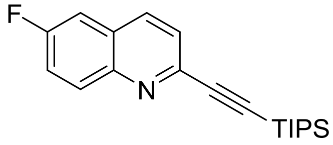

3ia

Following the ***general procedure I***, product **3ia** was obtained as a brown liquid (54.0 mg, 83% yield, 97/3 r.r.) after purification by flash column chromatography (PE/EA = 30:1). **^{1}H NMR (400 MHz, CDCl$_3$)** δ 8.09 (dd, *J* = 9.3, 5.3 Hz, 1H), 8.02 (d, *J* = 8.5 Hz, 1H), 7.54 – 7.52 (m, 1H), 7.49 – 7.43 (m, 1H), 7.37 (dd, *J* = 8.7, 2.8 Hz, 1H), 1.18 – 1.16 (m, 21H). **^{13}C NMR (101 MHz, CDCl$_3$)** δ 160.7 (d, *J* = 249.8 Hz), 145.1, 142.9 (d, *J* = 3.1 Hz), 135.2 (d, *J* = 5.6 Hz), 131.9 (d, *J* = 9.2 Hz), 127.8 (d, *J* = 10.1 Hz), 125.7, 120.2 (d, *J* = 25.9 Hz), 110.5 (d, *J* = 21.9 Hz), 106.2, 93.0, 18.6, 11.3. **^{19}F NMR (376 MHz, CDCl$_3$)** δ -112.11. **ATR-FTIR (cm^{-1})**: 2943, 2865, 1555, 1227, 882, 708. **HRMS m/z (ESI)**: calcd for $C_{20}H_{27}FNSi^+$ (M + H)$^+$ 328.1891, found 328.1891.

6-Fluoro-8-((triisopropylsilyl)ethynyl)quinolone (4ia)

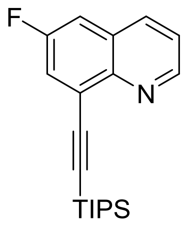

4ia

Following the ***general procedure II***, product **4ia** was obtained as a brown liquid (62.8 mg, 96% yield, 98/2 r.r.) after purification by flash column chromatography (PE/EA = 30:1). **^{1}H NMR (500 MHz, CDCl$_3$)** δ 8.94 (dd, *J* = 4.2, 1.8 Hz, 1H), 8.05 (dd, *J* = 8.4, 1.8 Hz, 1H), 7.69 (dd, *J* = 8.9, 2.8 Hz, 1H), 7.41 – 7.37 (m 2H), 1.24 – 1.20 (m, 21H). **^{13}C NMR (126 MHz, CDCl$_3$)** δ 159.3 (d, *J* = 247.5 Hz), 150.2 (d, *J* = 2.8 Hz), 145.8, 135.5 (d, *J* = 5.6 Hz), 128.8 (d, *J* = 10.4 Hz), 126.1 (d, *J* = 10.4 Hz), 123.9 (d, *J* = 26.6 Hz), 122.1, 111.5 (d, *J* = 21.4 Hz), 103.3 (d, *J* = 3.0 Hz), 99.1, 18.7, 11.4. **^{19}F NMR (471 MHz, CDCl$_3$)** δ -114.00.**ATR-FTIR (cm^{-1})**: 2942, 2864, 1613, 1460, 1068, 881, 697. **HRMS m/z (ESI)**: calcd for $C_{20}H_{27}FNSi^+$ $(M + H)^+$ 328.1891, found 328.1889.

6-Chloro-2-((triisopropylsilyl)ethynyl)quinoline (3ja)

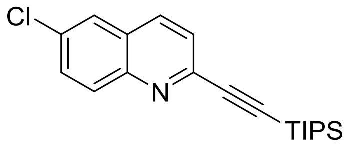

3ja

Following the ***general procedure I***, product **3ja** was obtained as a brown liquid (66.1 mg, 96% yield, 96/4 r.r.) after purification by flash column chromatography (PE/EA = 30:1). **^{1}H NMR (400 MHz, CDCl$_3$)** δ 8.02 (d, *J* = 9.1 Hz, 1H), 7.98 (d, *J* = 8.5 Hz, 1H), 7.72 (d, *J* = 2.3 Hz, 1H), 7.61 (dd, *J* = 9.0, 2.4 Hz, 1H), 7.53 (d, *J* = 8.4 Hz, 1H), 1.18 – 1.15 (m, 21H). **^{13}C NMR (101 MHz, CDCl$_3$)** δ 146.4, 143.7, 134.9, 132.8, 130.9, 130.8, 127.6, 126.1, 125.8, 106.2, 93.6, 18.6, 11.2. **ATR-FTIR (cm^{-1})**: 2943, 2865, 1485, 1264, 918, 735. **HRMS m/z (ESI)**: calcd for $C_{20}H_{27}ClNSi^+$ $(M + H)^+$ 344.1596, found 344.1597.

6-Chloro-8-((triisopropylsilyl)ethynyl)quinolone (4ja)

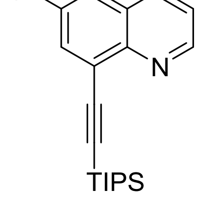

4ja

Following the ***general procedure II***, product **4ja** was obtained as a brown liquid (57.4 mg, 84% yield, 97/3 r.r.) after purification by flash column chromatography (PE/EA = 30:1). **^{1}H NMR (400 MHz, CDCl$_3$)** δ 8.97 (dd, *J* = 4.2, 1.8 Hz, 1H), 8.03 (dd, *J* = 8.3, 1.7 Hz, 1H), 7.86 (d, *J* = 2.3 Hz, 1H), 7.74 (d, *J* = 2.4 Hz, 1H), 7.42 (dd, *J* = 8.3, 4.2 Hz, 1H), 1.22 –1.20 (m, 21H). **^{13}C NMR (101 MHz, CDCl$_3$)** δ 151.1, 147.1, 135.2,

134.6, 131.4, 128.7, 126.8, 125.6, 122.3, 103.1, 99.2, 18.8, 11.4. **ATR-FTIR (cm^{-1})**: 2942, 2864, 1484, 1065, 867, 678. **HRMS m/z (ESI)**: calcd for $C_{20}H_{27}ClNSi^+$ (M + H)$^+$ 344.1596, found 344.1598.

4-Chloro-7-fluoro-2-((triisopropylsilyl)ethynyl)quinoline (3ka)

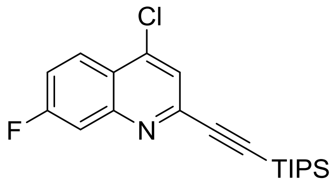

3ka

Following the ***general procedure I***, product **3ka** was obtained as a withe solid (32.6 mg, 45% yield, 82/18 r.r.) after purification by flash column chromatography (PE/EA = 30:1), m.p. = 71.3-72.5 °C. **^{1}H NMR (500 MHz, CDCl$_3$)** δ 8.21 – 8.18 (m, 1H), 7.76 (dd, *J* = 9.9, 2.6 Hz, 1H), 7.58 (s, 1H), 7.43 – 7.39 (m, 1H), 1.20 – 1.15 (m, 21H). **^{13}C NMR (126 MHz, CDCl$_3$)** δ 163.8 (d, *J* = 252.6 Hz), 149.9 (d, *J* = 13.0 Hz), 144.4, 142.4, 126.3 (d, *J* = 10.0 Hz), 124.2 (d, *J* = 2.4 Hz), 122.7, 118.4 (d, *J* = 25.7 Hz), 113.5 (d, *J* = 20.9 Hz), 105.1, 95.1, 18.7, 11.2. **^{19}F NMR (471 MHz, CDCl$_3$)** δ -107.62. **ATR-FTIR (cm^{-1})**: 2943, 2865, 1576, 1501, 1214, 885. **HRMS m/z (ESI)**: calcd for $C_{20}H_{26}ClFNSi^+$ (M + H)$^+$ 362.1502, found 362.1500.

4-Chloro-7-fluoro-8-((triisopropylsilyl)ethynyl)quinolone (4ka)

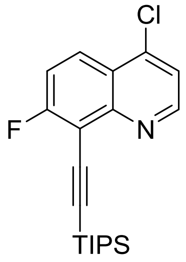

4ka

Following the ***general procedure II***, product **4ka** was obtained as a brown liquid (33.3 mg, 46% yield, 99/1 r.r.) after purification by flash column chromatography (PE/EA = 30:1). **^{1}H NMR (400 MHz, CDCl$_3$)** δ 8.86 (d, *J* = 4.7 Hz, 1H), 8.19 – 8.16 (m, 1H), 7.47 (d, *J* = 4.7 Hz, 1H), 7.45 – 7.40 (m, 1H), 1.22 – 1.19 (m, 21H). **^{13}C NMR (101 MHz, CDCl$_3$)** δ 165.1 (d, *J* = 257.2 Hz), 151.4, 150.4 (d, *J* = 6.3 Hz), 142.7 (d, *J* = 2.3 Hz), 125.5 (d, *J* = 10.5 Hz), 123.4, 121.0 (d, *J* = 2.8 Hz), 117.4 (d, *J* = 25.5 Hz), 110.3 (d, *J* = 15.2 Hz), 104.7 (d, *J* = 5.8 Hz), 96.5, 18.7, 11.4. **^{19}F NMR (376 MHz, CDCl$_3$)** δ-101.69. **ATR-FTIR (cm^{-1})**: 2942, 2964, 1494, 1292, 881, 674. **HRMS m/z (ESI)**: calcd for $C_{20}H_{26}ClFNSi^+$ (M + H)$^+$ 362.1502, found 362.1499.

4-Chloro-6,7-dimethoxy-2-((triisopropylsilyl)ethynyl)quinoline (3la)

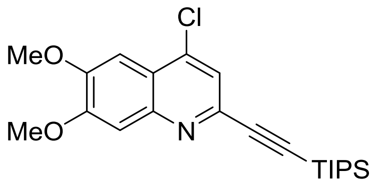

3la

Following the ***general procedure I***, product **3la** was obtained as a withe solid (37.7 mg, 46% yield, 99/1 r.r.) after purification by flash column chromatography (PE/EA = 10:1), m.p. = 109.7-111.3 °C. **^{1}H NMR (500 MHz, $CDCl_3$)** δ 7.49 (s, 1H), 7.41 (s, 1H), 7.32 (s, 1H), 4.03 (s, 3H), 4.00 (s, 3H), 1.16 – 1.13 (m, 21H). **^{13}C NMR (126 MHz, $CDCl_3$)** δ 153.3, 151.0, 145.8, 140.7, 139.9, 123.2, 121.3, 108.3, 105.6, 101.4, 92.5, 56.3, 56.2, 18.6, 11.2. **ATR-FTIR (cm^{-1})**: 2942, 2864, 1462, 1247, 1136, 887, 677. **HRMS m/z (ESI)**: calcd for $C_{22}H_{31}ClNO_2Si^+$ $(M + H)^+$ 404.1807, found 404.1806.

4-Chloro-6,7-dimethoxy-8-((triisopropylsilyl)ethynyl)quinolone (4la)

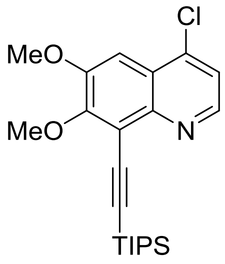

4la

Following the ***general procedure II***, product **4la** was obtained as a brown liquid (40.6 mg, 50% yield, 99/1 r.r.) after purification by flash column chromatography (PE/EA = 10:1). **^{1}H NMR (400 MHz, $CDCl_3$)** δ 8.67 (d, *J* = 4.8 Hz, 1H), 7.41 – 7.38 (m, 2H), 4.13 (s, 3H), 4.02 (s, 3H), 1.23 – 1.19 (m, 21H). **^{13}C NMR (101 MHz, $CDCl_3$)** δ 155.9, 153.0, 148.1, 146.3, 140.4, 123.7, 120.8, 116.1, 103.3, 102.6, 99.5, 61.2, 56.0, 18.7, 11.5. **ATR-FTIR (cm^{-1})**: 2940, 2863, 1463, 1293, 1096, 770, 669. **HRMS m/z (ESI)**: calcd for $C_{22}H_{31}ClNO_2Si^+$ $(M + H)^+$ 404.1807, found 404.1805.

6-Bromo-2-((triisopropylsilyl)ethynyl)quinoline (3ma)

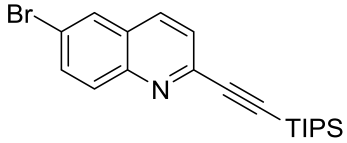

3ma

Following the ***general procedure I***, product **3ma** was obtained as a brown liquid (68.9 mg, 89% yield, 98/2 r.r.) after purification by flash column chromatography (PE/EA = 30:1). **^{1}H NMR (400 MHz, $CDCl_3$)** δ 7.96 (t, *J* = 8.6 Hz, 2H), 7.91 (d, *J* = 2.2 Hz, 1H), 7.75 (dd, *J* = 9.0, 2.2 Hz, 1H), 7.53 (d, *J* = 8.4 Hz, 1H), 1.18 – 1.15 (m, 21H). **^{13}C NMR (101 MHz, $CDCl_3$)** δ 146.6, 143.9, 134.8, 133.4, 131.1, 129.4, 128.1, 125.8, 121.0, 106.2, 93.7, 18.6, 11.2. **ATR-FTIR (cm^{-1})**:

2942, 2864, 1589, 1217, 910, 660. **HRMS m/z (ESI)**: calcd for $C_{20}H_{27}BrNSi^+$ (M + H)$^+$ 388.1091, found 388.1088.

6-Bromo-8-((triisopropylsilyl)ethynyl)quinolone (4ma)

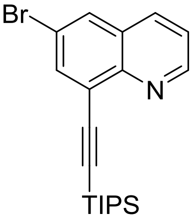

4ma

Following the ***general procedure II***, product **4ma** was obtained as a brown liquid (61.7 mg, 80% yield, 99/1 r.r.) after purification by flash column chromatography (PE/EA = 30:1). **^{1}H NMR (400 MHz, CDCl$_3$)** δ 8.98 (dd, *J* = 4.2, 1.8 Hz, 1H), 8.03 (dd, *J* = 8.3, 1.8 Hz, 1H), 7.99 (d, *J* = 2.3 Hz, 1H), 7.92 (d, *J* = 2.2 Hz, 1H), 7.42 (dd, *J* = 8.3, 4.2 Hz, 1H), 1.23 – 1.19 (m, 21H). **^{13}C NMR (101 MHz, CDCl$_3$)** δ 151.3, 147.3, 137.1, 135.1, 130.2, 129.2, 125.8, 122.3, 119.3, 103.0, 99.3, 18.8, 11.5. **ATR-FTIR (cm^{-1})**: 2942, 2864, 1485, 1264, 895, 734. **HRMS m/z (ESI)**: calcd for $C_{20}H_{27}BrNSi^+$ (M + H)$^+$ 388.1091, found 388.1090.

5-Bromo-2-((triisopropylsilyl)ethynyl)quinoline (3na)

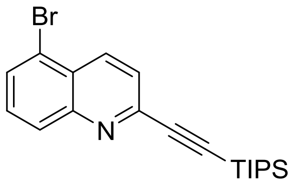

3na

Following the ***general procedure I***, product **3na** was obtained as a brown liquid (33.6 mg, 43% yield, 99/1 r.r.) after purification by flash column chromatography (PE/EA = 40:1). **^{1}H NMR (500 MHz, CDCl$_3$)** δ 8.46 (dd, *J* = 8.7, 0.8 Hz, 1H), 8.08 (d, *J* = 8.5 Hz, 1H), 7.80 (dd, *J* = 7.5, 1.0 Hz, 1H), 7.61 (d, *J* = 8.7 Hz, 1H), 7.57 – 7.54 (m, 1H), 1.20 – 1.16 (m, 21H). **^{13}C NMR (126 MHz, CDCl$_3$)** δ 148.8, 144.3, 135.4, 130.8, 130.1, 129.4, 126.6, 126.1, 121.6, 105.9, 94.3, 18.7, 11.3. **ATR-FTIR (cm^{-1})**: 2942, 2864, 1582, 1234, 1121, 810. **HRMS m/z (ESI)**: calcd for $C_{20}H_{27}BrNSi^+$ (M + H)$^+$ 388.1091, found 388.1091.

5-Bromo-8-((triisopropylsilyl)ethynyl)quinolone (4na)

Following the ***general procedure II***, product **4na** was obtained as a brown liquid (51.7 mg, 67% yield, 99/1 r.r.) after purification by flash column chromatography (PE/EA =

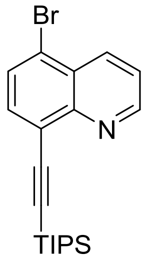

4na

30:1). **^{1}H NMR (400 MHz, CDCl$_3$)** δ 9.00 (dd, *J* = 4.2, 1.7 Hz, 1H), 8.51 (dd, *J* = 8.5, 1.7 Hz, 1H), 7.79 – 7.74 (m, 2H), 7.51 (dd, *J* = 8.5, 4.2 Hz, 1H), 1.23 – 1.19 (m, 21H). **^{13}C NMR (101 MHz, CDCl$_3$)** δ 151.6, 149.1, 135.5, 134.2, 129.8, 127.6, 123.9, 122.6, 122.2, 103.9, 98.7, 18.8, 11.5. **ATR-FTIR (cm^{-1})**: 2941, 2964, 1460, 1382, 1055, 743. **HRMS m/z (ESI)**: calcd for $C_{20}H_{27}BrNSi^{+}$ (M + H)$^{+}$ 388.1091, found 388.1090.

6-Bromo-4-chloro-2-((triisopropylsilyl)ethynyl)quinoline (3oa)

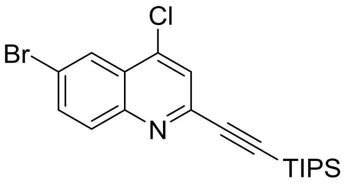

3oa

Following the ***general procedure I***, product **3oa** was obtained as a white solid (61.9 mg, 77% yield, 97/3 r.r.) after purification by flash column chromatography (PE/EA = 30:1), m.p. = 76.5-78.1 ^{o}C. **^{1}H NMR (500 MHz, CDCl$_3$)** δ 8.33 (d, *J* = 2.2 Hz, 1H), 7.97 (d, *J* = 8.9 Hz, 1H), 7.82 (dd, *J* = 9.0, 2.2 Hz, 1H), 7.62 (s, 1H), 1.19 – 1.15 (m, 21H). **^{13}C NMR (126 MHz, CDCl$_3$)**) δ 147.3, 143.4, 141.1, 134.4, 131.4, 126.6, 126.2, 125.5, 122.5, 105.1, 95.1, 18.7, 11.2. **ATR-FTIR (cm^{-1})**: 2942, 2864, 1577, 1471, 1288, 937, 682. **HRMS m/z (ESI)**: calcd for $C_{20}H_{26}BrClNSi^{+}$ (M + H)$^{+}$ 422.0701, found 422.0700.

6-Bromo-4-chloro-8-((triisopropylsilyl)ethynyl)quinolone (4oa)

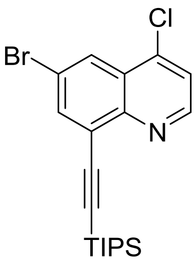

4oa

Following the ***general procedure II***, product **4oa** was obtained as a brown liquid (60.6 mg, 72% yield, 99/1 r.r.) after purification by flash column chromatography (PE/EA = 30:1). **^{1}H NMR (500 MHz, CDCl$_3$)** δ 8.83 (d, *J* = 4.6 Hz, 1H), 8.33 (d, *J* = 2.2 Hz, 1H), 8.04 (d, *J* = 2.2 Hz, 1H), 7.50 (d, *J* = 4.6 Hz, 1H), 1.21 – 1.19 (m, 21H). **^{13}C NMR (126 MHz, CDCl$_3$)** δ 150.5, 148.1, 141.5, 137.9, 127.5, 126.6, 126.2, 122.3, 120.8, 102.5, 100.1, 18.7, 11.4. **ATR-FTIR (cm^{-1})**: 2942, 2864, 1573, 1264, 882, 734. **HRMS m/z (ESI)**: calcd for $C_{20}H_{26}BrClNSi^{+}$ (M + H)$^{+}$ 422.0701, found 422.0697.

6-Iodo-2-((triisopropylsilyl)ethynyl)quinoline (3pa)

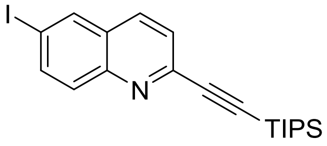

3pa

Following the ***general procedure I***, product **3pa** was obtained as a white solid (66.8 mg, 77% yield, 98/2 r.r.) after purification by flash column chromatography (PE/EA = 40:1). **^{1}H NMR (500 MHz, CDCl$_3$)** δ 8.16 (d, *J* = 2.0 Hz, 1H), 7.96 (d, *J* = 8.5 Hz, 1H), 7.92 (dd, *J* = 8.9, 2.0 Hz, 1H), 7.81 (d, *J* = 8.9 Hz, 1H), 7.52 (d, *J* = 8.5 Hz, 1H), 1.19 – 1.15 (m, 21H). **^{13}C NMR (126 MHz, CDCl$_3$)**) δ 147.0, 144.0, 138.6, 136.2, 134.6, 131.0, 128.6, 125.7, 106.2, 93.9, 92.8, 18.7, 11.2. **ATR-FTIR (cm^{-1})**: 2941, 2964, 1584, 1477, 1218, 905, 829. **HRMS m/z (ESI)**: calcd for $C_{20}H_{27}INSi^+$ (M + H)$^+$ 436.0952, found 436.0953.

6-Iodo-8-((triisopropylsilyl)ethynyl)quinolone (4pa)

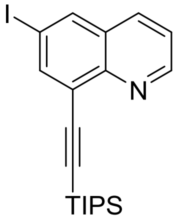

4pa

Following the ***general procedure II***, product **4pa** was obtained as a brown liquid (27.7 mg, 32% yield, >99/1 r.r.) after purification by flash column chromatography (PE/EA = 30:1). **^{1}H NMR (500 MHz, CDCl$_3$)** δ 8.98 (dd, *J* = 4.2, 1.8 Hz, 1H), 8.15 – 8.14 (m, 2H), 7.99 (dd, *J* = 8.3, 1.8 Hz, 1H), 7.40 (dd, *J* = 8.3, 4.2 Hz, 1H), 1.22 – 1.19 (m, 21H). **^{13}C NMR (126 MHz, CDCl$_3$)** δ 151.4, 147.6, 142.3, 136.9, 134.8, 129.6, 125.6, 122.1, 102.8, 99.2, 90.6, 18.8, 11.4. **ATR-FTIR (cm^{-1})**: 2941, 2863, 1463, 1264, 882, 742. **HRMS m/z (ESI)**: calcd for $C_{20}H_{27}INSi^+$ (M + H)$^+$ 436.0952, found 436.0951.

7-(Trifluoromethyl)-2-((triisopropylsilyl)ethynyl)quinoline (3qa)

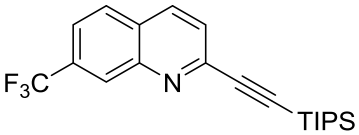

3qa

Following the ***general procedure I***, product **3qa** was obtained as a brown liquid (65.4 mg, 87% yield, >99/1 r.r.) after purification by flash column chromatography (PE/EA = 30:1). **^{1}H NMR (500 MHz, CDCl$_3$)** δ 8.42 (s, 1H), 8.13 (d, *J* = 8.5 Hz, 1H), 7.88 (d, *J* = 8.5 Hz, 1H), 7.68 (dd, *J* = 8.5, 1.8 Hz, 1H), 7.62 (d, *J* = 8.5 Hz, 1H), 1.20 – 1.16 (m, 21H). **^{13}C NMR (126 MHz, CDCl$_3$)** δ 147.1, 145.0, 135.7, 131.7 (q, *J* = 32.7 Hz), 128.6, 128.4, 127.2 (q, *J* = 4.4 Hz), 126.7, 124.9 (q, *J* = 272.7 Hz), 122.6 (q, *J* = 3.1 Hz), 105.9 , 94.5 , 18.6, 11.2. **^{19}F NMR (471 MHz,**

CDCl3) δ -62.84. **ATR-FTIR (cm^{-1})**: 2945, 2866, 1599, 1314, 1217, 1127, 734. **HRMS m/z (ESI)**: calcd for $C_{21}H_{27}F_3NSi^+$ $(M + H)^+$ 378.1859, found 378.1857.

7-(Trifluoromethyl)-8-((triisopropylsilyl)ethynyl)quinolone (4qa)

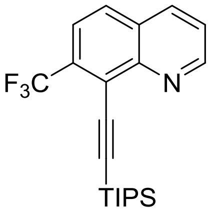

4qa

Following the ***general procedure II***, product **4qa** was obtained as a brown liquid (48.5 mg, 64% yield, 95/5 r.r.) after purification by flash column chromatography (PE/EA = 20:1). **^{1}H NMR (500 MHz, CDCl3)** δ 9.05 (dd, *J* = 4.1, 1.8 Hz, 1H), 8.15 (dd, *J* = 8.3, 1.8 Hz, 1H), 7.81 (d, *J* = 8.6 Hz, 1H), 7.75 (d, *J* = 8.6 Hz, 1H), 7.49 (dd, *J* = 8.3, 4.1 Hz, 1H), 1.26 – 1.21 (m, 21H). **^{13}C NMR (126 MHz, CDCl3)** δ 152.1, 148.9, 135.9, 133.3 (q, *J* = 30.2 Hz), 129.3, 127.8, 123.6 (q, *J* = 274.1 Hz), 123.1 (q, *J* = 2.5 Hz), 123.0, 122.3 (q, *J* = 5.0 Hz), 107.1 (d, *J* = 1.3 Hz), 99.2, 18.6, 11.5. **^{19}F NMR (471 MHz, CDCl3)** δ -61.18. **ATR-FTIR (cm^{-1})**: 2943, 2856, 1453, 1322, 1136, 882, 734. **HRMS m/z (ESI)**: calcd for $C_{21}H_{27}F_3NSi^+$ $(M + H)^+$ 378.1859, found 378.1858.

2-((Triisopropylsilyl)ethynyl)quinoline-6-carbonitrile (3ra)

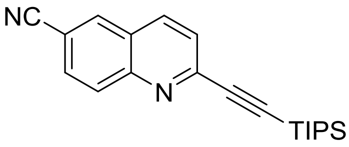

3ra

Following the ***general procedure I***, product **3ra** was obtained as a brown liquid (8.8 mg, 13% yield, 96/4 r.r.) after purification by flash column chromatography (PE/EA = 30:1). **^{1}H NMR (500 MHz, CDCl3)** δ 8.19 – 8.14 (m, 3H), 7.85 (dd, *J* = 8.7, 1.8 Hz, 1H), 7.64 (d, *J* = 8.6 Hz, 1H), 1.21 – 1.15 (m, 21H). **^{13}C NMR (126 MHz, CDCl3)** δ 149.0, 146.4, 136.2, 133.6, 130.8, 130.6, 126.6, 126.4, 118.4, 110.6, 105.8, 96.1, 18.7, 11.2. **ATR-FTIR (cm^{-1})**: 2943, 2865, 2229, 1590, 1221, 838. **HRMS m/z (ESI)**: calcd for $C_{21}H_{27}N_2Si^+$ $(M + H)^+$ 335.1938, found 335.1937.

8-((Triisopropylsilyl)ethynyl)quinoline-6-carbonitrile (4ra)

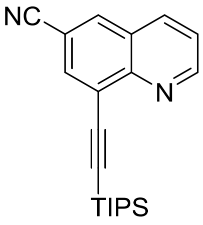

4ra

Following the ***general procedure II***, product **4ra** was obtained as a brown liquid (37.2 mg, 56% yield, 98/2 r.r.) after purification by flash column chromatography (PE/EA = 30:1). **^{1}H NMR (500 MHz, $CDCl_3$)** δ 9.11 (dd, *J* = 4.2, 1.8 Hz, 1H), 8.19 (dd, *J* = 8.3, 1.8 Hz, 1H), 8.14 (d, *J* = 1.8 Hz, 1H), 8.04 (d, *J* = 1.9 Hz, 1H), 7.53 (dd, *J* = 8.3, 4.2 Hz, 1H), 1.24 – 1.18 (m, 21H). **^{13}C NMR (126 MHz, $CDCl_3$)** δ 153.7, 149.4, 136.5, 134.4, 133.5, 127.6, 125.9, 123.0, 117.9, 110.1, 102.3, 100.8, 18.7, 11.4. **ATR-FTIR (cm^{-1})**: 2942, 2864, 2232, 1482, 1072, 884, 683. **HRMS m/z (ESI)**: calcd for $C_{21}H_{27}N_2Si^+$ $(M + H)^+$ 335.1938, found 335.1935.

3-(Phenylethynyl)-8-((triisopropylsilyl)ethynyl)quinolone (4sa)

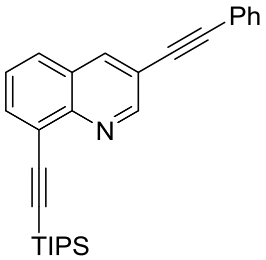

4sa

Following the ***general procedure II***, product **4sa** was obtained as a brown solid (39.4 mg, 48% yield, 97/3 r.r.) after purification by flash column chromatography (PE/EA = 30:1). **^{1}H NMR (500 MHz, $CDCl_3$)** δ 9.02 (d, *J* = 4.3 Hz, 1H), 7.96 (dd, *J* = 7.1, 1.5 Hz, 1H), 7.86 (dd, *J* = 8.4, 1.4 Hz, 1H), 7.54 – 7.46 (m, 5H), 7.41 (dd, *J* = 8.5, 7.1 Hz, 1H), 7.34 (d, *J* = 4.3 Hz, 1H), 1.27 – 1.21 (m, 21H). **^{13}C NMR (126 MHz, $CDCl_3$)** δ 150.5, 148.9, 148.5, 138.0, 134.4, 129.5, 128.5, 128.4, 126.7, 126.4, 125.8, 124.0, 121.7, 105.0, 97.2, 18.8, 11.5. **ATR-FTIR (cm^{-1})**: 2941, 2863, 2156, 1489, 884, 700. **HRMS m/z (ESI)**: calcd for $C_{28}H_{32}NSi^+$ $(M + H)^+$ 410.2299, found 410.2299.

8-((Triisopropylsilyl)ethynyl)-2,2'-biquinoline (4ta)

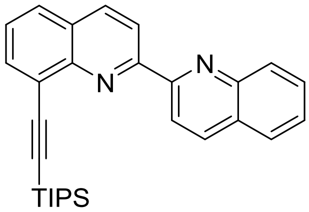

4ta

Following the ***general procedure II***, product **4ta** was obtained as a brown liquid (63.1 mg, 72% yield) after purification by flash column chromatography (PE/EA = 10:1). **^{1}H NMR (400 MHz, $CDCl_3$)** δ 8.48 (s, 1H), 8.29 – 8.19 (m, 3H), 8.01 (d, *J* = 8.4 Hz, 1H), 7.89 – 7.83 m, 2H), 7.77 – 7.72 (m, 2H), 7.61 – 7.56 (m, 2H), 0.97 – 0.88 (m, 21H). **^{13}C NMR (101 MHz,**

$CDCl_3$) δ 158.8, 157.5, 147.8, 146.6, 141.8, 136.2, 130.4, 130.2, 129.8, 129.4, 127.8, 127.5, 127.4, 127.1, 126.9, 121.8, 116.8, 103.9, 97.5, 96.0, 18.5, 11.1. **ATR-FTIR (cm^{-1})**: 2942, 2864, 2360, 1264, 732. **HRMS m/z (ESI)**: calcd for $C_{29}H_{33}N_2Si^+$ $(M + H)^+$ 437.2408, found 437.2405.

2-(3-((Tert-butyldimethylsilyl)oxy)-3-ethylpent-1-yn-1-yl)-3-methylquinoline (3ab)

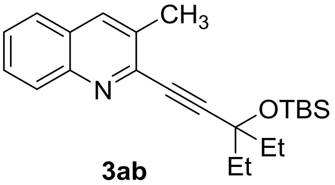

3ab

Following the ***general procedure I***,, product **3ab** was obtained as a brown liquid (13.2 mg, 18% yield, >99/1 r.r.) after purification by flash column chromatography (PE/EA = 30:1). **^{1}H NMR (500 MHz, $CDCl_3$)** δ 8.07 (d, *J* = 8.5 Hz, 1H), 7.93 (s, 1H), 7.70 (dd, *J* = 8.1, 1.4 Hz, 1H), 7.63 (ddd, *J* = 8.5, 6.8, 1.5 Hz, 1H), 7.49 (ddd, *J* = 8.0, 6.8, 1.2 Hz, 1H), 2.60 (d, *J* = 1.0 Hz, 3H), 1.84 (q, *J* = 7.4 Hz, 4H), 1.08 (t, *J* = 7.4 Hz, 6H), 0.91 (s, 9H), 0.24 (s, 6H). **^{13}C NMR (126 MHz, $CDCl_3$)** δ 146.8, 144.5, 135.1, 132.5, 129.1, 128.8, 127.5, 126.9, 126.7, 97.0, 83.6, 73.8, 35.0, 25.8, 20.0, 18.3, 8.9, -2.7. **ATR-FTIR (cm^{-1})**: 3053, 1264， 907, 737, 701, 649. **HRMS m/z (ESI)**: calcd for $C_{23}H_{34}NOSi^+$ $(M + H)^+$ 368.2404, found 368.2400.

8-(3-((Tert-butyldimethylsilyl)oxy)-3-ethylpent-1-yn-1-yl)-3-methylquinoline (4ab)

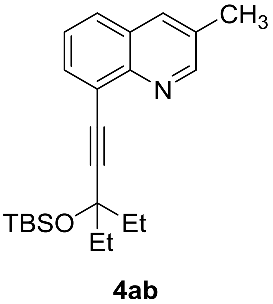

4ab

Following the ***general procedure II***, product **4ab** was obtained as a brown liquid (29.5 mg, 40% yield, >99/1 r.r.) after purification by flash column chromatography (PE/EA = 30:1). **^{1}H NMR (500 MHz, $CDCl_3$)** δ 8.82 (d, *J* = 2.3 Hz, 1H), 7.87 (dd, *J* = 2.3, 1.1 Hz, 1H), 7.78 (dd, *J* = 7.2, 1.4 Hz, 1H), 7.68 (dd, *J* = 8.2, 1.4 Hz, 1H), 7.43 (dd, *J* = 8.2, 7.2 Hz, 1H), 2.51 (d, *J* = 0.9 Hz, 3H), 1.84 (qd, *J* = 7.4, 4.0 Hz, 4H), 1.12 (t, *J* = 7.4 Hz, 6H), 0.91 (s, 9H), 0.29 (s, 6H). **^{13}C NMR (126 MHz, $CDCl_3$)** δ 152.8, 146.7, 134.6, 132.4, 130.9, 128.1, 127.3, 125.9, 123.4, 98.7, 83.0, 73.9, 35.3, 25.9, 18.6, 18.3, 8.9, -3.0. **ATR-FTIR (cm-**

1): 3053, 1264, 907, 740, 701, 649. **HRMS m/z (ESI)**: calcd for $C_{23}H_{34}NOSi^+$ $(M + H)^+$ 368.2404, found 368.2399.

2-((1-((Tert-butyldimethylsilyl)oxy)cycloheptyl)ethynyl)-3-methylquinoline (3ac)

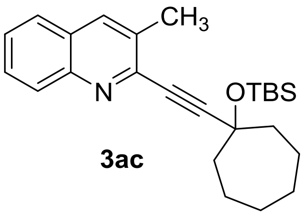

3ac

Following the ***general procedure I***,, product **3ac** was obtained as a brown liquid (22.1 mg, 28% yield, >99/1 r.r.) after purification by flash column Bchromatography (PE/EA = 30:1). **^{1}H NMR (500 MHz, CDCl$_3$)** δ 8.07 (d, *J* = 8.5 Hz, 1H), 7.92 (s, 1H), 7.70 (d, *J* = 8.1 Hz, 1H), 7.63 (ddd, *J* = 8.4, 6.7, 1.4 Hz, 1H), 7.49 (t, *J* = 7.5 Hz, 1H), 2.59 (s, 3H), 2.19 – 2.11 (m, 2H), 2.07 – 2.02 (m, 2H), 1.77 – 1.56 (m, 8H), 0.90 (s, 9H), 0.23 (s, 6H). **^{13}C NMR (126 MHz, CDCl$_3$)** δ 146.8, 144.5, 135.1, 132.6, 129.1, 128.8, 127.5, 126.9, 126.7, 99.3, 82.6, 72.9, 44.4, 28.3, 25.8, 21.9, 20.0, 18.2, -2.7. **ATR-FTIR (cm^{-1})**: 2985, 1264, 907, 732, 649. **HRMS m/z (ESI)**: calcd for $C_{25}H_{36}NOSi^+$ $(M + H)^+$ 394.2561, found 394.2556.

8-((1-((Tert-butyldimethylsilyl)oxy)cycloheptyl)ethynyl)-3-methylquinoline (4ac)

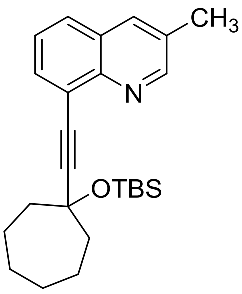

4ac

Following the ***general procedure II***, product **4ac** was obtained as a brown liquid (32.7 mg, 42% yield, >99/1 r.r.) after purification by flash column chromatography (PE/EA = 30:1). **^{1}H NMR (500 MHz, CDCl$_3$)** δ 8.82 (s, 1H), 7.87 (s, 1H), 7.77 (dd, *J* = 7.2, 2.2 Hz, 1H), 7.68 (dt, *J* = 8.1, 1.9 Hz, 1H), 7.43 (td, *J* = 7.5, 2.1 Hz, 1H), 2.51 (s, 3H), 2.17 – 2.12 (m, 2H), 2.06 – 2.01 (m, 2H), 1.69 – 1.60 (m, 8H), 0.92 (s, 9H), 0.30 (s, 6H). **^{13}C NMR (126 MHz, CDCl$_3$)** δ 152.8, 146.6, 134.6, 132.4, 130.9, 128.1, 127.2, 125.9, 123.5, 100.8, 82.0, 73.0, 44.6, 28.0, 25.9, 22.0, 18.6, 18.2, -3.0. **ATR-FTIR (cm^{-1})**: 3053, 1264, 907, 736, 701, 649. **HRMS m/z (ESI)**: calcd for $C_{25}H_{36}NOSi^+$ $(M + H)^+$ 394.2561, found 394.2553.

3-((Triisopropylsilyl)ethynyl)benzo[f]quinoline (6aa)

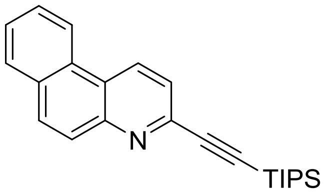

6aa

Following the ***general procedure I***, product **6aa** was obtained as a brown liquid (58.0 mg, 81% yield, >99/1 r.r.) after purification by flash column chromatography (PE/EA = 30:1). **^{1}H NMR (500 MHz, CDCl$_3$)** δ 8.85 (d, *J* = 8.5 Hz, 1H), 8.56 (d, *J* = 8.1 Hz, 1H), 7.98 (q, *J* = 9.1 Hz, 2H), 7.91 (dd, *J* = 7.7, 1.6 Hz, 1H), 7.71 – 7.63 (m, 3H), 1.22 – 1.18 (m, 21H). **^{13}C NMR (126 MHz, CDCl$_3$)** δ 148.1, 142.9, 131.8, 131.3, 130.6, 129.2, 128.7, 128.0, 127.5, 127.2, 125.4, 124.5, 122.8, 106.4, 92.8, 18.7, 11.3. **ATR-FTIR (cm^{-1})**: 2943, 2865, 1454, 1237, 829, 738. **HRMS m/z (ESI)**: calcd for $C_{24}H_{30}NSi^+$ $(M + H)^+$ 360.2142, found 360.2142.

5-((Triisopropylsilyl)ethynyl)benzo[f]quinolone (7aa)

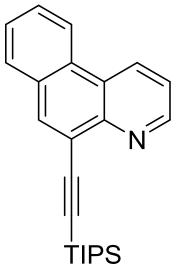

7aa

Following the ***general procedure II***, product **7aa** was obtained as a brown liquid (64.8 mg, 90% yield, 99/1 r.r.) after purification by flash column chromatography (PE/EA = 30:1). **^{1}H NMR (500 MHz, CDCl$_3$)** δ 9.04 (dd, *J* = 4.3, 1.7 Hz, 1H), 8.85 (dd, *J* = 8.4, 1.7 Hz, 1H), 8.51 (d, *J* = 8.1 Hz, 1H), 8.26 (s, 1H), 7.87 (dd, *J* = 7.8, 1.5 Hz, 1H), 7.69 – 7.59 (m, 2H), 7.54 (dd, *J* = 8.3, 4.3 Hz, 1H), 1.30 – 1.26 (m, 21H). **^{13}C NMR (126 MHz, CDCl$_3$)** δ 150.1, 147.6, 135.9, 130.9, 130.5, 129.5, 128.4, 127.6, 127.5, 125.2, 122.4, 122.2, 121.5, 104.9, 96.5, 18.8, 11.5. **ATR-FTIR (cm^{-1})**: 2941, 2863, 1463, 1074, 881, 701. **HRMS m/z (ESI)**: calcd for $C_{24}H_{30}NSi^+$ $(M + H)^+$ 360.2142, found 360.2140.

6-((Triisopropylsilyl)ethynyl)phenanthridine (6ab)

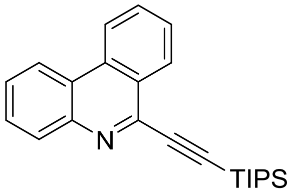

6ab

Following the ***general procedure I***, product **6ab** was obtained as a brown liquid (63.2 mg, 88% yield, >99/1 r.r.) after purification by flash column chromatography (PE/EA = 30:1). **^{1}H NMR (500 MHz, CDCl$_3$)** δ 8.63 (dd, *J* = 8.2, 1.3 Hz, 1H), 8.56 (d, *J* = 8.2 Hz, 1H), 8.50 (dd, *J* = 8.2, 1.4 Hz, 1H), 8.21

(dd, J = 8.2, 1.3 Hz, 1H), 7.86 – 7.82 (m, 1H), 7.76 – 7.70 (m, 2H), 7.67 – 7.63 (m, 1H), 1.30 – 1.23 (m, 21H). **^{13}C NMR (126 MHz, $CDCl_3$)** δ 144.8, 144.0, 132.4, 130.9, 130.2, 128.8, 128.1, 127.7, 127.6, 126.8, 123.8, 121.9, 121.9, 103.9, 96.9, 18.8, 11.4. **ATR-FTIR (cm^{-1})**: 2941, 2864, 1560, 1458, 1215, 760. **HRMS m/z (ESI)**: calcd for $C_{24}H_{30}NSi^+$ $(M + H)^+$ 360.2142, found 360.2143.

4-((Triisopropylsilyl)ethynyl)phenanthridine (7ab)

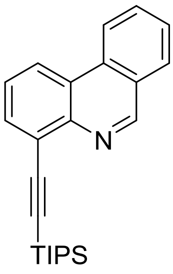

7ab

Following the ***general procedure II***, product **7ab** was obtained as a brown liquid (60.1 mg, 84% yield, 94/6 r.r.) after purification by flash column chromatography (PE/EA = 30:1). **^{1}H NMR (400 MHz, $CDCl_3$)** δ 9.36 (s, 1H), 8.57 – 8.50 (m, 2H), 8.04 (dd, J = 8.0, 1.3 Hz, 1H), 7.97 (dd, J = 7.3, 1.3 Hz, 1H), 7.86 – 7.82 (m, 1H), 7.72 – 7.68(m, 1H), 7.61 – 7.51 (m, 1H), 1.27 – 1.24 (m, 21H). **^{13}C NMR (101 MHz, $CDCl_3$)** δ 154.0, 145.1, 133.7, 132.3, 130.9, 128.6, 127.7, 126.4, 126.2, 124.3, 124.1, 122.5, 121.9, 105.3, 97.0, 18.8, 11.5. **ATR-FTIR (cm^{-1})**: 2941, 2863, 1461, 1264, 883, 731. **HRMS m/z (ESI)**: calcd for $C_{24}H_{30}NSi^+$ $(M + H)^+$ 360.2142, found 360.2138.

3-((Triisopropylsilyl)ethynyl)-4,7-phenanthroline (6ca)

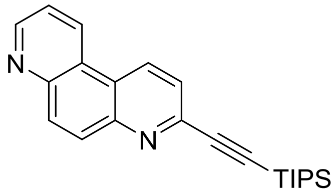

6ca

Following the ***general procedure I***, product **6ca** was obtained as a white solid (33.1 mg, 46% yield, >99/1 r.r.) after purification by flash column chromatography (PE/EA = 10:1), m.p. = 84.3-85.4 °C. **^{1}H NMR (500 MHz, $CDCl_3$)** δ 8.97 (dd, J = 4.3, 1.6 Hz, 1H), 8.79 (dd, J = 8.5, 1.6 Hz, 1H), 8.76 (d, J = 8.5 Hz, 1H), 8.21 – 8.17 (m, 2H), 7.71 (d, J = 8.5 Hz, 1H), 7.56 (dd, J = 8.3, 4.4 Hz, 1H), 1.21 – 1.15 (m, 21H). **^{13}C NMR (126 MHz, $CDCl_3$)** δ 150.5, 147.8, 147.6, 143.7, 132.5, 131.9, 130.7, 130.5, 125.7, 124.4, 123.7, 121.8, 106.0 93.7, 18.6, 11.2. **ATR-FTIR (cm^{-1})**: 2944, 2865, 2362, 1265, 907, 727. **HRMS m/z (ESI)**: calcd for $C_{23}H_{29}N_2Si^+$ $(M + H)^+$ 361.2095, found 361.2092.

3,8-Bis((triisopropylsilyl)ethynyl)-4,7-phenanthroline (6ca')

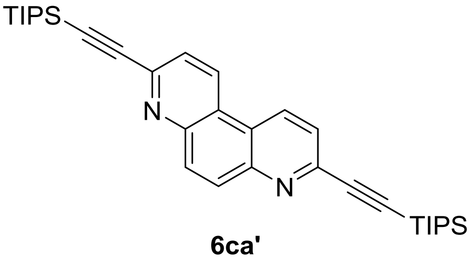

6ca'

Following the ***general procedure I***, product **6ca'** was obtained as a brown solid (46.4 mg, 43% yield, >99/1 r.r.) after purification by flash column chromatography (PE/EA = 10:1). **^{1}H NMR (500 MHz, CDCl$_3$)** δ 8.77 (d, *J* = 8.6 Hz, 2H), 8.21 (s, 2H), 7.73 (d, *J* = 8.5 Hz, 2H), 1.20 – 1.16 (m, 42H). **^{13}C NMR (126 MHz, CDCl$_3$)** δ 147.8, 143.9, 132.4, 130.7, 125.9, 123.4, 106.1, 94.0, 18.7, 11.3. **ATR-FTIR (cm^{-1})**: 2942, 2864, 1587, 1438, 1252, 717. **HRMS m/z (ESI)**: calcd for $C_{34}H_{49}N_2Si_2^+$ (M + H)$^+$ 541.3429, found 541.3427.

5-((Triisopropylsilyl)ethynyl)-4,7-phenanthroline (7ca)

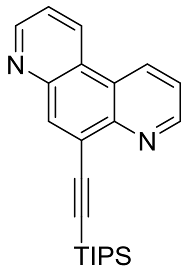

7ca

Following the ***general procedure II***, product **7ca** was obtained as a brown solid (26.9 mg, 37% yield, >99/1 r.r.) after purification by flash column chromatography (PE/EA = 10:1), m.p. = 94.0-94.9 °C. **^{1}H NMR (400 MHz, CDCl$_3$)** δ 9.08 (dd, *J* = 4.3, 1.6 Hz, 1H), 8.99 (dd, *J* = 4.4, 1.6 Hz, 1H), 8.85 – 8.81 (m, 2H), 8.48 (s, 1H), 7.62 – 7.55 (m, 2H), 1.25 – 1.20 (m, 21H). **^{13}C NMR (101 MHz, CDCl$_3$)** δ 150.84, 150.82, 147.2, 147.0, 136.9, 130.5, 130.4, 126.3, 124.9, 124.6, 121.98, 121.97, 104.1, 99.1, 18.8, 11.5. **ATR-FTIR (cm^{-1})**: 2943, 2864, 1471, 1373, 1076, 907, 785. **HRMS m/z (ESI)**: calcd for $C_{23}H_{29}N_2Si^+$ (M + H)$^+$ 361.2095, found 361.2092.

5,6-Bis((triisopropylsilyl)ethynyl)-4,7-phenanthroline (7ca')

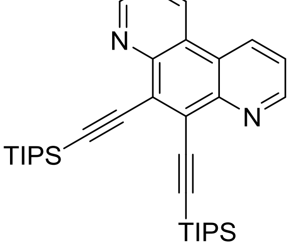

7ca'

Following the ***general procedure II***, product **7ca'** was obtained as a brown solid (41.9 mg, 39% yield, >99/1 r.r.) after purification by flash column chromatography (PE/EA = 10:1), m.p. = 197.5-198.8 °C. **^{1}H NMR (500 MHz, CDCl$_3$)** δ 8.99 (dd, *J* = 4.3, 1.6 Hz, 2H), 8.70 (dd, *J* = 8.4, 1.6 Hz, 2H), 7.51 (dd, *J* = 8.3, 4.3 Hz, 2H), 1.30 – 1.25 (d, *J* = 3.0 Hz, 42H). **^{13}C NMR (126 MHz, CDCl$_3$)** δ 150.8, 147.4, 130.1, 128.6, 124.2, 122.0, 105.4, 103.1, 18.9,

11.5. **ATR-FTIR (cm^{-1})**: 2942, 2864, 2362, 1460, 1020, 884. **HRMS m/z (ESI)**: calcd for $C_{34}H_{49}N_2Si_2^+$ (M + H)$^+$ 541.3429, found 541.3428.

8-((Triisopropylsilyl)ethynyl)-1,7-phenanthroline (6da)

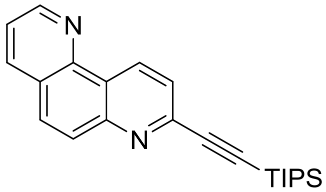

6da

Following the ***general procedure I***, product **6da** was obtained as a yellow solid (45.4 mg, 63% yield, 99/1 r.r.) after purification by flash column chromatography (PE/EA = 10:1), m.p. = 72.6-74.0 °C. **^{1}H NMR (500 MHz, $CDCl_3$)** δ 9.48 (d, *J* = 8.4 Hz, 1H), 9.02 (dd, *J* = 4.4, 1.7 Hz, 1H), 8.22 (dd, *J* = 8.1, 1.7 Hz, 1H), 8.08 (d, *J* = 9.1 Hz, 1H), 7.93 (d, *J* = 9.1 Hz, 1H), 7.79 (d, *J* = 8.4 Hz, 1H), 7.58 (dd, *J* = 8.0, 4.4 Hz, 1H), 1.23 – 1.17 (m, 21H). **^{13}C NMR (126 MHz, $CDCl_3$)** δ 149.5, 149.4, 145.5, 144.5, 136.0, 132.6, 129.5, 128.9, 126.4, 126.0, 125.9, 122.5, 106.4, 93.6, 18.7, 11.3. **ATR-FTIR (cm^{-1})**: 2943, 2865, 1246, 1264, 883, 736. **HRMS m/z (ESI)**: calcd for $C_{23}H_{29}N_2Si^+$ (M + H)$^+$ 361.2095, found 361.2095.

10-((Triisopropylsilyl)ethynyl)-1,7-phenanthroline (7da')

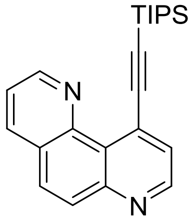

7da'

Following the ***general procedure II***, product **7da'** was obtained as a white solid (37.1 mg, 51% yield, 99/1 r.r.) after purification by flash column chromatography (PE/EA = 10:1), m.p. = 105.3-107.2 °C. **^{1}H NMR (500 MHz, $CDCl_3$)** δ 9.02 (dd, *J* = 4.3, 1.8 Hz, 1H), 8.92 (d, *J* = 4.7 Hz, 1H), 8.19 (dd, *J* = 8.1, 1.8 Hz, 1H), 8.04 (d, *J* = 9.0 Hz, 1H), 7.91 (d, *J* = 9.0 Hz, 1H), 7.81 (d, *J* = 4.7 Hz, 1H), 7.56 (dd, *J* = 8.0, 4.3 Hz, 1H), 1.27 – 1.24 (m, 21H). **^{13}C NMR (126 MHz, $CDCl_3$)** δ 150.5, 149.5, 148.4, 145.9, 135.3, 129.5, 129.5, 129.3, 129.1, 126.5, 125.7, 122.3, 106.7, 103.2, 18.8, 11.5. **ATR-FTIR (cm^{-1})**: 2942, 2864, 2361, 1565, 1000, 853. **HRMS m/z (ESI)**: calcd for $C_{23}H_{29}N_2Si^+$ (M + H)$^+$ 361.2095, found 361.2091.

4-((Triisopropylsilyl)ethynyl)acridine (7ea)

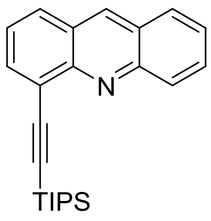
7ea

Following the ***general procedure II***, product **7ea** was obtained as a yellow solid (61.2 mg, 85% yield) after purification by flash column chromatography (PE/EA = 30:1). **^{1}H NMR (400 MHz, CDCl$_3$)** δ 8.69 (s, 1H), 8.28 (dd, *J* = 8.8, 1.1 Hz, 1H), 8.02 (dd, *J* = 7.0, 1.4 Hz, 1H), 7.96 – 7.91 (m, 2H), 7.80 – 7.76 (m, 1H), 7.55 – 7.51 (m, 1H), 7.46 – 7.42 (dd, *J* = 8.4, 6.9 Hz, 1H), 1.33 – 1.28 (m, 21H). **^{13}C NMR (101 MHz, CDCl$_3$)** δ 149.4, 148.9, 136.0, 134.6, 130.3, 130.2, 128.7, 127.8, 126.6, 126.3, 126.0, 124.9, 123.7, 104.9, 97.7, 18.9, 11.6. **ATR-FTIR (cm^{-1})**: 2942, 2864, 2362, 1462, 1264, 734. **HRMS m/z (ESI)**: calcd for $C_{24}H_{30}NSi^+$ $(M + H)^+$ 360.2142, found 360.2138.

2.3 Synthetic Applications on Regiodivergent C-H Alkynylation of Benzoazines.

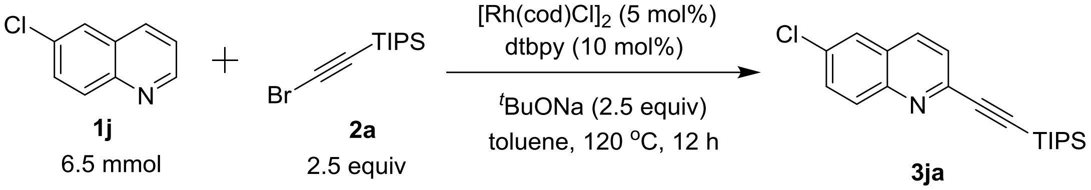

Under argon atmosphere, a flame-dried 100 mL Schlenk tube was added [Rh(cod)Cl]$_2$ (160.3 mg, 0.325 mmol, 5 mol%), dtbpy (174.5 mg, 0.65 mmol, 10 mol%) and anhydrous toluene (32.5 ml). The resulting solution was stirred for 30 min at room temperature. ^{t}BuONa (1.56 mg, 16.25 mmol, 2.5 equiv) was added in the glove box. Then **1j** (6.5 mmol, 1.06 g) and **2a** (4.23 g, 16.25 mmol, 2.5 equiv) were added and the tube was sealed. Then heated at 120 ℃ for 12 h. Afterwards, the mixture was cooled to room temperature. The mixture was diluted with DCM (~20 mL), silica gel was added and the solvent was evaporated under reduced pressure. It was purified by silica gel chromatography (PE/EA = 30:1) to afford the desired product **3ja** as a brown liquid (2.15 g, 96% yield, 96/4 r.r.).

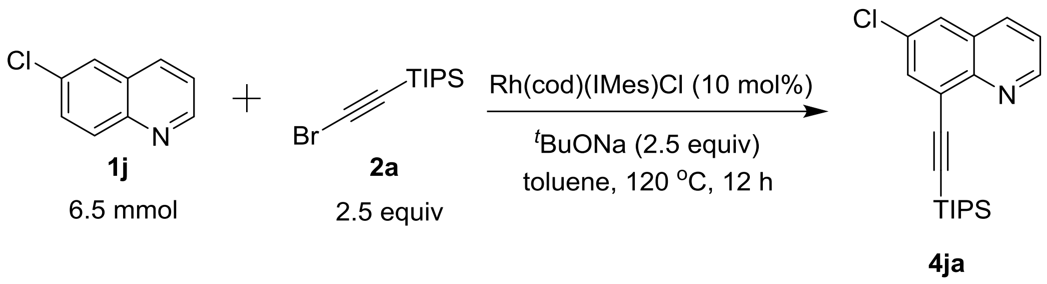

Under argon atmosphere, a flame-dried 100 mL Schlenk tube was added Rh(cod)(IMes)Cl (358 mg, 0.65 mmol, 10 mol%) and ^{t}BuONa (1.56 g, 16.25 mmol, 2.5 equiv) in the glove box. Then **1j** (6.5 mmol), **2a** (4.23 g, 16.25 mmol, 2.5 equiv) and anhydrous toluene (32.5 ml) were added and the tube was sealed. The resulting solution was stirred at 120 ℃ for 12 h. Afterwards, the mixture was cooled to room temperature. The mixture was diluted with DCM (~20 mL), silica gel was added and the solvent was evaporated under reduced pressure. It was purified by silica gel chromatography (PE/EA = 30:1) to afford the desired product **4ja** as a brown liquid (1.91 g, 85% yield, 97/3 r.r.).

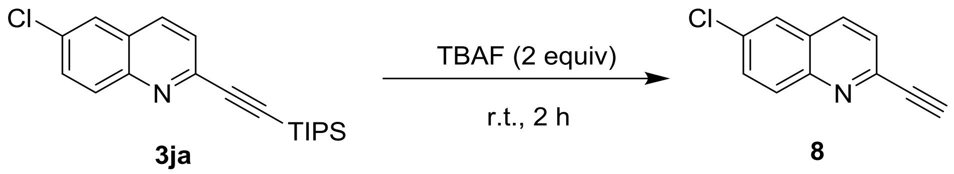

To a 25 mL Schlenk tube was added **3ja** (1.37 g, 4 mmol, 1.0 equiv) under Ar. And then TBAF (1M in THF) was added dropwise (8 ml, 8 mmol, 2.0 equiv). The solution stirred at room temperature for 2 hours. The reaction was quenched with 10 mL of H_2O and extracted with DCM 3×20 ml. The organic layer was dried over $MgSO_4$ and concentrated in vacuo. The crude product was purified by flash column chromatography using silica (PE/EA = 5:1) and the title compound **8** was isolated as a brown solid in 70% yield (523.8 mg), m.p. = 170.1-172.3 °C. **^{1}H NMR (400 MHz, $CDCl_3$)** δ 8.04 (dd, *J* = 8.7, 7.0 Hz, 2H), 7.79 (d, *J* = 2.4 Hz, 1H), 7.66 (dd, *J* = 9.1, 2.3 Hz, 1H), 7.56 (d, *J* = 8.5 Hz, 1H), 3.28 (s, 1H). **^{13}C NMR (101 MHz, $CDCl_3$)** δ 146.4, 142.6, 135.3, 133.3, 131.2, 131.0, 127.9, 126.2, 125.0, 83.0, 78.2. **ATR-FTIR (cm^{-1})**: 3163, 2923, 2098, 1590, 1071, 830. **HRMS m/z (ESI)**: calcd for $C_{11}H_7ClN^+$ $(M + H)^+$ 188.0262, found 188.0264.

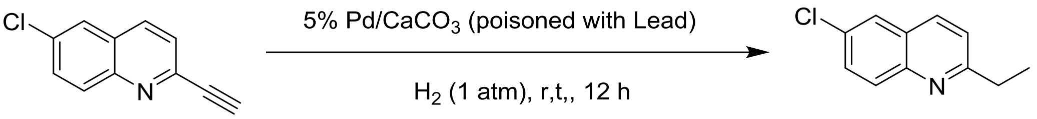

To the **8** (18.7 mg, 0.1 mmol, 1.0 euqiv) in MeOH (2 mL), was added palladium on calclum carbonate (5% on Calclum Caronate poisoned with Lead) (5.3 mg, 0.05 mmol, 5 mol%). The reaction solution was purged with hydrogen balloon for 15 minutes and then stirred at room temperature for 12 h under hydrogen balloon. Then, the reaction was filtered over a short path of Celite, concentrated in vacuo, and the crude mixture was purified by flash column chromatography (PE/EA = 5:1) to afford the final product **9a** (15.3 mg, 80% yield) as a yellow liquid. **^{1}H NMR (500 MHz, $CDCl_3$)** δ 7.97 (dd, *J* = 8.7, 4.0 Hz, 2H), 7.74 (d, *J* = 2.3 Hz, 1H), 7.60 (dd, *J* = 9.0, 2.4 Hz, 1H), 7.32 (d, *J* = 8.5 Hz, 1H), 2.99 (q, *J* = 7.6 Hz, 2H), 1.38 (t, *J* = 7.6 Hz, 3H). **^{13}C NMR (126 MHz, $CDCl_3$)** δ 164.3, 146.2, 135.4, 131.2, 130.4, 130.2, 127.3, 126.1, 121.7, 32.2, 13.8. **ATR-FTIR (cm^{-1})**: 2970, 2933, 1599, 1450, 1302, 832. **HRMS m/z (ESI)**: calcd for $C_{11}H_{11}ClN^+$ $(M + H)^+$ 192.0575, found 192.0576.

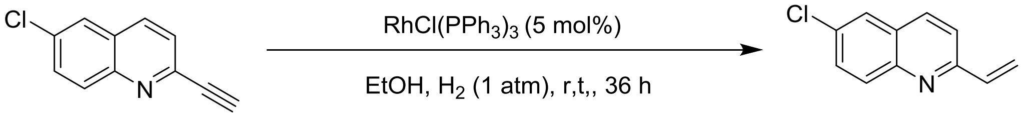

To the **8** (37.4 mg, 0.2 mmol, 1.0 euqiv) in EtOH (2 mL), was added $RhCl(PPh_3)_3$ (9.3 mg, 0.01 mmol, 5 mol%). The reaction solution was purged with hydrogen balloon for 15 minutes and then stirred at room temperature for 36 h under hydrogen balloon. Then, the reaction was filtered over a short path of Celite, concentrated in vacuo, and the crude mixture was purified by flash column chromatography (PE/EA = 5:1) to afford the final product **9b** (16.4 mg, 43% yield) as a yellow solid, m.p. = 70.9-72.3 ^{o}C. **^{1}H NMR (500 MHz, $CDCl_3$)** δ 8.01 (dd, *J* = 17.1, 8.8 Hz, 2H), 7.76 (d, *J* = 2.3 Hz, 1H), 7.63 – 7.60 (m, 2H), 7.01 (dd, *J* = 17.7, 10.9 Hz, 1H), 6.29 (d, *J* = 17.7 Hz, 1H), 5.68 (d, *J* = 10.9 Hz, 1H). **^{13}C NMR (126 MHz, $CDCl_3$)** δ 156.3, 146.4, 137.6, 135.4, 132.0, 130.9, 130.5, 128.0, 126.2, 120.4, 119.3. **ATR-FTIR (cm^{-1})**: 2924, 2853, 1610, 1497, 1073, 927. **HRMS m/z (ESI)**: calcd for $C_{11}H_9ClN^+$ $(M + H)^+$ 190.0418, found 190.0420.

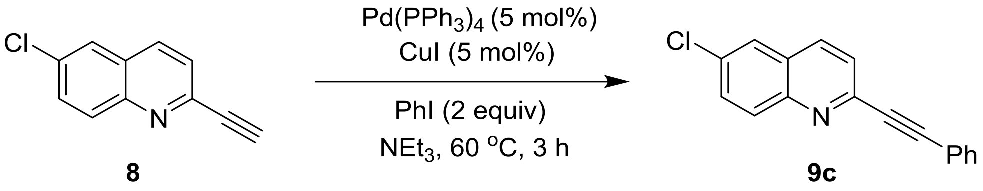

A 25 mL Schlenk tube was charged with **8** (37.4 mg, 0.20 mmol, 1.0 equiv), CuI (1.9 mg, 0.01 mmol, 5 mol%), $Pd(PPh_3)_4$ (11.6 mg, 0.01 mmol, 5 mol%), PhI (81.6 mg, 0.4 mmol, 2.0 equiv) and anhydrous NEt_3 (2 mL) under Ar atmosphere. The reaction mixture was stirred at 60 °C for 3 h. Afterwards, the mixture was cooled to room temperature. The mixture was diluted with DCM (~5 mL), silica gel was added and the solvent was evaporated under reduced pressure. It was purified by silica gel chromatography (PE/EA = 10:1) to afford the final product **9c** (51.8 mg, 98% yield) as a yellow solid, m.p. = 146.9-148.3 °C. **^{1}H NMR (500 MHz, $CDCl_3$)** δ 9.01 (dd, *J* = 4.1, 1.7 Hz, 1H), 8.05 (dd, *J* = 8.4, 1.7 Hz, 1H), 7.92 (d, *J* = 2.3 Hz, 1H), 7.74 (d, *J* = 2.4 Hz, 1H), 7.71 – 7.65 (m, 2H), 7.43 (dd, *J* = 8.3, 4.2 Hz, 1H), 7.39 – 7.35 (m, 3H). **^{13}C NMR (126 MHz, $CDCl_3$)** δ 151.0, 146.4, 135.5, 134.2, 132.0, 131.6, 128.8, 128.7, 128.2, 126.8, 125.2, 122.8, 122.3, 96.5, 85.9. **ATR-FTIR (cm^{-1})**: 3075, 2918, 2195, 1589, 1250, 732. **HRMS m/z (ESI)**: calcd for $C_{17}H_{11}ClN^+$ $(M + H)^+$ 264.0575, found 264.0573.

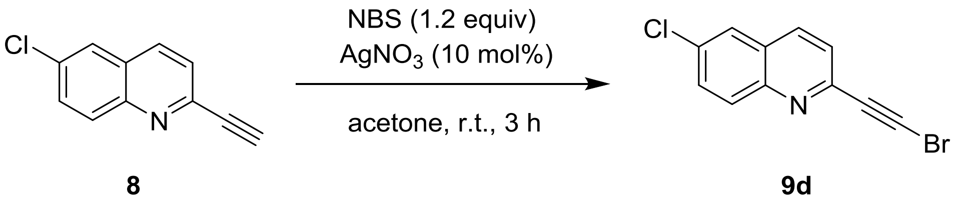

A 25 mL Schlenk tube was charged with **8** (37.4 mg, 0.20 mmol, 1.0 equiv), NBS (42.7 mg, 0.24 mmol, 1.2 equiv), $AgNO_3$ (3.4 mg, 0.02 mmol, 10 mol%) and anhydrous acetone (1 mL) under Ar atmosphere. The reaction mixture was stirred at at room temperature for 3 h. Afterwards, the mixture was diluted with DCM (~5 mL), silica gel was added and the solvent was evaporated under reduced pressure. It was purified by silica gel chromatography (PE/EA = 30:1) to afford the final product **9d** (26.4 mg, 50% yield) as a white solid, m.p. = 168.3-170.2 °C. **^{1}H NMR (400 MHz, $CDCl_3$)** δ 7.99 (t, *J* = 9.3 Hz, 2H), 7.75 (d, *J* = 2.4 Hz, 1H), 7.64 (dd, *J* = 8.9, 2.4 Hz, 1H), 7.48 (d, *J* =

8.4 Hz, 1H). **^{13}C NMR (101 MHz, CDCl$_3$)** δ 146.3, 142.9, 135.3, 133.2, 131.2, 130.8, 127.7, 126.1, 124.9, 80.1, 53.3. **ATR-FTIR (cm^{-1})**: 2924, 2853, 2363, 1589, 1485, 1074, 827. **HRMS m/z (ESI)**: calcd for $C_{11}H_6BrClN^+$ (M + H)$^+$ 265.9367, found 265.9369.

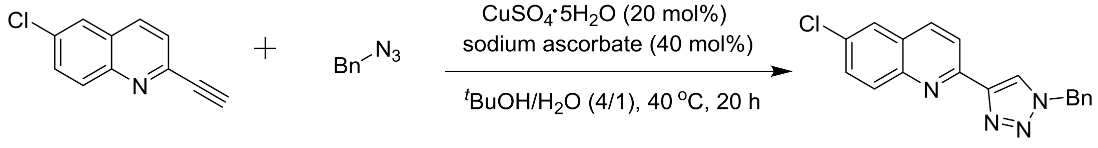

8 **9e**

To a stirred solution of the benzyl azide (16.0 mg, 0.12 mmol, 1.2 equiv) in ^{t}BuOH/H_2O (4:1, 2 mL) was added the compound **8** (18.7 mg, 0.1 mmol), CuSO4 5H_2O (5.0 mg, 0.02 mmol, 20 mol%) and sodium ascorbate (8.0 mg, 0.04 mmol, 40 mol%). The resulting solution was stirred at 40 ℃ for 20 h. The reaction mixture was diluted with H_2O (10 mL) and extracted with DCM (3 × 20 mL). The combined organic layers were washed H_2O (20 mL), dried (Na_2SO_4), filtered and the solvent was evaporated under reduced pressure. It was purified by silica gel chromatography (PE/EA = 2:1) to afford the final product **9e** (23.0 mg, 72% yield) as a white solid, m.p. = 185.3-187.2 °C. **^{1}H NMR (500 MHz, CDCl$_3$)** δ 8.34 (d, *J* = 8.6 Hz, 1H), 8.24 (s, 1H), 8.12 (d, *J* = 8.6 Hz, 1H), 7.91 (d, *J* = 8.9 Hz, 1H), 7.77 (d, *J* = 2.3 Hz, 1H), 7.60 (dd, *J* = 9.0, 2.3 Hz, 1H), 7.41 – 7.34 (m, 5H), 5.61 (s, 2H). **^{13}C NMR (126 MHz, CDCl$_3$)** δ 150.6, 148.6, 146.3, 135.9, 134.3, 131.9, 130.6, 130.4, 129.2, 128.9, 128.2, 126.4, 122.8, 119.5, 54.4. **ATR-FTIR (cm^{-1})**: 3361, 2922, 2851, 2362, 1602, 1488, 1233, 879. **HRMS m/z (ESI)**: calcd for $C_{18}H_{14}ClN_4^+$ (M + H)$^+$ 321.0902, found 321.0898.

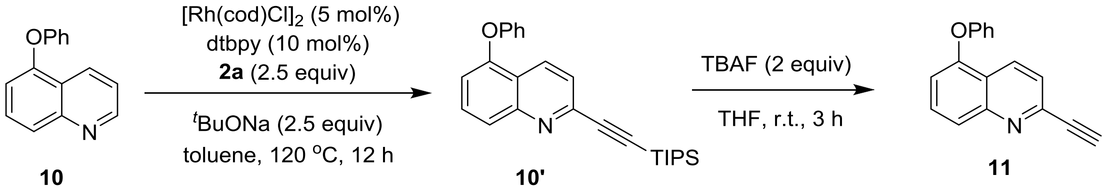

10 **10'** **11**

Under argon atmosphere, a flame-dried 25 mL Schlenk tube was added [Rh(cod)Cl]$_2$ (9.9 mg, 0.02 mmol, 5 mol%), dtbpy (10.7 mg, 0.04 mmol, 10 mol%) and anhydrous toluene (2 ml). The resulting solution was stirred for 30 min at room temperature. ^{t}BuONa (96.1 mg, 1.0 mmol, 2.5 equiv) was added in the glove box. Then

10 (0.4 mmol, 88.4 mg) and **2a** (260.1 mg, 1.0 mmol, 2.5 equiv) were added and the tube was sealed. Then heated at 120 ℃ for 12 h. Afterwards, the mixture was cooled to room temperature. The mixture was diluted with DCM (~5 mL), silica gel was added and the solvent was evaporated under reduced pressure. It was purified by silica gel chromatography (PE/EA = 30:1) to afford the desired product **10'** as a brown liquid (146.2 mg, 91% yield，99/1 r.r.). **^{1}H NMR (500 MHz, $CDCl_3$)** δ 8.49 (dd, *J* = 8.6, 0.8 Hz, 1H), 7.88 – 7.86 (m, 1H), 7.60 – 7.55 (m, 1H), 7.54 (d, *J* = 8.6 Hz, 1H), 7.39 – 7.35 (m, 2H), 7.18 – 7.14 (m, 1H), 7.06 – 7.05 (m, 2H), 6.93 (dd, *J* = 7.8, 0.9 Hz, 1H), 1.20 – 1.16 (m, 21H). **^{13}C NMR (126 MHz, $CDCl_3$)** δ 157.0, 152.8, 149.1, 144.1, 130.7, 130.0, 129.6, 124.8, 124.3, 123.9, 120.7, 119.0, 113.4, 106.4, 93.4, 18.7, 11.3. **ATR-FTIR (cm^{-1})**: 2942, 2864, 1585, 1488, 1224, 905, 726. **HRMS m/z (ESI)**: calcd for $C_{26}H_{32}NOSi^+$ $(M + H)^+$ 402.2248, found 402.2245.

To a 25 mL Schlenk tube was added **10'** (0.1 mmol, 40.1 mg, 1.0 equiv) and anhydrous THF (2 ml) under Ar. And then TBAF (1M in THF) was added dropwise (0.2 ml, 0.2 mmol, 2.0 equiv). The solution stirred at room temperature for 3 hours. The reaction was quenched with 10 mL of H_2O and extracted with DCM 3×20 ml. The organic layer was dried over $MgSO_4$ and concentrated in vacuo. The crude product was purified by flash column chromatography using silica (PE/EA = 10:1) and the title compound **11** was isolated as a brown solid in 80% yield (19.6 mg), m.p. = 115.8-117.0 ^{o}C. **^{1}H NMR (500 MHz, $CDCl_3$)** δ 8.56 (dd, *J* = 8.6, 0.9 Hz, 1H), 7.85 (dt, *J* = 8.5, 0.9 Hz, 1H), 7.62 – 7.59 (m, 1H), 7.55 (d, *J* = 8.6 Hz, 1H), 7.41 – 7.36 (m, 2H), 7.19 – 7.16 (m, 1H), 7.08 – 7.05 (m, 2H), 6.94 (dd, *J* = 7.7, 1.0 Hz, 1H), 3.28 (s, 1H). **^{13}C NMR (126 MHz, $CDCl_3$)** δ 156.8, 153.0, 149.0, 143.0, 131.2, 130.0, 129.9, 124.2, 124.0, 123.9, 121.0, 119.2, 113.4, 83.2, 78.0. **ATR-FTIR (cm^{-1})**: 3289, 3065, 2362, 1585, 1488, 1221, 905, 723. **HRMS m/z (ESI)**: calcd for $C_{17}H_{12}NO^+$ $(M + H)^+$ 246.0913, found 246.0913.

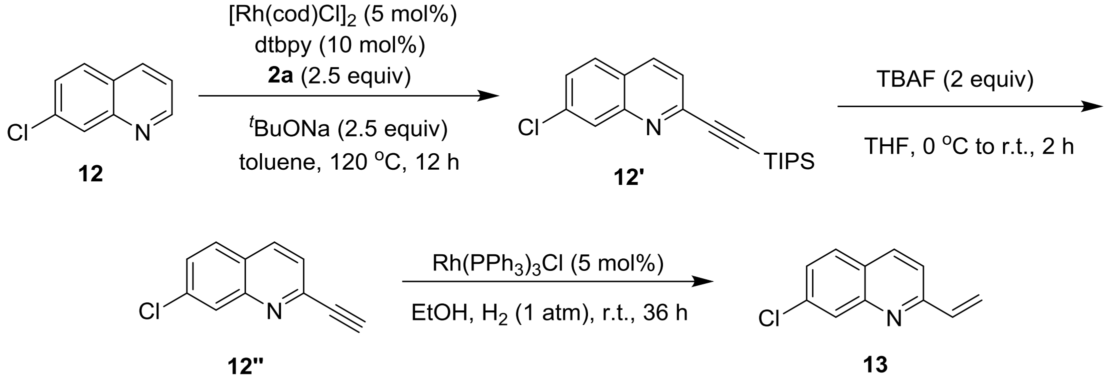

Under argon atmosphere, a flame-dried 25 mL Schlenk tube was added $[Rh(cod)Cl]_2$ (24.7 mg, 0.05 mmol, 5 mol%), dtbpy (26.8 mg, 0.1 mmol, 10 mol%) and anhydrous toluene (5 ml). The resulting solution was stirred for 30 min at room temperature. ^{t}BuONa (240.3 mg, 2.5 mmol, 2.5 equiv) was added in the glove box. Then **12** (1 mmol, 163.6 mg) and **2a** (650.2 mg, 2.5 mmol, 2.5 equiv) were added and the tube was sealed. Then heated at 120 ℃ for 12 h. Afterwards, the mixture was cooled to room temperature. The mixture was diluted with DCM (~10 mL), silica gel was added and the solvent was evaporated under reduced pressure. It was purified by silica gel chromatography (PE/EA = 30:1) to afford the desired product **12'** as a brown liquid (263.3 mg, 77% yield，98/2 r.r.). **^{1}H NMR (500 MHz, $CDCl_3$)** δ 8.10 (d, *J* = 2.0 Hz, 1H), 8.06 (d, *J* = 8.4 Hz, 1H), 7.70 (d, *J* = 8.7 Hz, 1H), 7.52 (d, *J* = 8.4 Hz, 1H), 7.47 (dd, *J* = 8.7, 2.1 Hz, 1H), 1.18 – 1.15 (m, 21H). **^{13}C NMR (126 MHz, $CDCl_3$)** δ 148.4, 144.5, 135.8, 135.7, 128.6, 128.3, 128.0, 125.5, 125.2, 106.1, 93.9, 18.7, 11.2. **ATR-FTIR (cm^{-1})**: 2942, 2865, 1612, 1494, 1214, 779. **HRMS m/z (ESI)**: calcd for $C_{20}H_{27}ClNSi^+$ $(M + H)^+$ 344.1596, found 344.1595.

To a 25 mL Schlenk tube was added **12'** (34.3 mg, 0.1 mmol, 1.0 equiv) under Ar. And then TBAF (1M in THF) was added dropwise (0.2 ml, 0.2 mmol, 2.0 equiv) at 0 °C and then warmed to room temperature and allowed to stirr for 2 hours. The reaction was quenched with 10 mL of H_2O and extracted with DCM 3×20 ml. The organic layer was dried over $MgSO_4$ and concentrated in vacuo. The crude product was purified by flash column chromatography using silica (PE/EA = 3:1) and the title compound **12''** was isolated as a brown solid in 84% yield (15.7 mg), m.p. = 119.8-120.9 °C. **^{1}H NMR**

(500 MHz, $CDCl_3$) δ 8.10 (dd, *J* = 8.5, 0.8 Hz, 1H), 8.08 (d, *J* = 2.0 Hz, 1H), 7.73 (d, *J* = 8.7 Hz, 1H), 7.52 (d, *J* = 8.5 Hz, 1H), 7.50 (dd, *J* = 8.7, 2.0 Hz, 1H), 3.28 (s, 1H). **^{13}C NMR (126 MHz, $CDCl_3$)** δ 148.3, 143.4, 136.11, 136.09, 128.7, 128.4, 128.3, 125.7, 124.3, 83.0, 78.4. **ATR-FTIR (cm^{-1})**: 3289, 2925, 2110, 1492, 1127, 844. **HRMS m/z (ESI)**: calcd for $C_{11}H_7ClN^+$ $(M + H)^+$ 188.0262, found 188.0264.

To the **12''** (18.7 mg, 0.1 mmol, 1.0 euqiv) in EtOH (1 mL), was added $RhCl(PPh_3)_3$ (4.7 mg, 0.005 mmol, 5 mol%). The reaction solution was purged with hydrogen balloon for 15 minutes and then stirred at room temperature for 36 h under hydrogen balloon. Then, the reaction was filtered over a short path of Celite, concentrated in vacuo, and the crude mixture was purified by flash column chromatography (PE/EA = 5:1) to afford the final product **13** (9.8 mg, 52% yield) as a yellow solid, m.p. = 72.6-73.9 °C. **^{1}H NMR (500 MHz, $CDCl_3$)** δ 8.09 – 8.06 (m, 2H), 7.71 (d, *J* = 8.6 Hz, 1H), 7.58 (d, *J* = 8.5 Hz, 1H), 7.45 (dd, *J* = 8.7, 2.1 Hz, 1H), 7.00 (dd, *J* = 17.7, 10.9 Hz, 1H), 6.31 (dd, *J* = 17.6, 0.8 Hz, 1H), 5.69 (dd, *J* = 10.8, 0.8 Hz, 1H). **^{13}C NMR (126 MHz, $CDCl_3$)** δ 157.0, 148.4, 137.5, 136.2, 135.4, 128.6, 128.3, 127.3, 125.8, 120.6, 118.7. **ATR-FTIR (cm^{-1})**: 2926, 2360, 1610, 1497, 1131, 926, 845. **HRMS m/z (ESI)**: calcd for $C_{11}H_9ClN^+$ $(M + H)^+$ 190.0418, found 190.0420.

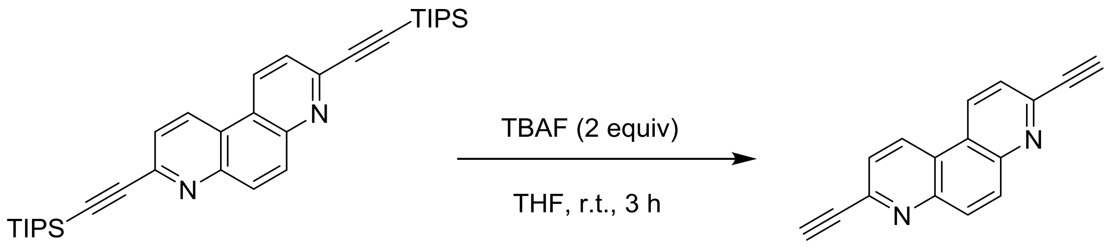

6ca' **15**

To a 25 mL Schlenk tube was added **6ca'** (0.05 mmol, 27.0 mg, 1.0 equiv) and anhydrous THF (1 ml) under Ar. And then TBAF (1M in THF) was added dropwise (0.1 ml, 0.1 mmol, 2.0 equiv). The solution stirred at room temperature for 3 hours. The reaction was quenched with 5 mL of H_2O and extracted with DCM 3×20 ml. The organic layer was dried over $MgSO_4$ and concentrated in vacuo. The crude product was

purified by flash column chromatography using silica (PE/EA = 3:1) and the title compound **15** (8.4 mg, 74% yield) was isolated as a brown solid, m.p. > 230 °C. **^{1}H NMR (500 MHz, $CDCl_3$)** δ 8.83 (d, *J* = 8.5 Hz, 2H), 8.23 (s, 2H), 7.77 (d, *J* = 8.5 Hz, 2H), 3.34 (s, 2H). **^{13}C NMR (126 MHz, $CDCl_3$)** δ 148.0, 143.0, 132.6, 131.1, 125.2, 123.8, 82.8, 78.8. **ATR-FTIR (cm^{-1})**: 3278, 2922, 2362, 1469, 905, 825. **HRMS m/z (ESI)**: calcd for $C_{16}H_9N_2^+$ $(M + H)^+$ 229.0760, found 229.0761.

2.4 Computational Details.

The calculations were carried out using Gaussian 09 package.[5] All geometry optimizations were conducted at the B3LYP-D3[6-7] level of theory with a mixed basis set of SDD[8-9] for Rh and 6-31G(d)[10-12] for other atoms in toluene with SMD[13] continuum solvation model. The frequency calculations were conducted at the same level of theory to determine the structures to be minima (no imaginary frequency) or transition states (only one imaginary frequency) and to obtain the thermal correction to free energies. Solvation single-point energy calculations were conducted at the M06-D3[14]/6-311+G(d,p)[15-16]-SDD level of theory with SMD toluene solvation model. All energies are reported here in kcal/mol and the calculated 3D optimized structures are displayed using CYLview visualization program.[17] The NPA analysis was performed at the level of SMD(toluene)-B3LYP-D3/6-311+G(d,p) -SDD level of theory.

Three DFT functional (B3LYP-D3, M06-D3, and wb97x-D) with SDD for Rh and 6-311+G(d,p) for other atoms was further used to compute the solvation single-point energies in toluene with SMD continuum model. The calculated energies were shown in Table S1. The calculated results indicated the solvation single-point energies of key transition states using M06-D3 functional is well agreement with the experimental observed site selectivity.[18-25]

Supplementary Table 2. The absolute (in Hartree) and relative (in kcal mol^{-1}) single-point energies in toluene as solution with SMD model by different DFT functional.

structure	G_{corr}	$E_{B3LYP\text{-}D3}$	$\Delta G_{B3LYP\text{-}D3}$	$E_{M06\text{-}D3}$	$\Delta G_{M06\text{-}D3}$	$E_{\omega b97xd}$	$\Delta G_{\omega b97xd}$
**INT3A-dtbpy**	0.860271	-4851.755973	0.0	-4849.976913	0.0	-4851.020005	0.0
**TS4A-dtbpy**	0.8555	-4851.702302	30.7	-4849.931004	25.8	-4850.96641	30.6
**TS4B-dtbpy**	0.856409	-4851.686011	41.5	-4849.918839	34.0	-4850.95572	37.9
**TS4C-dtbpy**	0.851927	-4851.679471	42.8	-4849.901695	42.0	-4850.943095	43.0
**TS4D-dtbpy**	0.854565	-4851.677143	45.9	-4849.901262	43.9	-4850.940878	46.1
**INT4A-dtbpy**	0.721759	-4617.949204	4.3	-4616.35412	4.3	-4617.300563	3.9
**INT4B-dtbpy**	0.725961	-4617.961323	-0.7	-4616.36335	1.1	-4617.3156	-2.9
**INT3A-IMes**	0.874798	-4966.076189	0	-4964.217692	0	-4965.300254	0
**TS4A-IMes**	0.871061	-4965.998936	46.1	-4964.144839	43.4	-4965.222756	46.3
**TS4B-IMes**	0.867808	-4966.024905	27.8	-4964.169054	26.1	-4965.250054	27.1
**TS4C-IMes**	0.86544	-4966.005256	38.6	-4964.148857	37.3	-4965.227737	39.6
**TS4D-IMes**	0.867027	-4966.000563	42.6	-4964.148247	38.7	-4965.224746	42.5
**INT4A-IMes**	0.73626	-4732.284845	-5.4	-4730.60852	-4.2	-4731.596793	-6.1
**INT4B-IMes**	0.735119	-4732.271978	2.0	-4730.591349	5.8	-4731.584638	0.8

For the ligand dtbpy, the energy profile for the C-H activation was shown in **Supplementary Figure 3**. The C2-H bond of quinoline undergoes direct oxidative addition to the Rh(I) center without the directing of nitrogen atom through transition state **TS2B-dtbpy** with a free energy barrier of 26.2 kcal mol^{-1}, which is much lower than that process through three-membered metallacycle transition state **TS2C-dbtpy** (26.2 vs 36.9 kcal mol^{-1}). Both reductive elimination and insertion into alkyne **2a** of **INT2B-dtbpy** need to overcome high activation energy barriers (total energy barriers of 35.6 and 43.4 kcal mol^{-1}, respectively), whereas reversible C-H bond oxidative addition requires an activation energy of 7.4 kcal mol^{-1}, suggesting that the direct C-H

activation without the formation of three-membered metallacycle might be a reversible process. Alternatively, a ^{t}butoxide-assisted deprotonation-type C-H activation was also calculated. The energy in this pathway through transition state **TS2D-dtbpy** is much higher than that the C-Br bond oxidative addition through transition state **TS2A-dtbpy**.

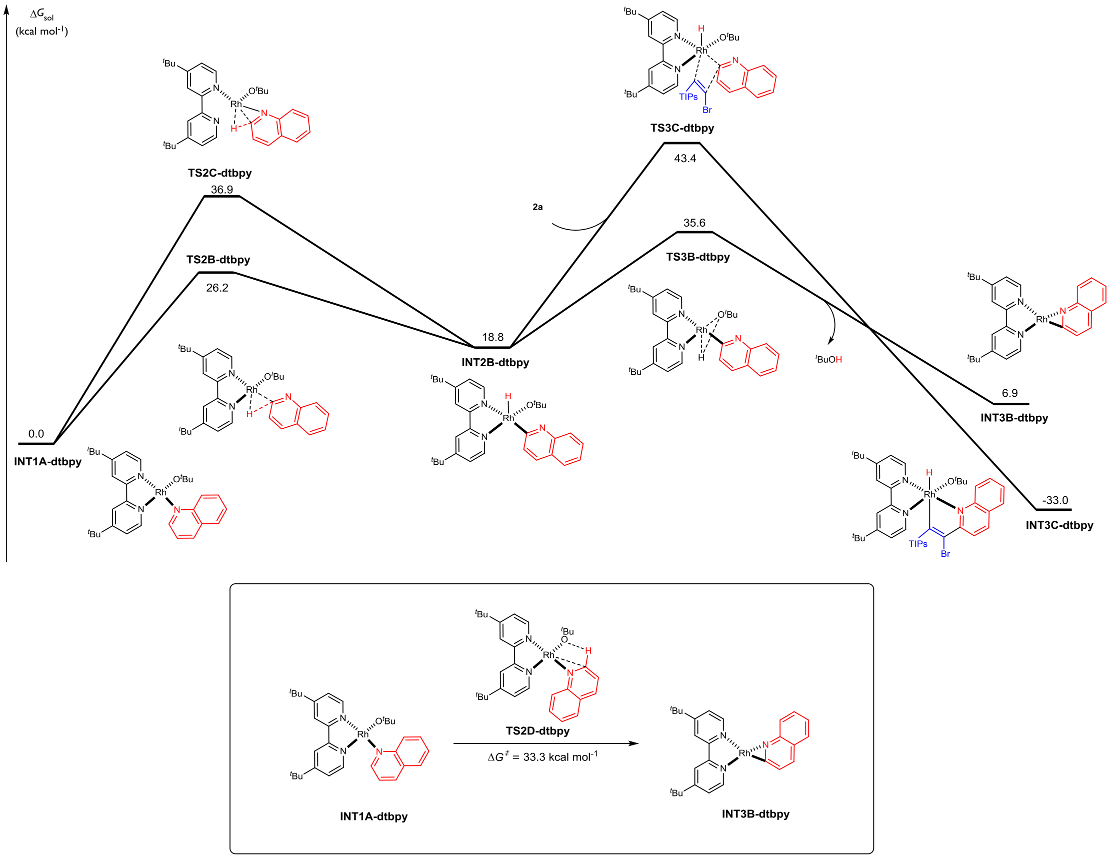

Supplementary Fig. 3. DFT-computed free energies of the competitive pathway for C-H bond activation using dtbpy as ligand.

For the ligand IMes, the computational results were presented in **Supplementary Figure 4**. The C-H bonds at 2- and 8-positions of quinoline undergo direct oxidative addition to the Rh(I) center through transition states **TS2B-IMes** and **TS2E-IMes** with free energy of 28.7 and 25.5 kcal mol^{-1}. The following reductive elimination of **INT2B-IMes** and **INT2E-IMes** have activation energy barriers of 20.0 and 19.5 kcal mol^{-1}, affording the total activation barriers of 27.6 and 30.9 kcal mol^{-1}, respectively. These calculated energy results are much higher than that the C-Br bond oxidative addition through transition state **TS2A-IMes**.

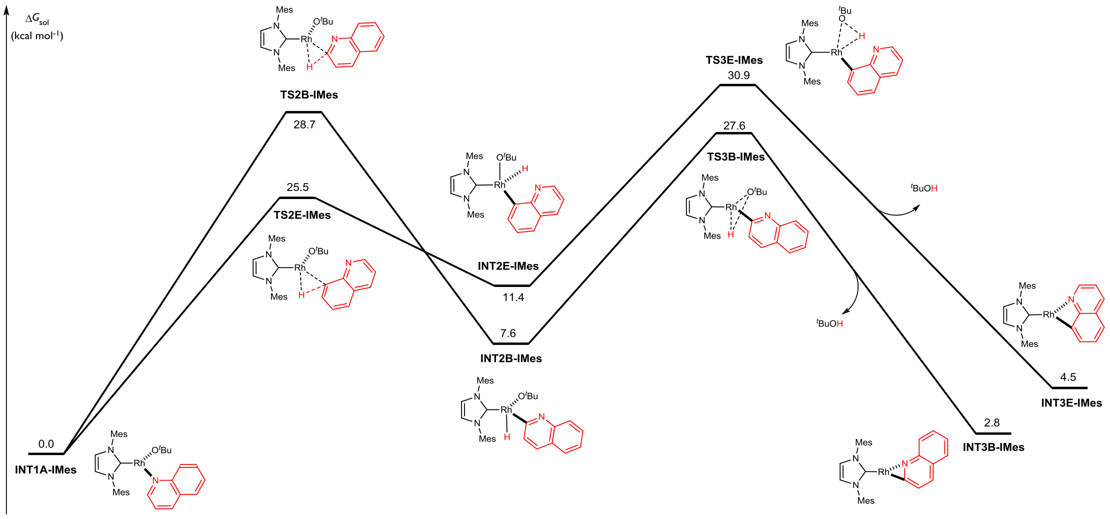

Supplementary Fig. 4. DFT-computed free energies of the competitive pathway for C-H bond activation using IMes as ligand.

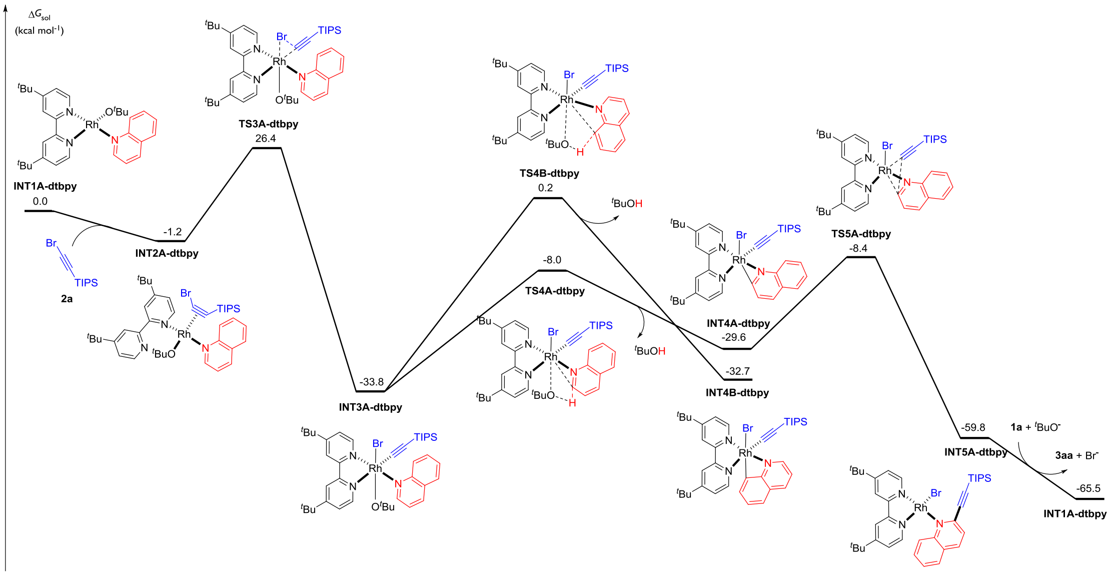

Supplementary Fig. 5. DFT-computed energy profile for the Rh-catalyzed C-H alkynylation with ligand dtbpy.

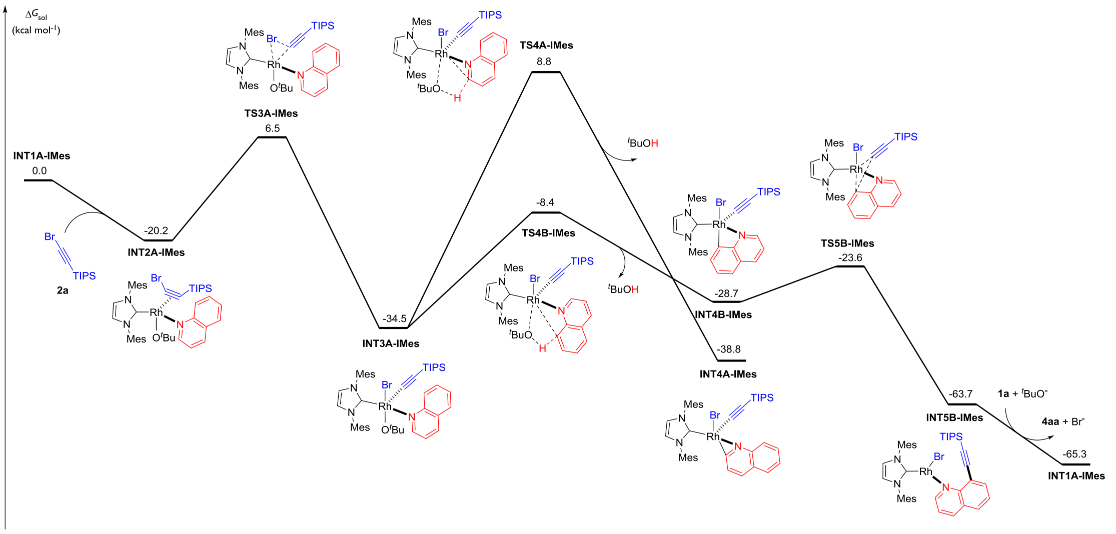

Supplementary Fig. 6. DFT-computed energy profile for the Rh-catalyzed C-H alkynylation with ligand IMes.

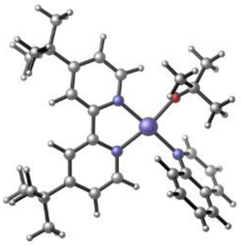

INT1A-dtbpy

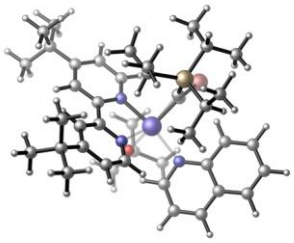

INT2A-dtbpy

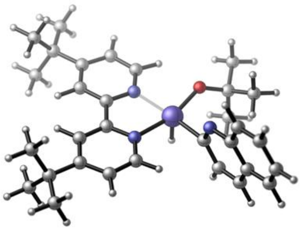

INT2B-dtbpy

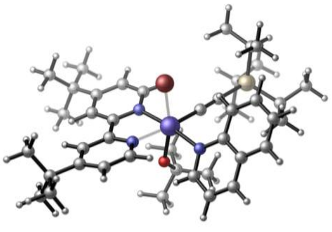

INT3A-dtbpy

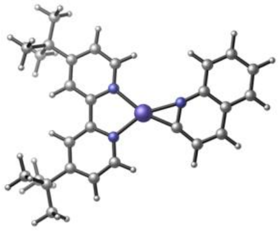

INT3B-dtbpy

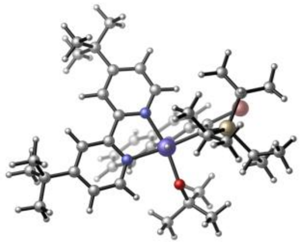

INT3C-dtbpy

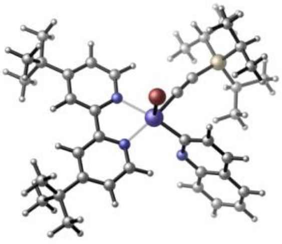

INT4A-dtbpy

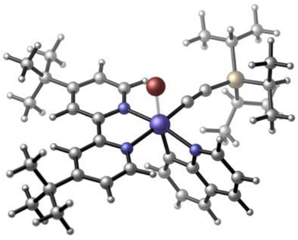

INT4B-dtbpy

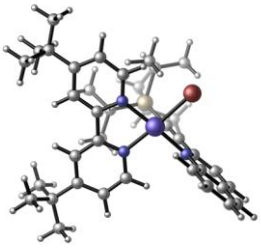

INT5A-dtbpy

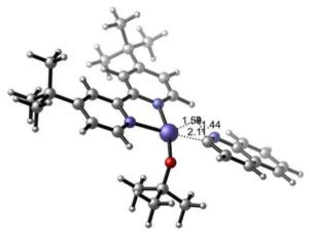

TS2B-dtbpy

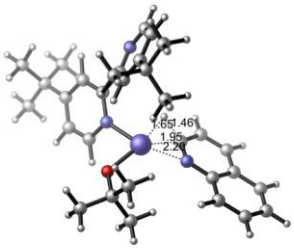

TS2C-dtbpy

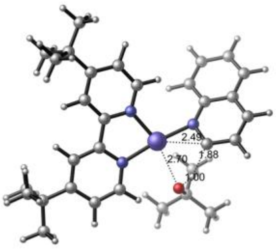

TS2D-dtbpy

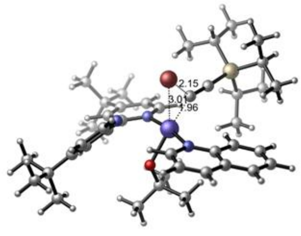

TS3A-dtbpy

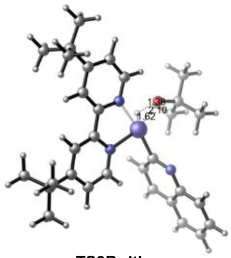

TS3B-dtbpy

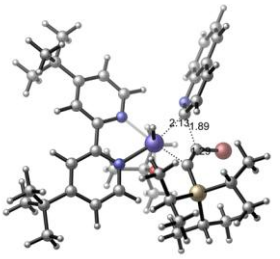

TS3C-dtbpy

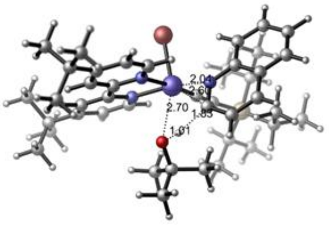

TS4A-dtbpy

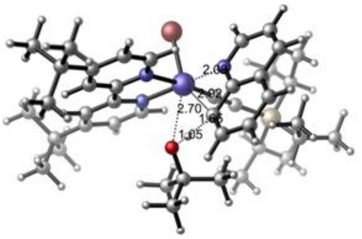

TS4B-dtbpy

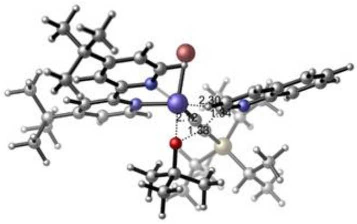

TS4C-dtbpy

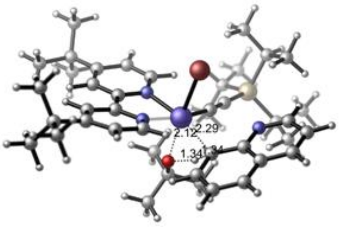

TS4D-dtbpy

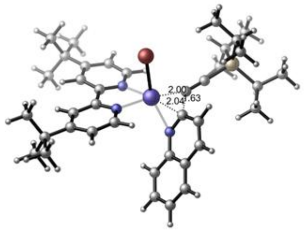

TS5A-dtbpy

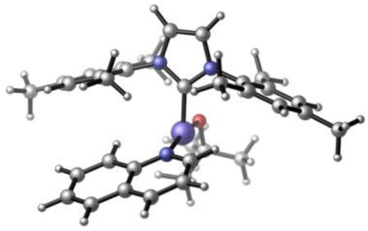

INT1A-IMes

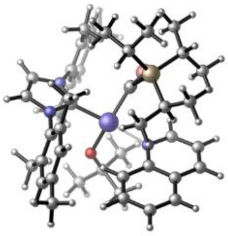

INT2A-IMes

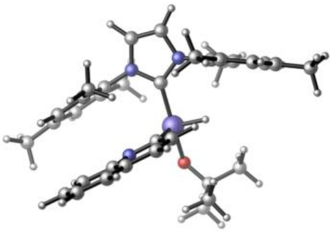

INT2B-IMes

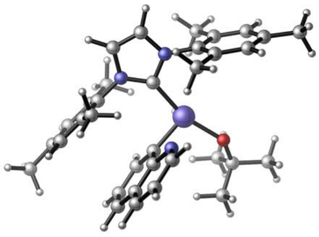

INT2E-IMes

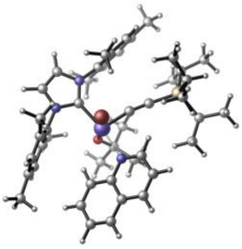

INT3A-IMes

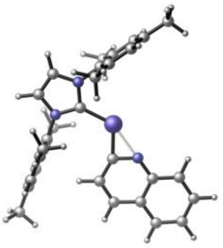

INT3B-IMes

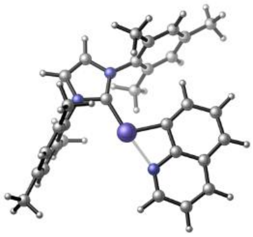

INT3E-IMes

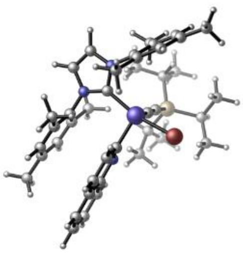

INT4A-IMes

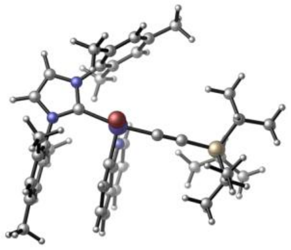

INT4B-IMes

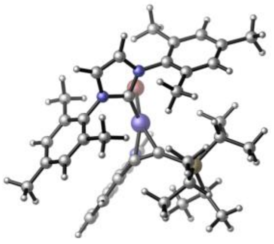

INT5B-IMes

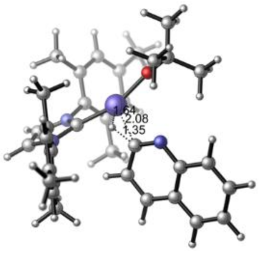

TS2B-IMes

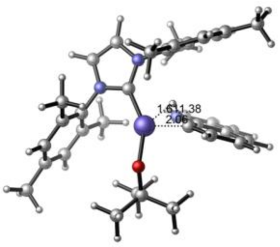

TS2E-IMes

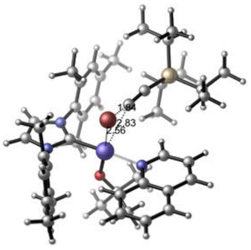

TS3A-IMes

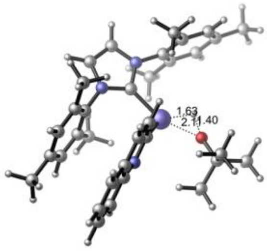

TS3B-IMes

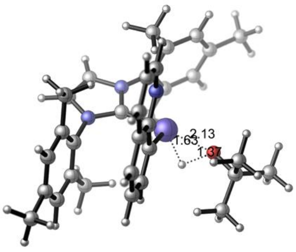

TS3E-IMes

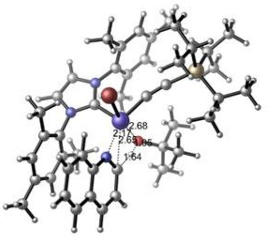

TS4A-IMes

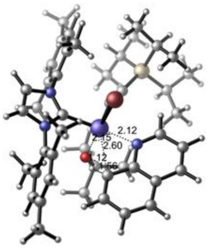

TS4B-IMes

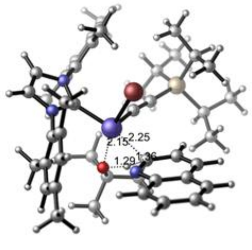

TS4C-IMes

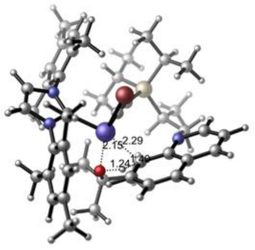

TS4D-IMes

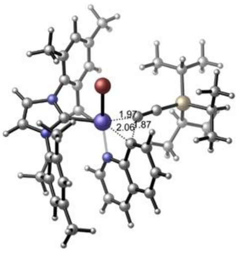

TS5B-IMes

Supplementary Fig. 7. The calculated optimized structures of the key intermediates and transition states. Distances are in Å.

2.5 Copies of ^{1}H NMR, ^{13}C NMR and ^{19}F NMR Spectras

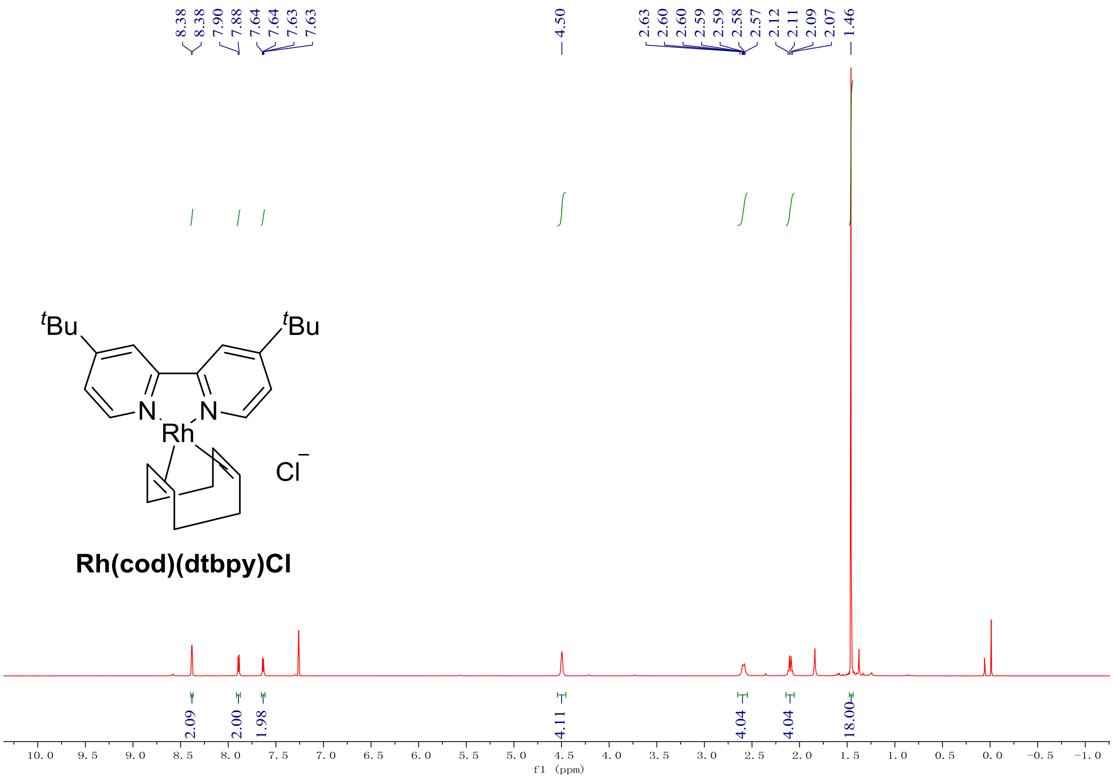

Supplementary Fig. 8. ^{1}H NMR spectra (500 MHz, $CDCl_3$, 25 ^{o}C) of **Rh(cod)(dtbpy)Cl**

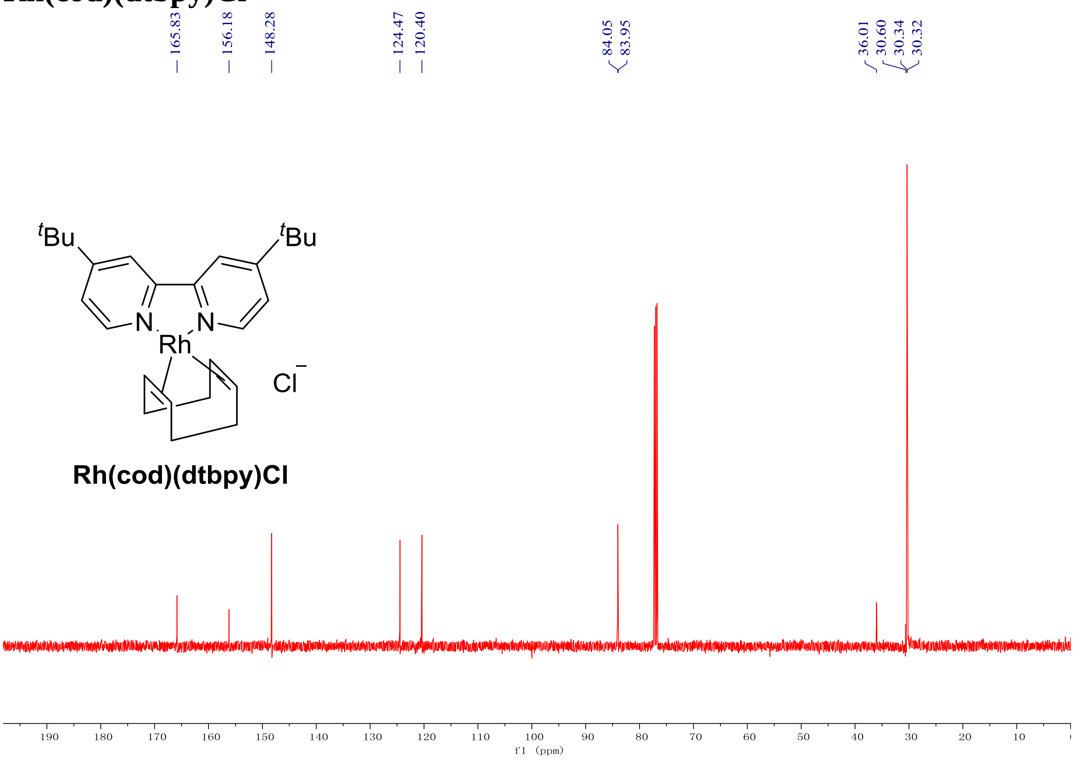

Supplementary Fig. 9. ^{13}C NMR spectra (126 MHz, $CDCl_3$, 25 ^{o}C) of **Rh(cod)(dtbpy)Cl**

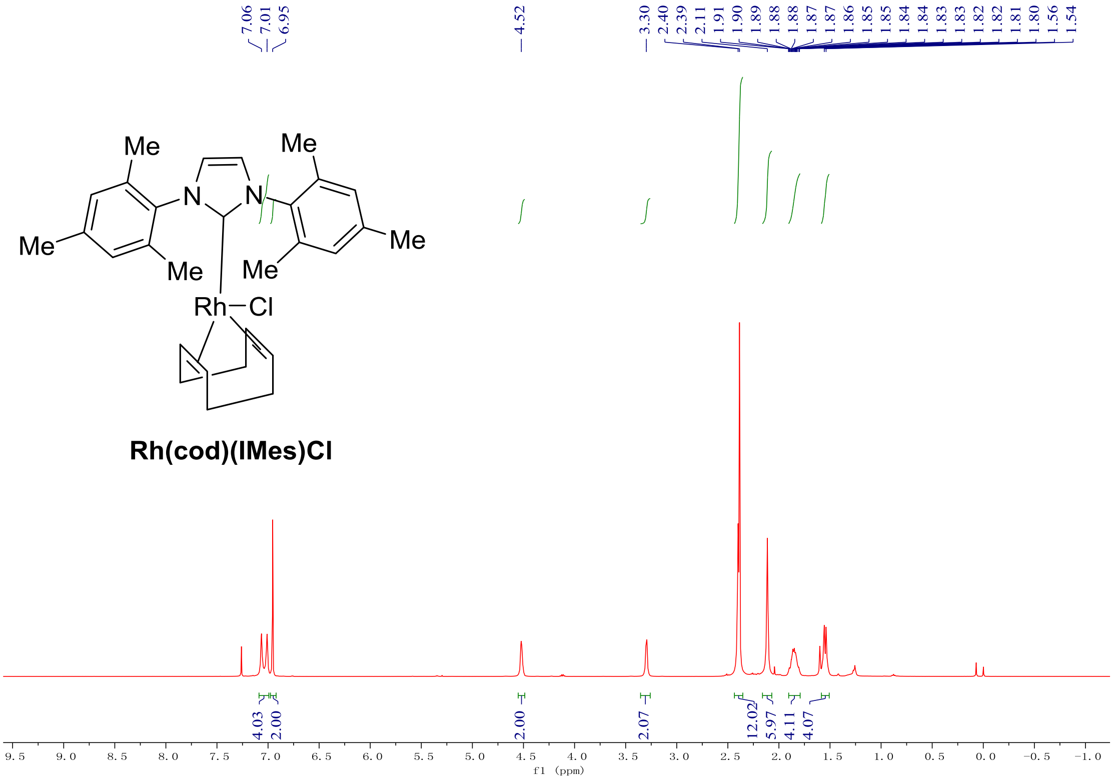

Supplementary Fig. 10. ^{1}H NMR spectra (500 MHz, $CDCl_3$, 25 °C) of **Rh(cod)(IMes)Cl**

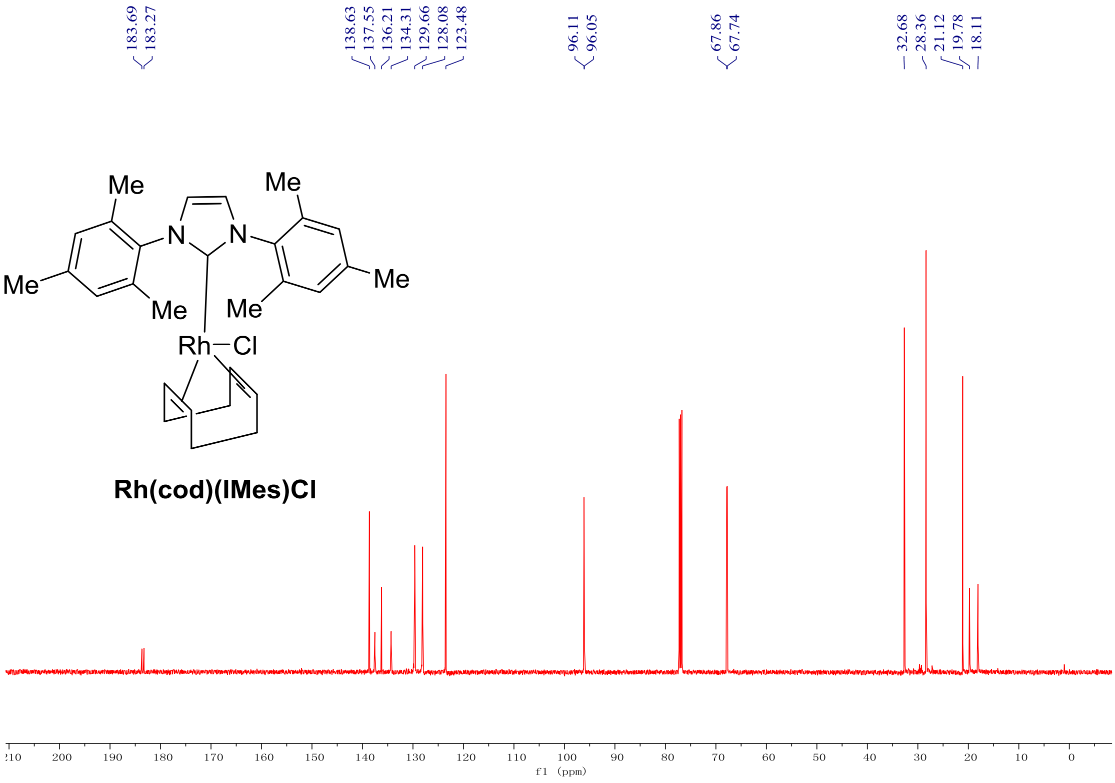

Supplementary Fig. 11. ^{13}C NMR spectra (126 MHz, $CDCl_3$, 25 °C) of **Rh(cod)(IMes)Cl**

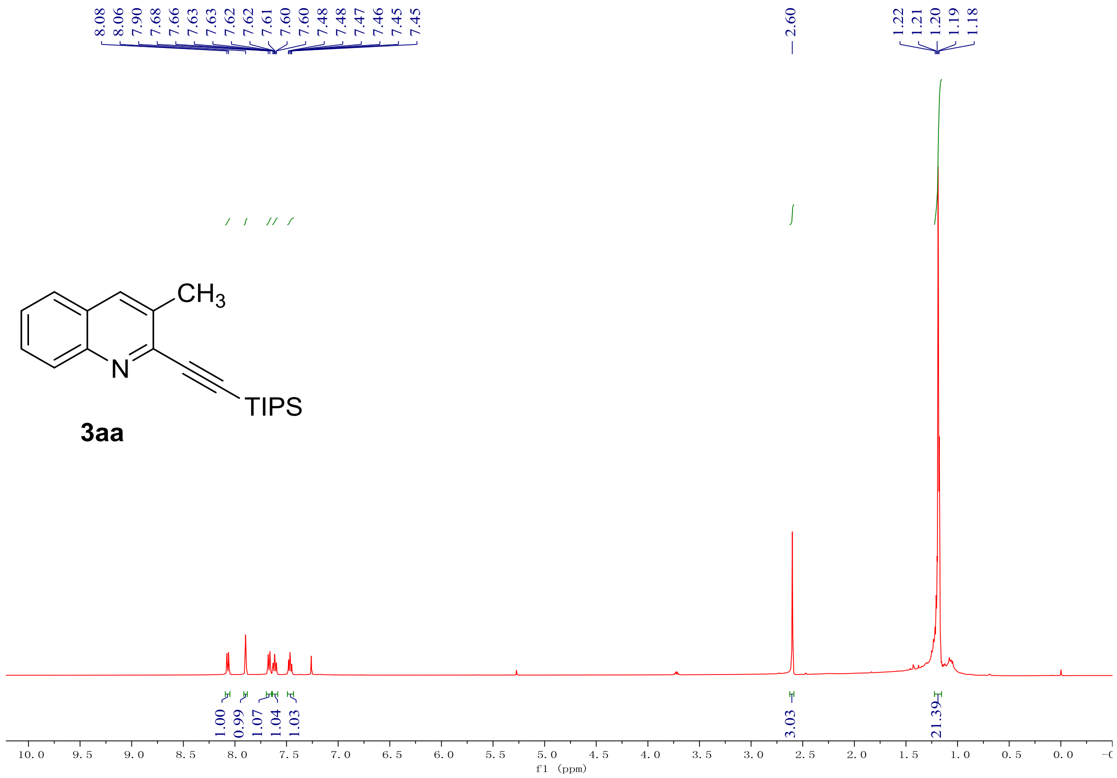

Supplementary Fig. 12. ^{1}H NMR spectra (500 MHz, $CDCl_3$, 25 °C) of **3aa**

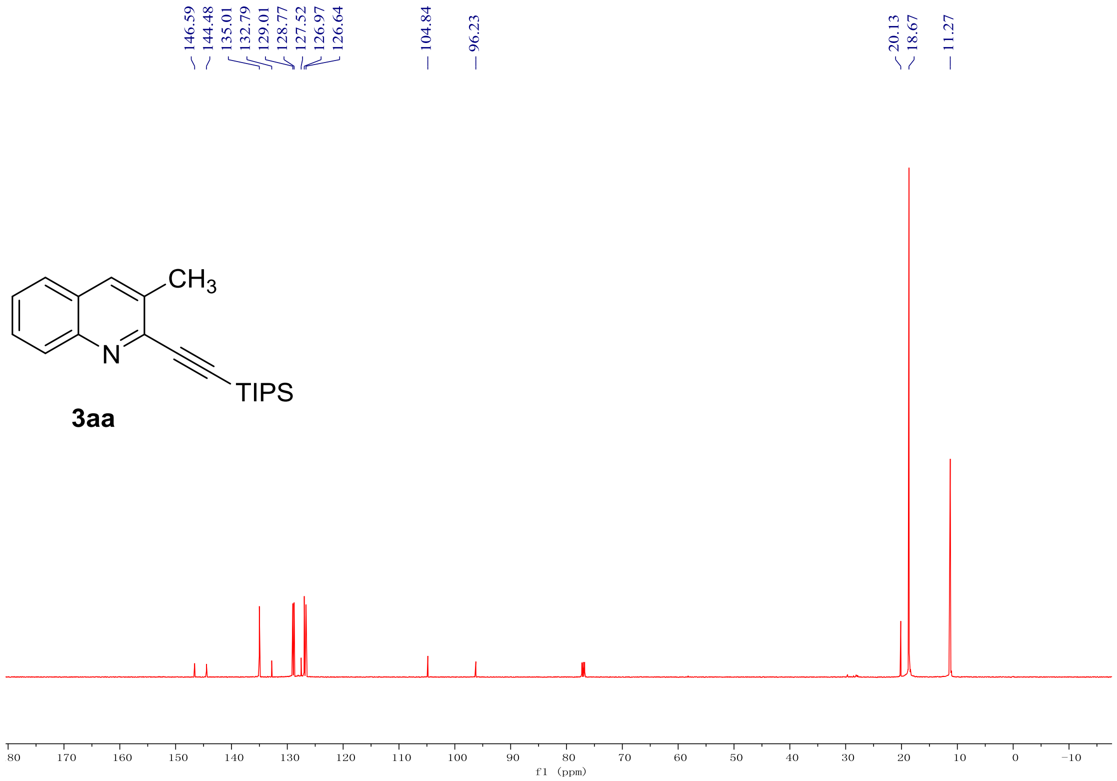

Supplementary Fig. 13. ^{13}C NMR spectra (126 MHz, $CDCl_3$, 25 °C) of **3aa**

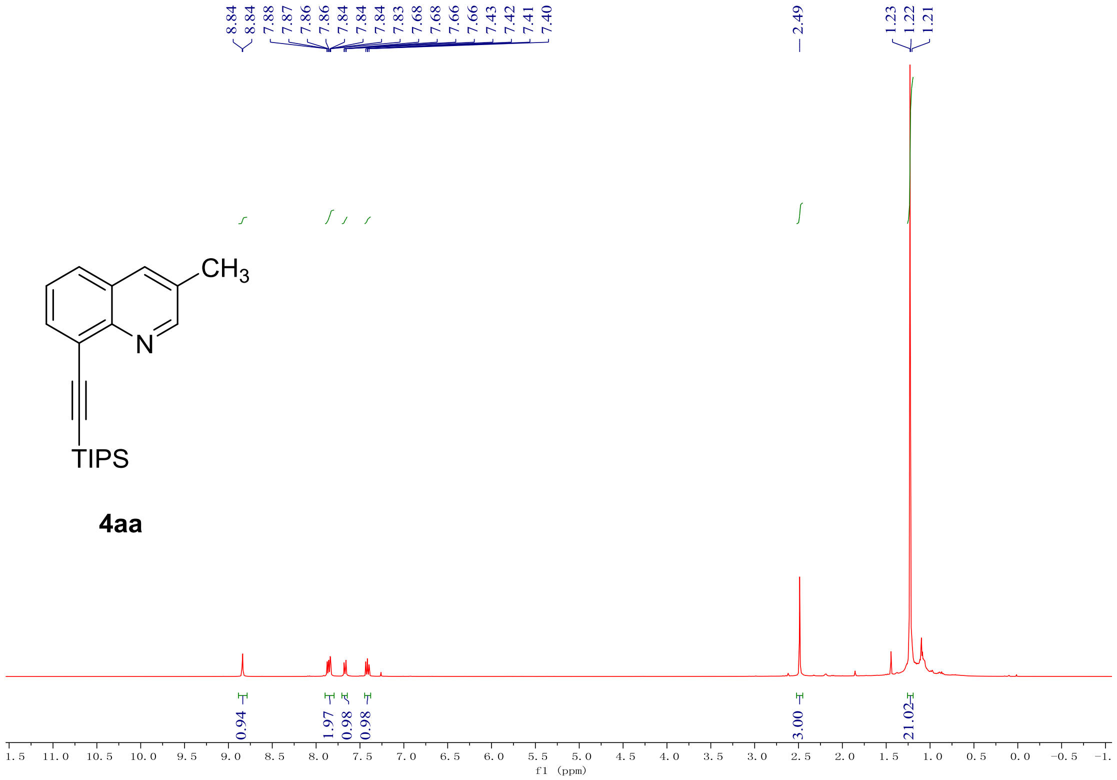

Supplementary Fig. 14. ^{1}H NMR spectra (400 MHz, $CDCl_3$, 25 ^{o}C) of **4aa**

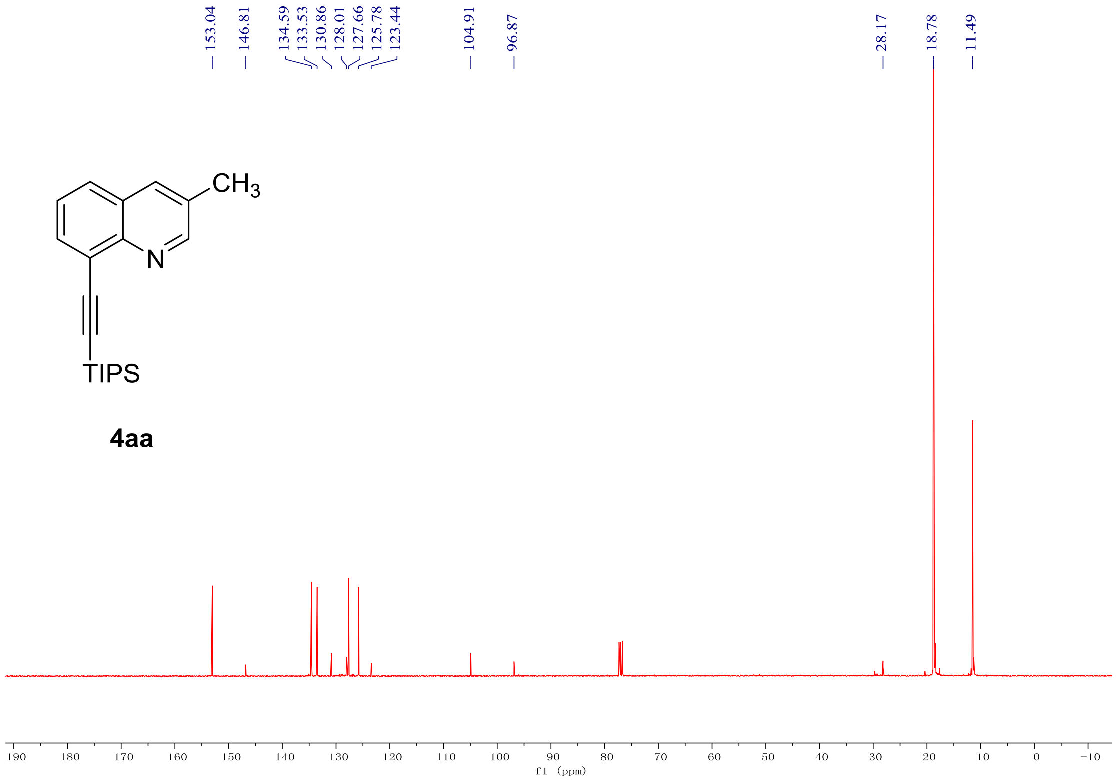

Supplementary Fig. 15. ^{13}C NMR spectra (101 MHz, $CDCl_3$, 25 ^{o}C) of **4aa**

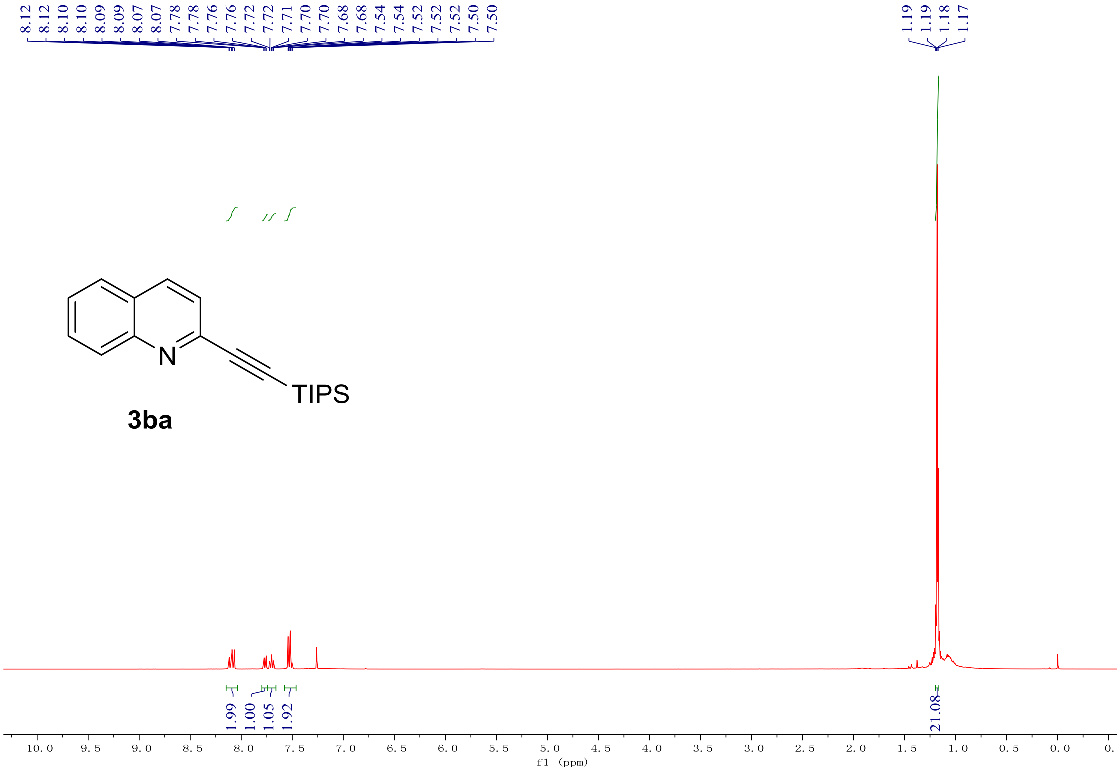

Supplementary Fig. 16. ^{1}H NMR spectra (400 MHz, $CDCl_3$, 25 °C) of **3ba**

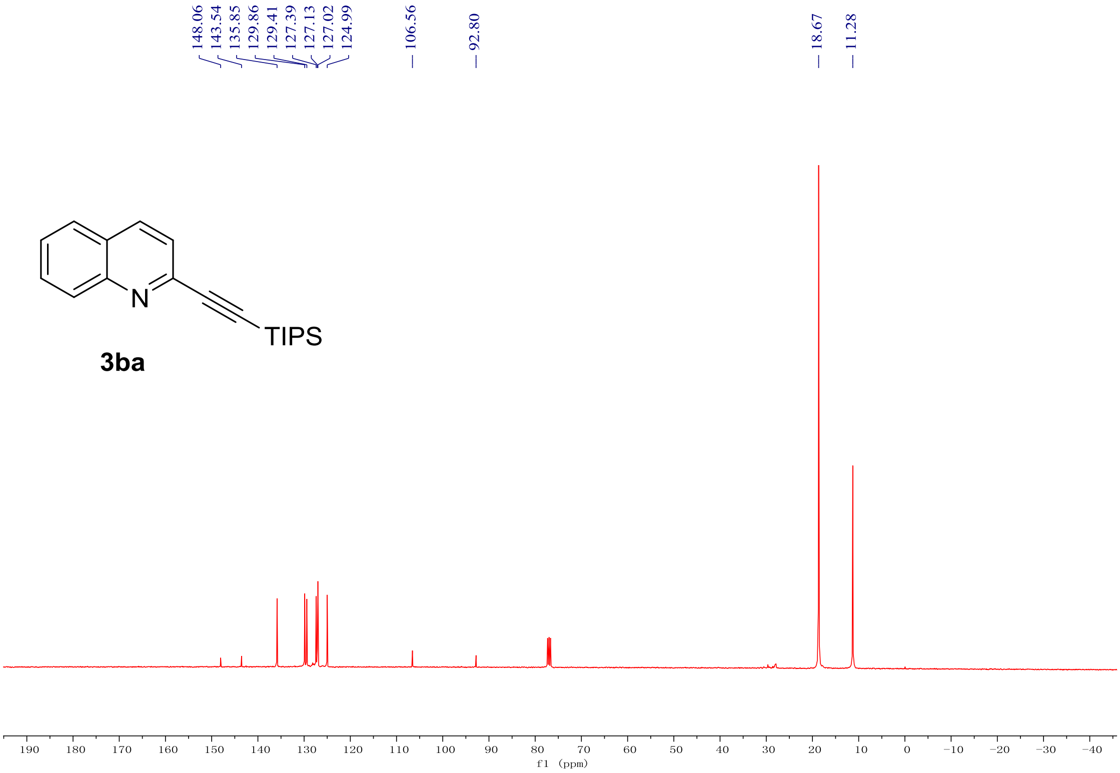

Supplementary Fig. 17. ^{13}C NMR spectra (101 MHz, $CDCl_3$, 25 °C) of **3ba**

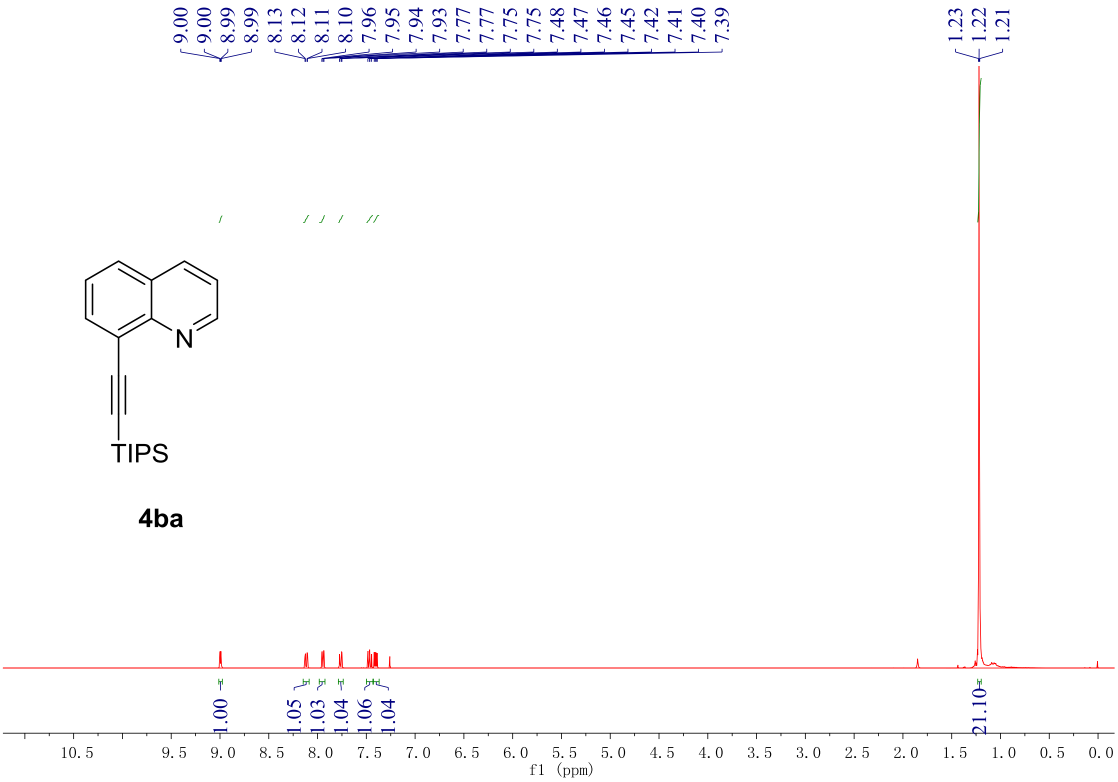

Supplementary Fig. 18. ^{1}H NMR spectra (400 MHz, $CDCl_3$, 25 ^{o}C) of **4ba**

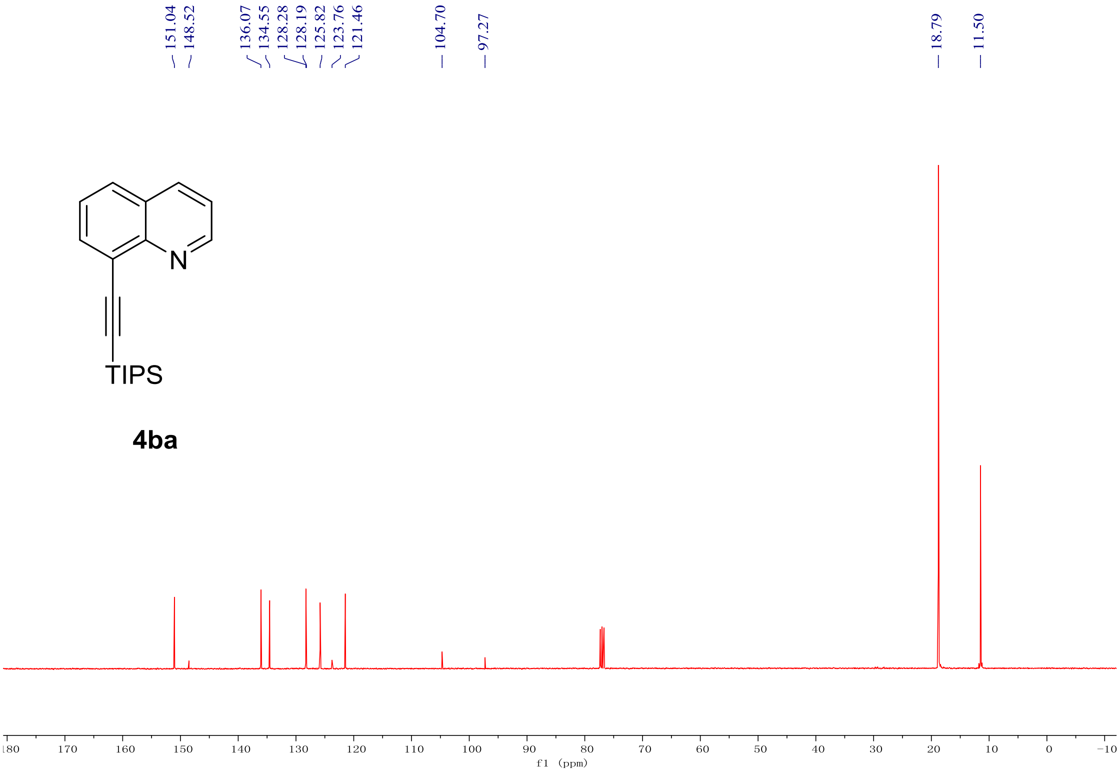

Supplementary Fig. 19. ^{13}C NMR spectra (101 MHz, $CDCl_3$, 25 ^{o}C) of **4ba**

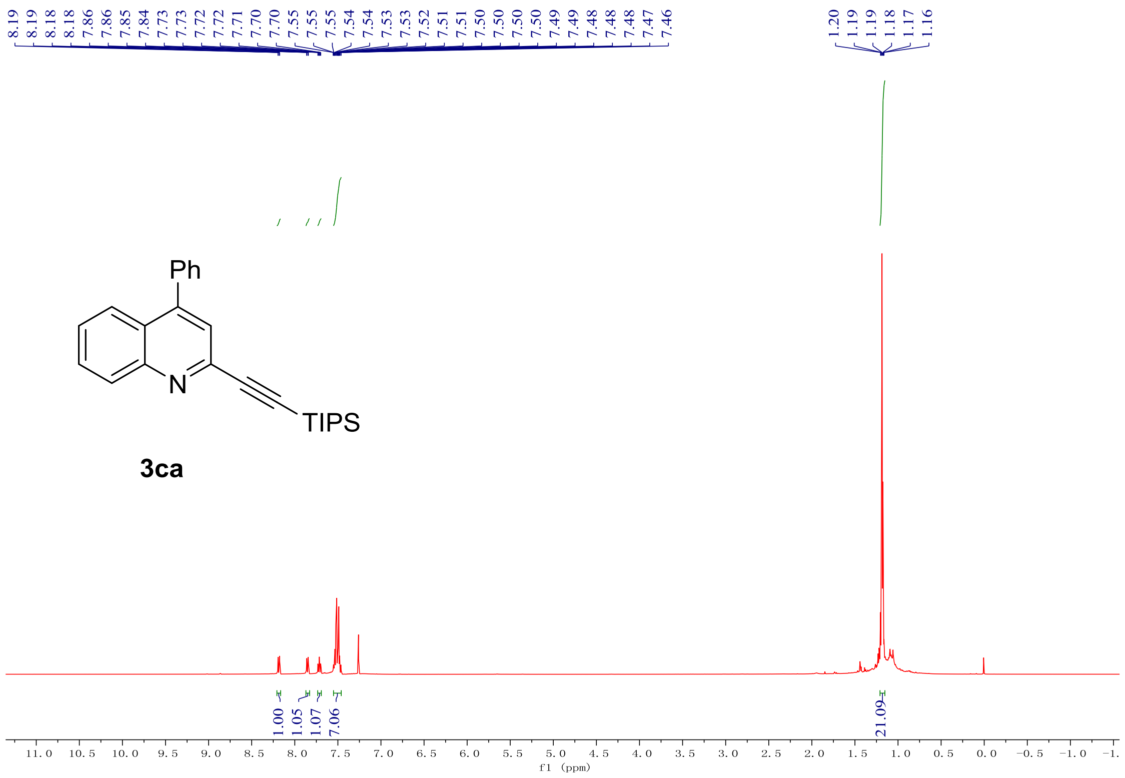

Supplementary Fig. 20. ^{1}H NMR spectra (500 MHz, $CDCl_3$, 25 °C) of **3ca**

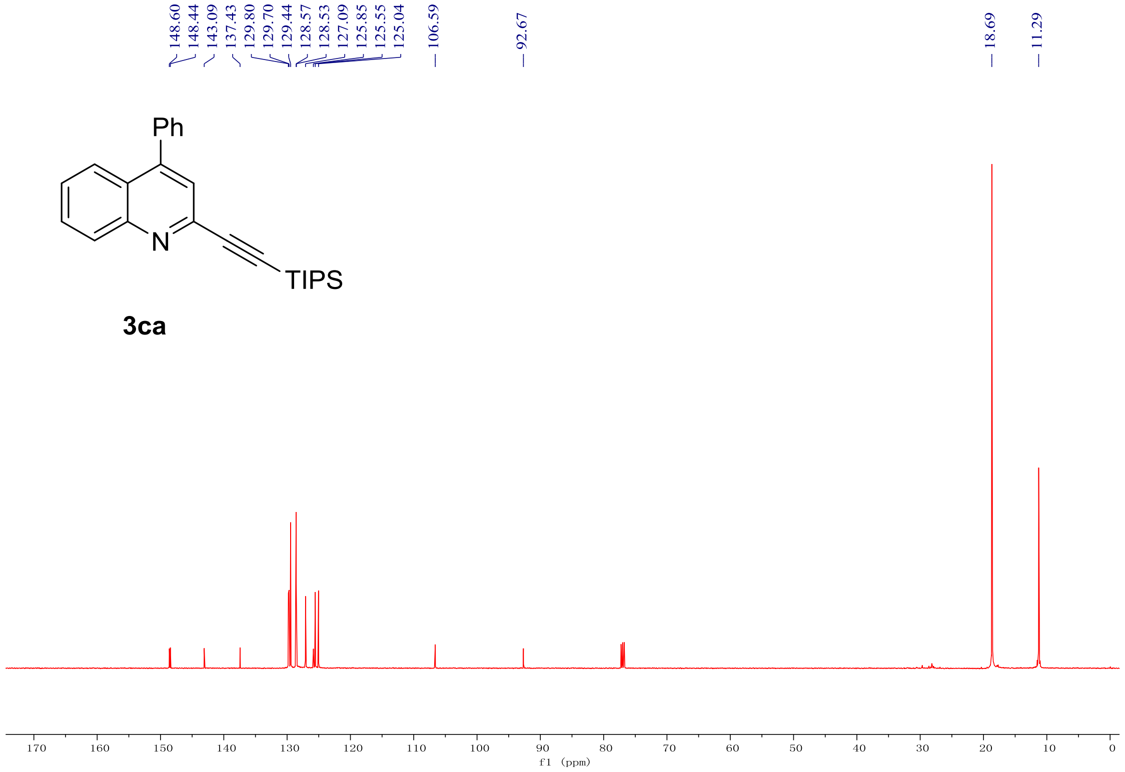

Supplementary Fig. 21. ^{13}C NMR spectra (126 MHz, $CDCl_3$, 25 °C) of **3ca**

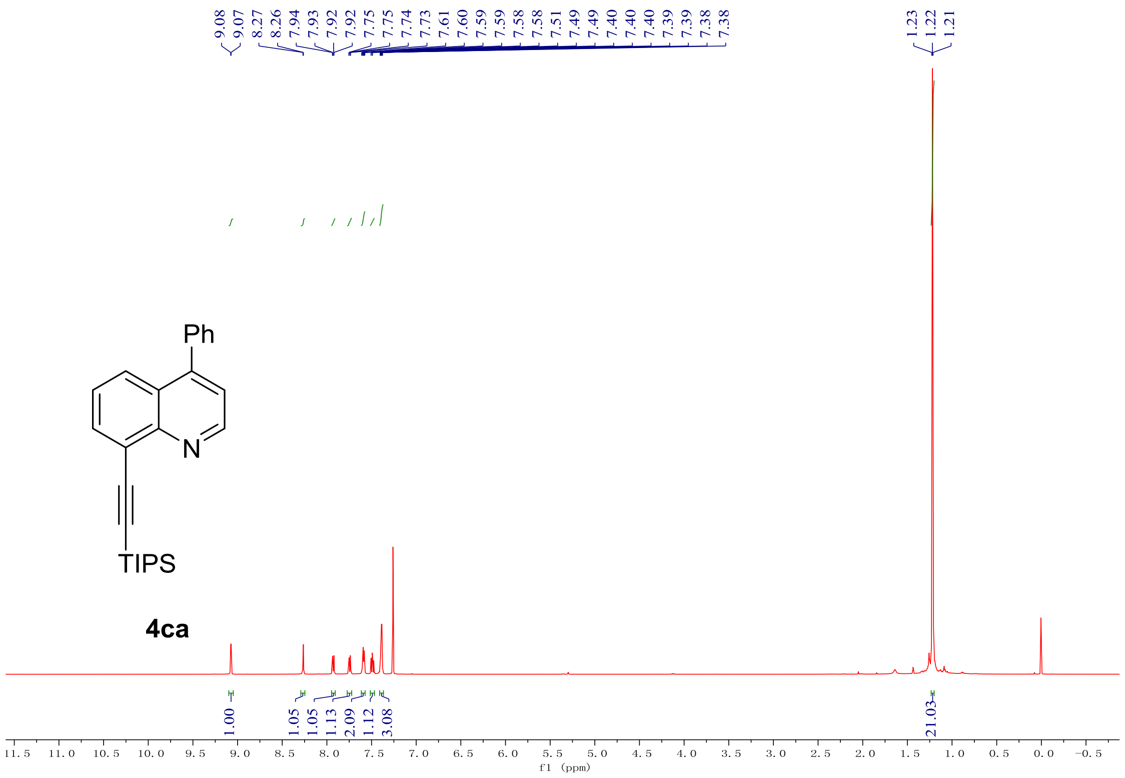

Supplementary Fig. 22. ^{1}H NMR spectra (500 MHz, $CDCl_3$, 25 ^{o}C) of **4ca**

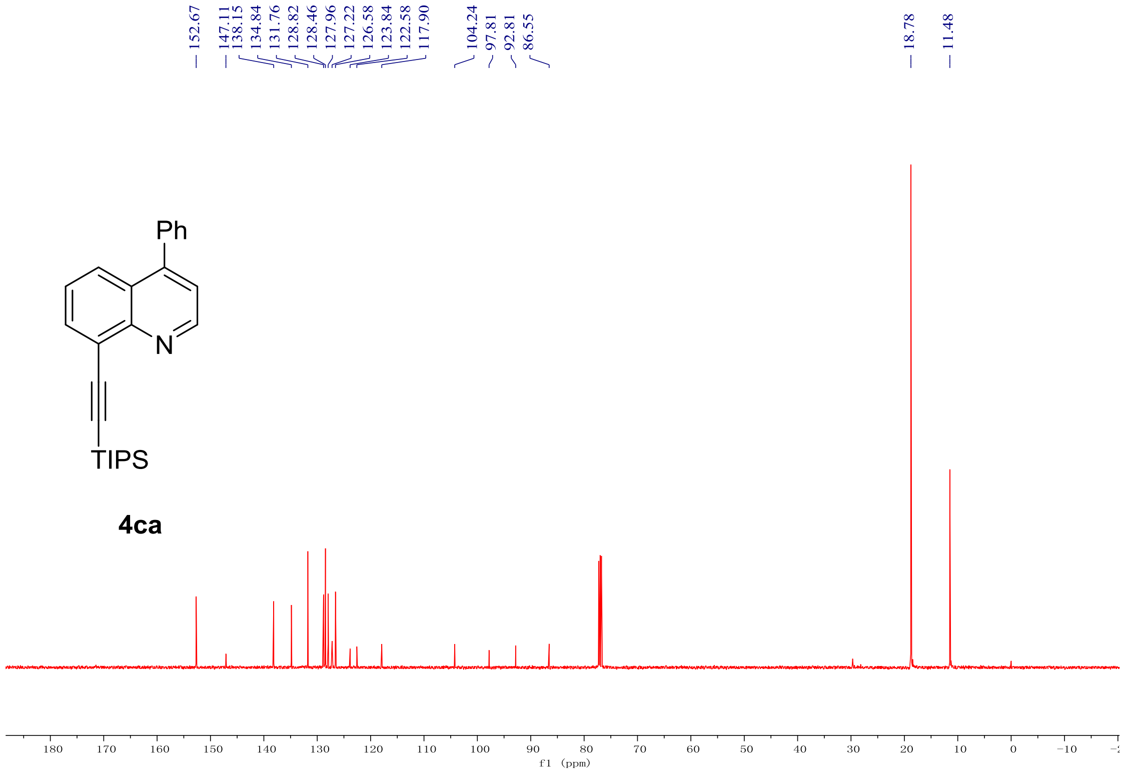

Supplementary Fig. 23. ^{13}C NMR spectra (126 MHz, $CDCl_3$, 25 ^{o}C) of **4ca**

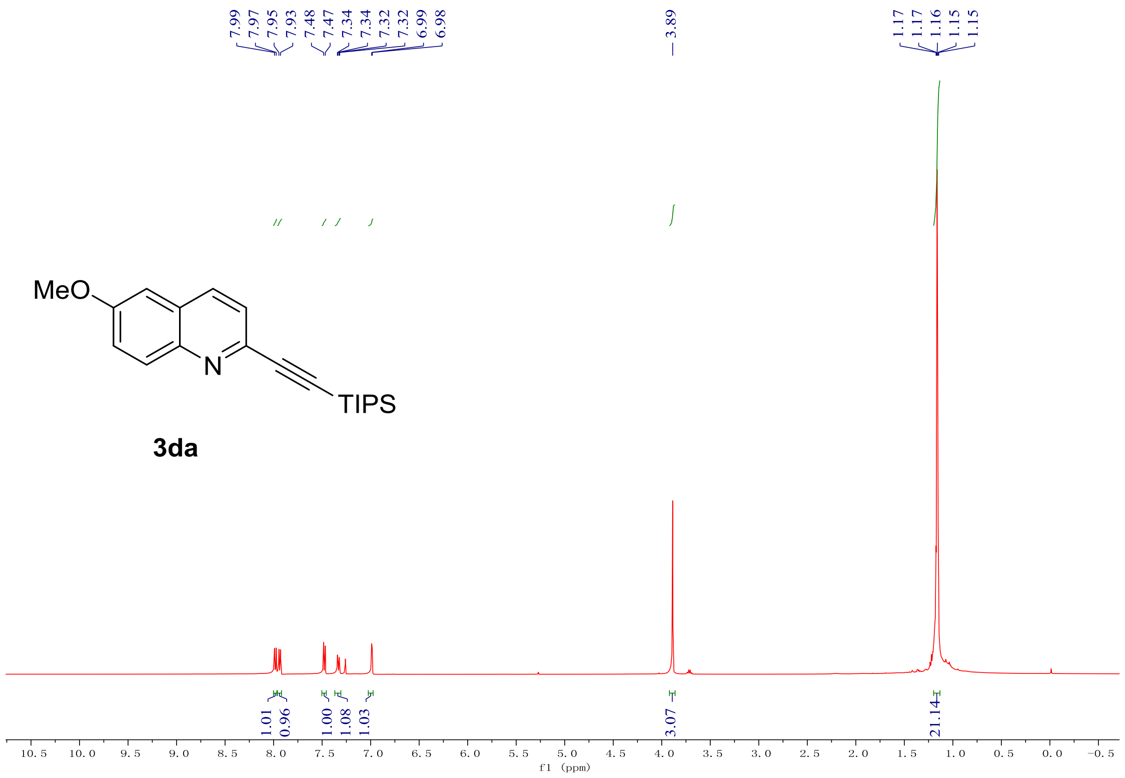

Supplementary Fig. 24. ^{1}H NMR spectra (500 MHz, $CDCl_3$, 25 °C) of **3da**

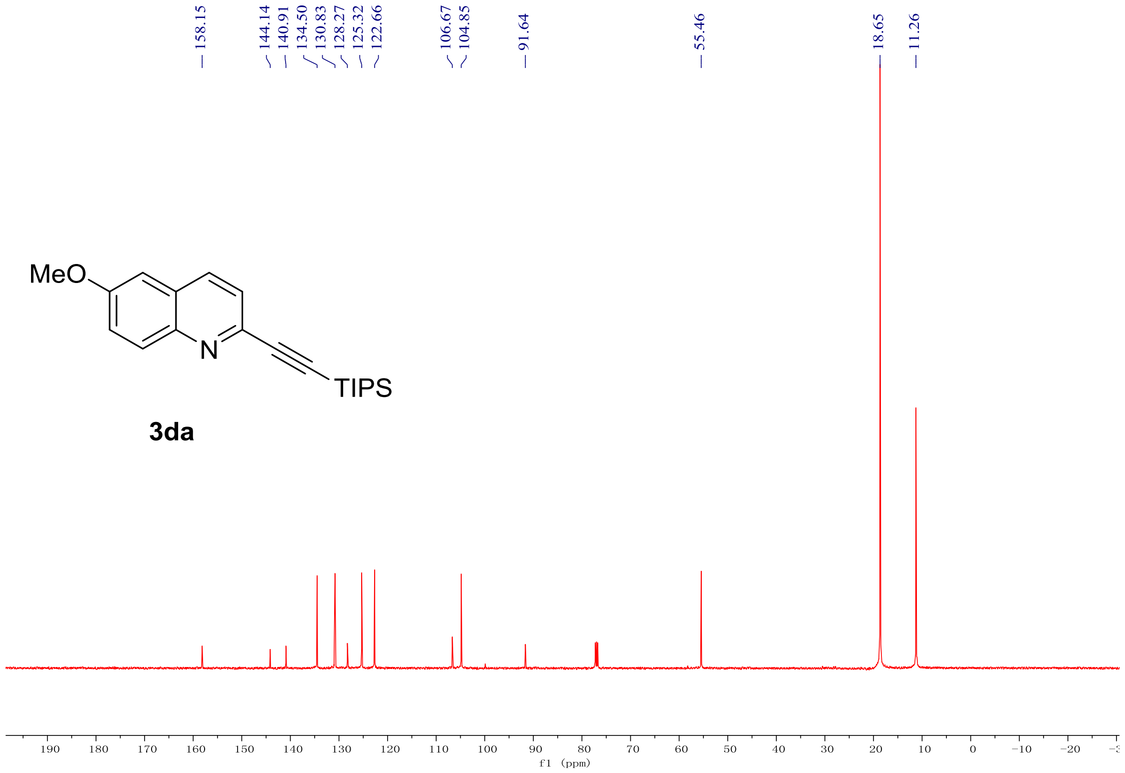

Supplementary Fig. 25. ^{13}C NMR spectra (126 MHz, $CDCl_3$, 25 °C) of **3da**

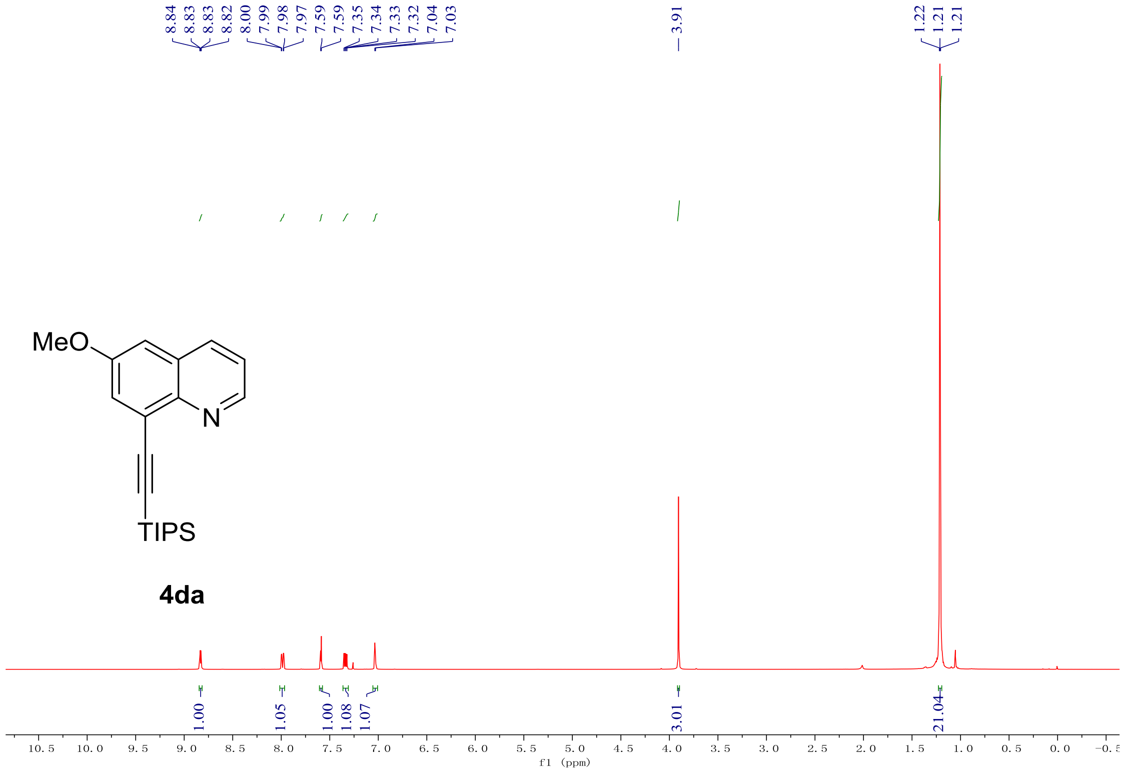

Supplementary Fig. 26. ^{1}H NMR spectra (400 MHz, $CDCl_3$, 25 ^{o}C) of **4da**

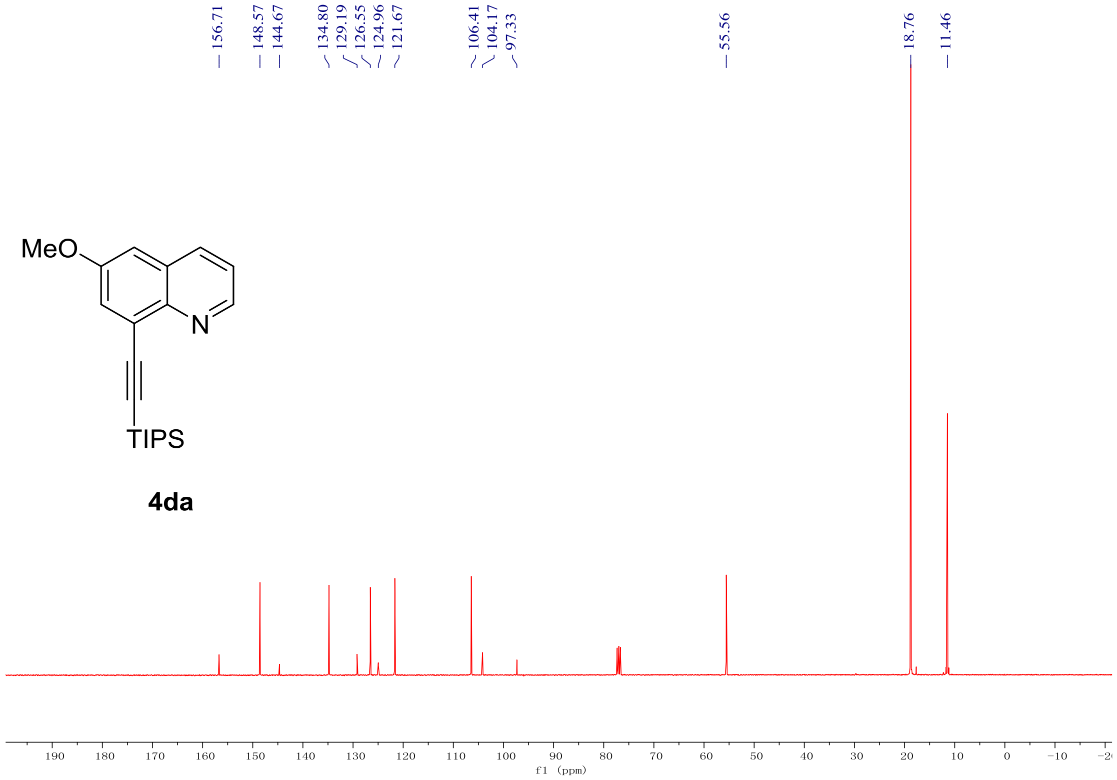

Supplementary Fig. 27. ^{13}C NMR spectra (101 MHz, $CDCl_3$, 25 ^{o}C)of **4da**

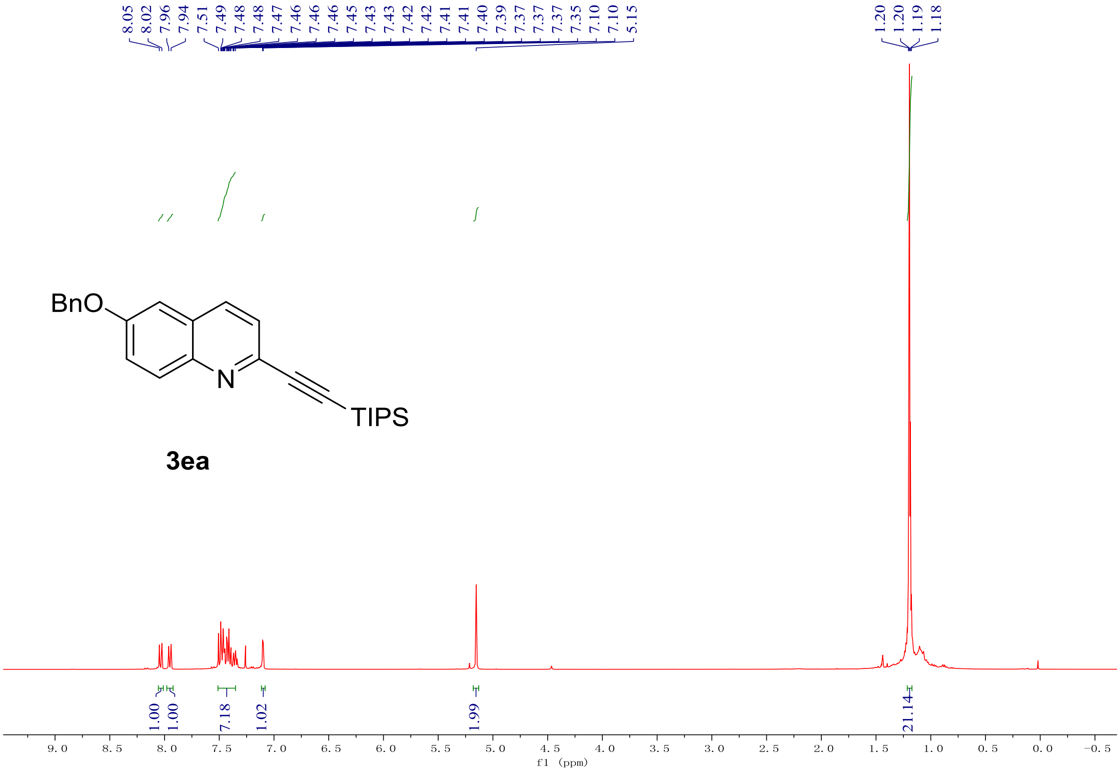

Supplementary Fig. 28. ^{1}H NMR spectra (400 MHz, $CDCl_3$, 25 °C) of **3ea**

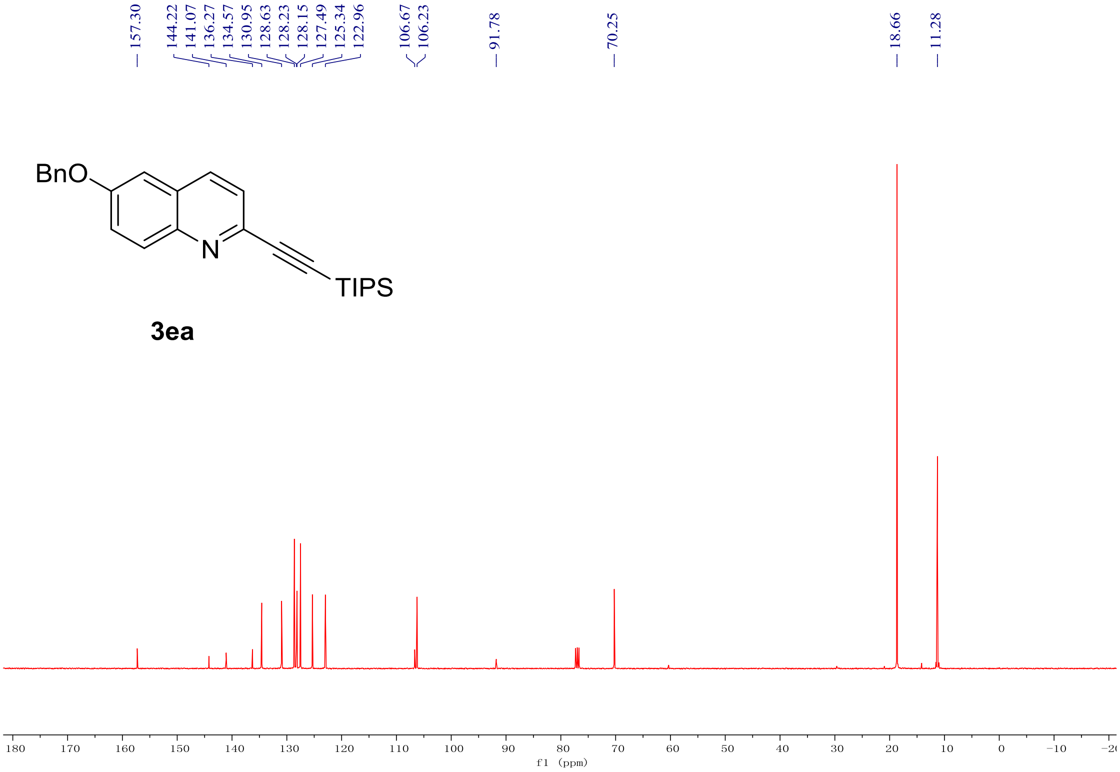

Supplementary Fig. 29. ^{13}C NMR spectra (101 MHz, $CDCl_3$, 25 °C) of **3ea**

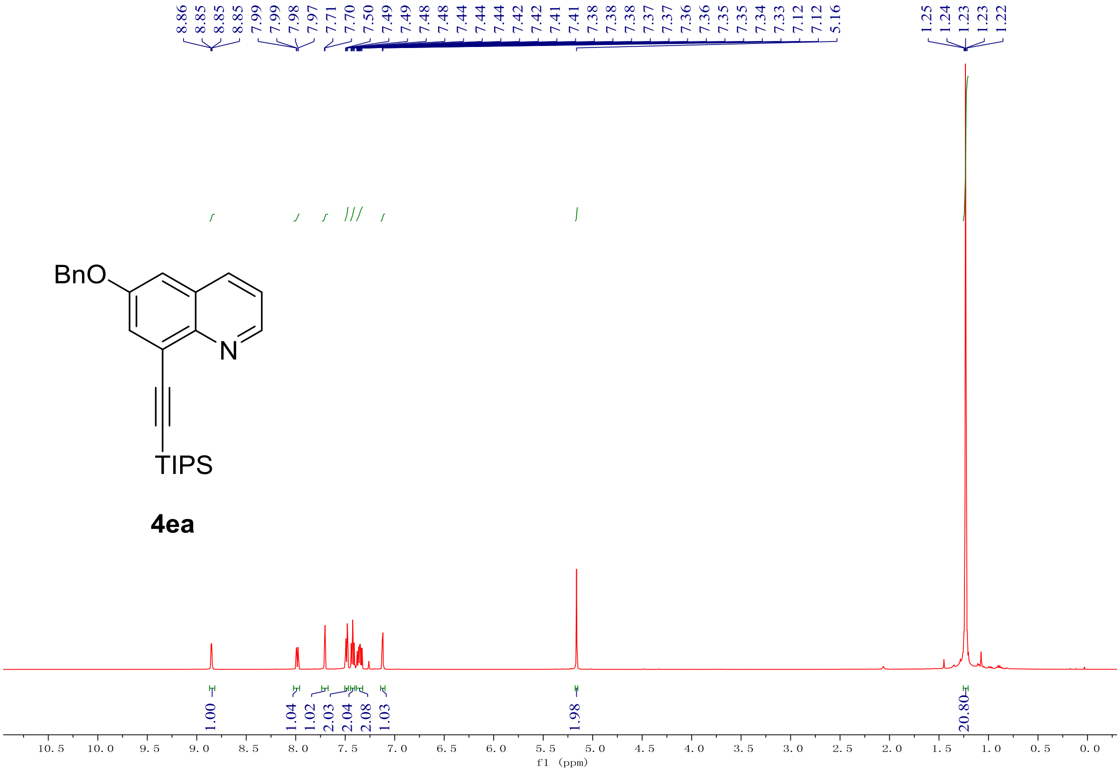

Supplementary Fig. 30. ^{1}H NMR spectra (500 MHz, $CDCl_3$, 25 °C) of **4ea**

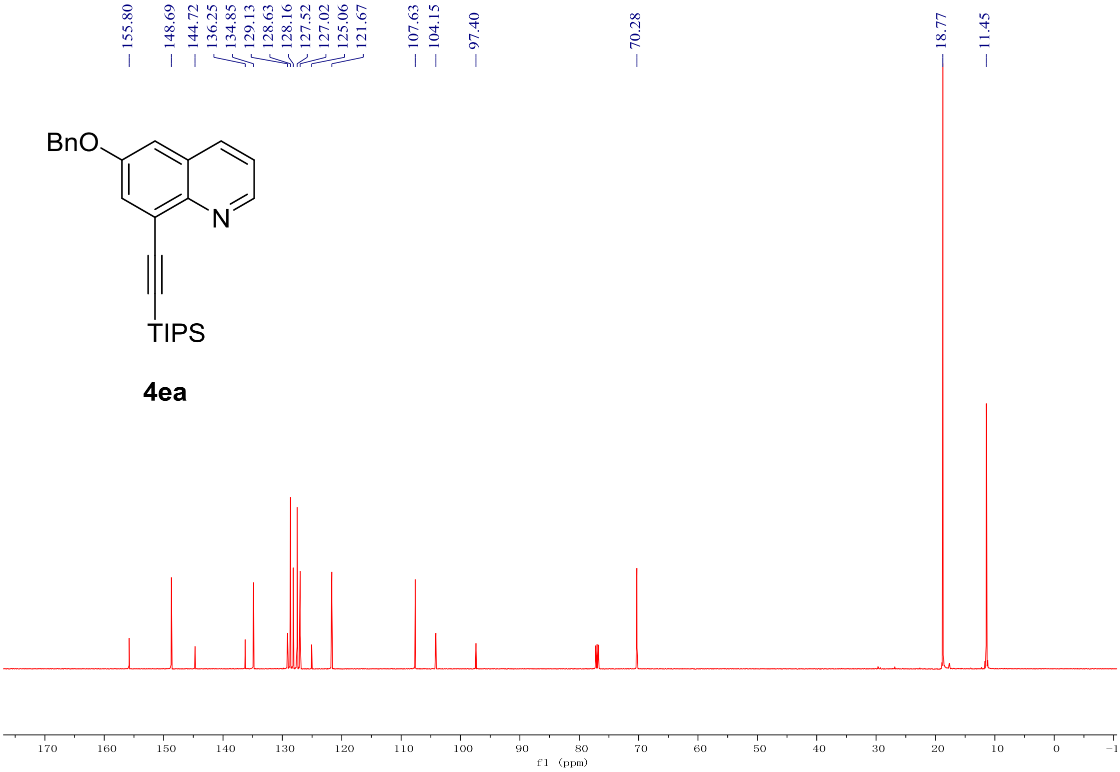

Supplementary Fig. 31. ^{13}C NMR spectra (126 MHz, $CDCl_3$, 25 °C) of **4ea**

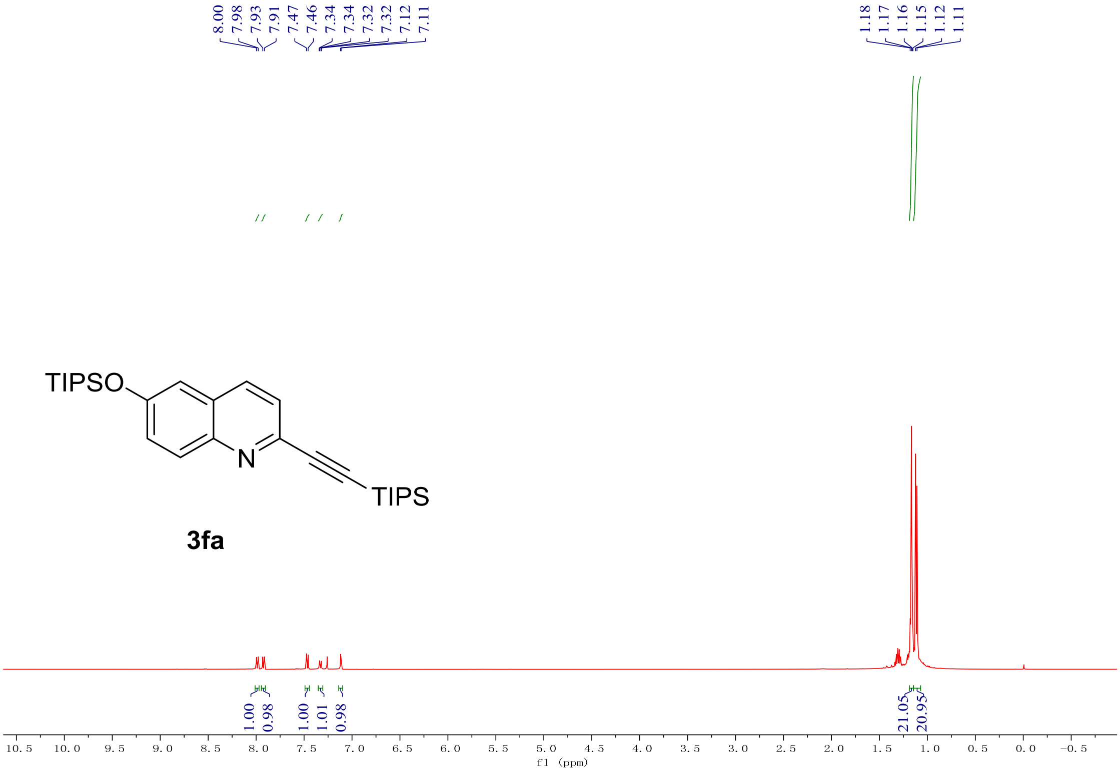

Supplementary Fig. 32. ^{1}H NMR spectra (500 MHz, $CDCl_3$, 25 ^oC) of **3fa**

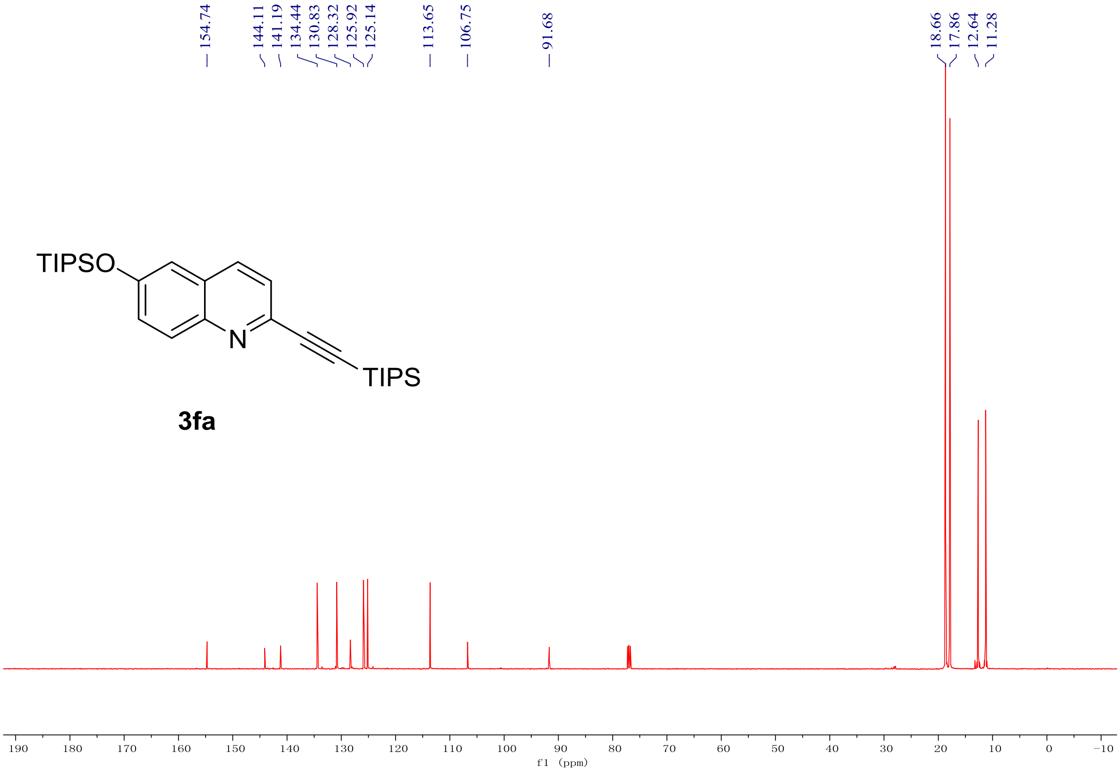

Supplementary Fig. 33. ^{13}C NMR spectra (126 MHz, $CDCl_3$, 25 ^oC) of **3fa**

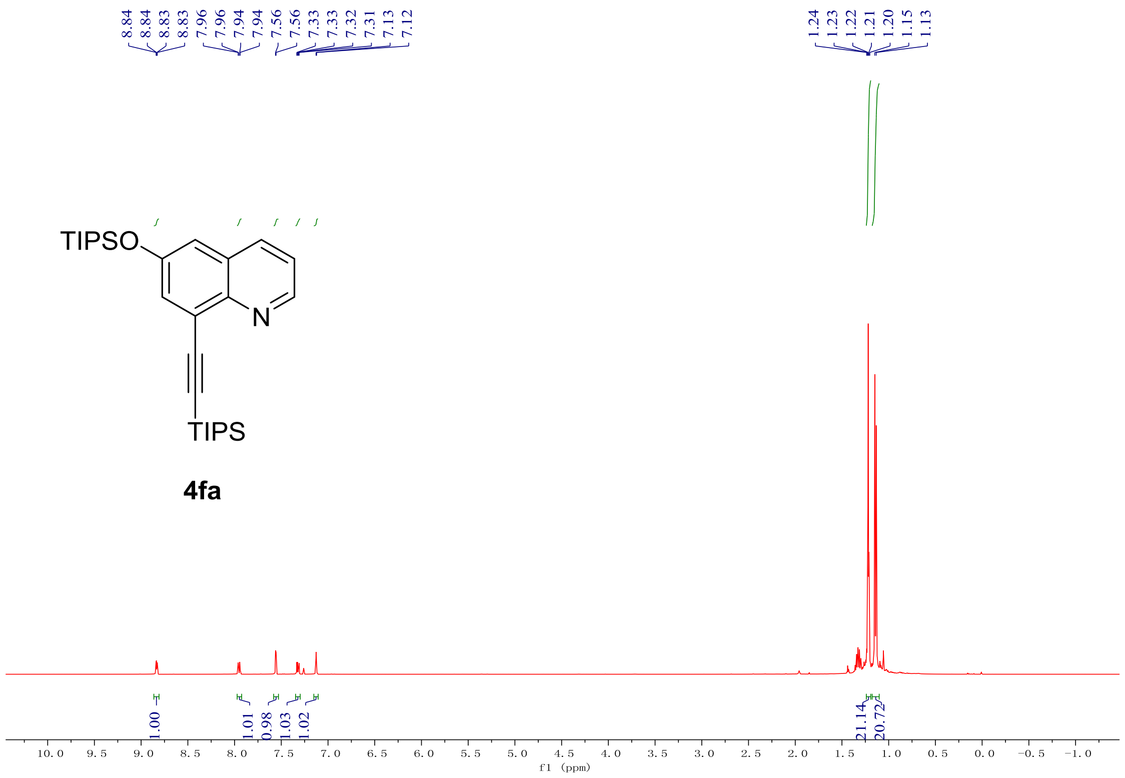

Supplementary Fig. 34. ^{1}H NMR spectra (500 MHz, $CDCl_3$, 25 ^{o}C) of **4fa**

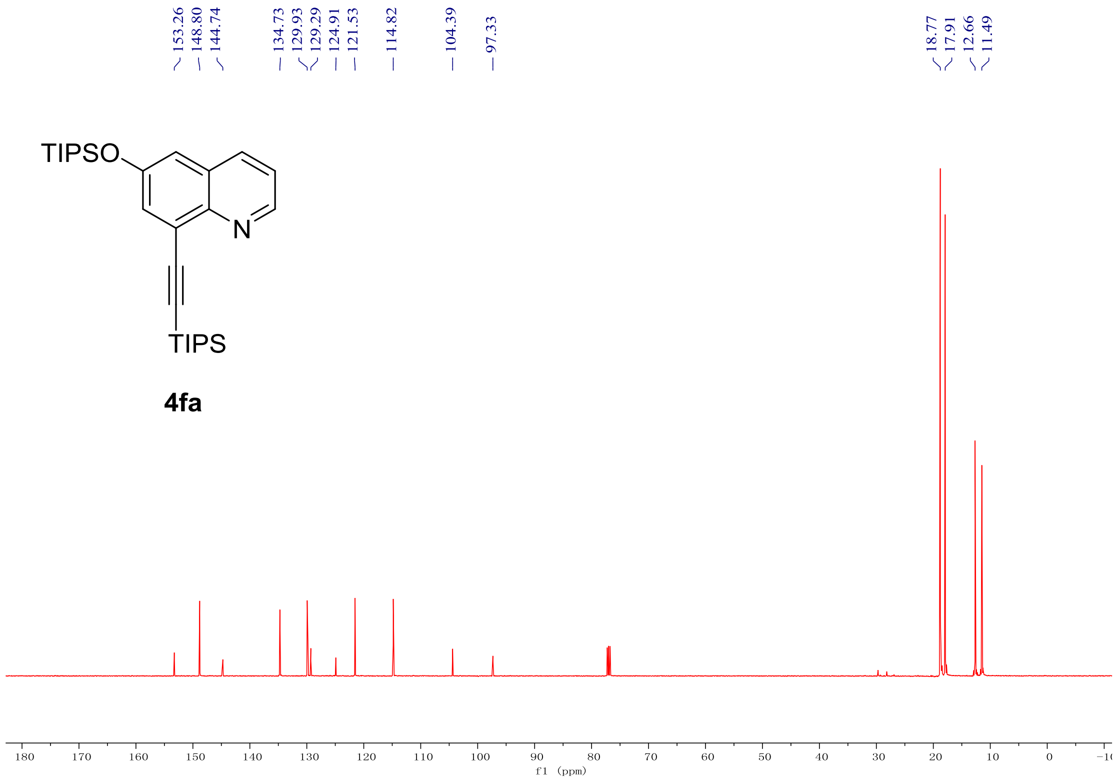

Supplementary Fig. 35. ^{13}C NMR spectra (126 MHz, $CDCl_3$, 25 ^{o}C)of **4fa**

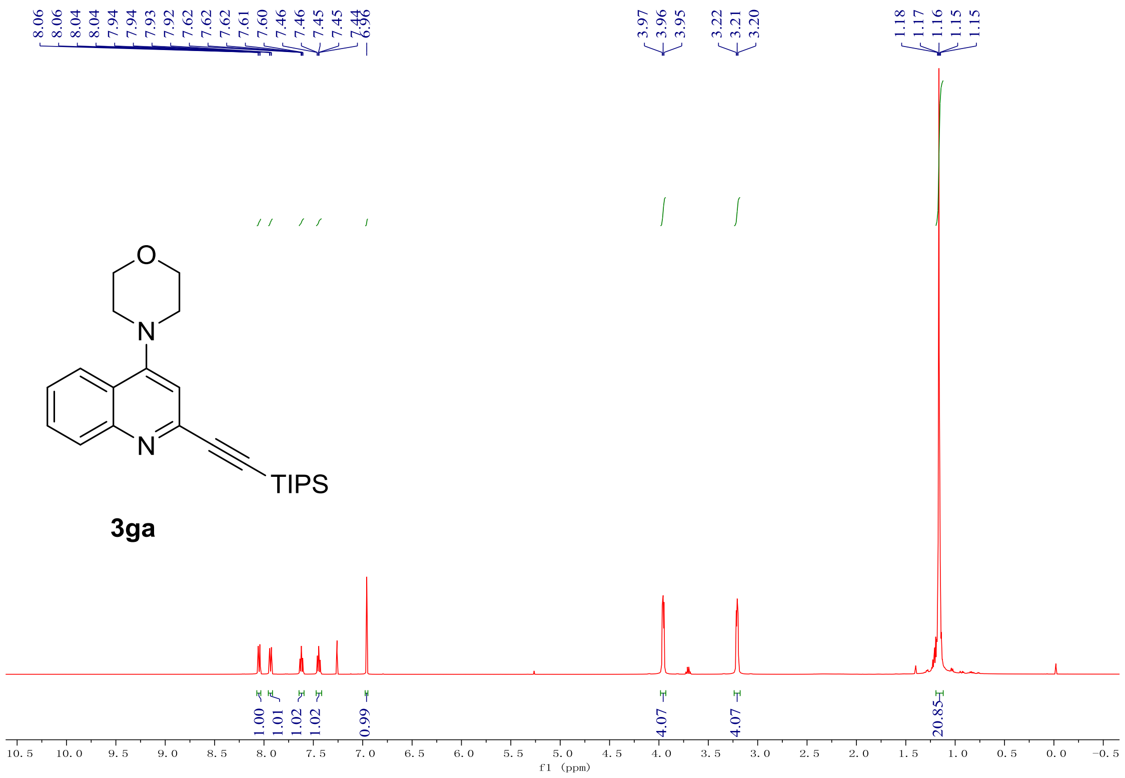

Supplementary Fig. 36. ^{1}H NMR spectra (500 MHz, $CDCl_3$, 25 ^{o}C) of **3ga**

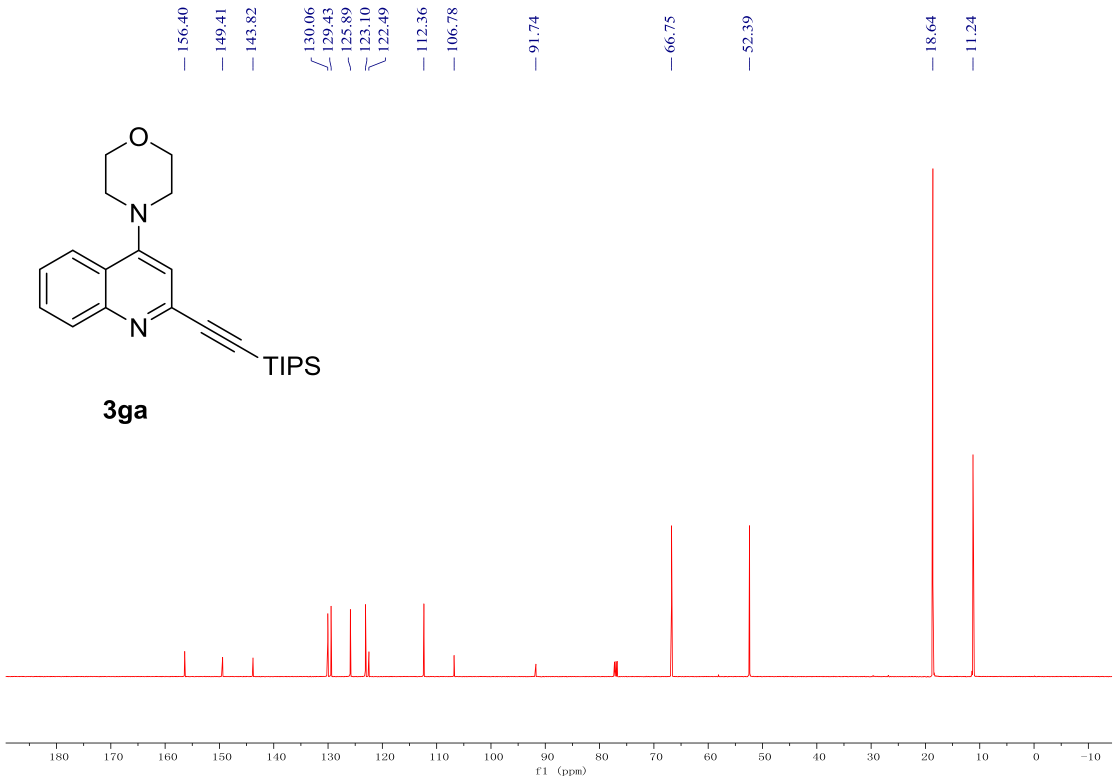

Supplementary Fig. 37. ^{13}C NMR spectra (126 MHz, $CDCl_3$, 25 ^{o}C) of **3ga**

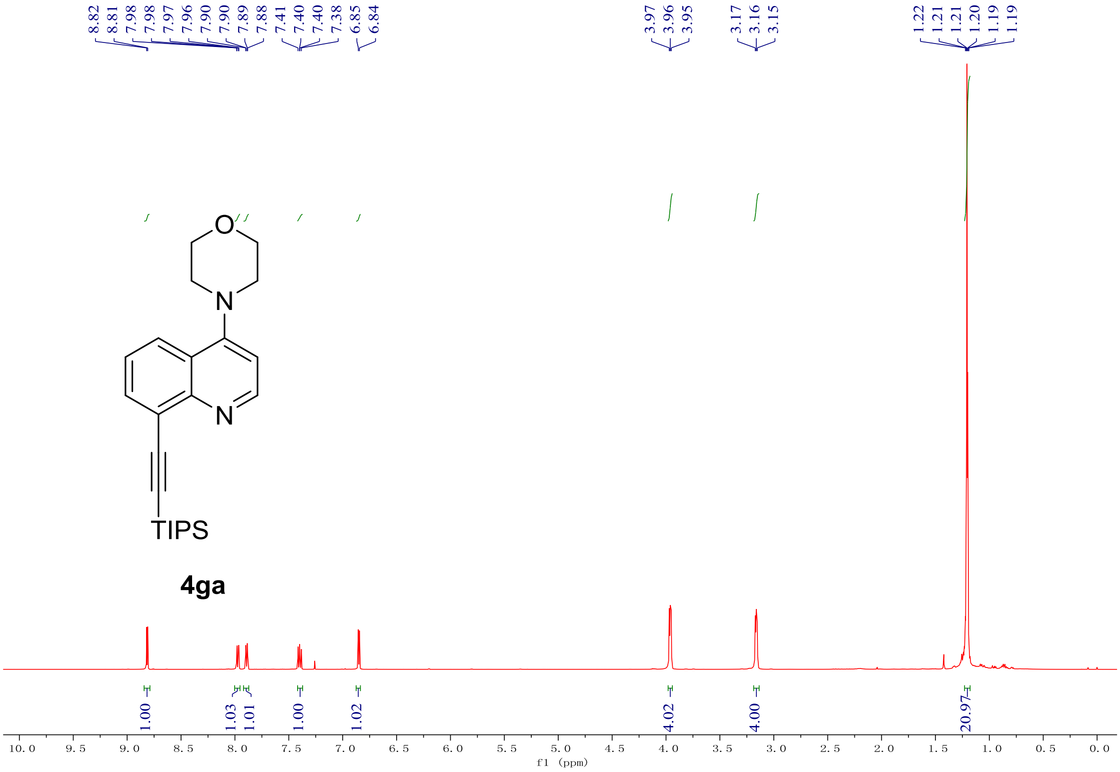

Supplementary Fig. 38. ^{1}H NMR spectra (500 MHz, $CDCl_3$, 25 °C) of **4ga**

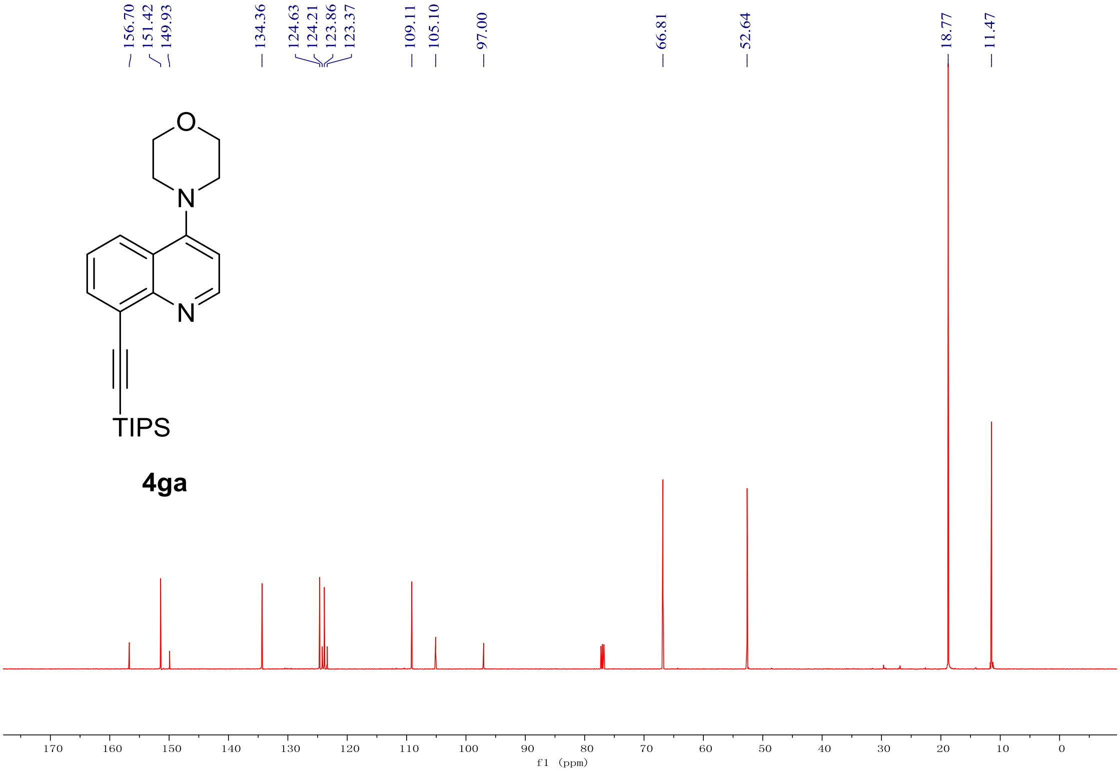

Supplementary Fig. 39. ^{13}C NMR spectra (126 MHz, $CDCl_3$, 25 °C) of **4ga**

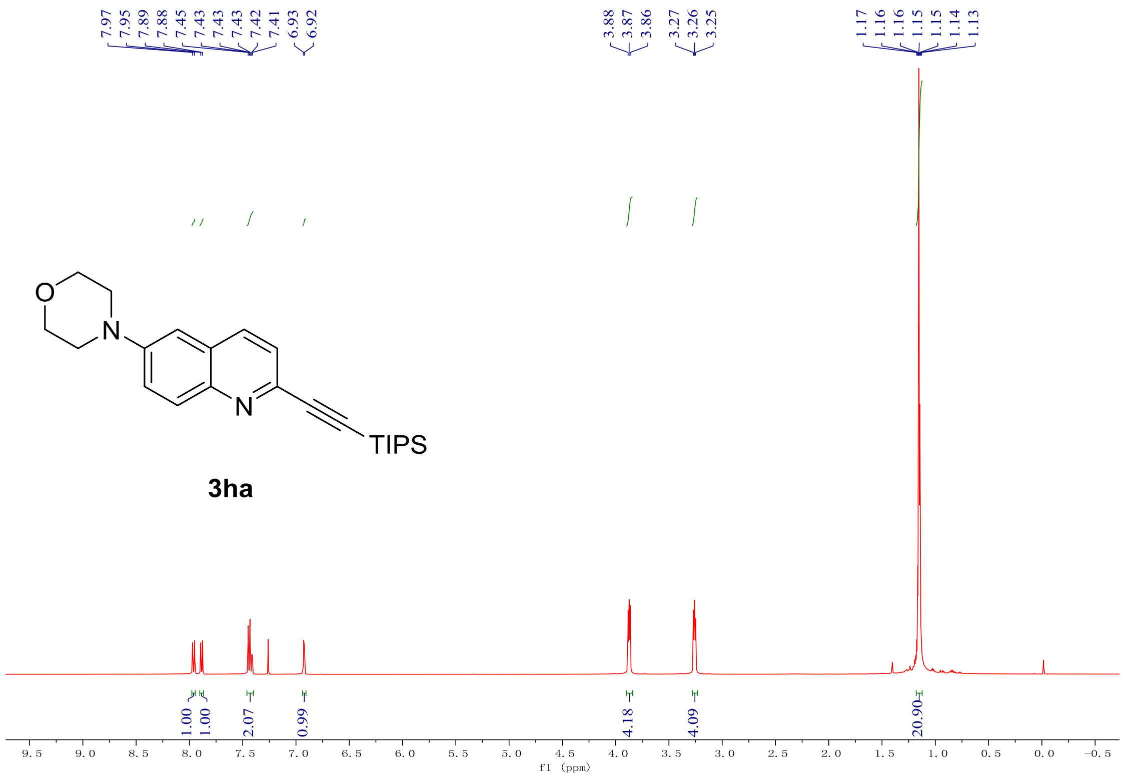

Supplementary Fig. 40. ^{1}H NMR spectra (500 MHz, $CDCl_3$, 25 °C) of **3ha**

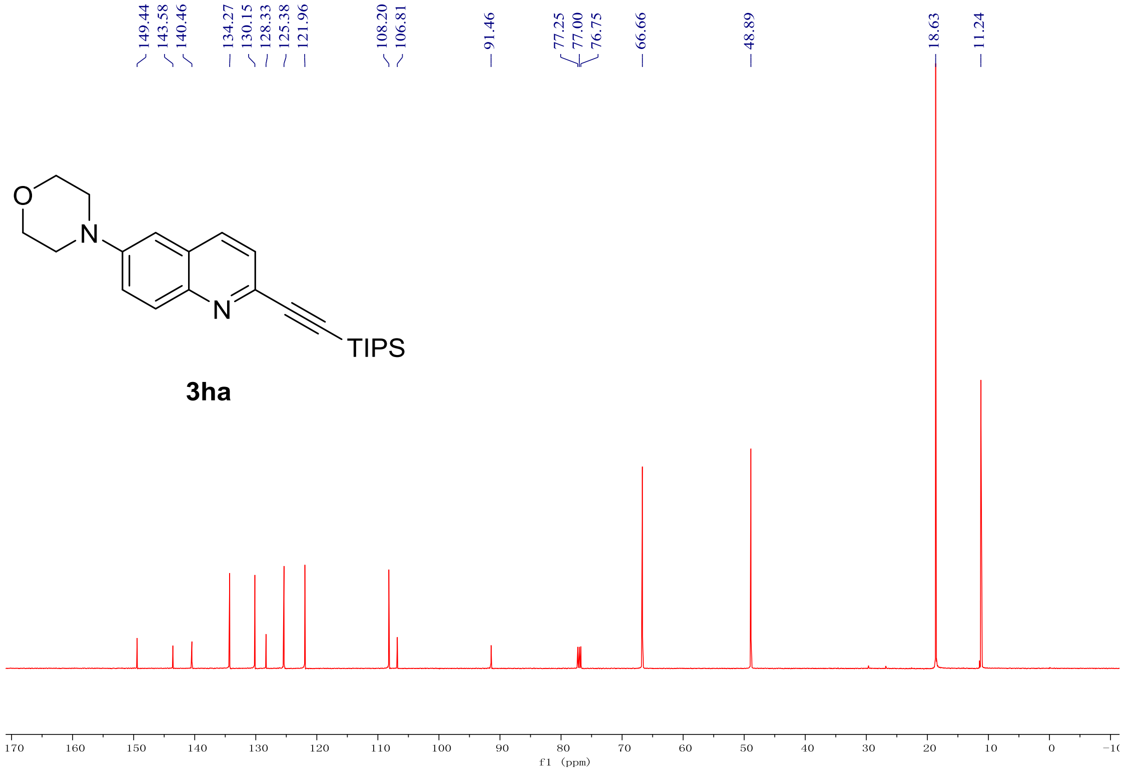

Supplementary Fig. 41. ^{13}C NMR spectra (126 MHz, $CDCl_3$, 25 °C) of **3ha**

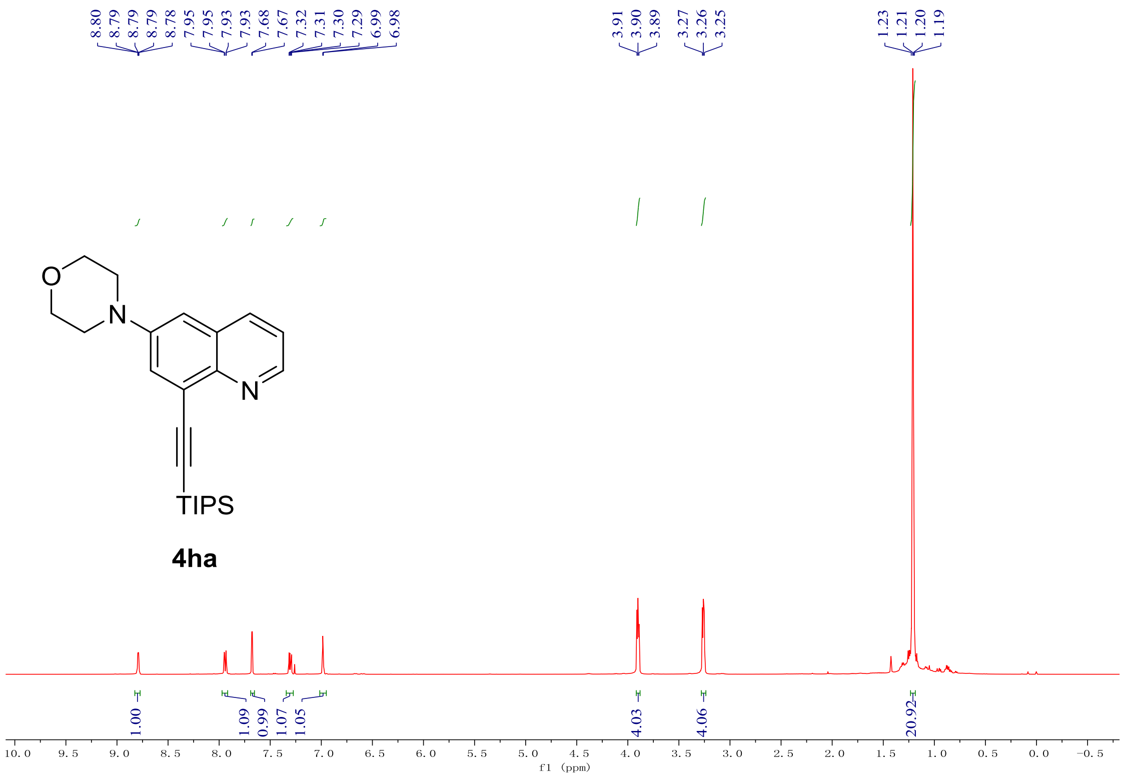

Supplementary Fig. 42. ^{1}H NMR spectra (500 MHz, $CDCl_3$, 25 °C) of **4ha**

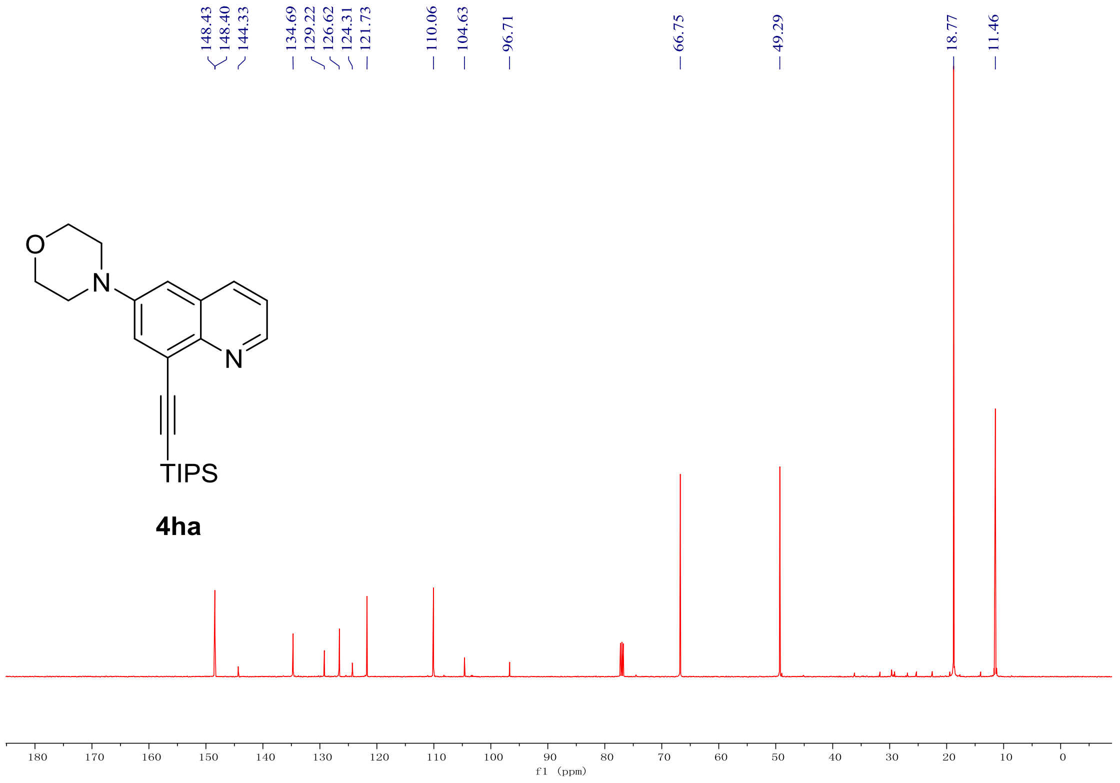

Supplementary Fig. 43. ^{13}C NMR spectra (126 MHz, $CDCl_3$, 25 °C) of **4ha**

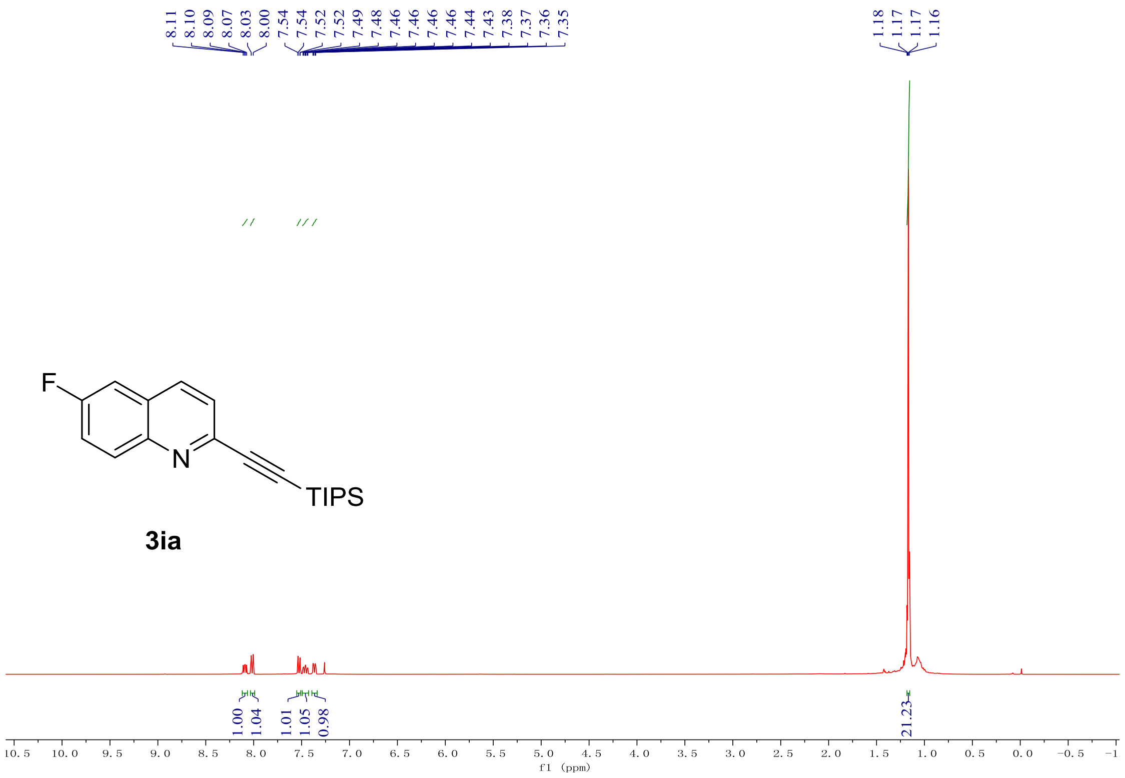

Supplementary Fig. 44. ^{1}H NMR spectra (400 MHz, $CDCl_3$, 25 ^{o}C) of **3ia**

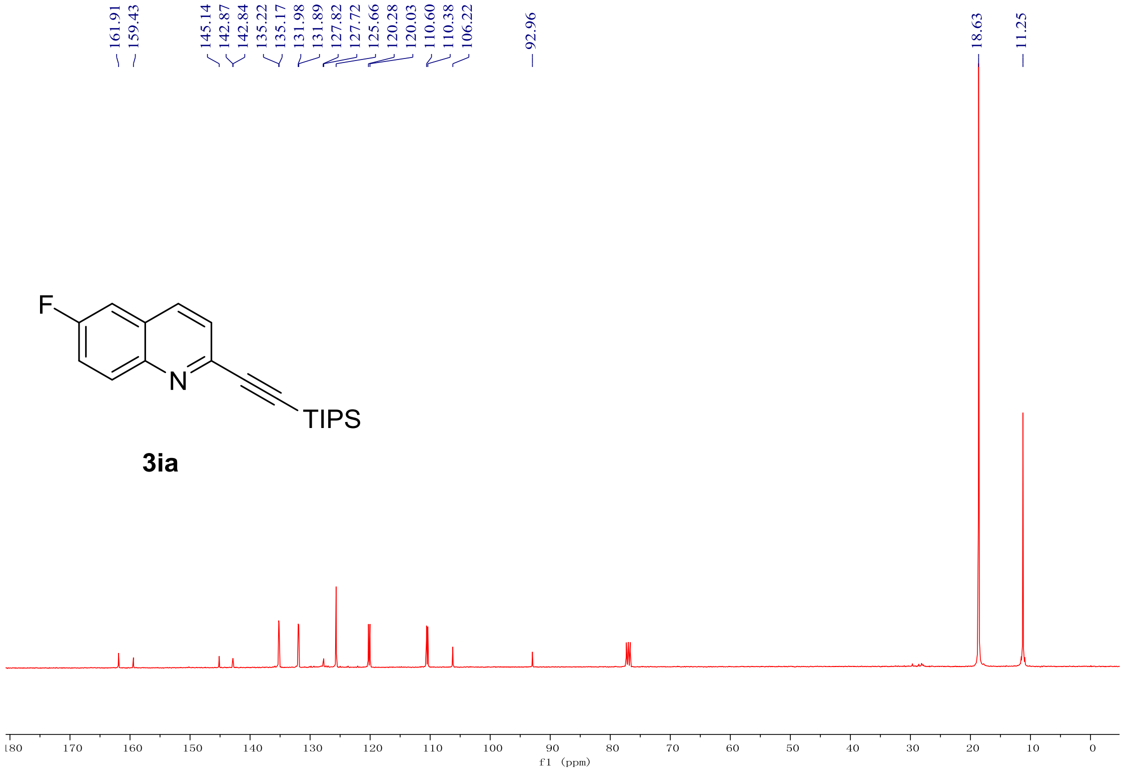

Supplementary Fig. 45. ^{13}C NMR spectra (101 MHz, $CDCl_3$, 25 ^{o}C) of **3ia**

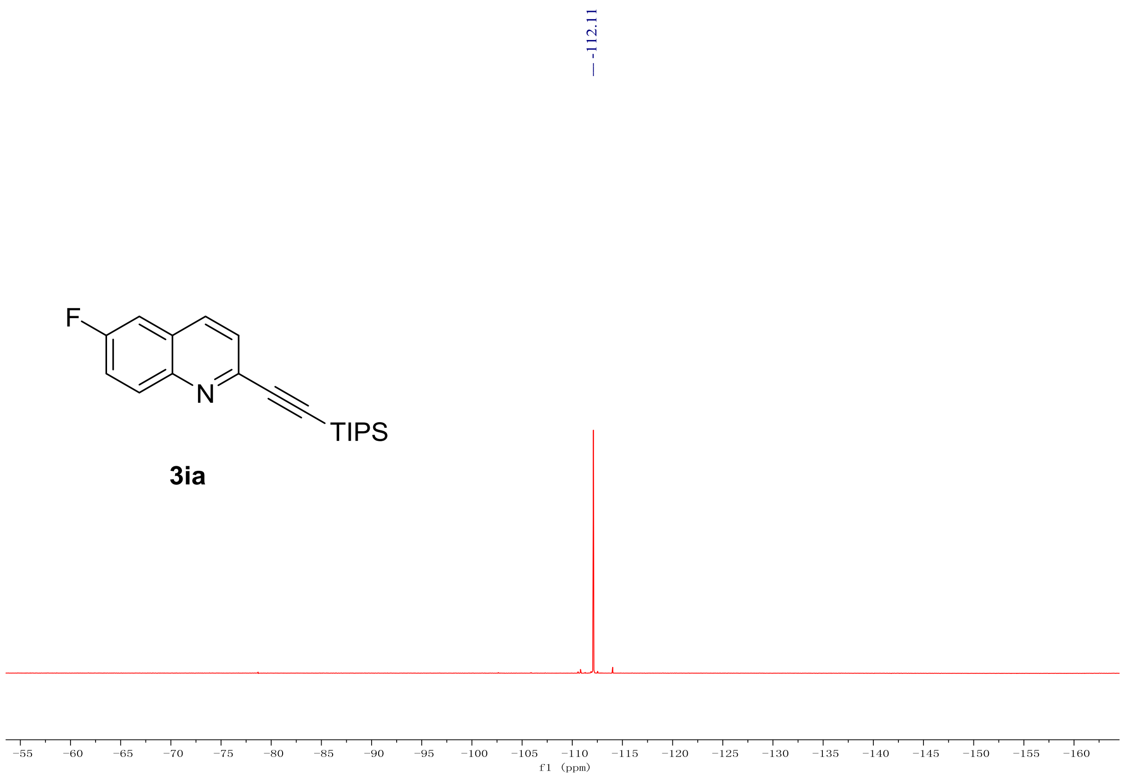

Supplementary Fig. 46. ^{19}F NMR spectra (376 MHz, $CDCl_3$, 25 ^{o}C) of **3ia**

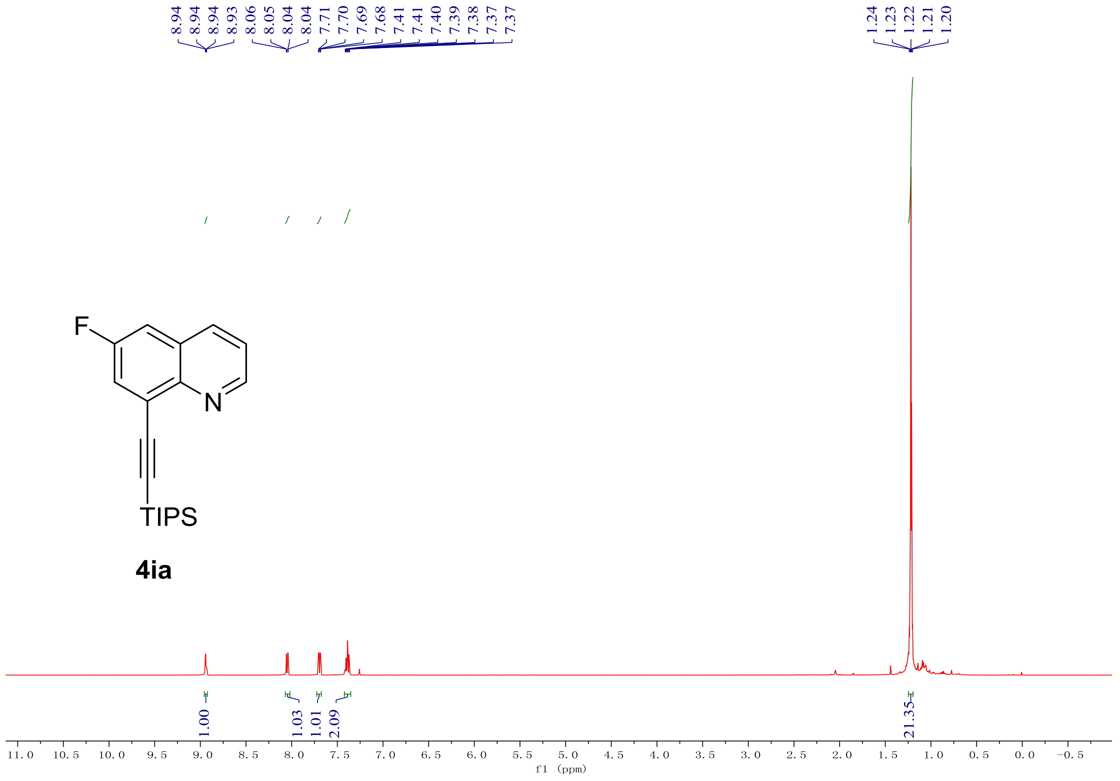

Supplementary Fig. 47. ^{1}H NMR spectra (500 MHz, $CDCl_3$, 25 ^{o}C) of **4ia**

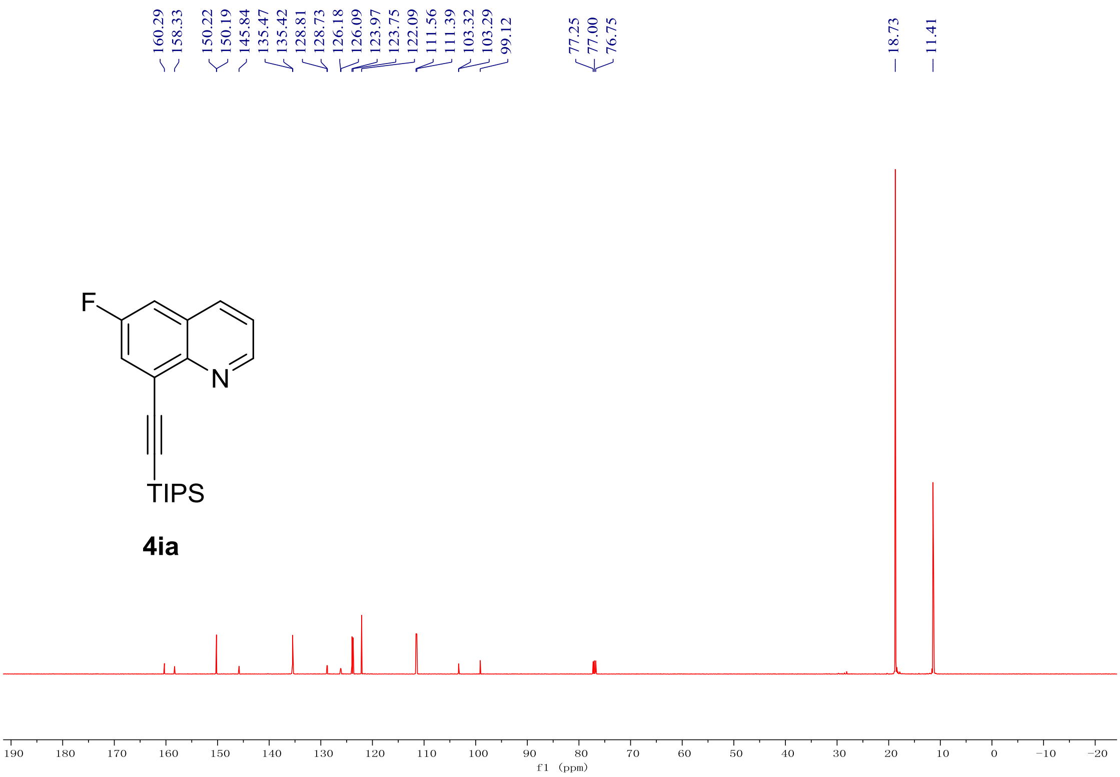

Supplementary Fig. 48. ^{13}C NMR spectra (126 MHz, $CDCl_3$, 25 °C) of **4ia**

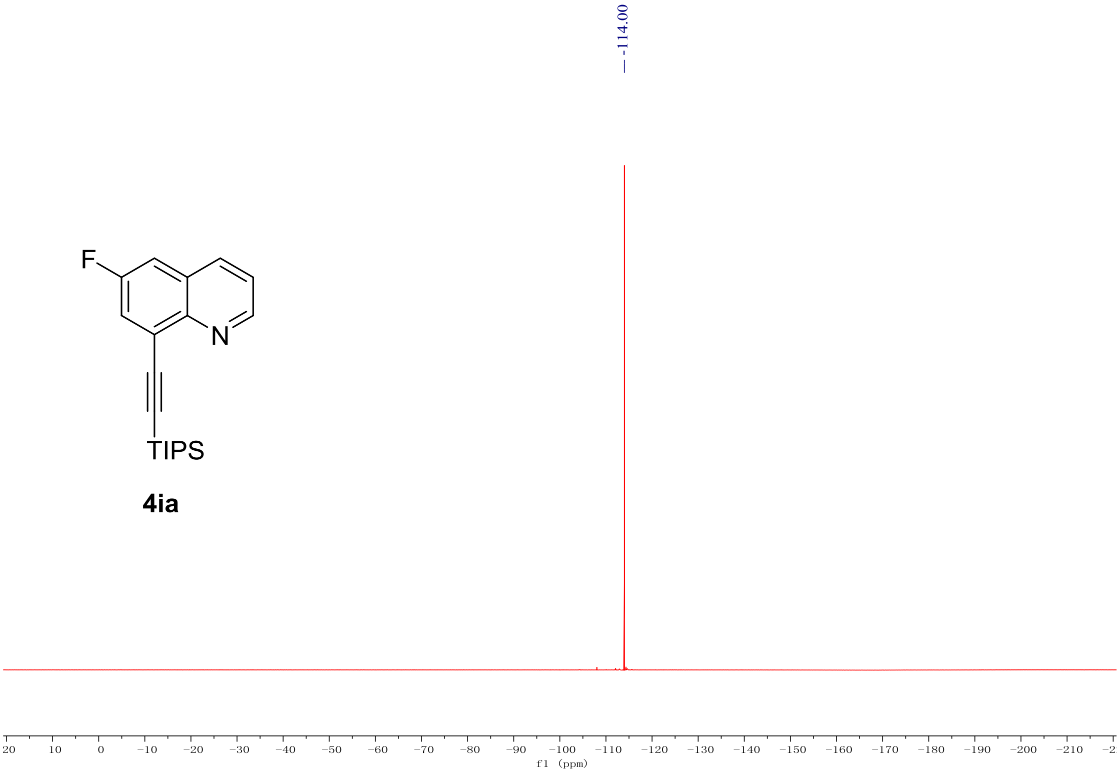

Supplementary Fig. 49. ^{19}F NMR spectra (471 MHz, $CDCl_3$, 25 °C) of **4ia**

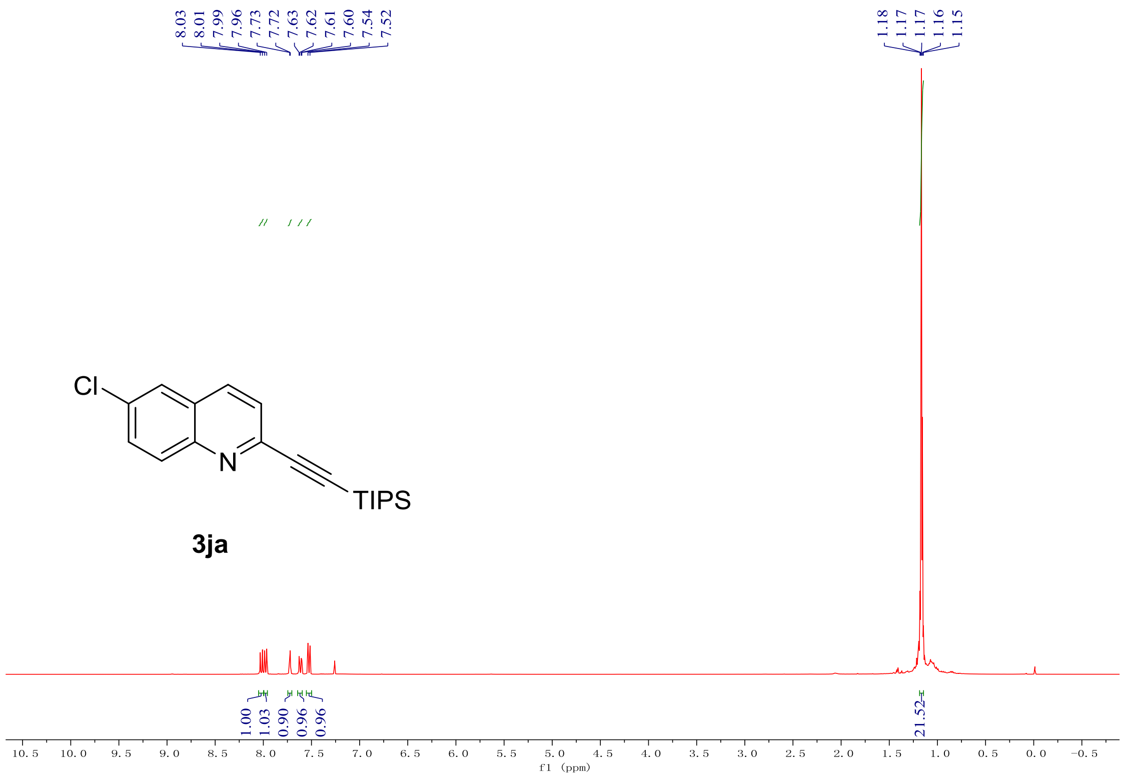

Supplementary Fig. 50. ^{1}H NMR spectra (400 MHz, $CDCl_3$, 25 °C) of **3ja**

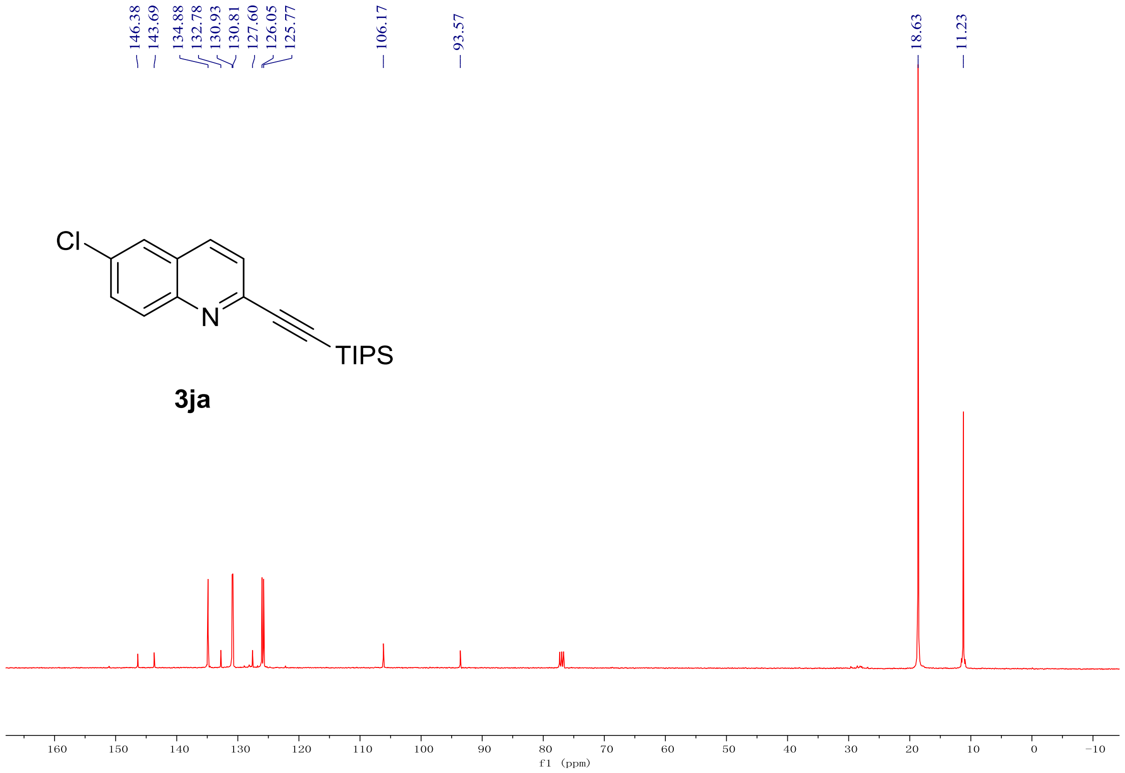

Supplementary Fig. 51. ^{13}C NMR spectra (101 MHz, $CDCl_3$, 25 °C) of **3ja**

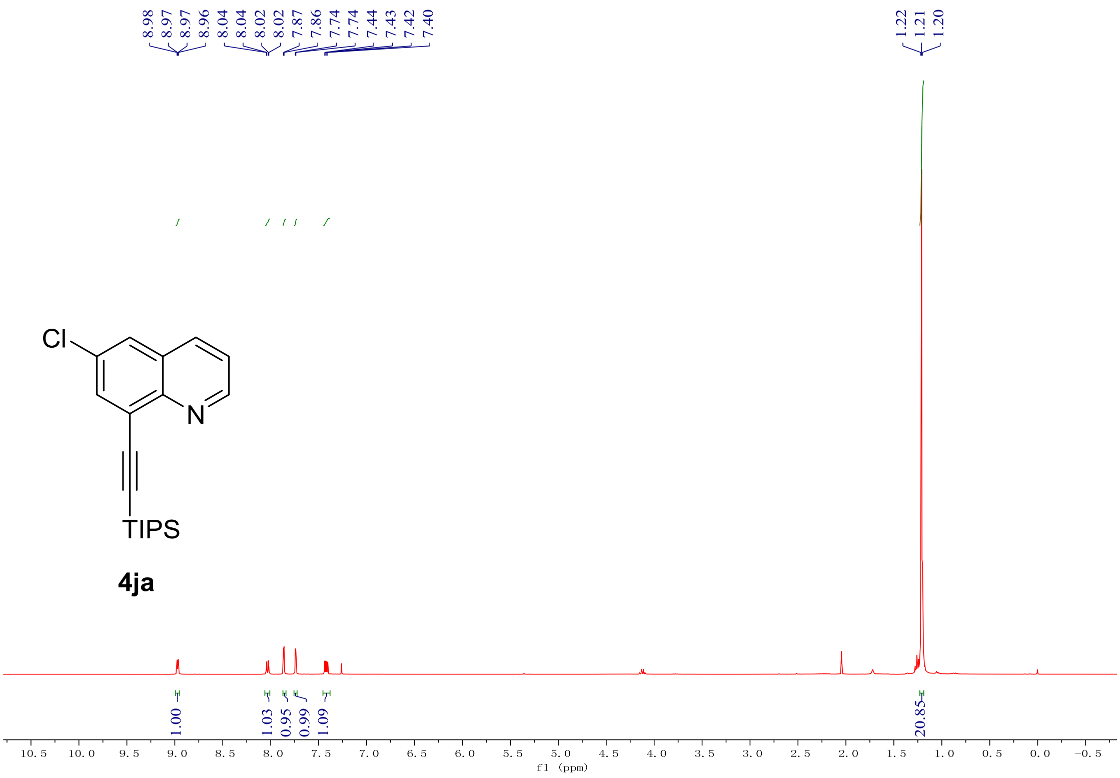

Supplementary Fig. 52. ^{1}H NMR spectra (400 MHz, $CDCl_3$, 25 ^{o}C) of **4ja**

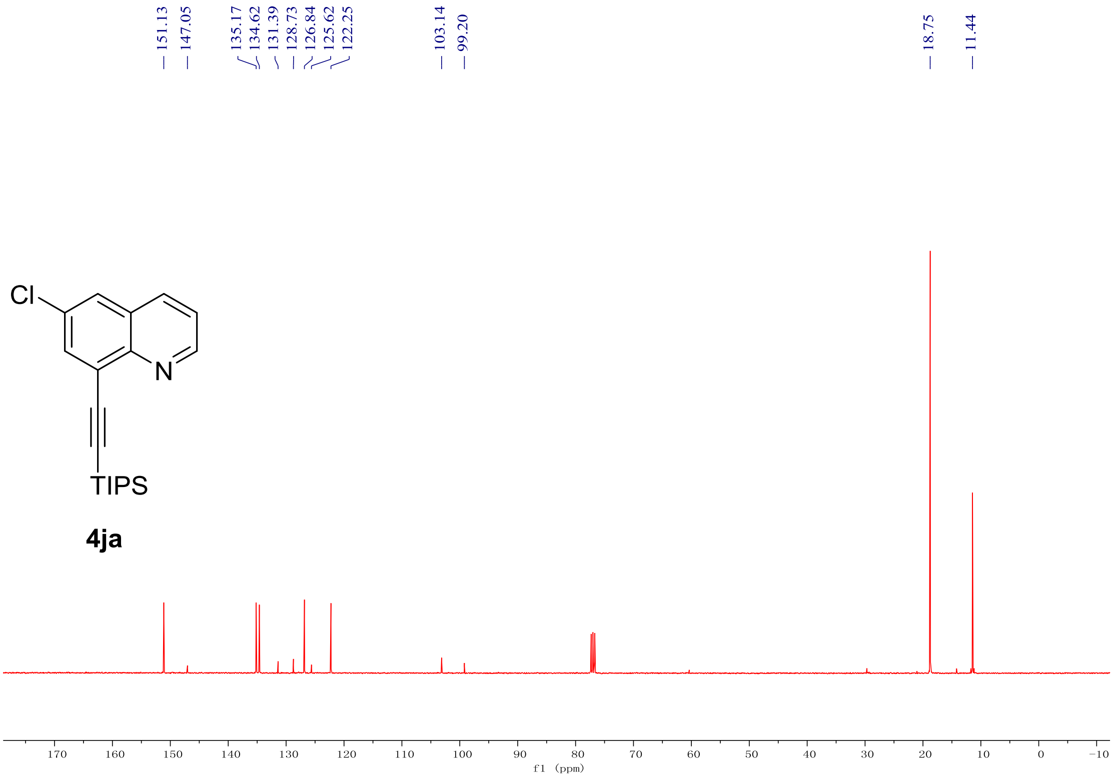

Supplementary Fig. 53. ^{13}C NMR spectra (101 MHz, $CDCl_3$, 25 ^{o}C) of **4ja**

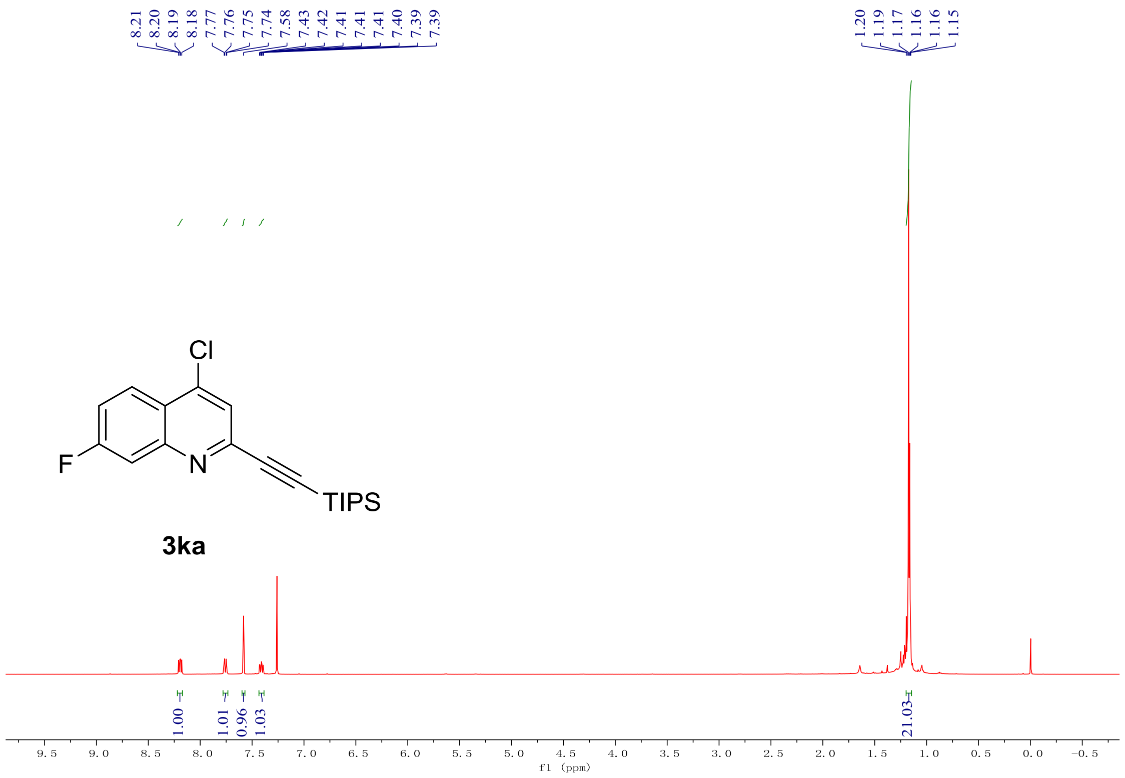

Supplementary Fig. 54. ^{1}H NMR spectra (500 MHz, $CDCl_3$, 25 °C) of **3ka**

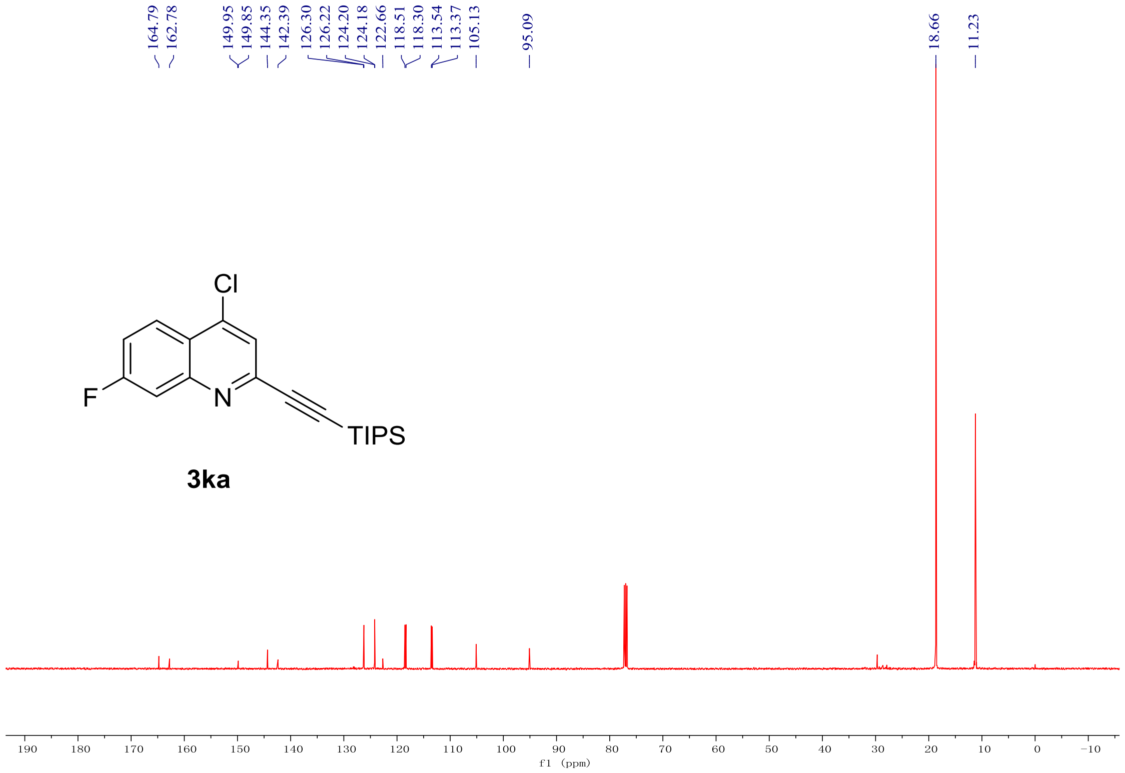

Supplementary Fig. 55. ^{13}C NMR spectra (126 MHz, $CDCl_3$, 25 °C) of **3ka**

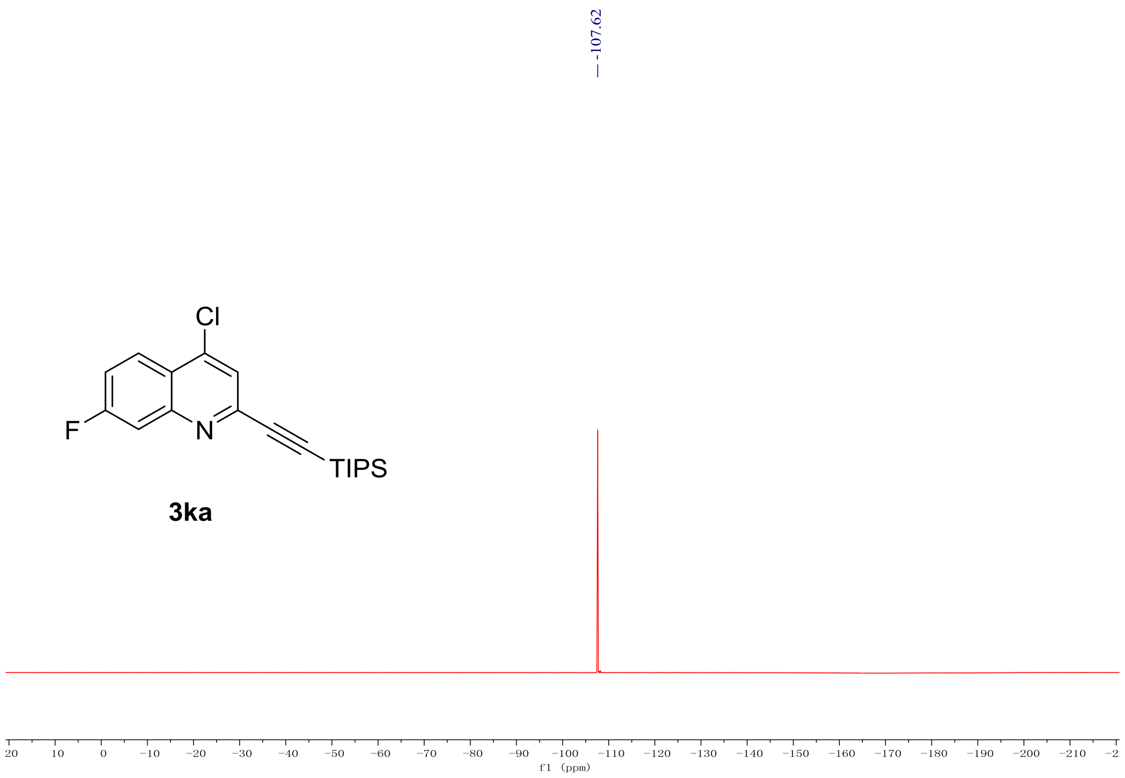

Supplementary Fig. 56. ^{19}F NMR spectra (471 MHz, $CDCl_3$, 25 ^{o}C) of **3ka**

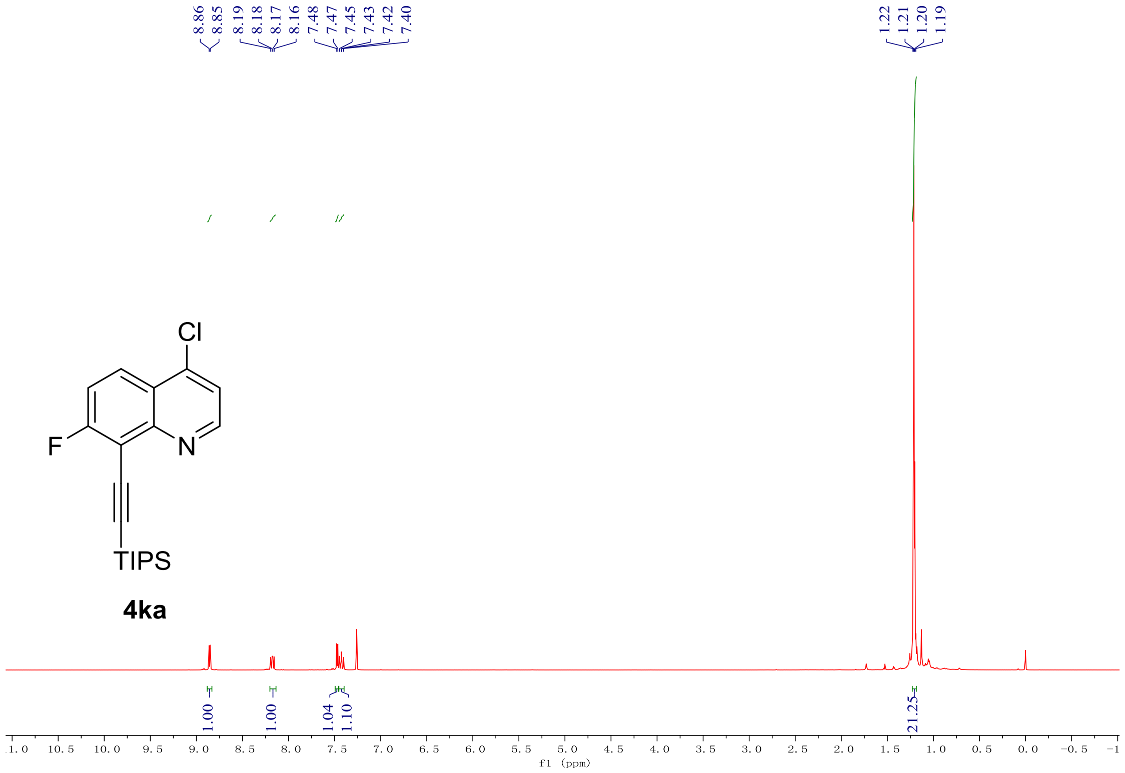

Supplementary Fig. 57. ^{1}H NMR spectra (400 MHz, $CDCl_3$, 25 ^{o}C) of **4ka**

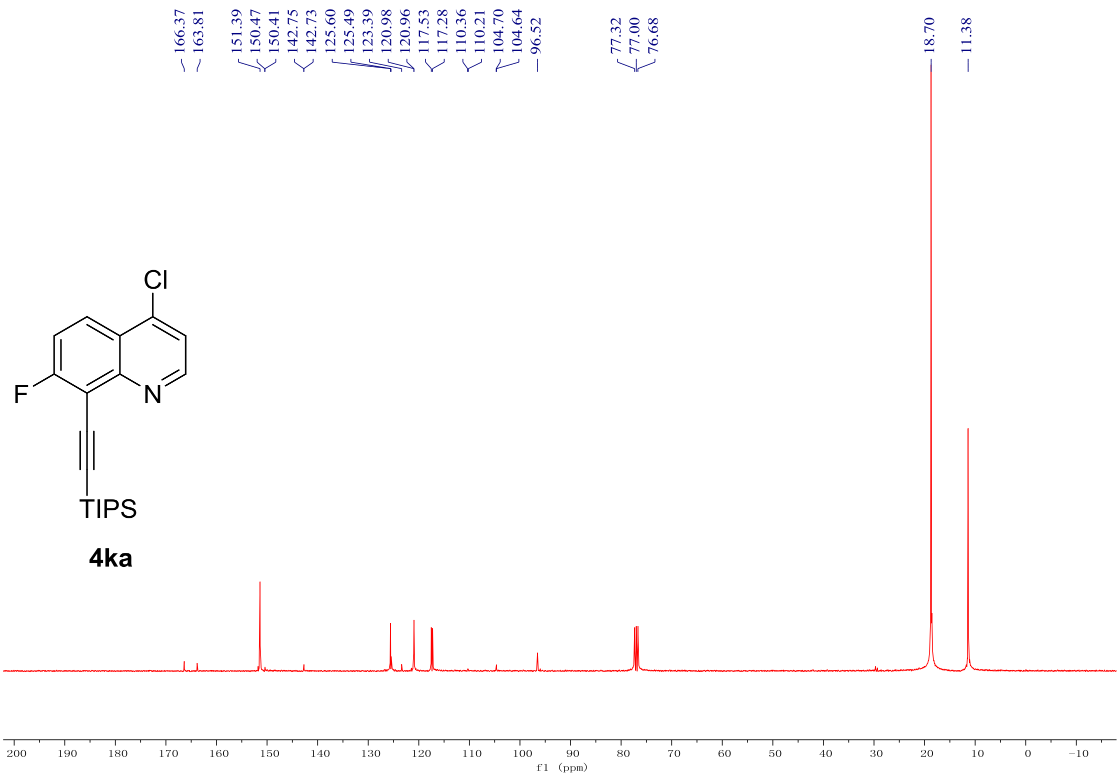

Supplementary Fig. 58. ^{13}C NMR spectra (101 MHz, $CDCl_3$, 25 °C) of **4ka**

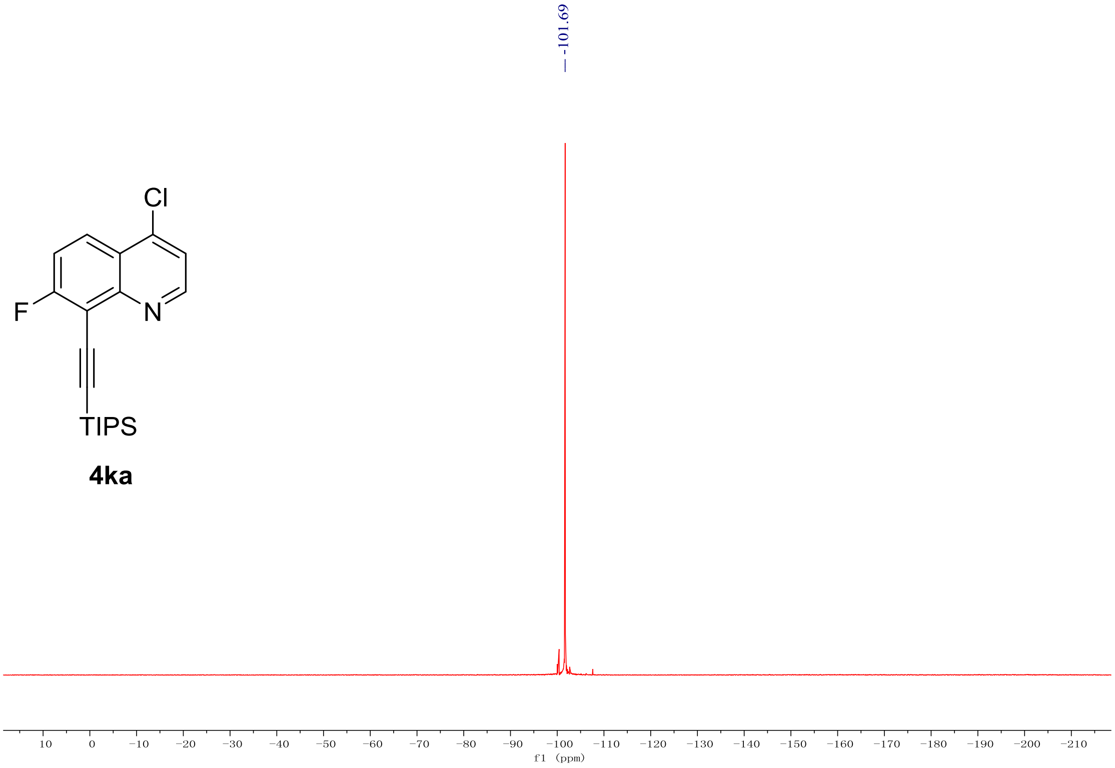

Supplementary Fig. 59. ^{19}F NMR spectra (376 MHz, $CDCl_3$, 25 °C) of **4ka**

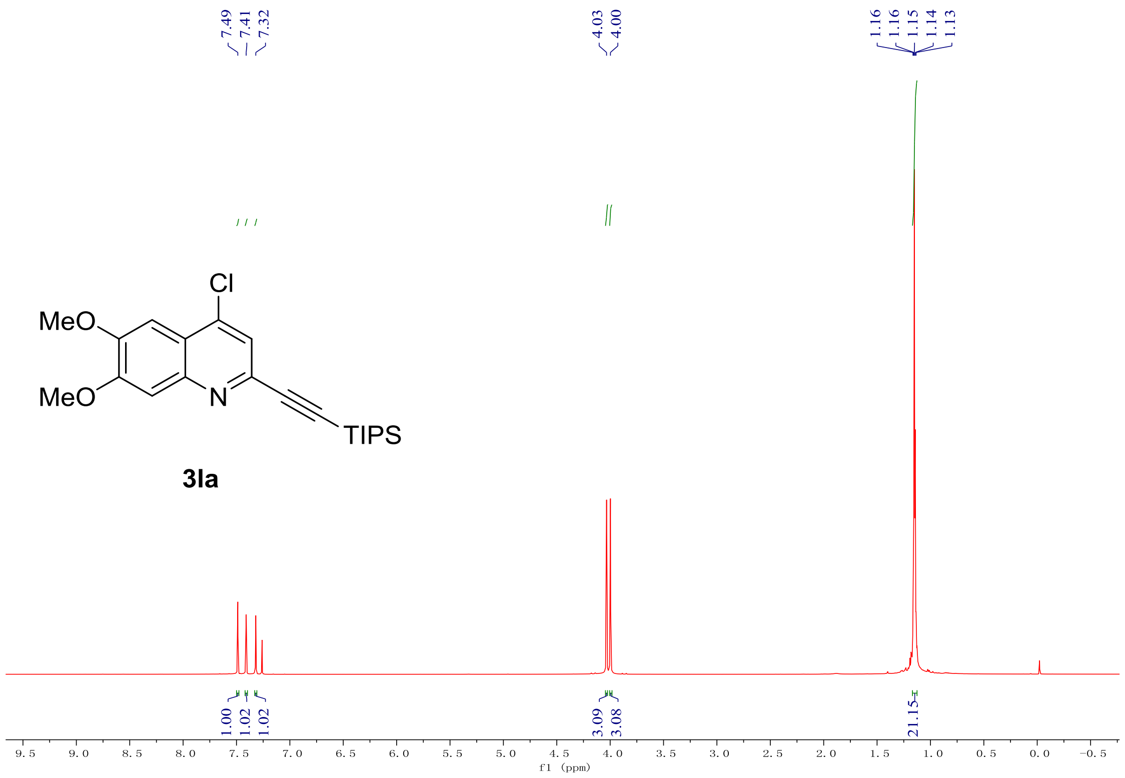

Supplementary Fig. 60. ^{1}H NMR spectra (500 MHz, $CDCl_3$, 25 °C) of **3la**

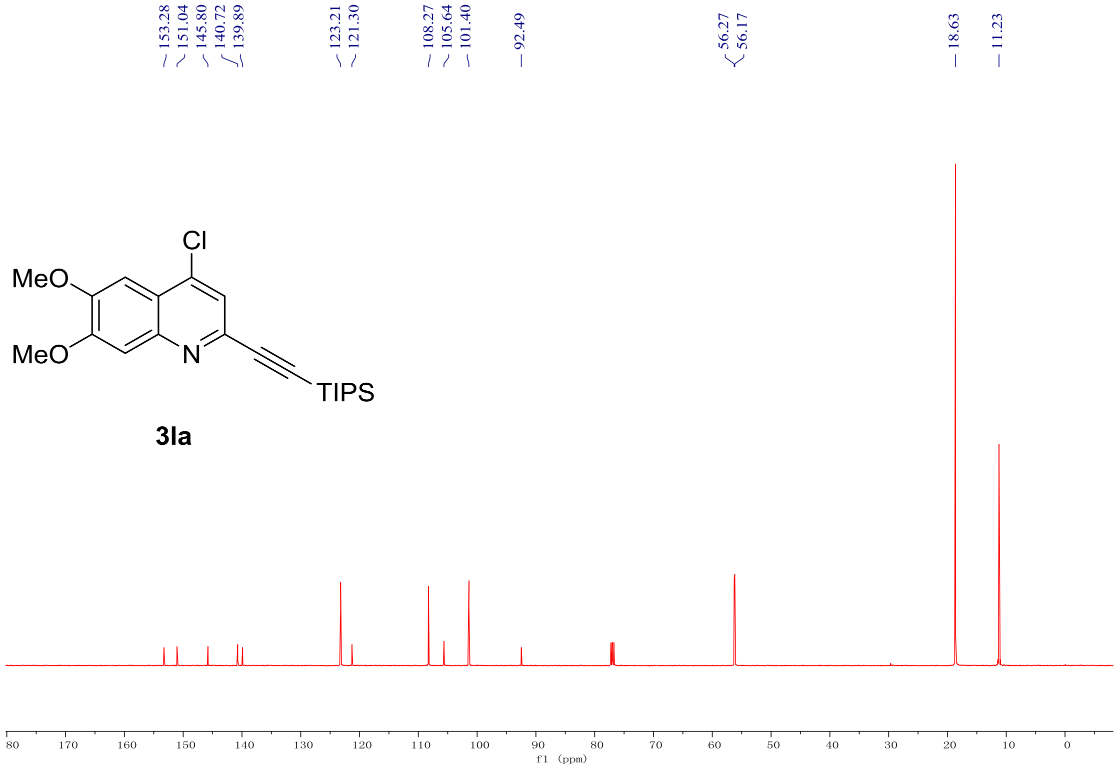

Supplementary Fig. 61. ^{13}C NMR spectra (126 MHz, $CDCl_3$, 25 °C) of **3la**

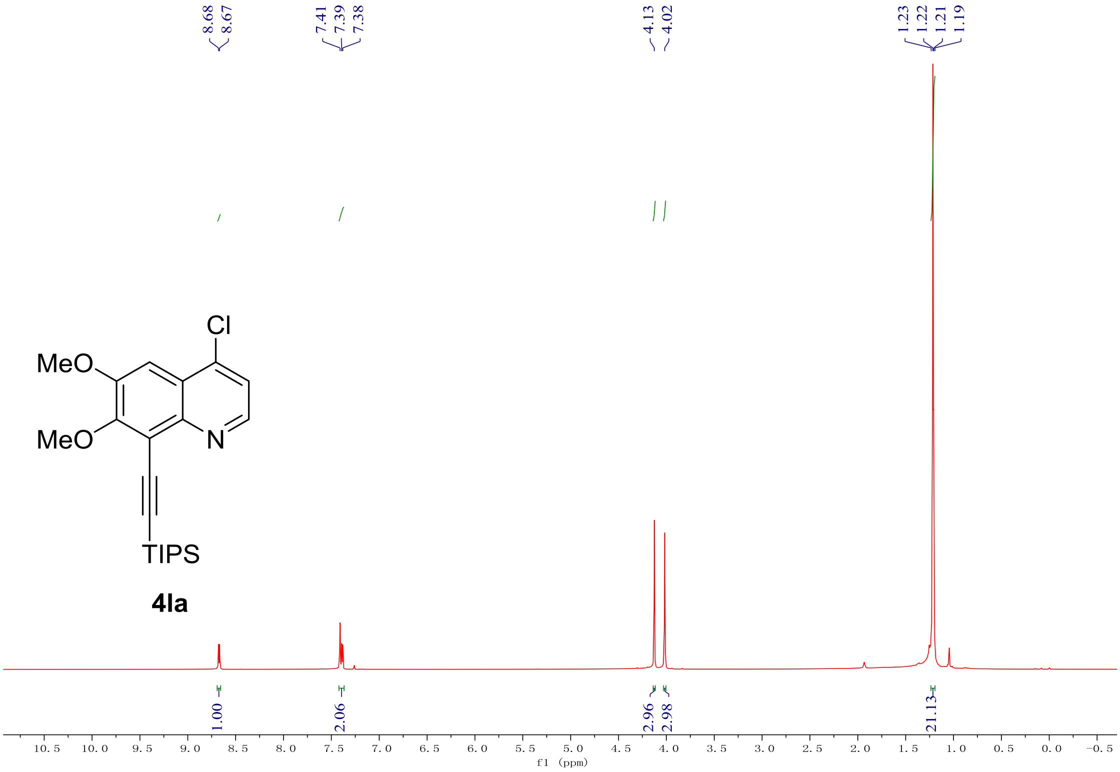

Supplementary Fig. 62. ^{1}H NMR spectra (400 MHz, $CDCl_3$, 25 °C) of **4la**

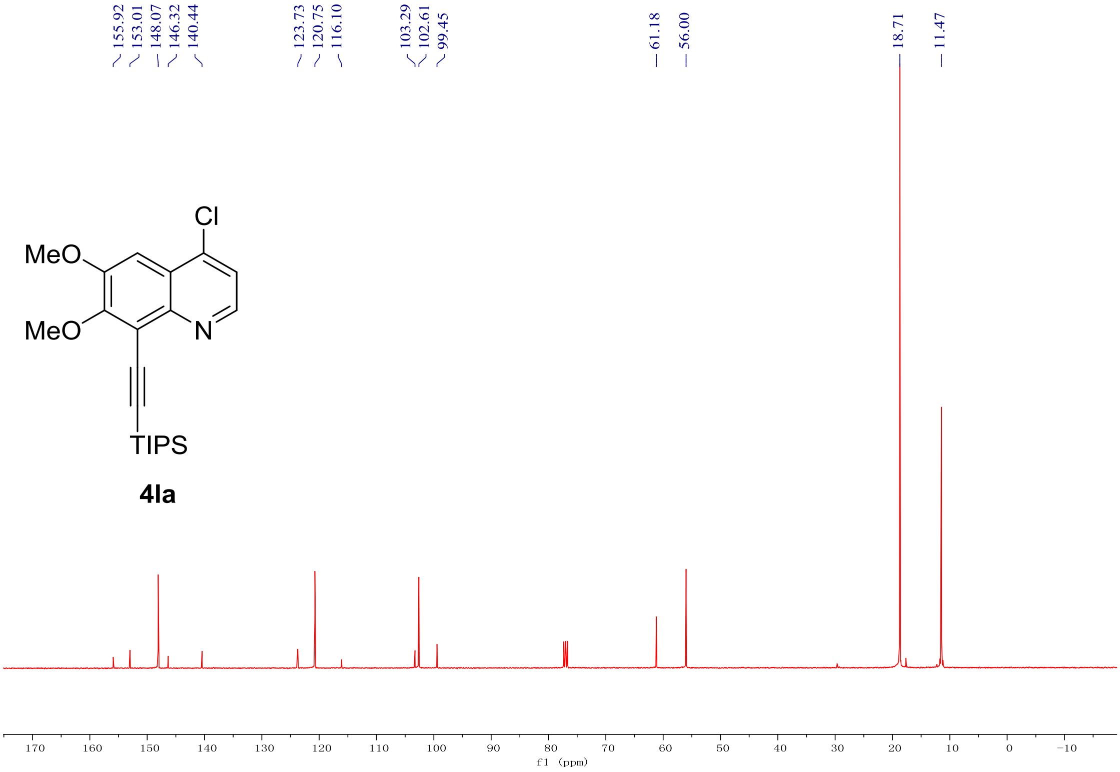

Supplementary Fig. 63. ^{13}C NMR spectra (101 MHz, $CDCl_3$, 25 °C)of **4la**

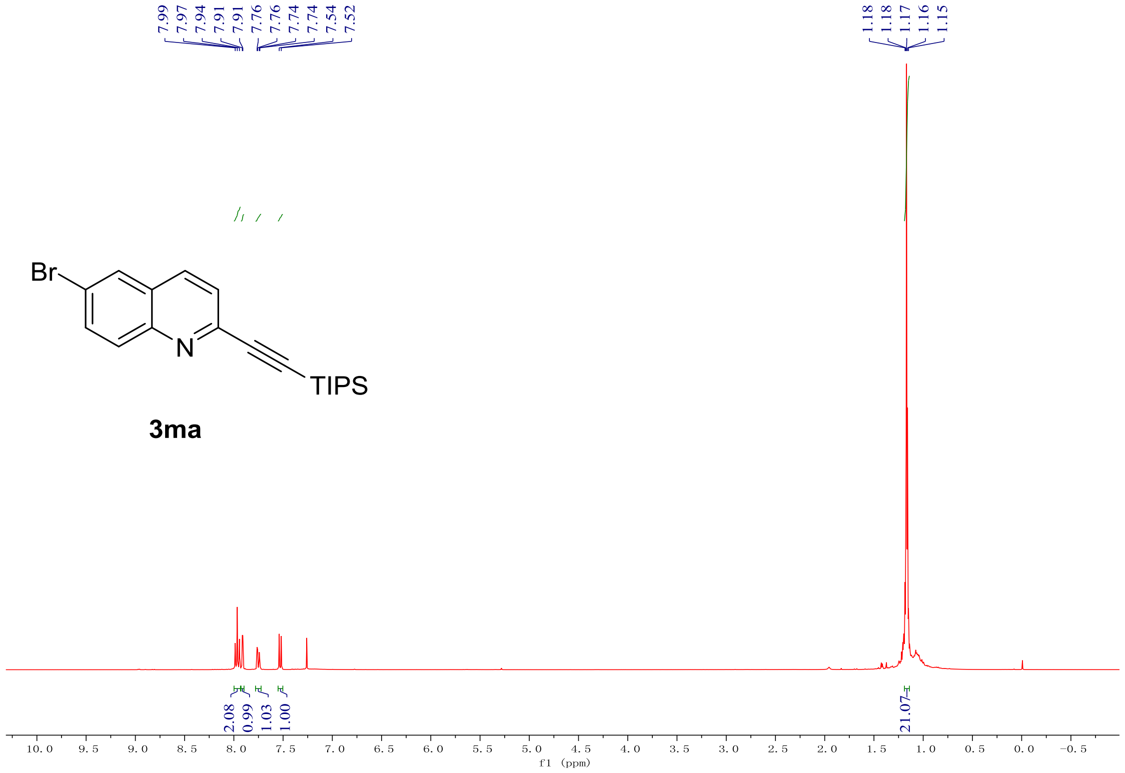

Supplementary Fig. 64. ^{1}H NMR spectra (400 MHz, $CDCl_3$, 25 °C) of **3ma**

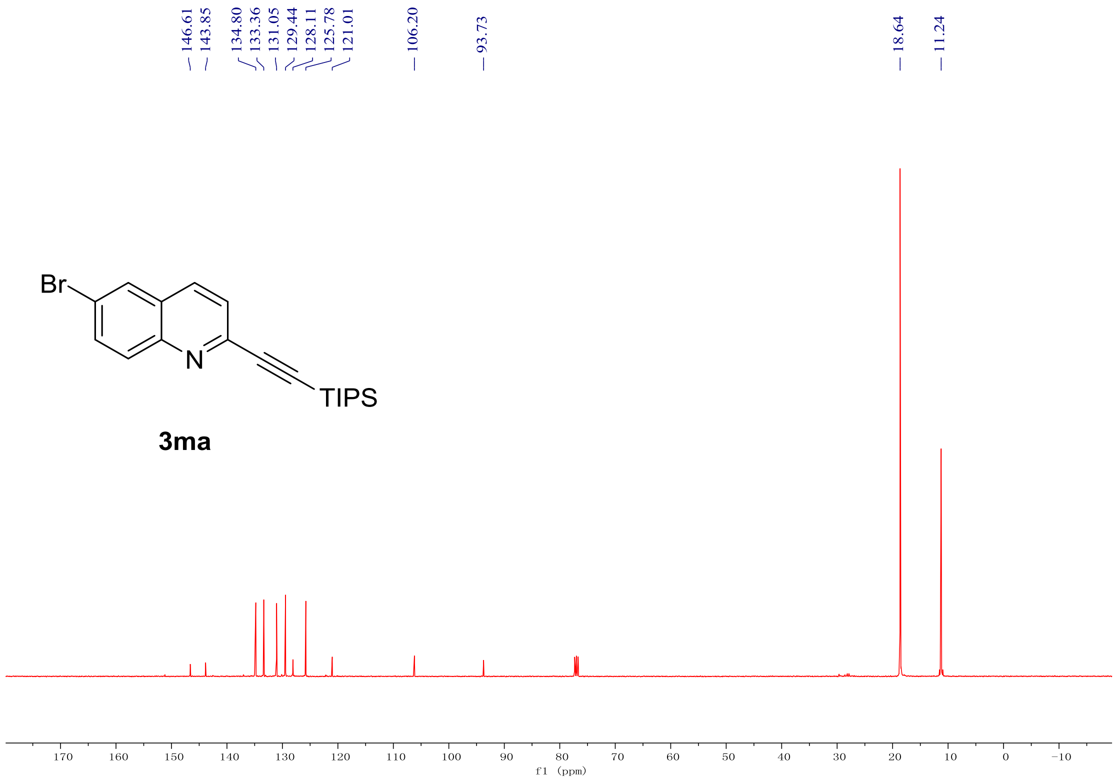

Supplementary Fig. 65. ^{13}C NMR spectra (101 MHz, $CDCl_3$, 25 °C) of **3ma**

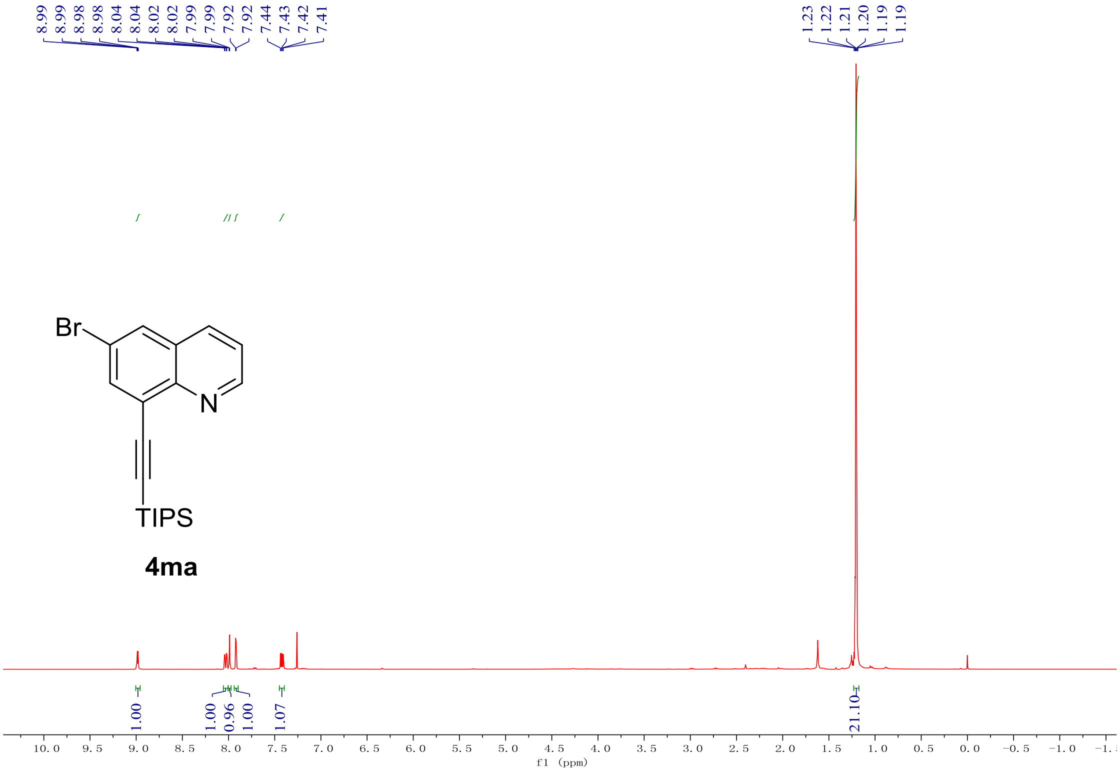

Supplementary Fig. 66. ^{1}H NMR spectra (400 MHz, $CDCl_3$, 25 ^{o}C) of **4ma**

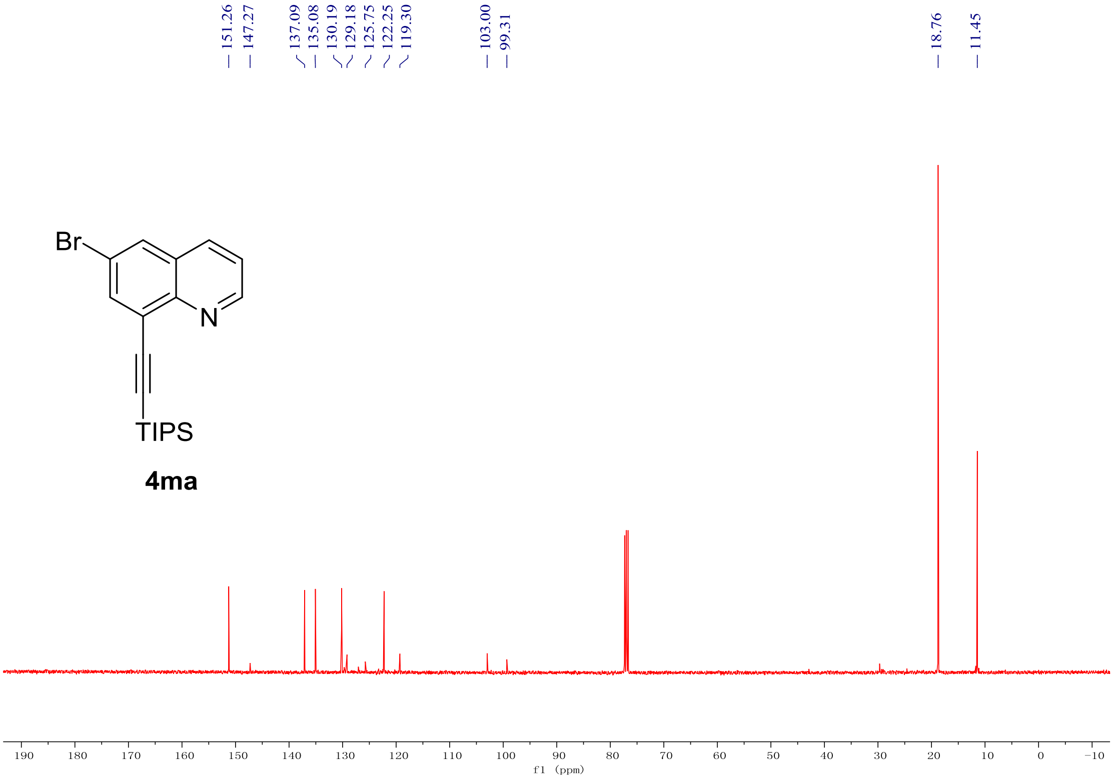

Supplementary Fig. 67. ^{13}C NMR spectra (101 MHz, $CDCl_3$, 25 ^{o}C) of **4ma**

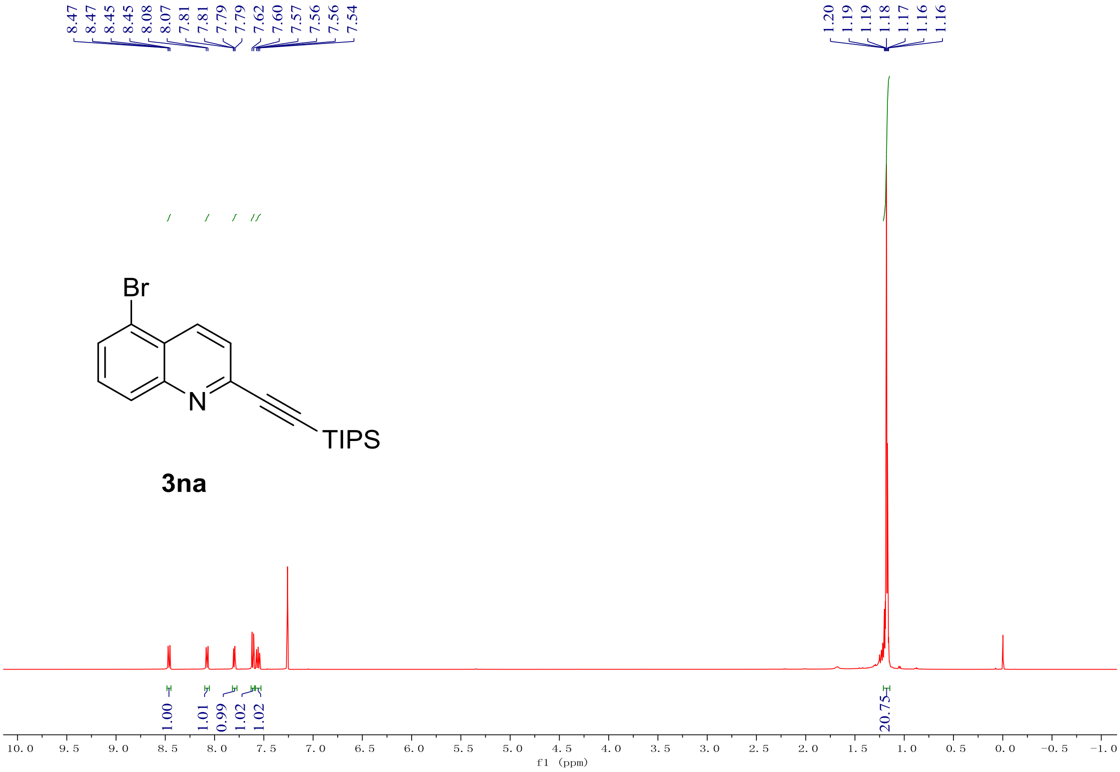

Supplementary Fig. 68. ^{1}H NMR spectra (500 MHz, $CDCl_3$, 25 °C) of **3na**

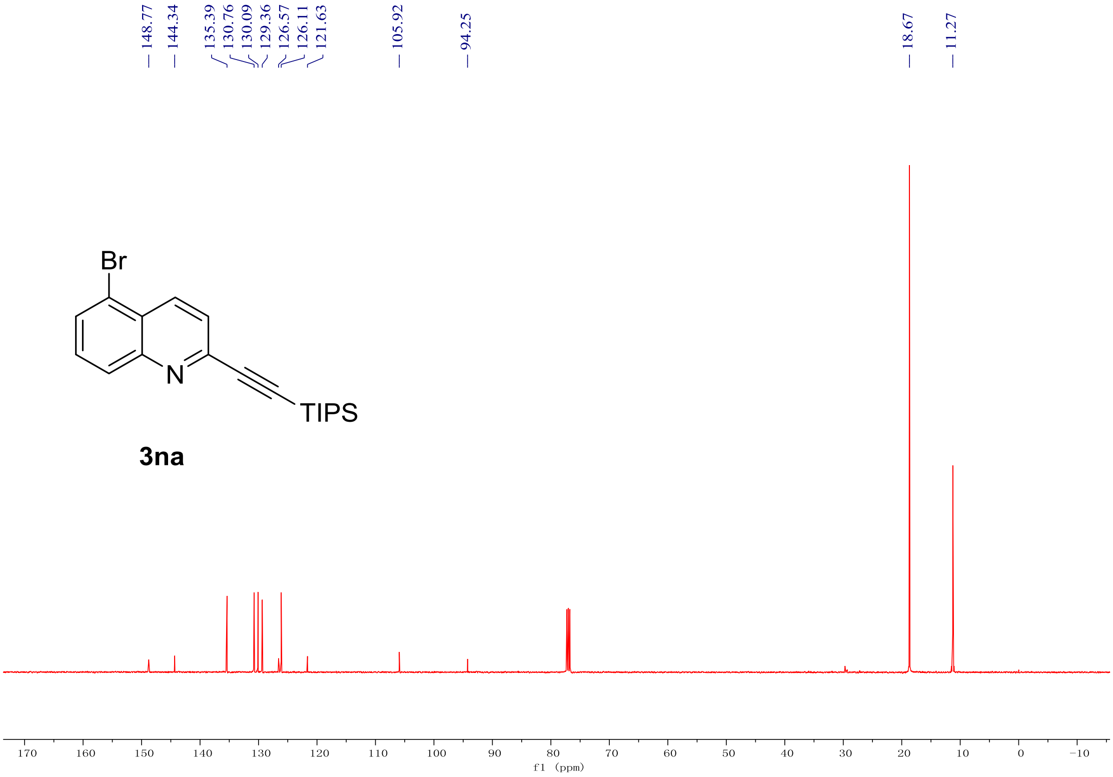

Supplementary Fig. 69. ^{13}C NMR spectra (126 MHz, $CDCl_3$, 25 °C) of **3na**

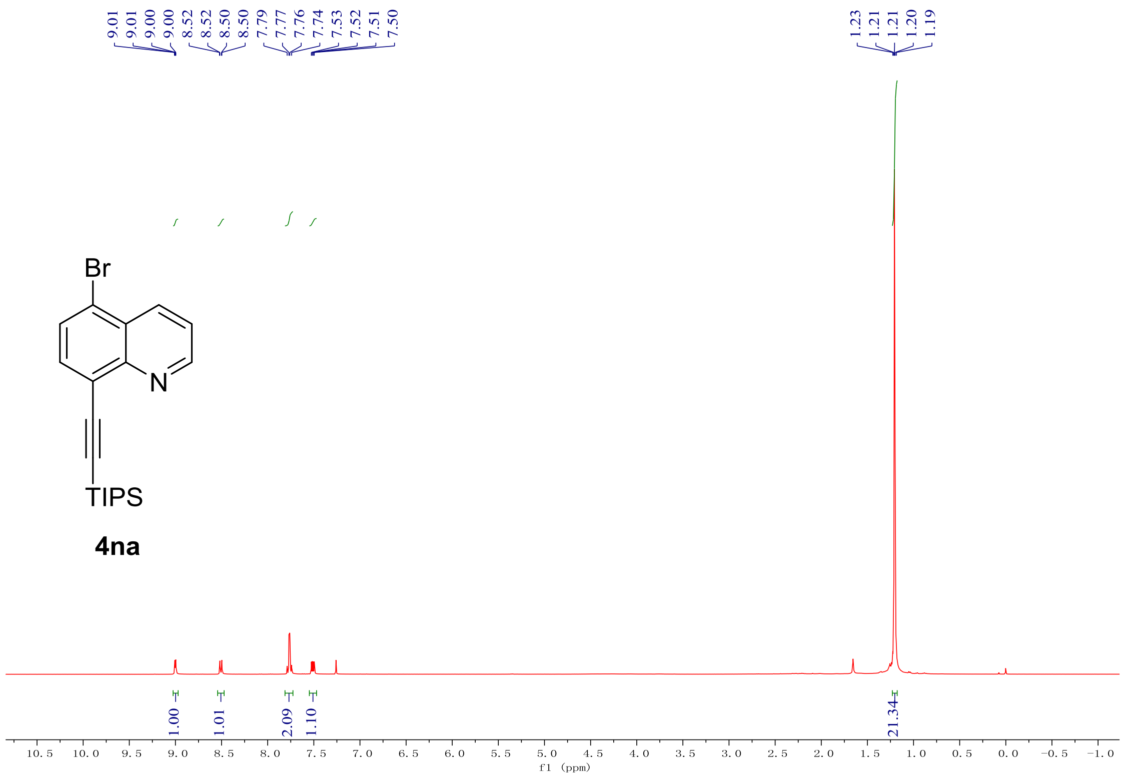

Supplementary Fig. 70. ^{1}H NMR spectra (400 MHz, $CDCl_3$, 25 °C) of **4na**

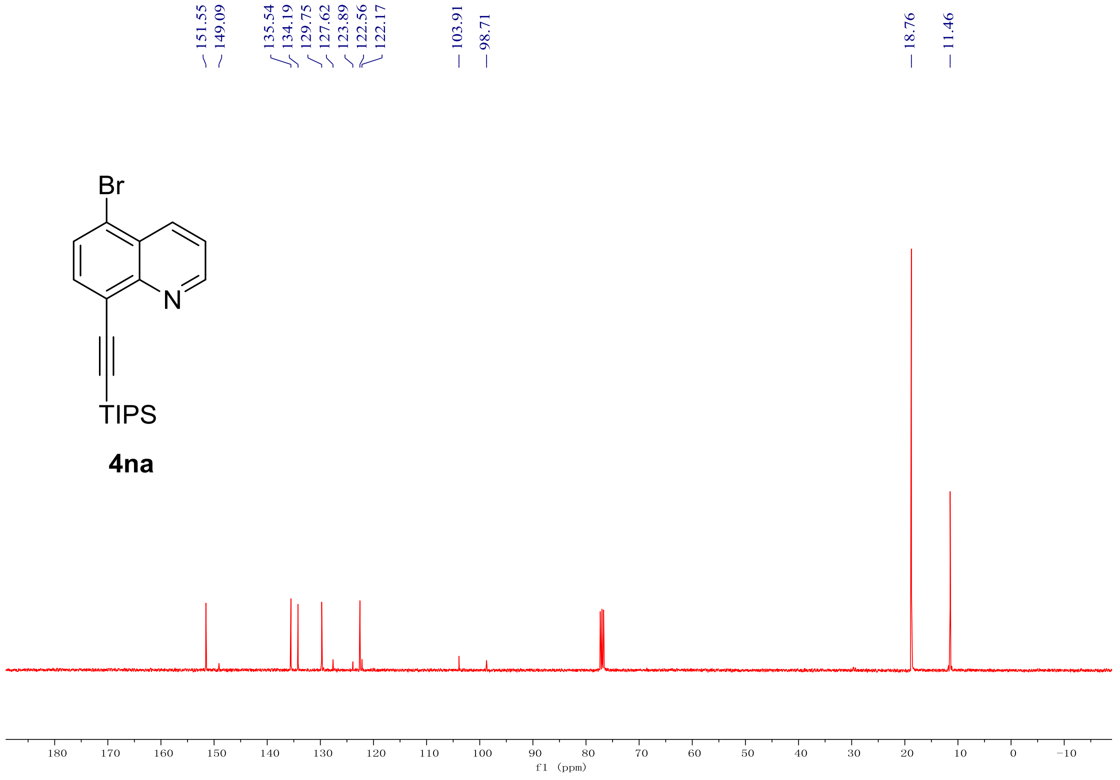

Supplementary Fig. 71. ^{13}C NMR spectra (101 MHz, $CDCl_3$, 25 °C) of **4na**

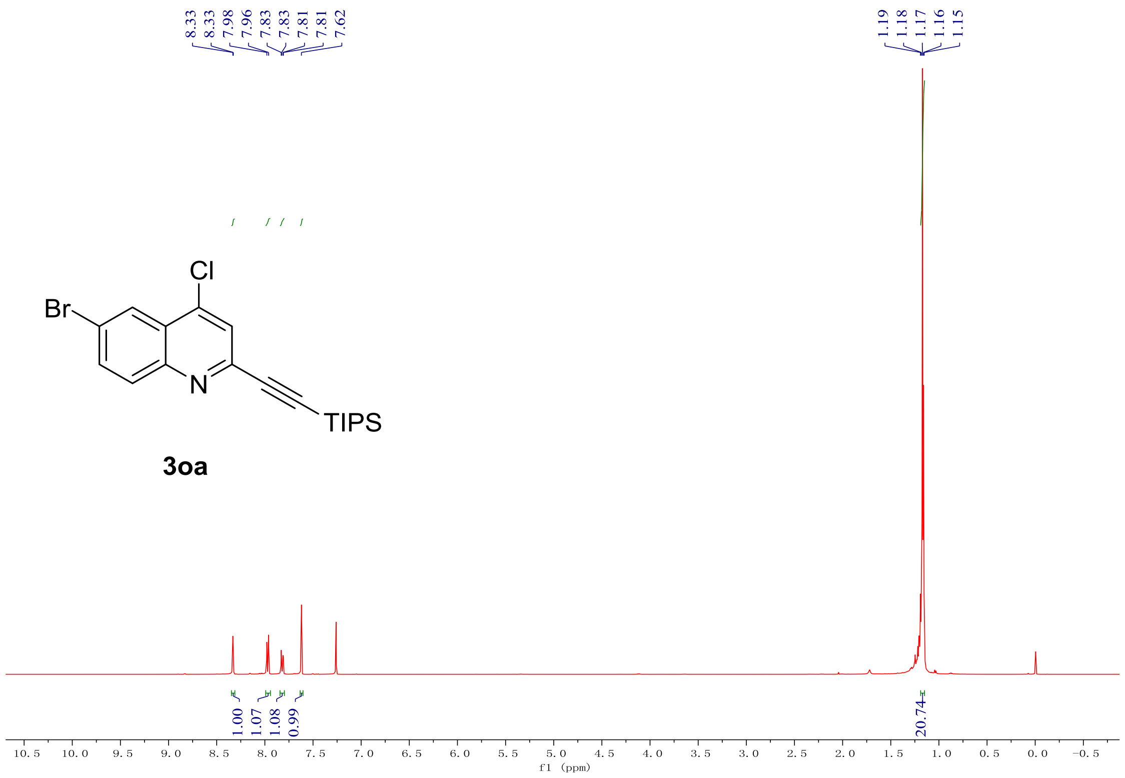

Supplementary Fig. 72. ^{1}H NMR spectra (500 MHz, $CDCl_3$, 25 °C) of **3oa**

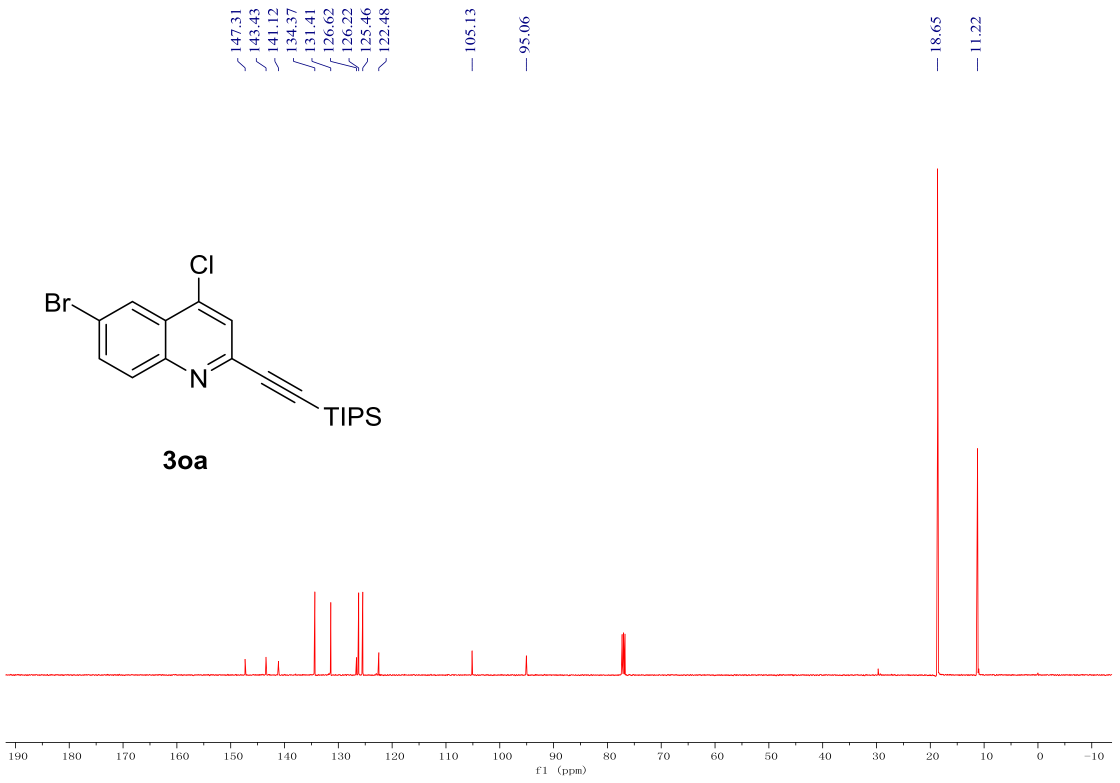

Supplementary Fig. 73. ^{13}C NMR spectra (126 MHz, $CDCl_3$, 25 °C) of **3oa**

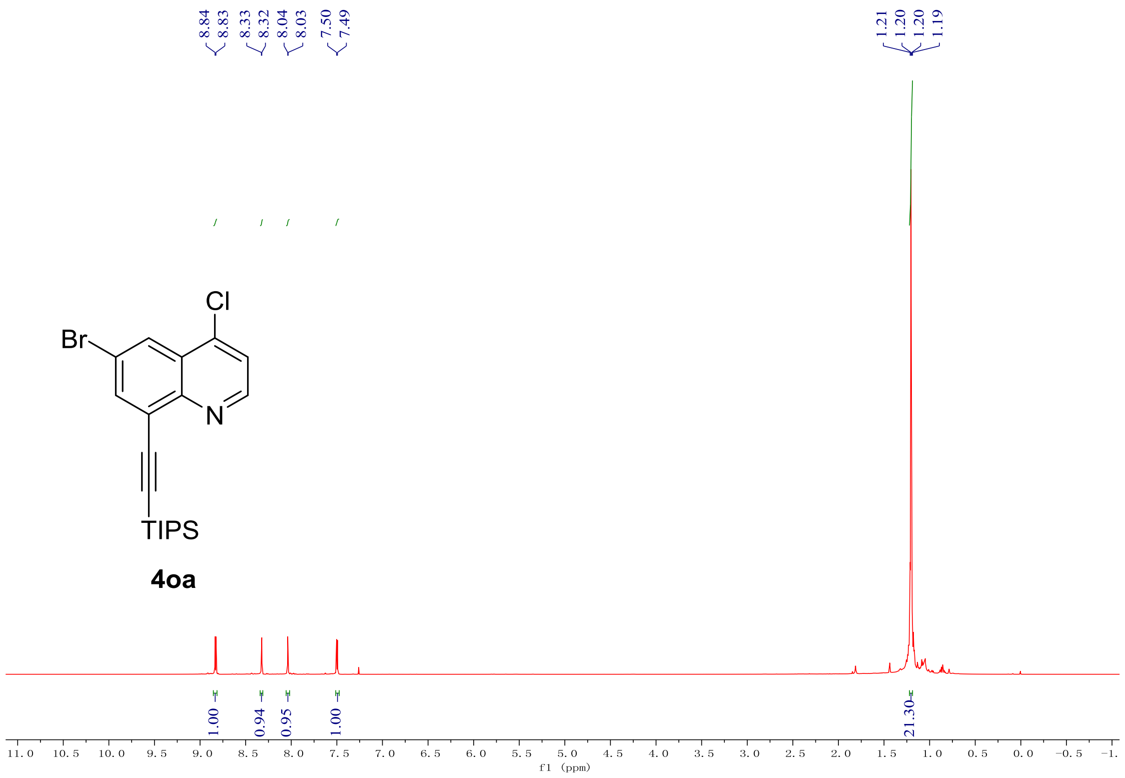

Supplementary Fig. 74. ^{1}H NMR spectra (500 MHz, $CDCl_3$, 25 °C) of **4oa**

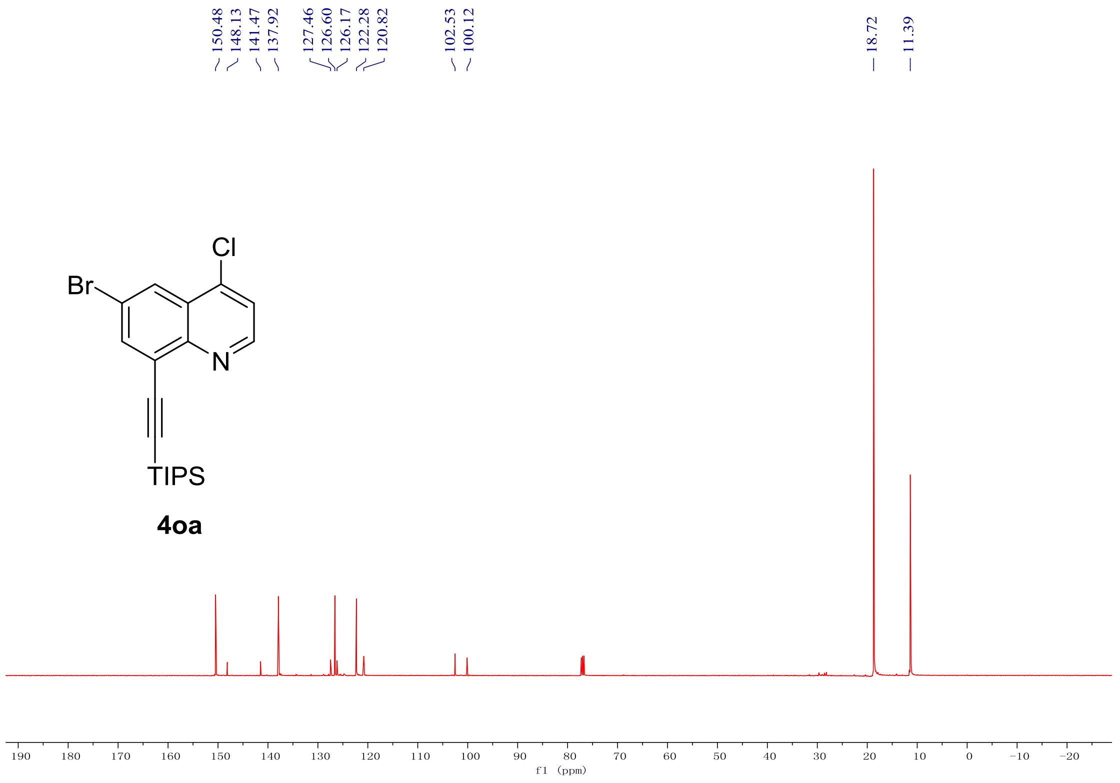

Supplementary Fig. 75. ^{13}C NMR spectra (126 MHz, $CDCl_3$, 25 °C) of **4oa**

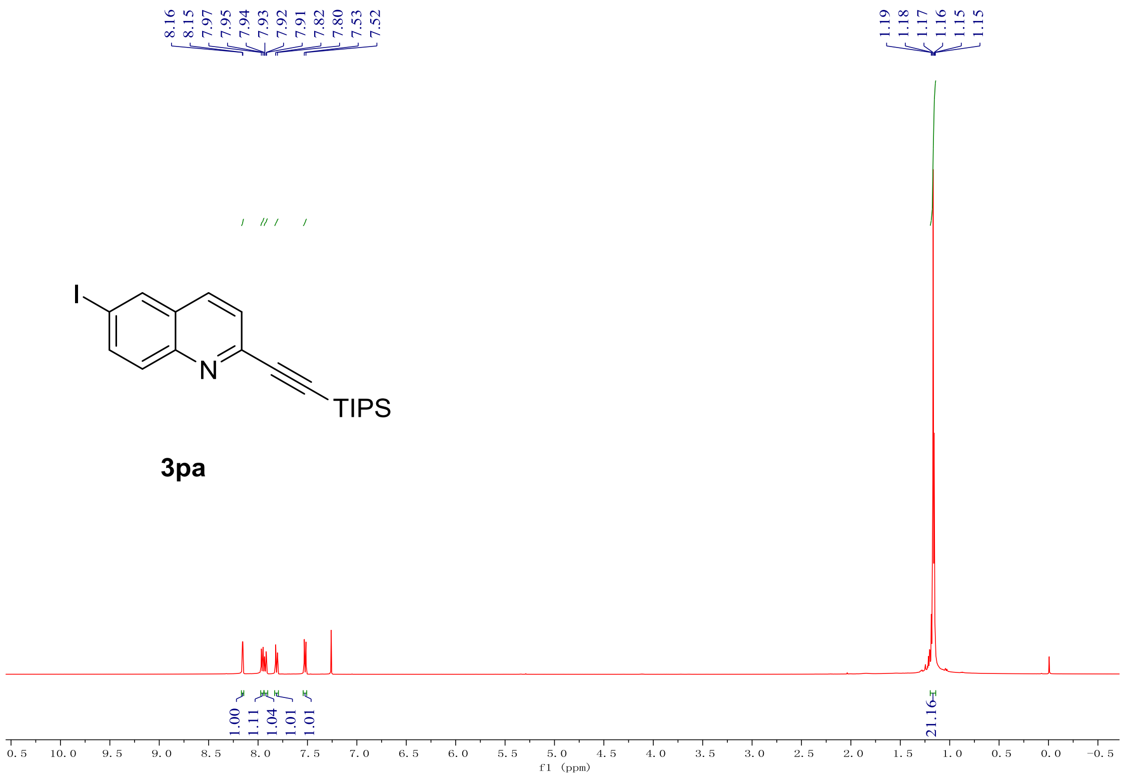

Supplementary Fig. 76. ^{1}H NMR spectra (500 MHz, $CDCl_3$, 25 °C) of **3pa**

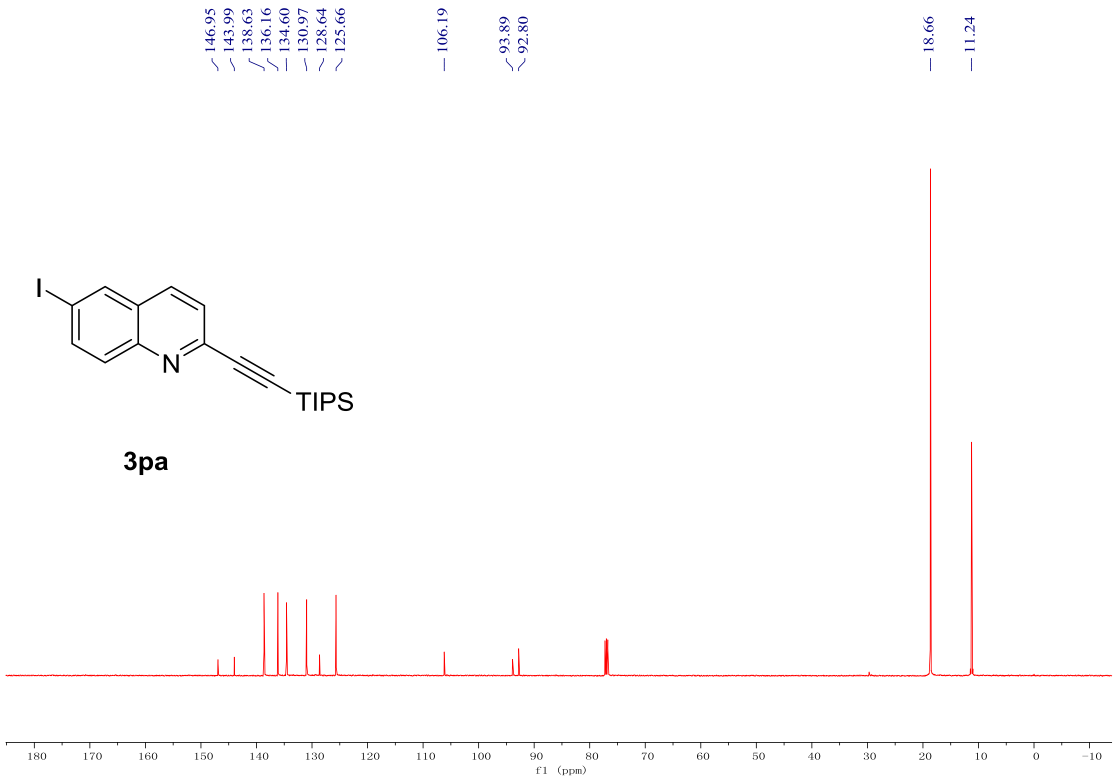

Supplementary Fig. 77. ^{13}C NMR spectra (126 MHz, $CDCl_3$, 25 °C) of **3pa**

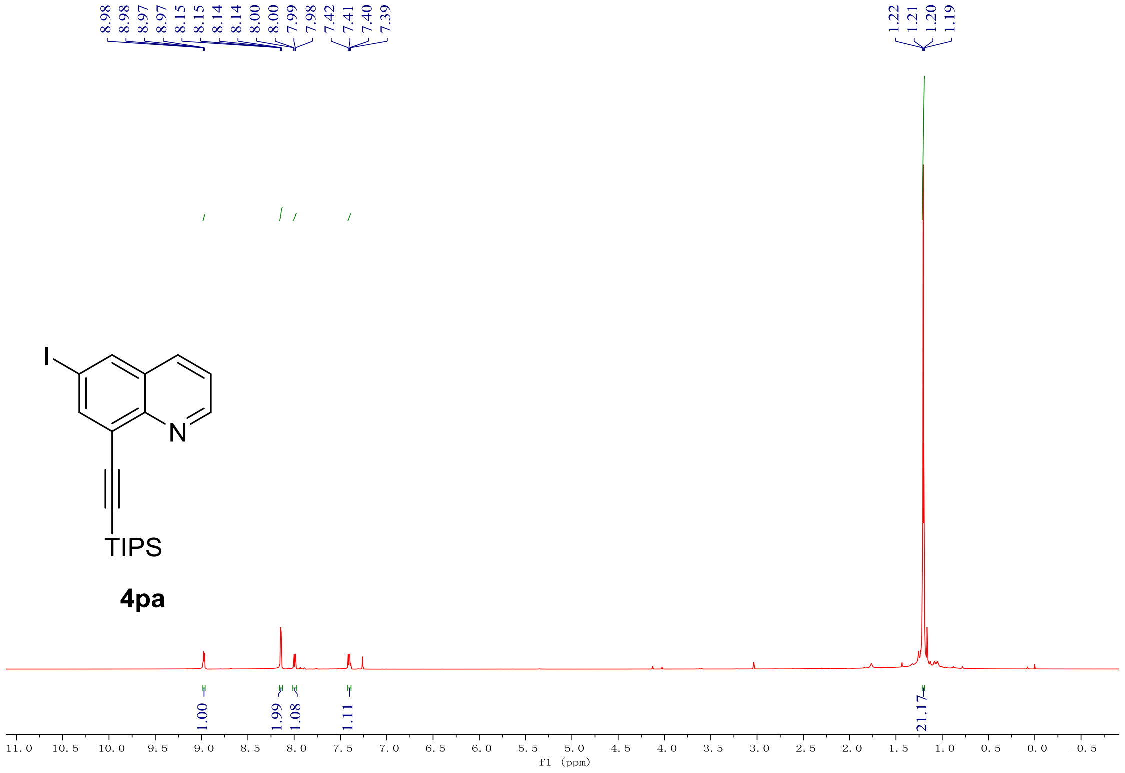

Supplementary Fig. 78. ^{1}H NMR spectra (500 MHz, $CDCl_3$, 25 ^{o}C) of **4pa**

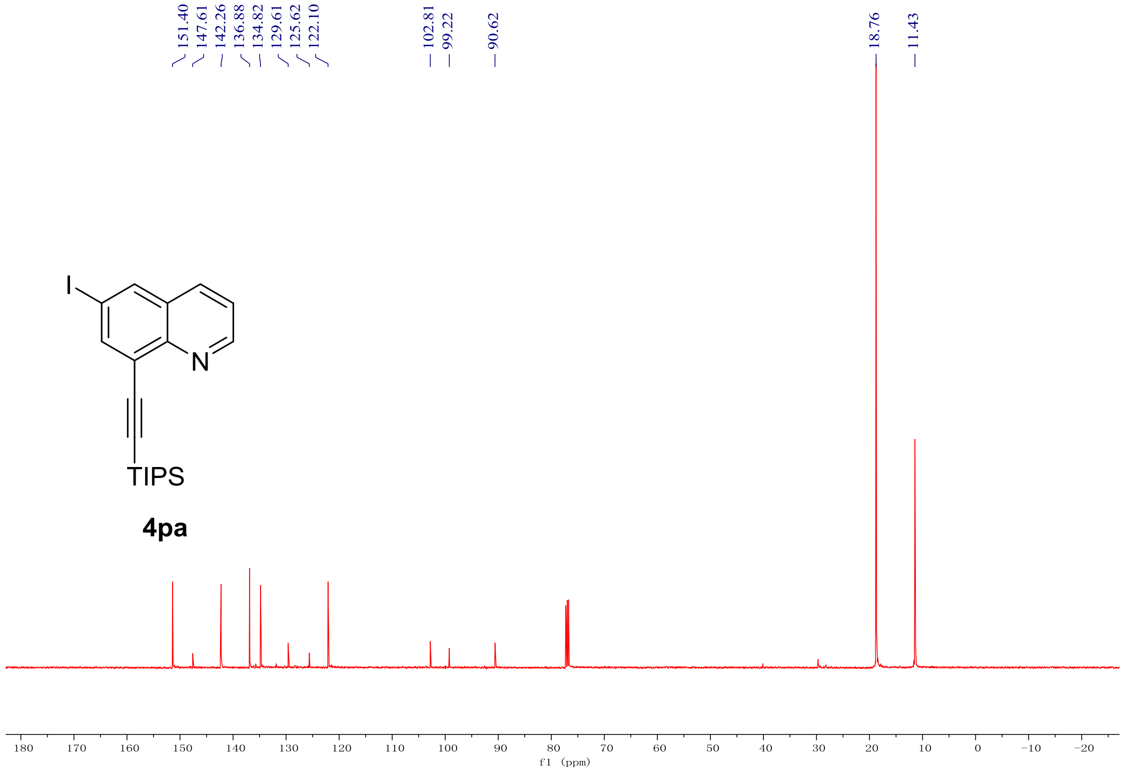

Supplementary Fig. 79. ^{13}C NMR spectra (126 MHz, $CDCl_3$, 25 ^{o}C) of **4pa**

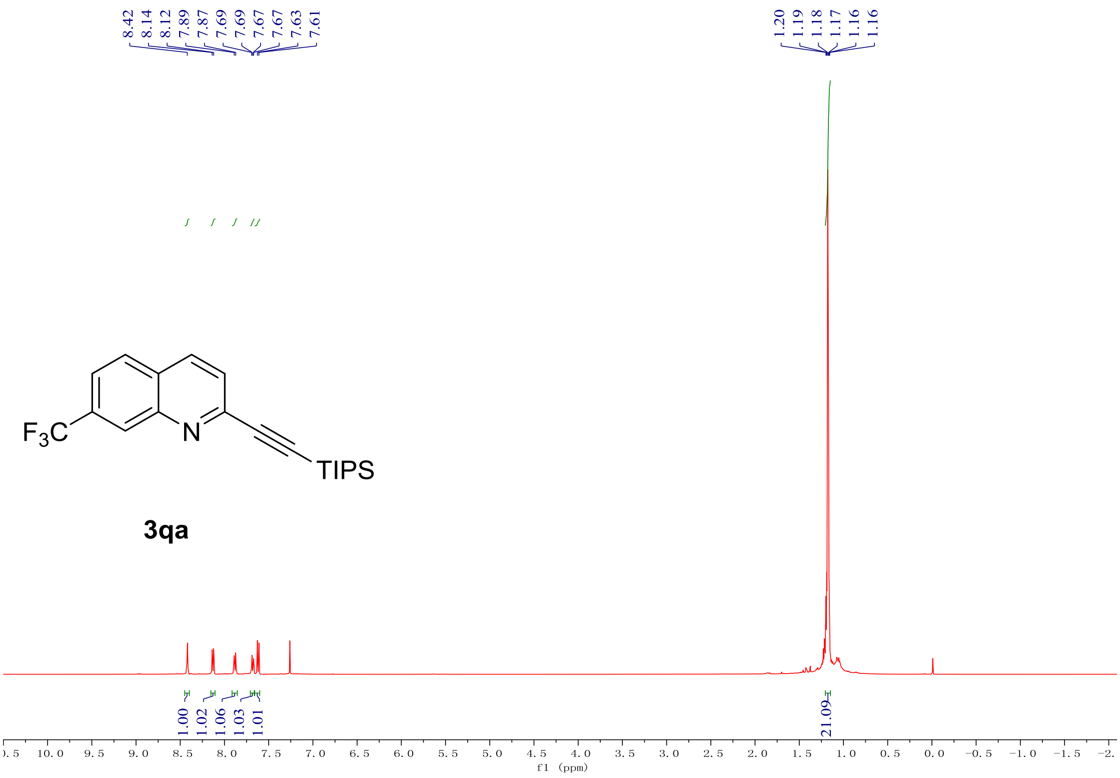

Supplementary Fig. 80. ^{1}H NMR spectra (500 MHz, $CDCl_3$, 25 °C) of **3qa**

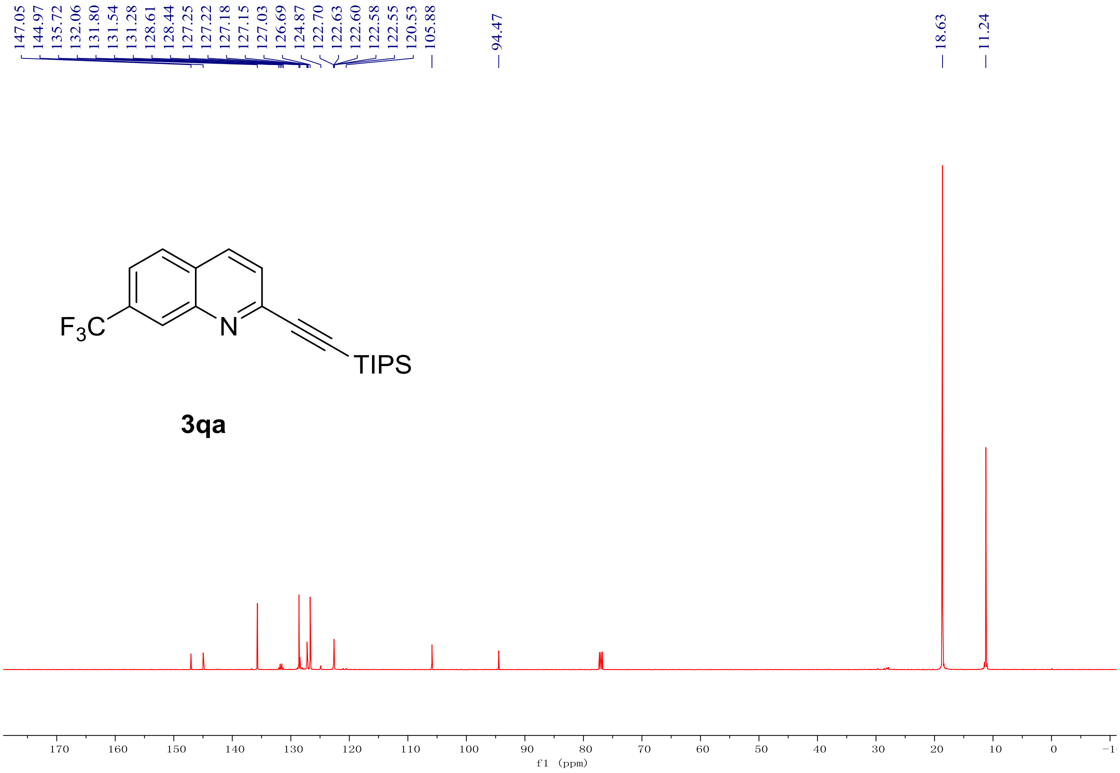

Supplementary Fig. 81. ^{13}C NMR spectra (126 MHz, $CDCl_3$, 25 °C) of **3qa**

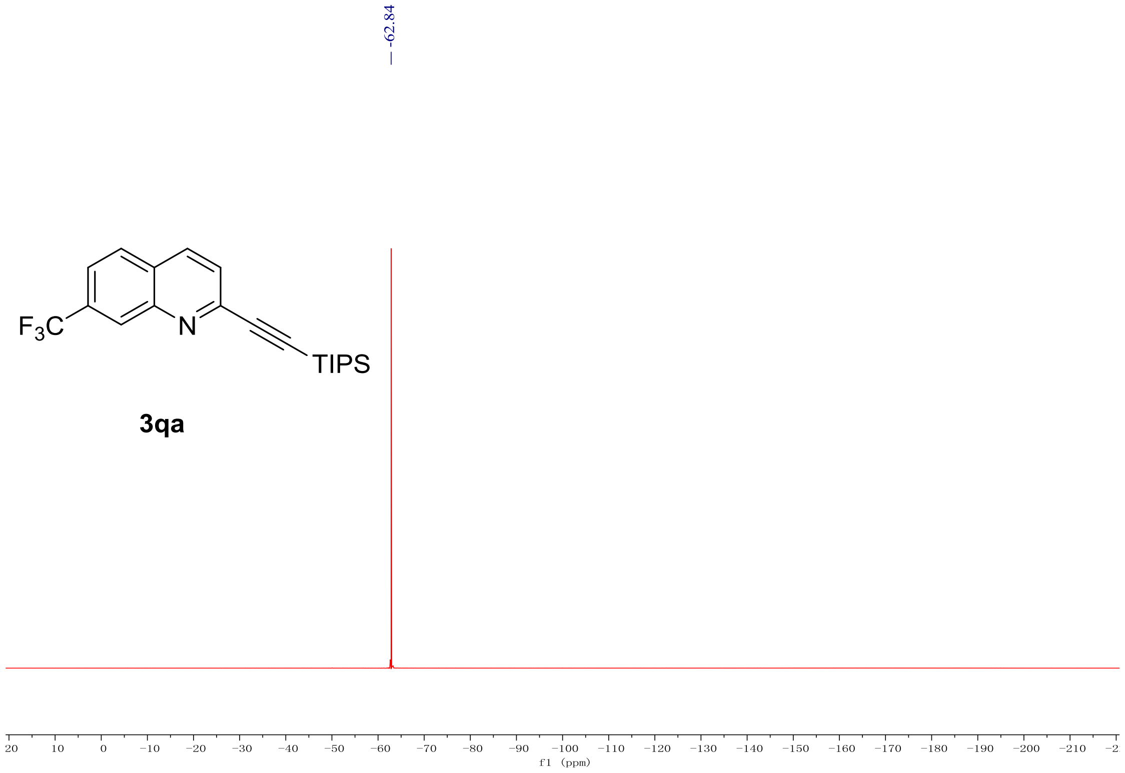

Supplementary Fig. 82. ^{19}F NMR spectra (471 MHz, $CDCl_3$, 25 °C) of **3qa**

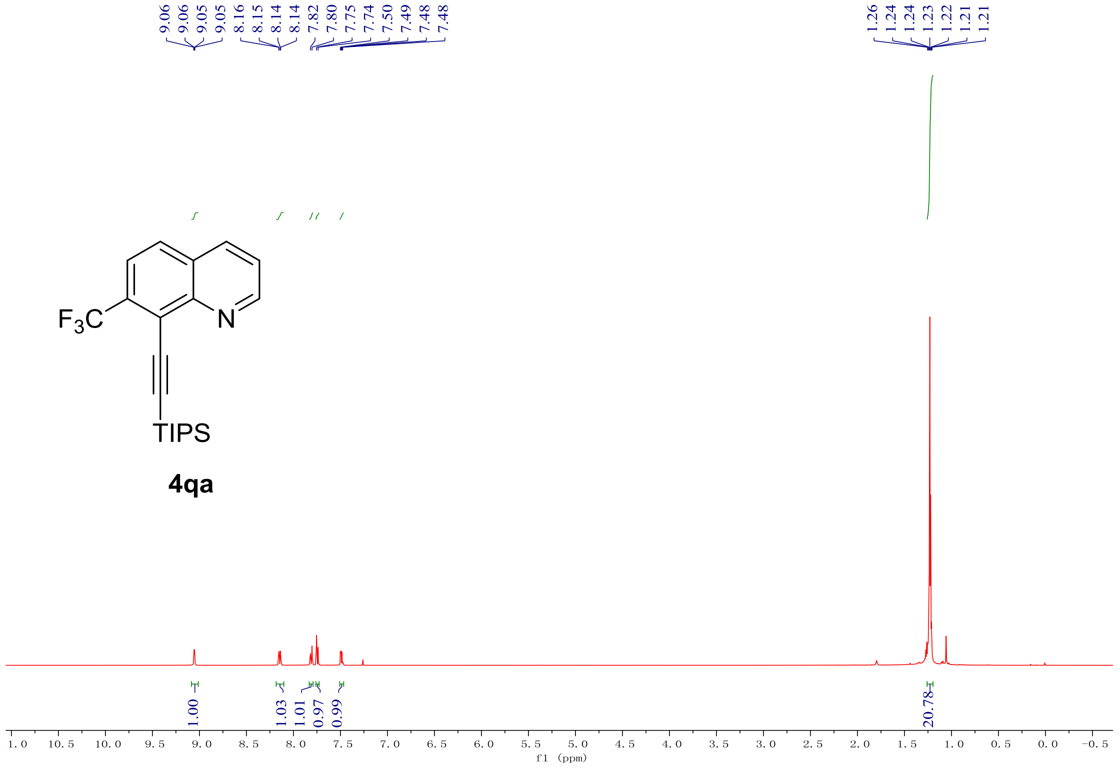

Supplementary Fig. 83. ^{1}H NMR spectra (500 MHz, $CDCl_3$, 25 °C) of **4qa**

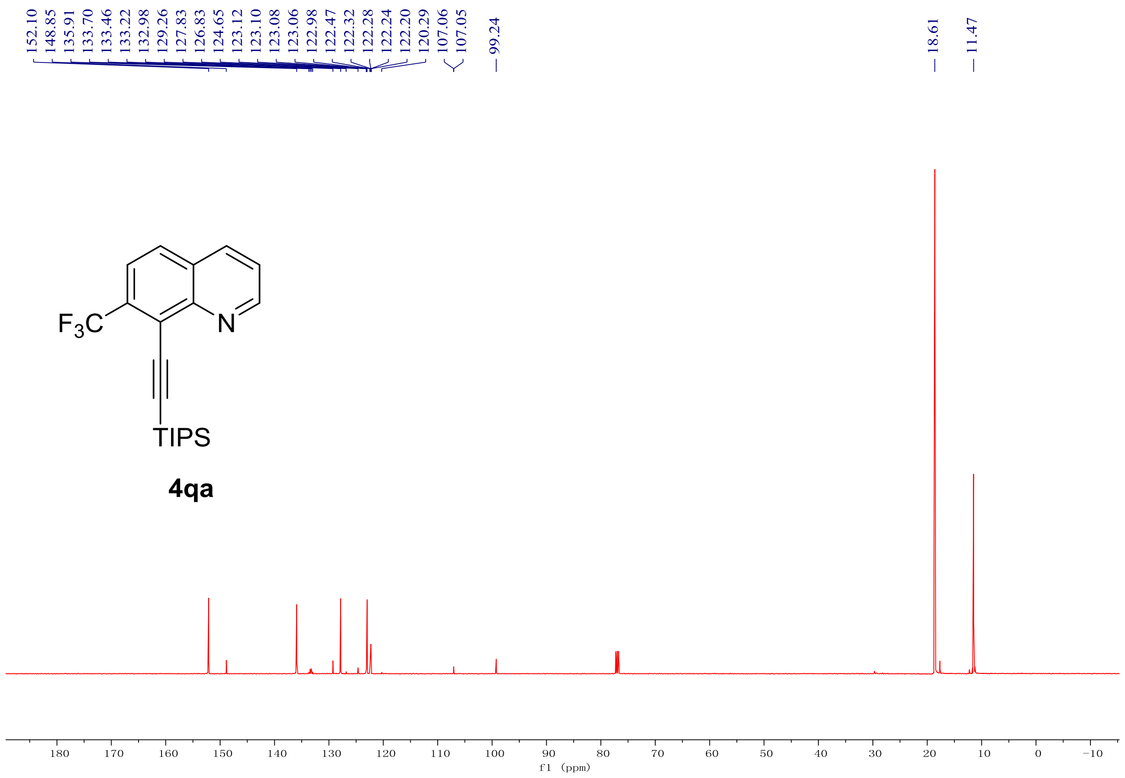

Supplementary Fig. 84. ^{13}C NMR spectra (126 MHz, $CDCl_3$, 25 °C) of **4qa**

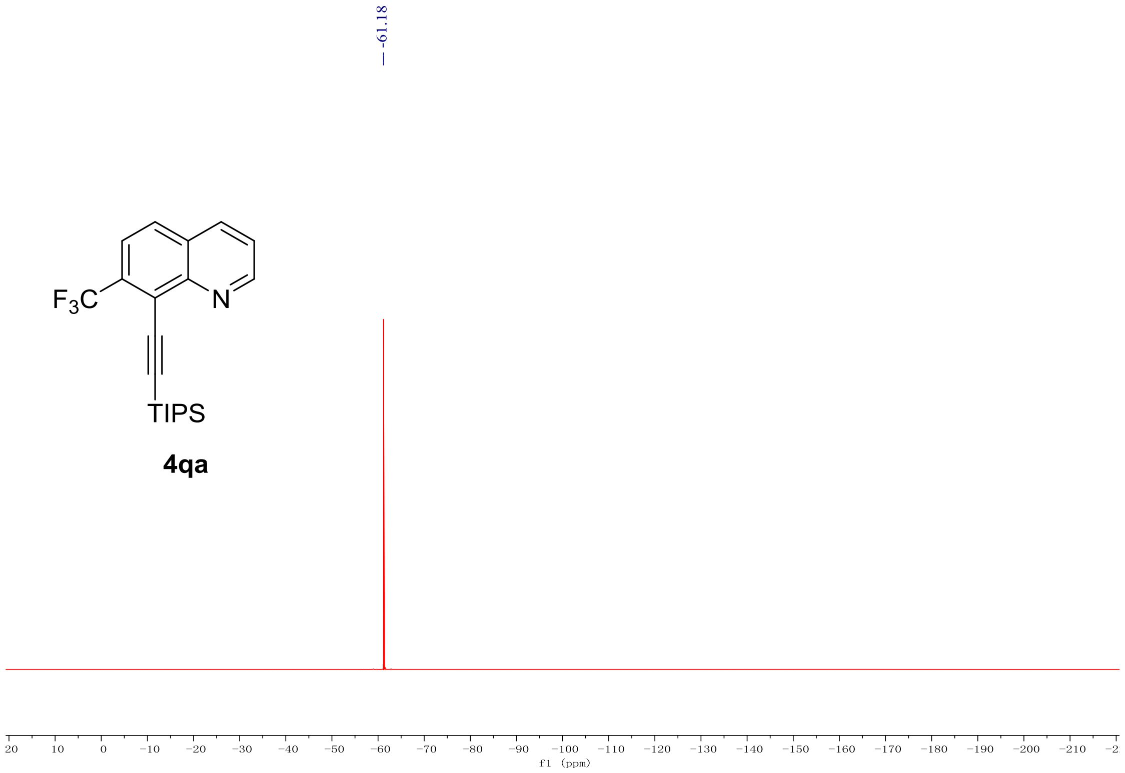

Supplementary Fig. 85. ^{19}F NMR spectra (471 MHz, $CDCl_3$, 25 °C) of **4qa**

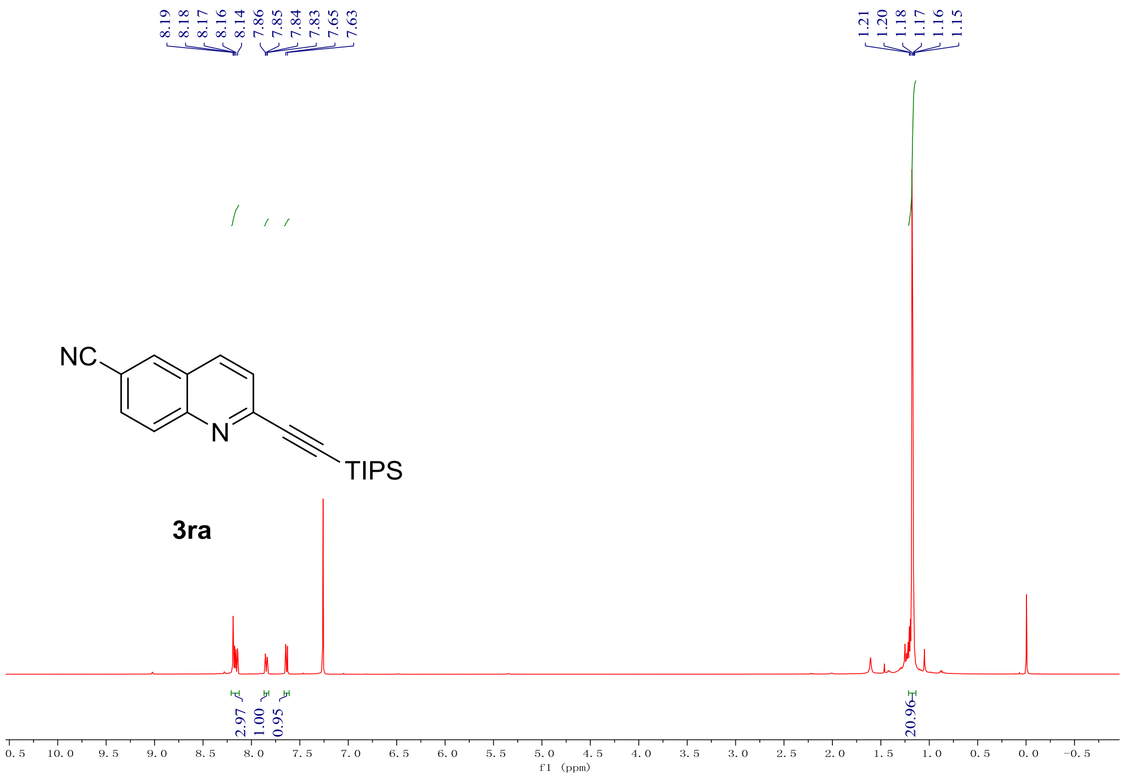

Supplementary Fig. 86. ^{1}H NMR spectra (500 MHz, $CDCl_3$, 25 °C) of **3ra**

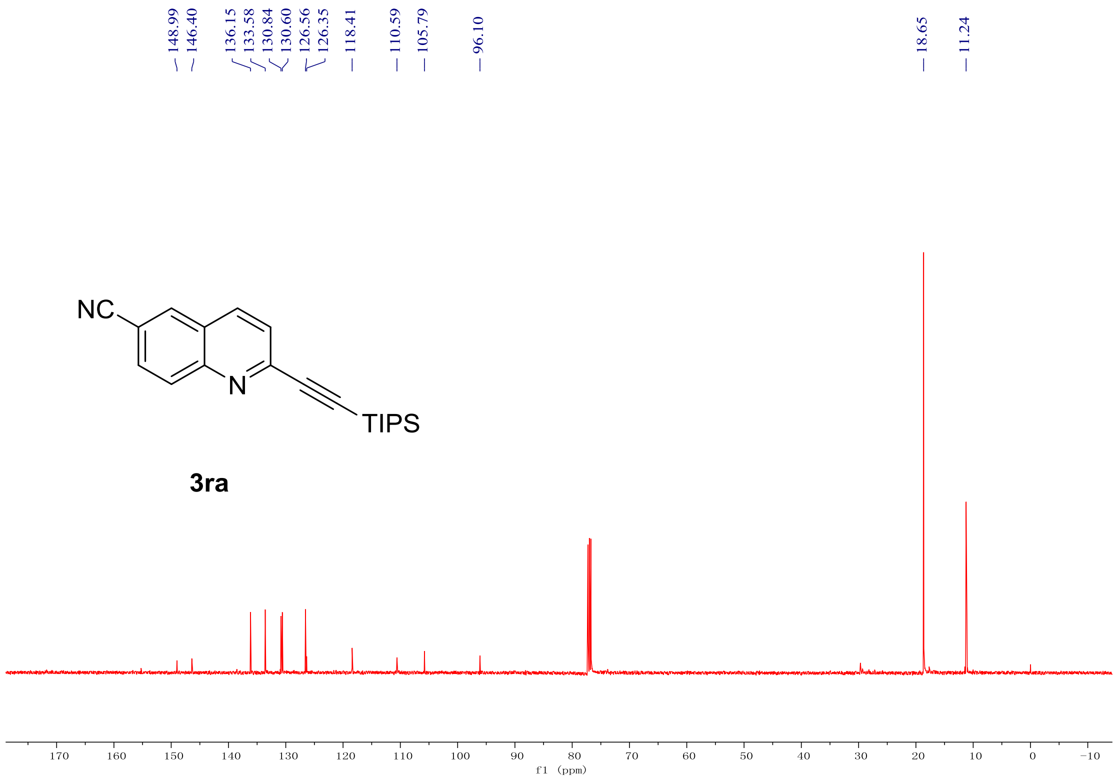

Supplementary Fig. 87. ^{13}C NMR spectra (126 MHz, $CDCl_3$, 25 °C) of **3ra**

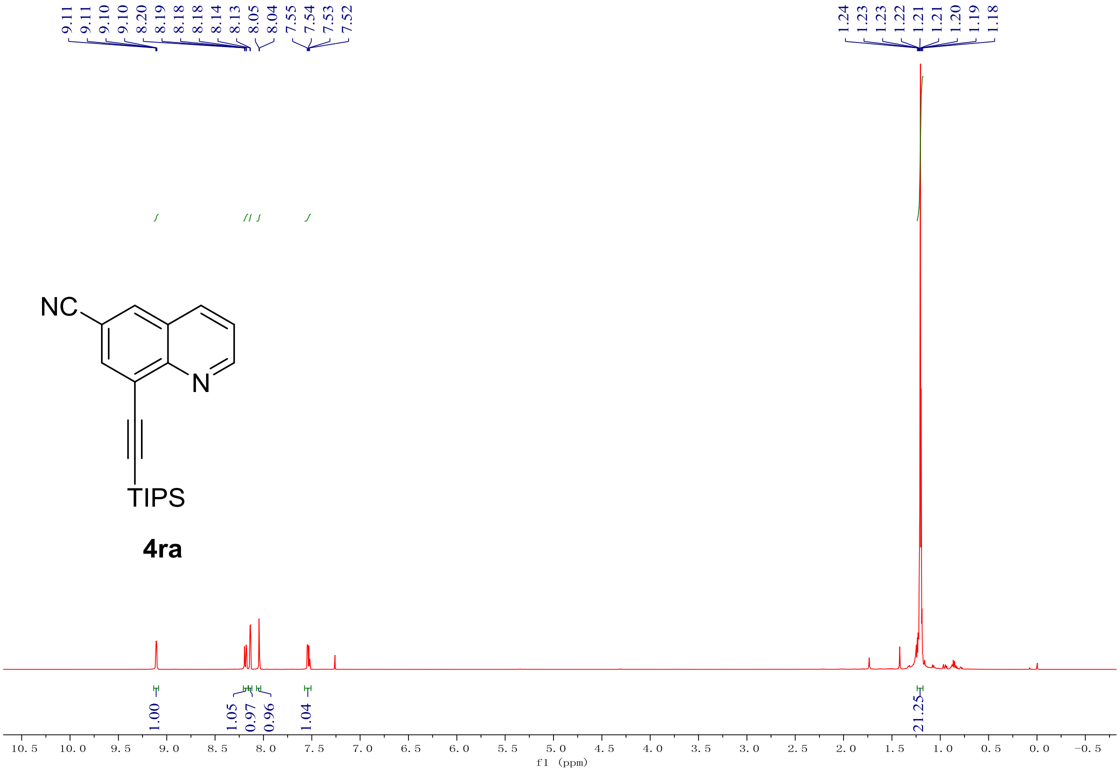

Supplementary Fig. 88. ^{1}H NMR spectra (500 MHz, $CDCl_3$, 25 ^{o}C) of **4ra**

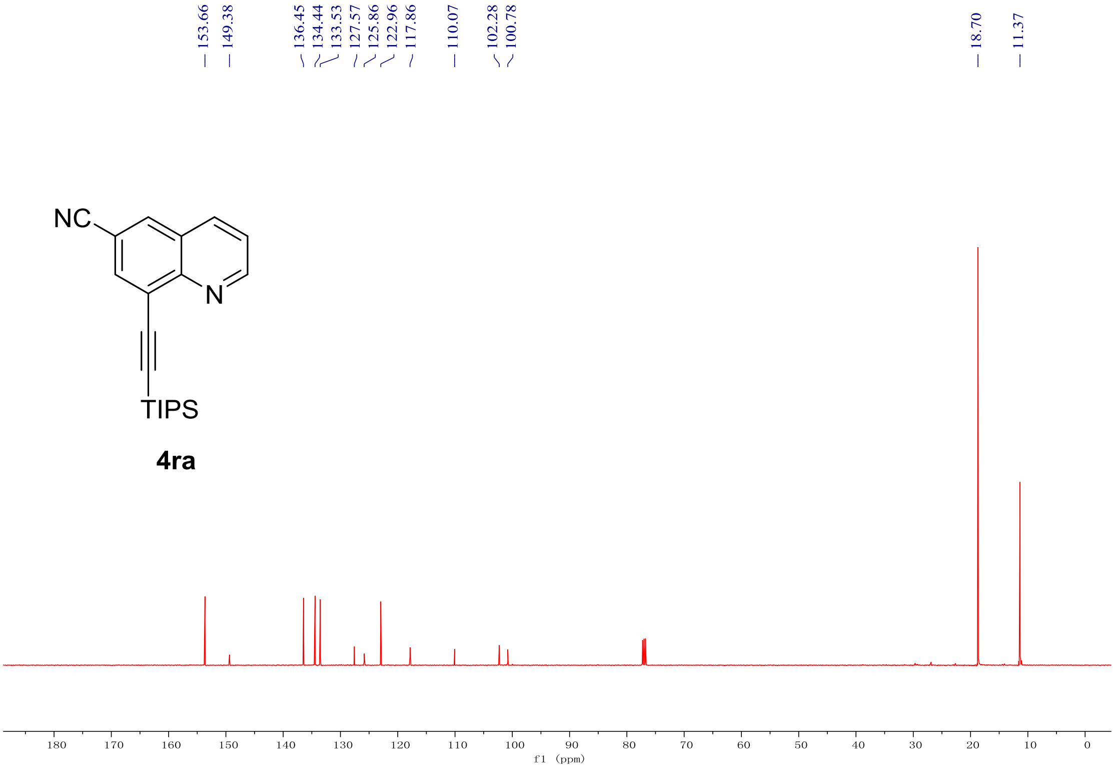

Supplementary Fig. 89. ^{13}C NMR spectra (126 MHz, $CDCl_3$, 25 ^{o}C) of **4ra**

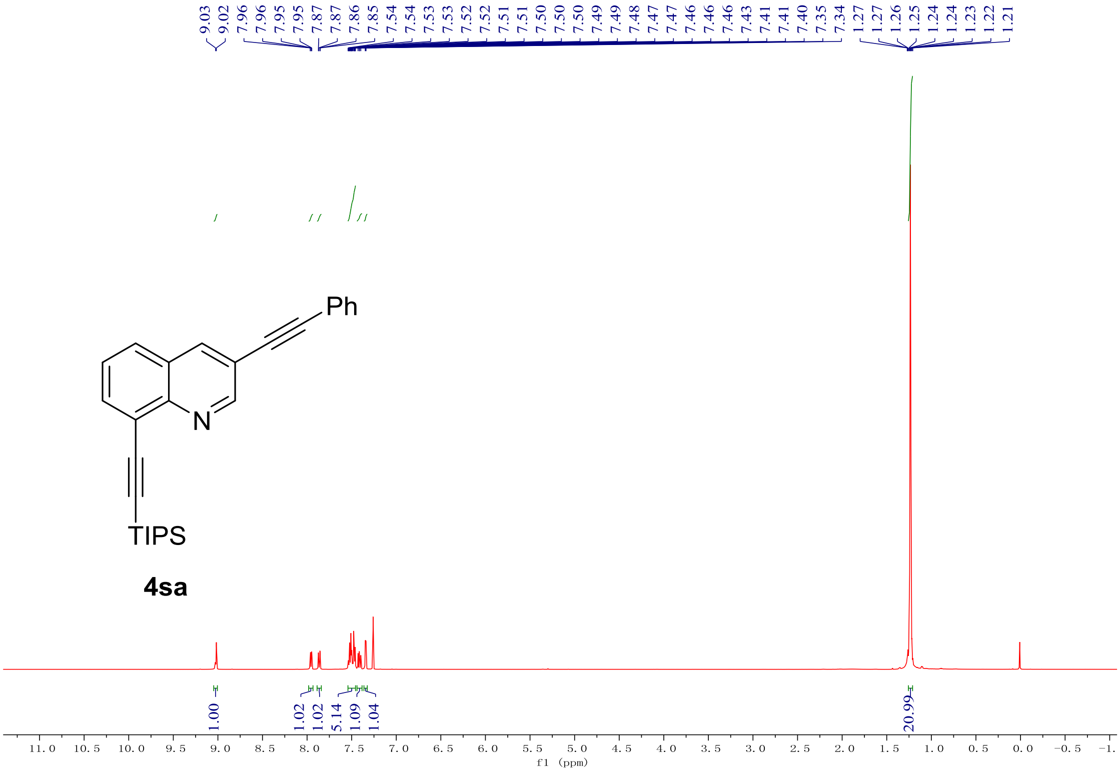

Supplementary Fig. 90. ^{1}H NMR spectra (500 MHz, $CDCl_3$, 25 ^{o}C) of **4sa**

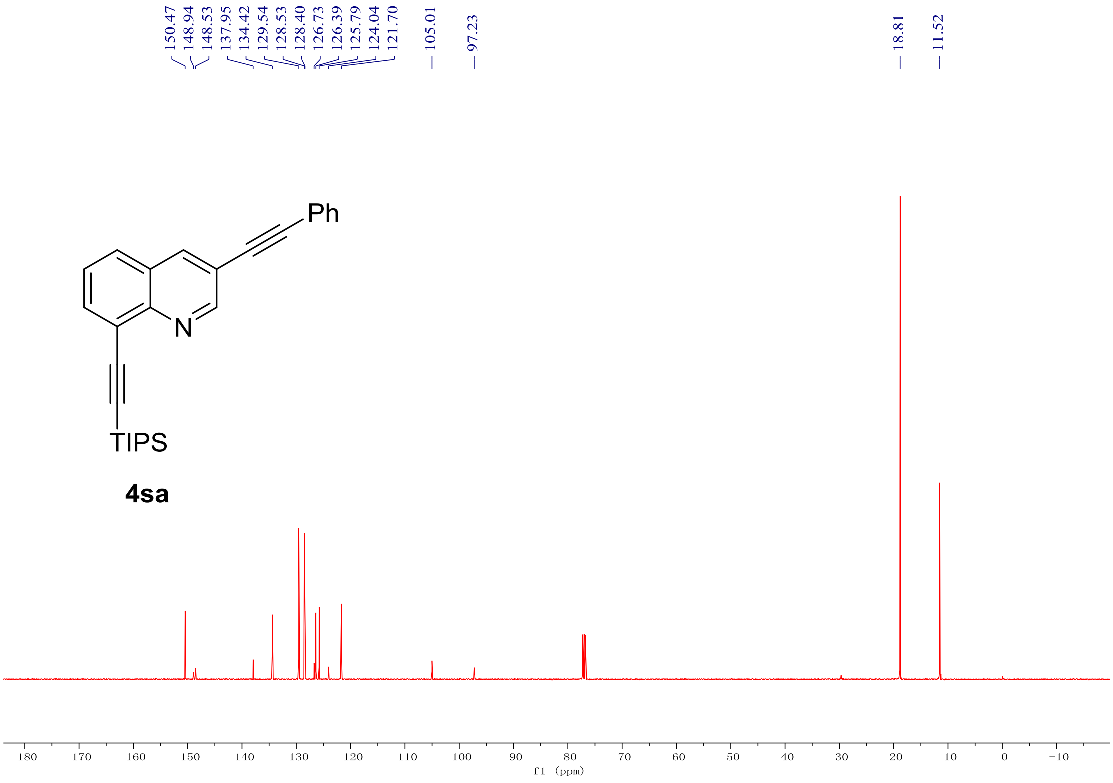

Supplementary Fig. 91. ^{13}C NMR spectra (126 MHz, $CDCl_3$, 25 ^{o}C) of **4sa**

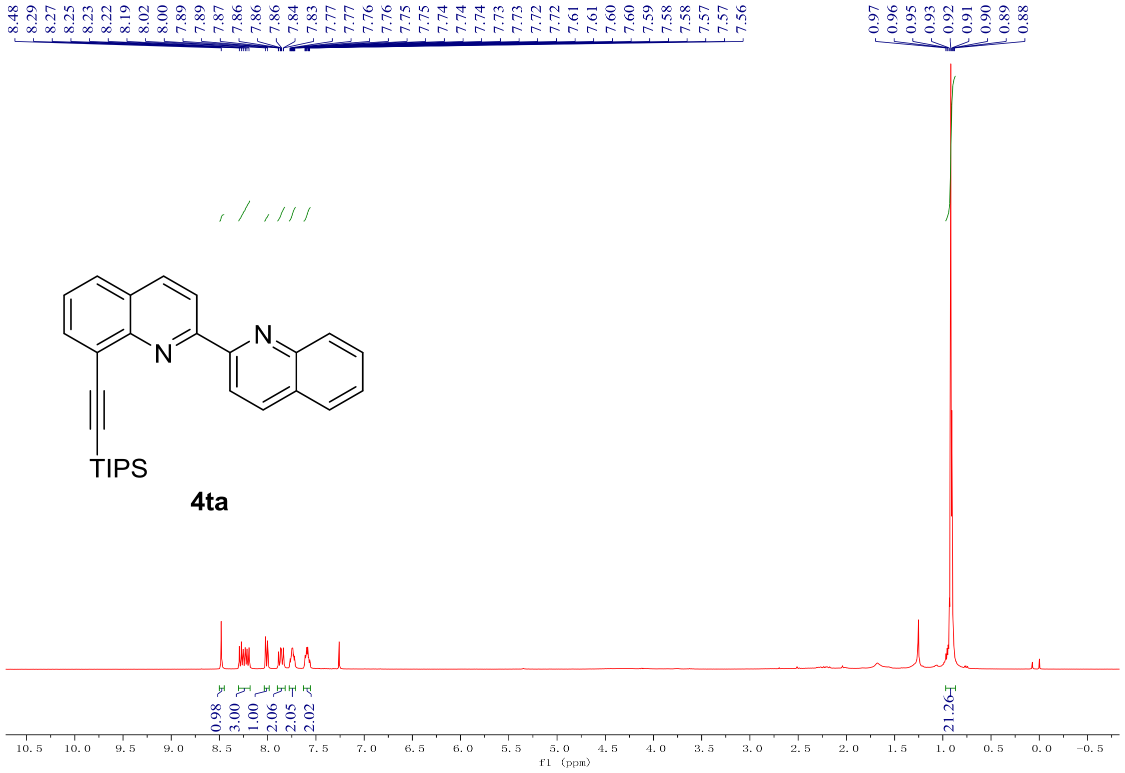

Supplementary Fig. 92. ^{1}H NMR spectra (400 MHz, $CDCl_3$, 25 °C) of **4ta**

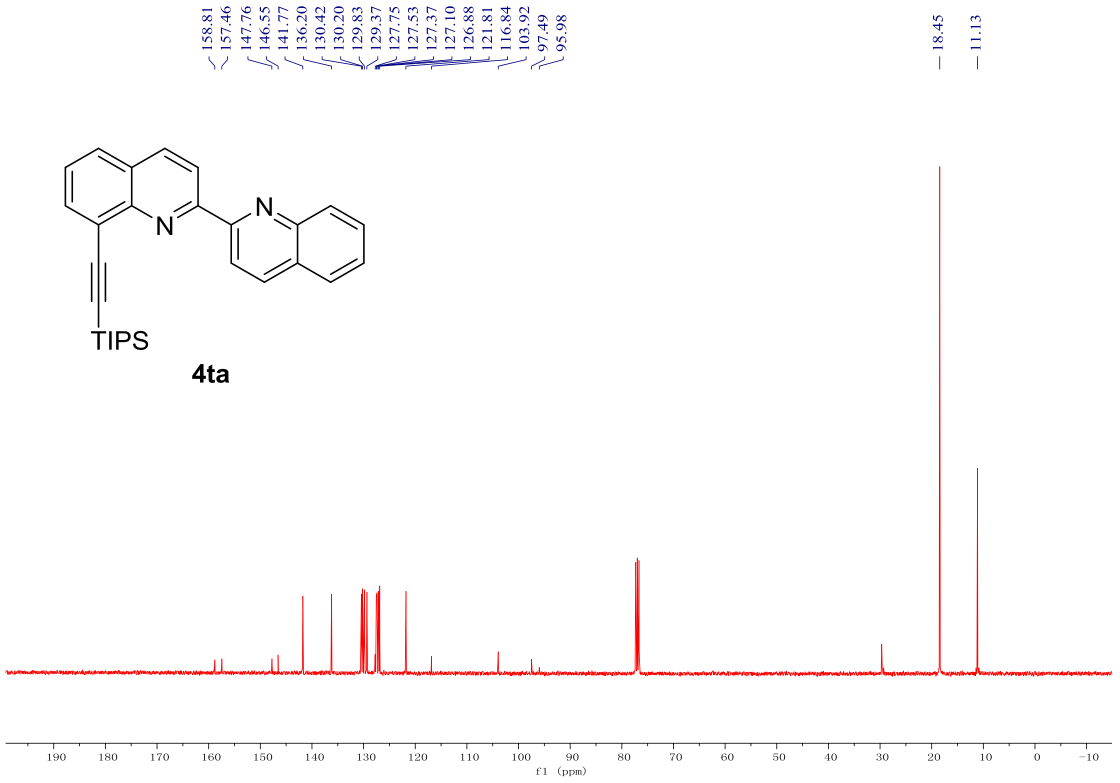

Supplementary Fig. 93. ^{13}C NMR spectra (101 MHz, $CDCl_3$, 25 °C) of **4ta**

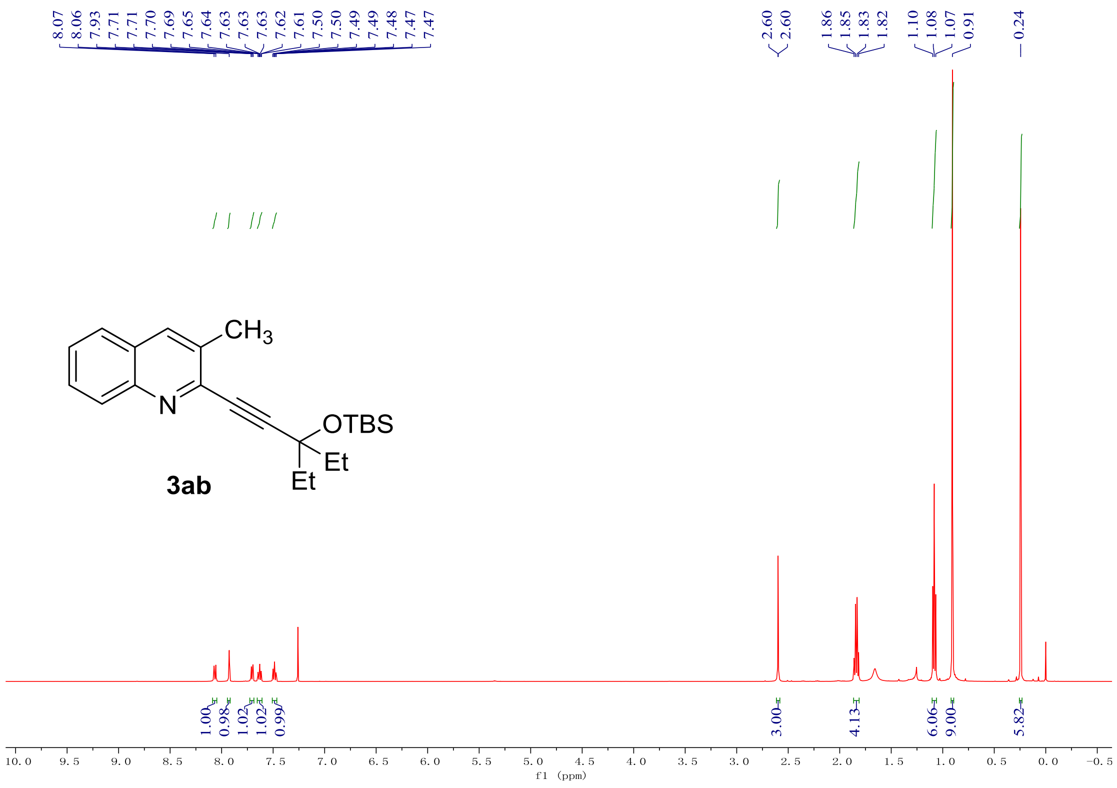

Supplementary Fig. 94. ^{1}H NMR spectra (500 MHz, $CDCl_3$, 25 ^{o}C) of **3ab**

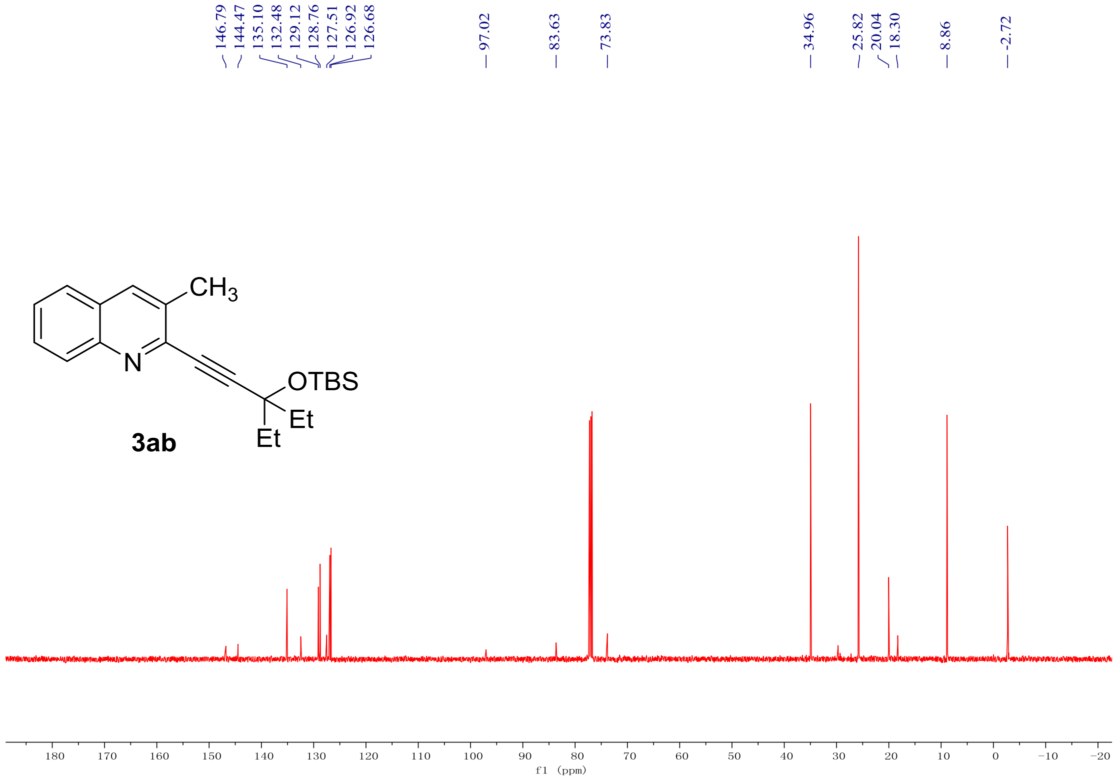

Supplementary Fig. 95. ^{13}C NMR spectra (126 MHz, $CDCl_3$, 25 ^{o}C) of **3ab**

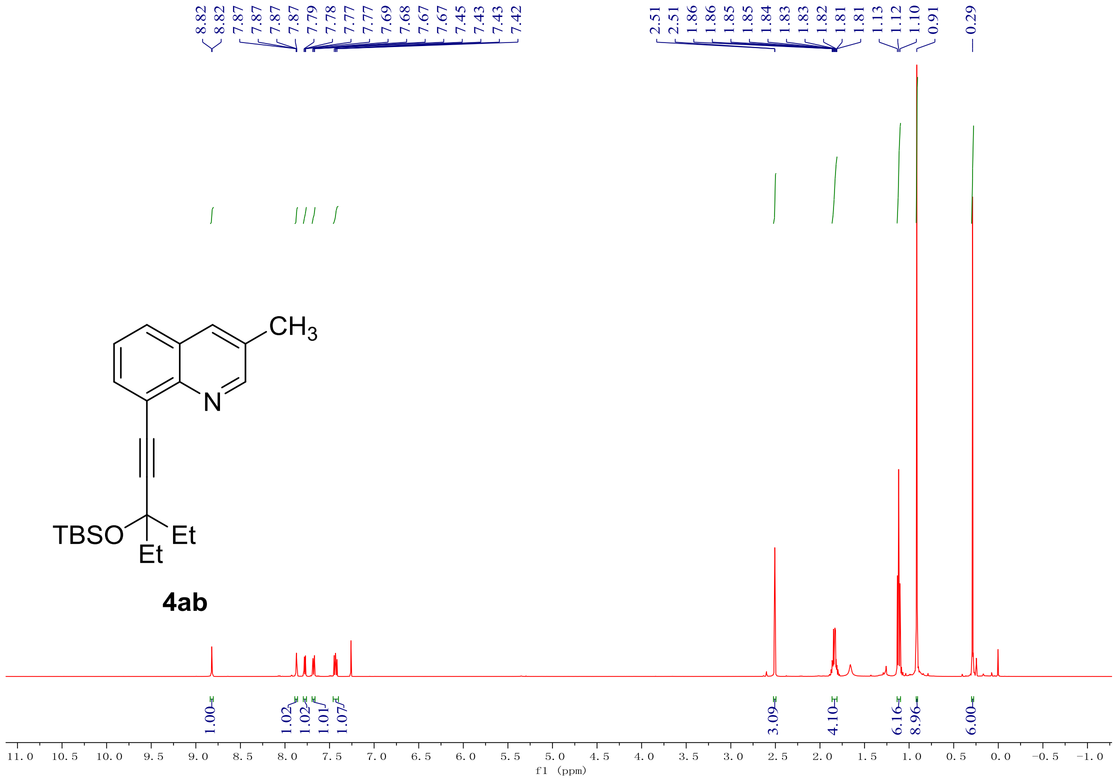

Supplementary Fig. 96. ^{1}H NMR spectra (500 MHz, $CDCl_3$, 25 °C) of **4ab**

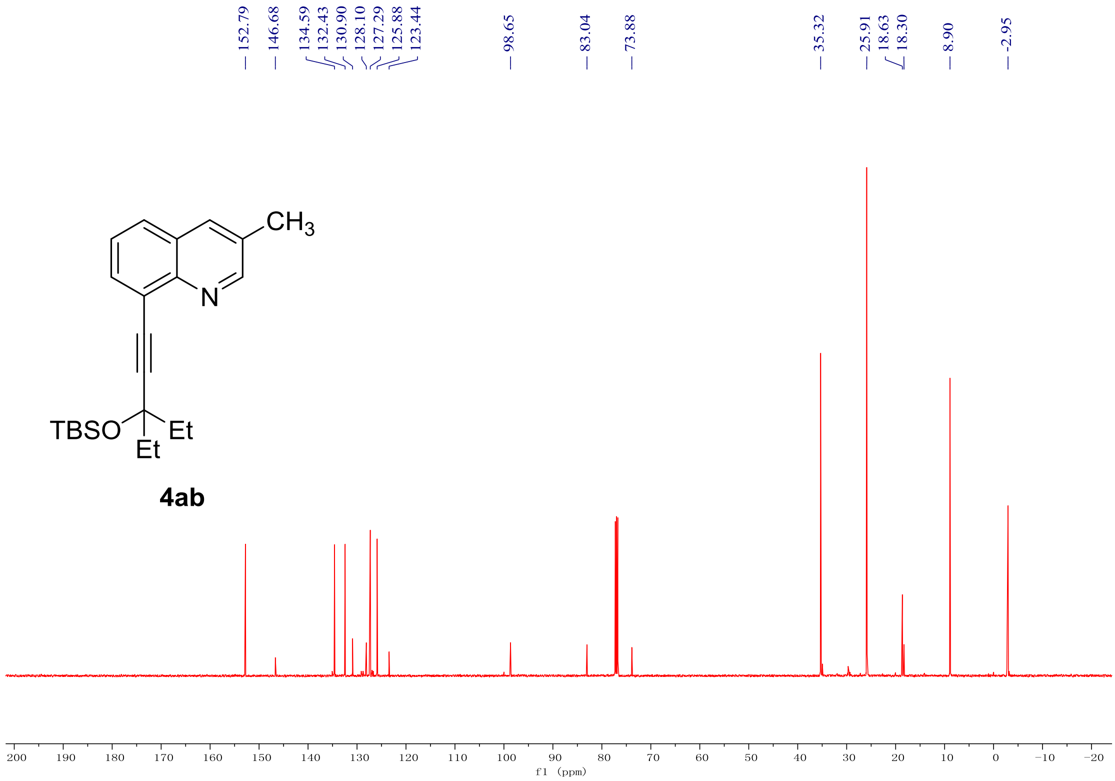

Supplementary Fig. 97. ^{13}C NMR spectra (126 MHz, $CDCl_3$, 25 °C) of **4ab**

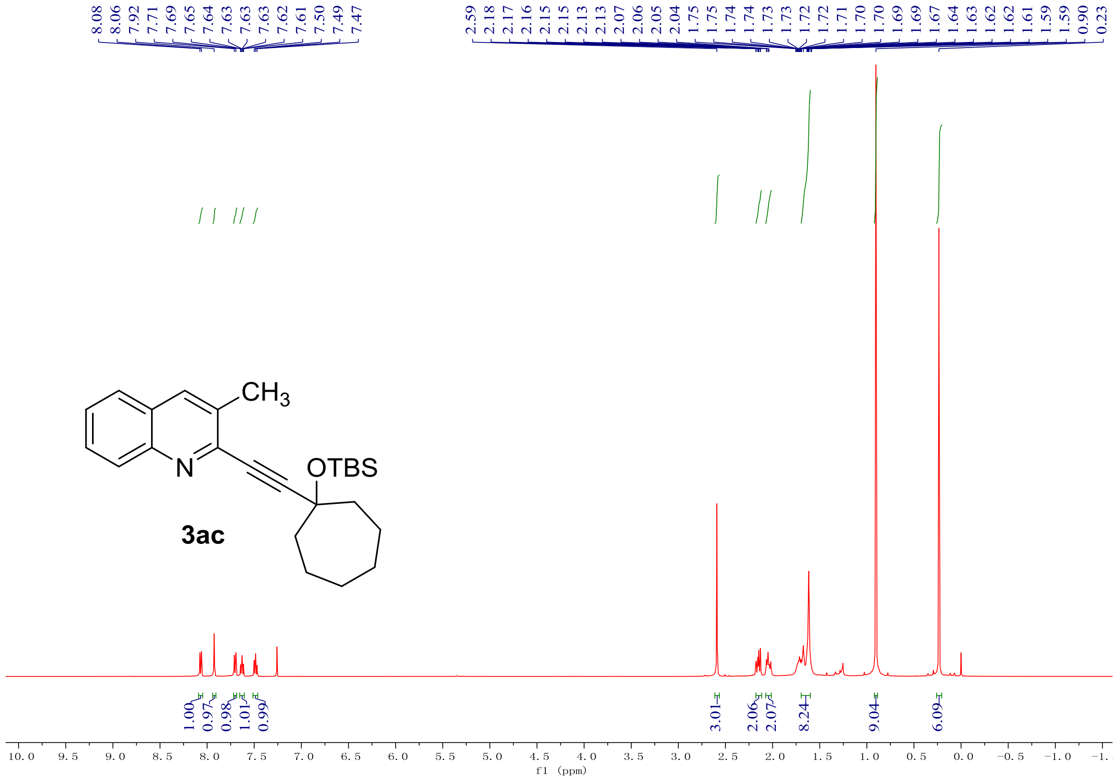

Supplementary Fig. 98. ^{1}H NMR spectra (500 MHz, $CDCl_3$, 25 ^{o}C) of **3ac**

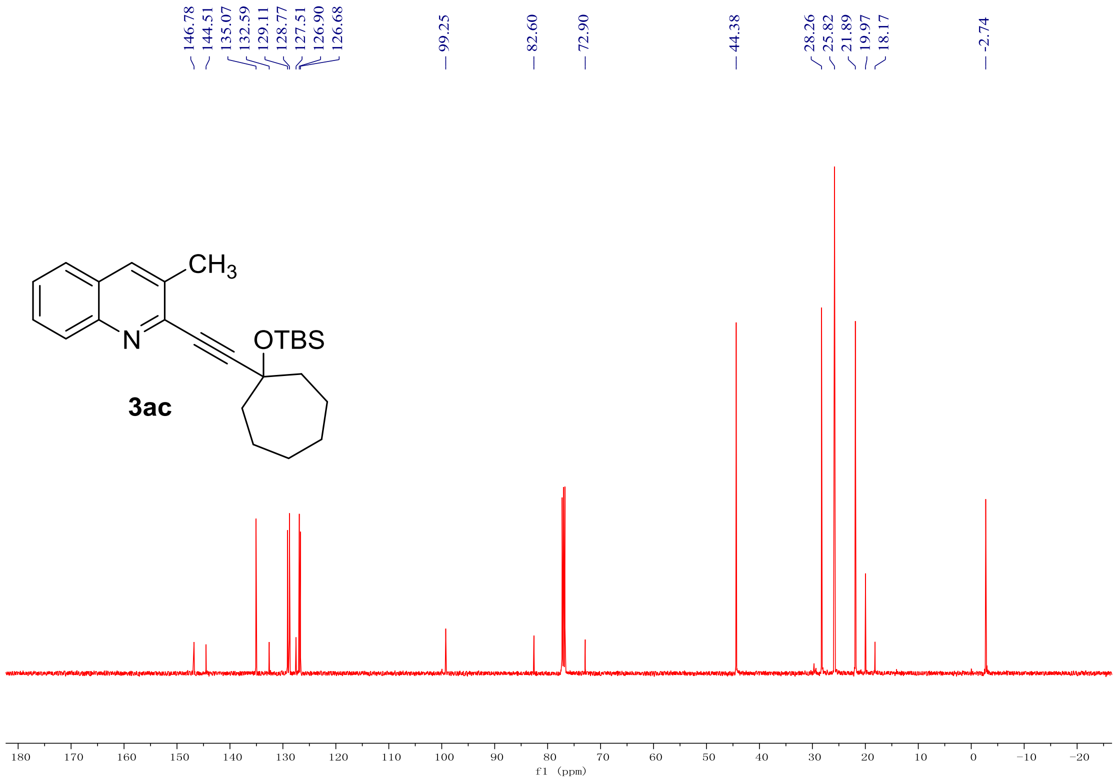

Supplementary Fig. 99. ^{13}C NMR spectra (126 MHz, $CDCl_3$, 25 ^{o}C) of **3ac**

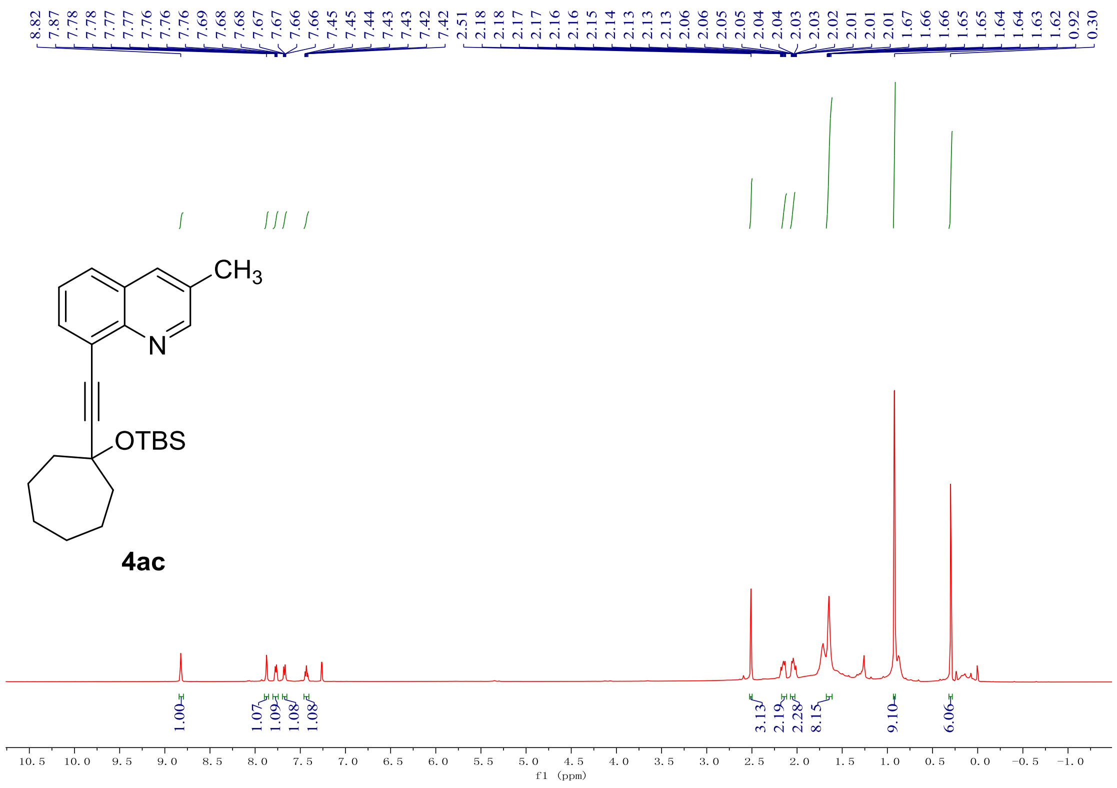

Supplementary Fig. 100. ^{1}H NMR spectra (500 MHz, $CDCl_3$, 25 °C) of **4ac**

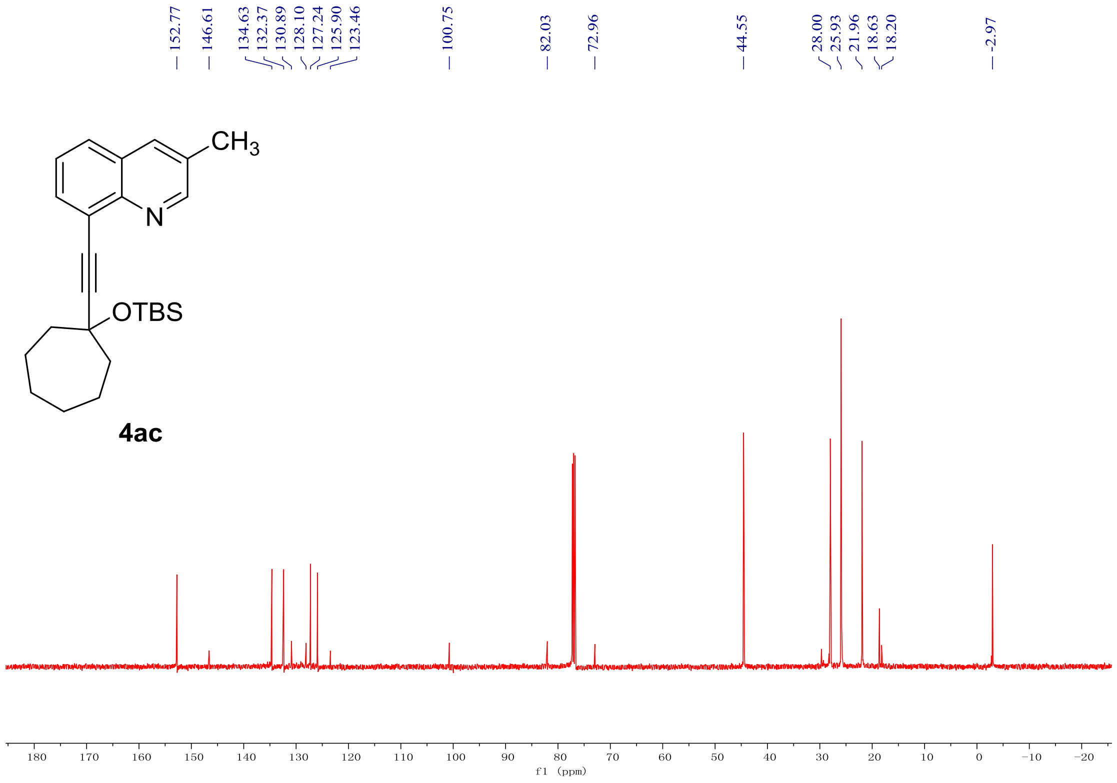

Supplementary Fig. 101. ^{13}C NMR spectra (126 MHz, $CDCl_3$, 25 °C) of **4ac**

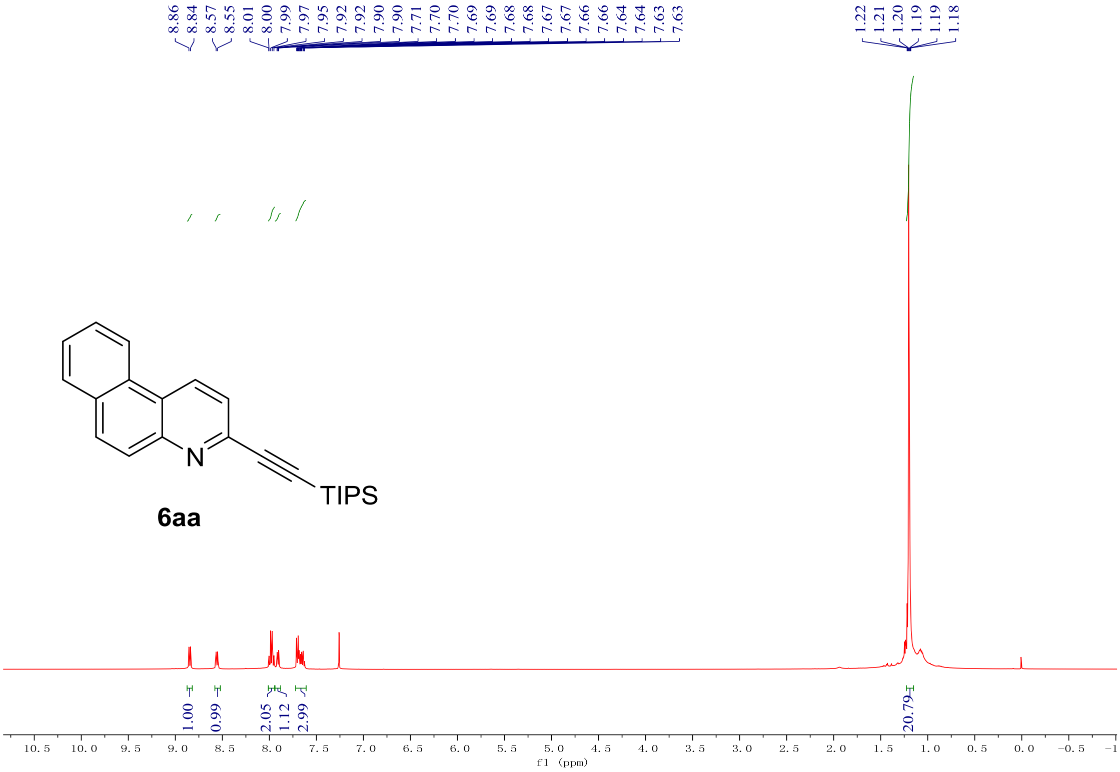

Supplementary Fig. 102. ^{1}H NMR spectra (500 MHz, $CDCl_3$, 25 ^{o}C) of **6aa**

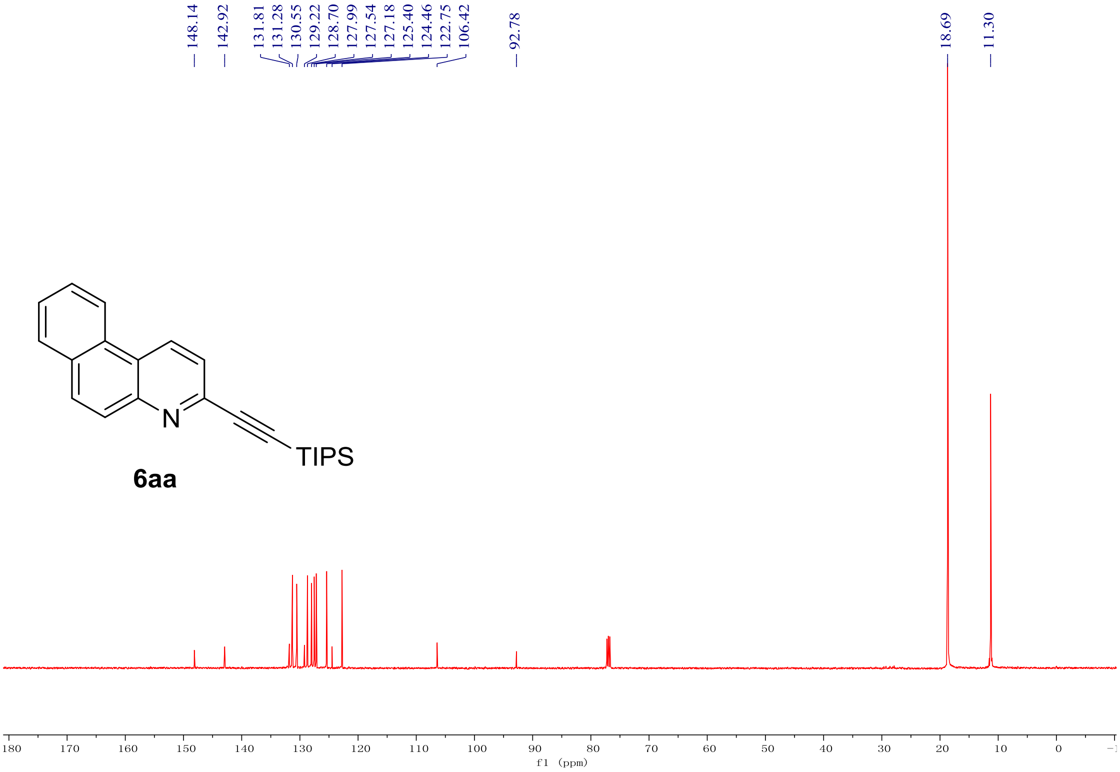

Supplementary Fig. 103. ^{13}C NMR spectra (126 MHz, $CDCl_3$, 25 ^{o}C) of **6aa**

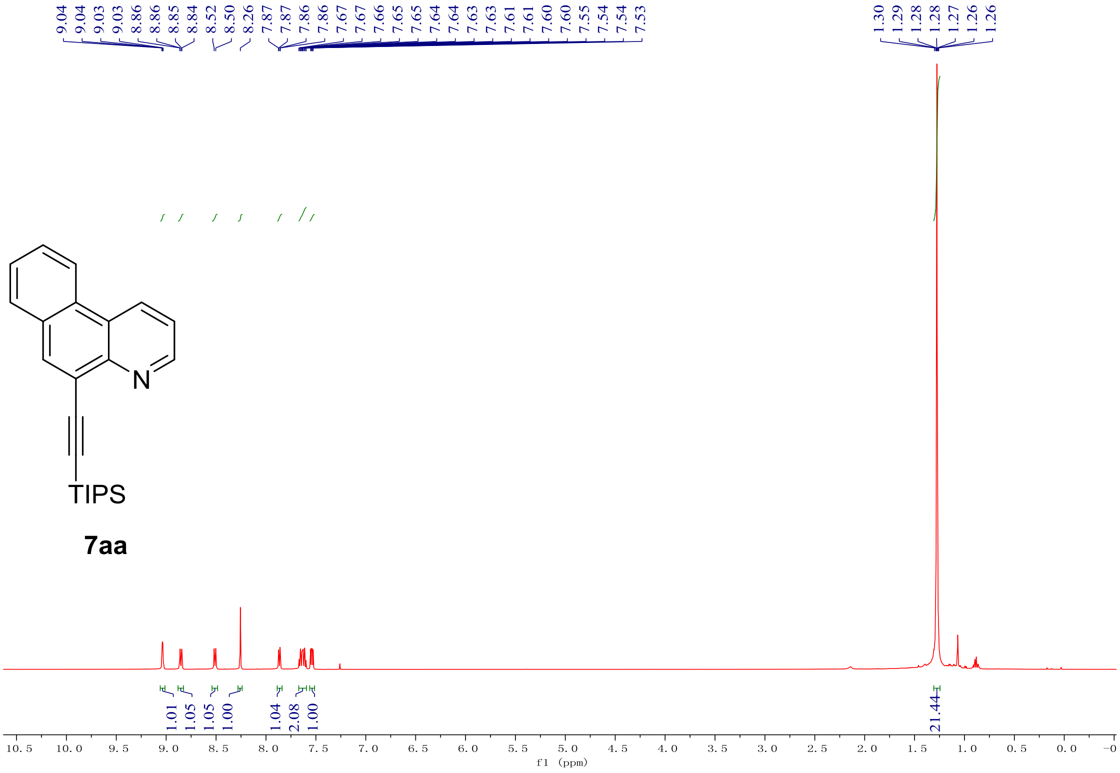

Supplementary Fig. 104. ^{1}H NMR spectra (500 MHz, $CDCl_3$, 25 °C) of **7aa**

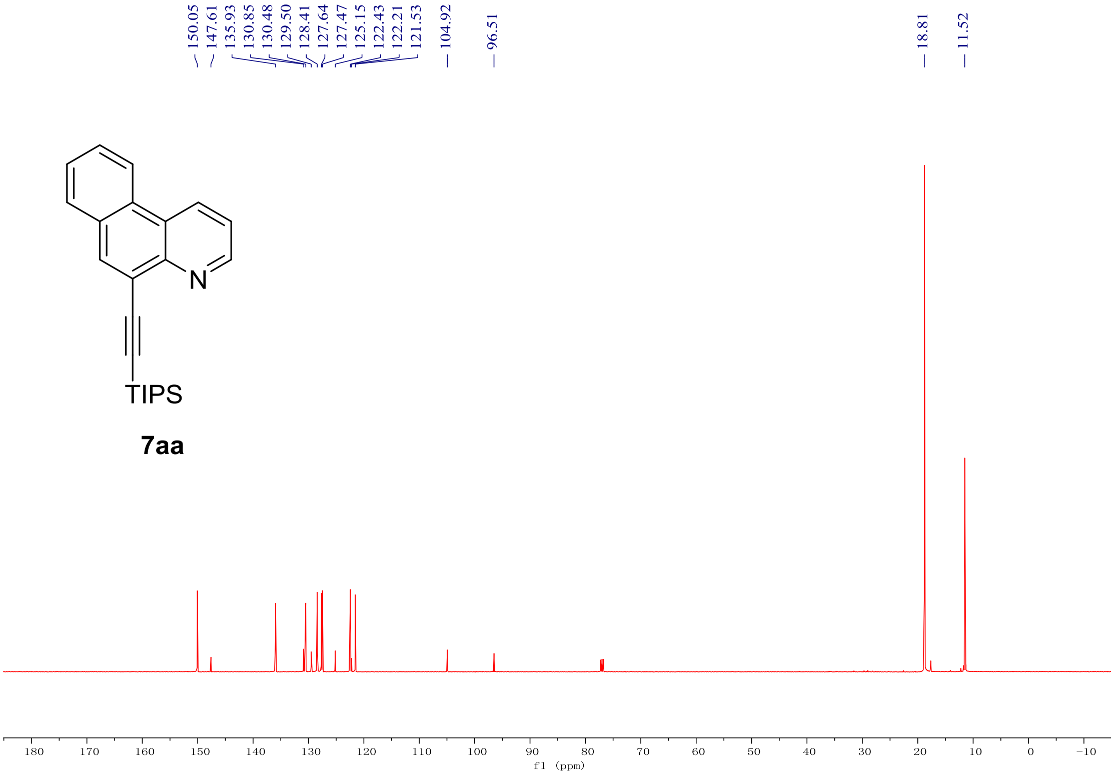

Supplementary Fig. 105. ^{13}C NMR spectra (126 MHz, $CDCl_3$, 25 °C) of **7aa**

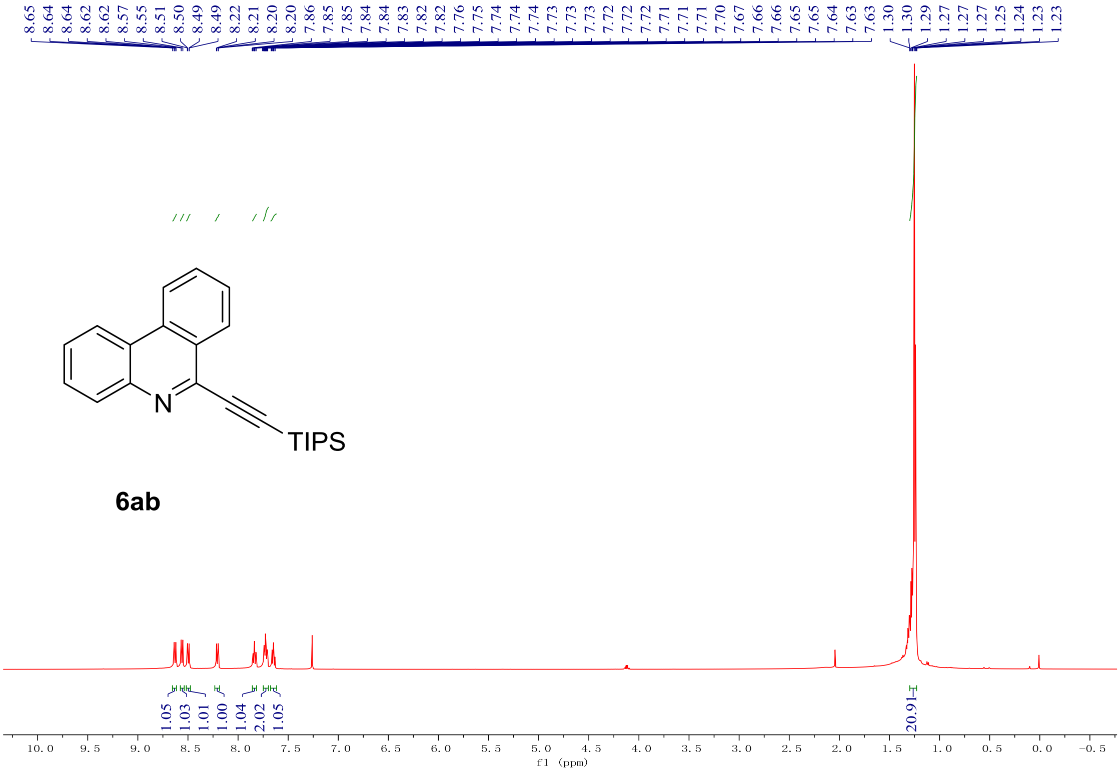

Supplementary Fig. 106. ^{1}H NMR spectra (500 MHz, $CDCl_3$, 25 °C) of **6ab**

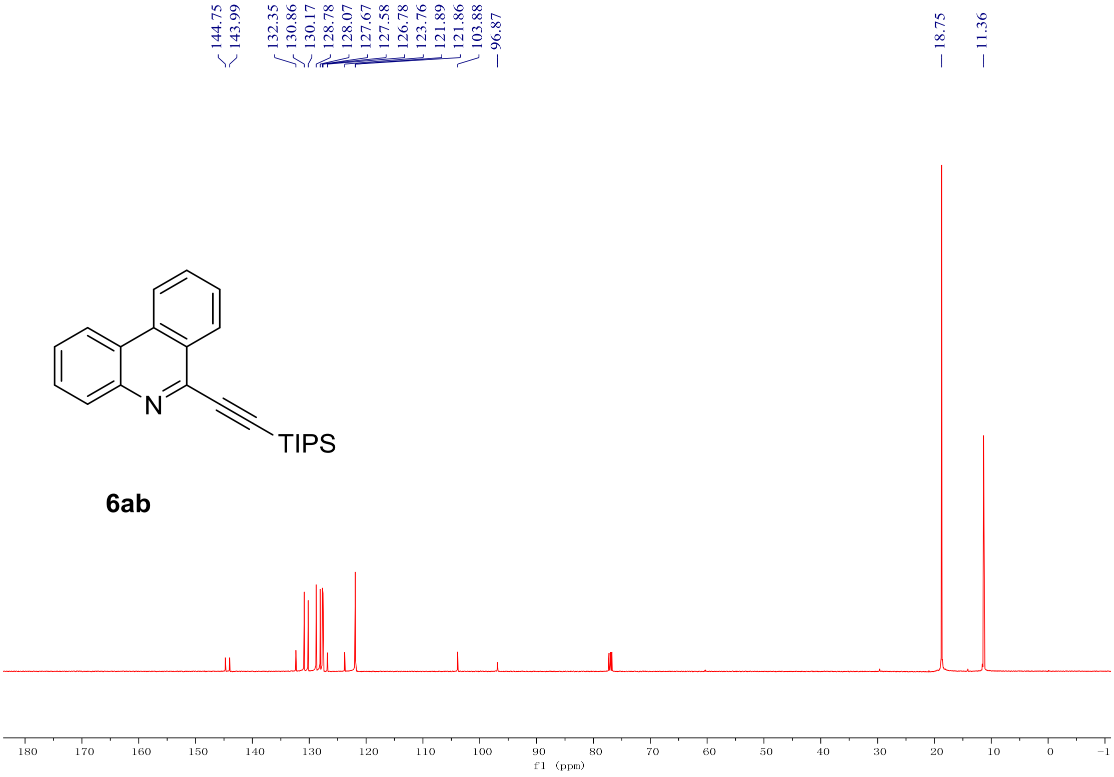

Supplementary Fig. 107. ^{13}C NMR spectra (126 MHz, $CDCl_3$, 25 °C) of **6ab**

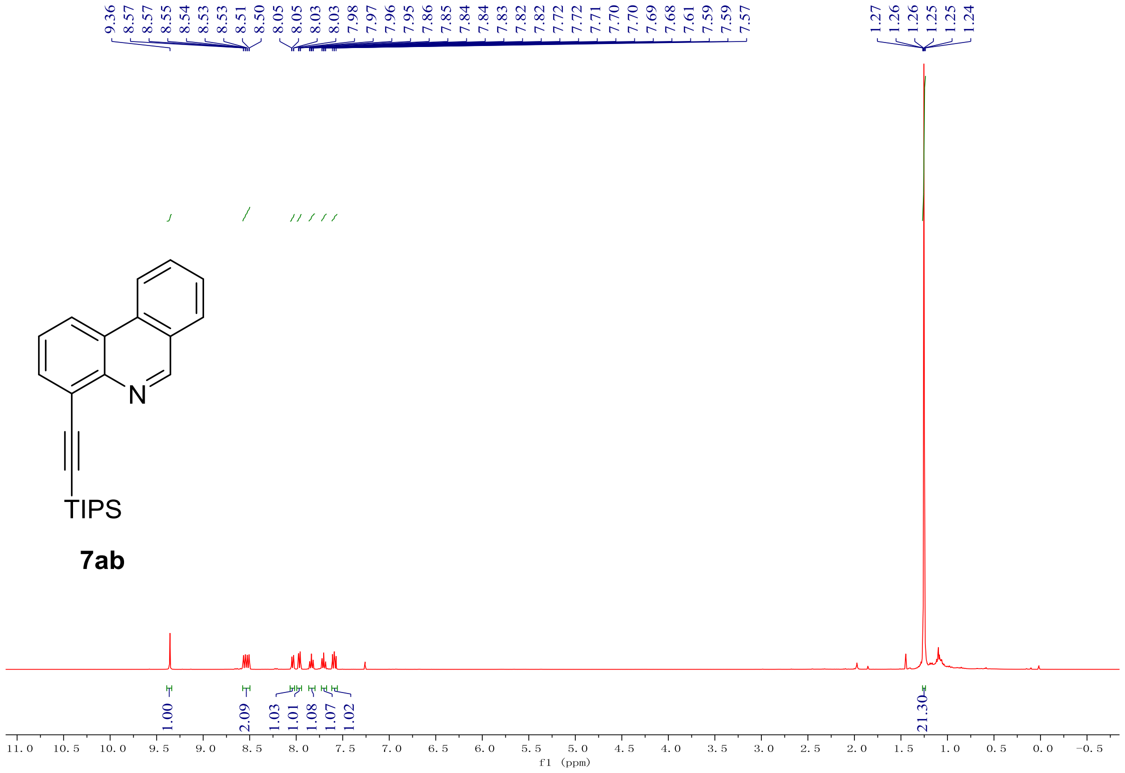

Supplementary Fig. 108. ^{1}H NMR spectra (400 MHz, $CDCl_3$, 25 ^{o}C) of **7ab**

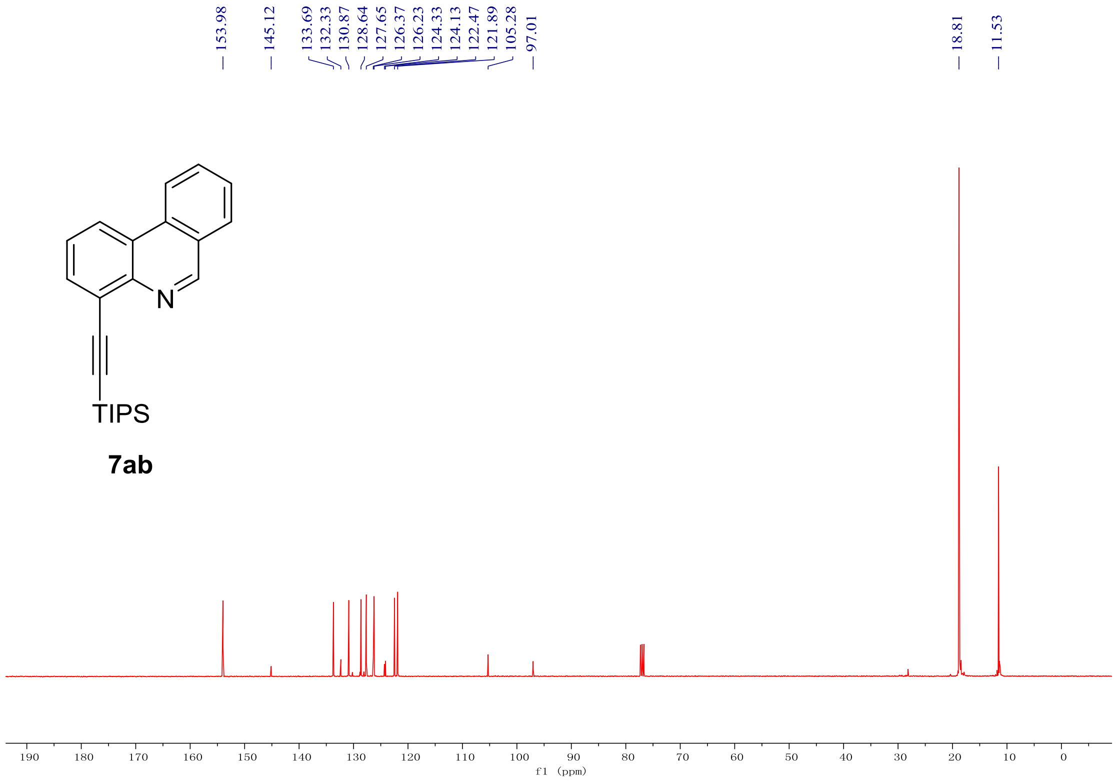

Supplementary Fig. 109. ^{13}C NMR spectra (101 MHz, $CDCl_3$, 25 ^{o}C) of **7ab**

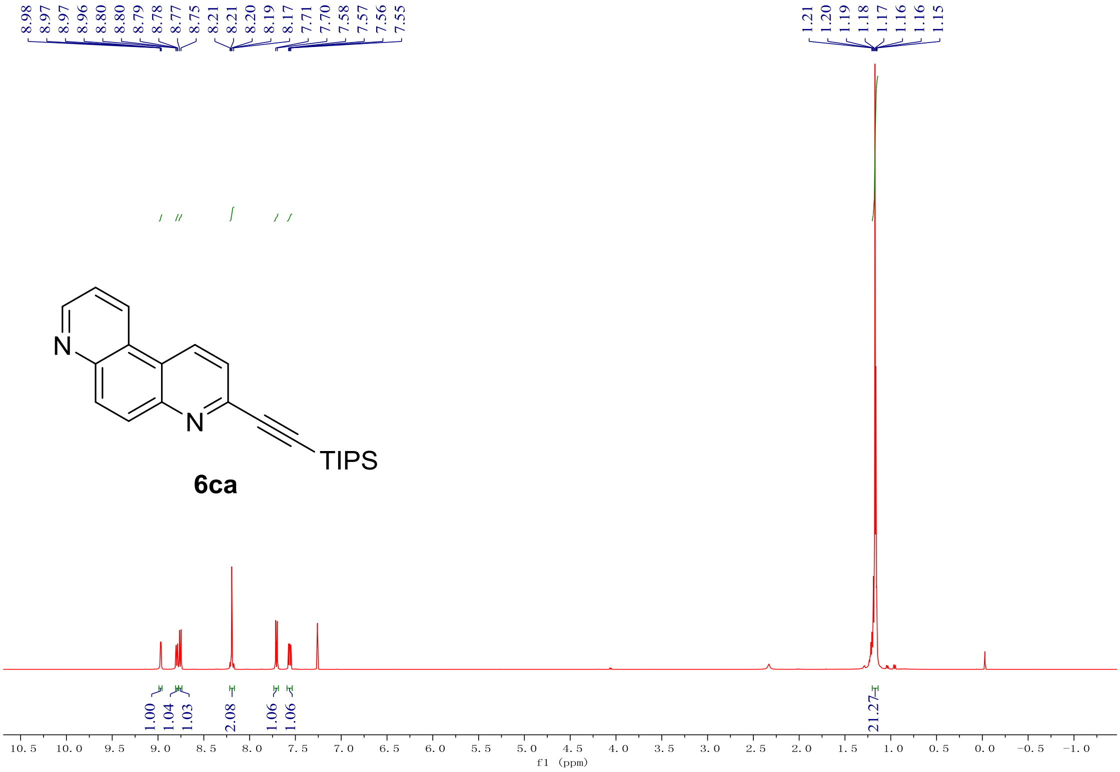

Supplementary Fig. 110. ^{1}H NMR spectra (500 MHz, $CDCl_3$, 25 ^{o}C) of **6ca**

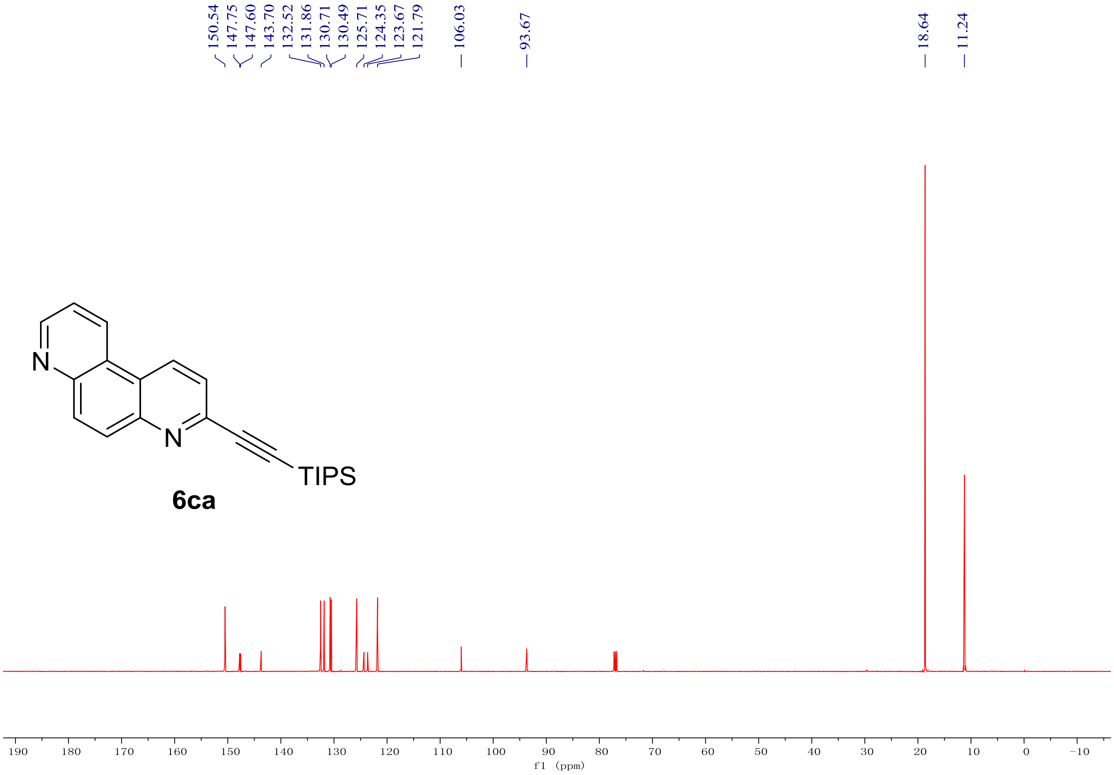

Supplementary Fig. 111. ^{13}C NMR spectra (126 MHz, $CDCl_3$, 25 ^{o}C) of **6ca**

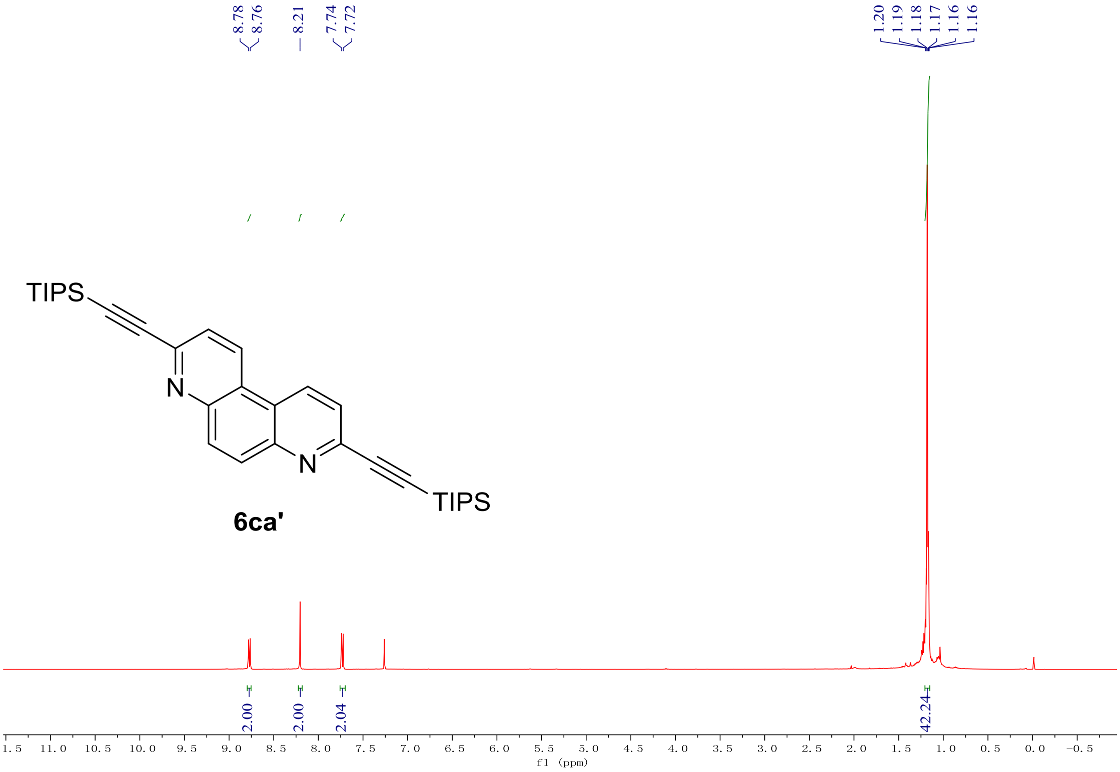

Supplementary Fig. 112. ^{1}H NMR spectra (500 MHz, $CDCl_3$, 25 °C) of **6ca'**

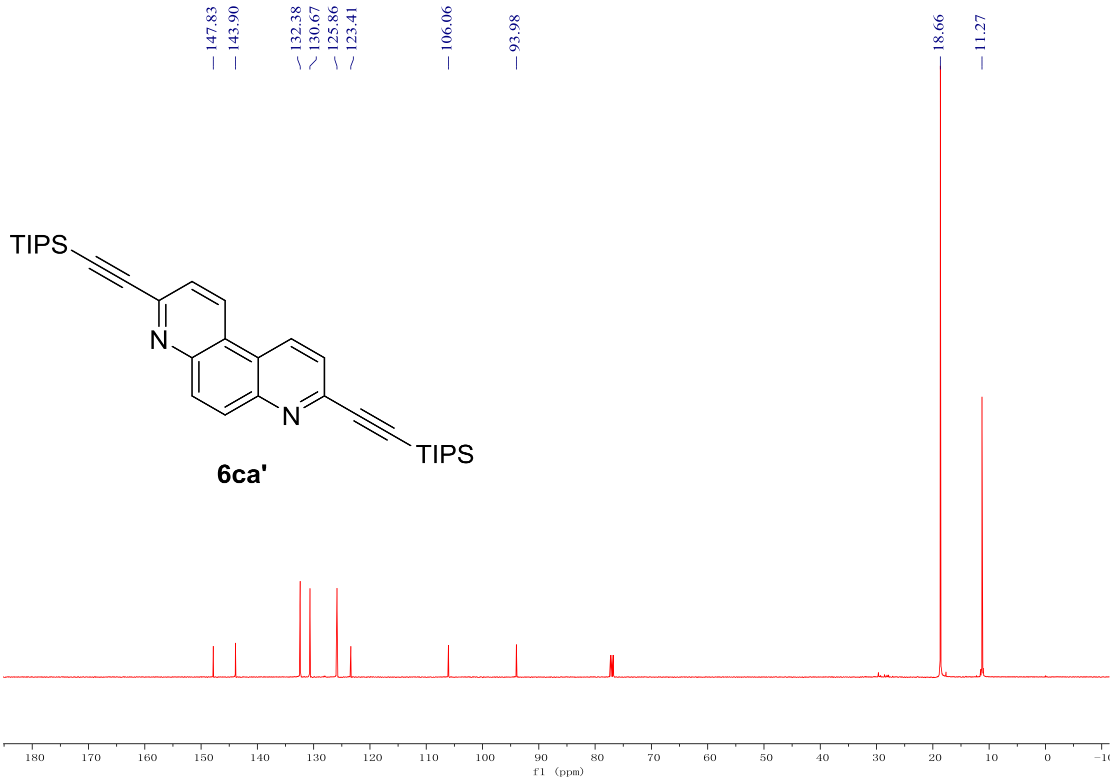

Supplementary Fig. 113. ^{13}C NMR spectra (126 MHz, $CDCl_3$, 25 °C) of **6ca'**

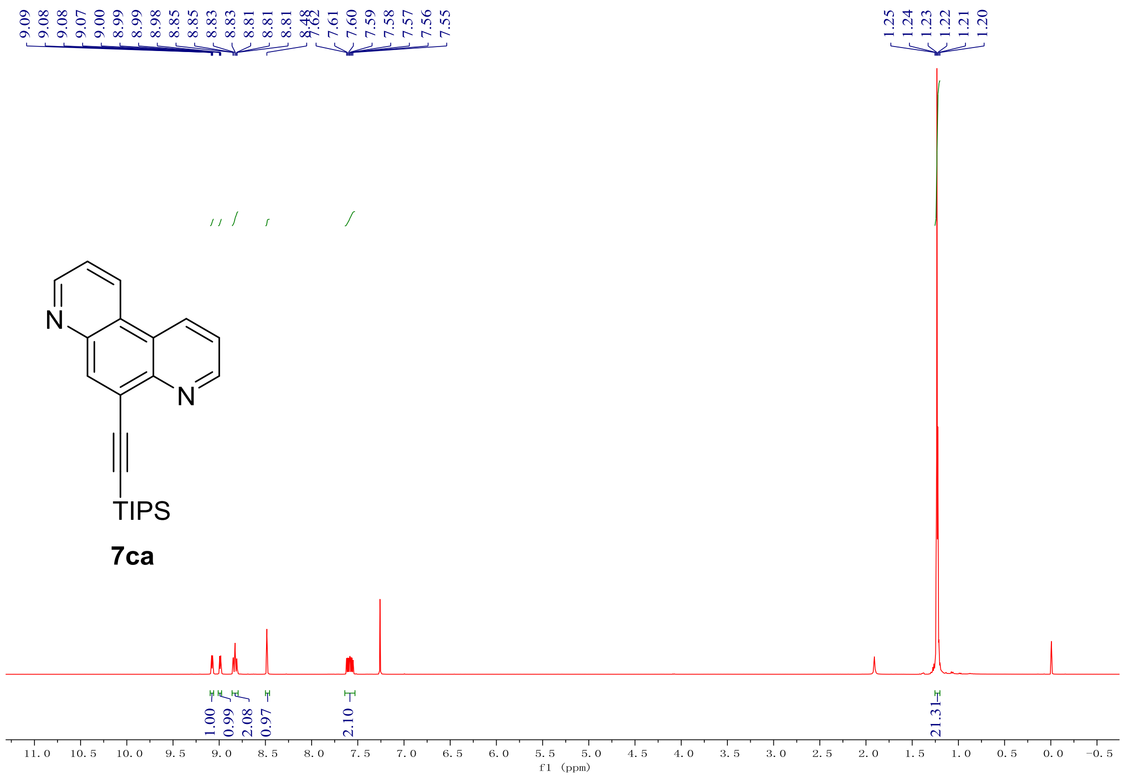

Supplementary Fig. 114. ^{1}H NMR spectra (400 MHz, $CDCl_3$, 25 °C) of **7ca**

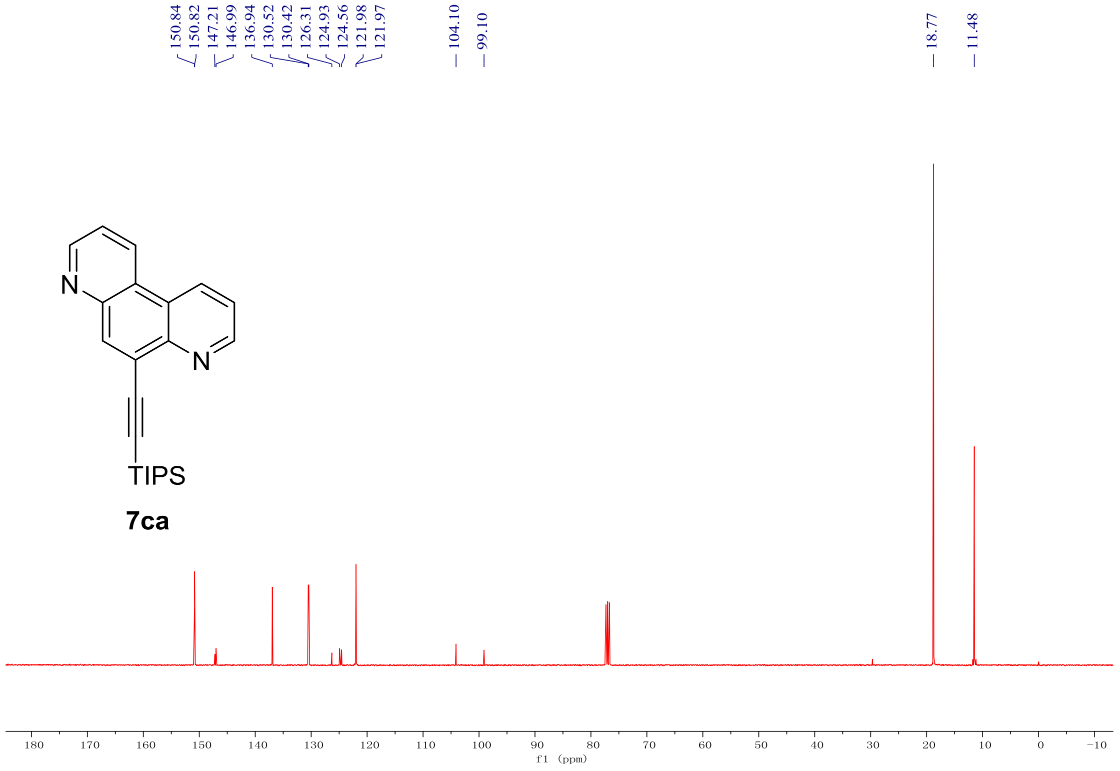

Supplementary Fig. 115. ^{13}C NMR spectra (101 MHz, $CDCl_3$, 25 °C) of **7ca**

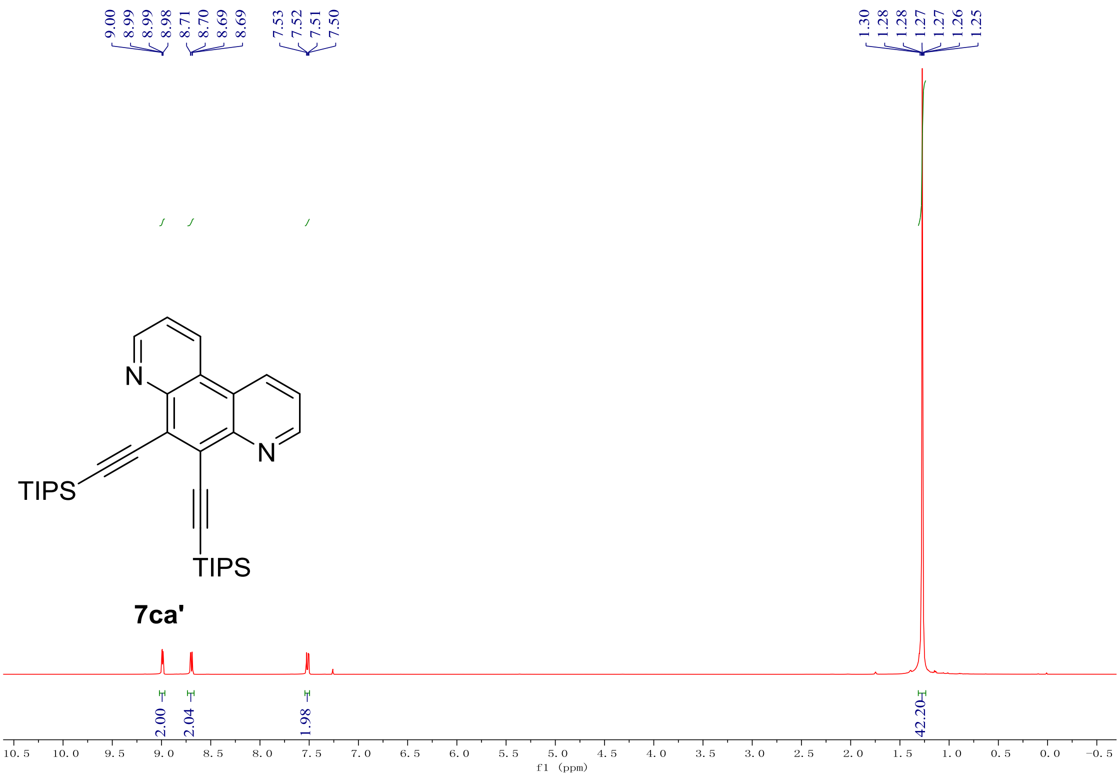

Supplementary Fig. 116. ^{1}H NMR spectra (500 MHz, $CDCl_3$, 25 °C) of **7ca'**

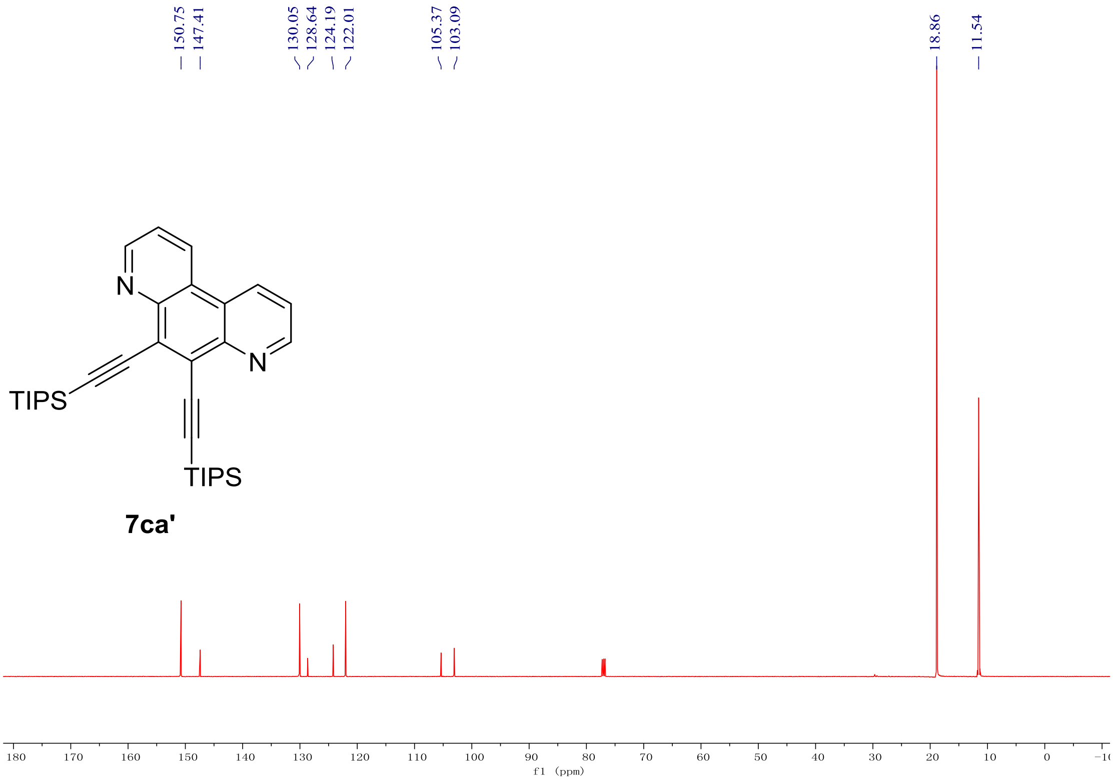

Supplementary Fig. 117. ^{13}C NMR spectra (126 MHz, $CDCl_3$, 25 °C) of **7ca'**

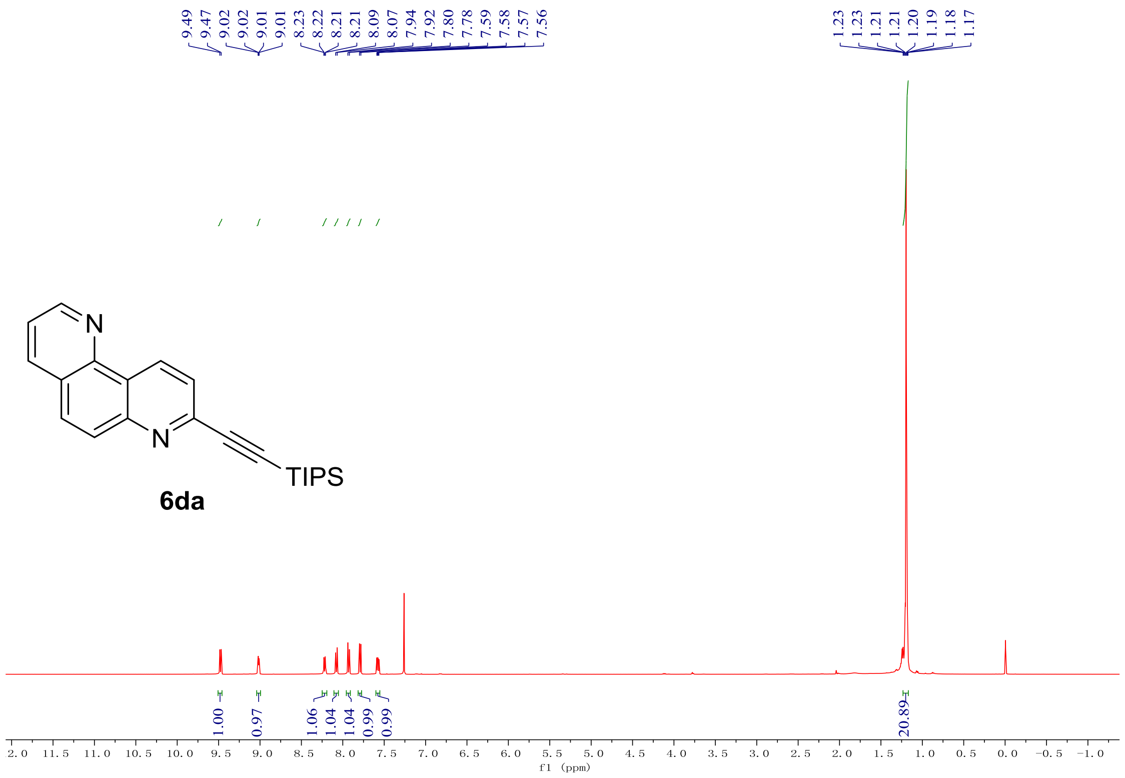

Supplementary Fig. 118. ^{1}H NMR spectra (500 MHz, $CDCl_3$, 25 °C) of **6da**

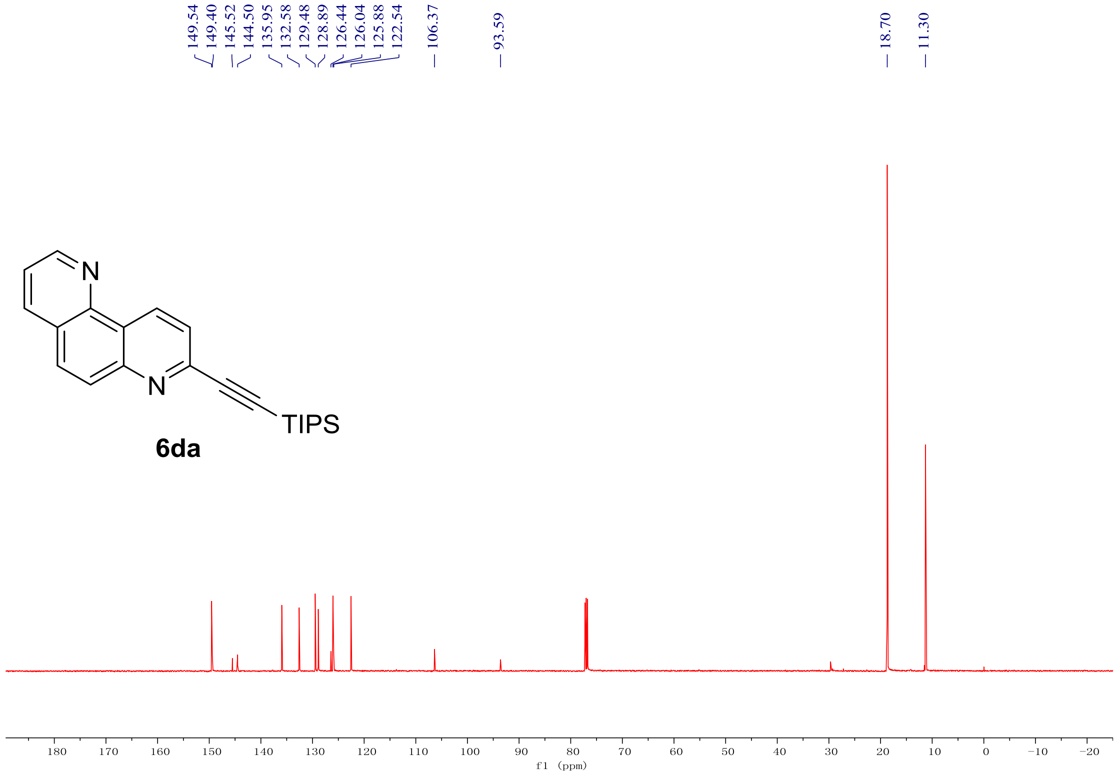

Supplementary Fig. 119. ^{13}C NMR spectra (126 MHz, $CDCl_3$, 25 °C) of **6da**

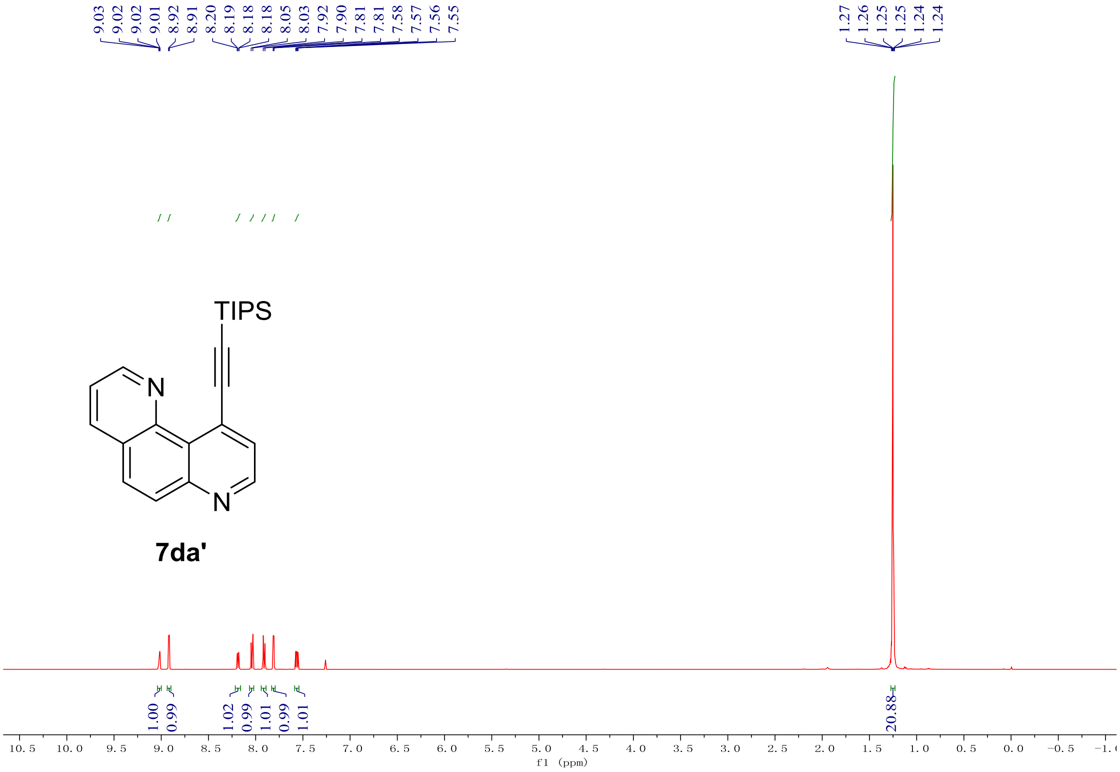

Supplementary Fig. 120. ^{1}H NMR spectra (500 MHz, $CDCl_3$, 25 °C) of **7da'**

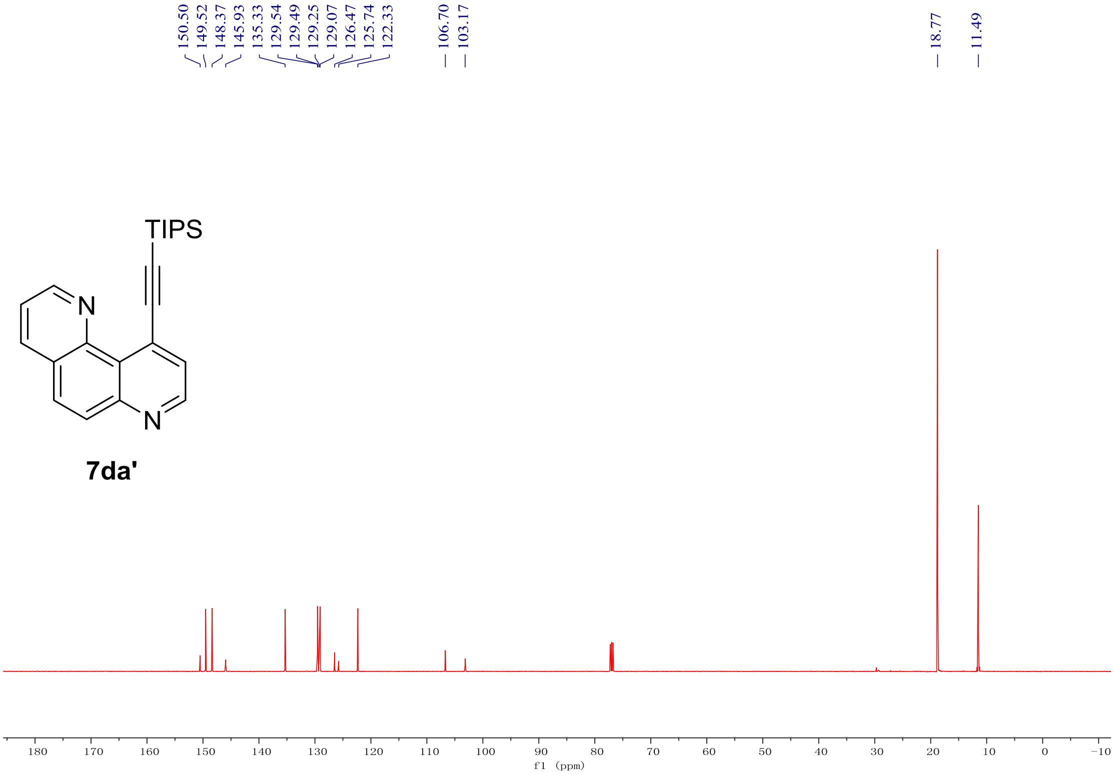

Supplementary Fig. 121. ^{13}C NMR spectra (126 MHz, $CDCl_3$, 25 °C) of **7da'**

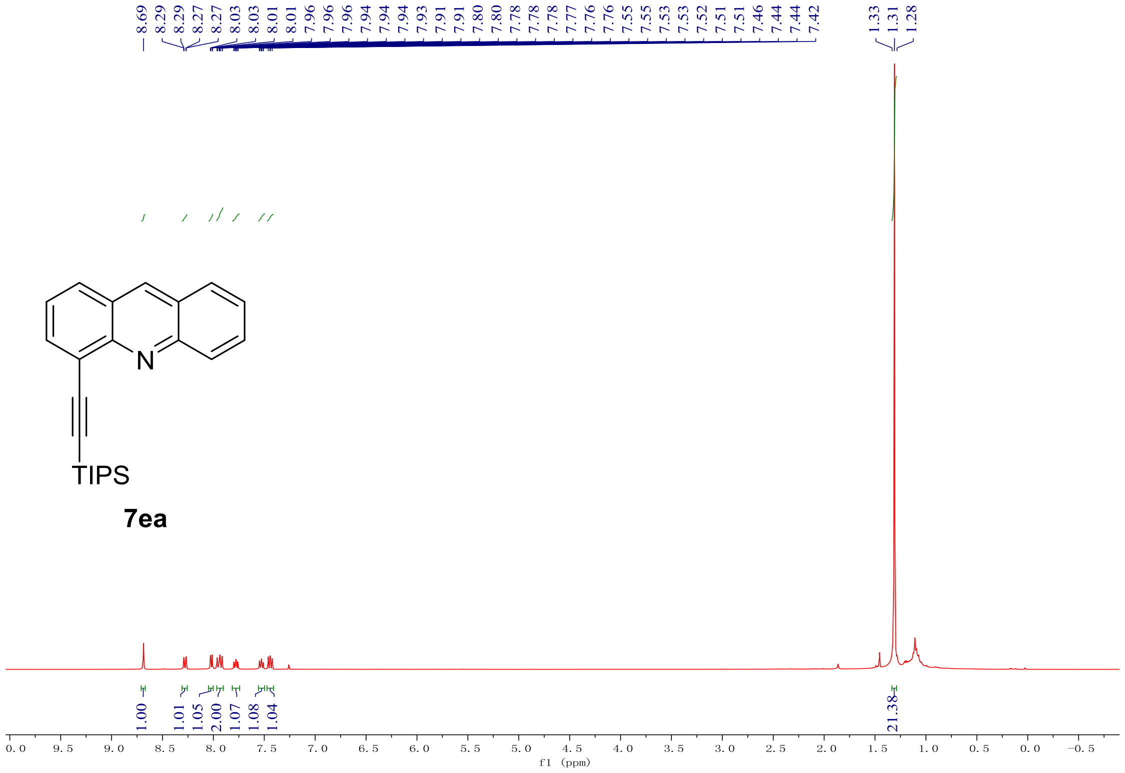

Supplementary Fig. 122. ^{1}H NMR spectra (400 MHz, $CDCl_3$, 25 °C) of **7ea**

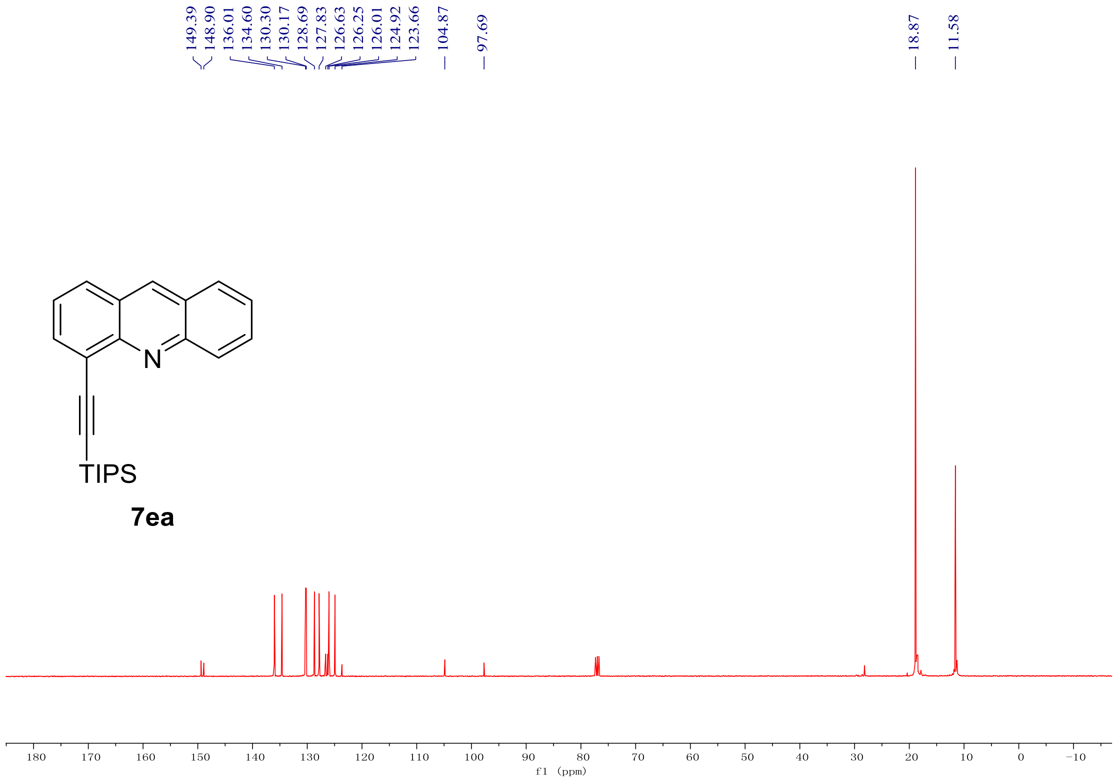

Supplementary Fig. 123. ^{13}C NMR spectra (101 MHz, $CDCl_3$, 25 °C) of **7ea**

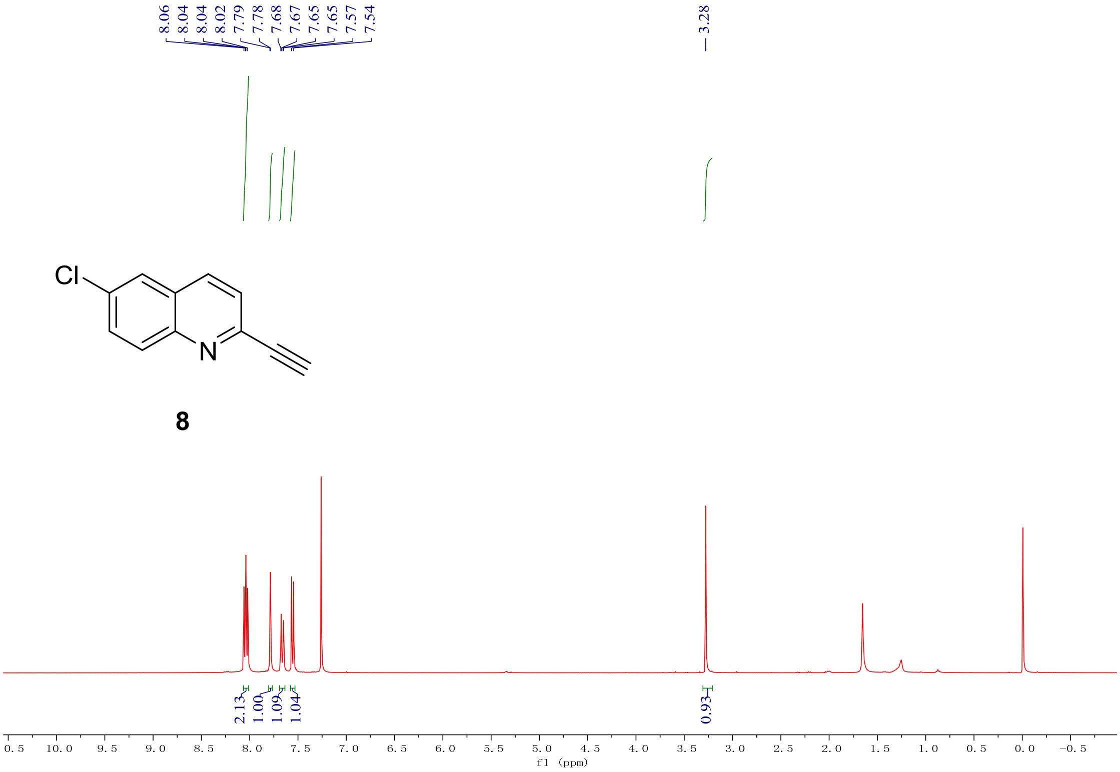

Supplementary Fig. 124. ^{1}H NMR spectra (400 MHz, $CDCl_3$, 25 ^{o}C) of **8**

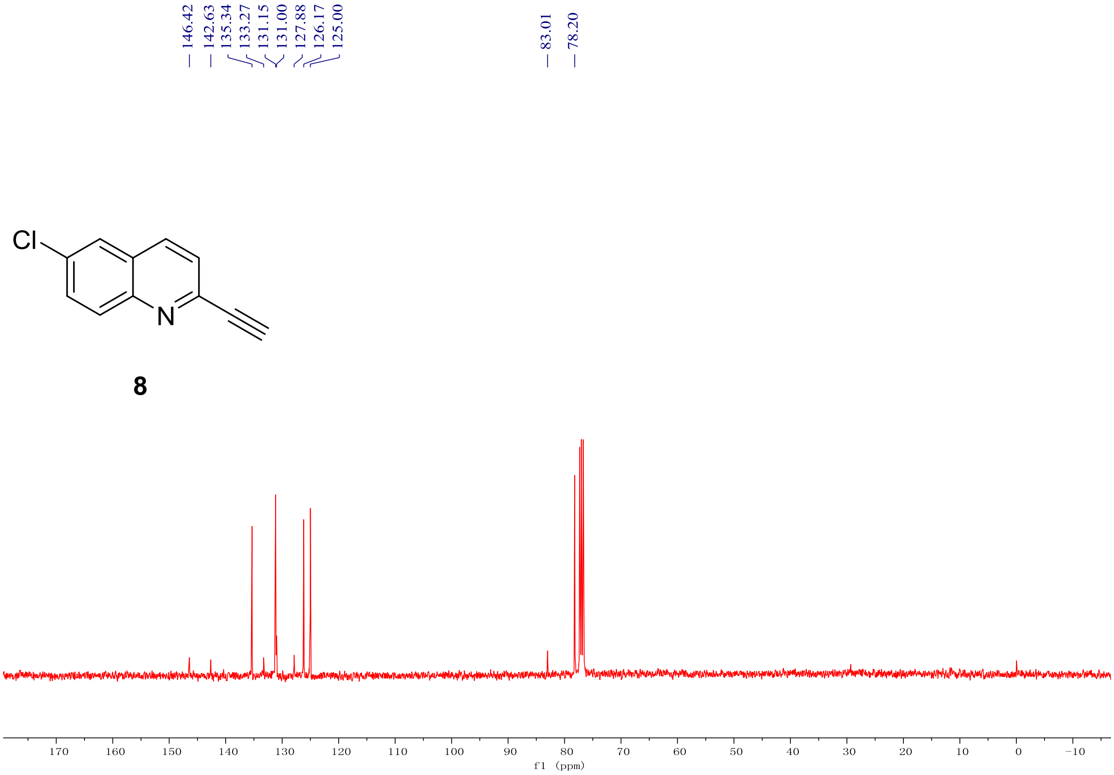

Supplementary Fig. 125. ^{13}C NMR spectra (101 MHz, $CDCl_3$, 25 ^{o}C) of **8**

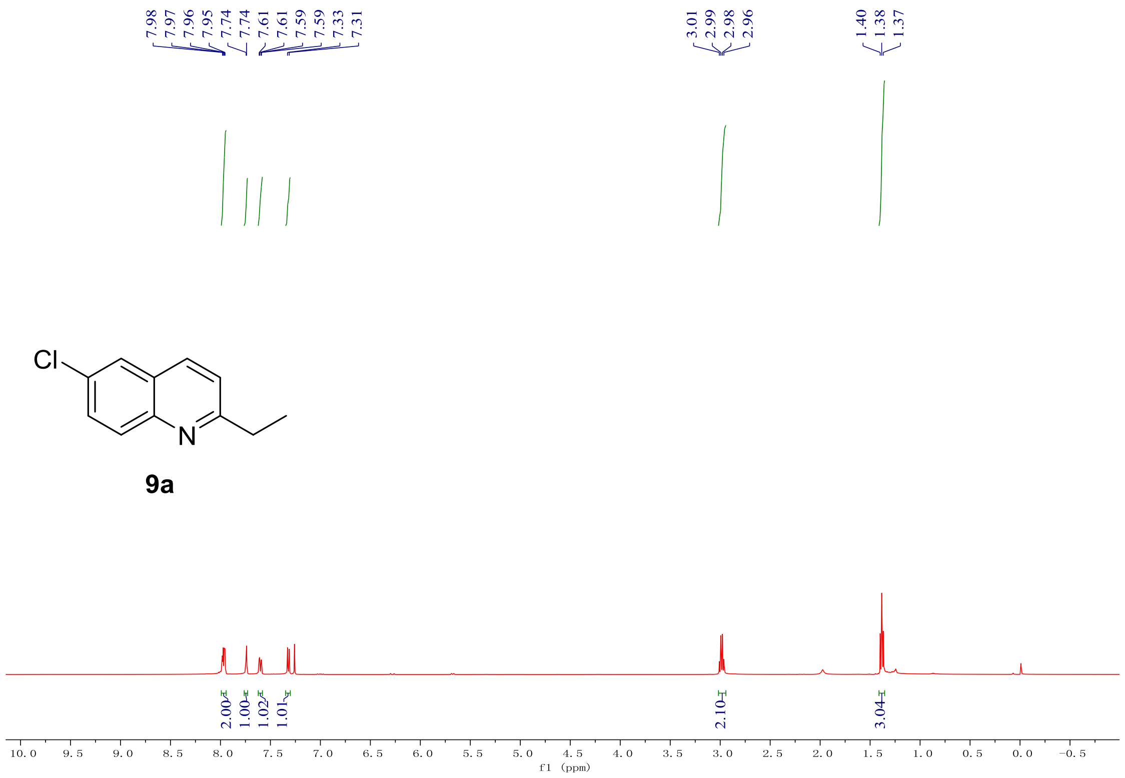

Supplementary Fig. 126. ^{1}H NMR spectra (500 MHz, $CDCl_3$, 25 ^{o}C) of **9a**

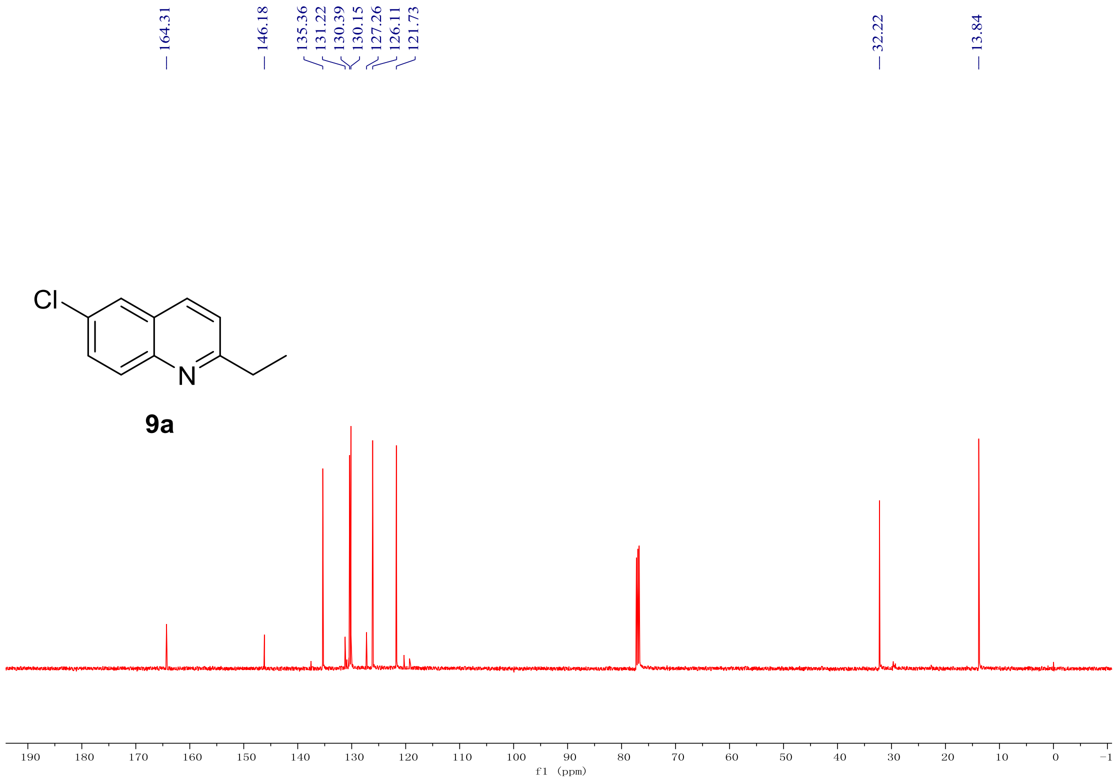

Supplementary Fig. 127. ^{13}C NMR spectra (126 MHz, $CDCl_3$, 25 ^{o}C) of **9a**

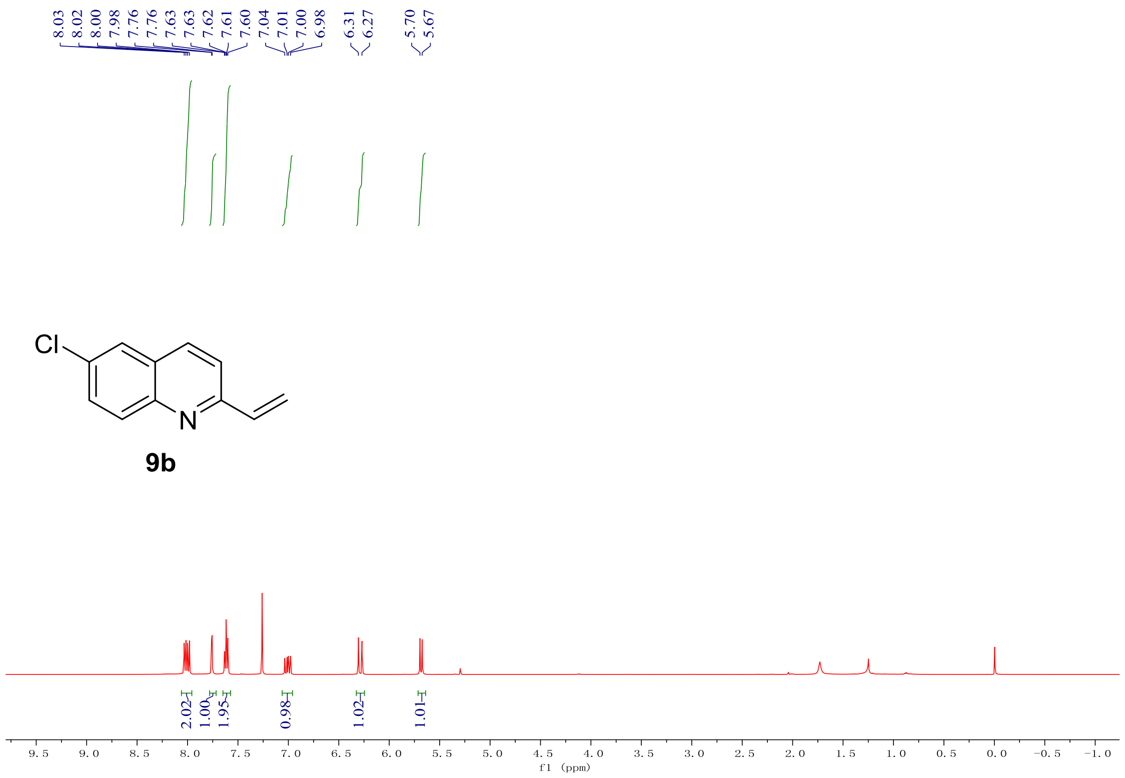

Supplementary Fig. 128. ^{1}H NMR spectra (500 MHz, $CDCl_3$, 25 °C) of **9b**

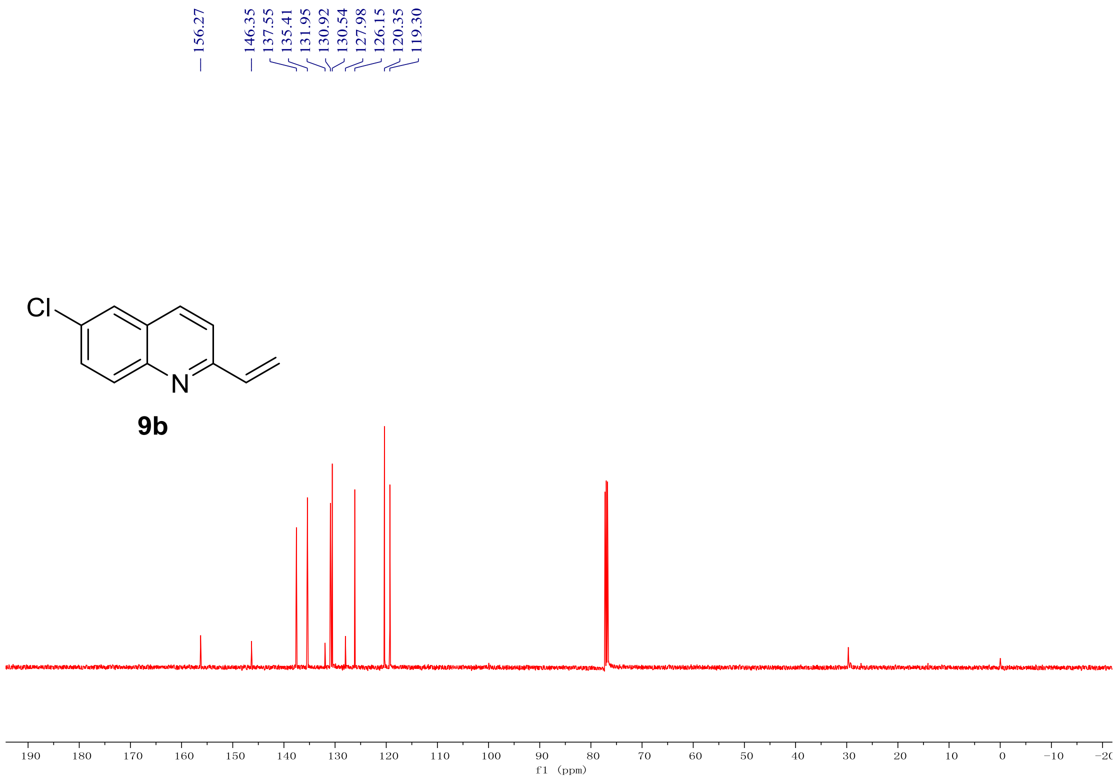

Supplementary Fig. 129. ^{13}C NMR spectra (126 MHz, $CDCl_3$, 25 °C) of **9b**

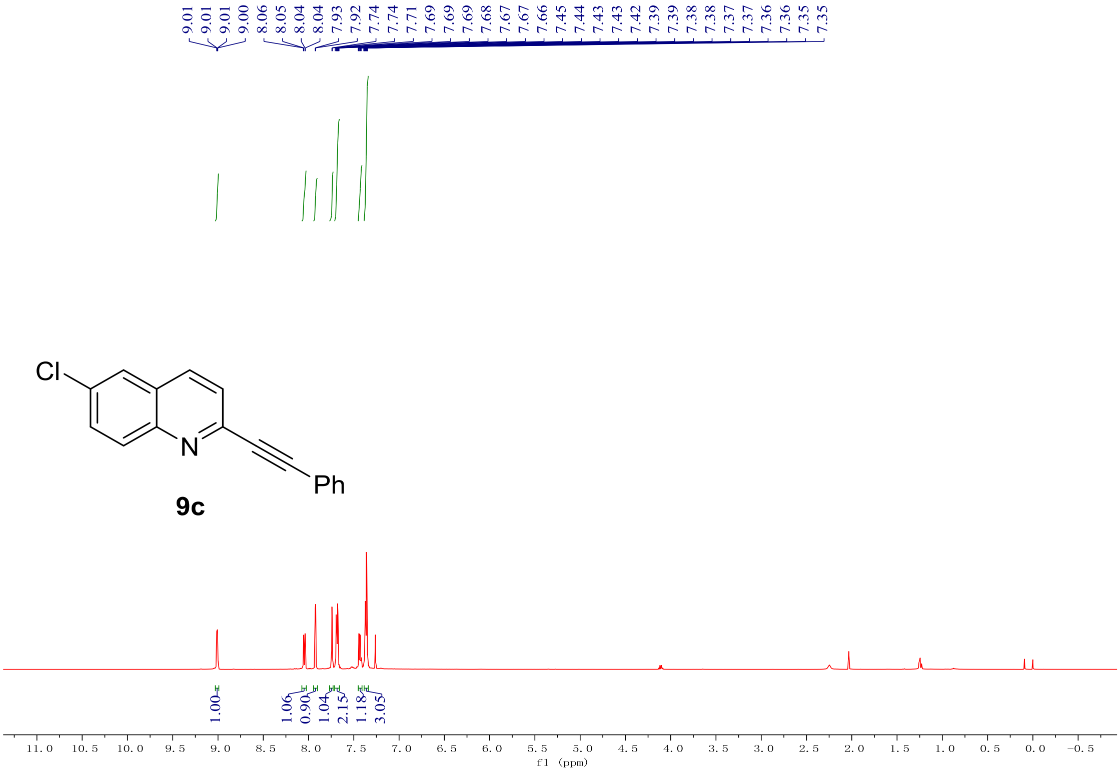

Supplementary Fig. 130. ^{1}H NMR spectra (500 MHz, $CDCl_3$, 25 ^{o}C) of **9c**

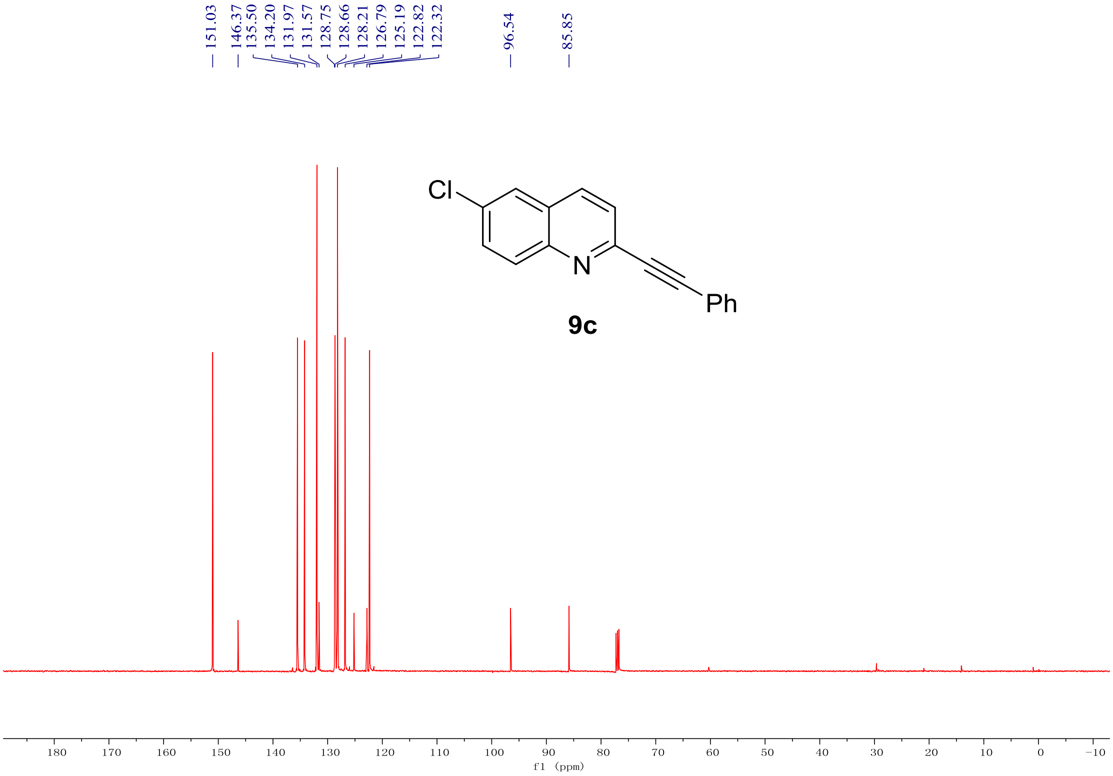

Supplementary Fig. 131. ^{13}C NMR spectra (126 MHz, $CDCl_3$, 25 ^{o}C) of **9c**

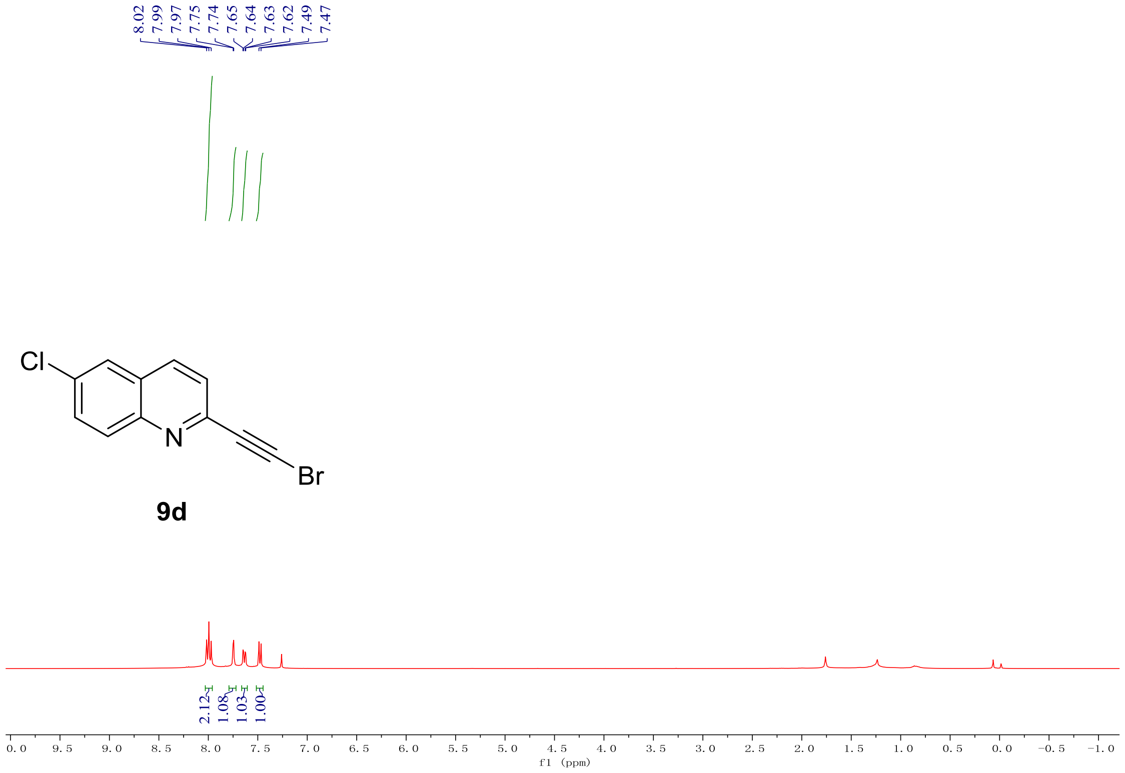

Supplementary Fig. 132. ^{1}H NMR spectra (400 MHz, $CDCl_3$, 25 °C) of **9d**

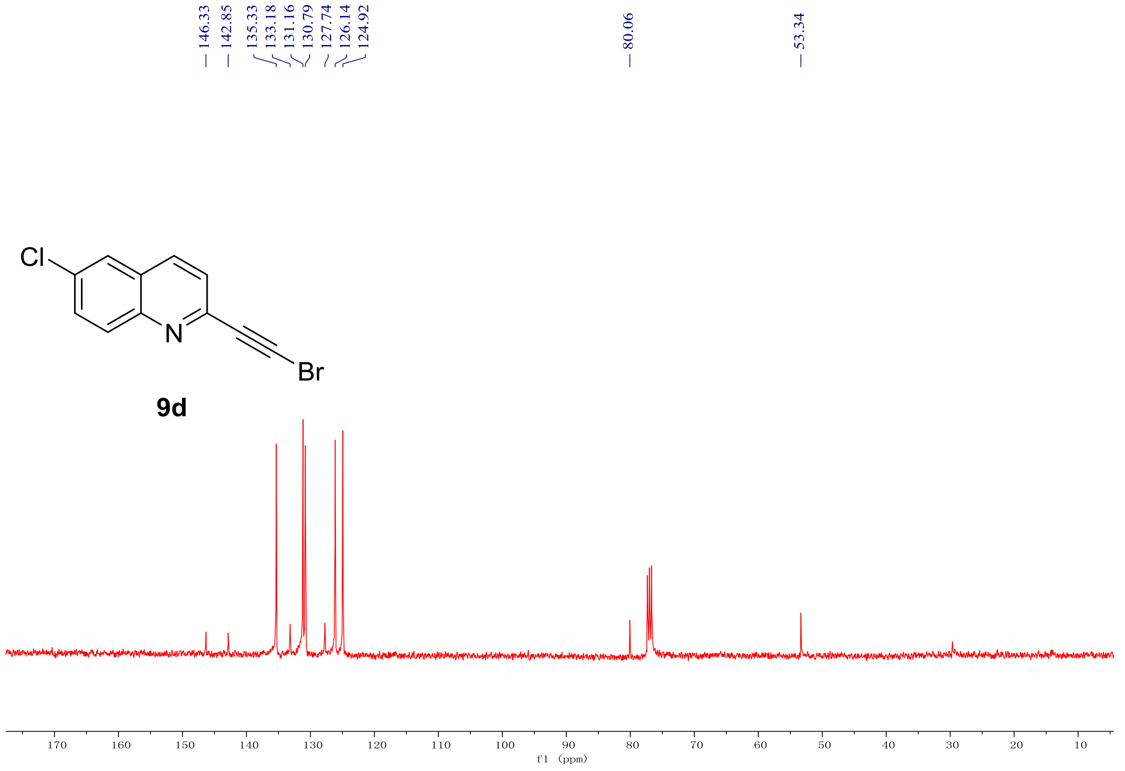

Supplementary Fig. 133. ^{13}C NMR spectra (101 MHz, $CDCl_3$, 25 °C) of **9d**

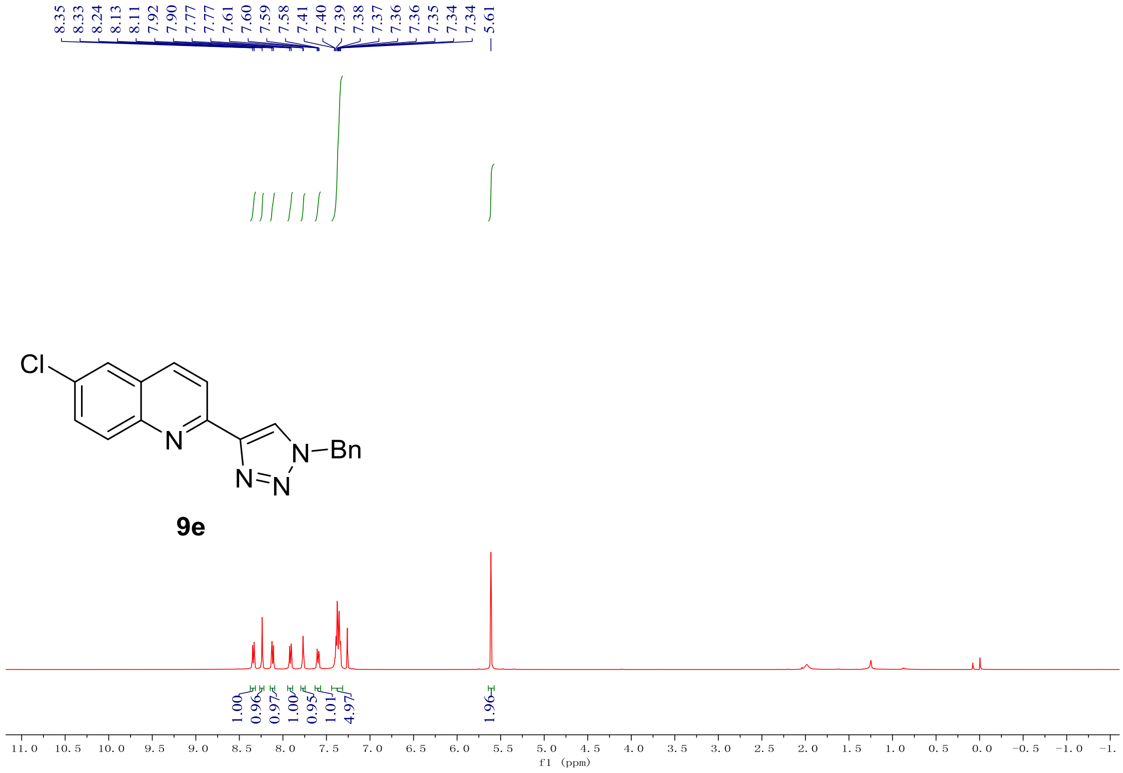

Supplementary Fig. 134. ^{1}H NMR spectra (500 MHz, $CDCl_3$, 25 ^{o}C) of **9e**

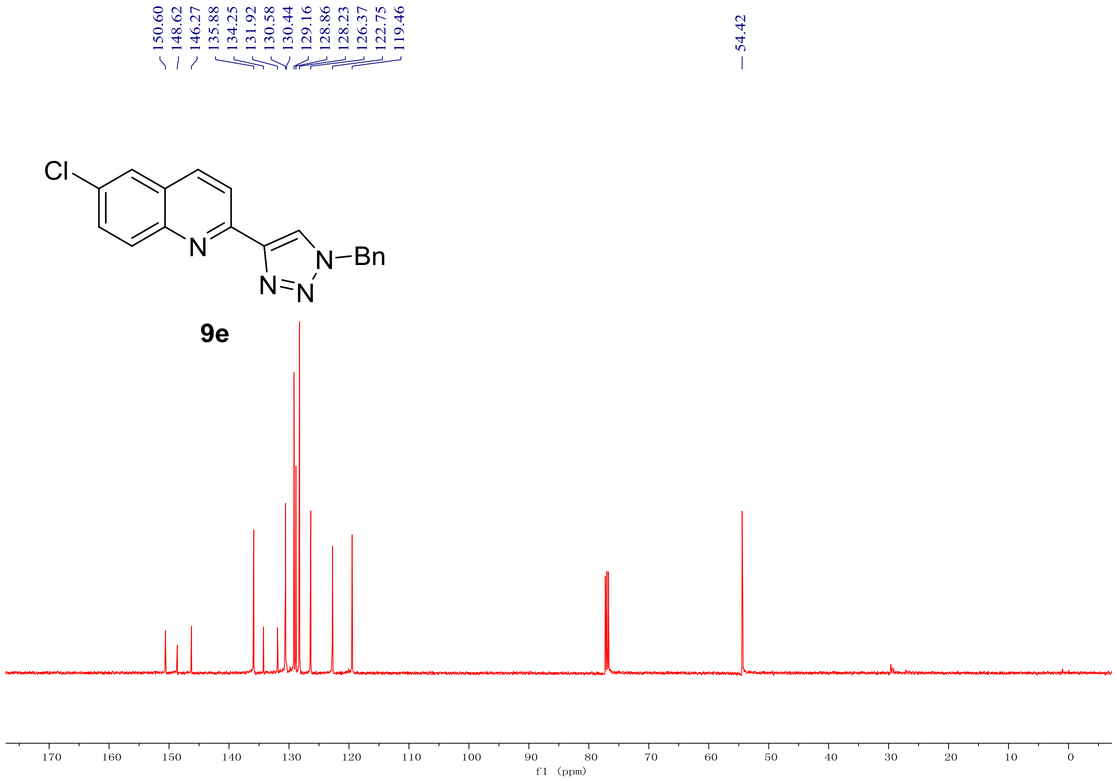

Supplementary Fig. 135. ^{13}C NMR spectra (126 MHz, $CDCl_3$, 25 ^{o}C) of **9e**

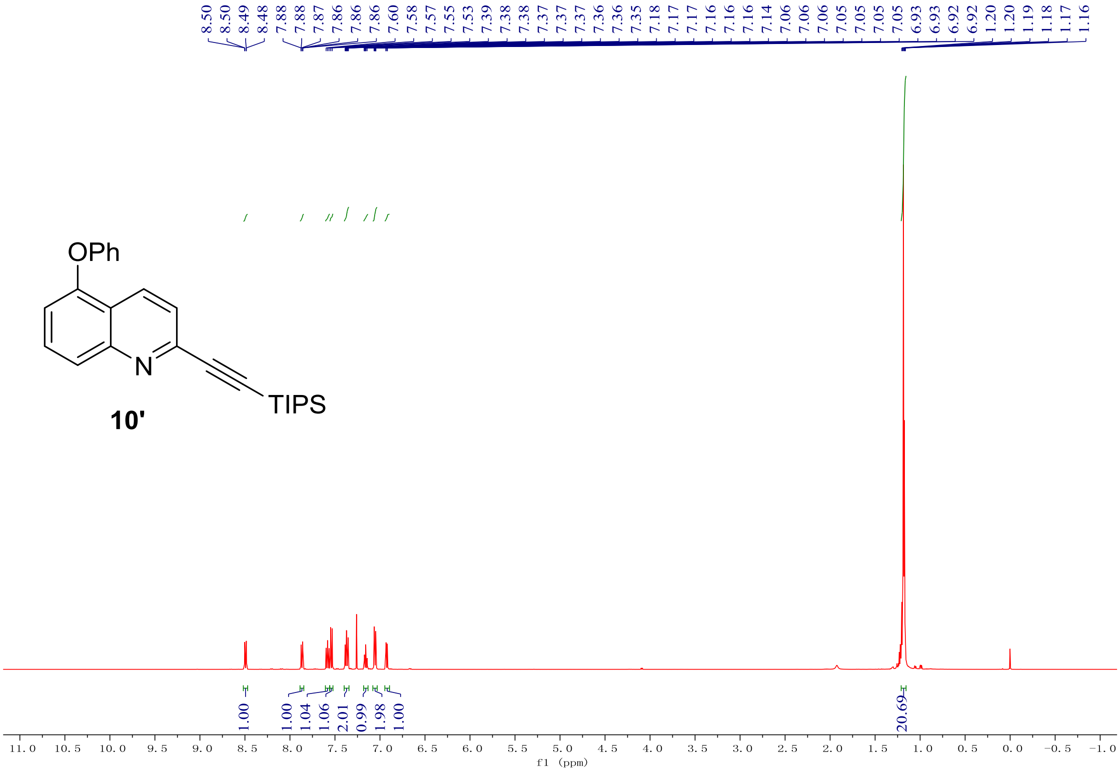

Supplementary Fig. 136. ^{1}H NMR spectra (500 MHz, $CDCl_3$, 25 °C) of **10'**

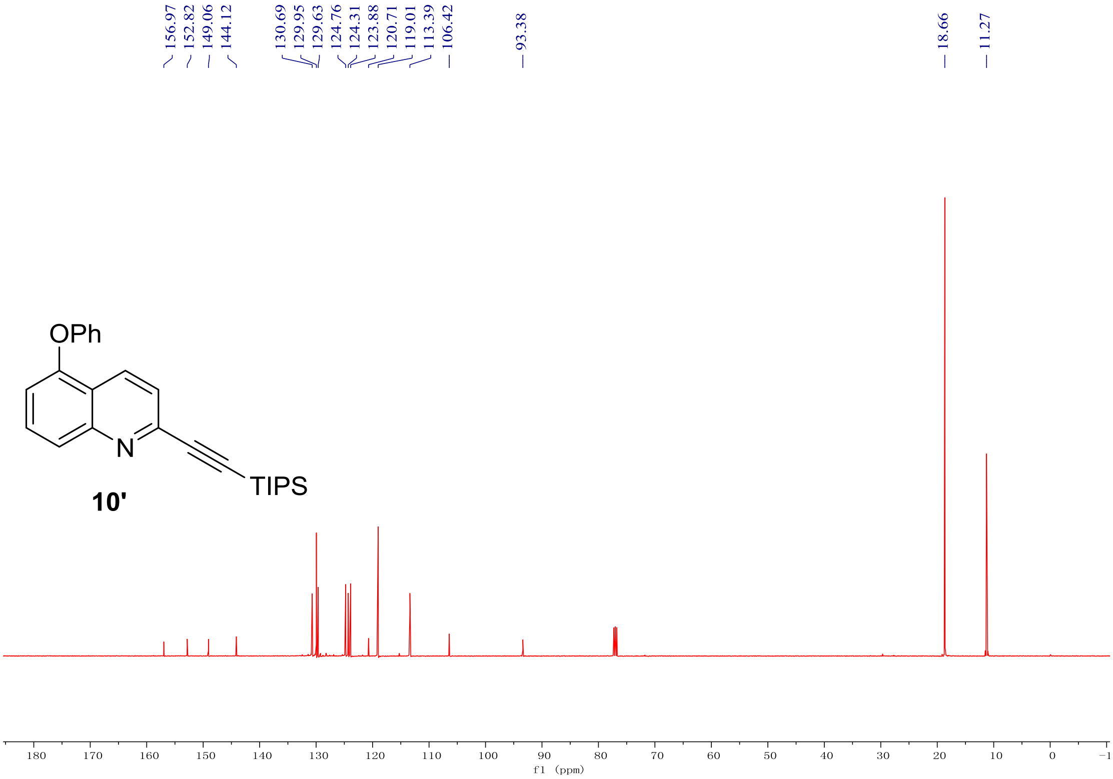

Supplementary Fig. 137. ^{13}C NMR spectra (126 MHz, $CDCl_3$, 25 °C) of **10'**

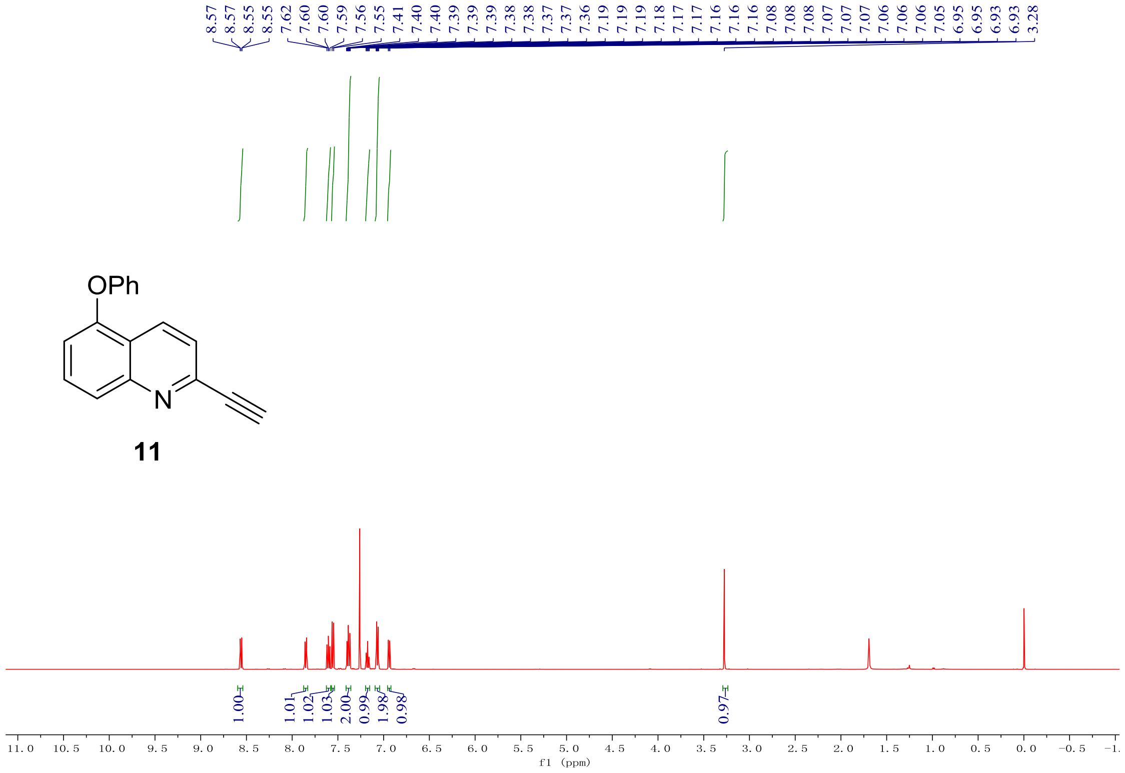

Supplementary Fig. 138. ^{1}H NMR spectra (500 MHz, $CDCl_3$, 25 ^{o}C) of **11**

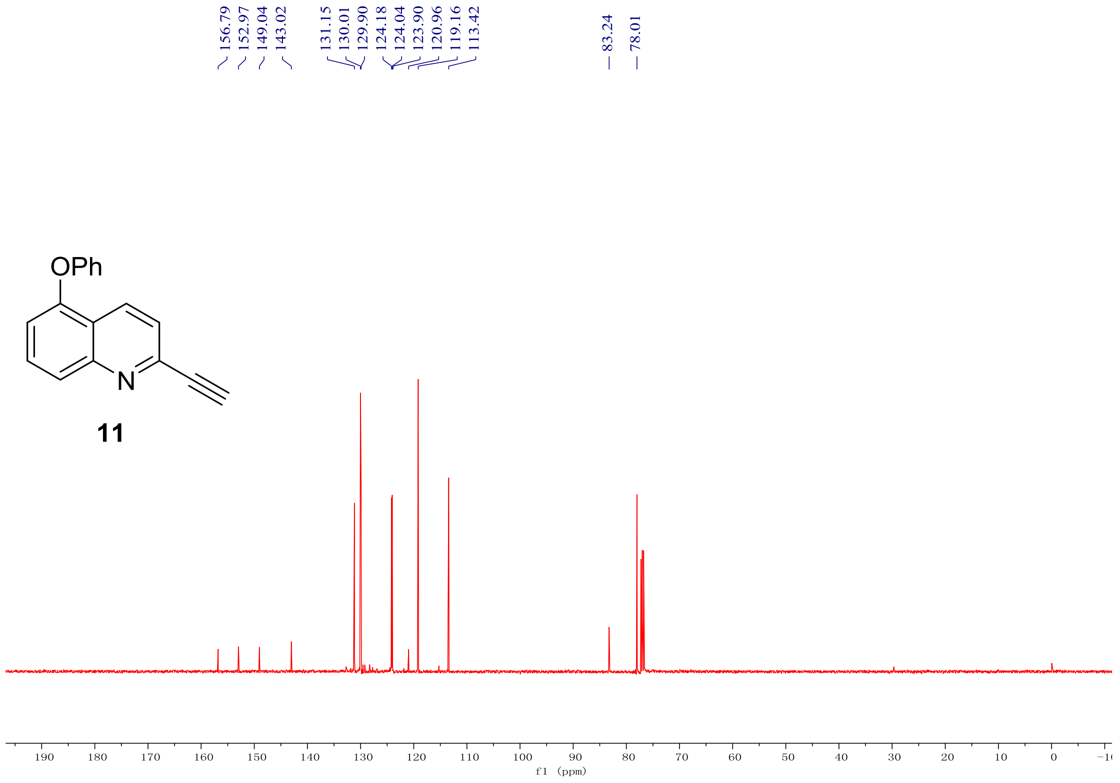

Supplementary Fig. 139. ^{13}C NMR spectra (126 MHz, $CDCl_3$, 25 ^{o}C) of **11**

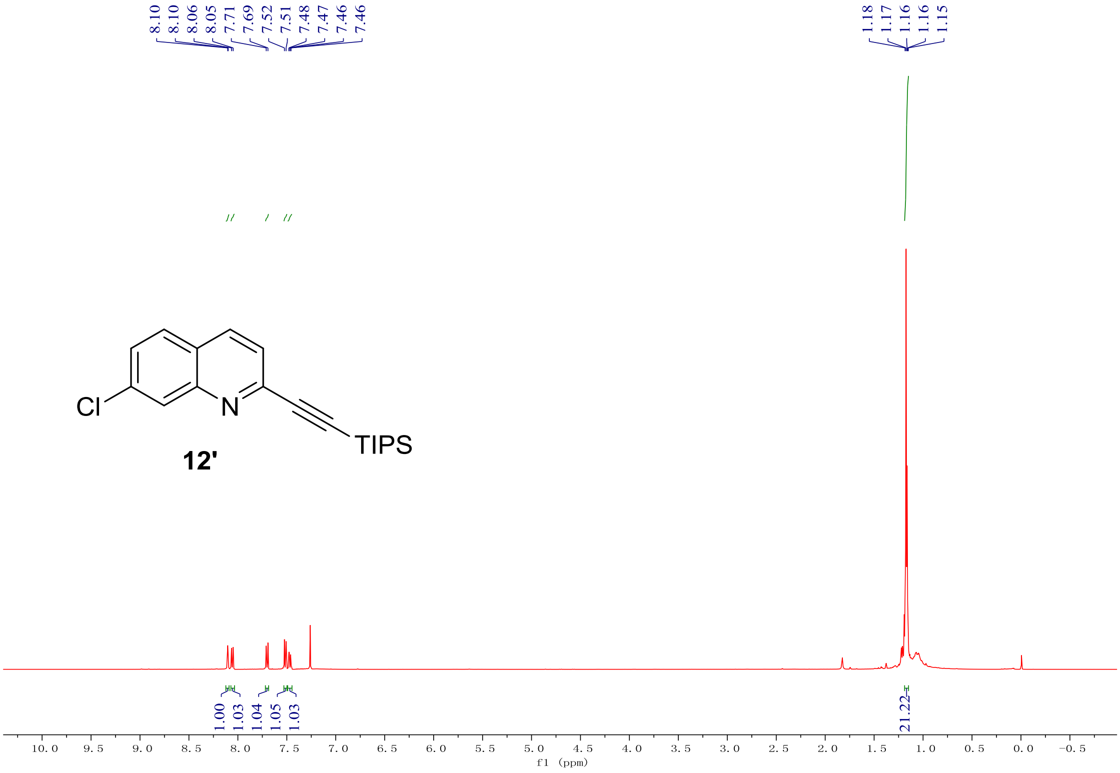

Supplementary Fig. 140. ^{1}H NMR spectra (500 MHz, $CDCl_3$, 25 °C) of **12'**

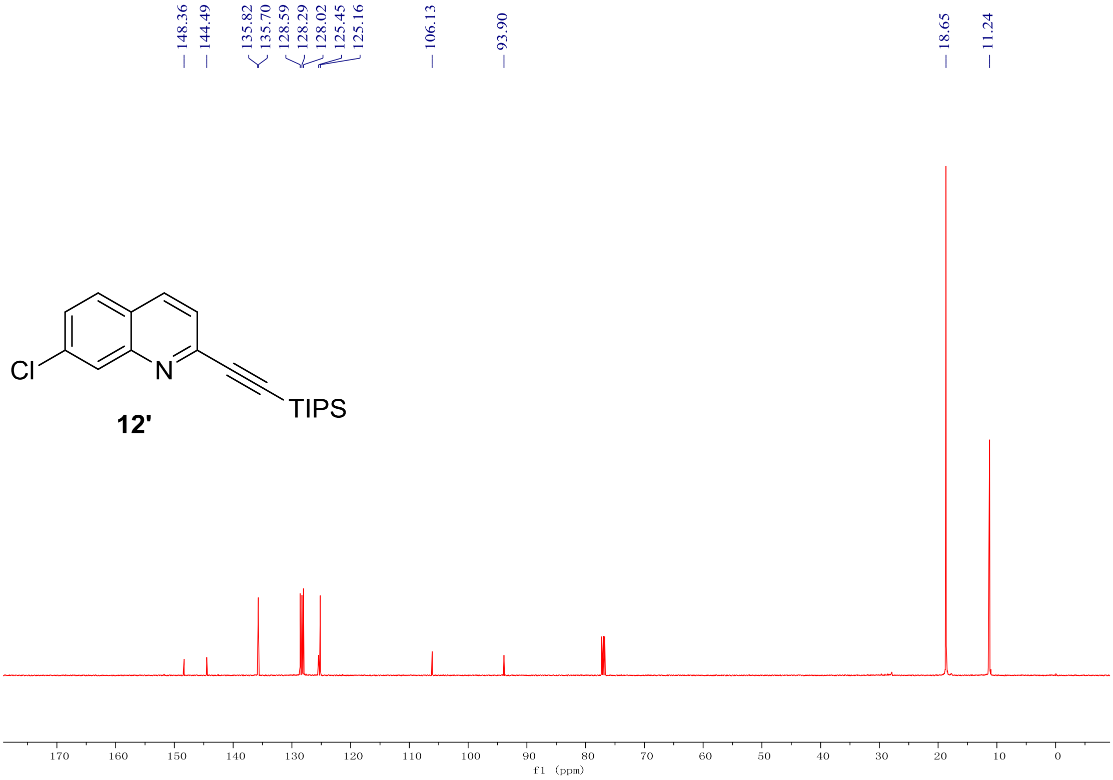

Supplementary Fig. 141. ^{13}C NMR spectra (126 MHz, $CDCl_3$, 25 °C) of **12'**

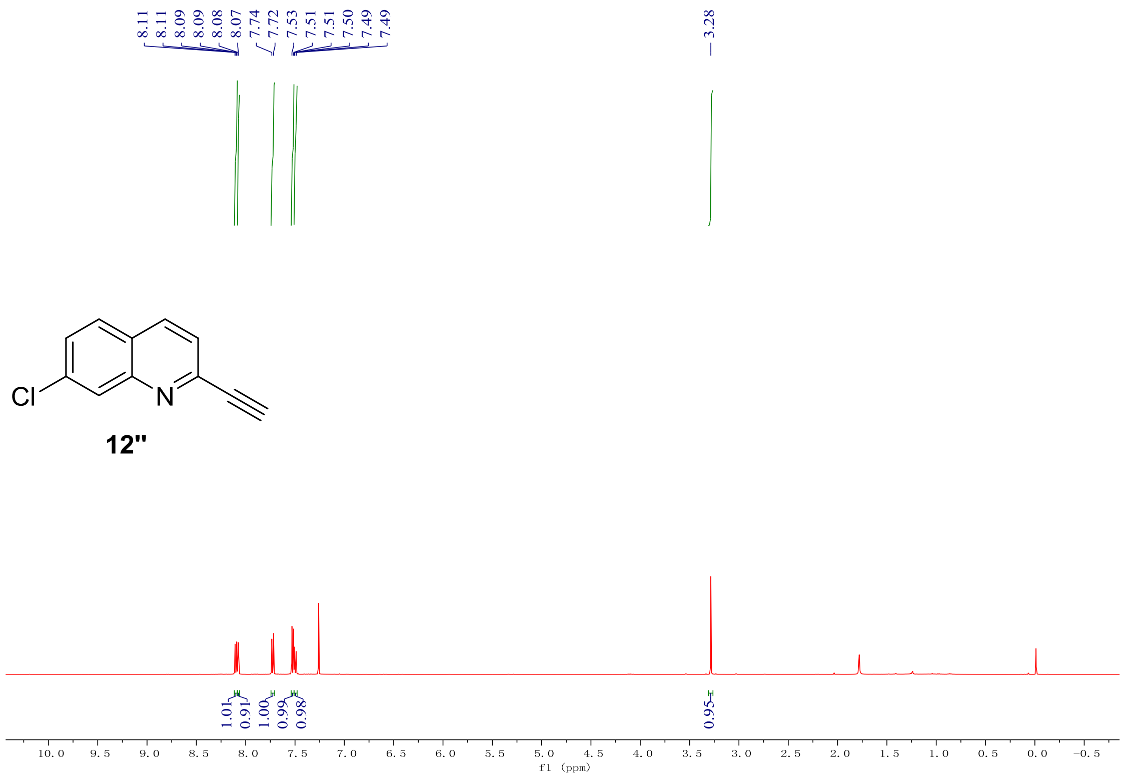

Supplementary Fig. 142. ^{1}H NMR spectra (500 MHz, $CDCl_3$, 25 °C) of **12''**

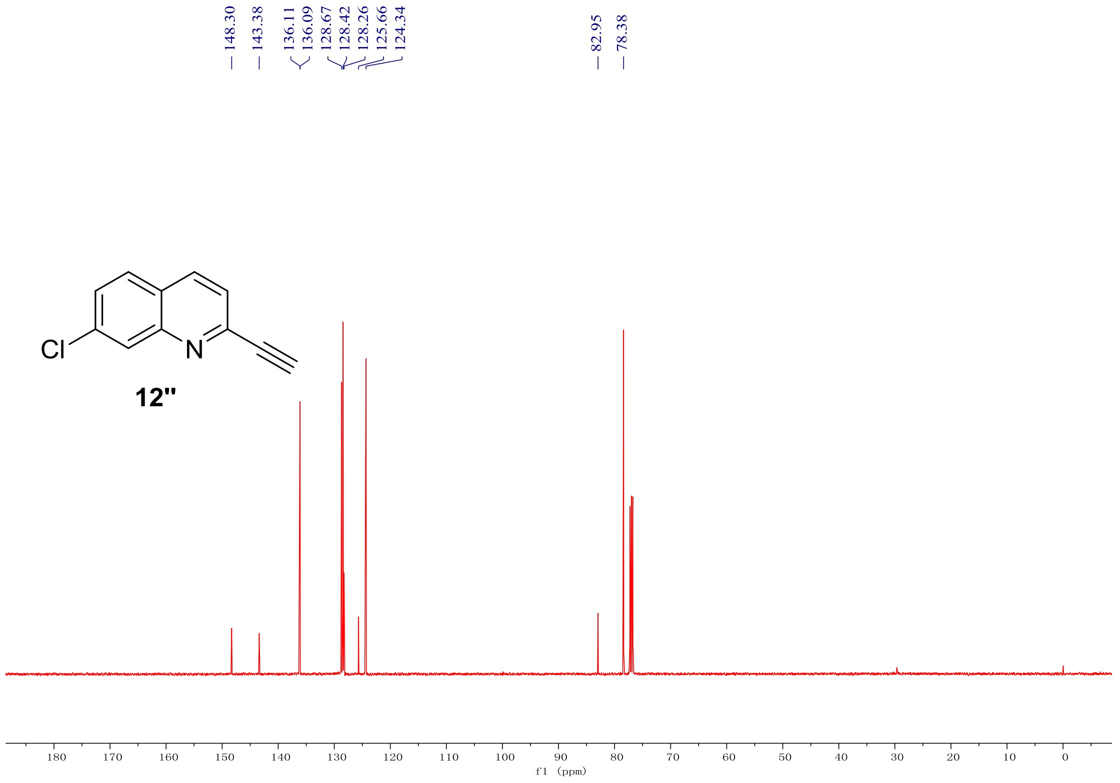

Supplementary Fig. 143. ^{13}C NMR spectra (126 MHz, $CDCl_3$, 25 °C) of **12''**

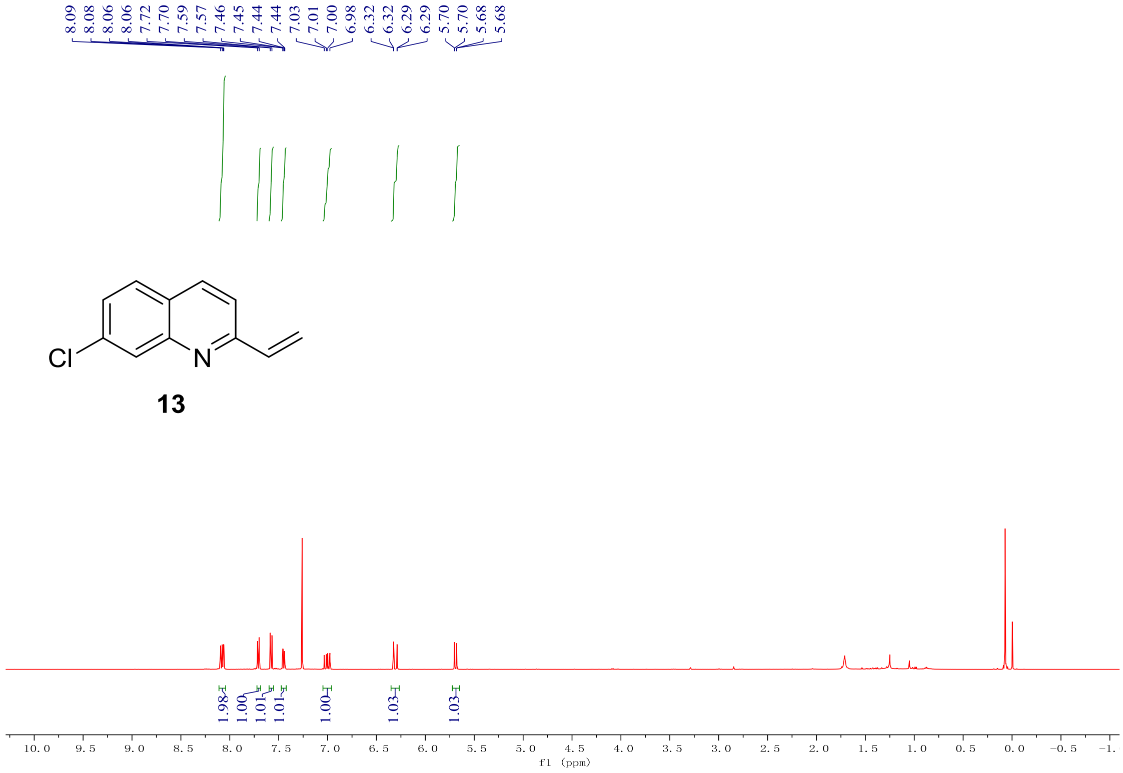

Supplementary Fig. 144. ^{1}H NMR spectra (500 MHz, $CDCl_3$, 25 ^{o}C) of **13**

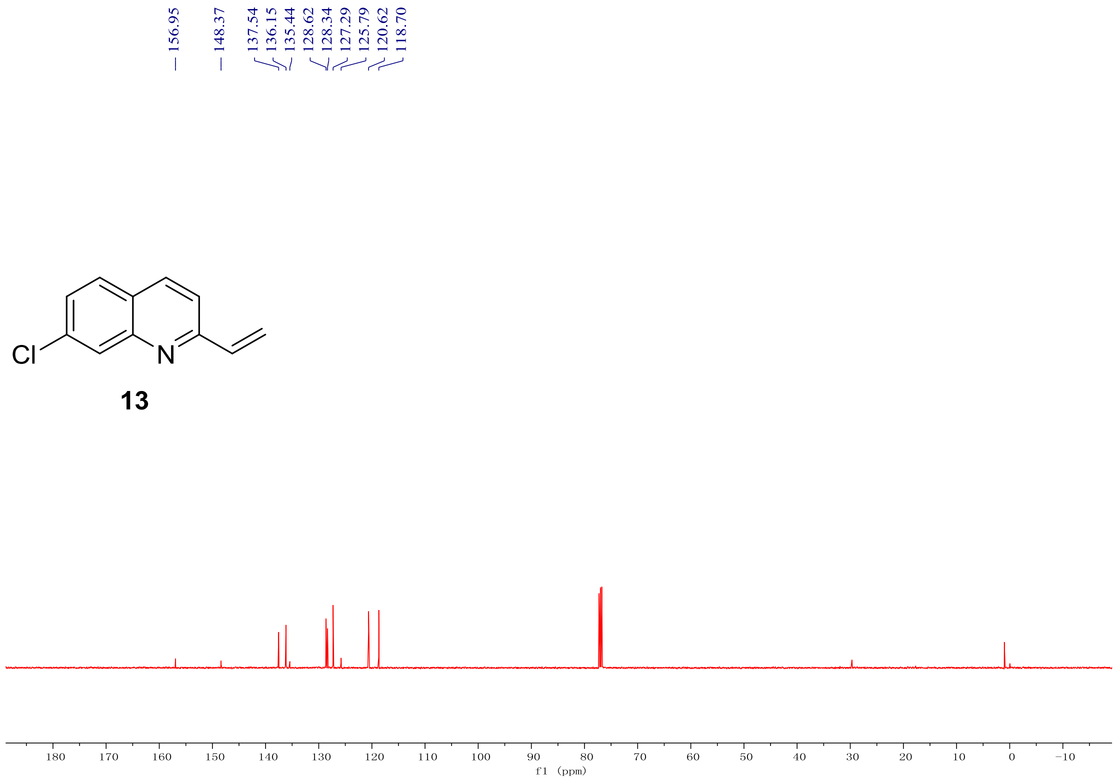

Supplementary Fig. 145. ^{13}C NMR spectra (126 MHz, $CDCl_3$, 25 ^{o}C) of **13**

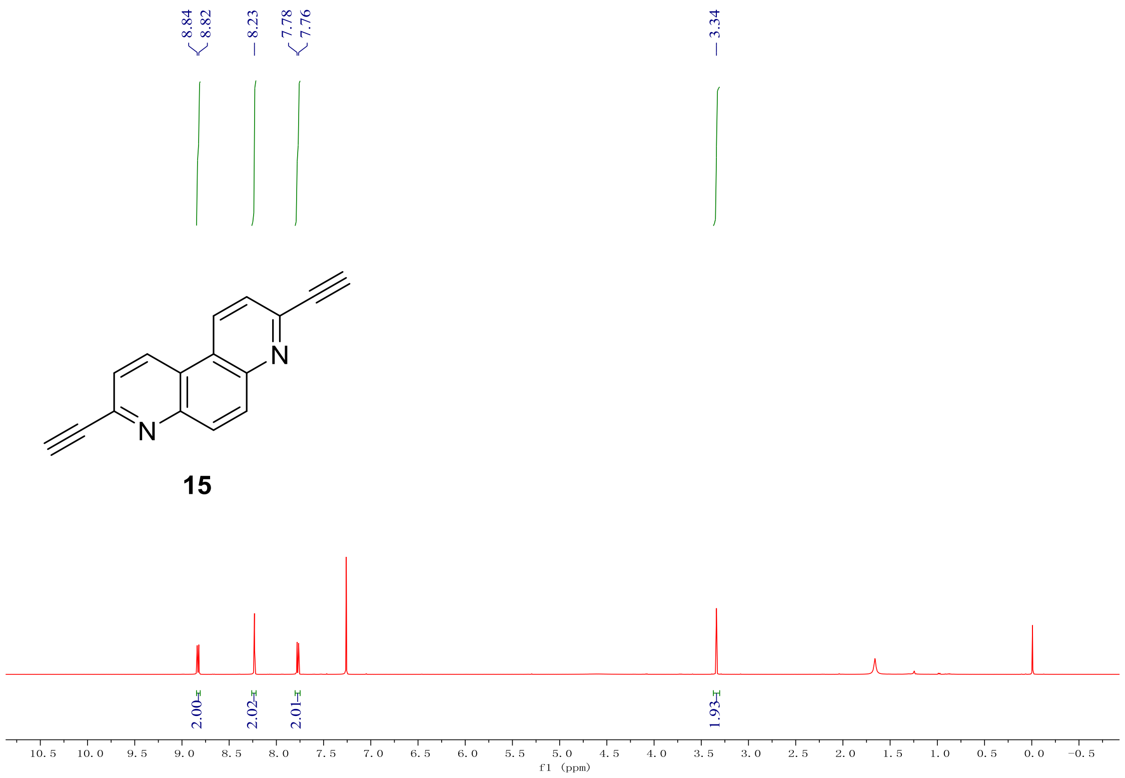

Supplementary Fig. 146. ^{1}H NMR spectra (500 MHz, $CDCl_3$, 25 ^{o}C) of **15**

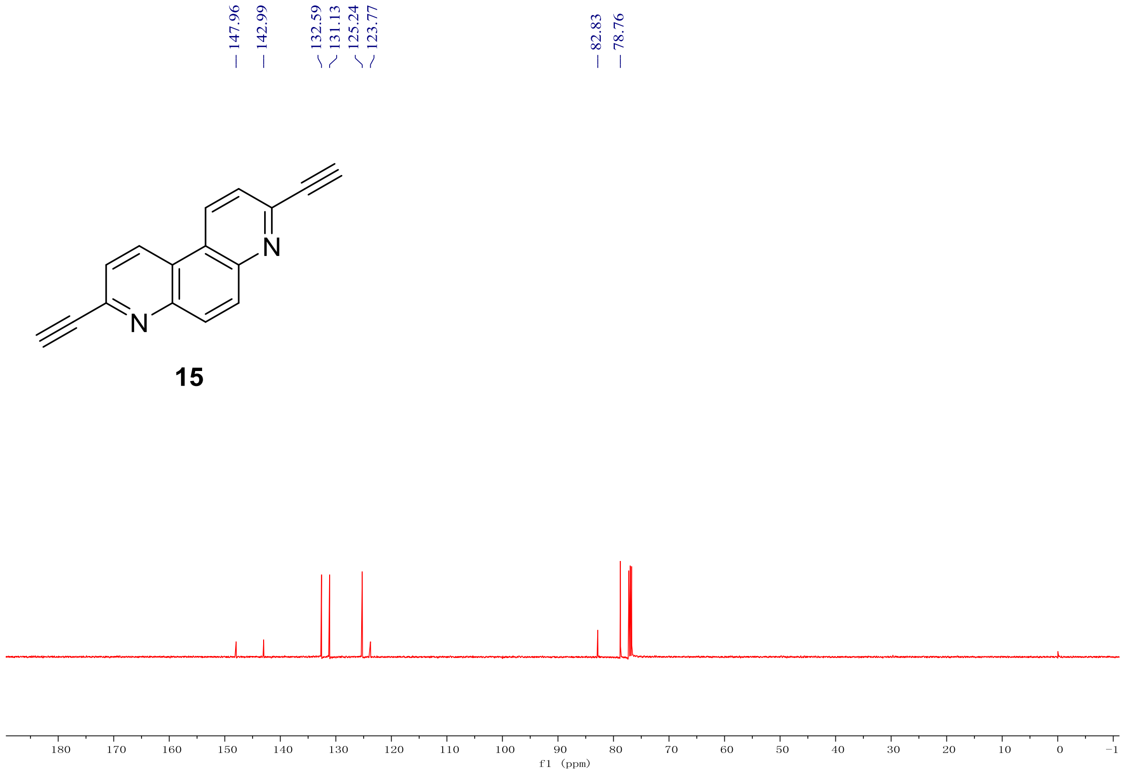

Supplementary Fig. 147. ^{13}C NMR spectra (126 MHz, $CDCl_3$, 25 ^{o}C) of **15**

3. Supplementary References

1. S. J. Proctor, J. Davis, J. Phipps, *Science*, **2018**, *360*, 419-422.
2. F. Berndt, M. Sajadi, N.P. Ernsting R. Mahrwald, *Carbohydrate Research*, **2011**, *346*, 2960-2964.
3. J. Kwak, M. Kim and S. Chang, *J. Am. Chem. Soc.* **2011**, *133*, 3780–3783.
4. S. Batsyts, R. Vedmid, J.C. Namyslo, M. Nieger, A. Schmidt, *Eur. J. Org. Chem.* **2019**, 1301–1310
5. Gaussian 09, Revision E.01, Frisch, M. J.; Trucks, G. W.; Schlegel, H. B.; Scuseria, G. E.; Robb, M. A.; Cheeseman, J. R.; Scalmani, G.; Barone, V.; Mennucci, B.; Petersson, G. A.; Nakatsuji, H.; Caricato, M.; Li, X.; Hratchian, H. P.; Izmaylov, A. F.; Bloino, J.; Zheng, G.; Sonnenberg, J. L.; Hada, M.; Ehara, M.; Toyota, K.; Fukuda, R.; Hasegawa, J.; Ishida, M.; Nakajima, T.; Honda, Y.; Kitao, O.; Nakai, H.; Vreven, T.; Montgomery, J. A.; Peralta, Jr., J. E.; Ogliaro, F.; Bearpark, M.; Heyd, J. J.; Brothers, E.; Kudin, K. N.; Staroverov, V. N.; Keith, T.; Kobayashi, R.; Normand, J.; Raghavachari, K.; Rendell, A.; Burant, J. C.; Iyengar, S. S.; Tomasi, J.; Cossi, M.; Rega, N.; Millam, J. M.; Klene, M.; Knox, J. E.; Cross, J. B.; Bakken, V.; Adamo, C.; Jaramillo, J.; Gomperts, R.; Stratmann, R. E.; Yazyev, O.; Austin, A. J.; Cammi, R.; Pomelli, C.; Ochterski, J. W.; Martin, R. L.; Morokuma, K.; Zakrzewski, V. G.; Voth, G. A.; Salvador, P.; Dannenberg, J. J.; Dapprich, S.; Daniels, A. D.; Farkas, O.; Foresman, J. B.; Ortiz, J. V.; Cioslowski, J.; and Fox, D. J. Gaussian, Inc., Wallingford CT, **2013**.
6. Becke, A. D. *J. Chem. Phys.* **1993**, 98, 5648.
7. Lee, C.; Yang, W.; Parr, R. G. *Phys. Rev. B*, **1988**, *37*, 785.
8. Dolg, M.; Wedig, U.; Stoll, H.; Preuss, H. *J. Chem. Phys.* **1987**, *86*, 866.
9. Nicklass, A.; Dolg, M.; Stoll, H.; Preuss, H. *J. Chem. Phys.* **1995**, *102*, 8942.
10. Ditchfield, R.; Hehre, W. J.; Pople, J. A. *J. Chem. Phys.* **1971**, *54*, 724.
11. Hehre, W. J.; Ditchfield, R.; Pople, J. A. *J. Chem. Phys.* **1972**, *56*, 2257.
12. Hariharan, P. C.; Pople, J. A. *Theor. Chem. Acc.* **1973**, *28*, 213.

13. Marenich, A. V.; Cramer, C. J.; Truhlar, D. G. *J. Phys. Chem. B.* **2009**, *113*, 6378.

14. Zhao, Y.; Truhlar, D. G. *Theor. Chem. Acc.* **2008**, *120*, 215.

15. Clark, T.; Chandrasekhar, J.; Spitznagel, G. W.; Schleyer, P. Von R. *J. Comput. Chem.* **1983**, *4*, 294.

16. Krishnan, R.; Binkley, J. S.; Seeger, R.; Pople, J. A. *J. Chem. Phys.* **1980**, *72*, 650.

17. Legault, C. Y. CYL View, version 1.0 b; Universite de Sherbrooke, Sherbrooke, Quebec, Canada, **2009**; http://www.cylview.org.

18. Ye, C.-X.; Chen, S.; Han, F.; Xie, X.; Ivlev, S.; Houk, K. N.; Meggers, E. *Angew. Chem. Int. Ed.* **2020**, *59*, 13552-13556.

19. Li, Y.; Chen, H.; Qu, L.-B.; Houk, K. N.; Lan, Y. *ACS Catal.* **2019**, *9*, 7154-7165.

20. Wang, X.; Li, Y.; Knecht, T.; Daniliuc, C.; Houk, K. N.; Glorius, F. *Angew. Chem. Int. Ed.* **2018**, *57*, 5520-5524.

21. Chen, S.; Huang, X.; Meggers, E.; Houk, K. N. *J. Am. Chem. Soc.* **2017**, *139*, 17902-17907.

22. Dziedzic, R. M.; Martin, J. L.; Axtell, J. C.; Saleh, L. M. A.; Ong, T.-C.; Yang, Y.-F.; Messina, M. S.; Rheingold, A. L.; Houk, K. N.; Spokoyny, A. M. *J. Am. Chem. Soc.* **2017**, *139*, 7729-7732.

23. Yang, Y.-F.; Houk, K. N.; Wu, Y.-D. *J. Am. Chem. Soc.* **2016**, *138*, 6861-6868.

24. L. Sun, H. Chen, B. Liu, J. Chang, L. Kong, F. Wang, Y. Lan, X. Li, *Angew. Chem. Int. Ed.* **2021**, *60*, 8391-8395.

25. Deng, L.; Fu, Y.; Lee, S. Y.; Wang, C.; Liu, P.; Dong, G. *J. Am. Chem. Soc.* **2019**, *141*, 16260-16265.
