# Supplementary material for: Tunably strained metallacycles enable modular differentiation of aza-arene C–H bonds
Source: Nat Commun. 2023 Jul 6;14:3986. doi: 10.1038/s41467-023-39753-2 (PMC10326034; doi:10.1038/s41467-023-39753-2)
Supplement: Supplementary file 2 — Description of Additional Supplementary Files [file 41467_2023_39753_MOESM2_ESM.docx]

File Name: Supplementary Data 1

Description: NPA charge analysis of **1a**

File Name: Supplementary Data 2

Description: Crystallographic Data
