# Supplementary material for: Tunably strained metallacycles enable modular differentiation of aza-arene C–H bonds
Source: Nat Commun. 2023 Jul 6;14:3986. doi: 10.1038/s41467-023-39753-2 (PMC10326034; doi:10.1038/s41467-023-39753-2)
Supplement: Supplementary file 3 — Supplementary Data 1 [file 41467_2023_39753_MOESM3_ESM.pdf]

NPA charge analysis of **1a**

Atom	No	Natural Charge	Natural Population: Core	Valence	Rydberg	Total
C	1	-0.20009	1.99916	4.18281	0.01811	6.20009
C	2	-0.19237	1.99908	4.17363	0.01966	6.19237
C	3	0.16238	1.99896	3.81376	0.02491	5.83762
C	4	-0.09717	1.99897	4.08180	0.01640	6.09717
C	5	-0.18382	1.99909	4.16680	0.01792	6.18382
C	6	-0.20523	1.99916	4.18779	0.01827	6.20523
H	7	0.21685	0.00000	0.78179	0.00136	0.78315
H	8	0.22565	0.00000	0.77212	0.00223	0.77435
C	9	-0.13925	1.99908	4.12236	0.01781	6.13925
H	10	0.21510	0.00000	0.78306	0.00183	0.78490
H	11	0.21647	0.00000	0.78215	0.00138	0.78353
C	12	-0.25351	1.99915	4.23721	0.01716	6.25351
C	13	0.06897	1.99925	3.90320	0.02858	5.93103
H	14	0.21814	0.00000	0.78006	0.00180	0.78186
H	15	0.22280	0.00000	0.77571	0.00149	0.77720
H	16	0.19407	0.00000	0.80453	0.00140	0.80593
N	17	-0.46899	1.99939	5.44582	0.02377	7.46899
* Total *		-0.00000	19.99130	47.79460	0.21410	68.00000

NPA charge analysis of **Rh-dtbpy**

Natural Population

Natural ---

Atom	No	Charge	Core	Valence	Rydberg	Total
C	1	0.29145	1.99909	3.67813	0.03133	5.70855
C	2	0.07924	1.99918	3.89789	0.02369	5.92076
C	3	-0.22257	1.99904	4.20766	0.01587	6.22257
C	4	0.01671	1.99904	3.94806	0.03619	5.98329
C	5	-0.24285	1.99909	4.21139	0.03236	6.24285
C	6	0.29264	1.99909	3.67674	0.03152	5.70736
C	7	0.08492	1.99918	3.89126	0.02463	5.91508
C	8	-0.22460	1.99903	4.20980	0.01576	6.22460
C	9	0.01936	1.99904	3.94554	0.03606	5.98064
C	10	-0.24548	1.99909	4.21441	0.03198	6.24548
H	11	0.21886	0.00000	0.77963	0.00151	0.78114
H	12	0.24275	0.00000	0.75523	0.00202	0.75725
H	13	0.23498	0.00000	0.76225	0.00277	0.76502
H	14	0.21735	0.00000	0.78118	0.00147	0.78265
H	15	0.24283	0.00000	0.75516	0.00201	0.75717
H	16	0.23494	0.00000	0.76229	0.00277	0.76506
C	17	0.00991	1.99914	3.96671	0.02424	5.99009
C	18	-0.59012	1.99935	4.56804	0.02273	6.59012
C	19	-0.59034	1.99935	4.56818	0.02281	6.59034
C	20	-0.58552	1.99936	4.56909	0.01708	6.58552
H	21	0.20982	0.00000	0.78862	0.00156	0.79018
H	22	0.20035	0.00000	0.79743	0.00221	0.79965
H	23	0.21467	0.00000	0.78324	0.00210	0.78533
H	24	0.20999	0.00000	0.78846	0.00155	0.79001
H	25	0.21474	0.00000	0.78316	0.00210	0.78526
H	26	0.19980	0.00000	0.79800	0.00219	0.80020

H	27	0.21531	0.00000	0.78255	0.00214	0.78469
H	28	0.20503	0.00000	0.79288	0.00209	0.79497
H	29	0.20509	0.00000	0.79282	0.00209	0.79491
C	30	0.00962	1.99914	3.96699	0.02425	5.99038
C	31	-0.59041	1.99935	4.56823	0.02283	6.59041
C	32	-0.59016	1.99935	4.56807	0.02274	6.59016
C	33	-0.58559	1.99936	4.56916	0.01707	6.58559
H	34	0.20992	0.00000	0.78853	0.00155	0.79008
H	35	0.19984	0.00000	0.79797	0.00219	0.80016
H	36	0.21471	0.00000	0.78319	0.00210	0.78529
H	37	0.20987	0.00000	0.78856	0.00157	0.79013
H	38	0.21458	0.00000	0.78332	0.00210	0.78542
H	39	0.20021	0.00000	0.79757	0.00222	0.79979
H	40	0.21514	0.00000	0.78271	0.00215	0.78486
H	41	0.20512	0.00000	0.79280	0.00209	0.79488
H	42	0.20514	0.00000	0.79277	0.00210	0.79486
N	43	-0.49600	1.99927	5.46121	0.03552	7.49600
N	44	-0.46920	1.99927	5.43663	0.03330	7.46920
Rh	45	0.53205	35.99033	8.46674	0.01087	44.46795

===

* Total *	1.04412	75.97413	113.40025	0.58150	189.95588

NPA charge analysis of **Rh-IMes**

Atom	No	Natural Charge	Natural Population Core	Valence	Rydberg	Total
Rh	1	0.58653	35.98796	8.42029	0.00522	44.41347

C	2	0.24901	1.99902	3.71625	0.03572	5.75099
C	3	-0.03249	1.99909	4.01370	0.01970	6.03249
C	4	-0.03586	1.99910	4.01718	0.01959	6.03586
H	5	0.24712	0.00000	0.75118	0.00170	0.75288
H	6	0.24800	0.00000	0.75039	0.00160	0.75200
N	7	-0.46356	1.99932	5.43566	0.02858	7.46356
N	8	-0.45903	1.99931	5.43116	0.02855	7.45903
C	9	0.23543	1.99871	3.74410	0.02176	5.76457
C	10	-0.05248	1.99906	4.02276	0.03067	6.05248
C	11	-0.05360	1.99905	4.02323	0.03131	6.05360
C	12	-0.21438	1.99895	4.19955	0.01587	6.21438
C	13	-0.21213	1.99895	4.19724	0.01594	6.21213
C	14	0.00556	1.99905	3.97837	0.01702	5.99444
H	15	0.21884	0.00000	0.77936	0.00180	0.78116
H	16	0.21862	0.00000	0.77964	0.00174	0.78138
C	17	0.24238	1.99870	3.73583	0.02309	5.75762
C	18	-0.05297	1.99906	4.02353	0.03038	6.05297
C	19	-0.05562	1.99906	4.02595	0.03061	6.05562
C	20	-0.20983	1.99895	4.19503	0.01584	6.20983
C	21	-0.21054	1.99896	4.19598	0.01560	6.21054
C	22	0.00240	1.99905	3.98164	0.01691	5.99760
H	23	0.21771	0.00000	0.78051	0.00178	0.78229
H	24	0.21719	0.00000	0.78098	0.00183	0.78281
C	25	-0.59950	1.99930	4.59041	0.00979	6.59950
H	26	0.21214	0.00000	0.78615	0.00170	0.78786
H	27	0.22449	0.00000	0.77399	0.00152	0.77551
H	28	0.22281	0.00000	0.77579	0.00140	0.77719
C	29	-0.59618	1.99930	4.58712	0.00976	6.59618
H	30	0.21949	0.00000	0.77896	0.00155	0.78051

H	31	0.21542	0.00000	0.78300	0.00158	0.78458
H	32	0.22292	0.00000	0.77571	0.00137	0.77708
C	33	-0.59971	1.99930	4.59008	0.01032	6.59971
H	34	0.21877	0.00000	0.77998	0.00125	0.78123
H	35	0.22496	0.00000	0.77377	0.00127	0.77504
H	36	0.21526	0.00000	0.78353	0.00121	0.78474
C	37	-0.60035	1.99930	4.59090	0.01016	6.60035
H	38	0.21341	0.00000	0.78528	0.00131	0.78659
H	39	0.22956	0.00000	0.76914	0.00130	0.77044
H	40	0.22268	0.00000	0.77584	0.00148	0.77732
C	41	-0.59859	1.99929	4.58926	0.01004	6.59859
H	42	0.22323	0.00000	0.77524	0.00153	0.77677
H	43	0.21417	0.00000	0.78392	0.00191	0.78583
H	44	0.22381	0.00000	0.77483	0.00136	0.77619
C	45	-0.59950	1.99930	4.58988	0.01032	6.59950
H	46	0.22339	0.00000	0.77533	0.00128	0.77661
H	47	0.22152	0.00000	0.77725	0.00124	0.77848
H	48	0.21644	0.00000	0.78236	0.00120	0.78356

===

* Total *	1.00692	81.96715	125.52723	0.49870	207.99308

DFT-Computed Energies and Cartesian Coordinate (unit:angstrom)

INT1A-dtbpy

G_{sol}(toluene) = -1554.242770 Hartree

C	2.022330	-0.275273	-0.101643
C	1.590394	-2.556948	-0.282671
C	2.938276	-2.863055	-0.143979
C	3.882620	-1.841824	0.039727
C	3.384683	-0.532581	0.050152
C	1.418687	1.063077	-0.161067
C	-0.536309	2.266374	-0.505120
C	0.139464	3.475269	-0.412685
C	1.520395	3.500622	-0.169507

C	2.140253	2.250588	-0.053464	H	4.130716	3.959943	-1.021136
H	0.818656	-3.305430	-0.428098	H	2.094852	6.944110	-0.105098
H	3.230812	-3.905327	-0.181841	H	1.002564	6.088035	-1.199961
H	4.067603	0.299800	0.170970	H	0.682124	6.106086	0.548147
H	-1.602225	2.243565	-0.693236	N	0.059944	1.062535	-0.366786
H	-0.432377	4.387199	-0.534774	N	1.126940	-1.294672	-0.247507
H	3.208515	2.197464	0.120950	Rh	-0.820815	-0.775686	-0.416631
C	5.386705	-2.096368	0.208563	C	-3.532935	0.598331	0.198332
C	6.151381	-1.402807	-0.942901	C	-3.383560	-0.598978	-1.796389
C	5.854332	-1.511557	1.561479	C	-2.960511	0.997450	1.432842
C	5.727341	-3.597099	0.184212	C	-4.872304	0.995526	-0.109889
H	5.832216	-1.791660	-1.916848	C	-4.699943	-0.251338	-2.163048
H	5.991714	-0.318854	-0.945476	H	-2.783063	-1.245354	-2.425208
H	7.229161	-1.579589	-0.842193	C	-3.679260	1.776337	2.314075
H	5.325093	-1.983376	2.397507	H	-1.951848	0.659334	1.653196
H	6.928790	-1.685450	1.696874	C	-5.582529	1.801918	0.819583
H	5.682924	-0.431212	1.622583	C	-5.442412	0.552851	-1.330095
H	6.806925	-3.731910	0.315673	H	-5.102389	-0.626870	-3.098330
H	5.225631	-4.141748	0.992377	C	-4.999161	2.187699	2.005601
H	5.450529	-4.062844	-0.768630	H	-3.231433	2.072515	3.258702
C	2.340591	4.790094	-0.038200	H	-6.599340	2.100972	0.577178
C	2.994878	4.834690	1.362232	H	-6.458507	0.844248	-1.583221
C	3.444171	4.807941	-1.121008	H	-5.552173	2.800113	2.712397
C	1.471766	6.048752	-0.210035	N	-2.796655	-0.178953	-0.675746
H	2.234169	4.824436	2.151595	O	-1.391066	-2.731541	-0.543985
H	3.662380	3.982157	1.528975	C	-2.021043	-3.477218	0.462907
H	3.589177	5.749806	1.474028	C	-1.229497	-3.426148	1.790843
H	3.009167	4.770417	-2.126533	C	-3.466482	-3.003800	0.740183
H	4.037880	5.726728	-1.041018	C	-2.069360	-4.936485	-0.036569

H	-0.211021	-3.806862	1.648065
H	-1.152714	-2.388708	2.137606
H	-1.707495	-4.024580	2.578815
H	-4.044838	-2.988204	-0.190823
H	-3.978775	-3.665358	1.452308
H	-3.468778	-1.993054	1.159314
H	-2.543676	-5.609872	0.690033
H	-2.631702	-4.990400	-0.976533
H	-1.053439	-5.300703	-0.232562

2a

G_{sol}(toluene) = -3294.819945 Hartree

C	-1.957112	0.108033	-0.029806
C	-0.738900	0.096470	-0.012484
Br	-3.750824	0.122264	-0.056586
Si	1.108244	0.065964	0.027521
C	1.616845	-1.093833	1.453991
H	2.715883	-1.146992	1.422458
C	1.669718	1.854567	0.386080
H	1.179764	2.118768	1.335169
C	1.747990	-0.545172	-1.669604
H	1.800953	0.357517	-2.297181
C	0.803163	-1.538101	-2.370910
H	-0.199904	-1.120329	-2.509783
H	1.194139	-1.810717	-3.361310
H	0.693499	-2.467292	-1.798390
C	3.171820	-1.127663	-1.568904
H	3.555646	-1.384769	-2.565874
H	3.884278	-0.427963	-1.116483
H	3.187911	-2.046862	-0.970164
C	1.066950	-2.520368	1.273329
H	1.427310	-2.990376	0.351281
H	1.370857	-3.164711	2.110234
H	-0.029845	-2.521912	1.239559
C	1.205368	-0.522789	2.823256
H	0.116265	-0.404652	2.893630
H	1.513509	-1.195949	3.635412
H	1.658973	0.455826	3.019931
C	3.191192	1.970247	0.590547
H	3.737221	1.748522	-0.334963
H	3.468045	2.990671	0.889828
H	3.562218	1.289688	1.366789
C	1.181023	2.848812	-0.681793
H	0.093799	2.803524	-0.816863
H	1.438697	3.880126	-0.403236
H	1.645863	2.653918	-1.657049

INT2A-dtbpy

G_{sol}(toluene) = -4849.064693 Hartree

C	2.352824	-0.369838	-0.509344
C	1.495138	-2.484084	-0.081283
C	2.765797	-3.012656	0.115133
C	3.893741	-2.198697	-0.026302
C	3.648585	-0.857687	-0.347532
C	2.056965	1.061129	-0.740236
C	0.448352	2.642445	-1.219534

C	1.347722	3.691244	-1.047364	H	4.619944	4.370657	-2.410069
C	2.671303	3.418265	-0.685152	H	5.684577	5.068294	-1.173087
C	3.006166	2.065210	-0.541075	H	5.401198	3.322910	-1.214708
H	0.615244	-3.103696	0.015746	H	3.941003	6.660661	-0.440028
H	2.844815	-4.062722	0.367109	H	2.824128	6.065388	-1.674438
H	4.482446	-0.178526	-0.473031	H	2.319466	6.137862	0.028935
H	-0.585696	2.843708	-1.475795	N	0.774556	1.355443	-1.063194
H	0.990469	4.704786	-1.181663	N	1.279353	-1.197113	-0.393635
H	4.008631	1.794153	-0.234693	C	-3.599278	0.849203	-1.057211
C	5.330738	-2.694376	0.172142	C	-2.021310	1.216023	-2.730130
C	6.139253	-2.448156	-1.122272	C	-4.053993	0.168224	0.099025
C	5.970354	-1.908710	1.340933	C	-4.494162	1.784007	-1.682047
C	5.383989	-4.195776	0.504512	C	-2.859127	2.125250	-3.407291
H	5.701255	-2.989960	-1.968395	H	-1.062145	0.914128	-3.143903
H	6.178290	-1.386129	-1.387856	C	-5.304581	0.419511	0.623378
H	7.171101	-2.795763	-0.992447	H	-3.400282	-0.546545	0.569025
H	5.406547	-2.056859	2.269360	C	-5.770368	2.027469	-1.108638
H	6.998606	-2.251326	1.507324	C	-4.085893	2.430075	-2.873810
H	6.006861	-0.832625	1.138404	H	-2.509196	2.568567	-4.334102
H	6.426503	-4.506896	0.634027	C	-6.171850	1.362853	0.027067
H	4.853516	-4.427033	1.435445	H	-5.622898	-0.120633	1.510951
H	4.954209	-4.805430	-0.298597	H	-6.427278	2.743972	-1.595713
C	3.719537	4.504408	-0.415104	H	-4.754809	3.140855	-3.352877
C	4.186367	4.390429	1.055275	H	-7.151438	1.550570	0.457498
C	4.925834	4.299064	-1.359861	N	-2.337653	0.615380	-1.581707
C	3.159199	5.919799	-0.640912	O	-0.064837	-0.802918	-2.888621
H	3.345471	4.525156	1.745637	C	-0.508002	-1.923303	-3.604307
H	4.641249	3.416639	1.267072	C	-2.048906	-1.964203	-3.726353
H	4.934163	5.162146	1.273863	C	0.107843	-1.817694	-5.014854

C	-0.041877	-3.242140	-2.951272
H	-2.501476	-1.988243	-2.728926
H	-2.420662	-1.073050	-4.246760
H	-2.391427	-2.848300	-4.281186
H	1.202122	-1.804175	-4.942385
H	-0.188104	-2.654080	-5.662708
H	-0.209567	-0.882389	-5.492105
H	-0.296938	-4.116815	-3.565439
H	1.045081	-3.226041	-2.806764
H	-0.521222	-3.365750	-1.974616
Rh	-0.554325	-0.428792	-0.843974
Br	-2.287519	-3.379319	0.639137
C	-1.427851	-1.644156	0.467059
C	-1.115290	-0.662659	1.240136
Si	-0.929688	0.292393	2.795085
C	-1.433680	-0.835823	4.263110
H	-1.218078	-0.296512	5.199179
C	-2.144424	1.774474	2.705411
H	-3.102938	1.309020	2.435776
C	0.887363	0.914595	2.820169
H	0.998896	1.336344	1.810218
C	1.935606	-0.204486	2.963173
H	1.721147	-1.066925	2.324590
H	2.937780	0.164330	2.699784
H	1.991215	-0.563032	3.998488
C	1.182294	2.038866	3.827990
H	2.240366	2.337677	3.779449
H	0.584768	2.935743	3.635696
H	0.985862	1.723611	4.861536
C	-0.664169	-2.170466	4.280932
H	0.415156	-2.033222	4.398106
H	-1.006276	-2.807813	5.109676
H	-0.830185	-2.728003	3.350658
C	-2.948370	-1.124613	4.208265
H	-3.209180	-1.652135	3.281189
H	-3.255234	-1.769525	5.044337
H	-3.556557	-0.214162	4.257002
C	-2.359534	2.521445	4.034269
H	-1.462611	3.064909	4.352425
H	-3.165651	3.263361	3.936833
H	-2.636983	1.845355	4.851799
C	-1.788862	2.746656	1.567601
H	-1.695452	2.219106	0.614260
H	-2.563296	3.519414	1.450098
H	-0.838784	3.263954	1.755182

TS3A-dtbpy

G_{sol}(toluene) = -4849.020632 Hartree

C	2.102051	1.423599	-0.194786
C	0.051463	2.240742	0.505399
C	0.574335	3.504382	0.752114
C	1.928183	3.756985	0.512756
C	2.674376	2.678051	0.020432
C	2.917929	0.305553	-0.735201
C	3.009580	-1.526115	-2.101309
C	4.362689	-1.736299	-1.829607
C	5.030067	-0.873344	-0.951438

C	4.270765	0.173323	-0.412578	H	6.191123	-0.394719	1.526044
H	-0.995229	2.033211	0.676975	H	8.214076	-2.311959	-0.948167
H	-0.096120	4.269980	1.121412	H	6.675345	-3.180759	-0.993650
H	3.720394	2.811635	-0.228890	H	7.150350	-2.133357	-2.348952
H	2.476936	-2.183977	-2.786017	N	2.288454	-0.540213	-1.567611
H	4.869073	-2.568084	-2.304546	N	0.782530	1.210650	0.049388
H	4.718067	0.861028	0.296101	C	-1.672272	-3.300963	0.570462
C	2.591472	5.119765	0.741111	C	0.407518	-3.514721	-0.462406
C	3.756044	4.953459	1.744840	C	-2.661908	-2.497967	1.188568
C	3.141728	5.644508	-0.605559	C	-1.850332	-4.722096	0.543477
C	1.603564	6.157010	1.303560	C	0.296572	-4.917155	-0.530029
H	3.396004	4.568539	2.706058	H	1.297073	-3.013927	-0.820709
H	4.522103	4.264869	1.372409	C	-3.777899	-3.079708	1.750242
H	4.238484	5.921644	1.924427	H	-2.523949	-1.425545	1.205741
H	2.335990	5.766276	-1.338632	C	-3.021863	-5.285063	1.116124
H	3.621508	6.619868	-0.460317	C	-0.834392	-5.521094	-0.035500
H	3.887404	4.966987	-1.035036	H	1.107429	-5.489957	-0.967513
H	2.119212	7.111658	1.455970	C	-3.967427	-4.481798	1.710954
H	0.769490	6.339075	0.616006	H	-4.526116	-2.450648	2.222941
H	1.192330	5.845620	2.270852	H	-3.148539	-6.364170	1.083260
C	6.504868	-1.035019	-0.558874	H	-0.958257	-6.600462	-0.068054
C	7.279597	0.244273	-0.950867	H	-4.857795	-4.918993	2.153699
C	6.599155	-1.245430	0.970206	N	-0.535930	-2.727496	0.042491
C	7.165480	-2.236553	-1.257589	O	1.611511	-1.241285	1.238958
H	7.228739	0.420278	-2.031898	C	1.573667	-1.230569	2.633284
H	6.883858	1.132582	-0.446470	C	0.590529	-2.286403	3.185815
H	8.335791	0.147209	-0.671444	C	3.000117	-1.567690	3.120258
H	6.047444	-2.140275	1.279091	C	1.171724	0.156982	3.183723
H	7.647271	-1.368146	1.269900	H	-0.433658	-2.049358	2.875635

H	0.840673	-3.276558	2.786787
H	0.609807	-2.338934	4.283129
H	3.708832	-0.818965	2.747909
H	3.070930	-1.599185	4.215905
H	3.303940	-2.543641	2.723610
H	1.199288	0.193628	4.281304
H	1.846334	0.928090	2.793686
H	0.152784	0.407682	2.861473
Rh	-0.011984	-0.712981	-0.012952
Br	-0.910448	-0.846548	-2.883287
C	-1.611877	-0.115560	-0.983245
C	-2.673001	0.506863	-0.729971
Si	-4.090459	1.587448	-0.346157
C	-5.718468	0.660660	-0.722313
H	-6.537297	1.331223	-0.419060
C	-3.942596	3.118925	-1.490213
H	-3.987874	2.705980	-2.508914
C	-4.005642	2.131858	1.500328
H	-3.350913	3.018078	1.502666
C	-3.376267	1.093553	2.446395
H	-2.371261	0.797155	2.127647
H	-3.298929	1.491942	3.468371
H	-3.982086	0.180278	2.501771
C	-5.383653	2.570026	2.035459
H	-5.294635	2.978922	3.052002
H	-5.855170	3.339488	1.414003
H	-6.077323	1.721671	2.089085
C	-5.836415	-0.641587	0.087875
H	-5.798947	-0.462613	1.169023
H	-6.784454	-1.156402	-0.125648
H	-5.023766	-1.333163	-0.161780
C	-5.867523	0.370384	-2.226944
H	-5.042748	-0.255112	-2.592746
H	-6.803224	-0.169365	-2.431661
H	-5.880329	1.287380	-2.827846
C	-5.107569	4.112601	-1.329314
H	-5.081900	4.601913	-0.347218
H	-5.049882	4.908756	-2.085140
H	-6.088046	3.631980	-1.435332
C	-2.589837	3.836690	-1.347570
H	-1.750026	3.160296	-1.537990
H	-2.512322	4.673866	-2.056168
H	-2.460591	4.257857	-0.341007

INT3A-dtbpy

G_{sol}(toluene) = -4849.116642 Hartree

C	2.531536	1.213877	-0.183499
C	0.574888	2.427397	-0.522584
C	1.274862	3.628439	-0.561775
C	2.660256	3.639951	-0.373553
C	3.274759	2.391616	-0.198298
C	3.138409	-0.135016	-0.110156
C	2.744307	-2.412596	-0.237349
C	4.106461	-2.701744	-0.215699
C	5.035338	-1.660441	-0.116639
C	4.513313	-0.359428	-0.075263
H	-0.501132	2.387589	-0.635499

H	0.712713	4.538658	-0.726269	H	6.502706	-3.936375	0.702975
H	4.348836	2.337383	-0.075238	H	6.582947	-3.836056	-1.070418
H	2.005912	-3.201747	-0.322032	N	2.267562	-1.169859	-0.162176
H	4.414697	-3.737722	-0.278416	N	1.179878	1.252976	-0.312693
H	5.189509	0.484538	-0.027230	C	-1.645656	-3.128436	-0.162662
C	3.501733	4.921492	-0.359007	C	0.041701	-3.048181	1.452871
C	4.221816	5.033006	1.005143	C	-2.410018	-2.530181	-1.194674
C	4.549093	4.854725	-1.494482	C	-1.999496	-4.452947	0.266266
C	2.641323	6.181486	-0.560899	C	-0.257479	-4.341462	1.921648
H	3.499679	5.076790	1.828636	H	0.801035	-2.439291	1.930476
H	4.889730	4.184364	1.188854	C	-3.450176	-3.217563	-1.782445
H	4.828467	5.945838	1.034669	H	-2.175306	-1.526559	-1.507431
H	4.063919	4.761194	-2.472804	C	-3.075726	-5.132055	-0.365268
H	5.153702	5.769405	-1.499880	C	-1.270369	-5.048618	1.322301
H	5.232760	4.006798	-1.375798	H	0.317442	-4.747665	2.747379
H	3.281164	7.070391	-0.534045	C	-3.788410	-4.528815	-1.374593
H	2.127722	6.174762	-1.529113	H	-4.022561	-2.733315	-2.568260
H	1.889260	6.292515	0.228764	H	-3.324361	-6.134760	-0.026859
C	6.552591	-1.880741	-0.070252	H	-1.530338	-6.052293	1.649573
C	7.208263	-1.158660	-1.270553	H	-4.612511	-5.050511	-1.853062
C	7.104972	-1.295239	1.250338	N	-0.596103	-2.457651	0.444045
C	6.923902	-3.372579	-0.137450	O	0.755919	-0.359290	1.941426
H	6.822053	-1.543794	-2.221217	C	0.087324	0.272843	3.012363
H	7.031268	-0.077808	-1.248738	C	-1.257355	-0.406756	3.332701
H	8.293070	-1.316930	-1.254234	C	1.041881	0.103225	4.212452
H	6.657934	-1.792384	2.119015	C	-0.122765	1.774363	2.748175
H	8.190982	-1.437928	1.297816	H	-1.928790	-0.338917	2.474245
H	6.906341	-0.221569	1.339015	H	-1.101276	-1.466850	3.566509
H	8.013105	-3.482049	-0.094482	H	-1.748622	0.063786	4.194638

H	2.008329	0.572708	3.994454
H	0.628635	0.559882	5.120936
H	1.217963	-0.960768	4.410313
H	-0.562323	2.277240	3.619372
H	0.837134	2.253897	2.522171
H	-0.790890	1.916309	1.896756
Rh	0.214132	-0.530868	-0.029560
Br	0.171991	-0.802227	-2.590741
C	-1.575901	0.270904	0.044801
C	-2.689775	0.797139	0.056583
Si	-4.383837	1.460664	0.017222
C	-4.914473	1.623540	-1.815506
H	-5.968339	1.942132	-1.815810
C	-4.377241	3.198747	0.825519
H	-3.622776	3.767482	0.259567
C	-5.578772	0.301934	0.981856
H	-5.556789	0.670656	2.019271
C	-5.126182	-1.168786	1.013215
H	-4.118075	-1.275196	1.426777
H	-5.810293	-1.775023	1.625820
H	-5.108827	-1.610857	0.010352
C	-7.034011	0.408329	0.485120
H	-7.708782	-0.185232	1.118955
H	-7.407386	1.439276	0.487843
H	-7.136466	0.027218	-0.538647
C	-4.818498	0.278872	-2.558787
H	-5.427463	-0.501909	-2.087916
H	-5.154978	0.376863	-3.601385
H	-3.780932	-0.075416	-2.579811
C	-4.088222	2.694060	-2.550370
H	-3.021431	2.434134	-2.547061
H	-4.398581	2.781574	-3.601824
H	-4.192986	3.686258	-2.094404
C	-5.721663	3.936502	0.693952
H	-6.509347	3.434178	1.270204
H	-5.649519	4.963571	1.080782
H	-6.064472	4.001973	-0.346042
C	-3.922285	3.161746	2.295204
H	-2.950885	2.669102	2.412115
H	-3.833887	4.177661	2.707918
H	-4.642857	2.622913	2.924673

TS2B-dtbpy

G_{sol}(toluene) = -1554.200949 Hartree

C	2.220023	-0.144152	-0.135265
C	1.960434	-2.440440	-0.384385
C	3.335876	-2.644878	-0.351045
C	4.202912	-1.555656	-0.190575
C	3.606364	-0.289731	-0.094770
C	1.512043	1.152090	-0.090996
C	-0.532864	2.241946	-0.240901
C	0.070740	3.485181	-0.080486
C	1.455149	3.583103	0.093540
C	2.160483	2.371785	0.090423
H	1.241894	-3.244309	-0.508166
H	3.707183	-3.657214	-0.450070
H	4.231467	0.589408	0.001652

H	-1.609119	2.130438	-0.331748	N	0.155165	1.087233	-0.252306
H	-0.565577	4.361586	-0.088968	N	1.410480	-1.225373	-0.261217
H	3.233876	2.378963	0.235030	C	-4.556216	0.875582	-0.048276
C	5.729992	-1.691893	-0.128304	C	-2.715393	-0.434969	-0.625183
C	6.359486	-0.903732	-1.300445	C	-5.056301	2.082460	0.509172
C	6.235339	-1.111410	1.213358	C	-5.486027	-0.116341	-0.495371
C	6.185531	-3.158654	-0.226016	C	-3.584574	-1.461252	-1.129515
H	6.010620	-1.287892	-2.265921	C	-6.414446	2.292783	0.620164
H	6.116799	0.163588	-1.255290	H	-4.338348	2.825228	0.846172
H	7.451706	-0.997332	-1.271428	C	-6.876581	0.129350	-0.370356
H	5.799802	-1.648422	2.063985	C	-4.943284	-1.302373	-1.060492
H	7.326135	-1.204886	1.274685	H	-3.128286	-2.362802	-1.519671
H	5.987024	-0.050036	1.322816	C	-7.333744	1.309857	0.177568
H	7.278404	-3.208881	-0.166045	H	-6.787080	3.218835	1.050390
H	5.781756	-3.766130	0.592103	H	-7.576008	-0.629968	-0.713440
H	5.886655	-3.616257	-1.175996	H	-5.617183	-2.074932	-1.426183
C	2.199439	4.910650	0.282532	H	-8.401188	1.490513	0.271862
C	2.926541	4.894289	1.647048	N	-3.203836	0.706841	-0.149025
C	3.236999	5.074329	-0.852506	O	-1.060751	-2.835918	-0.334363
C	1.246441	6.118571	0.251011	C	-1.324174	-3.463531	0.903933
H	2.214363	4.772688	2.471363	C	-0.455568	-2.900596	2.056424
H	3.658043	4.081379	1.711703	C	-2.805550	-3.338953	1.323453
H	3.464356	5.837932	1.797872	C	-0.990710	-4.956771	0.712552
H	2.748871	5.083766	-1.833894	H	0.612433	-3.012104	1.841226
H	3.779569	6.019659	-0.732718	H	-0.662164	-1.831525	2.207831
H	3.975448	4.265218	-0.851585	H	-0.663908	-3.416816	3.003137
H	1.819903	7.042290	0.386858	H	-3.459880	-3.756810	0.551020
H	0.717945	6.196427	-0.706063	H	-3.001576	-3.874460	2.262290
H	0.501837	6.071867	1.053975	H	-3.079892	-2.288960	1.466316

H	-1.205147	-5.545484	1.614365
H	-1.580279	-5.365582	-0.116221
H	0.070253	-5.084769	0.465167
Rh	-0.661826	-0.826806	-0.370617
H	-1.615344	-0.331380	-1.545649

INT2B-dtbpy

G_{sol}(toluene) = -1554.212777 Hartree

C	2.243714	-0.245600	0.047797
C	1.950442	-2.541239	0.233209
C	3.317218	-2.750551	0.400862
C	4.196070	-1.659964	0.386098
C	3.622702	-0.392286	0.203679
C	1.562360	1.059184	-0.140594
C	-0.441367	2.179446	-0.491285
C	0.193928	3.416023	-0.495874
C	1.578334	3.494426	-0.311049
C	2.244870	2.274781	-0.134578
H	1.220344	-3.346096	0.235070
H	3.671897	-3.764347	0.539322
H	4.261744	0.481637	0.182601
H	-1.513422	2.106336	-0.624848
H	-0.413777	4.300427	-0.640987
H	3.316628	2.272274	0.016583
C	5.715705	-1.795406	0.555480
C	6.415336	-1.256355	-0.713927
C	6.164980	-0.969044	1.783102
C	6.147947	-3.257028	0.767577
H	6.111352	-1.823059	-1.601612
H	6.183899	-0.200729	-0.893788
H	7.503335	-1.344575	-0.609854
H	5.676312	-1.325050	2.697410
H	7.249132	-1.058678	1.919818
H	5.932758	0.095624	1.670151
H	7.235857	-3.304551	0.888148
H	5.696938	-3.689026	1.668312
H	5.883943	-3.889083	-0.088148
C	2.360251	4.813219	-0.290635
C	3.064193	4.963025	1.078332
C	3.420379	4.791995	-1.416166
C	1.444216	6.030967	-0.504710
H	2.335108	4.975451	1.896668
H	3.770388	4.147449	1.269041
H	3.627111	5.903468	1.109451
H	2.949692	4.674686	-2.399098
H	3.983333	5.732928	-1.418670
H	4.140617	3.976133	-1.288525
H	2.041880	6.948897	-0.483891
H	0.933766	5.991838	-1.473976
H	0.685375	6.113943	0.281921
N	0.208000	1.016246	-0.316759
N	1.427787	-1.324763	0.061103
C	-4.269015	0.684315	0.902722
C	-2.543098	-0.209065	-0.402048
C	-4.749098	1.055630	2.186200
C	-5.117779	0.881658	-0.233126
C	-3.335070	-0.030036	-1.588356

C	-6.010238	1.594929	2.334108
H	-4.094688	0.896604	3.038999
C	-6.408680	1.438515	-0.049810
C	-4.595621	0.505152	-1.501551
H	-2.921816	-0.326043	-2.547562
C	-6.849356	1.789406	1.209559
H	-6.367884	1.872916	3.322447
H	-7.046171	1.583320	-0.919848
H	-5.208503	0.644960	-2.390814
H	-7.840609	2.214346	1.343466
N	-3.012452	0.156772	0.784901
O	-1.077177	-2.798611	0.095505
C	-2.048059	-3.685221	-0.411954
C	-2.087791	-3.647468	-1.951192
C	-1.606038	-5.088969	0.049328
C	-3.447928	-3.385015	0.157587
H	-2.415598	-2.662903	-2.298107
H	-1.085539	-3.834217	-2.354930
H	-2.773855	-4.402627	-2.357370
H	-1.540150	-5.120037	1.143224
H	-2.309017	-5.865968	-0.277133
H	-0.615964	-5.325775	-0.358896
H	-4.172769	-4.157632	-0.132524
H	-3.404519	-3.342160	1.251863
H	-3.818270	-2.422027	-0.201278
Rh	-0.685409	-0.881377	-0.261078
H	-0.572413	-0.972149	-1.785548

TS2C-dtbpy

G_{sol}(toluene) = -1554.183944 Hartree

C	-1.910608	1.511307	-0.766262
C	-1.964307	3.237140	-2.261194
C	-1.110569	4.018416	-1.478298
C	-0.648425	3.522399	-0.253152
C	-1.087022	2.238272	0.098375
C	-2.372435	0.142615	-0.405996
C	-1.988646	-1.915094	0.593048
C	-3.312089	-2.301076	0.416613
C	-4.214047	-1.448166	-0.230329
C	-3.704354	-0.205974	-0.628052
H	-2.338048	3.623554	-3.208082
H	-0.826384	5.000816	-1.837660
H	-0.783037	1.785179	1.033989
H	-1.272362	-2.552586	1.094939
H	-3.610403	-3.275075	0.784264
H	-4.335099	0.530261	-1.111456
C	0.314180	4.279151	0.670335
C	-0.302133	4.394727	2.083192
C	1.637377	3.481055	0.753294
C	0.621705	5.695269	0.153727
H	-1.251212	4.943388	2.057166
H	-0.490680	3.413248	2.530576
H	0.383994	4.933535	2.747627
H	2.103531	3.389397	-0.235060
H	2.344695	3.991087	1.419063
H	1.478453	2.468806	1.138051
H	1.304511	6.200396	0.846204

H	1.107409	5.674886	-0.828802
H	-0.285143	6.307115	0.076596
C	-5.683761	-1.800805	-0.487117
C	-6.583824	-0.772569	0.236903
C	-5.956908	-1.742607	-2.007841
C	-6.040201	-3.208268	0.023200
H	-6.402435	-0.782775	1.317979
H	-6.411732	0.246913	-0.124264
H	-7.640283	-1.012681	0.066714
H	-5.327704	-2.459349	-2.548353
H	-7.005958	-1.989399	-2.211000
H	-5.761812	-0.746612	-2.418992
H	-7.094997	-3.418881	-0.185734
H	-5.444473	-3.984461	-0.471145
H	-5.894042	-3.298962	1.105832
N	-1.503486	-0.731863	0.166536
N	-2.367506	2.006938	-1.930688
C	3.869193	-0.952983	-0.917655
C	1.585146	-0.719972	-1.398312
C	4.937969	-0.897525	0.011123
C	4.118056	-1.368949	-2.269796
C	1.740024	-1.098178	-2.756699
C	6.211374	-1.241725	-0.392206
H	4.726270	-0.584272	1.028681
C	5.442405	-1.706619	-2.644278
C	3.013148	-1.426357	-3.167725
H	0.883250	-1.146139	-3.420168
C	6.470053	-1.645776	-1.725359
H	7.030219	-1.201899	0.321224
H	5.633783	-2.018490	-3.668371
H	3.191271	-1.744499	-4.192630
H	7.481725	-1.909411	-2.020002
N	2.604896	-0.617171	-0.561568
O	0.315025	-1.124578	2.153560
C	1.382040	-1.194390	3.076584
C	2.346108	-2.338593	2.702320
C	2.154643	0.138689	3.153826
C	0.742930	-1.489388	4.446035
H	1.799669	-3.288776	2.665888
H	2.783055	-2.164408	1.713417
H	3.164938	-2.439241	3.427192
H	1.469584	0.954661	3.414257
H	2.949209	0.102576	3.911147
H	2.613428	0.377552	2.187921
H	1.497191	-1.573196	5.239213
H	0.043970	-0.688360	4.714067
H	0.179715	-2.428826	4.402243
Rh	0.566360	-0.539766	0.258973
H	0.620068	0.341552	-1.134599

TS3B-dtbpy

G_{sol}(toluene) = -1554.186044 Hartree

C	2.273141	-0.235344	0.071343
C	2.021282	-2.530854	0.287050
C	3.398179	-2.723038	0.361601
C	4.262877	-1.624258	0.280247
C	3.660898	-0.366473	0.130700

C	1.571073	1.059095	-0.078702	H	3.760300	4.154075	1.349323
C	-0.456284	2.157608	-0.356240	H	3.594466	5.910773	1.218980
C	0.163261	3.401459	-0.353851	H	2.870588	4.729070	-2.295436
C	1.550842	3.497631	-0.202591	H	3.908135	5.784563	-1.315882
C	2.235964	2.283822	-0.064360	H	4.088715	4.028060	-1.216585
H	1.316094	-3.355029	0.326140	H	1.965733	6.960030	-0.333422
H	3.771739	-3.733033	0.476742	H	0.850305	6.000668	-1.313669
H	4.283819	0.515619	0.051158	H	0.636317	6.096503	0.448265
H	-1.529491	2.076975	-0.467613	N	0.210720	0.994814	-0.225710
H	-0.460996	4.279262	-0.466110	N	1.465283	-1.321926	0.143234
H	3.310270	2.292497	0.068709	C	-4.403230	0.620540	0.780968
C	5.791898	-1.742272	0.339043	C	-2.502073	-0.212903	-0.314126
C	6.392950	-1.186421	-0.972982	C	-5.076276	0.878812	2.004787
C	6.319735	-0.919290	1.537131	C	-5.052561	0.973172	-0.444687
C	6.254297	-3.200357	0.507769	C	-3.101110	0.121709	-1.584249
H	6.029746	-1.748107	-1.841390	C	-6.328816	1.457478	2.010794
H	6.141171	-0.131221	-1.126021	H	-4.572323	0.604232	2.927552
H	7.486268	-1.266175	-0.948936	C	-6.339486	1.566603	-0.408387
H	5.905645	-1.289409	2.482168	C	-4.343163	0.700677	-1.646919
H	7.412370	-0.993553	1.592155	H	-2.546830	-0.094128	-2.493623
H	6.063752	0.142396	1.451105	C	-6.968847	1.805655	0.796146
H	7.348685	-3.237347	0.546219	H	-6.833127	1.648406	2.954961
H	5.876838	-3.643100	1.436730	H	-6.822834	1.829426	-1.347542
H	5.932661	-3.829331	-0.330328	H	-4.799362	0.954762	-2.602961
C	2.315648	4.826130	-0.175283	H	-7.956080	2.259751	0.817210
C	3.041783	4.964340	1.183251	N	-3.160361	0.047545	0.815983
C	3.356585	4.836842	-1.318852	O	-0.922512	-2.959350	-0.268047
C	1.380001	6.034398	-0.354547	C	-2.111997	-3.760240	-0.340090
H	2.327002	4.955837	2.014151	C	-2.930398	-3.449050	-1.599497

C	-1.601848	-5.209507	-0.398097
C	-2.950319	-3.542631	0.925775
H	-3.330135	-2.433067	-1.567485
H	-2.304089	-3.548513	-2.494891
H	-3.774159	-4.143530	-1.694160
H	-0.988581	-5.430166	0.483120
H	-2.434330	-5.923378	-0.425917
H	-0.984946	-5.360934	-1.291622
H	-3.842790	-4.181981	0.925517
H	-2.354947	-3.782808	1.814594
H	-3.265626	-2.499076	1.004130
Rh	-0.633439	-0.885692	-0.092596
H	-0.748647	-2.011095	-1.257555

INT3B-dtbpy

G_{sol}(toluene) = -1320.754289 Hartree

C	-1.688616	-0.642472	0.000047
C	-0.669013	-2.729453	-0.000073
C	-1.896591	-3.377109	-0.000040
C	-3.089517	-2.639259	0.000042
C	-2.945319	-1.247125	0.000084
C	-1.473439	0.813240	0.000072
C	0.120498	2.495934	-0.000008
C	-0.854782	3.486011	0.000050
C	-2.212472	3.134982	0.000139
C	-2.495547	1.763360	0.000146
H	0.259432	-3.286794	-0.000134
H	-1.899033	-4.460261	-0.000078
H	-3.827502	-0.618028	0.000145
H	1.175582	2.747883	-0.000069
H	-0.534952	4.520739	0.000032
H	-3.526850	1.430909	0.000207
C	-4.485281	-3.275802	0.000078
C	-5.253180	-2.819548	1.262399
C	-5.253308	-2.819391	-1.262108
C	-4.420422	-4.813672	-0.000020
H	-4.729497	-3.128503	2.174527
H	-5.374111	-1.731320	1.297420
H	-6.255054	-3.265618	1.277226
H	-4.729723	-3.128242	-2.174328
H	-6.255188	-3.265448	-1.276885
H	-5.374229	-1.731156	-1.296986
H	-5.435844	-5.225599	0.000049
H	-3.907858	-5.199669	-0.888799
H	-3.907693	-5.199773	0.888619
C	-3.353432	4.160768	0.000248
C	-4.222327	3.953911	-1.261980
C	-4.221825	3.954151	1.262861
C	-2.830679	5.608276	0.000003
H	-3.629571	4.089719	-2.174054
H	-4.664495	2.952107	-1.295574
H	-5.043909	4.680284	-1.278811
H	-3.628703	4.090131	2.174671
H	-5.043403	4.680525	1.279887
H	-4.663971	2.952349	1.296817
H	-3.675948	6.305489	0.000126
H	-2.226005	5.823101	0.888685

H	-2.226393	5.822938	-0.888984
N	-0.160793	1.181647	0.000004
N	-0.537409	-1.385469	-0.000031
C	4.606369	0.186200	-0.000156
C	2.889217	-1.455467	-0.000169
C	4.990666	1.549676	-0.000117
C	5.610153	-0.838829	-0.000208
C	3.837332	-2.515220	-0.000224
C	6.328203	1.885918	-0.000131
H	4.212313	2.307363	-0.000076
C	6.974099	-0.452113	-0.000222
C	5.178824	-2.196473	-0.000242
H	3.508846	-3.550730	-0.000249
C	7.328338	0.881649	-0.000183
H	6.622436	2.932322	-0.000100
H	7.738200	-1.226303	-0.000261
H	5.934380	-2.980240	-0.000283
H	8.376483	1.167671	-0.000193
N	3.302292	-0.190759	-0.000141
Rh	1.233688	-0.401259	-0.000069

^{t}BuOH

G_{sol}(toluene) = -233.477446 Hartree

C	0.686001	1.264136	-0.514100
C	-0.005595	-0.000005	0.014465
H	0.208155	2.159322	-0.101271
H	1.745025	1.278011	-0.223356
H	0.640580	1.318091	-1.608258
C	0.687466	-1.263282	-0.514240
C	-1.490634	-0.000830	-0.349636
H	0.210709	-2.159068	-0.101454
H	0.642048	-1.317207	-1.608399
H	1.746528	-1.275920	-0.223565
H	-1.626113	-0.000797	-1.436560
H	-1.983273	-0.888190	0.062880
H	-1.984289	0.885883	0.063059
O	0.022567	-0.000070	1.454499
H	0.956672	0.000321	1.721997

TS3C-dtbpy

G_{sol}(toluene) = -4848.993500 Hartree

C	2.518127	-1.128158	-0.215780
C	0.901113	-2.712673	0.263873
C	1.840857	-3.738922	0.276408
C	3.185062	-3.457984	0.008443
C	3.506323	-2.111062	-0.215856
C	2.794839	0.320437	-0.288083
C	1.947749	2.432465	0.117953
C	3.178763	3.026563	-0.148286
C	4.269814	2.239597	-0.531891
C	4.044337	0.856574	-0.589800
H	-0.134031	-2.888983	0.505272
H	1.500389	-4.741292	0.503606
H	4.540394	-1.821639	-0.357194
H	1.085244	3.021074	0.405178
H	3.255256	4.102928	-0.057127

H	4.850102	0.190649	-0.873968	C	-1.344175	4.157109	-0.046756
C	4.284887	-4.526089	-0.009490	C	-1.317630	1.873233	-0.476257
C	4.983055	-4.510231	-1.389160	C	-1.109802	5.213030	0.871715
C	5.317758	-4.204809	1.095609	C	-1.785664	4.471523	-1.370941
C	3.724430	-5.937681	0.237855	C	-1.789591	2.096310	-1.820426
H	4.271161	-4.726134	-2.194274	C	-1.306815	6.525092	0.490426
H	5.449573	-3.542405	-1.603017	H	-0.782374	4.951810	1.874121
H	5.771227	-5.271812	-1.418934	C	-1.983744	5.825074	-1.731071
H	4.847351	-4.203927	2.085535	C	-2.006741	3.374408	-2.254110
H	6.113233	-4.959381	1.096307	H	-1.967515	1.241879	-2.462173
H	5.787305	-3.226569	0.945577	C	-1.746999	6.835259	-0.818255
H	4.540243	-6.668073	0.204315	H	-1.127615	7.328766	1.199860
H	3.248263	-6.021442	1.221383	H	-2.323420	6.057825	-2.737932
H	2.992437	-6.224316	-0.526152	H	-2.349607	3.566139	-3.268574
C	5.653376	2.810112	-0.866297	H	-1.899527	7.872823	-1.102873
C	6.690378	2.220305	0.117612	N	-1.131768	2.872952	0.364731
C	6.030609	2.413371	-2.312480	O	-0.460603	-0.502435	2.022706
C	5.689332	4.344157	-0.754818	C	0.162827	-0.052957	3.185915
H	6.449885	2.491109	1.152200	C	0.016007	1.472334	3.364741
H	6.735235	1.127335	0.057939	C	-0.549554	-0.763813	4.358470
H	7.689906	2.607733	-0.112542	C	1.664163	-0.427529	3.217858
H	5.303824	2.808026	-3.031854	H	0.512084	2.002453	2.546691
H	7.016539	2.820058	-2.566588	H	-1.042572	1.750154	3.332829
H	6.076898	1.326668	-2.442451	H	0.446629	1.819222	4.315222
H	6.693243	4.706905	-1.001801	H	-0.444105	-1.850747	4.254831
H	4.986288	4.819210	-1.448655	H	-0.145056	-0.467692	5.336587
H	5.456820	4.684938	0.260591	H	-1.619016	-0.525651	4.334201
N	1.743175	1.113259	0.041527	H	2.135117	-0.172855	4.177820
N	1.218047	-1.448683	-0.021412	H	1.783153	-1.505354	3.051188

H	2.207560	0.101210	2.428369
H	-0.024042	0.224068	-1.582046
Rh	-0.194432	0.117383	-0.014058
C	-2.097953	-0.761950	-0.336023
C	-2.317864	0.404449	0.172920
Br	-3.608067	1.174604	1.324084
Si	-2.969445	-2.315923	-0.877995
C	-4.439097	-1.836619	-2.025165
C	-5.033695	-0.441469	-1.764965
C	-5.558663	-2.898535	-2.017443
H	-4.004611	-1.826033	-3.037595
H	-4.284768	0.350449	-1.863118
H	-5.843893	-0.227305	-2.477005
H	-5.455067	-0.361359	-0.756453
H	-5.192356	-3.909275	-2.225596
H	-6.073704	-2.930445	-1.050342
H	-6.317002	-2.662948	-2.777718
C	-3.566034	-3.259866	0.678177
C	-4.599143	-2.472705	1.505695
C	-2.395291	-3.675756	1.588830
H	-4.055320	-4.172048	0.300100
H	-5.464679	-2.149729	0.916659
H	-4.977910	-3.088933	2.333874
H	-4.147673	-1.576957	1.942946
H	-1.704038	-4.364761	1.086257
H	-1.830486	-2.796228	1.927204
H	-2.765772	-4.195513	2.484382
C	-1.777781	-3.435135	-1.880981
C	-0.991269	-2.661055	-2.953652
C	-2.494418	-4.644374	-2.511519
H	-1.050220	-3.836564	-1.164700
H	-0.409289	-1.840627	-2.522766
H	-0.296522	-3.327412	-3.485978
H	-1.662209	-2.229091	-3.709085
H	-3.050561	-5.235952	-1.773822
H	-3.200420	-4.332187	-3.290896
H	-1.768607	-5.317665	-2.989743

INT3C-dtbpy

G_{sol}(toluene) = -4849.076153 Hartree

C	2.733844	-0.409673	-0.749741
C	2.260087	-2.667221	-0.497495
C	3.576912	-3.020766	-0.779127
C	4.526662	-2.027493	-1.050673
C	4.065176	-0.702665	-1.044107
C	2.159118	0.953971	-0.767033
C	0.234984	2.243697	-0.653876
C	0.955350	3.420531	-0.829372
C	2.346648	3.377663	-0.966345
C	2.929661	2.103934	-0.929197
H	1.476192	-3.389722	-0.299660
H	3.836154	-4.072227	-0.786181
H	4.747628	0.103223	-1.283334
H	-0.839558	2.261285	-0.531662
H	0.406853	4.354060	-0.849039
H	4.004492	2.005860	-1.017048
C	6.000659	-2.326417	-1.353936

C	6.356940	-1.779809	-2.756005	C	0.745869	1.113239	3.964646
C	6.881244	-1.631069	-0.289700	C	-1.427144	1.794470	3.157994
C	6.303204	-3.835175	-1.328912	C	2.952719	-0.594380	3.676177
H	5.737271	-2.247312	-3.529852	H	1.946739	-1.378691	1.946986
H	6.218942	-0.695135	-2.824139	C	1.819884	1.165875	4.888185
H	7.407400	-1.994532	-2.985616	C	-0.415323	1.919156	4.073528
H	6.645723	-1.997297	0.716482	H	-2.319383	2.399372	3.241486
H	7.940660	-1.834195	-0.486405	C	2.908379	0.331922	4.745938
H	6.744944	-0.544028	-0.292526	H	3.802602	-1.265294	3.582297
H	7.364558	-4.000075	-1.544745	H	1.763511	1.870231	5.714735
H	6.093305	-4.277788	-0.348368	H	-0.498175	2.629160	4.892925
H	5.724951	-4.379362	-2.084370	H	3.727099	0.373864	5.458754
C	3.223440	4.622737	-1.143366	N	-0.200580	0.119043	1.943382
C	4.226433	4.702598	0.031291	O	-0.735023	-2.725687	0.114888
C	3.998179	4.510437	-2.476963	C	-1.125905	-3.451343	1.259508
C	2.391863	5.917174	-1.167664	C	-1.628572	-2.598487	2.439258
H	3.703007	4.774087	0.991718	C	-2.258901	-4.399060	0.810568
H	4.882881	3.826391	0.069953	C	0.061854	-4.303676	1.766020
H	4.861735	5.589638	-0.077258	H	-0.803677	-2.076887	2.930427
H	3.310594	4.439560	-3.327633	H	-2.343560	-1.849932	2.094535
H	4.627478	5.396619	-2.621095	H	-2.121424	-3.229077	3.190880
H	4.652601	3.632140	-2.498694	H	-1.932148	-4.974899	-0.063258
H	3.056168	6.778163	-1.300037	H	-2.551675	-5.102703	1.602048
H	1.673242	5.924119	-1.995295	H	-3.141077	-3.819495	0.522124
H	1.841554	6.066185	-0.231509	H	-0.210448	-4.907784	2.642421
N	0.806083	1.030479	-0.617058	H	0.406619	-4.988256	0.980542
N	1.854193	-1.394808	-0.454220	H	0.899415	-3.655442	2.052014
C	0.804755	0.199403	2.866443	H	-0.291530	-1.147908	-1.635515
C	1.927234	-0.662531	2.756840	Rh	-0.174354	-0.766061	-0.152501

C	-2.091686	-0.019570	-0.118363
Br	-4.040493	1.586951	1.422317
Si	-3.365520	-0.324017	-1.529883
C	-4.157860	1.303773	-2.237105
C	-3.248965	2.536442	-2.060370
C	-5.607146	1.646971	-1.851812
H	-4.175080	1.080928	-3.316040
H	-2.228998	2.357233	-2.416947
H	-3.644642	3.394222	-2.624612
H	-3.192234	2.836483	-1.008687
H	-6.309307	0.871740	-2.170325
H	-5.726801	1.786246	-0.774288
H	-5.918177	2.583748	-2.338750
C	-4.609429	-1.686090	-0.959463
C	-5.896585	-1.812053	-1.803951
C	-5.002285	-1.689101	0.526872
H	-4.007865	-2.591158	-1.138075
H	-5.746486	-1.663578	-2.877139
H	-6.342591	-2.808446	-1.671651
H	-6.647078	-1.084945	-1.475239
H	-4.137356	-1.621524	1.189254
H	-5.660597	-0.846679	0.763563
H	-5.544743	-2.612481	0.778544
C	-2.433419	-1.067844	-3.042660
C	-1.519879	-0.046979	-3.750019
C	-3.336410	-1.745965	-4.089054
H	-1.795983	-1.849281	-2.609404
H	-0.843139	0.462668	-3.056203
H	-0.895691	-0.543354	-4.507888
H	-2.106690	0.719885	-4.272235
H	-3.881989	-2.600765	-3.676319
H	-4.071913	-1.052625	-4.520484
H	-2.731446	-2.124609	-4.926413
C	-1.299189	0.873956	2.075191
C	-2.299076	0.689187	1.009219

TS2D-dtbpy

G_{sol}(toluene) = -1554.189705 Hartree

C	2.043727	-0.331100	-0.138392
C	1.373504	-2.529967	-0.492969
C	2.677765	-2.987906	-0.363220
C	3.725630	-2.092535	-0.097781
C	3.368066	-0.743921	0.010209
C	1.588863	1.058750	-0.065791
C	-0.251881	2.475003	-0.127192
C	0.558556	3.599392	-0.078623
C	1.954281	3.470910	-0.008781
C	2.442626	2.160425	-0.017888
H	0.542679	-3.198310	-0.680972
H	2.854889	-4.050988	-0.470756
H	4.127646	-0.000929	0.223098
H	-1.326848	2.573404	-0.192760
H	0.076715	4.569307	-0.100636
H	3.511975	1.985740	-0.005967
C	5.187229	-2.524319	0.081643
C	6.069899	-1.793779	-0.956618
C	5.652789	-2.148564	1.507561

C	5.370582	-4.040774	-0.106734	C	-4.968928	1.131440	-0.478467
H	5.760083	-2.039801	-1.978972	C	-4.425362	-0.740629	-1.918152
H	6.020753	-0.705240	-0.843892	H	-2.298976	-2.453698	-0.882312
H	7.118585	-2.092182	-0.837706	C	-4.196880	2.750475	1.692496
H	5.042349	-2.652580	2.265878	H	-2.335654	1.658462	1.659570
H	6.697280	-2.448393	1.656500	C	-5.854990	2.108232	0.045779
H	5.587251	-1.069880	1.687601	C	-5.315283	0.235512	-1.526522
H	6.426048	-4.303813	0.026341	H	-4.710032	-1.457196	-2.685092
H	4.793324	-4.615886	0.626444	C	-5.475743	2.907596	1.103772
H	5.073360	-4.365509	-1.110644	H	-3.921194	3.362190	2.547583
C	2.919846	4.661239	0.051192	H	-6.845139	2.205074	-0.393791
C	3.773732	4.560383	1.336536	H	-6.299654	0.320810	-1.984772
C	3.848376	4.627457	-1.185039	H	-6.164085	3.647779	1.502459
C	2.176399	6.008657	0.065537	N	-2.796678	0.078262	-0.414624
H	3.141420	4.578341	2.231845	O	-1.668778	-3.051190	-0.376536
H	4.366974	3.639785	1.363050	C	-2.316043	-3.706884	0.727909
H	4.470362	5.405505	1.394526	C	-1.193053	-4.393358	1.509195
H	3.269470	4.695334	-2.113361	C	-3.040247	-2.689000	1.620986
H	4.547148	5.472428	-1.156410	C	-3.309083	-4.739331	0.175270
H	4.441556	3.707359	-1.226117	H	-0.663152	-5.109399	0.870210
H	2.900897	6.829126	0.117689	H	-0.471338	-3.649201	1.864362
H	1.579115	6.154685	-0.841868	H	-1.591493	-4.934247	2.375397
H	1.512620	6.098519	0.933399	H	-3.851863	-2.195357	1.076181
N	0.222967	1.209528	-0.096382	H	-3.471625	-3.182489	2.500615
N	1.039579	-1.227826	-0.376033	H	-2.341110	-1.915009	1.956460
Rh	-0.856457	-0.471908	-0.359581	H	-3.797105	-5.297649	0.983872
C	-3.665322	1.011788	0.092597	H	-4.088954	-4.240463	-0.412900
C	-3.122473	-0.830961	-1.335992	H	-2.793564	-5.452122	-0.478137
C	-3.306745	1.817792	1.200258				

TS4A-dtbpy

G_{sol}(toluene) = -4849.075504 Hartree

C	2.721197	1.013333	-0.292583
C	0.937410	2.455783	-0.664416
C	1.782768	3.559455	-0.728528
C	3.159839	3.396350	-0.547436
C	3.610540	2.084580	-0.336704
C	3.142466	-0.398955	-0.131645
C	2.444358	-2.610393	-0.062207
C	3.752752	-3.072883	0.041604
C	4.812562	-2.159191	0.074003
C	4.471949	-0.801878	-0.017759
H	-0.135703	2.549917	-0.785528
H	1.340870	4.530515	-0.912882
H	4.669372	1.899173	-0.209777
H	1.603087	-3.294179	-0.081173
H	3.918338	-4.141381	0.095437
H	5.255610	-0.055166	-0.007555
C	4.162049	4.557168	-0.575883
C	4.911442	4.608250	0.775719
C	5.175501	4.324365	-1.720521
C	3.471065	5.913448	-0.801969
H	4.213806	4.759166	1.607620
H	5.472588	3.687652	0.969771
H	5.626977	5.438996	0.775770
H	4.668963	4.276314	-2.691265
H	5.898404	5.148026	-1.753230
H	5.738367	3.393958	-1.587911
H	4.222127	6.711041	-0.808208
H	2.945284	5.949130	-1.762901
H	2.753204	6.142754	-0.006021
C	6.284254	-2.574319	0.196481
C	7.062464	-2.052260	-1.033650
C	6.876150	-1.954922	1.484132
C	6.451506	-4.102316	0.267225
H	6.657428	-2.469877	-1.962589
H	7.024977	-0.960351	-1.111256
H	8.116957	-2.343722	-0.960545
H	6.335054	-2.300837	2.372353
H	7.927781	-2.246128	1.590593
H	6.834830	-0.860274	1.470380
H	7.515060	-4.351505	0.350840
H	5.943731	-4.528626	1.139999
H	6.067537	-4.596532	-0.632605
N	2.141669	-1.312397	-0.138171
N	1.388071	1.219288	-0.431271
C	-1.834391	-2.944492	-0.198597
C	-0.437684	-2.203795	1.591670
C	-2.326006	-2.752812	-1.508374
C	-2.411429	-3.953087	0.633879
C	-0.995009	-3.199351	2.447832
H	0.443683	-0.742698	2.256190
C	-3.348539	-3.550809	-1.980995
H	-1.896395	-1.969526	-2.117538
C	-3.451201	-4.762973	0.109303
C	-1.946969	-4.069075	1.971940
H	-0.663381	-3.255398	3.481306

C	-3.913605	-4.566114	-1.174104
H	-3.730217	-3.387990	-2.985170
H	-3.886498	-5.529039	0.746404
H	-2.378886	-4.837310	2.611484
H	-4.718131	-5.182259	-1.565933
N	-0.841547	-2.150308	0.337745
O	0.818233	0.193537	2.282240
C	0.123266	1.009016	3.244633
C	-1.340417	0.565573	3.379482
C	0.856062	0.880384	4.587679
C	0.193567	2.445375	2.722040
H	-1.836521	0.595873	2.405880
H	-1.409552	-0.456356	3.770413
H	-1.879187	1.227058	4.068533
H	1.903492	1.185109	4.480540
H	0.388919	1.504609	5.359876
H	0.838240	-0.160736	4.932123
H	-0.254181	3.145294	3.437276
H	1.235493	2.741791	2.553750
H	-0.346495	2.521882	1.774573
Rh	0.171587	-0.475257	-0.247224
Br	0.270578	-0.662938	-2.727291
C	-1.552103	0.445898	-0.286141
C	-2.663711	0.973329	-0.237109
Si	-4.419801	1.431353	-0.092809
C	-5.204809	1.331497	-1.835601
H	-6.286732	1.493428	-1.711181
C	-4.541712	3.227253	0.562213
H	-4.020647	3.838133	-0.191316
C	-5.288319	0.226829	1.130739
H	-5.172174	0.694582	2.121267
C	-4.628641	-1.163176	1.202346
H	-3.569865	-1.096255	1.469357
H	-5.128760	-1.794911	1.951636
H	-4.684348	-1.690342	0.242598
C	-6.798774	0.094048	0.853746
H	-7.287283	-0.514589	1.628617
H	-7.311812	1.062880	0.828079
H	-6.985546	-0.400001	-0.107928
C	-4.993891	-0.055979	-2.469759
H	-5.430315	-0.861088	-1.867436
H	-5.449861	-0.106694	-3.469609
H	-3.924597	-0.273609	-2.578515
C	-4.659393	2.430534	-2.764791
H	-3.571050	2.342410	-2.881062
H	-5.102083	2.355206	-3.768822
H	-4.874623	3.437734	-2.387707
C	-5.990443	3.736219	0.662949
H	-6.552716	3.189683	1.431162
H	-6.019531	4.799697	0.941613
H	-6.535649	3.630880	-0.283471
C	-3.801976	3.414017	1.898691
H	-2.753825	3.102205	1.829472
H	-3.819003	4.466743	2.217784
H	-4.267118	2.828050	2.702775

TS4B-dtbpy

G_{sol}(toluene) = -4849.062430 Hartree

C	2.485906	1.237694	-0.258920
C	0.602479	2.578254	-0.527380
C	1.354385	3.739485	-0.381755
C	2.729953	3.658660	-0.144650
C	3.280714	2.369328	-0.097470
C	3.013200	-0.145304	-0.272951
C	2.495671	-2.384994	-0.584426
C	3.826028	-2.763040	-0.434889
C	4.801527	-1.793175	-0.175653
C	4.358923	-0.464522	-0.103167
H	-0.467732	2.605887	-0.693806
H	0.840082	4.689603	-0.448041
H	4.344396	2.248953	0.064851
H	1.719199	-3.117576	-0.769966
H	4.073563	-3.813710	-0.516532
H	5.072172	0.327578	0.084164
C	3.626581	4.885068	0.061038
C	4.276626	4.797057	1.461473
C	4.729681	4.897748	-1.022298
C	2.835866	6.202037	-0.030198
H	3.513877	4.776864	2.248501
H	4.895867	3.899970	1.570259
H	4.919830	5.668392	1.632119
H	4.294871	4.949980	-2.027107
H	5.377577	5.771612	-0.885918
H	5.362861	4.005018	-0.975368
H	3.513763	7.048395	0.125510
H	2.369092	6.329964	-1.013630

H	2.053243	6.261304	0.734826
C	6.287647	-2.120173	0.017381
C	7.108383	-1.397634	-1.076361
C	6.737344	-1.628453	1.413104
C	6.565828	-3.630756	-0.080613
H	6.804976	-1.725542	-2.077358
H	6.989423	-0.309707	-1.028246
H	8.174726	-1.620918	-0.953510
H	6.159672	-2.115124	2.207227
H	7.796717	-1.864019	1.568379
H	6.619382	-0.545342	1.527312
H	7.635394	-3.817036	0.065715
H	6.024595	-4.195235	0.687381
H	6.293768	-4.033655	-1.063061
N	2.093600	-1.114871	-0.496973
N	1.147650	1.360703	-0.446667
C	-0.793021	-2.981046	0.466621
C	-1.574102	-2.688780	-1.732565
C	-0.104845	-2.383195	1.549918
C	-1.388095	-4.284329	0.522928
C	-2.209830	-3.947092	-1.722515
H	-1.623284	-2.040967	-2.598682
C	0.006299	-3.175481	2.685166
H	0.308628	-0.821402	1.891828
C	-1.235086	-5.032200	1.719029
C	-2.107808	-4.746568	-0.605302
H	-2.765534	-4.260986	-2.599966
C	-0.538946	-4.486095	2.775600
H	0.519211	-2.786420	3.566273

H	-1.675516	-6.024364	1.783440
H	-2.581766	-5.725386	-0.574952
H	-0.416979	-5.057984	3.693839
N	-0.882971	-2.239091	-0.694160
O	0.559193	0.185999	2.012235
C	-0.160496	0.812760	3.080344
C	-0.138729	2.319234	2.806303
C	-1.611395	0.309617	3.139382
C	0.571953	0.500144	4.395430
H	0.892982	2.676770	2.706972
H	-0.669427	2.538798	1.875797
H	-0.621723	2.875536	3.618715
H	-1.647566	-0.761929	3.361383
H	-2.174231	0.839584	3.917983
H	-2.101228	0.472615	2.177035
H	0.081899	0.980344	5.252158
H	0.587359	-0.581362	4.574277
H	1.609233	0.851528	4.345407
Rh	0.082880	-0.399022	-0.584491
Br	0.256043	-0.116421	-3.039224
C	-1.714569	0.375887	-0.536763
C	-2.877812	0.768457	-0.448270
Si	-4.656876	0.988394	-0.117397
C	-5.618215	0.708833	-1.747556
H	-6.688648	0.739160	-1.492710
C	-4.944117	2.779927	0.495271
H	-4.552934	3.411664	-0.317327
C	-5.190337	-0.279549	1.227271
H	-4.972094	0.215635	2.186437

C	-4.387262	-1.594109	1.202409
H	-3.310852	-1.420489	1.282459
H	-4.686440	-2.248435	2.034612
H	-4.556356	-2.153378	0.273693
C	-6.702969	-0.574971	1.187977
H	-6.998189	-1.219180	2.028809
H	-7.314768	0.333237	1.242554
H	-6.981918	-1.101946	0.266589
C	-5.311949	-0.670047	-2.360305
H	-5.579963	-1.492899	-1.687442
H	-5.866979	-0.817575	-3.298520
H	-4.243016	-0.766598	-2.588417
C	-5.335032	1.822955	-2.771306
H	-4.265632	1.869724	-3.015695
H	-5.877711	1.643290	-3.710914
H	-5.636035	2.811907	-2.404791
C	-6.427640	3.136757	0.695709
H	-6.864456	2.576038	1.531942
H	-6.549299	4.204376	0.930062
H	-7.032975	2.926887	-0.195085
C	-4.129238	3.101922	1.760339
H	-3.062551	2.893785	1.620481
H	-4.230945	4.161180	2.038792
H	-4.468940	2.509755	2.620234

TS4C-dtbpy

G_{sol}(toluene) = -4849.049768 Hartree

C	2.732550	1.082720	-0.184942

C	0.903282	2.442940	-0.656457	C	7.218875	-1.678650	-1.141993
C	1.701365	3.581716	-0.678920	C	7.058456	-1.749354	1.378257
C	3.069942	3.486585	-0.407311	C	6.736353	-3.837493	0.033225
C	3.567745	2.197474	-0.168259	H	6.823813	-2.056000	-2.092067
C	3.220549	-0.309498	-0.061593	H	7.123255	-0.587423	-1.147173
C	2.650461	-2.545903	-0.182086	H	8.288017	-1.917374	-1.098293
C	3.984894	-2.941248	-0.139556	H	6.554747	-2.186476	2.248182
C	4.992793	-1.979221	-0.022371	H	8.127664	-1.979719	1.452335
C	4.573367	-0.641203	-0.005992	H	6.948705	-0.660977	1.437517
H	-0.165255	2.489008	-0.826809	H	7.811659	-4.033442	0.106065
H	1.225752	4.529380	-0.897902	H	6.250869	-4.345261	0.874699
H	4.623504	2.062273	0.028840	H	6.381650	-4.293995	-0.897985
H	1.858090	-3.274573	-0.291669	N	2.264507	-1.269915	-0.103107
H	4.208842	-3.998704	-0.201668	N	1.396905	1.226161	-0.389542
H	5.313544	0.147805	0.029362	C	-2.773056	-3.507813	-0.080661
C	4.013549	4.694441	-0.366832	C	-0.904398	-2.327009	0.621767
C	4.629609	4.797879	1.048077	C	-4.073992	-3.479472	-0.656621
C	5.139046	4.496777	-1.409006	C	-2.189396	-4.770952	0.269009
C	3.282609	6.011632	-0.680389	C	-0.268244	-3.539170	1.038843
H	3.851268	4.936902	1.807261	H	-0.453649	-1.383713	1.458490
H	5.201985	3.902165	1.312941	C	-4.758824	-4.652378	-0.877750
H	5.311209	5.655161	1.097896	H	-4.489480	-2.511041	-0.916012
H	4.727750	4.408513	-2.421040	C	-2.923268	-5.961789	0.029158
H	5.819448	5.356233	-1.391445	C	-0.904450	-4.745832	0.866760
H	5.734644	3.599619	-1.208118	H	0.693930	-3.485850	1.537514
H	3.993892	6.843799	-0.639755	C	-4.179936	-5.901943	-0.532646
H	2.840901	6.004311	-1.683410	H	-5.750366	-4.628389	-1.321637
H	2.488773	6.220723	0.045836	H	-2.478567	-6.918084	0.295057
C	6.485878	-2.319366	0.059382	H	-0.444911	-5.677745	1.190343

H	-4.736471	-6.817217	-0.716070
N	-2.132761	-2.326552	0.119110
O	0.395788	-0.547608	2.052131
C	-0.140077	0.328630	3.062995
C	-1.633116	0.051637	3.283050
C	0.659671	-0.019374	4.328332
C	0.091922	1.793774	2.677741
H	-2.195802	0.248787	2.368869
H	-1.791230	-0.994808	3.571062
H	-2.032462	0.688412	4.082100
H	1.727764	0.166594	4.166638
H	0.326097	0.583003	5.182358
H	0.530569	-1.078085	4.579540
H	-0.213905	2.461102	3.493000
H	1.153675	1.970160	2.469775
H	-0.487358	2.047065	1.787391
Rh	0.246809	-0.461390	-0.064971
Br	0.110925	-0.910198	-2.520626
C	-1.446874	0.519380	-0.125481
C	-2.474483	1.193886	-0.162705
Si	-4.110242	1.992428	-0.132256
C	-4.934476	1.736778	-1.840247
H	-5.921489	2.224579	-1.809709
C	-3.832172	3.870718	0.156262
H	-3.022085	4.123888	-0.545993
C	-5.082180	1.223490	1.331567
H	-4.519418	1.564986	2.214844
C	-5.054179	-0.316821	1.351952
H	-4.052602	-0.717082	1.165851
H	-5.400315	-0.698436	2.323918
H	-5.721071	-0.737615	0.590028
C	-6.525426	1.742055	1.464622
H	-7.029809	1.286451	2.329504
H	-6.569647	2.828905	1.599835
H	-7.124578	1.496220	0.577707
C	-5.139060	0.248294	-2.178083
H	-5.856776	-0.231705	-1.503547
H	-5.521141	0.126296	-3.202410
H	-4.192428	-0.301765	-2.109629
C	-4.096534	2.413852	-2.942813
H	-3.095075	1.968319	-2.997190
H	-4.566903	2.286156	-3.928567
H	-3.973161	3.491152	-2.776676
C	-5.034948	4.768325	-0.183754
H	-5.877670	4.594047	0.496402
H	-4.769262	5.832118	-0.095444
H	-5.400084	4.606777	-1.204837
C	-3.317289	4.163226	1.577129
H	-2.444696	3.549062	1.831816
H	-3.025034	5.218171	1.686112
H	-4.090770	3.965482	2.330935

TS4D-dtbpy

G_{sol}(toluene) = -4849.046697 Hartree

C	-1.343252	-3.465199	0.136673
C	-3.348144	-3.439614	-1.001731
C	-0.260707	-2.754311	0.763354

C	-1.300028	-4.901586	0.075467	Br	0.122852	-1.091325	-2.427170
C	-3.378808	-4.848729	-1.145729	C	-1.610615	0.016553	0.041554
H	-4.159836	-2.837521	-1.406124	C	-2.685875	0.616186	0.020785
C	0.748251	-3.512424	1.358815	Si	-4.332129	1.367694	-0.135518
H	-0.225866	-1.709415	1.595803	C	-4.843518	1.290491	-1.979033
C	-0.228886	-5.607129	0.682214	H	-5.861271	1.704320	-2.052877
C	-2.352384	-5.576930	-0.595655	C	-4.192742	3.209883	0.391085
H	-4.201094	-5.326075	-1.670309	H	-3.356178	3.595194	-0.213811
C	0.771866	-4.924252	1.335049	C	-5.586965	0.450488	0.992267
H	1.535089	-2.985532	1.891323	H	-5.439641	0.891970	1.990499
H	-0.222117	-6.693987	0.632496	C	-5.314891	-1.059600	1.119474
H	-2.330034	-6.662538	-0.661594	H	-4.274729	-1.271517	1.385802
H	1.578579	-5.461410	1.827139	H	-5.965586	-1.510537	1.883612
N	-2.385301	-2.775989	-0.397624	H	-5.516272	-1.582199	0.177000
O	0.438433	-0.682090	2.140277	C	-7.050798	0.699024	0.580877
C	-0.212928	0.074512	3.176110	H	-7.745423	0.231869	1.294225
C	-0.233037	1.562656	2.814070	H	-7.301691	1.764980	0.531864
C	-1.633840	-0.450999	3.421150	H	-7.265124	0.267598	-0.405557
C	0.658758	-0.155862	4.421368	C	-4.867382	-0.158217	-2.498640
H	0.782686	1.920925	2.608834	H	-5.601320	-0.774469	-1.965603
H	-0.847561	1.722377	1.926835	H	-5.125826	-0.192350	-3.567423
H	-0.647089	2.157152	3.637814	H	-3.883533	-0.627369	-2.377786
H	-1.605713	-1.514831	3.687280	C	-3.909266	2.147673	-2.852982
H	-2.113375	0.093215	4.244194	H	-2.875922	1.783213	-2.790128
H	-2.244707	-0.332352	2.524081	H	-4.206610	2.102862	-3.910884
H	0.253172	0.377911	5.289880	H	-3.911636	3.204556	-2.558233
H	0.703196	-1.224243	4.661315	C	-5.433170	4.059740	0.064455
H	1.680658	0.197526	4.241168	H	-6.294139	3.762822	0.676664
Rh	0.235974	-0.643077	0.031843	H	-5.249321	5.124537	0.270655

H	-5.732803	3.977049	-0.987365
C	-3.801258	3.369119	1.870440
H	-2.910815	2.782679	2.123784
H	-3.589780	4.420486	2.116167
H	-4.611364	3.042550	2.535646
C	2.333892	1.401270	-0.134833
C	0.252932	2.361941	-0.525478
C	0.796828	3.636359	-0.537434
C	2.165255	3.826085	-0.295745
C	2.924628	2.668563	-0.105120
C	3.111824	0.142785	-0.080329
C	3.026162	-2.157783	-0.278107
C	4.414189	-2.256265	-0.329990
C	5.199145	-1.104549	-0.219500
C	4.505478	0.109559	-0.114545
H	-0.806254	2.187900	-0.664165
H	0.133997	4.475020	-0.719221
H	3.989180	2.743427	0.064471
H	2.401731	-3.035922	-0.375872
H	4.854180	-3.237012	-0.459796
H	5.060962	1.037990	-0.086366
C	2.761591	5.237419	-0.258201
C	2.060702	6.043452	0.860450
C	4.275122	5.227857	0.020064
C	2.515953	5.924029	-1.622060
H	0.981089	6.120122	0.694114
H	2.218249	5.576894	1.839934
H	2.465809	7.061544	0.898661
H	4.831123	4.693236	-0.759233

H	4.649830	6.256899	0.042356
H	4.509984	4.772825	0.989619
H	2.933310	6.937725	-1.610279
H	2.995240	5.367399	-2.435706
H	1.448874	6.006595	-1.853497
C	6.732660	-1.117286	-0.243446
C	7.226441	-0.309679	-1.466476
C	7.263841	-0.466339	1.054999
C	7.297986	-2.545409	-0.339433
H	6.856391	-0.745146	-2.401675
H	6.898697	0.734899	-1.428844
H	8.322375	-0.312556	-1.499167
H	6.922632	-1.015832	1.940021
H	8.360217	-0.470645	1.053795
H	6.936998	0.574059	1.157980
H	8.392792	-2.506963	-0.337945
H	6.987836	-3.164285	0.510546
H	6.987791	-3.046301	-1.263578
N	2.382200	-0.999456	-0.110737
N	0.995042	1.265680	-0.298359

INT4A-dtbpy

G_{sol}(toluene) = -4615.632361 Hartree

C	-2.747259	1.122975	-0.301193
C	-1.027587	2.674964	-0.426446
C	-1.920471	3.721152	-0.644857
C	-3.294865	3.464070	-0.691218
C	-3.689598	2.129475	-0.510698

C	-3.106878	-0.305155	-0.093333	H	-7.029435	-0.776444	0.788395
C	-2.350473	-2.441778	0.404350	H	-8.064168	-2.204109	0.932559
C	-3.636553	-2.969331	0.341746	H	-6.104554	-2.868334	-2.234787
C	-4.716148	-2.132601	0.039932	H	-7.738033	-2.690996	-1.564262
C	-4.414683	-0.780689	-0.179226	H	-6.688900	-1.275614	-1.722187
H	0.043973	2.840821	-0.371849	H	-7.337834	-4.433694	0.147017
H	-1.523182	4.720785	-0.767735	H	-5.716205	-4.726656	-0.491785
H	-4.743415	1.881917	-0.523288	H	-5.947651	-4.383521	1.237372
H	-1.497368	-3.072060	0.629576	N	-2.080746	-1.148808	0.191909
H	-3.768293	-4.027486	0.529336	N	-1.424033	1.409276	-0.262995
H	-5.214453	-0.093920	-0.424202	C	1.698273	-3.462414	-1.137221
C	-4.350650	4.553019	-0.920902	C	0.960725	-1.916097	0.440877
C	-5.145751	4.221367	-2.205123	C	1.693351	-3.932828	-2.472256
C	-5.312750	4.588847	0.289475	C	2.506982	-4.122953	-0.158068
C	-3.719879	5.947328	-1.083658	C	1.724383	-2.513172	1.483660
H	-4.485016	4.183122	-3.078944	C	2.466742	-5.019968	-2.825099
H	-5.663170	3.258424	-2.131852	H	1.077054	-3.411261	-3.199141
H	-5.904133	4.992401	-2.385459	C	3.286742	-5.238541	-0.554464
H	-4.771640	4.815440	1.215252	C	2.489260	-3.610508	1.171600
H	-6.073458	5.364381	0.140682	H	1.703110	-2.079040	2.476289
H	-5.833954	3.635625	0.429024	C	3.268447	-5.678887	-1.862017
H	-4.509031	6.690027	-1.244423	H	2.464760	-5.374446	-3.852493
H	-3.160638	6.248412	-0.190437	H	3.902400	-5.738812	0.189692
H	-3.044546	5.992464	-1.945885	H	3.104638	-4.092616	1.928827
C	-6.166587	-2.621739	-0.055447	H	3.871823	-6.532622	-2.157962
C	-7.025651	-1.855551	0.977459	N	0.939648	-2.378475	-0.783558
C	-6.701465	-2.343794	-1.479743	Rh	-0.107112	-0.272072	0.272255
C	-6.288566	-4.129178	0.227221	Br	-0.371386	0.162569	2.671988
H	-6.655625	-2.017684	1.996294	C	1.595114	0.669261	0.199642

C	2.696144	1.208923	0.071607
Si	4.448346	1.667860	-0.129444
C	5.249398	1.714809	1.608208
H	6.325870	1.894574	1.460307
C	4.540108	3.404520	-0.928476
H	3.996125	4.059460	-0.230629
C	5.309004	0.367187	-1.255968
H	5.163775	0.734867	-2.284118
C	4.669473	-1.032019	-1.178060
H	3.604224	-1.006067	-1.427165
H	5.163549	-1.729327	-1.870745
H	4.751339	-1.460833	-0.171934
C	6.827308	0.284019	-1.003110
H	7.309067	-0.389542	-1.726740
H	7.323990	1.258408	-1.083602
H	7.044201	-0.111324	-0.002790
C	5.084136	0.374587	2.348408
H	5.546171	-0.459841	1.807703
H	5.546110	0.416051	3.345916
H	4.022722	0.132072	2.483392
C	4.684250	2.866151	2.460567
H	3.599199	2.762355	2.591550
H	5.135745	2.871828	3.463475
H	4.874012	3.849355	2.012409
C	5.974663	3.944837	-1.063397
H	6.558288	3.353400	-1.780677
H	5.974991	4.981956	-1.429307
H	6.515370	3.934261	-0.108891
C	3.806526	3.462119	-2.280031
H	2.771407	3.108978	-2.196596
H	3.780785	4.488654	-2.674586
H	4.306337	2.841087	-3.035350

INT4B-dtbpy

G_{sol}(toluene) = -4615.637389 Hartree

C	2.399514	1.190940	0.159652
C	0.433507	2.442303	0.136282
C	1.132322	3.620363	0.376626
C	2.523679	3.598441	0.518857
C	3.139438	2.344636	0.404572
C	3.000205	-0.153222	0.017609
C	2.574445	-2.405088	-0.338642
C	3.929718	-2.718181	-0.292394
C	4.872941	-1.704236	-0.087968
C	4.369926	-0.404658	0.069309
H	-0.643126	2.427659	0.018484
H	0.565814	4.540554	0.443952
H	4.214760	2.267687	0.503868
H	1.823355	-3.168249	-0.511012
H	4.223626	-3.751536	-0.426579
H	5.055933	0.418794	0.221238
C	3.372151	4.849268	0.776311
C	4.155081	4.668435	2.097568
C	4.366942	5.027893	-0.394330
C	2.511446	6.119897	0.887316
H	3.473048	4.525289	2.943710
H	4.832263	3.807920	2.060900

H	4.762464	5.558997	2.297358	C	-1.507018	-3.123706	3.270658
H	3.837207	5.157438	-1.344869	H	-1.003141	-1.744038	4.815574
H	4.987971	5.915534	-0.225634	C	-1.914769	-4.537483	-0.219843
H	5.036340	4.166923	-0.497847	H	-1.235746	-3.465959	-2.016865
H	3.157461	6.985505	1.070591	H	-2.487406	-5.317382	1.698070
H	1.952462	6.315529	-0.034821	H	-1.946115	-3.846189	3.953456
H	1.797998	6.058409	1.717278	H	-2.317464	-5.375196	-0.779927
C	6.385860	-1.954047	-0.039981	Rh	0.095749	-0.506022	-0.353139
C	7.068463	-1.136706	-1.161614	Br	0.543122	-0.225499	-2.899792
C	6.931008	-1.502054	1.334749	C	-1.729074	0.230457	-0.425939
C	6.734270	-3.439785	-0.238659	C	-2.898500	0.612952	-0.372837
H	6.692591	-1.431459	-2.147970	Si	-4.692804	0.844999	-0.182753
H	6.902136	-0.060148	-1.046723	C	-5.558239	-0.622301	-1.060823
H	8.151092	-1.309134	-1.143184	H	-6.633254	-0.543524	-0.835688
H	6.460290	-2.063760	2.149959	C	-5.209688	2.488717	-1.016378
H	8.012671	-1.674055	1.383571	H	-4.894459	2.376826	-2.064827
H	6.756939	-0.435885	1.516272	C	-5.153896	0.881848	1.686007
H	7.821307	-3.569372	-0.198854	H	-5.079838	1.940633	1.981391
H	6.298601	-4.069814	0.545470	C	-4.177520	0.088584	2.573998
H	6.394001	-3.812603	-1.211598	H	-3.144155	0.425944	2.447632
N	2.116852	-1.160068	-0.187491	H	-4.443430	0.187768	3.637365
N	1.044379	1.252872	0.033908	H	-4.192204	-0.981767	2.334596
C	-0.895625	-2.387080	1.077882	C	-6.604445	0.429475	1.943666
C	-0.401897	-0.940583	2.892593	H	-6.876377	0.561882	3.001176
C	-1.489338	-3.387855	1.871966	H	-7.333968	0.988701	1.346314
C	-0.974913	-1.937532	3.745429	H	-6.737264	-0.634036	1.708574
H	-0.031908	-0.018481	3.335722	C	-5.053345	-1.976647	-0.529578
C	-1.305407	-3.472350	-0.933680	H	-5.231712	-2.097778	0.545551
C	-2.007984	-4.502481	1.160843	H	-5.551422	-2.813529	-1.041662

H	-3.974299	-2.079866	-0.695670
C	-5.378854	-0.551552	-2.587776
H	-4.315313	-0.570301	-2.859346
H	-5.860189	-1.408275	-3.082444
H	-5.814517	0.359221	-3.016312
C	-6.728471	2.735429	-1.002116
H	-7.097794	2.903912	0.018022
H	-6.990579	3.628767	-1.587658
H	-7.291461	1.892615	-1.422418
C	-4.454252	3.697555	-0.438011
H	-3.368045	3.553780	-0.486052
H	-4.693831	4.617671	-0.990979
H	-4.720415	3.876092	0.612820
N	-0.805678	-2.438341	-0.285009
C	-0.359097	-1.163894	1.534122

TS5A-dtbpy

G_{sol}(toluene) = -4615.598685 Hartree

C	-2.546186	1.326458	-0.347869
C	-0.763363	2.583239	-1.101470
C	-1.574538	3.693788	-1.326969
C	-2.941976	3.625931	-1.034810
C	-3.413733	2.403784	-0.533725
C	-2.980163	0.009863	0.185800
C	-2.313753	-2.036501	1.039579
C	-3.630723	-2.451605	1.215109
C	-4.681936	-1.603364	0.848906
C	-4.319533	-0.354193	0.326217
H	0.303886	2.616136	-1.299884
H	-1.119371	4.595721	-1.716859
H	-4.458663	2.302753	-0.266489
H	-1.483709	-2.678069	1.314817
H	-3.808727	-3.433958	1.634108
H	-5.090352	0.339206	0.013055
C	-3.906207	4.805200	-1.221208
C	-5.039596	4.389749	-2.187213
C	-4.510662	5.182612	0.151202
C	-3.201546	6.044508	-1.801282
H	-4.638663	4.110677	-3.168667
H	-5.614320	3.539462	-1.804067
H	-5.736903	5.223902	-2.329580
H	-3.726218	5.468619	0.861127
H	-5.196113	6.031433	0.039557
H	-5.076112	4.354553	0.592391
H	-3.926916	6.856766	-1.922532
H	-2.407087	6.408871	-1.140057
H	-2.765145	5.842067	-2.786379
C	-6.162272	-1.978957	0.993988
C	-6.850179	-0.962334	1.934442
C	-6.835900	-1.934558	-0.397435
C	-6.348232	-3.389866	1.579715
H	-6.389650	-0.972285	2.929034
H	-6.789703	0.060438	1.546760
H	-7.911538	-1.213472	2.047609
H	-6.362884	-2.644110	-1.086238
H	-7.895987	-2.200889	-0.309538
H	-6.781323	-0.938702	-0.850411

H	-7.417412	-3.613787	1.663562
H	-5.899178	-4.159282	0.940994
H	-5.914107	-3.476319	2.582420
N	-1.986606	-0.842773	0.531282
N	-1.230031	1.426887	-0.626395
C	0.353112	-3.196879	-1.575692
C	1.481907	-1.735194	-0.114388
C	-0.594422	-3.399619	-2.605373
C	0.981822	-4.327524	-0.959629
C	2.151160	-2.812033	0.553453
C	-0.903055	-4.683526	-3.017666
H	-1.064485	-2.527772	-3.051556
C	0.653956	-5.622144	-1.417543
C	1.893583	-4.082545	0.121435
H	2.817443	-2.596702	1.380984
C	-0.273759	-5.802511	-2.429745
H	-1.635555	-4.833325	-3.806679
H	1.135166	-6.479600	-0.952211
H	2.360452	-4.934236	0.611419
H	-0.522509	-6.803860	-2.769549
N	0.673198	-1.937143	-1.164163
Rh	0.073181	-0.283486	0.134214
Br	0.166951	0.919241	2.340899
C	2.047725	-0.202588	-0.144314
C	3.216773	0.237835	-0.137576
Si	4.937703	0.855071	0.027092
C	5.309228	1.006608	1.893416
H	6.357541	1.333239	1.977972
C	4.990883	2.578184	-0.801260
H	4.215195	3.159439	-0.280430
C	6.093256	-0.392591	-0.859497
H	6.092488	-0.083338	-1.915567
C	5.585966	-1.846313	-0.805953
H	4.579262	-1.946166	-1.227175
H	6.252679	-2.510866	-1.374064
H	5.551033	-2.226204	0.222405
C	7.542458	-0.301618	-0.343077
H	8.203324	-0.961385	-0.922901
H	7.951142	0.713263	-0.412494
H	7.614319	-0.613243	0.706405
C	5.165386	-0.339771	2.625910
H	5.841888	-1.106991	2.231107
H	5.385749	-0.227412	3.696940
H	4.139129	-0.719979	2.544012
C	4.411770	2.071055	2.554157
H	3.349644	1.805605	2.467750
H	4.640202	2.160717	3.625749
H	4.547072	3.063776	2.107774
C	6.336621	3.298022	-0.597854
H	7.150228	2.785368	-1.126926
H	6.294219	4.323133	-0.992147
H	6.618070	3.366023	0.460108
C	4.612445	2.527100	-2.291383
H	3.639668	2.046003	-2.449102
H	4.557976	3.539557	-2.716360
H	5.356759	1.972658	-2.878027

INT5A-dtbpy

G_{sol}(toluene) = -4615.680524 Hartree

C	1.625497	-1.084509	-1.113200
C	1.171729	0.340514	-2.890880
C	2.509070	0.358649	-3.268727
C	3.465527	-0.352985	-2.532723
C	2.979508	-1.087638	-1.444214
C	1.054887	-1.811263	0.027816
C	-0.856672	-2.174638	1.296037
C	-0.187702	-3.059868	2.129344
C	1.167611	-3.350282	1.916090
C	1.768608	-2.696013	0.834337
H	0.419034	0.905109	-3.428186
H	2.782136	0.951621	-4.132799
H	3.667052	-1.657609	-0.830861
H	-1.904157	-1.948031	1.451755
H	-0.744937	-3.514608	2.939231
H	2.813848	-2.876692	0.612919
C	4.965737	-0.344455	-2.850930
C	5.735773	0.206016	-1.627704
C	5.433899	-1.787913	-3.146878
C	5.294633	0.535448	-4.069860
H	5.411630	1.224387	-1.384436
H	5.587393	-0.415440	-0.737879
H	6.811746	0.231720	-1.838538
H	4.902791	-2.203946	-4.010845
H	6.507729	-1.798357	-3.369368
H	5.264285	-2.455875	-2.295173
H	6.373947	0.514950	-4.258060
H	4.794854	0.178078	-4.977556
H	5.007402	1.580674	-3.907182
C	1.982984	-4.305036	2.796133
C	2.573454	-5.433773	1.920071
C	3.133928	-3.516208	3.463287
C	1.125762	-4.945462	3.902373
H	1.779006	-6.003345	1.424098
H	3.241033	-5.043887	1.143999
H	3.154486	-6.127685	2.539503
H	2.743375	-2.712547	4.097893
H	3.736660	-4.184133	4.090750
H	3.802047	-3.064457	2.721716
H	1.744133	-5.621609	4.503359
H	0.706038	-4.193395	4.580390
H	0.299798	-5.534562	3.486902
N	-0.269493	-1.546291	0.255632
N	0.719141	-0.346897	-1.825143
Rh	-1.163218	-0.274309	-1.039034
C	-4.106880	-0.869355	-0.359167
C	-3.057294	0.708154	1.015778
C	-4.040651	-1.787342	-1.438035
C	-5.343163	-0.704326	0.342661
C	-4.256019	0.922040	1.749824
C	-5.155572	-2.511282	-1.800158
H	-3.094450	-1.881003	-1.963285
C	-6.471682	-1.465552	-0.057942
C	-5.386102	0.221626	1.415160
H	-4.247189	1.643353	2.559378
C	-6.381057	-2.352477	-1.107611

H	-5.095995	-3.207629	-2.631890
H	-7.407193	-1.332133	0.479431
H	-6.315471	0.369404	1.958854
H	-7.249792	-2.930578	-1.409903
N	-2.979978	-0.162827	-0.004921
Br	-2.164829	1.266181	-2.765244
C	-1.874675	1.423879	1.363509
C	-0.877595	2.043601	1.701041
Si	0.800053	2.797267	1.860742
C	1.024890	3.985201	0.386342
H	1.992417	4.485622	0.549891
C	2.007633	1.327676	1.712287
H	1.707498	0.847412	0.771145
C	0.949809	3.685074	3.548981
H	1.244050	2.900254	4.262904
C	-0.375117	4.288684	4.050522
H	-1.167525	3.534571	4.118436
H	-0.247698	4.733403	5.047866
H	-0.736789	5.082621	3.385519
C	2.064734	4.749312	3.539550
H	2.199112	5.182977	4.540498
H	3.033676	4.340251	3.228084
H	1.822955	5.575682	2.859197
C	-0.069939	5.066157	0.336125
H	-0.094466	5.677458	1.246231
H	0.092640	5.745635	-0.512216
H	-1.061670	4.615205	0.207310
C	1.093789	3.219485	-0.948162
H	0.175926	2.650571	-1.135019
H	1.224271	3.917465	-1.787882
H	1.931500	2.513967	-0.978244
C	3.480154	1.754768	1.582306
H	3.850448	2.213050	2.508694
H	4.120026	0.884325	1.377505
H	3.639957	2.473550	0.769648
C	1.833924	0.292993	2.836226
H	0.805874	-0.083196	2.887901
H	2.491180	-0.570796	2.673292
H	2.090249	0.712750	3.818088

^{t}BuO^{-}

G_{sol}(toluene) = -232.931993 Hartree

C	0.130787	1.453640	-0.433694
C	-0.000060	-0.000337	0.158157
H	-0.704002	2.063679	-0.059851
H	1.057681	1.907519	-0.054283
H	0.140123	1.521684	-1.536599
C	1.193477	-0.838353	-0.437589
C	-1.323762	-0.612234	-0.437886
H	1.121565	-1.869799	-0.063544
H	1.248744	-0.874433	-1.540589
H	2.139065	-0.423155	-0.060030
H	-1.383614	-0.640104	-1.540914
H	-1.437613	-1.639021	-0.061772
H	-2.180231	-0.034044	-0.062237
O	-0.000546	-0.003578	1.485736

1a

G_{sol}(toluene) = -401.621130 Hartree

C	-2.399751	-0.716069	-0.000004
C	-1.207592	-1.405584	-0.000002
C	0.028123	-0.705444	0.000003
C	0.014537	0.727116	0.000002
C	-1.230949	1.408668	-0.000001
C	-2.412768	0.701751	-0.000002
H	-3.341244	-1.258738	-0.000006
H	-1.174258	-2.491102	-0.000004
C	1.266733	1.394191	0.000000
H	-1.234456	2.496299	-0.000002
H	-3.362798	1.229072	-0.000003
C	2.424480	0.652983	0.000000
C	2.326891	-0.762051	0.000006
H	1.293301	2.481537	-0.000002
H	3.401494	1.126647	-0.000004
H	3.237518	-1.360711	-0.000003
N	1.188889	-1.428053	0.000001

3aa

G_{sol}(toluene) = -1121.833810 Hartree

C	6.117851	1.580839	0.197584
C	4.745836	1.695073	0.211210
C	3.927264	0.542219	0.067324
C	4.553371	-0.737822	-0.090783
C	5.969141	-0.821797	-0.100938
C	6.735570	0.314280	0.040265
H	6.737052	2.466938	0.307807
H	4.252505	2.654962	0.330248
C	3.704078	-1.865568	-0.230579
H	6.437228	-1.795837	-0.222016
H	7.819750	0.244097	0.031697
C	2.343336	-1.695303	-0.211252
C	1.815048	-0.377589	-0.050090
H	4.140801	-2.853942	-0.352034
H	1.661662	-2.532919	-0.315399
N	2.575352	0.704179	0.085056
C	0.393607	-0.197587	-0.034511
C	-0.821364	-0.081085	-0.025895
Si	-2.657819	0.113040	-0.049135
C	-3.377817	-1.508036	-0.753544
H	-4.472470	-1.400642	-0.714356
C	-3.025191	1.573543	-1.221950
H	-2.566679	1.285645	-2.179854
C	-3.267599	0.470960	1.729683
H	-3.184348	1.562370	1.844147
C	-2.403350	-0.172335	2.829578
H	-1.355452	0.138948	2.760926
H	-2.773124	0.109174	3.825699
H	-2.424265	-1.267776	2.774010
C	-4.751110	0.094178	1.915404
H	-5.107893	0.406210	2.906831
H	-5.404170	0.564793	1.171235
H	-4.901161	-0.990352	1.846229
C	-2.988378	-2.734050	0.092242

H	-3.343958	-2.656695	1.125894
H	-3.413600	-3.654139	-0.332945
H	-1.898769	-2.862097	0.126317
C	-2.970146	-1.714107	-2.223416
H	-1.878886	-1.771027	-2.328479
H	-3.385622	-2.652437	-2.617012
H	-3.323529	-0.903250	-2.870993
C	-4.531098	1.784356	-1.463417
H	-5.040936	2.118512	-0.550797
H	-4.698837	2.558987	-2.224656
H	-5.032764	0.872182	-1.809668
C	-2.353636	2.879126	-0.761315
H	-1.274003	2.755264	-0.617163
H	-2.501764	3.676960	-1.502491
H	-2.777255	3.238495	0.185731

INT1A-IMes

G_{sol}(toluene) = -1668.467921 Hartree

C	0.427066	-0.696784	-1.027681
C	1.367269	-1.217313	-3.049766
C	0.043435	-1.485072	-3.142950
H	2.170687	-1.323576	-3.761900
H	-0.552539	-1.875609	-3.953132
N	1.590196	-0.736513	-1.759817
N	-0.519227	-1.162370	-1.909256
C	2.863450	-0.275549	-1.283180
C	3.284278	1.014057	-1.642612
C	3.656263	-1.133537	-0.502543
C	4.517261	1.462107	-1.154686
C	4.880567	-0.639612	-0.040688
C	5.321700	0.654621	-0.343381
H	4.855225	2.462719	-1.416759
H	5.507719	-1.285560	0.570209
C	-1.922430	-1.259350	-1.616169
C	-2.748992	-0.176315	-1.946705
C	-2.420612	-2.433139	-1.026164
C	-4.114169	-0.279829	-1.657366
C	-3.791188	-2.487477	-0.754669
C	-4.650488	-1.422476	-1.055607
H	-4.766992	0.556909	-1.895819
H	-4.198078	-3.385401	-0.293884
C	-2.168969	1.075538	-2.555799
H	-1.375125	1.481151	-1.918945
H	-1.722812	0.880472	-3.538975
H	-2.936274	1.846160	-2.676747
C	-1.498180	-3.566227	-0.664674
H	-0.866613	-3.857431	-1.513354
H	-0.827378	-3.257064	0.151726
H	-2.067034	-4.446010	-0.345332
C	-6.119139	-1.503773	-0.710209
H	-6.280169	-1.346944	0.365073
H	-6.702733	-0.746102	-1.244260
H	-6.535916	-2.487752	-0.955767
C	3.196122	-2.530540	-0.180815
H	2.287097	-2.512375	0.437901
H	2.953085	-3.085329	-1.096667
H	3.977122	-3.082973	0.352360

C	2.436432	1.890822	-2.533512
H	2.463675	1.547662	-3.575776
H	1.386179	1.883618	-2.224873
H	2.790925	2.926385	-2.516472
C	6.630901	1.169273	0.207158
H	6.503461	1.557627	1.226731
H	7.385364	0.375860	0.256993
H	7.034420	1.983486	-0.404852
Rh	0.155766	-0.246348	0.835546
C	-3.760969	2.173824	1.120154
C	-2.527940	1.600275	0.893319
C	-1.369178	2.410221	0.793133
C	-1.499338	3.831567	0.905060
C	-2.785061	4.388434	1.139942
C	-3.892765	3.577462	1.250834
H	-4.641404	1.540671	1.183670
H	-2.405225	0.527674	0.772750
C	-0.333594	4.627434	0.775161
H	-2.875382	5.468120	1.230904
H	-4.871914	4.013520	1.429280
C	0.875357	4.011241	0.547659
C	0.930259	2.605520	0.459950
H	-0.410526	5.708581	0.856905
H	1.793425	4.580101	0.440047
H	1.873607	2.100447	0.291665
N	-0.136226	1.812478	0.582973
O	0.443061	-2.173965	1.441144
C	0.454138	-2.181979	2.847204
C	1.908756	-2.228671	3.361724
C	-0.233668	-0.901639	3.388649
C	-0.316992	-3.417733	3.339647
H	2.409824	-3.122475	2.972587
H	2.458080	-1.350602	3.001254
H	1.965359	-2.252584	4.458955
H	-1.298055	-0.881293	3.128189
H	-0.148181	-0.811446	4.479387
H	0.267387	0.017765	3.004884
H	-0.332773	-3.489923	4.435753
H	-1.351173	-3.381895	2.977025
H	0.149032	-4.326633	2.940726

INT2A-IMes

G_{sol}(toluene) = -4963.319996 Hartree

C	2.614508	0.184274	-1.895788
C	0.741245	1.181927	-2.845354
C	3.184739	-0.768943	-1.014165
C	3.480765	0.973405	-2.718260
C	1.527444	1.981284	-3.702662
H	-0.338184	1.258435	-2.880244
C	4.552361	-0.918795	-0.946511
H	2.530319	-1.387612	-0.410479
C	4.886769	0.807796	-2.606255
C	2.896243	1.890075	-3.627834
H	1.034922	2.660271	-4.390643
C	5.413480	-0.121122	-1.737853
H	4.971471	-1.660327	-0.275422
H	5.534062	1.420934	-3.228323

H	3.538134	2.500608	-4.257789	H	-0.899813	1.789783	3.253881
H	6.489574	-0.250441	-1.661881	H	-0.683039	2.998721	4.529605
N	1.243503	0.329512	-1.961867	H	-1.708219	3.352322	3.134492
O	0.166589	-2.499620	-1.153636	C	0.625117	4.927787	3.069286
C	0.118672	-2.931806	-2.500017	H	0.733994	5.052777	4.156313
C	1.452580	-2.704474	-3.243313	H	1.511724	5.369798	2.600683
C	-0.143752	-4.454609	-2.471765	H	-0.240671	5.527132	2.762592
C	-1.017811	-2.233964	-3.272353	C	-1.956293	4.690356	0.368466
H	1.625878	-1.645653	-3.441772	H	-2.056224	4.852426	1.447683
H	2.286220	-3.081936	-2.640950	H	-2.453205	5.532266	-0.136038
H	1.460074	-3.228439	-4.208580	H	-2.516707	3.783743	0.113312
H	-1.070729	-4.681445	-1.936402	C	-0.388446	4.475497	-1.602999
H	-0.224536	-4.868635	-3.485353	H	-0.919282	3.589559	-1.968174
H	0.677430	-4.969815	-1.959812	H	-0.846795	5.351911	-2.084343
H	-1.090074	-2.603401	-4.304394	H	0.648216	4.411228	-1.954311
H	-1.979790	-2.399225	-2.776940	C	2.952633	4.486789	0.123836
H	-0.846075	-1.153469	-3.306920	H	3.126111	4.886675	1.130980
Rh	-0.007992	-0.591204	-0.407258	H	3.937593	4.403503	-0.358776
Br	-2.772962	1.309885	-1.474913	H	2.378814	5.235549	-0.434654
C	-1.201731	0.968317	-0.424989	C	3.126371	2.096138	0.921151
C	-0.329946	1.551129	0.307876	H	2.655820	1.110977	0.968372
Si	0.448770	3.150198	0.806956	H	4.100488	1.969072	0.428840
C	-0.485061	4.576369	-0.070014	H	3.323286	2.425343	1.950120
H	0.032177	5.497627	0.240474	C	-0.839538	-1.415632	1.227884
C	2.255369	3.115448	0.167546	C	-0.879012	-2.498427	3.241414
H	2.162504	2.764609	-0.869356	C	-2.158600	-2.212249	2.912814
C	0.443288	3.439448	2.707979	H	-0.450166	-2.995288	4.097554
H	1.329840	2.908242	3.080670	H	-3.091704	-2.415245	3.414411
C	-0.778041	2.859922	3.442384	N	-0.084686	-2.011766	2.207113

N	-2.123064	-1.550989	1.684077
C	1.348570	-2.159027	2.202027
C	1.908890	-3.340534	1.693085
C	2.131740	-1.144930	2.774459
C	3.304418	-3.459942	1.722482
C	3.518618	-1.308434	2.778176
C	4.122443	-2.458808	2.256149
H	3.758981	-4.362669	1.320467
H	4.139732	-0.521598	3.200297
C	-3.342047	-1.257243	0.974396
C	-3.787073	-2.185969	0.019853
C	-4.092931	-0.128353	1.326411
C	-5.005649	-1.934519	-0.615280
C	-5.318561	0.066174	0.680554
C	-5.784405	-0.814737	-0.298773
H	-5.361778	-2.638951	-1.364107
H	-5.909670	0.943226	0.934856
C	-2.980909	-3.423501	-0.276063
H	-1.954311	-3.169158	-0.559593
H	-2.918900	-4.072197	0.608806
H	-3.437648	-4.003087	-1.084193
C	-3.565358	0.887356	2.304666
H	-3.206332	0.429110	3.232795
H	-2.716748	1.417030	1.859849
H	-4.332568	1.624605	2.561068
C	-7.081964	-0.551143	-1.024852
H	-6.898656	-0.029473	-1.974137
H	-7.606947	-1.482871	-1.264891
H	-7.756444	0.076914	-0.432600
C	1.481310	0.078265	3.360694
H	0.899995	0.600010	2.597401
H	0.788009	-0.184700	4.169243
H	2.228049	0.767759	3.762634
C	1.036017	-4.423117	1.119515
H	0.303061	-4.780989	1.853754
H	0.490649	-4.017550	0.258918
H	1.636022	-5.278742	0.793303
C	5.624416	-2.616419	2.295612
H	6.130903	-1.681026	2.030241
H	5.965892	-2.892299	3.302450
H	5.967531	-3.397183	1.608336

TS3A-IMes

G_{sol}(toluene) = -4963.277477 Hartree

C	-1.963432	2.759282	0.624192
C	0.288736	2.548857	1.161636
C	-3.201190	2.160871	0.277712
C	-1.899828	4.178528	0.795243
C	0.427173	3.938471	1.367422
H	1.140306	1.892664	1.281078
C	-4.320375	2.941565	0.087185
H	-3.236777	1.080621	0.175339
C	-3.072073	4.953510	0.586242
C	-0.662570	4.754273	1.179083
H	1.394416	4.334407	1.657757
C	-4.258335	4.348807	0.236111
H	-5.261130	2.466482	-0.173848

H	-3.012946	6.031613	0.713916	C	3.175634	4.067981	-0.864792
H	-0.593359	5.829985	1.319943	H	2.147387	3.695106	-0.896277
H	-5.151385	4.947918	0.080469	H	3.150810	5.089581	-0.458597
N	-0.849422	1.962999	0.809683	H	3.528017	4.142085	-1.900501
O	-1.330100	-0.900592	2.189386	C	5.518300	3.756970	0.035527
C	-1.284300	-0.302018	3.461834	H	5.499981	4.778264	0.441605
C	-2.213819	0.924619	3.585346	H	6.197409	3.168534	0.662589
C	-1.791302	-1.369891	4.461181	H	5.964009	3.817089	-0.965250
C	0.156732	0.088928	3.843910	C	5.018837	1.841989	-3.228629
H	-1.834832	1.774249	3.017005	H	5.058815	2.918546	-3.029421
H	-3.209145	0.673900	3.200407	H	5.735885	1.630785	-4.034830
H	-2.318656	1.243812	4.631347	H	4.015898	1.613774	-3.611924
H	-1.169069	-2.269130	4.417619	C	5.419275	-0.489297	-2.333262
H	-1.778179	-0.995551	5.493540	H	4.445010	-0.869762	-2.665935
H	-2.819934	-1.658645	4.215812	H	6.134933	-0.659127	-3.150336
H	0.216547	0.501673	4.861020	H	5.740652	-1.100441	-1.482175
H	0.811286	-0.787384	3.785679	C	5.810931	0.401964	1.574425
H	0.543692	0.838118	3.149422	H	5.842674	1.366256	2.097684
Rh	-0.905452	-0.143581	0.308379	H	6.018408	-0.373783	2.325145
Br	-0.479022	0.660873	-2.081876	H	6.638444	0.387044	0.853363
C	1.212616	0.710162	-1.356369	C	3.308264	0.170217	1.947337
C	2.402608	0.938045	-1.164916	H	2.347199	-0.102707	1.499454
Si	4.094663	1.325250	-0.560891	H	3.509249	-0.553041	2.749499
C	5.337314	1.005962	-1.975927	H	3.199798	1.153729	2.425970
H	6.322235	1.319799	-1.597016	C	-1.128216	-2.043127	-0.281997
C	4.443665	0.163643	0.909730	C	-2.219114	-3.915462	-1.033756
H	4.455842	-0.836696	0.460645	C	-0.948794	-4.278555	-0.741247
C	4.095063	3.166291	-0.020375	H	-3.058934	-4.481090	-1.406267
H	3.698773	3.155429	1.007706	H	-0.442475	-5.229155	-0.803060

N	-2.315078	-2.555806	-0.754916
N	-0.293358	-3.130453	-0.294065
C	-3.492102	-1.769566	-1.008837
C	-4.382186	-1.512264	0.045560
C	-3.719760	-1.295754	-2.312078
C	-5.491435	-0.700617	-0.224890
C	-4.838296	-0.486213	-2.529673
C	-5.729660	-0.170680	-1.496912
H	-6.183112	-0.476968	0.584273
H	-5.019273	-0.095488	-3.529214
C	1.103811	-3.153628	0.051685
C	1.467212	-3.406704	1.383714
C	2.054675	-3.026252	-0.970922
C	2.829852	-3.497335	1.681907
C	3.405460	-3.149939	-0.626921
C	3.811558	-3.379002	0.691207
H	3.129647	-3.676569	2.712401
H	4.154925	-3.055042	-1.407998
C	0.408654	-3.571806	2.438792
H	-0.263464	-2.703386	2.435641
H	-0.204224	-4.463502	2.247028
H	0.858153	-3.680867	3.431245
C	1.642991	-2.750646	-2.395075
H	0.861964	-3.439124	-2.737757
H	1.245233	-1.735184	-2.490643
H	2.496935	-2.839583	-3.073763
C	5.275121	-3.502529	1.044331
H	5.917745	-3.149867	0.230605
H	5.518511	-2.921689	1.941645
H	5.547695	-4.545995	1.252478
C	-2.810861	-1.669723	-3.458642
H	-1.766878	-1.757349	-3.148244
H	-3.099148	-2.640443	-3.884148
H	-2.870121	-0.930791	-4.264653
C	-4.132095	-2.074859	1.417742
H	-4.057536	-3.169939	1.387414
H	-3.180194	-1.694135	1.818930
H	-4.944804	-1.809080	2.102168
C	-6.928583	0.708109	-1.767139
H	-7.451782	0.973268	-0.842443
H	-6.635439	1.638079	-2.269628
H	-7.650981	0.204272	-2.422981

INT3A-IMes

G_{sol}(toluene) = -4963.342894 Hartree

C	-2.576802	2.527211	0.183876
C	-0.492622	2.907070	-0.782975
C	-3.468367	1.685306	0.896164
C	-3.003828	3.849204	-0.164952
C	-0.832588	4.230064	-1.133865
H	0.481845	2.507614	-1.029953
C	-4.730621	2.130801	1.225923
H	-3.127439	0.708937	1.217360
C	-4.313299	4.271206	0.185709
C	-2.089014	4.697078	-0.837772
H	-0.097560	4.844213	-1.642917
C	-5.163879	3.428767	0.865573

H	-5.398192	1.472651	1.773549	H	3.976612	3.073736	1.847299
H	-4.625870	5.275602	-0.089051	C	2.473081	4.041442	0.673865
H	-2.394682	5.705078	-1.106802	H	1.637559	3.450396	1.056527
H	-6.163198	3.759596	1.134294	H	2.507117	4.980926	1.245269
N	-1.314865	2.082184	-0.149883	H	2.241482	4.307648	-0.364882
O	-1.128060	-0.239296	1.935623	C	4.958443	4.194433	0.297126
C	-0.573099	0.465507	3.038226	H	4.999984	5.119643	0.889966
C	-0.336783	1.960487	2.772915	H	5.941345	3.716360	0.370300
C	-1.612936	0.318239	4.167661	H	4.816117	4.490282	-0.750153
C	0.756159	-0.180217	3.467638	C	3.251233	2.511462	-2.716354
H	0.379014	2.088946	1.959841	H	3.065557	3.514408	-2.313253
H	-1.267330	2.468442	2.506732	H	3.551966	2.632819	-3.767309
H	0.070639	2.446294	3.668392	H	2.299525	1.965461	-2.708906
H	-1.790328	-0.739478	4.390940	C	4.613189	0.392329	-2.577635
H	-1.268809	0.809152	5.086919	H	3.728438	-0.252662	-2.537993
H	-2.565436	0.770663	3.868194	H	4.883308	0.516834	-3.636662
H	1.240684	0.390128	4.271107	H	5.437612	-0.141086	-2.089272
H	0.588640	-1.197776	3.831897	C	6.565127	0.933234	0.713044
H	1.433835	-0.221777	2.611587	H	6.722243	1.835835	1.316829
Rh	-0.813361	-0.058023	-0.060456	H	7.262982	0.172200	1.091999
Br	-0.820218	0.104459	-2.607292	H	6.865009	1.166786	-0.316255
C	1.057548	0.304504	-0.014503	C	4.726800	0.141040	2.264621
C	2.200006	0.761723	0.004906	H	3.727998	-0.300608	2.336432
Si	3.840955	1.561394	-0.074075	H	5.435960	-0.562580	2.725432
C	4.325368	1.751233	-1.916973	H	4.736296	1.050597	2.880376
H	5.251289	2.346245	-1.936506	C	-0.730800	-2.076830	-0.225006
C	5.111473	0.438421	0.806278	C	-1.674141	-4.120142	-0.584043
H	5.041010	-0.504836	0.251831	C	-0.334407	-4.291530	-0.531744
C	3.808648	3.286713	0.779928	H	-2.485313	-4.814461	-0.736519

H	0.282770	-5.170954	-0.624208
N	-1.904303	-2.763601	-0.392544
N	0.231372	-3.034961	-0.308471
C	-3.224648	-2.185669	-0.323876
C	-3.822861	-2.049884	0.943888
C	-3.902311	-1.856143	-1.510736
C	-5.088055	-1.458147	1.004227
C	-5.160777	-1.256667	-1.390786
C	-5.757606	-1.028483	-0.147240
H	-5.560759	-1.333424	1.975871
H	-5.689920	-0.971027	-2.297403
C	1.651509	-2.928528	-0.078571
C	2.117133	-3.186940	1.219502
C	2.511595	-2.732872	-1.165678
C	3.498906	-3.212101	1.422102
C	3.886726	-2.785544	-0.914766
C	4.397445	-3.024924	0.365045
H	3.881742	-3.395804	2.423471
H	4.574290	-2.633203	-1.742549
C	1.150909	-3.478672	2.339915
H	0.316858	-2.772071	2.342249
H	0.725241	-4.486314	2.238132
H	1.650734	-3.425106	3.311333
C	1.976680	-2.476232	-2.549180
H	1.231263	-3.226590	-2.840035
H	1.476065	-1.503164	-2.600452
H	2.782456	-2.492771	-3.289239
C	5.886560	-3.103545	0.604488
H	6.167795	-2.597306	1.534509
H	6.217143	-4.147581	0.691299
H	6.452060	-2.646537	-0.214114
C	-3.368322	-2.215481	-2.874107
H	-2.286472	-2.342864	-2.880154
H	-3.830704	-3.152862	-3.215221
H	-3.611970	-1.439144	-3.604656
C	-3.127102	-2.539682	2.185729
H	-2.840298	-3.594643	2.091074
H	-2.219898	-1.949094	2.356304
H	-3.779054	-2.441543	3.059279
C	-7.089230	-0.324523	-0.057819
H	-6.963669	0.753740	-0.221174
H	-7.788720	-0.687873	-0.819664
H	-7.554936	-0.459965	0.924230

TS2B-IMes

G_{sol}(toluene) = -1668.422172 Hartree

C	-0.841068	-1.489311	-0.291569
C	-0.845010	-3.691351	-0.924368
C	-2.056801	-3.180299	-1.249939
H	-0.430882	-4.680114	-1.042884
H	-2.921564	-3.628131	-1.714049
N	-0.105912	-2.654324	-0.349619
N	-2.037141	-1.842418	-0.867110
C	1.219989	-2.812784	0.180817
C	2.307565	-2.968128	-0.692010
C	1.387444	-2.820250	1.580270
C	3.586805	-3.083167	-0.133557

C	2.683425	-2.932511	2.084334	H	2.486674	-4.006764	-2.574869
C	3.798315	-3.049714	1.244736	H	1.137234	-2.867514	-2.530957
H	4.438254	-3.192994	-0.802279	H	2.796508	-2.270192	-2.670434
H	2.826954	-2.930555	3.162541	C	5.192241	-3.120401	1.822048
C	-3.132634	-0.917606	-0.959520	H	5.574430	-2.114598	2.039993
C	-3.207006	-0.054384	-2.062533	H	5.209141	-3.684074	2.761449
C	-4.064512	-0.876784	0.090053	H	5.893199	-3.594565	1.127086
C	-4.267498	0.854786	-2.105626	C	4.424375	4.207444	-0.904954
C	-5.105587	0.049880	0.002102	C	3.501785	3.536382	-0.132302
C	-5.220368	0.924957	-1.083890	C	2.802380	2.416780	-0.658543
H	-4.342851	1.533975	-2.951843	C	3.077036	1.996378	-1.999776
H	-5.835083	0.099906	0.807240	C	4.032659	2.705982	-2.768717
C	-2.149780	-0.078439	-3.138731	C	4.693637	3.791101	-2.232910
H	-1.216969	0.358711	-2.762404	H	4.951869	5.065625	-0.497154
H	-1.921055	-1.100412	-3.461990	H	3.277489	3.838571	0.885921
H	-2.467155	0.496880	-4.013484	C	2.364441	0.864171	-2.475352
C	-3.915306	-1.785753	1.286768	H	4.235005	2.381247	-3.787208
H	-3.925125	-2.843760	0.998295	H	5.423895	4.332532	-2.828148
H	-2.961321	-1.603206	1.796613	C	1.460668	0.235824	-1.657858
H	-4.721286	-1.620104	2.007001	C	1.211934	0.756172	-0.344295
C	-6.327195	1.951951	-1.129794	H	2.540723	0.507587	-3.488665
H	-6.019099	2.875229	-0.622166	H	0.910818	-0.629365	-1.997929
H	-6.588944	2.216324	-2.159917	N	1.890106	1.789187	0.133501
H	-7.232051	1.590687	-0.629185	O	-1.147949	1.875851	1.580258
C	0.208750	-2.703450	2.513855	C	-0.676291	2.752204	2.569490
H	-0.151873	-1.666785	2.541439	C	0.463725	2.095190	3.370323
H	-0.630181	-3.326280	2.183350	C	-0.174252	4.042559	1.892005
H	0.485437	-3.003051	3.529141	C	-1.863227	3.067781	3.498907
C	2.163933	-3.028798	-2.196570	H	0.106863	1.163058	3.825378

H	1.293026	1.860930	2.696193
H	0.828388	2.755546	4.168357
H	-0.989642	4.501119	1.320821
H	0.192534	4.772827	2.625716
H	0.635175	3.794482	1.200091
H	-1.579864	3.778101	4.285956
H	-2.686817	3.498694	2.918491
H	-2.222885	2.145883	3.970683
Rh	-0.596658	0.295553	0.577621
H	0.997401	-0.097730	0.674054

INT2B-IMes

G_{sol}(toluene) = -1668.455787 Hartree

C	0.615833	-1.370170	-0.367669
C	1.431737	-2.851522	-1.903525
C	0.153300	-3.212573	-1.638486
H	2.175431	-3.280280	-2.556537
H	-0.454624	-4.022093	-2.011261
N	1.701620	-1.726492	-1.126975
N	-0.332013	-2.300263	-0.706216
C	2.950703	-1.015986	-1.166694
C	3.208049	-0.176334	-2.262447
C	3.869759	-1.178565	-0.117820
C	4.415309	0.529873	-2.275130
C	5.062604	-0.451849	-0.177903
C	5.349580	0.412940	-1.240013
H	4.628713	1.188845	-3.113853
H	5.783984	-0.562836	0.628797
C	-1.676596	-2.294717	-0.194011
C	-2.690239	-1.731626	-0.986078
C	-1.928012	-2.824615	1.080283
C	-3.984728	-1.702227	-0.463458
C	-3.241044	-2.767880	1.560969
C	-4.277351	-2.204691	0.809835
H	-4.778245	-1.255192	-1.057252
H	-3.455368	-3.171896	2.547906
C	-2.385479	-1.150143	-2.343714
H	-1.596818	-0.393297	-2.277411
H	-2.038986	-1.917025	-3.047672
H	-3.272135	-0.674132	-2.771099
C	-0.825673	-3.425506	1.919928
H	-0.122037	-4.007758	1.315252
H	-0.244771	-2.647181	2.432226
H	-1.239800	-4.081661	2.691905
C	-5.675276	-2.107478	1.371904
H	-5.815489	-1.154582	1.899895
H	-6.432117	-2.156131	0.581125
H	-5.879240	-2.909499	2.089806
C	3.575621	-2.078063	1.055932
H	2.897743	-1.580640	1.761856
H	3.093238	-3.011701	0.746697
H	4.493938	-2.327375	1.596676
C	2.220516	-0.042477	-3.396750
H	2.213085	-0.938795	-4.030254
H	1.199276	0.098920	-3.029851
H	2.473671	0.810442	-4.034042
C	6.626769	1.218379	-1.253650

H	6.504773	2.152937	-0.689802
H	7.454242	0.667897	-0.792221
H	6.923240	1.488217	-2.273034
C	-4.903181	2.590867	-1.311865
C	-4.036004	1.892439	-0.496123
C	-2.640016	1.917306	-0.746719
C	-2.146010	2.683517	-1.849848
C	-3.061344	3.393297	-2.666153
C	-4.416071	3.346748	-2.405683
H	-5.971956	2.563447	-1.114752
H	-4.390788	1.303683	0.344180
C	-0.738590	2.674544	-2.067842
H	-2.678421	3.974033	-3.502852
H	-5.112616	3.891502	-3.037280
C	0.078370	1.933381	-1.252021
C	-0.512153	1.172148	-0.189736
H	-0.324587	3.255986	-2.889801
H	1.152649	1.905825	-1.396911
N	-1.803467	1.185573	0.049949
O	0.343865	0.913365	2.837831
C	0.987571	2.064655	3.335765
C	2.510160	1.841922	3.419551
C	0.685000	3.276108	2.434679
C	0.423658	2.314924	4.746694
H	2.725832	0.964631	4.041995
H	2.918744	1.657061	2.419042
H	3.028491	2.708057	3.851995
H	-0.398462	3.394512	2.320526
H	1.098150	4.204636	2.850506
H	1.112454	3.122881	1.437304
H	0.879824	3.194346	5.219226
H	-0.659852	2.471342	4.693776
H	0.609685	1.443269	5.385401
Rh	0.440339	0.035203	1.069405
H	1.809376	0.567061	0.653398

TS3B-IMes

G_{sol}(toluene) = -1668.423998 Hartree

C	-0.883497	-1.369277	-0.064764
C	-1.888011	-3.398785	0.286119
C	-0.560559	-3.623985	0.142279
H	-2.714137	-4.069589	0.463786
H	0.016234	-4.535186	0.162883
N	-2.070557	-2.025751	0.159857
N	0.042248	-2.384851	-0.070457
C	-3.346183	-1.365681	0.224335
C	-3.829251	-0.950334	1.475767
C	-4.059610	-1.146976	-0.963868
C	-5.064663	-0.300142	1.515098
C	-5.292698	-0.491217	-0.873164
C	-5.809875	-0.061289	0.353093
H	-5.451763	0.035682	2.474694
H	-5.857824	-0.308414	-1.784313
C	1.455658	-2.247916	-0.304038
C	2.332450	-2.401748	0.782922
C	1.917623	-1.987584	-1.601772
C	3.702334	-2.277786	0.541261

C	3.299134	-1.877543	-1.792619	C	4.232601	1.557551	-0.855999
C	4.204807	-2.014277	-0.737913	C	3.174322	1.284021	0.048693
H	4.393267	-2.368033	1.376381	C	3.464311	1.211417	1.447521
H	3.672586	-1.659095	-2.790382	C	4.795913	1.402699	1.892976
C	1.816011	-2.679980	2.173386	C	5.808026	1.662292	0.990696
H	0.972835	-2.028989	2.423519	H	6.322951	1.948141	-1.096533
H	1.468230	-3.716019	2.277347	H	3.995789	1.605525	-1.914963
H	2.602064	-2.517321	2.917005	C	2.373829	0.935141	2.318579
C	0.966805	-1.778648	-2.752239	H	5.006607	1.342770	2.958955
H	0.135837	-2.492391	-2.734214	H	6.826943	1.809441	1.338889
H	0.533160	-0.770835	-2.709495	C	1.121182	0.732982	1.799865
H	1.485098	-1.878559	-3.711205	C	0.918425	0.814126	0.377976
C	5.691458	-1.875108	-0.959147	H	2.550766	0.877465	3.391385
H	6.187931	-2.855173	-0.951178	H	0.275662	0.517892	2.444695
H	5.911795	-1.398641	-1.919694	N	1.913661	1.087875	-0.443438
H	6.149016	-1.268031	-0.170846	O	-1.532344	2.565986	-0.656399
C	-3.500828	-1.572220	-2.300803	C	-0.948328	3.856256	-0.439678
H	-2.662397	-0.930208	-2.601095	C	-0.429685	3.999283	0.998267
H	-3.121003	-2.599829	-2.276893	C	0.194557	4.051255	-1.445890
H	-4.264181	-1.506370	-3.082033	C	-2.070793	4.873734	-0.694186
C	-3.013251	-1.166239	2.724980	H	-1.235595	3.799020	1.715493
H	-2.745935	-2.221030	2.860857	H	0.385542	3.297396	1.191635
H	-2.074678	-0.601306	2.669242	H	-0.056750	5.014967	1.178670
H	-3.559478	-0.835493	3.613501	H	-0.188430	3.961907	-2.469471
C	-7.154888	0.620398	0.431937	H	0.658695	5.040230	-1.336514
H	-7.944363	-0.095626	0.698606	H	0.960345	3.283533	-1.300839
H	-7.159949	1.406845	1.194880	H	-1.708498	5.904048	-0.587448
H	-7.432897	1.074315	-0.525027	H	-2.469317	4.750071	-1.707486
C	5.519320	1.742685	-0.393822	H	-2.891727	4.718803	0.015764

Rh	-0.825237	0.585602	-0.455235
H	-1.595350	1.704571	0.441391

INT3B-IMes

G_{sol}(toluene) = -1434.985939 Hartree

C	0.987687	-1.169839	0.000329
C	2.549621	-2.857698	0.000022
C	1.326374	-3.437936	-0.000101
H	3.542802	-3.279753	0.000099
H	1.027479	-4.474629	-0.000250
N	2.333585	-1.482643	0.000282
N	0.383564	-2.405849	0.000069
C	3.351045	-0.469223	0.000733
C	3.826153	0.019342	-1.229579
C	3.824281	0.019910	1.230393
C	4.803914	1.018211	-1.203015
C	4.802733	1.019588	1.204271
C	5.300714	1.532227	0.001178
H	5.184987	1.405393	-2.145524
H	5.182152	1.407123	2.147126
C	-1.030680	-2.648807	-0.000008
C	-1.695583	-2.787743	-1.227947
C	-1.695735	-2.787405	1.227814
C	-3.062911	-3.082581	-1.203227
C	-3.063127	-3.082240	1.202983
C	-3.762450	-3.235404	-0.000124
H	-3.593809	-3.190493	-2.146554
H	-3.594118	-3.189879	2.146277
C	-0.959243	-2.591707	-2.528887
H	-0.607325	-1.556476	-2.614441
H	-0.075312	-3.237019	-2.596691
H	-1.606968	-2.807719	-3.384102
C	-0.959586	-2.591073	2.528815
H	-0.075592	-3.236274	2.596842
H	-0.607778	-1.555788	2.614178
H	-1.607397	-2.806980	3.383992
C	-5.246778	-3.514315	-0.000477
H	-5.821250	-2.577965	-0.007393
H	-5.548936	-4.086722	-0.884437
H	-5.551559	-4.075475	0.889726
C	3.279724	-0.509492	2.533862
H	2.220366	-0.247257	2.643114
H	3.345033	-1.602602	2.585885
H	3.826279	-0.092430	3.385188
C	3.282566	-0.511700	-2.532767
H	3.351684	-1.604590	-2.584979
H	2.222233	-0.253145	-2.641199
H	3.827146	-0.092640	-3.384373
C	6.331762	2.635620	-0.002899
H	5.853311	3.617245	-0.119926
H	6.901580	2.659138	0.932191
H	7.040366	2.520737	-0.831010
C	-2.884549	5.506012	0.000097
C	-1.870521	4.570368	0.000477
C	-2.180760	3.186993	0.000004
C	-3.554413	2.772319	-0.000854
C	-4.567462	3.762500	-0.001213

C	-4.242755	5.104504	-0.000754
H	-2.640795	6.565444	0.000454
H	-0.824643	4.864669	0.001135
C	-3.825896	1.372338	-0.001283
H	-5.608513	3.446381	-0.001859
H	-5.026894	5.856588	-0.001036
C	-2.806035	0.449806	-0.000933
C	-1.470738	0.944537	-0.000120
H	-4.863819	1.043227	-0.001902
H	-3.000429	-0.613831	-0.001197
N	-1.202034	2.243572	0.000335
Rh	0.395551	0.686388	0.000780

TS2E-IMes

G_{sol}(toluene) = -1668.427354 Hartree

C	-0.149215	-1.483717	0.038556
C	0.552694	-3.493626	0.871161
C	-0.780134	-3.409546	1.098833
H	1.268654	-4.266380	1.102420
H	-1.471492	-4.092390	1.567572
N	0.928923	-2.318713	0.222052
N	-1.193937	-2.183184	0.587289
C	2.273372	-2.018881	-0.185965
C	2.598791	-2.066506	-1.553801
C	3.222676	-1.679091	0.791900
C	3.894818	-1.702758	-1.930027
C	4.505873	-1.323695	0.362708
C	4.855749	-1.311130	-0.990660
H	4.158490	-1.723716	-2.985217
H	5.246538	-1.041998	1.107962
C	-2.544982	-1.689150	0.611872
C	-3.388501	-1.954403	-0.477564
C	-2.966444	-0.941755	1.723767
C	-4.692732	-1.450111	-0.429822
C	-4.277420	-0.459126	1.724231
C	-5.151863	-0.698212	0.656466
H	-5.360770	-1.642750	-1.266281
H	-4.620428	0.127998	2.573280
C	-2.895748	-2.721659	-1.680507
H	-2.244191	-2.093842	-2.302458
H	-2.313789	-3.604653	-1.394599
H	-3.732543	-3.048665	-2.305402
C	-2.010804	-0.638297	2.848549
H	-1.618809	-1.555959	3.305557
H	-1.151226	-0.073189	2.469207
H	-2.501184	-0.050571	3.630859
C	-6.545701	-0.117760	0.661684
H	-6.536982	0.912242	0.280687
H	-7.226533	-0.695222	0.026966
H	-6.965658	-0.086099	1.673413
C	2.896183	-1.722057	2.264616
H	1.859280	-1.442552	2.456464
H	3.053272	-2.730851	2.670427
H	3.541513	-1.038317	2.824631
C	1.585016	-2.477570	-2.591593
H	1.007637	-3.351875	-2.270022
H	0.866359	-1.667591	-2.771636

H	2.075014	-2.718088	-3.540409
C	6.228528	-0.864720	-1.432971
H	6.978853	-1.031977	-0.652527
H	6.552247	-1.392957	-2.336807
H	6.231350	0.208898	-1.664757
C	1.791862	2.155533	-1.421030
C	1.190748	1.382163	-0.424206
C	1.543669	1.659053	0.945862
C	2.467451	2.724088	1.233067
C	3.038568	3.479884	0.177981
C	2.699215	3.195294	-1.125873
H	1.547004	1.950441	-2.458442
C	2.763072	2.995961	2.593956
H	3.735087	4.279806	0.417009
H	3.126970	3.771549	-1.942461
C	2.163980	2.256202	3.585745
H	3.458180	3.797912	2.833014
H	2.361069	2.444148	4.637009
N	0.969657	0.937155	1.950930
O	-1.654125	1.528646	-1.802497
C	-2.259319	2.718082	-1.343290
C	-2.416862	2.722516	0.189673
C	-1.403299	3.921559	-1.781493
C	-3.648933	2.796280	-2.002026
H	-2.999337	1.850365	0.508137
H	-1.436502	2.665519	0.676116
H	-2.927588	3.629544	0.541744
H	-1.260393	3.904178	-2.868404
H	-1.874817	4.875333	-1.508734
H	-0.417072	3.874479	-1.308151
H	-4.188910	3.705707	-1.707316
H	-3.549759	2.789758	-3.093755
H	-4.248283	1.925632	-1.710944
Rh	-0.455601	0.234746	-0.898647
H	1.147285	0.088454	-0.912303
C	1.268268	1.231155	3.201775
H	0.782575	0.624128	3.965863

INT2E-IMes

G_{sol}(toluene) = -1668.449823 Hartree

C	-0.349260	-1.458157	-0.018414
C	0.269240	-3.606375	0.444906
C	-1.068700	-3.527981	0.642565
H	0.962048	-4.426909	0.547372
H	-1.793189	-4.263845	0.955054
N	0.694418	-2.340515	0.040275
N	-1.429660	-2.216491	0.354833
C	2.066648	-2.046101	-0.269722
C	2.487375	-2.103666	-1.603561
C	2.940344	-1.726352	0.782081
C	3.828559	-1.810243	-1.876531
C	4.266992	-1.438247	0.459170
C	4.727472	-1.468063	-0.862931
H	4.172478	-1.838337	-2.908126
H	4.954358	-1.165782	1.256852
C	-2.766527	-1.688113	0.408748
C	-3.562826	-1.739711	-0.747570

C	-3.220282	-1.116395	1.608851	H	6.407035	-1.289204	-2.223331
C	-4.848772	-1.195922	-0.676386	C	2.224354	1.232720	-1.133940
C	-4.512982	-0.585398	1.628203	C	1.205229	1.043438	-0.215788
C	-5.337150	-0.609585	0.497068	C	1.431221	1.450215	1.135453
H	-5.480417	-1.224183	-1.561456	C	2.678285	2.028384	1.534170
H	-4.880630	-0.133318	2.546506	C	3.695373	2.198301	0.559054
C	-3.034451	-2.337051	-2.028865	C	3.460538	1.806269	-0.742823
H	-2.245725	-1.704549	-2.453971	H	2.087078	0.936712	-2.168971
H	-2.597914	-3.329479	-1.866998	C	2.811591	2.400655	2.896599
H	-3.830308	-2.430229	-2.773875	H	4.645668	2.639043	0.850240
C	-2.323281	-1.051866	2.819462	H	4.237040	1.936234	-1.493305
H	-1.941161	-2.042230	3.095351	C	1.758562	2.209532	3.763844
H	-1.454603	-0.414173	2.617528	H	3.745970	2.839331	3.240716
H	-2.858688	-0.640265	3.680515	H	1.830308	2.488864	4.810840
C	-6.711338	0.014668	0.530350	N	0.398037	1.265346	2.013314
H	-6.666890	1.063661	0.208639	O	-1.535338	2.239648	-0.760378
H	-7.405847	-0.502717	-0.140857	C	-1.299210	3.327872	-1.618925
H	-7.136748	0.000606	1.539743	C	-0.223104	4.245727	-1.004180
C	2.455656	-1.688727	2.209700	C	-0.844240	2.863205	-3.016021
H	1.543341	-1.093117	2.304365	C	-2.628298	4.096586	-1.742344
H	2.224328	-2.695681	2.580980	H	-0.531762	4.558870	0.000039
H	3.214195	-1.254170	2.867208	H	0.724697	3.705884	-0.914734
C	1.518535	-2.424560	-2.713418	H	-0.056185	5.144907	-1.612865
H	0.915593	-3.311222	-2.485222	H	-1.581081	2.169091	-3.437980
H	0.818285	-1.594192	-2.866690	H	-0.728009	3.710064	-3.705495
H	2.045806	-2.603322	-3.655642	H	0.114851	2.339591	-2.944922
C	6.156538	-1.095932	-1.175086	H	-2.527923	4.989108	-2.373617
H	6.327675	-0.028689	-0.981778	H	-3.398178	3.447775	-2.177239
H	6.864078	-1.653041	-0.548801	H	-2.971426	4.410852	-0.749802

Rh	-0.650904	0.483266	-0.494688
H	-0.408869	0.140774	-1.966208
C	0.560969	1.637214	3.269196
H	-0.283825	1.476706	3.937492

TS3E-IMes

G_{sol}(toluene) = -1668.418718 Hartree

C	-0.406358	-1.416346	-0.199533
C	0.187506	-3.620879	-0.111811
C	-1.146406	-3.562256	0.116989
H	0.868766	-4.455821	-0.162156
H	-1.876277	-4.332852	0.310928
N	0.627696	-2.310457	-0.301991
N	-1.492488	-2.216960	0.064851
C	1.999661	-1.990990	-0.585074
C	2.387728	-1.783910	-1.914631
C	2.906268	-1.911225	0.483515
C	3.729947	-1.476577	-2.161577
C	4.233678	-1.595720	0.187310
C	4.662354	-1.369279	-1.125828
H	4.047700	-1.298945	-3.186717
H	4.946892	-1.505690	1.003661
C	-2.817224	-1.683101	0.232531
C	-3.646741	-1.566009	-0.895021
C	-3.225524	-1.266283	1.512313
C	-4.918892	-1.012254	-0.714112
C	-4.505176	-0.720455	1.639748
C	-5.363407	-0.583997	0.541049
H	-5.572827	-0.906679	-1.576700
H	-4.837602	-0.385273	2.619849
C	-3.167242	-1.993551	-2.261063
H	-2.349296	-1.349229	-2.605367
H	-2.784726	-3.020860	-2.255658
H	-3.976601	-1.934099	-2.994933
C	-2.291460	-1.368655	2.691552
H	-1.866063	-2.375110	2.784934
H	-1.451492	-0.672463	2.574771
H	-2.812698	-1.129141	3.623742
C	-6.748206	-0.009223	0.719771
H	-6.740749	0.841751	1.410273
H	-7.169251	0.330403	-0.232222
H	-7.434952	-0.758620	1.136558
C	2.457479	-2.147242	1.904150
H	1.541416	-1.593636	2.128194
H	2.248561	-3.209386	2.088456
H	3.227655	-1.830052	2.613399
C	1.377612	-1.827355	-3.032085
H	0.769777	-2.739415	-2.998670
H	0.684882	-0.980353	-2.948863
H	1.868597	-1.778312	-4.009076
C	6.089535	-0.961206	-1.398497
H	6.248734	0.087297	-1.112473
H	6.800080	-1.563926	-0.820449
H	6.345306	-1.057985	-2.458965
C	2.114450	1.362723	-0.911415
C	1.137168	1.032000	0.020849
C	1.481593	1.165803	1.410957

C	2.778376	1.649314	1.798671
C	3.724753	1.992858	0.799681
C	3.389289	1.843858	-0.528568
H	1.907620	1.239385	-1.969638
C	3.054108	1.755193	3.186439
H	4.703784	2.361203	1.097161
H	4.112867	2.091025	-1.302455
C	2.093094	1.403716	4.106200
H	4.029472	2.116624	3.506444
H	2.274724	1.474899	5.174673
N	0.545446	0.819256	2.348852
O	-1.584122	2.298344	-1.195099
C	-1.143399	3.650377	-1.365179
C	-0.479440	4.154419	-0.076421
C	-0.172180	3.755153	-2.549971
C	-2.417701	4.457819	-1.655889
H	-1.156701	4.011951	0.773410
H	0.438928	3.596789	0.125437
H	-0.229808	5.221065	-0.148708
H	-0.636221	3.349815	-3.457462
H	0.105082	4.798943	-2.743185
H	0.743350	3.192766	-2.344822
H	-2.191315	5.521864	-1.799903
H	-2.906462	4.080327	-2.561279
H	-3.123248	4.363077	-0.822758
Rh	-0.718597	0.533876	-0.381674
H	-0.819584	1.330650	-1.800454
C	0.844592	0.938029	3.628345
H	0.067942	0.645464	4.334785

INT3E-IMes

G_{sol}(toluene) = -1434.983291 Hartree

C	-0.270788	-1.298270	-0.006682
C	-1.090435	-3.449247	0.046295
C	0.260287	-3.528578	0.082588
H	-1.854095	-4.211844	0.054673
H	0.924808	-4.377030	0.131761
N	-1.399724	-2.095159	-0.011336
N	0.750699	-2.219224	0.048652
C	-2.716880	-1.524483	-0.025390
C	-3.340262	-1.220525	1.197547
C	-3.334548	-1.265426	-1.263454
C	-4.616664	-0.646459	1.156297
C	-4.608894	-0.693361	-1.250999
C	-5.263056	-0.374206	-0.053695
H	-5.115032	-0.408167	2.093296
H	-5.102160	-0.487677	-2.198620
C	2.155618	-1.933703	0.102056
C	2.731157	-1.548905	1.323208
C	2.918035	-2.085804	-1.066352
C	4.110033	-1.319505	1.351346
C	4.295656	-1.853178	-0.986473
C	4.909057	-1.472823	0.212456
H	4.570173	-1.007706	2.286460
H	4.899009	-1.956925	-1.885624
C	1.881600	-1.344943	2.550551
H	1.242582	-0.462320	2.421945

H	1.217012	-2.198403	2.730055
H	2.503575	-1.199510	3.439395
C	2.260311	-2.437195	-2.378002
H	1.757396	-3.410848	-2.337817
H	1.496306	-1.694229	-2.636543
H	2.994901	-2.469246	-3.188414
C	6.400148	-1.244226	0.283563
H	6.645802	-0.413473	0.954755
H	6.918639	-2.134069	0.666164
H	6.820588	-1.019643	-0.702603
C	-2.625924	-1.580011	-2.557006
H	-1.736126	-0.948352	-2.665850
H	-2.286319	-2.621946	-2.589925
H	-3.280539	-1.404708	-3.416171
C	-2.648261	-1.495638	2.510566
H	-2.303212	-2.534050	2.576551
H	-1.763433	-0.857233	2.621773
H	-3.318136	-1.302646	3.354088
C	-6.627999	0.271401	-0.078456
H	-6.551401	1.340190	-0.318905
H	-7.272716	-0.184152	-0.839126
H	-7.132530	0.187226	0.889794
C	2.521715	1.439703	-0.475648
C	1.159062	1.542860	-0.222654
C	0.651641	2.868638	-0.101372
C	1.418209	4.056950	-0.198634
C	2.808280	3.892243	-0.443104
C	3.320605	2.613155	-0.577612
H	3.003569	0.479002	-0.599577
C	0.704914	5.274699	-0.043812
H	3.450748	4.765383	-0.525760
H	4.385311	2.491236	-0.770136
C	-0.658180	5.243292	0.180315
H	1.233741	6.223578	-0.104265
H	-1.223916	6.162326	0.300544
N	-0.702586	2.850542	0.118581
Rh	-0.565463	0.635636	0.035814
C	-1.338759	4.002833	0.254129
H	-2.411476	3.962781	0.425745

TS4A-IMes

G_{sol}(toluene) = -4963.273778 Hartree

C	3.566933	-1.721565	-0.255594
C	2.317489	-1.196324	1.695642
C	3.692260	-1.714280	-1.662498
C	4.598330	-2.323892	0.529430
C	3.313618	-1.814032	2.508864
H	1.019376	-0.473422	2.381955
C	4.802654	-2.268324	-2.267030
H	2.900106	-1.271032	-2.248388
C	5.733292	-2.871078	-0.121020
C	4.440475	-2.352398	1.941245
H	3.169464	-1.839747	3.586239
C	5.838264	-2.842828	-1.495513
H	4.883156	-2.256238	-3.350857
H	6.515712	-3.319646	0.486634
H	5.220642	-2.809957	2.548118

H	6.708668	-3.268824	-1.986953	H	-2.560061	-4.742448	0.989504
N	2.477616	-1.149672	0.378012	H	-2.954709	-4.544711	-0.719239
O	0.141093	0.063235	2.580342	C	-5.265654	-4.000866	0.617492
C	-0.719223	-0.685953	3.460328	H	-5.108542	-4.918750	1.202730
C	-0.914939	-2.112631	2.924223	H	-6.117749	-3.475627	1.062064
C	-0.058598	-0.717901	4.847393	H	-5.558302	-4.311196	-0.394664
C	-2.061182	0.046552	3.517582	C	-3.210008	-2.462936	-2.967538
H	-1.377240	-2.075386	1.935633	H	-3.057447	-3.481423	-2.595754
H	0.044480	-2.636161	2.840694	H	-3.367127	-2.532622	-4.054343
H	-1.563216	-2.695071	3.590371	H	-2.278815	-1.906771	-2.803294
H	0.140087	0.301897	5.195785	C	-4.660961	-0.407773	-2.980729
H	-0.703385	-1.217195	5.581590	H	-3.800562	0.259460	-2.853771
H	0.895570	-1.258175	4.809899	H	-4.815669	-0.542438	-4.061285
H	-2.797611	-0.538788	4.081226	H	-5.543192	0.109315	-2.585803
H	-1.955319	1.021000	4.005565	C	-7.010740	-0.924837	-0.208821
H	-2.433868	0.201222	2.502633	H	-7.296622	-1.882547	0.241207
Rh	0.538227	-0.277905	-0.046541	H	-7.778487	-0.193266	0.083408
Br	0.473527	-0.571253	-2.494263	H	-7.072771	-1.045076	-1.296501
C	-1.420211	-0.220169	-0.074760	C	-5.581140	-0.297902	1.785872
C	-2.586081	-0.611755	-0.165333	H	-4.638338	0.145158	2.122600
Si	-4.158126	-1.510749	-0.405237	H	-6.397373	0.352358	2.134283
C	-4.401708	-1.761611	-2.289635	H	-5.700144	-1.260215	2.301297
H	-5.296073	-2.391475	-2.420525	C	0.807938	1.663639	-0.247696
C	-5.617865	-0.462477	0.257640	C	1.870459	3.642556	-0.592641
H	-5.434976	0.526511	-0.181923	C	0.543295	3.886125	-0.620038
C	-3.991845	-3.136712	0.599566	H	2.725318	4.290781	-0.700832
H	-3.818230	-2.774261	1.625085	H	-0.018398	4.796384	-0.755329
C	-2.772668	-3.994839	0.211777	N	2.027602	2.280747	-0.362423
H	-1.868189	-3.393970	0.067706	N	-0.103404	2.673146	-0.404348

C	3.356484	1.749114	-0.166095
C	3.847501	1.664295	1.147401
C	4.148105	1.473302	-1.287123
C	5.154587	1.205176	1.316663
C	5.458958	1.035934	-1.062283
C	5.972028	0.878777	0.226487
H	5.546250	1.102020	2.325822
H	6.079720	0.786916	-1.918726
C	-1.537521	2.693063	-0.213909
C	-2.009898	2.947002	1.083956
C	-2.382200	2.626239	-1.327650
C	-3.389779	3.068714	1.256819
C	-3.755038	2.771700	-1.102744
C	-4.276600	2.988421	0.176177
H	-3.780067	3.248896	2.255966
H	-4.431684	2.712690	-1.951453
C	-1.053711	3.101655	2.237745
H	-0.477514	2.184086	2.393018
H	-0.342463	3.918271	2.056442
H	-1.593741	3.330903	3.161112
C	-1.847349	2.376182	-2.712097
H	-0.988600	3.017588	-2.943055
H	-1.503753	1.341040	-2.807160
H	-2.618862	2.555502	-3.467034
C	-5.759030	3.180004	0.391597
H	-6.111602	2.613292	1.260215
H	-5.994519	4.236814	0.577020
H	-6.339811	2.860441	-0.479693
C	3.616579	1.647879	-2.687236
H	2.632672	1.184524	-2.805579
H	3.506497	2.710645	-2.940869
H	4.296092	1.200395	-3.418675
C	3.003158	2.073313	2.327462
H	3.559250	1.948786	3.261629
H	2.705180	3.127136	2.250932
H	2.085669	1.483551	2.403793
C	7.381513	0.386920	0.450447
H	7.426690	-0.314094	1.291407
H	7.774372	-0.122461	-0.434887
H	8.058901	1.219182	0.685513

TS4B-IMes

G_{sol}(toluene) = -4963.301246 Hartree

C	-2.139032	2.633760	-0.074062
C	0.028870	3.241121	-0.717026
C	-2.913062	1.554205	0.431882
C	-2.649144	3.921977	-0.403873
C	-0.395408	4.554523	-1.020877
H	1.050599	2.913746	-0.864817
C	-4.268423	1.824780	0.567390
H	-2.130133	0.716685	1.482864
C	-4.042498	4.126448	-0.227773
C	-1.726024	4.889796	-0.876726
H	0.331077	5.272638	-1.387087
C	-4.827517	3.091523	0.240686
H	-4.933813	1.061696	0.958439
H	-4.473847	5.096964	-0.461585

H	-2.077045	5.887399	-1.131532	H	2.226042	3.288873	1.342808
H	-5.895424	3.251593	0.376657	H	3.323005	4.656891	1.593337
N	-0.812698	2.338499	-0.252211	H	2.930187	4.128056	-0.044168
O	-1.267596	0.184664	1.956182	C	5.603400	3.559379	0.513647
C	-0.697912	0.856315	3.116616	H	5.799841	4.443762	1.136972
C	0.477185	0.003305	3.592490	H	6.503127	2.935179	0.541090
C	-0.219061	2.271479	2.787610	H	5.482692	3.913553	-0.517934
C	-1.820210	0.893678	4.161851	C	3.661450	2.277361	-2.522551
H	0.133747	-0.977705	3.932984	H	3.645537	3.281122	-2.080240
H	1.193375	-0.134103	2.777088	H	3.973376	2.388062	-3.571399
H	0.989858	0.493648	4.428645	H	2.633641	1.893506	-2.525342
H	-1.040489	2.899433	2.431599	C	4.641906	-0.046103	-2.472859
H	0.202261	2.737782	3.685854	H	3.660015	-0.531532	-2.441900
H	0.555311	2.235789	2.020056	H	4.922504	0.066802	-3.530481
H	-1.461434	1.341579	5.096036	H	5.365516	-0.728795	-2.011617
H	-2.665997	1.488877	3.798401	C	6.697691	0.056318	0.802467
H	-2.179018	-0.118132	4.378145	H	7.015369	0.913318	1.409851
Rh	-0.747739	0.222118	-0.134621	H	7.261733	-0.815767	1.164949
Br	-0.592437	0.323716	-2.622971	H	7.017907	0.245337	-0.229823
C	1.181508	0.242125	0.005442	C	4.772504	-0.446251	2.370667
C	2.359537	0.583911	0.119705	H	3.708711	-0.695247	2.451874
Si	4.098030	1.143641	0.076151	H	5.344177	-1.281639	2.801703
C	4.597802	1.318895	-1.764319	H	4.962824	0.428813	3.006859
H	5.612408	1.745760	-1.778177	C	-1.020309	-1.807467	-0.287567
C	5.183197	-0.187341	0.912048	C	-2.123471	-3.759171	-0.713260
H	4.946255	-1.092124	0.341196	C	-0.802153	-4.031041	-0.733939
C	4.342146	2.816467	0.998412	H	-2.990418	-4.383954	-0.858421
H	4.507529	2.531884	2.049198	H	-0.258419	-4.948073	-0.896069
C	3.135735	3.768748	0.971781	N	-2.249294	-2.401518	-0.433966

N	-0.137257	-2.835642	-0.469139
C	-3.570526	-1.858350	-0.207674
C	-4.078142	-1.893126	1.102058
C	-4.360548	-1.493777	-1.308287
C	-5.407558	-1.504916	1.296006
C	-5.681703	-1.107126	-1.058823
C	-6.221702	-1.103687	0.230726
H	-5.817250	-1.524296	2.303470
H	-6.305950	-0.811836	-1.899004
C	1.286161	-2.896814	-0.227320
C	1.703779	-3.154092	1.087583
C	2.171665	-2.906134	-1.312140
C	3.060496	-3.400493	1.306765
C	3.518950	-3.170473	-1.042950
C	3.980184	-3.426837	0.251973
H	3.403689	-3.593017	2.320581
H	4.223621	-3.178032	-1.870586
C	0.703943	-3.217906	2.213654
H	0.016061	-2.370362	2.184304
H	0.097537	-4.131633	2.147858
H	1.207230	-3.219366	3.184800
C	1.693620	-2.655841	-2.717564
H	0.916783	-3.372385	-3.013487
H	1.250216	-1.659029	-2.806647
H	2.518922	-2.737917	-3.431212
C	5.432817	-3.750928	0.509283
H	5.780283	-3.307662	1.448771
H	5.586281	-4.836111	0.585660
H	6.077191	-3.384541	-0.296629
C	-3.825539	-1.551154	-2.715119
H	-4.591797	-1.247288	-3.434753
H	-2.956593	-0.897391	-2.836591
H	-3.498132	-2.565304	-2.977472
C	-3.228558	-2.363842	2.255149
H	-2.846839	-3.377943	2.082512
H	-2.366887	-1.703297	2.388858
H	-3.806384	-2.374082	3.184619
C	-7.635425	-0.633823	0.472647
H	-8.289307	-0.867166	-0.374934
H	-8.065238	-1.088391	1.372015
H	-7.663093	0.455827	0.612450

TS4C-IMes

G_{sol}(toluene) = -4963.283417 Hartree

C	-3.829480	2.481065	-0.061082
C	-1.741054	1.731418	0.617169
C	-5.199930	2.202458	-0.313259
C	-3.320621	3.798263	-0.317092
C	-1.151324	3.002408	0.377263
H	-1.343118	0.889268	1.609753
C	-6.031875	3.189454	-0.791385
H	-5.552351	1.193601	-0.124983
C	-4.207300	4.792663	-0.806701
C	-1.940158	4.030728	-0.078693
H	-0.086336	3.122670	0.532211
C	-5.532983	4.494990	-1.036994
H	-7.078928	2.972654	-0.985943

H	-3.821436	5.790845	-1.000406	C	2.343578	4.169535	1.463752
H	-1.523545	5.015810	-0.279346	H	1.629355	3.484975	1.928549
H	-6.204228	5.261907	-1.414115	H	2.448068	5.039912	2.128394
N	-3.029948	1.484610	0.406949	H	1.898242	4.530097	0.528175
O	-1.115333	-0.209156	2.245347	C	4.720269	4.545986	0.714672
C	-0.262607	-0.017014	3.395208	H	4.837623	5.359708	1.444833
C	0.315422	1.403517	3.419427	H	5.715300	4.124582	0.533901
C	-1.185442	-0.232784	4.604694	H	4.383099	5.002979	-0.224190
C	0.863731	-1.051648	3.385927	C	2.659396	3.279851	-2.238435
H	0.960911	1.559906	2.553886	H	2.514032	4.200282	-1.660233
H	-0.486198	2.151504	3.404981	H	2.784396	3.572107	-3.291404
H	0.909032	1.561570	4.327696	H	1.736264	2.691343	-2.165931
H	-1.623892	-1.235879	4.577092	C	4.103137	1.252864	-2.660590
H	-0.629621	-0.120948	5.543909	H	3.257889	0.558141	-2.605595
H	-2.002036	0.497573	4.594493	H	4.206471	1.564740	-3.710366
H	1.537833	-0.899129	4.237899	H	5.009663	0.697448	-2.391628
H	0.456772	-2.064546	3.457216	C	6.495367	1.348651	0.425588
H	1.444553	-0.968439	2.463709	H	6.689489	2.151810	1.147819
Rh	-0.765936	-0.229240	0.120568	H	7.262684	0.578542	0.592939
Br	-0.855289	0.411777	-2.289014	H	6.659513	1.759519	-0.578600
C	1.029666	0.416201	0.176602	C	4.887307	0.199086	2.012180
C	2.104534	1.012561	0.217148	H	3.909166	-0.281688	2.123224
Si	3.671329	1.946000	0.085849	H	5.656266	-0.550932	2.250052
C	3.870937	2.466526	-1.745182	H	4.963457	0.984776	2.776344
H	4.761926	3.110721	-1.797055	C	-0.443644	-2.196815	-0.397190
C	5.083124	0.764366	0.595538	C	-1.230453	-4.249738	-0.998348
H	4.976293	-0.071862	-0.105248	C	0.116449	-4.275397	-1.121056
C	3.706306	3.499435	1.221619	H	-1.984562	-5.000872	-1.173191
H	4.068277	3.131908	2.194453	H	0.795327	-5.055569	-1.426856

N	-1.559090	-2.974995	-0.555994
N	0.587089	-3.014956	-0.748600
C	-2.896524	-2.558648	-0.204689
C	-3.272492	-2.618304	1.151100
C	-3.783381	-2.154503	-1.215070
C	-4.550289	-2.172769	1.489522
C	-5.053468	-1.718824	-0.819701
C	-5.445735	-1.701266	0.521710
H	-4.854275	-2.193743	2.533512
H	-5.750268	-1.384355	-1.584386
C	2.008673	-2.780793	-0.654668
C	2.667412	-3.190023	0.515851
C	2.701298	-2.312043	-1.779714
C	4.058273	-3.060916	0.561612
C	4.094156	-2.222270	-1.690813
C	4.788352	-2.583840	-0.531817
H	4.583077	-3.355740	1.467648
H	4.648080	-1.856147	-2.551063
C	1.923884	-3.827326	1.663865
H	0.905527	-3.447931	1.759455
H	1.855730	-4.914729	1.521522
H	2.442512	-3.651590	2.611051
C	1.978598	-1.940558	-3.046868
H	1.343662	-2.762238	-3.401770
H	1.320508	-1.080290	-2.889486
H	2.688205	-1.692848	-3.841863
C	6.293256	-2.480026	-0.460399
H	6.762358	-3.471035	-0.524608
H	6.694177	-1.870285	-1.276443
H	6.614612	-2.030545	0.485932
C	-3.401844	-2.218357	-2.671075
H	-2.513578	-1.612404	-2.873681
H	-3.177651	-3.247493	-2.979318
H	-4.216887	-1.848726	-3.300561
C	-2.328831	-3.160334	2.193749
H	-1.932100	-4.141153	1.904037
H	-1.487524	-2.477692	2.332814
H	-2.837266	-3.269616	3.156311
C	-6.804636	-1.188061	0.932780
H	-6.710420	-0.263598	1.516780
H	-7.436859	-0.973518	0.064927
H	-7.331637	-1.913710	1.564596

TS4D-IMes

G_{sol}(toluene) = -4963.281220 Hartree

C	-1.676547	3.169377	-0.238622
C	0.051722	4.572857	-0.830288
C	-2.116368	1.946122	0.360475
C	-2.636780	4.047765	-0.845321
C	-0.813963	5.493058	-1.470353
H	1.121295	4.770580	-0.807291
C	-3.482868	1.675969	0.368782
H	-1.612656	1.229209	1.457452
C	-4.016421	3.709570	-0.813399
C	-2.162930	5.229856	-1.468094
H	-0.407146	6.383097	-1.941088
C	-4.436843	2.547706	-0.206733

H	-3.839158	0.759107	0.833947	H	3.324010	2.055763	2.552174
H	-4.731579	4.386744	-1.275893	C	2.739161	3.785358	1.437489
H	-2.872169	5.908157	-1.937617	H	1.762107	3.449775	1.080942
H	-5.493051	2.292364	-0.176114	H	2.583170	4.360969	2.361953
N	-0.345318	3.463481	-0.237895	H	3.148242	4.483359	0.696331
O	-1.382167	0.188947	2.095154	C	5.102739	3.149433	2.053169
C	-0.518741	0.442138	3.232612	H	5.030660	3.908174	2.846463
C	0.517995	-0.676333	3.356795	H	5.771164	2.363173	2.419573
C	0.165464	1.806738	3.090662	H	5.593822	3.629963	1.196064
C	-1.461110	0.439812	4.445456	C	3.305688	3.009457	-1.975955
H	0.032570	-1.641530	3.535071	H	3.348170	3.922333	-1.373363
H	1.113890	-0.741340	2.444314	H	3.581529	3.283098	-3.005248
H	1.191175	-0.480443	4.200326	H	2.264117	2.666655	-1.990785
H	-0.575961	2.612617	3.040787	C	4.300057	0.739240	-2.424602
H	0.817354	1.999425	3.951089	H	3.310927	0.280094	-2.540117
H	0.764790	1.837064	2.181308	H	4.624476	1.081904	-3.418120
H	-0.901721	0.620924	5.371566	H	4.992184	-0.048261	-2.105380
H	-2.219251	1.223925	4.339950	C	6.394462	0.118217	0.569042
H	-1.974253	-0.524090	4.532042	H	6.790265	0.925214	1.196298
Rh	-1.031184	-0.033264	-0.011600	H	6.985298	-0.781229	0.796871
Br	-1.098474	0.394113	-2.452397	H	6.601710	0.387389	-0.473616
C	0.845810	0.293946	0.060532	C	4.645531	-0.597442	2.264378
C	2.030834	0.603809	0.155982	H	3.586241	-0.819270	2.439434
Si	3.723211	1.283549	0.303793	H	5.217137	-1.508484	2.495760
C	4.239907	1.915291	-1.429690	H	4.949002	0.165467	2.993192
H	5.252656	2.337871	-1.332999	C	-0.860928	-2.036032	-0.350071
C	4.897501	-0.136036	0.819417	C	-1.867106	-4.042861	-0.756838
H	4.590215	-0.956212	0.162576	C	-0.539280	-4.202415	-0.950024
C	3.706569	2.614964	1.683446	H	-2.695906	-4.728692	-0.835942

H	0.046820	-5.062042	-1.233653
N	-2.050677	-2.716151	-0.389720
N	0.069115	-2.970519	-0.695999
C	-3.328965	-2.166971	-0.004694
C	-3.654199	-2.140480	1.365795
C	-4.238980	-1.770373	-0.999418
C	-4.890480	-1.604729	1.730156
C	-5.464734	-1.239457	-0.578233
C	-5.800186	-1.132251	0.774297
H	-5.151839	-1.558574	2.784811
H	-6.174327	-0.907072	-1.331779
C	1.514761	-2.916962	-0.682274
C	2.166243	-3.373205	0.472508
C	2.206742	-2.618212	-1.866371
C	3.557455	-3.513233	0.427499
C	3.594339	-2.777413	-1.860654
C	4.286002	-3.229966	-0.730485
H	4.078997	-3.856438	1.317740
H	4.149437	-2.544167	-2.766031
C	1.396579	-3.758830	1.711461
H	0.529248	-3.114781	1.866477
H	1.028210	-4.791546	1.641589
H	2.031650	-3.692815	2.600101
C	1.486776	-2.152170	-3.101585
H	0.668613	-2.830277	-3.374331
H	1.034284	-1.169020	-2.940808
H	2.173023	-2.085572	-3.951319
C	5.786503	-3.391754	-0.771928
H	6.273196	-2.474819	-1.124137
H	6.193972	-3.629494	0.215884
H	6.079740	-4.196618	-1.458673
C	-3.934002	-1.944858	-2.463792
H	-3.787048	-3.003001	-2.714233
H	-4.755522	-1.564343	-3.078010
H	-3.021248	-1.411493	-2.745283
C	-2.702962	-2.687837	2.396709
H	-2.393461	-3.711219	2.151683
H	-1.808401	-2.064627	2.449217
H	-3.167813	-2.699592	3.386860
C	-7.107454	-0.516084	1.209820
H	-7.652531	-1.177454	1.894442
H	-6.934132	0.426054	1.745916
H	-7.757787	-0.300644	0.356116

INT4A-IMes

G_{sol}(toluene) = -4729.872260 Hartree

C	-3.481533	0.186305	-1.324983
C	-1.525381	-1.003511	-1.065595
C	-4.159504	1.428664	-1.250880
C	-4.203419	-0.976723	-1.735843
C	-2.148508	-2.225203	-1.488798
C	-5.498490	1.510121	-1.571988
H	-3.595947	2.299961	-0.934839
C	-5.580575	-0.863292	-2.054624
C	-3.481953	-2.198868	-1.808605
H	-1.576608	-3.144681	-1.575011
C	-6.217139	0.358125	-1.975801

H	-6.011304	2.466652	-1.514428	H	3.770764	2.248842	-1.812907
H	-6.123123	-1.752426	-2.368359	H	4.459962	3.635841	-2.670273
H	-3.991335	-3.103318	-2.134620	H	4.573443	3.544194	-0.906626
H	-7.271417	0.441335	-2.225673	C	3.363856	5.132509	1.592835
N	-2.154078	0.130169	-0.989077	H	2.590836	5.878135	1.818044
Rh	0.400773	-1.229876	-0.695422	H	4.088142	5.164062	2.419878
Br	1.016568	-1.538848	-3.093762	H	3.887972	5.462603	0.687598
C	0.850444	0.599705	-0.438906	C	2.080132	3.285006	2.751192
C	1.206806	1.761793	-0.249488	H	1.665065	2.274312	2.677861
Si	1.620173	3.531863	-0.087209	H	2.793053	3.292863	3.588783
C	2.568223	4.068240	-1.656539	H	1.258048	3.959989	3.023674
H	2.768038	5.146652	-1.558590	C	0.295454	-1.391195	1.321318
C	2.760126	3.724626	1.442496	C	1.208057	-1.899244	3.341314
H	3.587689	3.024658	1.251708	C	-0.060486	-1.473042	3.557940
C	-0.010771	4.514309	0.153734	H	1.978298	-2.237280	4.016509
H	-0.227300	4.436734	1.230831	H	-0.634486	-1.361829	4.464341
C	-1.207140	3.900623	-0.598731	N	1.410367	-1.843063	1.968858
H	-1.331347	2.835447	-0.380905	N	-0.607331	-1.163670	2.313638
H	-2.139562	4.421478	-0.332045	C	2.619120	-2.233443	1.284066
H	-1.087209	3.988929	-1.685450	C	2.664523	-3.481593	0.639390
C	0.137861	6.009243	-0.187484	C	3.704040	-1.337484	1.266001
H	-0.790237	6.556215	0.033533	C	3.835641	-3.815450	-0.049730
H	0.943759	6.493605	0.376644	C	4.848232	-1.721388	0.564286
H	0.350615	6.155777	-1.254090	C	4.929768	-2.947807	-0.107590
C	1.737752	3.846088	-2.933982	H	3.886246	-4.774292	-0.560228
H	0.810325	4.430573	-2.933270	H	5.694565	-1.039244	0.531694
H	2.307829	4.137656	-3.828107	C	-1.983763	-0.756908	2.167710
H	1.462950	2.789808	-3.046386	C	-2.948791	-1.753302	1.954432
C	3.918495	3.335493	-1.761652	C	-2.312510	0.597656	2.296479

C	-4.281857	-1.352830	1.847977
C	-3.664003	0.945483	2.195069
C	-4.658203	-0.009331	1.967769
H	-5.044643	-2.105983	1.664368
H	-3.940803	1.993493	2.279123
C	-2.556075	-3.206062	1.850626
H	-1.832232	-3.368439	1.045327
H	-2.090778	-3.562846	2.778493
H	-3.428906	-3.833175	1.646985
C	-1.246639	1.639255	2.510850
H	-0.669934	1.446659	3.424020
H	-0.541125	1.640753	1.674745
H	-1.685681	2.637789	2.592697
C	-6.109557	0.385142	1.843154
H	-6.231689	1.472644	1.832230
H	-6.542447	-0.010199	0.917849
H	-6.700776	-0.011703	2.679126
C	3.617024	0.004949	1.944481
H	2.841746	0.615076	1.469450
H	3.361421	-0.083220	3.006789
H	4.566980	0.541692	1.867927
C	1.490737	-4.432719	0.647099
H	0.905742	-4.365374	1.569363
H	0.812552	-4.225093	-0.192534
H	1.830914	-5.466585	0.530644
C	6.164903	-3.306252	-0.897109
H	6.146573	-2.820471	-1.881540
H	7.077252	-2.974544	-0.388483
H	6.238922	-4.385877	-1.064550

INT4B-IMes

G_{sol}(toluene) = -4729.856230 Hartree

C	0.664467	-1.775268	1.047792
C	-0.116647	-0.821695	2.998660
C	0.902753	-1.448295	-0.299153
C	0.799384	-3.068397	1.594847
C	-0.005011	-2.076382	3.650489
H	-0.488250	0.052978	3.521837
C	1.293725	-2.441431	-1.162961
C	1.236444	-4.078970	0.693010
C	0.452736	-3.187527	2.965101
H	-0.294301	-2.148743	4.693990
C	1.468633	-3.757012	-0.632645
H	1.462596	-2.251068	-2.216663
H	1.368698	-5.097846	1.046589
H	0.530986	-4.149341	3.466225
H	1.790491	-4.540760	-1.314274
N	0.230144	-0.676338	1.732017
Rh	0.346240	0.441976	-0.215875
Br	0.557271	0.953001	-2.628289
C	-1.548242	-0.126884	-0.477448
C	-2.732400	-0.470002	-0.486642
Si	-4.456457	-1.042224	-0.384295
C	-4.696952	-2.458184	-1.649133
H	-5.709599	-2.861794	-1.493002
C	-5.603621	0.430353	-0.810628
H	-5.362739	0.684096	-1.853826

C	-4.834034	-1.655327	1.401802	H	5.424047	1.975842	0.597718
H	-5.203002	-0.764250	1.934719	H	3.734459	4.154738	1.026866
C	-3.586761	-2.151434	2.156112	N	3.536109	0.992923	0.201457
H	-2.811851	-1.380167	2.200660	N	2.237153	2.670403	0.531683
H	-3.839138	-2.444302	3.186739	C	4.031424	-0.334359	-0.052983
H	-3.136396	-3.026044	1.669507	C	4.111594	-1.234827	1.021338
C	-5.952759	-2.714172	1.445882	C	4.415270	-0.678721	-1.354531
H	-6.223438	-2.957316	2.483947	C	4.557692	-2.529412	0.752232
H	-6.867011	-2.382670	0.939513	C	4.857403	-1.989362	-1.573117
H	-5.636746	-3.650264	0.968932	C	4.922563	-2.927880	-0.539735
C	-3.685217	-3.596409	-1.415447	H	4.610417	-3.248058	1.566884
H	-3.773241	-4.033504	-0.413524	H	5.146915	-2.282503	-2.579633
H	-3.830774	-4.409234	-2.142246	C	1.011436	3.413619	0.677321
H	-2.655433	-3.233341	-1.525222	C	0.296272	3.296320	1.883062
C	-4.600762	-1.949187	-3.098602	C	0.547455	4.192925	-0.395296
H	-3.624696	-1.484239	-3.288703	C	-0.960550	3.902708	1.958316
H	-4.716047	-2.775943	-3.814994	C	-0.713777	4.784490	-0.266548
H	-5.373664	-1.206191	-3.330442	C	-1.494044	4.624301	0.883758
C	-7.098975	0.074254	-0.739047	H	-1.536747	3.806368	2.875776
H	-7.404690	-0.159995	0.289109	H	-1.100868	5.373210	-1.094772
H	-7.723819	0.914601	-1.075141	C	0.891717	2.583671	3.072296
H	-7.352170	-0.791267	-1.364587	H	1.271657	1.594002	2.807918
C	-5.292642	1.671423	0.045773	H	1.736681	3.152061	3.482843
H	-4.238194	1.960346	-0.036961	H	0.151715	2.469262	3.870163
H	-5.905665	2.530311	-0.265405	C	1.382432	4.389585	-1.633749
H	-5.506335	1.493056	1.108710	H	2.357672	4.830106	-1.392361
C	2.223284	1.347975	0.190311	H	1.562491	3.434100	-2.136208
C	4.351067	2.072859	0.541827	H	0.876161	5.053486	-2.340721
C	3.529772	3.132148	0.750493	C	-2.887994	5.197558	0.958193

H	-3.624732	4.456976	0.618757
H	-3.155819	5.477936	1.983031
H	-2.996053	6.081790	0.320750
C	4.330387	0.319423	-2.480342
H	3.290303	0.617812	-2.656767
H	4.894782	1.232507	-2.253800
H	4.728455	-0.103291	-3.407934
C	3.729042	-0.813396	2.418144
H	4.372018	-0.001441	2.781058
H	2.699892	-0.445115	2.455050
H	3.814317	-1.650588	3.116918
C	5.340370	-4.353828	-0.802994
H	4.473487	-5.025403	-0.743328
H	5.783344	-4.470530	-1.797401
H	6.069521	-4.703596	-0.062306

TS5B-IMes

G_{sol}(toluene) = -4729.847984 Hartree

C	0.721454	-2.295778	0.281511
C	1.336055	-1.664568	2.414096
C	0.204936	-1.751048	-0.921588
C	1.061171	-3.652537	0.460748
C	1.645877	-3.013021	2.721538
H	1.458197	-0.875952	3.148360
C	0.101316	-2.585910	-2.021900
C	0.899662	-4.495272	-0.672774
C	1.530500	-3.995891	1.756472
H	1.988940	-3.255364	3.722435
C	0.450912	-3.954347	-1.868857
H	-0.269715	-2.215979	-2.971547
H	1.135936	-5.553041	-0.597571
H	1.792314	-5.026465	1.983497
H	0.349138	-4.606499	-2.733031
N	0.897041	-1.318277	1.215874
Rh	0.565328	0.194763	-0.357828
Br	0.262165	1.403497	-2.512566
C	-1.223161	-0.616663	-0.503189
C	-2.428337	-0.855109	-0.371807
Si	-4.166926	-1.231198	0.021063
C	-4.755844	-2.614716	-1.161019
H	-5.762266	-2.915468	-0.830600
C	-5.195445	0.358494	-0.245094
H	-5.094657	0.586517	-1.316856
C	-4.277303	-1.792073	1.857772
H	-4.463870	-0.867382	2.426273
C	-2.969699	-2.402873	2.395065
H	-2.128110	-1.709285	2.293382
H	-3.068666	-2.667210	3.458519
H	-2.697750	-3.319333	1.855541
C	-5.465909	-2.739198	2.112932
H	-5.570055	-2.957736	3.185611
H	-6.419184	-2.318671	1.771343
H	-5.330133	-3.699685	1.600427
C	-3.837581	-3.849574	-1.084623
H	-3.787172	-4.270182	-0.073286
H	-4.192887	-4.646649	-1.754097
H	-2.812280	-3.599084	-1.385568

C	-4.856267	-2.111615	-2.612295	C	0.122743	4.470415	-0.287849
H	-3.887864	-1.736369	-2.968550	C	-1.654651	3.694176	1.738781
H	-5.164022	-2.921252	-3.290081	C	-1.157071	5.020184	-0.202531
H	-5.585375	-1.299748	-2.720410	C	-2.065226	4.634102	0.791889
C	-6.690066	0.158785	0.062939	H	-2.343758	3.390523	2.523340
H	-6.855653	-0.037312	1.130565	H	-1.462135	5.754256	-0.945158
H	-7.269196	1.058474	-0.190545	C	0.013567	2.096199	2.745023
H	-7.123609	-0.678563	-0.498639	H	-0.189939	1.081449	2.382664
C	-4.623830	1.557202	0.533752	H	1.078984	2.150610	2.993973
H	-3.574022	1.741824	0.280685	H	-0.560120	2.244277	3.665968
H	-5.190741	2.473281	0.313550	C	1.065269	4.866885	-1.396253
H	-4.681183	1.398734	1.619678	H	1.981021	5.334616	-1.014864
C	2.007363	1.608481	0.216956	H	1.355507	3.984429	-1.976848
C	3.972753	2.622313	0.746384	H	0.584950	5.577988	-2.075113
C	2.986419	3.537004	0.912658	C	-3.459312	5.212777	0.816915
H	5.040360	2.686531	0.886122	H	-4.036386	4.871236	-0.051565
H	3.012118	4.566440	1.233042	H	-4.005669	4.912639	1.716806
N	3.357054	1.445680	0.324724	H	-3.439159	6.309169	0.780510
N	1.790498	2.901224	0.584162	C	3.958674	0.497767	-2.352293
C	4.014929	0.173908	0.165699	H	2.880671	0.579626	-2.527867
C	4.351191	-0.548623	1.321521	H	4.341331	1.520499	-2.251661
C	4.254880	-0.323480	-1.124974	H	4.416280	0.046887	-3.238503
C	4.874723	-1.834545	1.159387	C	4.172111	0.039606	2.700584
C	4.779037	-1.615850	-1.232832	H	4.986708	0.734485	2.943500
C	5.068529	-2.394892	-0.107517	H	3.238486	0.603381	2.788590
H	5.118283	-2.417581	2.044646	H	4.173736	-0.745317	3.462885
H	4.954501	-2.026059	-2.224730	C	5.542485	-3.819921	-0.257690
C	0.487393	3.510817	0.673196	H	6.136090	-4.143416	0.604457
C	-0.378007	3.119020	1.705211	H	4.684183	-4.500666	-0.340962

H	6.149677	-3.951721	-1.160116

INT5B-IMes

G_{sol}(toluene) = -4729.911872 Hartree

C	2.756072	0.502691	-1.563963
C	1.663810	-0.168319	-3.496447
C	4.005515	0.603109	-2.233735
C	2.860876	-0.089103	-4.243099
H	0.735786	-0.487982	-3.956533
C	3.770454	1.294780	0.499717
C	5.144505	1.019525	-1.491384
C	4.028652	0.282713	-3.615818
H	2.839810	-0.328896	-5.301355
C	5.022004	1.355017	-0.159167
H	3.687873	1.582638	1.541380
H	6.107407	1.073592	-1.992254
H	4.965583	0.342961	-4.163749
H	5.895162	1.683024	0.398116
N	1.604953	0.103841	-2.200060
Rh	0.051909	-0.523600	-0.685568
Br	-0.608346	-2.385144	-2.259660
C	-0.709013	-1.577891	0.824150
C	-0.632401	-3.098620	2.535930
C	-1.923445	-2.994833	2.148992
H	-0.155749	-3.698139	3.295632
H	-2.820142	-3.477547	2.504571
N	0.096930	-2.227896	1.729874
N	-1.961906	-2.065354	1.110345
C	1.509991	-2.019697	1.907969
C	2.422252	-2.809968	1.191534
C	1.924471	-1.062913	2.846990
C	3.785300	-2.583850	1.407690
C	3.298489	-0.884827	3.037585
C	4.242156	-1.624044	2.317419
H	4.508434	-3.176565	0.851487
H	3.637175	-0.143501	3.758141
C	-3.208610	-1.554149	0.606988
C	-3.941854	-2.258341	-0.355446
C	-3.704483	-0.383753	1.210628
C	-5.149617	-1.691973	-0.789931
C	-4.916285	0.133377	0.754791
C	-5.641488	-0.494934	-0.267220
H	-5.721708	-2.215314	-1.553468
H	-5.304997	1.044115	1.205515
C	-3.534201	-3.619000	-0.862295
H	-2.493916	-3.852171	-0.638823
H	-4.173726	-4.390405	-0.409977
H	-3.656686	-3.683220	-1.947667
C	-2.948740	0.259417	2.345647
H	-2.790135	-0.448824	3.168632
H	-1.959647	0.592055	2.018556
H	-3.490264	1.121270	2.743654
C	-6.916585	0.120621	-0.791122
H	-6.696230	0.902898	-1.530074
H	-7.552983	-0.623037	-1.282681
H	-7.497509	0.589485	0.011701
C	0.915518	-0.240820	3.609059

H	0.296376	0.338290	2.916917
H	0.239773	-0.871061	4.199894
H	1.410947	0.456969	4.291108
C	1.948012	-3.866979	0.229178
H	1.262871	-4.572234	0.715926
H	1.397048	-3.423628	-0.607759
H	2.792268	-4.435903	-0.173049
C	5.720239	-1.366465	2.479506
H	6.092202	-0.742439	1.655783
H	5.941149	-0.843793	3.416494
H	6.297104	-2.298497	2.465075
C	2.644247	0.873981	-0.183848
C	1.280079	0.853388	0.267348
C	0.127140	1.397624	0.282539
Si	-0.753170	3.041564	0.299826
C	-0.428653	3.862269	2.002637
H	-0.712886	4.916542	1.869952
C	-2.611061	2.844102	-0.069608
H	-3.044876	2.279394	0.761291
C	-0.025789	4.163673	-1.095981
H	-0.753121	4.067621	-1.916138
C	1.336397	3.720027	-1.650477
H	1.280470	2.716631	-2.079226
H	1.676631	4.404472	-2.441489
H	2.112448	3.705187	-0.875743
C	0.012100	5.651632	-0.695543
H	0.293071	6.274072	-1.557132
H	-0.954752	6.018440	-0.331642
H	0.753391	5.838368	0.091354
C	1.068646	3.820528	2.361445
H	1.699436	4.256766	1.577413
H	1.267872	4.372975	3.290955
H	1.405032	2.787339	2.512112
C	-1.273973	3.303192	3.159198
H	-1.029029	2.255834	3.370601
H	-1.089121	3.868777	4.084077
H	-2.349185	3.357721	2.952076
C	-3.330651	4.205370	-0.126402
H	-2.973999	4.817554	-0.964569
H	-4.411813	4.066498	-0.270078
H	-3.195992	4.790385	0.792629
C	-2.863187	2.037286	-1.357008
H	-2.417754	1.037774	-1.309070
H	-3.941001	1.912717	-1.530033
H	-2.448684	2.543729	-2.239219

4aa

G_{sol}(toluene) = -1121.833487 Hartree

C	-2.558399	-2.336170	0.005019
C	-2.073030	-1.033380	0.033121
C	-3.003831	0.062983	0.057978
C	-4.406487	-0.220592	0.053843
C	-4.854191	-1.566839	0.024824
C	-3.944297	-2.600915	0.000631
H	-1.852090	-3.160455	-0.013931
C	-5.296454	0.884399	0.079140
H	-5.923018	-1.765872	0.021749

H	-4.287833	-3.631111	-0.021849
C	-4.788785	2.161378	0.105865
C	-3.382063	2.330260	0.107300
H	-6.368691	0.703471	0.076770
H	-5.439077	3.030622	0.125429
H	-2.959689	3.334043	0.127805
N	-2.517211	1.334704	0.084939
C	-0.673507	-0.774215	0.037950
C	0.525101	-0.542820	0.042076
Si	2.297242	-0.047097	0.054276
C	3.127166	-0.799632	-1.492081
H	4.169412	-0.444427	-1.484488
C	3.068725	-0.793268	1.636065
H	2.865215	-1.872296	1.561001
C	2.373205	1.865700	0.066501
H	2.272870	2.145982	1.126319
C	1.215736	2.538463	-0.696050
H	0.234926	2.211271	-0.334232
H	1.271079	3.631745	-0.590862
H	1.257242	2.315711	-1.769573
C	3.733461	2.393448	-0.430111
H	3.787879	3.486195	-0.325742
H	4.579889	1.970592	0.123906
H	3.888296	2.163728	-1.491744
C	2.471252	-0.321922	-2.800385
H	2.541354	0.764272	-2.927198
H	2.954632	-0.785196	-3.672352
H	1.407647	-0.589810	-2.833240
C	3.147044	-2.337699	-1.426439
H	2.128904	-2.745782	-1.380820
H	3.628896	-2.761972	-2.318646
H	3.693253	-2.709824	-0.551559
C	4.594365	-0.605023	1.711962
H	4.861256	0.453564	1.821528
H	5.010933	-1.132088	2.581891
H	5.108668	-0.986597	0.821414
C	2.387908	-0.281549	2.917484
H	1.301636	-0.428152	2.890565
H	2.773649	-0.806023	3.803124
H	2.574336	0.789209	3.072134
