# Supplementary material for: Tunably strained metallacycles enable modular differentiation of aza-arene C–H bonds
Source: Nat Commun. 2023 Jul 6;14:3986. doi: 10.1038/s41467-023-39753-2 (PMC10326034; doi:10.1038/s41467-023-39753-2)
Supplement: Supplementary file 4 — Supplementary Data 2 [file 41467_2023_39753_MOESM4_ESM.pdf]

Crystallographic Data

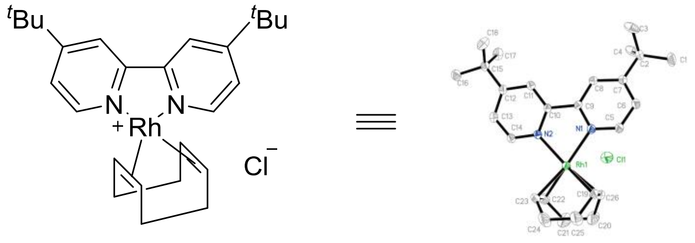

Rh(cod)(dtbpy)Cl

Supplementary Table 3. Crystal data and structure refinement for Rh(cod)(dtbpy)Cl

Identification code	Rh(cod)(dtbpy)Cl
Empirical formula	C27H38Cl3N2Rh
Formula weight	599.85
Temperature/K	193.00
Crystal system	monoclinic
Space group	P21/c
a/Å	11.7519(10)
b/Å	25.591(2)
c/Å	18.5358(17)
α / °	90
β / °	93.065(3)
γ / °	90
Volume/Å3	5566.5(9)
Z	8
ρ calcg/cm3	1.432
μ /mm-1	0.919
F(000)	2480.0
Crystal size/mm3	0.13 × 0.12 × 0.1
Radiation	MoKα (λ = 0.71073)
2Θ range for data collection/°	3.818 to 55.062
Index ranges	-15 ≤ h ≤ 15, 0 ≤ k ≤ 33, 0 ≤ l ≤ 24
Reflections collected	12777
Independent reflections	12777 [Rint = ?, Rsigma = 0.0844]
Data/restraints/parameters	12777/132/636
Goodness-of-fit on F2	1.031
Final R indexes [I>=2σ (I)]	R1 = 0.0752, wR2 = 0.1814
Final R indexes [all data]	R1 = 0.1028, wR2 = 0.2052
Largest diff. peak/hole / e Å-3	1.46/-1.51

Supplementary Table 4. Fractional Atomic Coordinates (×104) and Equivalent Isotropic Displacement Parameters (Å2 ×103) for Rh(cod)(dtbpy)Cl Ueq is defined as 1/3 of the trace of the orthogonalised UIJ tensor.

**Atom**	**x**	**y**	**z**	**U(eq)**
Rh(2)	1399.4(4)	4268.2(2)	8082.8(3)	25.34(14)
Rh(1)	6542.7(4)	4105.5(2)	6680.2(3)	27.78(14)
Cl(1)	8095.9(18)	4111.9(8)	4271.5(11)	44.7(4)
Cl(2)	2926(2)	4038.5(9)	522.3(12)	54.2(6)
Cl(3)	7522(4)	2522.1(13)	4745(2)	120.8(16)
Cl(4)	9923(4)	2587.3(15)	4554(3)	122.7(15)
N(3)	107(5)	4267(2)	8808(3)	27.8(12)
Cl(5A)	3590(13)	2634(5)	5873(7)	105(3)
N(4)	626(4)	4995(2)	7887(3)	25.9(11)
N(2)	5885(4)	4825(2)	6989(3)	27.1(11)
C(12)	4973(6)	5828(3)	7228(4)	30.6(14)
N(1)	5216(5)	4214(2)	5897(3)	32.2(13)
C(9)	4572(5)	4645(2)	5986(3)	26.7(13)
C(37)	-1451(6)	4749(3)	9262(4)	30.5(14)
C(48)	2995(6)	4483(3)	7697(4)	32.7(15)
C(36)	-509(6)	4710(2)	8844(4)	26.6(13)
C(32)	531(6)	5851(3)	7396(4)	37.3(17)
C(49)	2367(7)	4231(3)	7145(4)	38.2(17)
C(34)	-537(6)	5643(3)	8409(4)	30.9(14)
Cl(6)	4684(7)	2414(2)	4840(5)	132(3)
C(11)	4543(5)	5501(3)	6660(4)	27.6(13)
C(22)	7399(8)	3931(3)	7685(4)	49(2)
C(33)	-181(5)	6027(3)	7916(4)	32.0(15)
C(8)	3546(5)	4720(2)	5582(3)	27.1(13)
C(35)	-139(5)	5137(2)	8372(4)	27.4(13)
C(31)	921(6)	5349(3)	7393(4)	33.7(15)
C(26)	7449(6)	3525(3)	6102(4)	36.9(17)
C(38)	-1823(5)	4314(3)	9632(3)	26.5(13)
C(10)	5003(5)	5009(2)	6563(3)	25.2(12)
C(40)	-220(6)	3857(3)	9206(4)	33.6(15)
C(23)	8146(7)	4175(3)	7246(5)	46(2)
C(13)	5852(6)	5614(3)	7686(4)	36.6(16)
C(7)	3164(6)	4342(3)	5078(4)	30.6(14)
C(15)	4483(6)	6366(3)	7339(4)	36.3(16)
C(45)	2426(6)	3687(3)	8627(4)	37.0(16)
C(39)	-1162(6)	3865(3)	9618(4)	35.9(16)
C(6)	3872(7)	3922(3)	4971(4)	40.1(17)
C(47)	3975(6)	4250(4)	8147(5)	51(2)
C(2)	1976(6)	4383(3)	4698(4)	36.9(16)
C(19)	6691(7)	3281(3)	6541(5)	43.4(18)

C(41)	-2930(6)	4337(3)	10039(4)	31.6(15)
C(17)	3234(7)	6305(4)	7505(5)	50(2)
C(14)	6274(6)	5126(3)	7539(4)	32.9(15)
C(52)	1734(7)	3448(3)	8086(4)	41.0(18)
C(5)	4904(6)	3877(3)	5379(4)	38.4(17)
C(28)	-538(6)	6596(3)	7991(4)	35.7(16)
C(42)	-3281(8)	3793(3)	10292(6)	58(3)
C(43)	-2721(7)	4687(4)	10705(4)	50(2)
C(46)	3679(7)	3772(4)	8569(5)	60(3)
C(44)	-3883(6)	4559(4)	9553(5)	48(2)
C(50)	2620(10)	3673(3)	6911(5)	60(3)
C(3)	1916(8)	4872(4)	4238(5)	60(3)
C(25)	8708(7)	3560(5)	6271(6)	70(3)
C(27)	-1830(6)	6629(3)	7852(5)	48(2)
C(16)	5095(8)	6656(4)	7973(5)	53(2)
C(30)	-210(9)	6791(3)	8739(5)	60(3)
C(51)	2156(11)	3266(4)	7364(6)	68(3)
C(29)	7(8)	6938(3)	7423(6)	55(2)
C(4)	1074(7)	4422(4)	5261(5)	55(2)
C(20)	7012(13)	3010(4)	7249(6)	83(3)
C(18)	4563(9)	6684(3)	6656(5)	58(2)
Cl(6A)	3423(14)	2500(4)	4330(6)	136(4)
C(24)	9080(8)	3916(5)	6848(7)	82(3)
C(54)	8561(15)	2800(5)	4274(8)	112(6)
C(1)	1699(10)	3905(5)	4222(8)	101(5)
C(21)	7390(14)	3342(4)	7837(6)	94(4)
C(53A)	3610(40)	2218(7)	5159(6)	123(4)
C(53)	3352(13)	2246(9)	5074(13)	122(4)
Cl(5)	2902(11)	2642(4)	5736(7)	136(3)

Supplementary Table 5. Anisotropic Displacement Parameters (Å2×103) for Rh(cod)(dtbpy)Cl The Anisotropic displacement factor exponent takes the form: -2π2[h2a*2U11+2hka*b*U12+…].

Atom	U11	U22	U33	U23	U13	U12
Rh(2)	23.4(2)	25.9(2)	27.2(3)	0.86(19)	6.0(2)	-0.80(18)
Rh(1)	25.6(2)	28.4(3)	29.4(3)	-1.0(2)	1.7(2)	-0.38(18)
Cl(1)	46.0(11)	41.7(10)	45.6(11)	-2.7(8)	-4.3(9)	8.9(8)
Cl(2)	76.5(15)	45.5(11)	41.7(12)	-1.5(9)	12.9(10)	17.0(10)
Cl(3)	182(4)	53.3(17)	134(3)	-1.3(19)	74(3)	-2(2)
Cl(4)	162(4)	67(2)	139(4)	21(2)	9(3)	12(2)
N(3)	27(3)	27(3)	31(3)	4(2)	9(2)	4(2)
Cl(5A)	149(8)	62(4)	105(6)	9(4)	12(6)	-9(6)
N(4)	26(3)	26(3)	26(3)	-3(2)	2(2)	-4(2)
N(2)	26(3)	28(3)	27(3)	-1(2)	2(2)	-1(2)

C(12)	27(3)	33(4)	33(4)	-8(3)	7(3)	-3(3)
N(1)	26(3)	33(3)	38(3)	-7(2)	-1(2)	1(2)
C(9)	30(3)	26(3)	25(3)	0(2)	5(3)	1(2)
C(37)	31(3)	28(3)	33(4)	-4(3)	8(3)	-3(3)
C(48)	26(3)	35(4)	39(4)	2(3)	16(3)	-4(3)
C(36)	31(3)	23(3)	26(3)	2(2)	3(3)	-4(2)
C(32)	31(4)	34(4)	46(4)	15(3)	-7(3)	0(3)
C(49)	51(4)	34(4)	30(4)	6(3)	17(3)	6(3)
C(34)	31(3)	29(3)	33(4)	-1(3)	4(3)	-1(3)
Cl(6)	139(5)	62(3)	204(7)	-18(4)	89(5)	-18(3)
C(11)	21(3)	34(3)	29(3)	-1(3)	6(2)	-2(2)
C(22)	68(6)	48(5)	29(4)	4(3)	-12(4)	7(4)
C(33)	22(3)	25(3)	49(4)	6(3)	0(3)	-3(2)
C(8)	27(3)	28(3)	26(3)	0(3)	3(2)	1(2)
C(35)	23(3)	30(3)	29(3)	-2(3)	4(3)	0(2)
C(31)	40(4)	31(4)	31(4)	3(3)	5(3)	-3(3)
C(26)	32(4)	35(4)	44(4)	-17(3)	4(3)	8(3)
C(38)	24(3)	33(3)	23(3)	-4(3)	9(2)	-5(2)
C(10)	22(3)	27(3)	27(3)	-1(2)	4(2)	-4(2)
C(40)	34(4)	27(3)	42(4)	8(3)	11(3)	3(3)
C(23)	36(4)	51(5)	50(5)	-14(4)	-14(4)	6(3)
C(13)	35(4)	38(4)	37(4)	-7(3)	4(3)	-5(3)
C(7)	33(4)	32(4)	26(3)	2(3)	4(3)	-2(3)
C(15)	31(4)	36(4)	42(4)	-10(3)	4(3)	3(3)
C(45)	32(4)	42(4)	38(4)	11(3)	5(3)	8(3)
C(39)	40(4)	29(4)	39(4)	11(3)	12(3)	1(3)
C(6)	40(4)	43(4)	36(4)	-12(3)	-7(3)	5(3)
C(47)	25(4)	62(5)	68(6)	19(4)	6(4)	-1(3)
C(2)	35(4)	44(4)	31(4)	-5(3)	-3(3)	-6(3)
C(19)	46(4)	28(4)	54(5)	-8(3)	-14(4)	1(3)
C(41)	27(3)	37(4)	33(4)	-5(3)	15(3)	-5(3)
C(17)	35(4)	55(5)	61(6)	-18(4)	17(4)	2(3)
C(14)	31(3)	37(4)	30(4)	-4(3)	0(3)	-1(3)
C(52)	47(4)	28(3)	50(5)	8(3)	20(4)	4(3)
C(5)	37(4)	36(4)	41(4)	-13(3)	-3(3)	7(3)
C(28)	33(3)	28(3)	46(4)	7(3)	3(3)	2(3)
C(42)	52(5)	47(5)	80(7)	4(4)	41(5)	-10(4)
C(43)	49(5)	63(6)	39(5)	-19(4)	12(4)	-14(4)
C(46)	30(4)	92(7)	57(6)	36(5)	3(4)	6(4)
C(44)	30(4)	68(6)	46(5)	1(4)	2(3)	-1(4)
C(50)	101(8)	39(5)	42(5)	-4(4)	28(5)	12(5)
C(3)	43(5)	89(7)	47(5)	33(5)	-5(4)	-8(5)
C(25)	31(4)	97(7)	84(7)	-38(5)	3(4)	9(4)
C(27)	32(4)	39(4)	74(6)	5(4)	4(4)	6(3)

C(16)	49(5)	50(5)	59(6)	-19(4)	-8(4)	8(4)
C(30)	80(7)	35(5)	64(6)	-6(4)	-16(5)	1(4)
C(51)	109(9)	38(5)	61(6)	-12(4)	40(6)	11(5)
C(29)	48(5)	32(4)	87(7)	14(4)	19(5)	4(3)
C(4)	30(4)	89(7)	44(5)	20(5)	1(3)	-2(4)
C(20)	139(9)	43(5)	64(6)	13(4)	-25(6)	1(6)
C(18)	79(7)	37(5)	59(6)	1(4)	16(5)	0(4)
Cl(6A)	222(9)	69(5)	119(7)	-4(4)	33(6)	-30(6)
C(24)	33(4)	115(8)	95(7)	-58(6)	-8(5)	14(5)
C(54)	177(16)	57(7)	110(12)	12(7)	65(11)	21(9)
C(1)	73(8)	79(8)	142(12)	-64(8)	-68(8)	24(6)
C(21)	172(10)	52(5)	56(6)	16(5)	-24(6)	13(6)
C(53A)	152(7)	89(6)	131(7)	-2(6)	37(6)	-18(6)
C(53)	152(7)	89(6)	130(7)	-2(6)	45(6)	-16(5)
Cl(5)	183(8)	90(4)	146(6)	-18(5)	93(6)	-17(6)

Supplementary Table 6. Bond Lengths for Rh(cod)(dtbpy)Cl.

**Atom**	**Atom**	**Length/Å**	**Atom**	**Atom**	**Length/Å**
Rh(2)	N(3)	2.081(5)	Cl(6)	C(53)	1.703(5)
Rh(2)	N(4)	2.092(5)	C(11)	C(10)	1.385(9)
Rh(2)	C(48)	2.116(6)	C(22)	C(23)	1.377(13)
Rh(2)	C(49)	2.129(7)	C(22)	C(21)	1.534(13)
Rh(2)	C(45)	2.135(7)	C(33)	C(28)	1.525(9)
Rh(2)	C(52)	2.134(7)	C(8)	C(7)	1.401(9)
Rh(1)	N(2)	2.088(5)	C(26)	C(19)	1.387(11)
Rh(1)	N(1)	2.092(6)	C(26)	C(25)	1.498(11)
Rh(1)	C(22)	2.116(8)	C(38)	C(39)	1.389(10)
Rh(1)	C(26)	2.148(7)	C(38)	C(41)	1.539(8)
Rh(1)	C(23)	2.115(8)	C(40)	C(39)	1.379(10)
Rh(1)	C(19)	2.133(7)	C(23)	C(24)	1.508(12)
Cl(3)	C(54)	1.695(15)	C(13)	C(14)	1.377(10)
Cl(4)	C(54)	1.742(18)	C(7)	C(6)	1.382(10)
N(3)	C(36)	1.348(8)	C(7)	C(2)	1.532(10)
N(3)	C(40)	1.351(8)	C(15)	C(17)	1.523(10)
Cl(5A)	C(53A)	1.699(5)	C(15)	C(16)	1.537(11)
N(4)	C(35)	1.354(8)	C(15)	C(18)	1.512(12)
N(4)	C(31)	1.348(9)	C(45)	C(52)	1.398(11)
N(2)	C(10)	1.353(8)	C(45)	C(46)	1.498(10)
N(2)	C(14)	1.339(8)	C(6)	C(5)	1.400(10)
C(12)	C(11)	1.419(9)	C(47)	C(46)	1.502(12)
C(12)	C(13)	1.412(10)	C(2)	C(3)	1.514(12)
C(12)	C(15)	1.510(10)	C(2)	C(4)	1.530(11)
N(1)	C(9)	1.352(8)	C(2)	C(1)	1.533(12)
N(1)	C(5)	1.327(9)	C(19)	C(20)	1.515(12)

C(9)	C(8)	1.398(9)	C(41)	C(42)	1.532(10)
C(9)	C(10)	1.486(9)	C(41)	C(43)	1.534(10)
C(37)	C(36)	1.389(9)	C(41)	C(44)	1.510(11)
C(37)	C(38)	1.389(9)	C(52)	C(51)	1.524(11)
C(48)	C(49)	1.387(11)	C(28)	C(27)	1.529(10)
C(48)	C(47)	1.508(11)	C(28)	C(30)	1.505(12)
C(36)	C(35)	1.480(9)	C(28)	C(29)	1.535(11)
C(32)	C(33)	1.383(11)	C(50)	C(51)	1.461(13)
C(32)	C(31)	1.364(10)	C(25)	C(24)	1.455(13)
C(49)	C(50)	1.526(10)	C(20)	C(21)	1.434(15)
C(34)	C(33)	1.420(9)	Cl(6A)	C(53A)	1.702(5)
C(34)	C(35)	1.379(9)	C(53)	Cl(5)	1.697(5)

Supplementary Table 7. Bond Angles for Rh(cod)(dtbpy)Cl.

**Atom**	**Atom**	**Atom**	**Angle/°**	**Atom**	**Atom**	**Atom**	**Angle/°**
N(3)	Rh(2)	N(4)	77.8(2)	N(4)	C(35)	C(34)	121.6(6)
N(3)	Rh(2)	C(48)	155.4(3)	C(34)	C(35)	C(36)	123.5(6)
N(3)	Rh(2)	C(49)	165.3(3)	N(4)	C(31)	C(32)	122.2(7)
N(3)	Rh(2)	C(45)	96.1(3)	C(19)	C(26)	Rh(1)	70.5(4)
N(3)	Rh(2)	C(52)	97.9(2)	C(19)	C(26)	C(25)	124.2(8)
N(4)	Rh(2)	C(48)	95.5(2)	C(25)	C(26)	Rh(1)	111.4(5)
N(4)	Rh(2)	C(49)	98.3(2)	C(37)	C(38)	C(41)	120.5(6)
N(4)	Rh(2)	C(45)	158.5(3)	C(39)	C(38)	C(37)	117.6(6)
N(4)	Rh(2)	C(52)	162.2(3)	C(39)	C(38)	C(41)	121.9(6)
C(48)	Rh(2)	C(49)	38.1(3)	N(2)	C(10)	C(9)	115.0(5)
C(48)	Rh(2)	C(45)	81.5(3)	N(2)	C(10)	C(11)	122.2(6)
C(48)	Rh(2)	C(52)	95.2(3)	C(11)	C(10)	C(9)	122.9(6)
C(49)	Rh(2)	C(45)	92.4(3)	N(3)	C(40)	C(39)	123.4(6)
C(49)	Rh(2)	C(52)	81.5(3)	C(22)	C(23)	Rh(1)	71.0(5)
C(52)	Rh(2)	C(45)	38.2(3)	C(22)	C(23)	C(24)	126.4(9)
N(2)	Rh(1)	N(1)	78.4(2)	C(24)	C(23)	Rh(1)	111.9(6)
N(2)	Rh(1)	C(22)	96.3(3)	C(14)	C(13)	C(12)	119.6(7)
N(2)	Rh(1)	C(26)	161.3(3)	C(8)	C(7)	C(2)	120.7(6)
N(2)	Rh(1)	C(23)	97.1(3)	C(6)	C(7)	C(8)	117.3(6)
N(2)	Rh(1)	C(19)	159.8(3)	C(6)	C(7)	C(2)	121.9(6)
N(1)	Rh(1)	C(22)	160.2(3)	C(12)	C(15)	C(17)	108.2(6)
N(1)	Rh(1)	C(26)	96.7(3)	C(12)	C(15)	C(16)	112.2(6)
N(1)	Rh(1)	C(23)	160.8(3)	C(12)	C(15)	C(18)	109.6(6)
N(1)	Rh(1)	C(19)	96.3(3)	C(17)	C(15)	C(16)	108.2(7)
C(22)	Rh(1)	C(26)	93.9(3)	C(18)	C(15)	C(17)	109.1(7)
C(22)	Rh(1)	C(19)	82.0(3)	C(18)	C(15)	C(16)	109.5(7)
C(23)	Rh(1)	C(22)	38.0(4)	C(52)	C(45)	Rh(2)	70.9(4)
C(23)	Rh(1)	C(26)	81.6(3)	C(52)	C(45)	C(46)	123.2(8)
C(23)	Rh(1)	C(19)	93.8(3)	C(46)	C(45)	Rh(2)	113.4(5)

C(19)	Rh(1)	C(26)	37.8(3)	C(40)	C(39)	C(38)	119.3(6)
C(36)	N(3)	Rh(2)	116.3(4)	C(7)	C(6)	C(5)	119.8(7)
C(36)	N(3)	C(40)	117.2(5)	C(46)	C(47)	C(48)	114.8(6)
C(40)	N(3)	Rh(2)	126.3(4)	C(7)	C(2)	C(1)	111.7(7)
C(35)	N(4)	Rh(2)	115.0(4)	C(3)	C(2)	C(7)	109.2(6)
C(31)	N(4)	Rh(2)	126.2(5)	C(3)	C(2)	C(4)	108.5(7)
C(31)	N(4)	C(35)	118.2(6)	C(3)	C(2)	C(1)	109.5(9)
C(10)	N(2)	Rh(1)	115.5(4)	C(4)	C(2)	C(7)	109.8(6)
C(14)	N(2)	Rh(1)	126.8(5)	C(4)	C(2)	C(1)	108.1(9)
C(14)	N(2)	C(10)	117.6(6)	C(26)	C(19)	Rh(1)	71.7(4)
C(11)	C(12)	C(15)	121.1(6)	C(26)	C(19)	C(20)	125.2(9)
C(13)	C(12)	C(11)	115.9(6)	C(20)	C(19)	Rh(1)	111.5(6)
C(13)	C(12)	C(15)	123.0(6)	C(42)	C(41)	C(38)	111.4(6)
C(9)	N(1)	Rh(1)	115.1(4)	C(42)	C(41)	C(43)	108.5(7)
C(5)	N(1)	Rh(1)	125.6(5)	C(43)	C(41)	C(38)	108.3(6)
C(5)	N(1)	C(9)	118.9(6)	C(44)	C(41)	C(38)	110.0(6)
N(1)	C(9)	C(8)	121.2(6)	C(44)	C(41)	C(42)	108.7(7)
N(1)	C(9)	C(10)	115.3(6)	C(44)	C(41)	C(43)	110.0(7)
C(8)	C(9)	C(10)	123.4(6)	N(2)	C(14)	C(13)	124.1(7)
C(36)	C(37)	C(38)	119.9(6)	C(45)	C(52)	Rh(2)	70.9(4)
C(49)	C(48)	Rh(2)	71.5(4)	C(45)	C(52)	C(51)	124.0(8)
C(49)	C(48)	C(47)	125.7(7)	C(51)	C(52)	Rh(2)	111.5(5)
C(47)	C(48)	Rh(2)	112.0(5)	N(1)	C(5)	C(6)	122.4(7)
N(3)	C(36)	C(37)	122.4(6)	C(33)	C(28)	C(27)	108.2(6)
N(3)	C(36)	C(35)	114.6(6)	C(33)	C(28)	C(29)	110.8(6)
C(37)	C(36)	C(35)	123.0(6)	C(27)	C(28)	C(29)	107.5(7)
C(31)	C(32)	C(33)	121.6(7)	C(30)	C(28)	C(33)	110.1(6)
C(48)	C(49)	Rh(2)	70.4(4)	C(30)	C(28)	C(27)	109.9(7)
C(48)	C(49)	C(50)	122.7(8)	C(30)	C(28)	C(29)	110.3(7)
C(50)	C(49)	Rh(2)	113.2(5)	C(45)	C(46)	C(47)	114.4(7)
C(35)	C(34)	C(33)	120.3(6)	C(51)	C(50)	C(49)	114.8(7)
C(10)	C(11)	C(12)	120.5(6)	C(24)	C(25)	C(26)	116.5(7)
C(23)	C(22)	Rh(1)	71.0(5)	C(50)	C(51)	C(52)	115.8(7)
C(23)	C(22)	C(21)	124.4(10)	C(21)	C(20)	C(19)	116.1(8)
C(21)	C(22)	Rh(1)	111.2(6)	C(25)	C(24)	C(23)	115.9(8)
C(32)	C(33)	C(34)	115.7(6)	Cl(3)	C(54)	Cl(4)	113.1(8)
C(32)	C(33)	C(28)	123.6(6)	C(20)	C(21)	C(22)	116.6(9)
C(34)	C(33)	C(28)	120.7(6)	Cl(5A)	C(53A)	Cl(6A)	115.6(12)
C(9)	C(8)	C(7)	120.0(6)	Cl(5)	C(53)	Cl(6)	111.1(7)
N(4)	C(35)	C(36)	114.8(5)				

Supplementary Table 8. Hydrogen Atom Coordinates (Å×104) and Isotropic Displacement Parameters (Å2×103) for Rh(cod)(dtbpy)Cl.

Atom	x	y	z	U(eq)

H(37)	-1840.72	5072.96	9295.46	37
H(48)	3074.41	4867.62	7616.41	39
H(32)	752.87	6086.33	7032.64	45
H(49)	2085.89	4466.04	6743.14	46
H(34)	-1051.89	5734.59	8765.77	37
H(11)	3935.08	5619.34	6342.81	33
H(22)	7157.45	4151.9	8094.69	59
H(8)	3109.21	5026.5	5648.86	32
H(31)	1417.85	5246.45	7031.06	40
H(26)	7227.34	3506.74	5573.96	44
H(40)	222.64	3546.01	9201.68	40
H(23)	8333.15	4541.5	7398.35	55
H(13)	6150.73	5805.62	8091.98	44
H(45)	2199.82	3608.79	9128.44	44
H(39)	-1357.05	3566.73	9890.12	43
H(6)	3659.31	3663.58	4620.9	48
H(47A)	4588.72	4158.45	7824.41	62
H(47B)	4279.09	4519.03	8489.42	62
H(19)	6022.58	3120.16	6268.42	52
H(17A)	2830.41	6114.7	7109.46	75
H(17B)	2889.47	6650.73	7554.54	75
H(17C)	3178.08	6109.4	7956.06	75
H(14)	6878.8	4994.16	7847.19	40
H(52)	1105.92	3230.42	8269.83	49
H(5)	5399.35	3595.54	5282.34	46
H(42A)	-2698.36	3657.6	10640.5	87
H(42B)	-3358.25	3557.35	9875.35	87
H(42C)	-4011.84	3816.75	10520.97	87
H(43A)	-2478.97	5034.46	10551.74	75
H(43B)	-2124.71	4532.38	11027.02	75
H(43C)	-3426.59	4717.85	10960.54	75
H(46A)	4005.48	3462.3	8336.63	71
H(46B)	4041.02	3800.86	9061.77	71
H(44A)	-3953.57	4356.47	9104.03	72
H(44B)	-3714.41	4924.31	9440.61	72
H(44C)	-4600	4540.65	9798.53	72
H(50A)	2307.17	3623.97	6409.2	72
H(50B)	3456.08	3627.74	6910.14	72
H(3A)	2109.7	5177.04	4540.63	90
H(3B)	1142.35	4912.69	4020.87	90
H(3C)	2456.63	4843.73	3855.68	90
H(25A)	8991.92	3206.2	6399.26	84
H(25B)	9075.35	3667.51	5825.84	84
H(27A)	-2039.84	6492.56	7368.74	73

H(27B)	-2074.19	6994.07	7882.87	73
H(27C)	-2204.92	6421.16	8214.48	73
H(16A)	5914.58	6667.95	7900.7	80
H(16B)	4963.71	6472.34	8425.32	80
H(16C)	4797.63	7012.97	7998.69	80
H(30A)	-437.2	7158.14	8781.16	91
H(30B)	617.3	6761.69	8828.68	91
H(30C)	-595.04	6581.6	9094.96	91
H(51A)	2752.57	2998.5	7459.9	82
H(51B)	1514.61	3095.69	7087.71	82
H(29A)	838.5	6908.82	7479.62	83
H(29B)	-217.27	7303.28	7489	83
H(29C)	-251.91	6821.1	6938.5	83
H(4A)	1207.42	4737.76	5552.93	82
H(4B)	1121.21	4113.93	5575.23	82
H(4C)	315.06	4439.51	5016.04	82
H(20A)	6342.56	2810.18	7397.87	100
H(20B)	7624.4	2755.32	7164.48	100
H(18A)	4117.79	6513.56	6260.32	87
H(18B)	5361.29	6708.62	6532.92	87
H(18C)	4260.26	7035.16	6732.78	87
H(24A)	9552.95	4192.59	6640.37	98
H(24B)	9574.57	3720.18	7202.92	98
H(54A)	8523.19	3183.83	4329.24	135
H(54B)	8421.97	2717.26	3755.18	135
H(1A)	2278.82	3868.01	3864.48	151
H(1B)	949.2	3951.22	3973.09	151
H(1C)	1693.04	3591.15	4524.17	151
H(21A)	8171.68	3235.61	7999.3	113
H(21B)	6894.48	3279.22	8244.01	113
H(53A)	3012.97	1952.26	5209.31	147
H(53B)	4356.59	2034.09	5182.64	147
H(53C)	2815.99	2271.41	4644.8	147
H(53D)	3354.92	1879.12	5243.85	147

Supplementary Table 9. Atomic Occupancy for Rh(cod)(dtbpy)Cl.

**Atom**	**Occupancy**	**Atom**	**Occupancy**	**Atom**	**Occupancy**
Cl(5A)	0.405(8)	Cl(6)	0.595(8)	Cl(6A)	0.405(8)
C(53A)	0.405(8)	H(53A)	0.405(8)	H(53B)	0.405(8)
C(53)	0.595(8)	H(53C)	0.595(8)	H(53D)	0.595(8)
Cl(5)	0.595(8)				

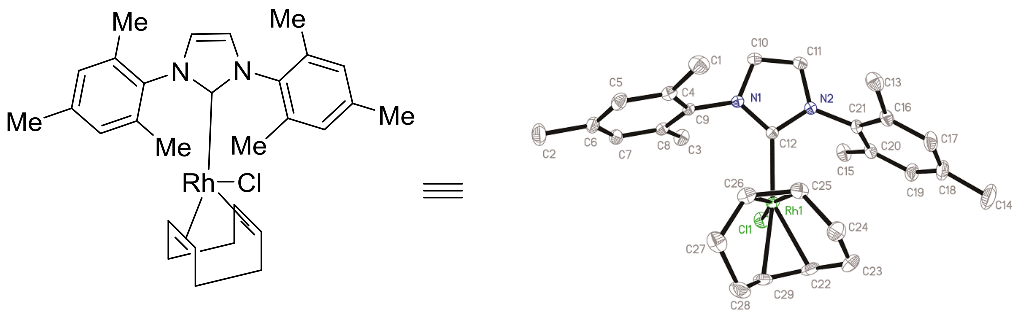

Rh(cod)(IMes)Cl

Supplementary Table 10. Crystal data and structure refinement for Rh(cod)(IMes)Cl.

Identification code	Rh(cod)(IMes)Cl
Empirical formula	C29 H36 Cl N2 Rh
Formula weight	550.96
Temperature	296(2) K
Wavelength	0.71073 Å
Crystal system	Tetragonal
Space group	I41/a
Unit cell dimensions	a = 32.6076(8) Å α = 90 °.
	b = 32.6076(8) Å β = 90 °.
	c = 9.9491(4) Å γ = 90 °.
Volume	10578.4(7) Å3
Z	16
Density (calculated)	1.384 Mg/m3
Absorption coefficient	0.766 mm-1
F(000)	4576
Crystal size	0.120 x 0.110 x 0.080 mm3
Theta range for data collection	2.140 to 27.580 °.
Index ranges	-41<=h<=42, -42<=k<=42, -12<=l<=12
Reflections collected	48770
Independent reflections	6090 [R(int) = 0.0400]
Completeness to theta = 25.242 °	99.7 %
Refinement method	Full-matrix least-squares on F2
Data / restraints / parameters	6090 / 0 / 304
Goodness-of-fit on F2	1.044
Final R indices [I>2sigma(I)]	R1 = 0.0224, wR2 = 0.0581
R indices (all data)	R1 = 0.0261, wR2 = 0.0607
Extinction coefficient	n/a
Largest diff. peak and hole	0.277 and -0.329 e.Å-3

Supplementary Table 11. Atomic coordinates (x 104) and equivalent isotropic displacement parameters (Å2x 103) for Rh(cod)(IMes)Cl. U(eq) is defined as one third of the trace of the orthogonalized Uij tensor.

	x	y	z	U(eq)
C(1)	3034(1)	7595(1)	6344(2)	45(1)
C(2)	4462(1)	7742(1)	8117(2)	54(1)
C(3)	3898(1)	6333(1)	7193(2)	38(1)
C(4)	3434(1)	7397(1)	6727(2)	30(1)
C(5)	3753(1)	7636(1)	7225(2)	37(1)
C(6)	4122(1)	7468(1)	7618(2)	35(1)
C(7)	4168(1)	7046(1)	7573(2)	33(1)
C(8)	3857(1)	6790(1)	7107(2)	27(1)
C(9)	3499(1)	6975(1)	6642(2)	24(1)
C(10)	2824(1)	6622(1)	6862(2)	33(1)
C(11)	2569(1)	6423(1)	6036(2)	33(1)
C(12)	3140(1)	6590(1)	4833(2)	22(1)
C(13)	2135(1)	6874(1)	3421(2)	42(1)
C(14)	2171(1)	5811(1)	-247(3)	78(1)
C(15)	2939(1)	5565(1)	4048(2)	40(1)
C(16)	2306(1)	6479(1)	2876(2)	32(1)
C(17)	2174(1)	6332(1)	1637(2)	43(1)
C(18)	2303(1)	5958(1)	1128(2)	48(1)
C(19)	2553(1)	5717(1)	1928(2)	42(1)
C(20)	2693(1)	5848(1)	3170(2)	31(1)
C(21)	2584(1)	6240(1)	3590(2)	26(1)
C(22)	3665(1)	6516(1)	1121(2)	43(1)
C(23)	3313(1)	6687(1)	304(2)	52(1)
C(24)	3143(1)	7088(1)	854(2)	48(1)
C(25)	3165(1)	7103(1)	2376(2)	34(1)
C(26)	3495(1)	7269(1)	3110(2)	33(1)
C(27)	3881(1)	7448(1)	2519(2)	44(1)
C(28)	4060(1)	7187(1)	1388(2)	50(1)
C(29)	3990(1)	6735(1)	1623(2)	42(1)
Cl(1)	3874(1)	6012(1)	3781(1)	36(1)
N(1)	3172(1)	6722(1)	6121(1)	24(1)
N(2)	2764(1)	6405(1)	4798(1)	24(1)
Rh(1)	3526(1)	6629(1)	3213(1)	23(1)

Supplementary Table 12. Bond lengths [Å] and angles [°] for Rh(cod)(IMes)Cl.

C(13)-C(16)	1.504(2)	C(18)-C(14)-H(14B)	109.5
C(13)-H(13A)	0.9600	H(14A)-C(14)-H(14B)	109.5
C(13)-H(13B)	0.9600	C(18)-C(14)-H(14C)	109.5
C(13)-H(13C)	0.9600	H(14A)-C(14)-H(14C)	109.5
C(14)-C(18)	1.511(3)	H(14B)-C(14)-H(14C)	109.5

C(14)-H(14A)	0.9600	C(20)-C(15)-H(15A)	109.5
C(14)-H(14B)	0.9600	C(20)-C(15)-H(15B)	109.5
C(14)-H(14C)	0.9600	H(15A)-C(15)-H(15B)	109.5
C(15)-C(20)	1.501(2)	C(20)-C(15)-H(15C)	109.5
C(15)-H(15A)	0.9600	H(15A)-C(15)-H(15C)	109.5
C(15)-H(15B)	0.9600	H(15B)-C(15)-H(15C)	109.5
C(15)-H(15C)	0.9600	C(21)-C(16)-C(17)	117.41(16)
C(16)-C(21)	1.393(2)	C(21)-C(16)-C(13)	122.47(17)
C(16)-C(17)	1.391(3)	C(17)-C(16)-C(13)	120.06(16)
C(17)-C(18)	1.388(3)	C(18)-C(17)-C(16)	122.17(18)
C(17)-H(17)	0.9300	C(18)-C(17)-H(17)	118.9
C(18)-C(19)	1.383(3)	C(16)-C(17)-H(17)	118.9
C(19)-C(20)	1.386(3)	C(19)-C(18)-C(17)	117.96(18)
C(19)-H(19)	0.9300	C(19)-C(18)-C(14)	120.6(2)
C(20)-C(21)	1.391(2)	C(17)-C(18)-C(14)	121.4(2)
C(21)-N(2)	1.442(2)	C(18)-C(19)-C(20)	122.19(18)
C(22)-C(29)	1.373(3)	C(18)-C(19)-H(19)	118.9
C(22)-C(23)	1.513(3)	C(20)-C(19)-H(19)	118.9
C(22)-Rh(1)	2.1622(18)	C(19)-C(20)-C(21)	117.80(16)
C(22)-H(22)	0.9800	C(19)-C(20)-C(15)	120.40(17)
C(23)-C(24)	1.522(3)	C(21)-C(20)-C(15)	121.74(16)
C(23)-H(23A)	0.9700	C(20)-C(21)-C(16)	121.96(15)
C(23)-H(23B)	0.9700	C(20)-C(21)-N(2)	119.36(14)
C(24)-C(25)	1.517(3)	C(16)-C(21)-N(2)	118.68(14)
C(24)-H(24A)	0.9700	C(29)-C(22)-C(23)	126.0(2)
C(24)-H(24B)	0.9700	C(29)-C(22)-Rh(1)	73.93(11)
C(25)-C(26)	1.408(3)	C(23)-C(22)-Rh(1)	107.15(13)
C(25)-Rh(1)	2.1146(17)	C(29)-C(22)-H(22)	113.9
C(25)-H(25)	0.9800	C(23)-C(22)-H(22)	113.9
C(26)-C(27)	1.508(3)	Rh(1)-C(22)-H(22)	113.9
C(26)-Rh(1)	2.0913(16)	C(22)-C(23)-C(24)	113.66(17)
C(26)-H(26)	0.9800	C(22)-C(23)-H(23A)	108.8
C(27)-C(28)	1.527(3)	C(24)-C(23)-H(23A)	108.8
C(27)-H(27A)	0.9700	C(22)-C(23)-H(23B)	108.8
C(27)-H(27B)	0.9700	C(24)-C(23)-H(23B)	108.8
C(28)-C(29)	1.508(3)	H(23A)-C(23)-H(23B)	107.7
C(28)-H(28A)	0.9700	C(25)-C(24)-C(23)	111.70(17)
C(28)-H(28B)	0.9700	C(25)-C(24)-H(24A)	109.3
C(29)-Rh(1)	2.2173(17)	C(23)-C(24)-H(24A)	109.3
C(29)-H(29)	0.9800	C(25)-C(24)-H(24B)	109.3

Cl(1)-Rh(1)	2.3778(4)	C(23)-C(24)-H(24B)	109.3
C(4)-C(1)-H(1A)	109.5	H(24A)-C(24)-H(24B)	107.9
C(4)-C(1)-H(1B)	109.5	C(26)-C(25)-C(24)	124.48(17)
H(1A)-C(1)-H(1B)	109.5	C(26)-C(25)-Rh(1)	69.55(9)
C(4)-C(1)-H(1C)	109.5	C(24)-C(25)-Rh(1)	113.28(13)
H(1A)-C(1)-H(1C)	109.5	C(26)-C(25)-H(25)	113.9
H(1B)-C(1)-H(1C)	109.5	C(24)-C(25)-H(25)	113.9
C(6)-C(2)-H(2A)	109.5	Rh(1)-C(25)-H(25)	113.9
C(6)-C(2)-H(2B)	109.5	C(25)-C(26)-C(27)	125.75(17)
H(2A)-C(2)-H(2B)	109.5	C(25)-C(26)-Rh(1)	71.34(10)
C(6)-C(2)-H(2C)	109.5	C(27)-C(26)-Rh(1)	111.44(12)
H(2A)-C(2)-H(2C)	109.5	C(25)-C(26)-H(26)	113.6
H(2B)-C(2)-H(2C)	109.5	C(27)-C(26)-H(26)	113.6
C(8)-C(3)-H(3A)	109.5	Rh(1)-C(26)-H(26)	113.6
C(8)-C(3)-H(3B)	109.5	C(26)-C(27)-C(28)	113.01(17)
H(3A)-C(3)-H(3B)	109.5	C(26)-C(27)-H(27A)	109.0
C(8)-C(3)-H(3C)	109.5	C(28)-C(27)-H(27A)	109.0
H(3A)-C(3)-H(3C)	109.5	C(26)-C(27)-H(27B)	109.0
H(3B)-C(3)-H(3C)	109.5	C(28)-C(27)-H(27B)	109.0
C(5)-C(4)-C(9)	117.61(16)	H(27A)-C(27)-H(27B)	107.8
C(5)-C(4)-C(1)	119.75(16)	C(29)-C(28)-C(27)	111.86(16)
C(9)-C(4)-C(1)	122.61(16)	C(29)-C(28)-H(28A)	109.2
C(6)-C(5)-C(4)	122.04(16)	C(27)-C(28)-H(28A)	109.2
C(6)-C(5)-H(5)	119.0	C(29)-C(28)-H(28B)	109.2
C(4)-C(5)-H(5)	119.0	C(27)-C(28)-H(28B)	109.2
C(5)-C(6)-C(7)	118.59(16)	H(28A)-C(28)-H(28B)	107.9
C(5)-C(6)-C(2)	119.95(18)	C(22)-C(29)-C(28)	124.7(2)
C(7)-C(6)-C(2)	121.44(18)	C(22)-C(29)-Rh(1)	69.56(11)
C(6)-C(7)-C(8)	121.90(16)	C(28)-C(29)-Rh(1)	111.54(13)
C(6)-C(7)-H(7)	119.0	C(22)-C(29)-H(29)	114.2
C(8)-C(7)-H(7)	119.0	C(28)-C(29)-H(29)	114.2
C(7)-C(8)-C(9)	117.52(15)	Rh(1)-C(29)-H(29)	114.2
C(7)-C(8)-C(3)	120.75(15)	C(12)-N(1)-C(10)	111.35(13)
C(9)-C(8)-C(3)	121.60(15)	C(12)-N(1)-C(9)	125.26(13)
C(8)-C(9)-C(4)	122.13(15)	C(10)-N(1)-C(9)	123.04(13)
C(8)-C(9)-N(1)	119.44(14)	C(12)-N(2)-C(11)	111.70(13)
C(4)-C(9)-N(1)	118.33(14)	C(12)-N(2)-C(21)	123.47(13)
C(11)-C(10)-N(1)	107.16(15)	C(11)-N(2)-C(21)	124.69(13)
C(11)-C(10)-H(10)	126.4	C(12)-Rh(1)-C(26)	94.05(6)
N(1)-C(10)-H(10)	126.4	C(12)-Rh(1)-C(25)	90.76(6)
C(10)-C(11)-N(2)	106.30(14)	C(26)-Rh(1)-C(25)	39.11(7)

C(10)-C(11)-H(11)	126.8	C(12)-Rh(1)-C(22)	151.18(7)
N(2)-C(11)-H(11)	126.8	C(26)-Rh(1)-C(22)	97.65(8)
N(1)-C(12)-N(2)	103.48(13)	C(25)-Rh(1)-C(22)	82.12(8)
N(1)-C(12)-Rh(1)	132.60(11)	C(12)-Rh(1)-C(29)	172.07(7)
N(2)-C(12)-Rh(1)	123.92(11)	C(26)-Rh(1)-C(29)	80.91(8)
C(16)-C(13)-H(13A)	109.5	C(25)-Rh(1)-C(29)	89.14(7)
C(16)-C(13)-H(13B)	109.5	C(22)-Rh(1)-C(29)	36.51(8)
H(13A)-C(13)-H(13B)	109.5	C(12)-Rh(1)-Cl(1)	93.09(4)
C(16)-C(13)-H(13C)	109.5	C(26)-Rh(1)-Cl(1)	151.43(5)
H(13A)-C(13)-H(13C)	109.5	C(25)-Rh(1)-Cl(1)	168.03(5)
H(13B)-C(13)-H(13C)	109.5	C(22)-Rh(1)-Cl(1)	89.10(6)
C(18)-C(14)-H(14A)	109.5	C(29)-Rh(1)-Cl(1)	88.60(6)

Supplementary Table 13. Anisotropic displacement parameters (Å2x 103) for Rh(cod)(IMes)Cl. The anisotropic displacement factor exponent takes the form: -2 2[h2 a*2U11 + ... + 2 h k a* b* U12]

	**U11**	**U22**	**U33**	**U23**	**U13**	**U12**
C(1)	41(1)	39(1)	54(1)	-1(1)	2(1)	12(1)
C(2)	56(1)	56(1)	50(1)	-6(1)	-9(1)	-25(1)
C(3)	43(1)	32(1)	40(1)	-1(1)	-5(1)	7(1)
C(4)	33(1)	29(1)	27(1)	-2(1)	3(1)	2(1)
C(5)	51(1)	26(1)	34(1)	-5(1)	1(1)	-4(1)
C(6)	40(1)	40(1)	26(1)	-4(1)	-1(1)	-12(1)
C(7)	30(1)	44(1)	26(1)	0(1)	-4(1)	-2(1)
C(8)	31(1)	30(1)	20(1)	-2(1)	1(1)	1(1)
C(9)	26(1)	29(1)	17(1)	-3(1)	1(1)	-3(1)
C(10)	29(1)	46(1)	25(1)	-2(1)	7(1)	-5(1)
C(11)	26(1)	43(1)	30(1)	-1(1)	6(1)	-5(1)
C(12)	22(1)	21(1)	22(1)	1(1)	-1(1)	2(1)
C(13)	32(1)	36(1)	57(1)	1(1)	-4(1)	9(1)
C(14)	80(2)	102(2)	54(2)	-33(2)	-33(1)	12(2)
C(15)	36(1)	30(1)	56(1)	2(1)	-9(1)	3(1)
C(16)	25(1)	32(1)	39(1)	1(1)	-6(1)	2(1)
C(17)	36(1)	51(1)	42(1)	2(1)	-18(1)	5(1)
C(18)	44(1)	59(1)	42(1)	-13(1)	-17(1)	0(1)
C(19)	40(1)	41(1)	46(1)	-17(1)	-9(1)	2(1)
C(20)	26(1)	30(1)	38(1)	-3(1)	-5(1)	0(1)
C(21)	22(1)	30(1)	27(1)	-2(1)	-4(1)	-1(1)
C(22)	46(1)	59(1)	23(1)	-8(1)	10(1)	5(1)
C(23)	56(1)	80(2)	21(1)	-2(1)	-2(1)	-3(1)
C(24)	44(1)	68(1)	31(1)	14(1)	-7(1)	2(1)
C(25)	33(1)	39(1)	31(1)	10(1)	1(1)	8(1)
C(26)	42(1)	26(1)	30(1)	7(1)	3(1)	4(1)
C(27)	45(1)	37(1)	50(1)	18(1)	-2(1)	-6(1)

C(28)	40(1)	71(2)	40(1)	20(1)	8(1)	-9(1)
C(29)	35(1)	61(1)	30(1)	1(1)	14(1)	5(1)
Cl(1)	33(1)	27(1)	47(1)	-5(1)	-1(1)	7(1)
N(1)	24(1)	29(1)	20(1)	-2(1)	2(1)	-3(1)
N(2)	22(1)	28(1)	24(1)	-2(1)	0(1)	-2(1)
Rh(1)	24(1)	25(1)	19(1)	-1(1)	2(1)	2(1)

Supplementary Table 14. Hydrogen coordinates (x 104) and isotropic displacement parameters (Å2x 10 3) for Rh(cod)(IMes)Cl.

	x	y	z	U(eq)
H(1A)	2851	7591	7100	68
H(1B)	2913	7447	5610	68
H(1C)	3083	7874	6077	68
H(2A)	4670	7766	7437	81
H(2B)	4580	7627	8916	81
H(2C)	4353	8009	8316	81
H(3A)	4166	6264	7529	57
H(3B)	3863	6215	6316	57
H(3C)	3693	6226	7790	57
H(5)	3715	7918	7296	44
H(7)	4413	6930	7864	40
H(10)	2778	6681	7764	40
H(11)	2312	6318	6248	39
H(13A)	1890	6819	3923	63
H(13B)	2073	7056	2691	63
H(13C)	2335	7000	4000	63
H(14A)	1934	5637	-159	117
H(14B)	2390	5660	-656	117
H(14C)	2103	6043	-798	117
H(15A)	2766	5456	4743	60
H(15B)	3162	5714	4447	60
H(15C)	3046	5344	3515	60
H(17)	1993	6490	1132	52
H(19)	2629	5458	1621	51
H(22)	3730	6229	918	51
H(23A)	3095	6486	276	63
H(23B)	3406	6732	-611	63
H(24A)	3297	7316	483	57
H(24B)	2860	7118	572	57
H(25)	2897	7138	2813	41
H(26)	3412	7398	3958	39
H(27A)	4084	7477	3224	53
H(27B)	3821	7720	2173	53
H(28A)	3935	7266	542	60

H(28B)	4353	7238	1322	60
H(29)	4242	6575	1733	50

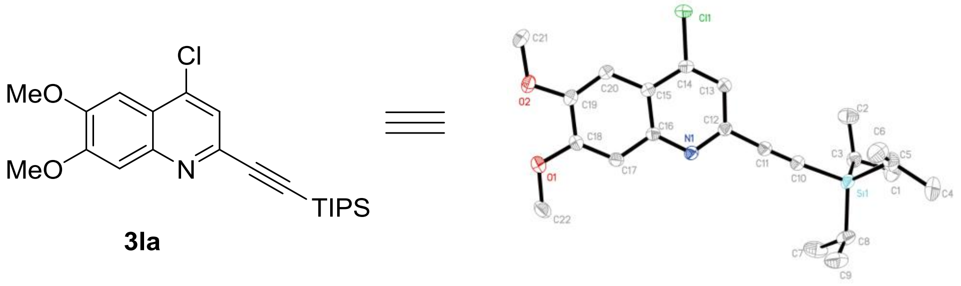

3la

Supplementary Table 15. Crystal data and structure refinement for 3la.

Identification code	3la
Empirical formula	C22 H30 Cl N O2 Si
Formula weight	404.01
Temperature	190(2) K
Wavelength	1.34139 Å
Crystal system	Triclinic
Space group	P-1
Unit cell dimensions	a = 8.9926(5) Å α = 87.566(2) °.
	b = 12.7888(7) Å β = 88.073(2) °.
	c = 20.1548(10) Å γ =77.676(2) °.
Volume	2261.7(2) Å^3
Z	4
Density (calculated)	1.186 Mg/m^3
Absorption coefficient	1.394 mm^{-1}
F(000)	864
Crystal size	0.120 x 0.110 x 0.090 mm^3
Theta range for data collection	3.079 to 53.829 °.
Index ranges	-10<=h<=10, -15<=k<=15, -24<=l<=24
Reflections collected	29902
Independent reflections	8213 [R(int) = 0.0411]
Completeness to theta = 53.594 °	99.7 %
Refinement method	Full-matrix least-squares on F^2
Data / restraints / parameters	8213 / 0 / 503
Goodness-of-fit on F^2	1.046

Final R indices [I>2sigma(I)]	R1 = 0.0432, wR2 = 0.1237
R indices (all data)	R1 = 0.0502, wR2 = 0.1322
Extinction coefficient	n/a
Largest diff. peak and hole	0.654 and -0.359 e.Å^{-3}

Supplementary Table 16. Atomic coordinates (x 10^4) and equivalent isotropic displacement parameters (Å^{2}x 10^3) for 3la. U(eq) is defined as one third of the trace of the orthogonalized U^{ij} tensor.

	x	y	z	U(eq)
Cl(1)	11418(1)	2360(1)	5565(1)	48(1)
Cl(2)	3722(1)	2674(1)	4188(1)	47(1)
Si(1)	6651(1)	629(1)	8540(1)	41(1)
Si(2)	9457(1)	4681(1)	1552(1)	41(1)
O(4)	7894(2)	4521(1)	6339(1)	45(1)
O(3)	5580(1)	3665(1)	6529(1)	44(1)
O(2)	9312(2)	1252(1)	3325(1)	48(1)
O(1)	7008(2)	410(1)	3587(1)	52(1)
N(1)	7376(2)	807(1)	5928(1)	36(1)
N(2)	7797(2)	4220(1)	3988(1)	36(1)
C(16)	7911(2)	953(1)	5300(1)	33(1)
C(38)	7181(2)	4050(1)	4595(1)	33(1)
C(34)	7164(2)	3912(1)	3463(1)	36(1)
C(42)	5340(2)	3428(1)	5356(1)	34(1)
C(15)	9180(2)	1430(1)	5148(1)	33(1)
C(12)	8087(2)	1134(1)	6420(1)	35(1)
C(40)	7312(2)	4240(1)	5778(1)	36(1)
C(35)	5897(2)	3427(1)	3506(1)	39(1)
C(37)	5904(2)	3566(1)	4698(1)	32(1)
C(41)	6019(2)	3753(1)	5882(1)	35(1)
C(19)	8934(2)	1191(1)	3979(1)	38(1)
C(20)	9668(2)	1540(1)	4481(1)	36(1)
C(13)	9345(2)	1625(1)	6327(1)	38(1)
C(11)	7536(2)	965(1)	7091(1)	38(1)
C(33)	7857(2)	4113(2)	2829(1)	40(1)
C(18)	7645(2)	716(1)	4130(1)	39(1)

C(39)	7858(2)	4378(1)	5152(1)	36(1)
C(36)	5293(2)	3264(1)	4121(1)	35(1)
C(10)	7140(2)	830(2)	7657(1)	41(1)
C(17)	7166(2)	604(1)	4769(1)	38(1)
C(32)	8499(2)	4302(2)	2318(1)	43(1)
C(44)	9228(2)	4963(2)	6245(1)	46(1)
C(14)	9866(2)	1762(1)	5698(1)	35(1)
C(43)	4289(2)	3195(2)	6665(1)	48(1)
C(3)	8283(2)	-387(2)	8898(1)	48(1)
C(26)	8470(2)	4245(2)	839(1)	48(1)
C(29)	9118(2)	6186(2)	1546(1)	49(1)
C(21)	10597(2)	1709(2)	3153(1)	51(1)
C(22)	5688(2)	-42(2)	3711(1)	54(1)
C(24)	11498(2)	3888(2)	1546(1)	57(1)
C(28)	6799(3)	4814(2)	803(1)	63(1)
C(8)	4844(2)	121(2)	8597(1)	59(1)
C(31)	9657(3)	6647(2)	2160(1)	65(1)
C(6)	6070(3)	2927(2)	8494(1)	69(1)
C(2)	9818(3)	-85(2)	8744(1)	68(1)
C(30)	9697(3)	6683(2)	908(1)	71(1)
C(5)	6497(3)	1958(2)	8953(1)	57(1)
C(4)	5483(3)	2055(2)	9587(1)	73(1)
C(27)	8611(3)	3032(2)	854(1)	74(1)
C(23)	12363(3)	4045(3)	900(1)	77(1)
C(1)	8061(3)	-608(2)	9643(1)	79(1)
C(25)	12428(3)	3990(3)	2128(1)	90(1)
C(7)	5049(3)	-992(2)	8306(2)	84(1)
C(9)	3537(3)	908(2)	8254(2)	76(1)

Supplementary Table 17. Bond lengths [Å] and angles [°] for 3la.

Cl(1)-C(14)	1.7370(17)	C(42)-C(41)-C(40)	120.35(16)
Cl(2)-C(36)	1.7373(16)	O(2)-C(19)-C(20)	125.35(16)
Si(1)-C(10)	1.839(2)	O(2)-C(19)-C(18)	114.80(16)
Si(1)-C(8)	1.873(2)	C(20)-C(19)-C(18)	119.84(16)
Si(1)-C(3)	1.881(2)	C(19)-C(20)-C(15)	120.54(16)
Si(1)-C(5)	1.900(2)	C(19)-C(20)-H(20)	119.7
Si(2)-C(32)	1.8384(19)	C(15)-C(20)-H(20)	119.7
Si(2)-C(26)	1.879(2)	C(14)-C(13)-C(12)	118.05(16)

Si(2)-C(29)	1.883(2)	C(14)-C(13)-H(13)	121.0
Si(2)-C(24)	1.898(2)	C(12)-C(13)-H(13)	121.0
O(4)-C(40)	1.355(2)	C(10)-C(11)-C(12)	177.15(19)
O(4)-C(44)	1.435(2)	C(32)-C(33)-C(34)	176.30(19)
O(3)-C(41)	1.356(2)	C(17)-C(18)-O(1)	125.66(17)
O(3)-C(43)	1.430(2)	C(17)-C(18)-C(19)	120.17(16)
O(2)-C(19)	1.354(2)	O(1)-C(18)-C(19)	114.16(16)
O(2)-C(21)	1.429(2)	C(40)-C(39)-C(38)	121.26(15)
O(1)-C(18)	1.361(2)	C(40)-C(39)-H(39)	119.4
O(1)-C(22)	1.438(2)	C(38)-C(39)-H(39)	119.4
N(1)-C(12)	1.326(2)	C(35)-C(36)-C(37)	121.68(15)
N(1)-C(16)	1.359(2)	C(35)-C(36)-Cl(2)	118.82(14)
N(2)-C(34)	1.331(2)	C(37)-C(36)-Cl(2)	119.49(13)
N(2)-C(38)	1.356(2)	C(11)-C(10)-Si(1)	176.44(17)
C(16)-C(17)	1.418(2)	C(18)-C(17)-C(16)	121.19(16)
C(16)-C(15)	1.423(2)	C(18)-C(17)-H(17)	119.4
C(38)-C(39)	1.414(2)	C(16)-C(17)-H(17)	119.4
C(38)-C(37)	1.422(2)	C(33)-C(32)-Si(2)	176.33(18)
C(34)-C(35)	1.408(2)	O(4)-C(44)-H(44A)	109.5
C(34)-C(33)	1.440(3)	O(4)-C(44)-H(44B)	109.5
C(42)-C(41)	1.364(2)	H(44A)-C(44)-H(44B)	109.5
C(42)-C(37)	1.420(2)	O(4)-C(44)-H(44C)	109.5
C(42)-H(42)	0.9500	H(44A)-C(44)-H(44C)	109.5
C(15)-C(20)	1.409(2)	H(44B)-C(44)-H(44C)	109.5
C(15)-C(14)	1.411(2)	C(13)-C(14)-C(15)	121.56(15)
C(12)-C(13)	1.410(2)	C(13)-C(14)-Cl(1)	119.31(13)
C(12)-C(11)	1.448(2)	C(15)-C(14)-Cl(1)	119.13(13)
C(40)-C(39)	1.357(2)	O(3)-C(43)-H(43A)	109.5
C(40)-C(41)	1.437(2)	O(3)-C(43)-H(43B)	109.5
C(35)-C(36)	1.362(3)	H(43A)-C(43)-H(43B)	109.5
C(35)-H(35)	0.9500	O(3)-C(43)-H(43C)	109.5
C(37)-C(36)	1.406(2)	H(43A)-C(43)-H(43C)	109.5
C(19)-C(20)	1.367(3)	H(43B)-C(43)-H(43C)	109.5
C(19)-C(18)	1.437(3)	C(2)-C(3)-C(1)	111.3(2)
C(20)-H(20)	0.9500	C(2)-C(3)-Si(1)	112.16(14)
C(13)-C(14)	1.354(3)	C(1)-C(3)-Si(1)	112.44(16)
C(13)-H(13)	0.9500	C(2)-C(3)-H(3)	106.9
C(11)-C(10)	1.200(3)	C(1)-C(3)-H(3)	106.9

C(33)-C(32)	1.206(3)	Si(1)-C(3)-H(3)	106.9
C(18)-C(17)	1.356(3)	C(28)-C(26)-C(27)	110.1(2)
C(39)-H(39)	0.9500	C(28)-C(26)-Si(2)	112.67(15)
C(17)-H(17)	0.9500	C(27)-C(26)-Si(2)	111.98(15)
C(44)-H(44A)	0.9800	C(28)-C(26)-H(26)	107.3
C(44)-H(44B)	0.9800	C(27)-C(26)-H(26)	107.3
C(44)-H(44C)	0.9800	Si(2)-C(26)-H(26)	107.3
C(43)-H(43A)	0.9800	C(31)-C(29)-C(30)	110.72(19)
C(43)-H(43B)	0.9800	C(31)-C(29)-Si(2)	115.05(15)
C(43)-H(43C)	0.9800	C(30)-C(29)-Si(2)	113.69(15)
C(3)-C(2)	1.529(3)	C(31)-C(29)-H(29)	105.5
C(3)-C(1)	1.531(3)	C(30)-C(29)-H(29)	105.5
C(3)-H(3)	1.0000	Si(2)-C(29)-H(29)	105.5
C(26)-C(28)	1.526(3)	O(2)-C(21)-H(21A)	109.5
C(26)-C(27)	1.529(3)	O(2)-C(21)-H(21B)	109.5
C(26)-H(26)	1.0000	H(21A)-C(21)-H(21B)	109.5
C(29)-C(31)	1.529(3)	O(2)-C(21)-H(21C)	109.5
C(29)-C(30)	1.533(3)	H(21A)-C(21)-H(21C)	109.5
C(29)-H(29)	1.0000	H(21B)-C(21)-H(21C)	109.5
C(21)-H(21A)	0.9800	O(1)-C(22)-H(22A)	109.5
C(21)-H(21B)	0.9800	O(1)-C(22)-H(22B)	109.5
C(21)-H(21C)	0.9800	H(22A)-C(22)-H(22B)	109.5
C(22)-H(22A)	0.9800	O(1)-C(22)-H(22C)	109.5
C(22)-H(22B)	0.9800	H(22A)-C(22)-H(22C)	109.5
C(22)-H(22C)	0.9800	H(22B)-C(22)-H(22C)	109.5
C(24)-C(25)	1.488(3)	C(25)-C(24)-C(23)	110.6(2)
C(24)-C(23)	1.522(3)	C(25)-C(24)-Si(2)	116.98(17)
C(24)-H(24)	1.0000	C(23)-C(24)-Si(2)	112.92(16)
C(28)-H(28A)	0.9800	C(25)-C(24)-H(24)	105.1
C(28)-H(28B)	0.9800	C(23)-C(24)-H(24)	105.1
C(28)-H(28C)	0.9800	Si(2)-C(24)-H(24)	105.1
C(8)-C(7)	1.535(4)	C(26)-C(28)-H(28A)	109.5
C(8)-C(9)	1.538(3)	C(26)-C(28)-H(28B)	109.5
C(8)-H(8)	1.0000	H(28A)-C(28)-H(28B)	109.5
C(31)-H(31A)	0.9800	C(26)-C(28)-H(28C)	109.5
C(31)-H(31B)	0.9800	H(28A)-C(28)-H(28C)	109.5
C(31)-H(31C)	0.9800	H(28B)-C(28)-H(28C)	109.5
C(6)-C(5)	1.505(3)	C(7)-C(8)-C(9)	110.1(2)

C(6)-H(6A)	0.9800	C(7)-C(8)-Si(1)	111.83(16)
C(6)-H(6B)	0.9800	C(9)-C(8)-Si(1)	111.31(16)
C(6)-H(6C)	0.9800	C(7)-C(8)-H(8)	107.8
C(2)-H(2A)	0.9800	C(9)-C(8)-H(8)	107.8
C(2)-H(2B)	0.9800	Si(1)-C(8)-H(8)	107.8
C(2)-H(2C)	0.9800	C(29)-C(31)-H(31A)	109.5
C(30)-H(30A)	0.9800	C(29)-C(31)-H(31B)	109.5
C(30)-H(30B)	0.9800	H(31A)-C(31)-H(31B)	109.5
C(30)-H(30C)	0.9800	C(29)-C(31)-H(31C)	109.5
C(5)-C(4)	1.538(3)	H(31A)-C(31)-H(31C)	109.5
C(5)-H(5)	1.0000	H(31B)-C(31)-H(31C)	109.5
C(4)-H(4A)	0.9800	C(5)-C(6)-H(6A)	109.5
C(4)-H(4B)	0.9800	C(5)-C(6)-H(6B)	109.5
C(4)-H(4C)	0.9800	H(6A)-C(6)-H(6B)	109.5
C(27)-H(27A)	0.9800	C(5)-C(6)-H(6C)	109.5
C(27)-H(27B)	0.9800	H(6A)-C(6)-H(6C)	109.5
C(27)-H(27C)	0.9800	H(6B)-C(6)-H(6C)	109.5
C(23)-H(23A)	0.9800	C(3)-C(2)-H(2A)	109.5
C(23)-H(23B)	0.9800	C(3)-C(2)-H(2B)	109.5
C(23)-H(23C)	0.9800	H(2A)-C(2)-H(2B)	109.5
C(1)-H(1A)	0.9800	C(3)-C(2)-H(2C)	109.5
C(1)-H(1B)	0.9800	H(2A)-C(2)-H(2C)	109.5
C(1)-H(1C)	0.9800	H(2B)-C(2)-H(2C)	109.5
C(25)-H(25A)	0.9800	C(29)-C(30)-H(30A)	109.5
C(25)-H(25B)	0.9800	C(29)-C(30)-H(30B)	109.5
C(25)-H(25C)	0.9800	H(30A)-C(30)-H(30B)	109.5
C(7)-H(7A)	0.9800	C(29)-C(30)-H(30C)	109.5
C(7)-H(7B)	0.9800	H(30A)-C(30)-H(30C)	109.5
C(7)-H(7C)	0.9800	H(30B)-C(30)-H(30C)	109.5
C(9)-H(9A)	0.9800	C(6)-C(5)-C(4)	111.7(2)
C(9)-H(9B)	0.9800	C(6)-C(5)-Si(1)	114.48(17)
C(9)-H(9C)	0.9800	C(4)-C(5)-Si(1)	112.82(17)
C(10)-Si(1)-C(8)	108.37(10)	C(6)-C(5)-H(5)	105.7
C(10)-Si(1)-C(3)	106.50(9)	C(4)-C(5)-H(5)	105.7
C(8)-Si(1)-C(3)	111.12(10)	Si(1)-C(5)-H(5)	105.7
C(10)-Si(1)-C(5)	107.56(9)	C(5)-C(4)-H(4A)	109.5
C(8)-Si(1)-C(5)	113.40(11)	C(5)-C(4)-H(4B)	109.5
C(3)-Si(1)-C(5)	109.59(10)	H(4A)-C(4)-H(4B)	109.5

C(32)-Si(2)-C(26)	106.82(9)	C(5)-C(4)-H(4C)	109.5
C(32)-Si(2)-C(29)	105.09(9)	H(4A)-C(4)-H(4C)	109.5
C(26)-Si(2)-C(29)	110.07(9)	H(4B)-C(4)-H(4C)	109.5
C(32)-Si(2)-C(24)	108.06(9)	C(26)-C(27)-H(27A)	109.5
C(26)-Si(2)-C(24)	107.96(10)	C(26)-C(27)-H(27B)	109.5
C(29)-Si(2)-C(24)	118.26(11)	H(27A)-C(27)-H(27B)	109.5
C(40)-O(4)-C(44)	115.60(14)	C(26)-C(27)-H(27C)	109.5
C(41)-O(3)-C(43)	116.77(14)	H(27A)-C(27)-H(27C)	109.5
C(19)-O(2)-C(21)	116.71(15)	H(27B)-C(27)-H(27C)	109.5
C(18)-O(1)-C(22)	116.26(15)	C(24)-C(23)-H(23A)	109.5
C(12)-N(1)-C(16)	117.33(14)	C(24)-C(23)-H(23B)	109.5
C(34)-N(2)-C(38)	117.59(14)	H(23A)-C(23)-H(23B)	109.5
N(1)-C(16)-C(17)	117.97(15)	C(24)-C(23)-H(23C)	109.5
N(1)-C(16)-C(15)	123.63(16)	H(23A)-C(23)-H(23C)	109.5
C(17)-C(16)-C(15)	118.40(16)	H(23B)-C(23)-H(23C)	109.5
N(2)-C(38)-C(39)	117.37(15)	C(3)-C(1)-H(1A)	109.5
N(2)-C(38)-C(37)	123.60(16)	C(3)-C(1)-H(1B)	109.5
C(39)-C(38)-C(37)	119.02(15)	H(1A)-C(1)-H(1B)	109.5
N(2)-C(34)-C(35)	123.67(16)	C(3)-C(1)-H(1C)	109.5
N(2)-C(34)-C(33)	115.45(15)	H(1A)-C(1)-H(1C)	109.5
C(35)-C(34)-C(33)	120.88(16)	H(1B)-C(1)-H(1C)	109.5
C(41)-C(42)-C(37)	120.43(15)	C(24)-C(25)-H(25A)	109.5
C(41)-C(42)-H(42)	119.8	C(24)-C(25)-H(25B)	109.5
C(37)-C(42)-H(42)	119.8	H(25A)-C(25)-H(25B)	109.5
C(20)-C(15)-C(14)	124.61(15)	C(24)-C(25)-H(25C)	109.5
C(20)-C(15)-C(16)	119.85(16)	H(25A)-C(25)-H(25C)	109.5
C(14)-C(15)-C(16)	115.54(16)	H(25B)-C(25)-H(25C)	109.5
N(1)-C(12)-C(13)	123.89(16)	C(8)-C(7)-H(7A)	109.5
N(1)-C(12)-C(11)	117.55(15)	C(8)-C(7)-H(7B)	109.5
C(13)-C(12)-C(11)	118.56(16)	H(7A)-C(7)-H(7B)	109.5
O(4)-C(40)-C(39)	125.28(16)	C(8)-C(7)-H(7C)	109.5
O(4)-C(40)-C(41)	114.96(16)	H(7A)-C(7)-H(7C)	109.5
C(39)-C(40)-C(41)	119.76(16)	H(7B)-C(7)-H(7C)	109.5
C(36)-C(35)-C(34)	117.85(16)	C(8)-C(9)-H(9A)	109.5
C(36)-C(35)-H(35)	121.1	C(8)-C(9)-H(9B)	109.5
C(34)-C(35)-H(35)	121.1	H(9A)-C(9)-H(9B)	109.5
C(36)-C(37)-C(42)	125.21(15)	C(8)-C(9)-H(9C)	109.5
C(36)-C(37)-C(38)	115.61(15)	H(9A)-C(9)-H(9C)	109.5

C(42)-C(37)-C(38)	119.17(15)	H(9B)-C(9)-H(9C)	109.5
O(3)-C(41)-C(42)	125.58(15)		
O(3)-C(41)-C(40)	114.07(15)		

Supplementary Table 18. Anisotropic displacement parameters ($Å^2$x 10^3) for 3la. The anisotropic displacement factor exponent takes the form: -2 ^{2}[h^2 $a^{*2}U^{11}$ + ... + 2 h k a* b* U^{12}]

	U^{11}	U^{22}	U^{33}	U^{23}	U^{13}	U^{12}
Cl(1)	41(1)	58(1)	51(1)	-4(1)	2(1)	-25(1)
Cl(2)	40(1)	58(1)	49(1)	-3(1)	-1(1)	-25(1)
Si(1)	46(1)	44(1)	33(1)	1(1)	6(1)	-12(1)
Si(2)	43(1)	50(1)	30(1)	5(1)	1(1)	-11(1)
O(4)	45(1)	61(1)	34(1)	-6(1)	-2(1)	-22(1)
O(3)	45(1)	56(1)	34(1)	-2(1)	5(1)	-19(1)
O(2)	56(1)	56(1)	32(1)	1(1)	4(1)	-15(1)
O(1)	56(1)	66(1)	39(1)	-6(1)	-7(1)	-23(1)
N(1)	35(1)	39(1)	33(1)	2(1)	0(1)	-10(1)
N(2)	34(1)	43(1)	33(1)	5(1)	-3(1)	-12(1)
C(16)	32(1)	31(1)	35(1)	1(1)	-1(1)	-7(1)
C(38)	32(1)	33(1)	34(1)	5(1)	-2(1)	-8(1)
C(34)	36(1)	40(1)	33(1)	4(1)	-2(1)	-9(1)
C(42)	31(1)	34(1)	38(1)	1(1)	2(1)	-9(1)
C(15)	32(1)	30(1)	35(1)	1(1)	0(1)	-5(1)
C(12)	34(1)	36(1)	33(1)	2(1)	0(1)	-7(1)
C(40)	36(1)	38(1)	35(1)	-1(1)	-4(1)	-8(1)
C(35)	37(1)	45(1)	35(1)	1(1)	-5(1)	-13(1)
C(37)	30(1)	31(1)	35(1)	2(1)	-2(1)	-6(1)
C(41)	37(1)	35(1)	34(1)	1(1)	2(1)	-7(1)
C(19)	42(1)	37(1)	34(1)	2(1)	3(1)	-6(1)
C(20)	35(1)	36(1)	36(1)	2(1)	3(1)	-9(1)
C(13)	37(1)	42(1)	36(1)	-4(1)	-3(1)	-10(1)
C(11)	38(1)	41(1)	37(1)	0(1)	-1(1)	-10(1)
C(33)	39(1)	46(1)	35(1)	5(1)	-5(1)	-13(1)
C(18)	42(1)	40(1)	35(1)	-2(1)	-6(1)	-9(1)
C(39)	32(1)	41(1)	38(1)	1(1)	-3(1)	-13(1)

C(36)	30(1)	36(1)	40(1)	1(1)	-2(1)	-9(1)
C(10)	42(1)	44(1)	38(1)	0(1)	2(1)	-11(1)
C(17)	36(1)	40(1)	40(1)	1(1)	-2(1)	-12(1)
C(32)	44(1)	49(1)	37(1)	4(1)	-3(1)	-13(1)
C(44)	42(1)	54(1)	46(1)	-7(1)	-6(1)	-18(1)
C(14)	30(1)	35(1)	40(1)	-1(1)	-1(1)	-9(1)
C(43)	49(1)	57(1)	42(1)	3(1)	9(1)	-21(1)
C(3)	57(1)	48(1)	41(1)	4(1)	-2(1)	-12(1)
C(26)	54(1)	52(1)	36(1)	0(1)	-1(1)	-8(1)
C(29)	56(1)	53(1)	39(1)	4(1)	1(1)	-16(1)
C(21)	59(1)	54(1)	40(1)	4(1)	12(1)	-14(1)
C(22)	58(1)	59(1)	52(1)	-7(1)	-13(1)	-23(1)
C(24)	51(1)	72(1)	43(1)	5(1)	2(1)	-7(1)
C(28)	56(1)	75(2)	58(1)	-4(1)	-13(1)	-11(1)
C(8)	51(1)	66(1)	62(1)	6(1)	11(1)	-22(1)
C(31)	85(2)	61(1)	51(1)	-3(1)	-10(1)	-23(1)
C(6)	74(2)	53(1)	81(2)	-9(1)	19(1)	-17(1)
C(2)	52(1)	69(2)	85(2)	11(1)	-14(1)	-13(1)
C(30)	107(2)	59(1)	49(1)	9(1)	11(1)	-29(1)
C(5)	63(1)	56(1)	53(1)	-10(1)	4(1)	-12(1)
C(4)	93(2)	74(2)	49(1)	-18(1)	12(1)	-8(1)
C(27)	90(2)	59(1)	72(2)	-7(1)	-16(1)	-15(1)
C(23)	52(1)	116(2)	52(1)	13(1)	11(1)	0(1)
C(1)	94(2)	87(2)	47(1)	16(1)	-3(1)	0(2)
C(25)	58(2)	133(3)	64(2)	-10(2)	-10(1)	14(2)
C(7)	74(2)	67(2)	120(3)	4(2)	-10(2)	-36(1)
C(9)	45(1)	83(2)	101(2)	-4(2)	2(1)	-17(1)

Supplementary Table 19. Hydrogen coordinates (x 10^4) and isotropic displacement parameters (Å^{2}x 10 ^{3}) for 3la.

	x	y	z	U(eq)
H(42)	4486	3108	5429	41
H(35)	5476	3221	3118	46
H(20)	10515	1859	4380	43
H(13)	9814	1852	6695	45
H(39)	8711	4702	5086	44
H(17)	6316	286	4863	45

H(44A)	10035	4445	6025	69
H(44B)	9575	5124	6677	69
H(44C)	8985	5623	5968	69
H(43A)	3406	3616	6433	73
H(43B)	4067	3187	7144	73
H(43C)	4510	2460	6511	73
H(3)	8309	-1074	8674	58
H(26)	8991	4442	421	57
H(29)	7986	6442	1550	59
H(21A)	10416	2441	3311	77
H(21B)	10751	1724	2669	77
H(21C)	11507	1276	3360	77
H(22A)	5959	-691	3996	81
H(22B)	5316	-224	3288	81
H(22C)	4889	480	3933	81
H(24)	11416	3120	1560	68
H(28A)	6343	4568	420	94
H(28B)	6726	5589	755	94
H(28C)	6257	4650	1211	94
H(8)	4553	57	9078	70
H(31A)	10766	6423	2184	97
H(31B)	9181	6384	2559	97
H(31C)	9368	7431	2129	97
H(6A)	5029	2987	8345	104
H(6B)	6773	2856	8109	104
H(6C)	6132	3570	8730	104
H(2A)	9833	594	8949	103
H(2B)	9966	-7	8262	103
H(2C)	10637	-648	8924	103
H(30A)	9412	7465	922	106
H(30B)	9241	6444	523	106
H(30C)	10808	6456	873	106
H(5)	7545	1959	9103	69
H(4A)	5502	2729	9797	110
H(4B)	5866	1453	9896	110
H(4C)	4436	2045	9472	110
H(27A)	8102	2810	1257	110
H(27B)	9689	2674	852	110

H(27C)	8131	2833	463	110
H(23A)	12464	4792	849	116
H(23B)	11804	3869	526	116
H(23C)	13376	3573	909	116
H(1A)	8905	-1171	9800	119
H(1B)	7098	-841	9723	119
H(1C)	8036	48	9882	119
H(25A)	13431	3511	2083	134
H(25B)	11915	3794	2537	134
H(25C)	12551	4731	2147	134
H(7A)	4067	-1210	8318	126
H(7B)	5781	-1510	8569	126
H(7C)	5428	-966	7845	126
H(9A)	3821	1021	7786	114
H(9B)	3341	1593	8475	114
H(9C)	2616	612	8280	114

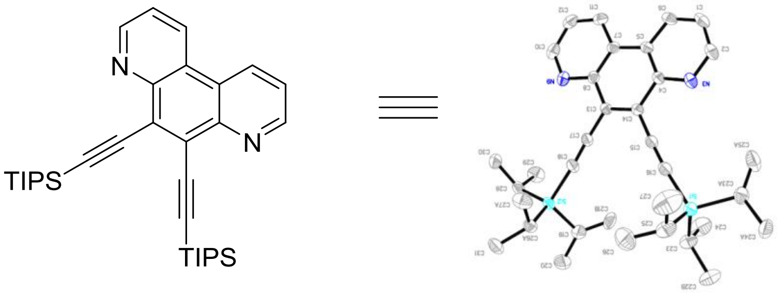

7ca'

Supplementary Table 20. Crystal data and structure refinement for 7ca'.

Identification code	**7ca'**
Empirical formula	C34 H48 N2 Si2
Formula weight	540.92
Temperature	296(2) K
Wavelength	0.71073 Å
Crystal system	Triclinic
Space group	P-1
Unit cell dimensions	a = 8.190(3) Å
	b = 11.886(4) Å
	c = 17.490(6) Å
Volume	1649.0(9) Å3
Z	2
Density (calculated)	1.089 Mg/m3
Absorption coefficient	0.131 mm-1

F(000)	588
Crystal size	0.060 x 0.050 x 0.050 mm3
Theta range for data collection	2.320 to 26.371 °.
Index ranges	-10<=h<=10, -14<=k<=14, -18<=l<=21
Reflections collected	10675
Independent reflections	6604 [R(int) = 0.0442]
Completeness to theta = 25.242 °	97.8 %
Refinement method	Full-matrix least-squares on F2
Data / restraints / parameters	6604 / 164 / 428
Goodness-of-fit on F2	0.982
Final R indices [I>2sigma(I)]	R1 = 0.0682, wR2 = 0.1721
R indices (all data)	R1 = 0.1110, wR2 = 0.2004
Extinction coefficient	n/a
Largest diff. peak and hole	0.567 and -0.304 e.Å-3

Supplementary Table 21. Atomic coordinates (x 104) and equivalent isotropic displacement parameters (Å2x 103) for 7ca'.U(eq) is defined as one third of the trace of the orthogonalized Uij tensor.

	**x**	**y**	**z**	**U(eq)**
Si(1)	7611(1)	2826(1)	8590(1)	54(1)
Si(2)	4468(1)	7793(1)	7297(1)	45(1)
C(1)	13589(4)	3626(3)	5234(2)	55(1)
C(2)	12852(4)	3105(3)	5960(2)	54(1)
N(3)	11636(3)	3600(2)	6359(2)	47(1)
C(4)	11119(3)	4698(2)	6037(2)	38(1)
C(5)	11822(3)	5315(2)	5322(2)	39(1)
C(6)	13089(4)	4735(3)	4911(2)	49(1)
C(7)	11217(4)	6495(2)	5035(2)	40(1)
C(8)	9891(4)	6988(2)	5465(2)	39(1)
N(9)	9266(3)	8097(2)	5235(2)	51(1)
C(10)	9953(5)	8700(3)	4578(2)	60(1)
C(11)	11883(4)	7186(3)	4336(2)	52(1)
C(12)	11243(5)	8290(3)	4111(2)	62(1)
C(13)	9112(3)	6314(2)	6169(2)	36(1)
C(14)	9717(3)	5205(2)	6456(2)	36(1)
C(15)	8967(4)	4508(2)	7152(2)	43(1)
C(16)	8382(4)	3884(3)	7733(2)	53(1)
C(17)	7648(4)	6790(2)	6549(2)	41(1)
C(18)	6370(4)	7149(3)	6855(2)	46(1)
C(19)	2766(5)	6770(3)	7378(3)	78(1)
C(20)	1134(5)	7211(4)	7724(3)	86(1)
C(23)	5345(5)	2791(3)	8543(2)	67(1)
C(24)	4878(7)	2555(4)	7782(3)	98(2)
C(25)	8005(6)	3331(4)	9489(2)	74(1)

C(26)	7156(8)	4512(5)	9505(3)	115(2)
C(27)	9841(7)	3327(6)	9588(4)	130(2)
C(28)	3941(4)	9157(3)	6587(2)	51(1)
C(29)	3540(5)	8914(4)	5809(2)	75(1)
C(30)	5353(6)	9967(3)	6432(2)	74(1)
C(31)	3746(5)	8970(3)	8563(2)	68(1)
C(21B)	3440(30)	5770(20)	7200(20)	71(6)
C(22B)	4474(17)	1890(20)	9214(8)	85(4)
C(23A)	8462(13)	1347(6)	8417(6)	60(2)
C(24A)	8160(20)	423(17)	9249(15)	72(4)
C(25A)	9640(30)	1178(12)	7856(8)	83(4)
C(26A)	4807(7)	7960(5)	8289(3)	48(1)
C(27A)	6618(8)	8105(7)	8371(4)	73(2)
C(21A)	3189(8)	5563(4)	7774(6)	81(2)
C(22A)	4490(40)	2390(40)	9329(18)	83(6)
C(23B)	9140(20)	1483(10)	8668(7)	60(3)
C(24B)	8750(30)	498(18)	9226(15)	93(5)
C(25B)	10277(16)	1394(12)	8050(9)	75(3)
C(26B)	5440(30)	8480(20)	8154(11)	56(3)
C(27B)	6350(30)	7562(19)	8706(14)	68(4)

Supplementary Table 22. Bond lengths [Å] and angles [°] for 7ca'.

Si(1)-C(16)	1.831(4)	C(13)-C(14)-C(15)	121.9(3)
Si(1)-C(25)	1.867(4)	C(13)-C(14)-C(4)	120.2(3)
Si(1)-C(23)	1.871(4)	C(15)-C(14)-C(4)	117.9(2)
Si(1)-C(23A)	1.905(7)	C(16)-C(15)-C(14)	177.3(3)
Si(1)-C(23B)	1.932(11)	C(15)-C(16)-Si(1)	174.8(3)
Si(2)-C(18)	1.837(3)	C(18)-C(17)-C(13)	176.5(3)
Si(2)-C(26A)	1.840(5)	C(17)-C(18)-Si(2)	176.2(3)
Si(2)-C(28)	1.871(3)	C(21B)-C(19)-C(20)	137.3(12)
Si(2)-C(19)	1.904(4)	C(21A)-C(19)-C(20)	111.1(5)
Si(2)-C(26B)	2.108(19)	C(21B)-C(19)-Si(2)	108.7(11)
C(1)-C(6)	1.365(5)	C(21A)-C(19)-Si(2)	114.5(4)
C(1)-C(2)	1.384(5)	C(20)-C(19)-Si(2)	113.3(3)
C(1)-H(1)	0.9300	C(21B)-C(19)-H(19)	92.7
C(2)-N(3)	1.317(4)	C(20)-C(19)-H(19)	92.7
C(2)-H(2)	0.9300	Si(2)-C(19)-H(19)	92.7
N(3)-C(4)	1.357(4)	C(21A)-C(19)-H(19A)	105.7
C(4)-C(5)	1.398(4)	C(20)-C(19)-H(19A)	105.7
C(4)-C(14)	1.446(4)	Si(2)-C(19)-H(19A)	105.7
C(5)-C(6)	1.408(4)	C(19)-C(20)-H(20A)	109.5
C(5)-C(7)	1.448(4)	C(19)-C(20)-H(20B)	109.5
C(6)-H(6)	0.9300	H(20A)-C(20)-H(20B)	109.5
C(7)-C(11)	1.402(4)	C(19)-C(20)-H(20C)	109.5

C(7)-C(8)	1.406(4)	H(20A)-C(20)-H(20C)	109.5
C(8)-N(9)	1.364(4)	H(20B)-C(20)-H(20C)	109.5
C(8)-C(13)	1.434(4)	C(22A)-C(23)-C(24)	125.5(16)
N(9)-C(10)	1.314(4)	C(24)-C(23)-C(22B)	105.8(9)
C(10)-C(12)	1.379(5)	C(22A)-C(23)-Si(1)	111.2(12)
C(10)-H(10)	0.9300	C(24)-C(23)-Si(1)	114.7(3)
C(11)-C(12)	1.363(5)	C(22B)-C(23)-Si(1)	114.5(6)
C(11)-H(11)	0.9300	C(24)-C(23)-H(23)	107.1
C(12)-H(12)	0.9300	C(22B)-C(23)-H(23)	107.1
C(13)-C(14)	1.376(4)	Si(1)-C(23)-H(23)	107.1
C(13)-C(17)	1.435(4)	C(22A)-C(23)-H(23A)	99.8
C(14)-C(15)	1.427(4)	C(24)-C(23)-H(23A)	99.8
C(15)-C(16)	1.202(4)	Si(1)-C(23)-H(23A)	99.8
C(17)-C(18)	1.205(4)	C(23)-C(24)-H(24A)	109.5
C(19)-C(21B)	1.34(2)	C(23)-C(24)-H(24B)	109.5
C(19)-C(21A)	1.484(7)	H(24A)-C(24)-H(24B)	109.5
C(19)-C(20)	1.500(5)	C(23)-C(24)-H(24C)	109.5
C(19)-H(19)	0.9800	H(24A)-C(24)-H(24C)	109.5
C(19)-H(19A)	0.9800	H(24B)-C(24)-H(24C)	109.5
C(20)-H(20A)	0.9600	C(26)-C(25)-C(27)	110.6(4)
C(20)-H(20B)	0.9600	C(26)-C(25)-Si(1)	111.0(3)
C(20)-H(20C)	0.9600	C(27)-C(25)-Si(1)	113.3(4)
C(23)-C(22A)	1.47(3)	C(26)-C(25)-H(25)	107.2
C(23)-C(24)	1.513(5)	C(27)-C(25)-H(25)	107.2
C(23)-C(22B)	1.564(14)	Si(1)-C(25)-H(25)	107.2
C(23)-H(23)	0.9800	C(25)-C(26)-H(26A)	109.5
C(23)-H(23A)	0.9800	C(25)-C(26)-H(26B)	109.5
C(24)-H(24A)	0.9600	H(26A)-C(26)-H(26B)	109.5
C(24)-H(24B)	0.9600	C(25)-C(26)-H(26C)	109.5
C(24)-H(24C)	0.9600	H(26A)-C(26)-H(26C)	109.5
C(25)-C(26)	1.515(6)	H(26B)-C(26)-H(26C)	109.5
C(25)-C(27)	1.532(7)	C(25)-C(27)-H(27A)	109.5
C(25)-H(25)	0.9800	C(25)-C(27)-H(27B)	109.5
C(26)-H(26A)	0.9600	H(27A)-C(27)-H(27B)	109.5
C(26)-H(26B)	0.9600	C(25)-C(27)-H(27C)	109.5
C(26)-H(26C)	0.9600	H(27A)-C(27)-H(27C)	109.5
C(27)-H(27A)	0.9600	H(27B)-C(27)-H(27C)	109.5
C(27)-H(27B)	0.9600	C(29)-C(28)-C(30)	109.4(3)
C(27)-H(27C)	0.9600	C(29)-C(28)-Si(2)	111.6(2)
C(28)-C(29)	1.525(5)	C(30)-C(28)-Si(2)	111.0(2)
C(28)-C(30)	1.534(5)	C(29)-C(28)-H(28)	108.2
C(28)-H(28)	0.9800	C(30)-C(28)-H(28)	108.2
C(29)-H(29A)	0.9600	Si(2)-C(28)-H(28)	108.2
C(29)-H(29B)	0.9600	C(28)-C(29)-H(29A)	109.5

C(29)-H(29C)	0.9600	C(28)-C(29)-H(29B)	109.5
C(30)-H(30A)	0.9600	H(29A)-C(29)-H(29B)	109.5
C(30)-H(30B)	0.9600	C(28)-C(29)-H(29C)	109.5
C(30)-H(30C)	0.9600	H(29A)-C(29)-H(29C)	109.5
C(31)-C(26A)	1.547(5)	H(29B)-C(29)-H(29C)	109.5
C(31)-C(26B)	1.607(19)	C(28)-C(30)-H(30A)	109.5
C(31)-H(31A)	0.9600	C(28)-C(30)-H(30B)	109.5
C(31)-H(31B)	0.9600	H(30A)-C(30)-H(30B)	109.5
C(31)-H(31C)	0.9600	C(28)-C(30)-H(30C)	109.5
C(31)-H(31D)	0.9600	H(30A)-C(30)-H(30C)	109.5
C(31)-H(31E)	0.9600	H(30B)-C(30)-H(30C)	109.5
C(31)-H(31F)	0.9600	C(26A)-C(31)-H(31A)	109.5
C(21B)-H(21A)	0.9600	C(26A)-C(31)-H(31B)	109.5
C(21B)-H(21B)	0.9600	H(31A)-C(31)-H(31B)	109.5
C(21B)-H(21C)	0.9600	C(26A)-C(31)-H(31C)	109.5
C(22B)-H(22A)	0.9600	H(31A)-C(31)-H(31C)	109.5
C(22B)-H(22B)	0.9600	H(31B)-C(31)-H(31C)	109.5
C(22B)-H(22C)	0.9600	C(26B)-C(31)-H(31D)	109.5
C(23A)-C(25A)	1.332(12)	C(26B)-C(31)-H(31E)	109.5
C(23A)-C(24A)	1.63(2)	H(31D)-C(31)-H(31E)	109.5
C(23A)-H(23B)	0.9800	C(26B)-C(31)-H(31F)	109.5
C(24A)-H(24D)	0.9600	H(31D)-C(31)-H(31F)	109.5
C(24A)-H(24E)	0.9600	H(31E)-C(31)-H(31F)	109.5
C(24A)-H(24F)	0.9600	C(19)-C(21B)-H(21A)	109.5
C(25A)-H(25A)	0.9600	C(19)-C(21B)-H(21B)	109.5
C(25A)-H(25B)	0.9600	H(21A)-C(21B)-H(21B)	109.5
C(25A)-H(25C)	0.9600	C(19)-C(21B)-H(21C)	109.5
C(26A)-C(27A)	1.534(9)	H(21A)-C(21B)-H(21C)	109.5
C(26A)-H(26D)	0.9800	H(21B)-C(21B)-H(21C)	109.5
C(27A)-H(27D)	0.9600	C(23)-C(22B)-H(22A)	109.5
C(27A)-H(27E)	0.9600	C(23)-C(22B)-H(22B)	109.5
C(27A)-H(27F)	0.9600	H(22A)-C(22B)-H(22B)	109.5
C(21A)-H(21D)	0.9600	C(23)-C(22B)-H(22C)	109.5
C(21A)-H(21E)	0.9600	H(22A)-C(22B)-H(22C)	109.5
C(21A)-H(21F)	0.9600	H(22B)-C(22B)-H(22C)	109.5
C(22A)-H(22D)	0.9600	C(25A)-C(23A)-C(24A)	122.9(11)
C(22A)-H(22E)	0.9600	C(25A)-C(23A)-Si(1)	124.3(8)

C(22A)-H(22F)	0.9600	C(24A)-C(23A)-Si(1)	107.6(9)
C(23B)-C(25B)	1.357(12)	C(25A)-C(23A)-H(23B)	97.6
C(23B)-C(24B)	1.39(2)	C(24A)-C(23A)-H(23B)	97.6
C(23B)-H(23C)	0.9800	Si(1)-C(23A)-H(23B)	97.6
C(24B)-H(24G)	0.9600	C(23A)-C(24A)-H(24D)	109.5
C(24B)-H(24H)	0.9600	C(23A)-C(24A)-H(24E)	109.5
C(24B)-H(24I)	0.9600	H(24D)-C(24A)-H(24E)	109.5
C(25B)-H(25D)	0.9600	C(23A)-C(24A)-H(24F)	109.5
C(25B)-H(25E)	0.9600	H(24D)-C(24A)-H(24F)	109.5
C(25B)-H(25F)	0.9600	H(24E)-C(24A)-H(24F)	109.5
C(26B)-C(27B)	1.50(3)	C(23A)-C(25A)-H(25A)	109.5
C(26B)-H(26E)	0.9800	C(23A)-C(25A)-H(25B)	109.5
C(27B)-H(27G)	0.9600	H(25A)-C(25A)-H(25B)	109.5
C(27B)-H(27H)	0.9600	C(23A)-C(25A)-H(25C)	109.5
C(27B)-H(27I)	0.9600	H(25A)-C(25A)-H(25C)	109.5
		H(25B)-C(25A)-H(25C)	109.5
C(16)-Si(1)-C(25)	107.71(17)	C(27A)-C(26A)-C(31)	108.3(5)
C(16)-Si(1)-C(23)	107.05(16)	C(27A)-C(26A)-Si(2)	112.8(4)
C(25)-Si(1)-C(23)	110.36(19)	C(31)-C(26A)-Si(2)	113.8(3)
C(16)-Si(1)-C(23A)	106.7(2)	C(27A)-C(26A)-H(26D)	107.2
C(25)-Si(1)-C(23A)	121.0(4)	C(31)-C(26A)-H(26D)	107.2
C(23)-Si(1)-C(23A)	103.3(4)	Si(2)-C(26A)-H(26D)	107.2
C(16)-Si(1)-C(23B)	106.9(4)	C(26A)-C(27A)-H(27D)	109.5
C(25)-Si(1)-C(23B)	99.6(5)	C(26A)-C(27A)-H(27E)	109.5
C(23)-Si(1)-C(23B)	124.2(6)	H(27D)-C(27A)-H(27E)	109.5

C(18)-Si(2)-C(26A)	108.69(18)	C(26A)-C(27A)-H(27F)	109.5
C(18)-Si(2)-C(28)	105.97(15)	H(27D)-C(27A)-H(27F)	109.5
C(26A)-Si(2)-C(28)	115.8(2)	H(27E)-C(27A)-H(27F)	109.5
C(18)-Si(2)-C(19)	108.28(16)	C(19)-C(21A)-H(21D)	109.5
C(26A)-Si(2)-C(19)	109.2(3)	C(19)-C(21A)-H(21E)	109.5
C(28)-Si(2)-C(19)	108.61(19)	H(21D)-C(21A)-H(21E)	109.5
C(18)-Si(2)-C(26B)	99.9(6)	C(19)-C(21A)-H(21F)	109.5
C(28)-Si(2)-C(26B)	100.1(6)	H(21D)-C(21A)-H(21F)	109.5
C(19)-Si(2)-C(26B)	131.5(7)	H(21E)-C(21A)-H(21F)	109.5
C(6)-C(1)-C(2)	119.3(3)	C(23)-C(22A)-H(22D)	109.5
C(6)-C(1)-H(1)	120.4	C(23)-C(22A)-H(22E)	109.5
C(2)-C(1)-H(1)	120.4	H(22D)-C(22A)-H(22E)	109.5
N(3)-C(2)-C(1)	124.0(3)	C(23)-C(22A)-H(22F)	109.5
N(3)-C(2)-H(2)	118.0	H(22D)-C(22A)-H(22F)	109.5
C(1)-C(2)-H(2)	118.0	H(22E)-C(22A)-H(22F)	109.5
C(2)-N(3)-C(4)	117.0(3)	C(25B)-C(23B)-C(24B)	119.5(12)
N(3)-C(4)-C(5)	123.4(3)	C(25B)-C(23B)-Si(1)	118.9(8)
N(3)-C(4)-C(14)	116.5(3)	C(24B)-C(23B)-Si(1)	118.5(11)
C(5)-C(4)-C(14)	120.0(3)	C(25B)-C(23B)-H(23C)	95.8
C(4)-C(5)-C(6)	117.2(3)	C(24B)-C(23B)-H(23C)	95.8
C(4)-C(5)-C(7)	119.5(3)	Si(1)-C(23B)-H(23C)	95.8
C(6)-C(5)-C(7)	123.3(3)	C(23B)-C(24B)-H(24G)	109.5
C(1)-C(6)-C(5)	119.0(3)	C(23B)-C(24B)-H(24H)	109.5
C(1)-C(6)-H(6)	120.5	H(24G)-C(24B)-H(24H)	109.5
C(5)-C(6)-H(6)	120.5	C(23B)-C(24B)-H(24I)	109.5
C(11)-C(7)-C(8)	117.2(3)	H(24G)-C(24B)-H(24I)	109.5
C(11)-C(7)-C(5)	123.2(3)	H(24H)-C(24B)-H(24I)	109.5
C(8)-C(7)-C(5)	119.5(3)	C(23B)-C(25B)-H(25D)	109.5

N(9)-C(8)-C(7)	122.8(3)	C(23B)-C(25B)-H(25E)	109.5
N(9)-C(8)-C(13)	117.1(3)	H(25D)-C(25B)-H(25E)	109.5
C(7)-C(8)-C(13)	120.1(3)	C(23B)-C(25B)-H(25F)	109.5
C(10)-N(9)-C(8)	116.9(3)	H(25D)-C(25B)-H(25F)	109.5
N(9)-C(10)-C(12)	124.6(3)	H(25E)-C(25B)-H(25F)	109.5
N(9)-C(10)-H(10)	117.7	C(27B)-C(26B)-C(31)	113.1(18)
C(12)-C(10)-H(10)	117.7	C(27B)-C(26B)-Si(2)	111.7(15)
C(12)-C(11)-C(7)	119.4(3)	C(31)-C(26B)-Si(2)	98.9(11)
C(12)-C(11)-H(11)	120.3	C(27B)-C(26B)-H(26E)	110.9
C(7)-C(11)-H(11)	120.3	C(31)-C(26B)-H(26E)	110.9
C(11)-C(12)-C(10)	119.1(3)	Si(2)-C(26B)-H(26E)	110.9
C(11)-C(12)-H(12)	120.5	C(26B)-C(27B)-H(27G)	109.5
C(10)-C(12)-H(12)	120.5	C(26B)-C(27B)-H(27H)	109.5
C(14)-C(13)-C(8)	120.4(2)	H(27G)-C(27B)-H(27H)	109.5
C(14)-C(13)-C(17)	120.3(3)	C(26B)-C(27B)-H(27I)	109.5
C(8)-C(13)-C(17)	119.2(2)	H(27G)-C(27B)-H(27I)	109.5
		H(27H)-C(27B)-H(27I)	109.5

Supplementary Table 23. Anisotropic displacement parameters (Å2x 103) for 7ca'. The anisotropic displacement factor exponent takes the form:-2 2[h2 a*2U11 + ... + 2 h k a* b* U12]

	**U11**	**U22**	**U33**	**U23**	**U13**	**U12**
Si(1)	63(1)	42(1)	50(1)	-1(1)	5(1)	5(1)
Si(2)	47(1)	40(1)	43(1)	-9(1)	4(1)	10(1)
C(1)	36(2)	68(2)	71(2)	-42(2)	-7(2)	8(2)
C(2)	48(2)	53(2)	64(2)	-24(2)	-15(2)	14(2)
N(3)	45(2)	46(1)	53(2)	-16(1)	-10(1)	9(1)
C(4)	33(2)	42(2)	43(2)	-17(1)	-6(1)	0(1)
C(5)	31(2)	50(2)	41(2)	-19(1)	-3(1)	-5(1)
C(6)	33(2)	69(2)	52(2)	-30(2)	-1(1)	-5(2)
C(7)	36(2)	46(2)	41(2)	-13(1)	-2(1)	-11(1)
C(8)	39(2)	38(2)	42(2)	-10(1)	-6(1)	-5(1)
N(9)	58(2)	38(1)	56(2)	-5(1)	-7(1)	-4(1)
C(10)	75(3)	43(2)	60(2)	0(2)	-11(2)	-12(2)
C(11)	46(2)	66(2)	46(2)	-13(2)	3(2)	-18(2)

C(12)	68(2)	59(2)	55(2)	-1(2)	1(2)	-24(2)
C(13)	35(2)	37(1)	37(2)	-12(1)	-5(1)	0(1)
C(14)	34(2)	40(2)	37(2)	-11(1)	-4(1)	-2(1)
C(15)	48(2)	41(2)	40(2)	-11(1)	-1(1)	4(1)
C(16)	59(2)	50(2)	48(2)	-13(2)	3(2)	8(2)
C(17)	47(2)	37(2)	40(2)	-11(1)	-4(1)	1(1)
C(18)	53(2)	43(2)	43(2)	-12(1)	-3(2)	8(1)
C(19)	68(3)	60(2)	100(3)	-16(2)	22(2)	-5(2)
C(20)	61(3)	93(3)	107(4)	-34(3)	17(2)	-19(2)
C(23)	74(3)	72(2)	51(2)	-1(2)	1(2)	-12(2)
C(24)	114(4)	113(4)	75(3)	-20(3)	-12(3)	-40(3)
C(25)	82(3)	83(3)	54(2)	1(2)	-5(2)	-22(2)
C(26)	157(5)	113(4)	88(4)	-57(3)	4(4)	-14(4)
C(27)	101(4)	166(6)	121(5)	-3(4)	-38(4)	-41(4)
C(28)	51(2)	44(2)	52(2)	-11(1)	0(2)	12(1)
C(29)	78(3)	88(3)	56(2)	-7(2)	-17(2)	6(2)
C(30)	105(3)	43(2)	68(2)	-11(2)	17(2)	-11(2)
C(31)	86(3)	63(2)	55(2)	-22(2)	5(2)	15(2)
C(21B)	74(10)	47(9)	98(12)	-17(10)	-10(11)	-26(8)
C(22B)	80(5)	97(9)	68(5)	11(5)	1(4)	-32(6)
C(23A)	63(5)	51(3)	62(4)	-3(3)	-13(4)	7(3)
C(24A)	75(9)	41(4)	92(7)	1(5)	-6(8)	0(6)
C(25A)	124(10)	61(6)	65(6)	-25(4)	-13(6)	26(6)
C(26A)	50(3)	50(3)	42(2)	-11(2)	-2(2)	0(2)
C(27A)	62(4)	106(5)	57(4)	-27(3)	-9(3)	-5(4)
C(21A)	70(4)	56(3)	112(6)	0(3)	-10(4)	-16(3)
C(22A)	64(8)	92(13)	84(10)	2(10)	3(7)	-25(10)
C(23B)	71(7)	58(5)	51(5)	-12(4)	-18(5)	16(5)
C(24B)	94(10)	68(7)	92(7)	14(6)	17(9)	29(7)
C(25B)	68(6)	61(6)	95(7)	-21(5)	-10(5)	23(5)
C(26B)	61(6)	59(6)	48(5)	-18(5)	-8(5)	10(5)
C(27B)	65(9)	71(9)	63(9)	-15(7)	-5(8)	14(8)

Supplementary Table 24. Hydrogen coordinates (x 104) and isotropic displacement parameters (Å2x 10 3) for 7ca'.

	**x**	**y**	**z**	**U(eq)**
H(1)	14416	3226	4969	66
H(2)	13239	2360	6180	64
H(6)	13577	5102	4426	59
H(10)	9541	9458	4417	72
H(11)	12753	6893	4029	62
H(12)	11669	8759	3649	74
H(19)	2504	7085	6838	94
H(19A)	2609	6737	6836	94

H(20A)	1229	7302	8249	129
H(20B)	322	6671	7736	129
H(20C)	807	7943	7408	129
H(23)	4843	3555	8592	81
H(23A)	5023	3618	8468	81
H(24A)	5358	3101	7347	148
H(24B)	3701	2627	7783	148
H(24C)	5280	1787	7733	148
H(25)	7515	2792	9943	89
H(26A)	7344	4741	9980	173
H(26B)	5994	4483	9485	173
H(26C)	7591	5061	9059	173
H(27A)	10354	2569	9587	195
H(27B)	9964	3539	10077	195
H(27C)	10354	3871	9162	195
H(28)	2965	9549	6818	61
H(29A)	4466	8499	5584	113
H(29B)	3304	9630	5454	113
H(29C)	2598	8459	5900	113
H(30A)	5568	10152	6917	110
H(30B)	5049	10663	6071	110
H(30C)	6325	9594	6210	110
H(31A)	4001	9682	8208	103
H(31B)	3978	9006	9082	103
H(31C)	2601	8851	8567	103
H(31D)	3250	9581	8191	103
H(31E)	3970	9263	9008	103
H(31F)	3007	8362	8732	103
H(21A)	3684	5875	6638	107
H(21B)	2674	5182	7378	107
H(21C)	4433	5551	7448	107
H(22A)	4936	1128	9184	127
H(22B)	3317	1938	9156	127
H(22C)	4633	2050	9715	127
H(23B)	7531	1168	8169	72
H(24D)	8728	643	9640	107
H(24E)	8577	-333	9180	107
H(24F)	7004	417	9416	107
H(25A)	10083	1901	7603	125
H(25B)	9195	850	7475	125
H(25C)	10507	659	8084	125
H(26D)	4496	7249	8655	57
H(27D)	7277	7431	8274	110
H(27E)	6724	8204	8893	110

H(27F)	6985	8770	7997	110
H(21D)	4164	5268	7501	121
H(21E)	2294	5096	7769	121
H(21F)	3385	5544	8308	121
H(22D)	4741	1573	9496	124
H(22E)	3324	2537	9308	124
H(22F)	4852	2788	9695	124
H(23C)	9894	1754	8978	72
H(24G)	8500	693	9733	140
H(24H)	9672	-64	9242	140
H(24I)	7813	183	9087	140
H(25D)	9994	1962	7598	113
H(25E)	10291	638	7937	113
H(25F)	11346	1521	8178	113
H(26E)	6147	9103	7907	67
H(27G)	5616	6978	8963	102
H(27H)	6753	7899	9094	102
H(27I)	7256	7221	8417	102

Supplementary Table 25. Torsion angles [°] for 7ca'.

C(6)-C(1)-C(2)-N(3)	2.4(5)
C(1)-C(2)-N(3)-C(4)	-1.7(5)
C(2)-N(3)-C(4)-C(5)	-0.8(4)
C(2)-N(3)-C(4)-C(14)	176.2(3)
N(3)-C(4)-C(5)-C(6)	2.4(4)
C(14)-C(4)-C(5)-C(6)	-174.5(2)
N(3)-C(4)-C(5)-C(7)	-178.4(3)
C(14)-C(4)-C(5)-C(7)	4.6(4)
C(2)-C(1)-C(6)-C(5)	-0.6(4)
C(4)-C(5)-C(6)-C(1)	-1.6(4)
C(7)-C(5)-C(6)-C(1)	179.3(3)
C(4)-C(5)-C(7)-C(11)	178.1(3)
C(6)-C(5)-C(7)-C(11)	-2.7(4)
C(4)-C(5)-C(7)-C(8)	-2.3(4)
C(6)-C(5)-C(7)-C(8)	176.8(3)
C(11)-C(7)-C(8)-N(9)	-1.2(4)
C(5)-C(7)-C(8)-N(9)	179.3(3)
C(11)-C(7)-C(8)-C(13)	177.6(3)
C(5)-C(7)-C(8)-C(13)	-1.9(4)
C(7)-C(8)-N(9)-C(10)	0.7(4)
C(13)-C(8)-N(9)-C(10)	-178.1(3)
C(8)-N(9)-C(10)-C(12)	0.2(5)
C(8)-C(7)-C(11)-C(12)	0.8(4)
C(5)-C(7)-C(11)-C(12)	-179.7(3)

C(7)-C(11)-C(12)-C(10)	0.0(5)
N(9)-C(10)-C(12)-C(11)	-0.6(6)
N(9)-C(8)-C(13)-C(14)	-177.2(3)
C(7)-C(8)-C(13)-C(14)	4.0(4)
N(9)-C(8)-C(13)-C(17)	5.8(4)
C(7)-C(8)-C(13)-C(17)	-173.0(3)
C(8)-C(13)-C(14)-C(15)	-179.6(3)
C(17)-C(13)-C(14)-C(15)	-2.7(4)
C(8)-C(13)-C(14)-C(4)	-1.7(4)
C(17)-C(13)-C(14)-C(4)	175.3(2)
N(3)-C(4)-C(14)-C(13)	-179.8(2)
C(5)-C(4)-C(14)-C(13)	-2.7(4)
N(3)-C(4)-C(14)-C(15)	-1.8(4)
C(5)-C(4)-C(14)-C(15)	175.4(3)
C(16)-Si(1)-C(23)-C(22A)	-155(2)
C(25)-Si(1)-C(23)-C(22A)	-38(2)
C(23B)-Si(1)-C(23)-C(22A)	79(2)
C(16)-Si(1)-C(23)-C(24)	54.9(3)
C(25)-Si(1)-C(23)-C(24)	171.9(3)
C(23A)-Si(1)-C(23)-C(24)	-57.5(4)
C(23B)-Si(1)-C(23)-C(24)	-70.3(6)
C(16)-Si(1)-C(23)-C(22B)	177.5(11)
C(25)-Si(1)-C(23)-C(22B)	-65.5(11)
C(23A)-Si(1)-C(23)-C(22B)	65.2(11)
C(16)-Si(1)-C(25)-C(26)	59.0(4)
C(23)-Si(1)-C(25)-C(26)	-57.5(4)
C(23A)-Si(1)-C(25)-C(26)	-178.1(4)
C(23B)-Si(1)-C(25)-C(26)	170.3(6)
C(16)-Si(1)-C(25)-C(27)	-66.2(4)
C(23)-Si(1)-C(25)-C(27)	177.3(3)
C(23A)-Si(1)-C(25)-C(27)	56.7(5)
C(23B)-Si(1)-C(25)-C(27)	45.1(6)
C(18)-Si(2)-C(28)-C(29)	64.3(3)
C(26A)-Si(2)-C(28)-C(29)	-175.1(3)
C(19)-Si(2)-C(28)-C(29)	-51.8(3)
C(26B)-Si(2)-C(28)-C(29)	167.8(7)
C(18)-Si(2)-C(28)-C(30)	-58.0(3)
C(26A)-Si(2)-C(28)-C(30)	62.6(3)
C(19)-Si(2)-C(28)-C(30)	-174.1(2)
C(26B)-Si(2)-C(28)-C(30)	45.5(7)
C(18)-Si(2)-C(26A)-C(27A)	27.6(6)
C(28)-Si(2)-C(26A)-C(27A)	-91.5(5)
C(19)-Si(2)-C(26A)-C(27A)	145.5(5)
C(18)-Si(2)-C(26A)-C(31)	151.5(4)

C(28)-Si(2)-C(26A)-C(31)	32.4(5)
C(19)-Si(2)-C(26A)-C(31)	-90.5(4)

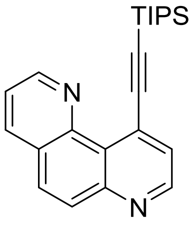

7da'

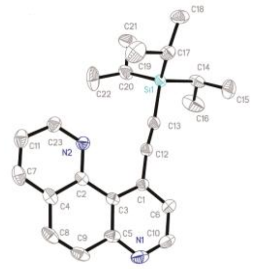

Supplementary Table 26. Crystal data and structure refinement for 7da'.

Identification code	7da'
Empirical formula	C23 H28 N2 Si
Formula weight	360.56
Temperature	193(2) K
Wavelength	1.34139 Å
Crystal system	Orthorhombic
Space group	Pbca
Unit cell dimensions	a = 15.5894(9) Å α = 90 °.
	b = 8.0980(5) Å β = 90 °.
	c = 33.5692(19) Å γ = 90 °.
Volume	4237.9(4) $Å^3$
Z	8
Density (calculated)	1.130 Mg/m^3
Absorption coefficient	0.660 mm^{-1}
F(000)	1552
Crystal size	? x ? x ? mm^3
Theta range for data collection	3.366 to 53.996 °.
Index ranges	-18<=h<=13, -9<=k<=9, -37<=l<=40
Reflections collected	29520
Independent reflections	3888 [R(int) = 0.0472]
Completeness to theta = 53.594 °	99.9 %
Refinement method	Full-matrix least-squares on F^2
Data / restraints / parameters	3888 / 0 / 241
Goodness-of-fit on F^2	0.951

Final R indices [I>2sigma(I)]	R1 = 0.0542, wR2 = 0.1548
R indices (all data)	R1 = 0.0695, wR2 = 0.1725
Extinction coefficient	n/a
Largest diff. peak and hole	0.907 and -0.440 e.Å^{-3}

Supplementary Table 27. Atomic coordinates (x 10^4) and equivalent isotropic displacement parameters (Å^{2}x 10^3) for 7da'. U(eq) is defined as one third of the trace of the orthogonalized U^{ij} tensor.

	x	y	z	U(eq)
C(1)	5847(1)	5026(3)	4056(1)	42(1)
C(2)	4516(1)	5591(3)	4469(1)	41(1)
C(3)	5402(2)	5895(2)	4360(1)	40(1)
C(4)	4155(2)	6444(3)	4796(1)	50(1)
C(5)	5863(2)	7114(3)	4571(1)	49(1)
C(6)	6690(2)	5468(3)	3982(1)	54(1)
C(7)	3296(2)	6106(3)	4894(1)	60(1)
C(8)	4660(2)	7620(3)	5008(1)	60(1)
C(9)	5468(2)	7948(3)	4901(1)	61(1)
C(10)	7074(2)	6717(4)	4201(1)	63(1)
C(11)	2848(2)	4988(4)	4674(1)	60(1)
C(12)	5508(1)	3689(3)	3824(1)	43(1)
C(13)	5338(2)	2548(3)	3609(1)	48(1)
C(14)	5824(2)	41(4)	2990(1)	60(1)
C(15)	6188(2)	1332(6)	2706(1)	95(1)
C(16)	6539(3)	-667(6)	3247(1)	111(2)
C(17)	4113(2)	1916(3)	2958(1)	56(1)
C(18)	3786(2)	825(4)	2620(1)	74(1)
C(19)	3373(2)	2734(5)	3190(1)	98(1)
C(20)	4547(2)	-793(4)	3636(1)	70(1)
C(21)	4211(3)	-2326(4)	3430(1)	90(1)
C(22)	3981(3)	-248(5)	3967(1)	103(1)
C(23)	3257(2)	4208(3)	4359(1)	54(1)
N(1)	6691(2)	7531(3)	4488(1)	62(1)
N(2)	4062(1)	4482(2)	4254(1)	45(1)
Si(1)	4911(1)	882(1)	3293(1)	42(1)

Supplementary Table 28. Bond lengths [Å] and angles [°] for 7da'.

C(1)-C(6)	1.384(3)	C(9)-C(8)-H(8F)	119.4
C(1)-C(3)	1.420(3)	C(4)-C(8)-H(8F)	119.4
C(1)-C(12)	1.434(3)	C(8)-C(9)-C(5)	121.2(2)
C(2)-N(2)	1.352(3)	C(8)-C(9)-H(9G)	119.4
C(2)-C(4)	1.414(3)	C(5)-C(9)-H(9G)	119.4
C(2)-C(3)	1.451(3)	N(1)-C(10)-C(6)	124.1(3)
C(3)-C(5)	1.411(3)	N(1)-C(10)-H(10H)	117.9
C(4)-C(7)	1.405(4)	C(6)-C(10)-H(10H)	117.9
C(4)-C(8)	1.427(4)	C(7)-C(11)-C(23)	118.8(2)
C(5)-N(1)	1.363(3)	C(7)-C(11)-H(11J)	120.6
C(5)-C(9)	1.436(4)	C(23)-C(11)-H(11J)	120.6
C(6)-C(10)	1.387(4)	C(13)-C(12)-C(1)	170.8(2)
C(6)-H(6B)	0.9500	C(12)-C(13)-Si(1)	171.5(2)
C(7)-C(11)	1.362(4)	C(16)-C(14)-C(15)	109.9(3)
C(7)-H(7D)	0.9500	C(16)-C(14)-Si(1)	112.5(2)
C(8)-C(9)	1.336(4)	C(15)-C(14)-Si(1)	111.8(2)
C(8)-H(8F)	0.9500	C(16)-C(14)-H(14I)	107.4
C(9)-H(9G)	0.9500	C(15)-C(14)-H(14I)	107.4
C(10)-N(1)	1.311(4)	Si(1)-C(14)-H(14I)	107.4
C(10)-H(10H)	0.9500	C(14)-C(15)-H(15A)	109.5
C(11)-C(23)	1.387(4)	C(14)-C(15)-H(15B)	109.5
C(11)-H(11J)	0.9500	H(15A)-C(15)-H(15B)	109.5
C(12)-C(13)	1.203(3)	C(14)-C(15)-H(15C)	109.5
C(13)-Si(1)	1.840(2)	H(15A)-C(15)-H(15C)	109.5
C(14)-C(16)	1.522(4)	H(15B)-C(15)-H(15C)	109.5
C(14)-C(15)	1.522(5)	C(14)-C(16)-H(16A)	109.5
C(14)-Si(1)	1.879(3)	C(14)-C(16)-H(16B)	109.5
C(14)-H(14I)	1.0000	H(16A)-C(16)-H(16B)	109.5
C(15)-H(15A)	0.9800	C(14)-C(16)-H(16C)	109.5
C(15)-H(15B)	0.9800	H(16A)-C(16)-H(16C)	109.5
C(15)-H(15C)	0.9800	H(16B)-C(16)-H(16C)	109.5
C(16)-H(16A)	0.9800	C(18)-C(17)-C(19)	112.0(3)
C(16)-H(16B)	0.9800	C(18)-C(17)-Si(1)	114.16(19)
C(16)-H(16C)	0.9800	C(19)-C(17)-Si(1)	112.6(2)
C(17)-C(18)	1.525(4)	C(18)-C(17)-H(17E)	105.7
C(17)-C(19)	1.541(4)	C(19)-C(17)-H(17E)	105.7

C(17)-Si(1)	1.876(2)	Si(1)-C(17)-H(17E)	105.7
C(17)-H(17E)	1.0000	C(17)-C(18)-H(18A)	109.5
C(18)-H(18A)	0.9800	C(17)-C(18)-H(18B)	109.5
C(18)-H(18B)	0.9800	H(18A)-C(18)-H(18B)	109.5
C(18)-H(18C)	0.9800	C(17)-C(18)-H(18C)	109.5
C(19)-H(19A)	0.9800	H(18A)-C(18)-H(18C)	109.5
C(19)-H(19B)	0.9800	H(18B)-C(18)-H(18C)	109.5
C(19)-H(19C)	0.9800	C(17)-C(19)-H(19A)	109.5
C(20)-C(22)	1.484(4)	C(17)-C(19)-H(19B)	109.5
C(20)-C(21)	1.515(4)	H(19A)-C(19)-H(19B)	109.5
C(20)-Si(1)	1.868(3)	C(17)-C(19)-H(19C)	109.5
C(20)-H(20K)	1.0000	H(19A)-C(19)-H(19C)	109.5
C(21)-H(21A)	0.9800	H(19B)-C(19)-H(19C)	109.5
C(21)-H(21B)	0.9800	C(22)-C(20)-C(21)	112.3(3)
C(21)-H(21C)	0.9800	C(22)-C(20)-Si(1)	115.2(2)
C(22)-H(22A)	0.9800	C(21)-C(20)-Si(1)	114.8(2)
C(22)-H(22B)	0.9800	C(22)-C(20)-H(20K)	104.3
C(22)-H(22C)	0.9800	C(21)-C(20)-H(20K)	104.3
C(23)-N(2)	1.322(3)	Si(1)-C(20)-H(20K)	104.3
C(23)-H(23C)	0.9500	C(20)-C(21)-H(21A)	109.5
C(6)-C(1)-C(3)	117.7(2)	C(20)-C(21)-H(21B)	109.5
C(6)-C(1)-C(12)	116.6(2)	H(21A)-C(21)-H(21B)	109.5
C(3)-C(1)-C(12)	125.7(2)	C(20)-C(21)-H(21C)	109.5
N(2)-C(2)-C(4)	122.1(2)	H(21A)-C(21)-H(21C)	109.5
N(2)-C(2)-C(3)	118.45(19)	H(21B)-C(21)-H(21C)	109.5
C(4)-C(2)-C(3)	119.5(2)	C(20)-C(22)-H(22A)	109.5
C(5)-C(3)-C(1)	117.3(2)	C(20)-C(22)-H(22B)	109.5
C(5)-C(3)-C(2)	118.5(2)	H(22A)-C(22)-H(22B)	109.5
C(1)-C(3)-C(2)	124.18(19)	C(20)-C(22)-H(22C)	109.5
C(7)-C(4)-C(2)	117.8(2)	H(22A)-C(22)-H(22C)	109.5
C(7)-C(4)-C(8)	122.6(2)	H(22B)-C(22)-H(22C)	109.5
C(2)-C(4)-C(8)	119.6(2)	N(2)-C(23)-C(11)	124.3(3)
N(1)-C(5)-C(3)	123.6(2)	N(2)-C(23)-H(23C)	117.9
N(1)-C(5)-C(9)	116.6(2)	C(11)-C(23)-H(23C)	117.9
C(3)-C(5)-C(9)	119.9(2)	C(10)-N(1)-C(5)	117.1(2)
C(1)-C(6)-C(10)	120.1(2)	C(23)-N(2)-C(2)	117.7(2)
C(1)-C(6)-H(6B)	119.9	C(13)-Si(1)-C(20)	106.72(12)
C(10)-C(6)-H(6B)	119.9	C(13)-Si(1)-C(17)	104.99(12)

C(11)-C(7)-C(4)	119.4(2)	C(20)-Si(1)-C(17)	119.52(14)
C(11)-C(7)-H(7D)	120.3	C(13)-Si(1)-C(14)	107.71(12)
C(4)-C(7)-H(7D)	120.3	C(20)-Si(1)-C(14)	107.53(14)
C(9)-C(8)-C(4)	121.3(2)	C(17)-Si(1)-C(14)	109.79(12)

Supplementary Table 29. Anisotropic displacement parameters (Å^{2}x 10^3) for 7da'.

The anisotropic displacement factor exponent takes the form:-2 ^{2}[h^2 a*^{2}U^{11} + ... + 2 h k a* b* U^{12}]

	**U^{11}**	U^{22}	U^{33}	U^{23}	U^{13}	U^{12}
C(1)	44(1)	37(1)	45(1)	2(1)	-2(1)	-3(1)
C(2)	52(1)	33(1)	37(1)	2(1)	-4(1)	9(1)
C(3)	52(1)	31(1)	37(1)	3(1)	-6(1)	1(1)
C(4)	70(2)	39(1)	40(1)	4(1)	0(1)	17(1)
C(5)	67(2)	33(1)	48(1)	4(1)	-14(1)	-5(1)
C(6)	49(1)	52(1)	61(2)	3(1)	2(1)	-7(1)
C(7)	70(2)	61(2)	48(1)	8(1)	12(1)	28(1)
C(8)	98(2)	40(1)	42(1)	-7(1)	-4(1)	17(1)
C(9)	100(2)	35(1)	49(1)	-6(1)	-18(1)	1(1)
C(10)	54(1)	61(2)	75(2)	7(2)	-8(1)	-20(1)
C(11)	49(1)	73(2)	59(2)	12(1)	9(1)	15(1)
C(12)	44(1)	42(1)	43(1)	-1(1)	6(1)	1(1)
C(13)	52(1)	46(1)	45(1)	-4(1)	5(1)	0(1)
C(14)	58(2)	69(2)	54(1)	-19(1)	-13(1)	18(1)
C(15)	65(2)	138(3)	84(2)	-6(2)	30(2)	0(2)
C(16)	90(2)	133(4)	110(3)	-34(3)	-42(2)	60(3)
C(17)	45(1)	56(2)	68(2)	8(1)	-5(1)	3(1)
C(18)	69(2)	79(2)	73(2)	13(2)	-30(2)	-10(2)
C(19)	60(2)	103(3)	131(3)	4(2)	1(2)	32(2)
C(20)	95(2)	65(2)	50(2)	4(1)	-1(1)	-15(2)
C(21)	140(3)	59(2)	71(2)	17(2)	-29(2)	-39(2)
C(22)	145(3)	76(2)	88(2)	24(2)	62(2)	10(2)
C(23)	44(1)	60(2)	58(1)	2(1)	1(1)	2(1)
N(1)	72(1)	49(1)	65(1)	5(1)	-19(1)	-17(1)
N(2)	41(1)	46(1)	48(1)	-3(1)	1(1)	1(1)
Si(1)	51(1)	40(1)	36(1)	-1(1)	0(1)	-2(1)

Supplementary Table 30. Hydrogen coordinates (x 10^4) and isotropic displacement parameters ($Å^2$x 10^3) for 7da'.

	x	y	z	U(eq)
H(6B)	7006	4914	3780	65
H(7D)	3031	6653	5112	72
H(8F)	4418	8180	5230	72
H(9G)	5787	8748	5046	73
H(10H)	7650	7001	4139	76
H(11J)	2267	4746	4735	72
H(14I)	5592	-882	2824	72
H(15A)	6398	2279	2860	143
H(15B)	5737	1697	2523	143
H(15C)	6662	850	2554	143
H(16A)	6980	-1158	3076	167
H(16B)	6303	-1517	3424	167
H(16C)	6794	218	3408	167
H(17E)	4430	2841	2826	68
H(18A)	3462	-103	2732	110
H(18B)	4274	403	2467	110
H(18C)	3413	1474	2445	110
H(19A)	3052	3466	3012	147
H(19B)	3608	3379	3412	147
H(19C)	2990	1879	3294	147
H(20K)	5084	-1170	3772	84
H(21A)	4049	-3150	3630	135
H(21B)	4658	-2782	3257	135
H(21C)	3707	-2038	3270	135
H(22A)	3435	146	3858	155
H(22B)	4260	648	4114	155
H(22C)	3874	-1178	4147	155
H(23C)	2938	3425	4209	65
